# Diversity of Dictyostelid Cellular Slime Molds, Including Two Species New to Science, in Forest Soils of Changbai Mountain, China

**DOI:** 10.1128/spectrum.02402-22

**Published:** 2022-10-03

**Authors:** Yue Zou, Jiangan Hou, Songning Guo, Changtian Li, Zhuang Li, Steven L. Stephenson, Igor N. Pavlov, Pu Liu, Yu Li

**Affiliations:** a Engineering Research Center of Edible and Medicinal Fungi, Ministry of Education, Jilin Agricultural University, Changchun, China; b Shandong Provincial Key Laboratory for Biology of Vegetable Diseases and Insect Pests, College of Plant Protection, Shandong Agricultural University, Tai’an, China; c Department of Biological Sciences, University of Arkansas, Fayetteville, Arkansas, USA; d Laboratory of Reforestation, Mycology and Plant Pathology, V.N. Sukachev Institute of Forest SB RAS, Krasnoyarsk, Russia; e Department of Chemical Technology of Wood and Biotechnology, Reshetnev Siberian State University of Science and Technology, Krasnoyarsk, Russia; University of Huddersfield

**Keywords:** dictyostelids, new species, environmental adaptability, *Dictyostelium robusticaule*, *Heterostelium recretum*, differences of diversity

## Abstract

Dictyostelid cellular slime molds (dictyostelids) are protists that are common inhabitants of most soils, where they feed upon bacteria. Changbai Mountain is the highest mountain in northeast China. Soil samples collected on Changbai Mountain yielded 11 isolates representing six species of dictyostelid samples. Two of these species (*Dictyostelium robusticaule* and *Heterostelium recretum*) were found to be new to science, based on morphology, SSU rDNA sequences, and an ATPase subunit 1 gene (*atp1*) phylogeny. The present study also demonstrated that the increased accuracy and lower costs associated with the use of *atp1* sequences make them a complement of SSU rDNA sequences for identifying dictyostelids. Changbai Mountain is characterized by a higher diversity of dictyostelids than indicated by the few previous reports. Moreover, the data for Changbai Mountain, compared with comparable data for Taiwan, suggest that differences in diversity at the family level are possibly related to latitude. Mixed broadleaf-conifer forests produced more isolates and species than broadleaf forests at the same elevation and also had the highest species richness, which indicates an effect of vegetation on dictyostelids. However, the pattern of slightly decreasing diversity with increasing elevation in dictyostelids was also apparent.

**IMPORTANCE** *Dictyostelium robusticaule* and *Heterostelium recretum* are two new species of dictyostelids reported in this study. The potential use of *atp1* sequences is a complement of SSU rDNA sequences for the identifying dictyostelids. A pattern of slightly decreasing diversity with increasing elevation in dictyostelids was observed, with the conditions that exist at lower elevations apparently more suitable for dictyostelids, whereas differences of diversity observed at the family level are possibly related to latitude.

## INTRODUCTION

Dictyostelid cellular slime molds (dictyostelids) are a monophyletic group of sorocarp-forming social amoebae belonging to the supergroup Amoebozoa (1, 2). Dictyostelids feed upon bacteria and yeasts, and these organisms are common in the soil and leaf litter layer of forests and other types of vegetation. Some species even occur in animal dung, which is the substrate from which dictyostelids were first recorded (3, 4). These organisms are a normal component of the microbiota of soils and maintain the natural balance in the soil microhabitat (5). The first species of dictyostelid (*Dictyostelium mucoroides*) was described in 1869 by Brefeld, and representatives of three other genera—*Coenonia*, *Polysphondylium*, and *Acytostelium*—were described later (6–10). Sheikh et al. (9) reclassified the dictyostelids into 12 genera (*Cavenderia*, *Acytostelium*, *Rostrostelium*, *Heterostelium*, *Tieghemostelium*, *Hagiwaraea*, *Raperostelium*, *Speleostelium*, *Dictyostelium*, *Polysphondylium*, *Coremiostelium*, and *Synstelium*) and this taxonomic treatment is the one in current use.

Dictyostelids have both asexual and sexual life cycles, but asexual spores produced on sorocarps are the primary means of reproduction (11–13). Dictyostelid spores can be carried on and dispersed by water, earthworms, pillbugs, humans, *Drosophila* flies, rodents, terrestrial salamanders, and ground-feeding migratory songbirds (3, 14–19). Dictyostelids are widespread throughout the world and have been shown to display biogeographical patterns (2).

**FIG 1:**
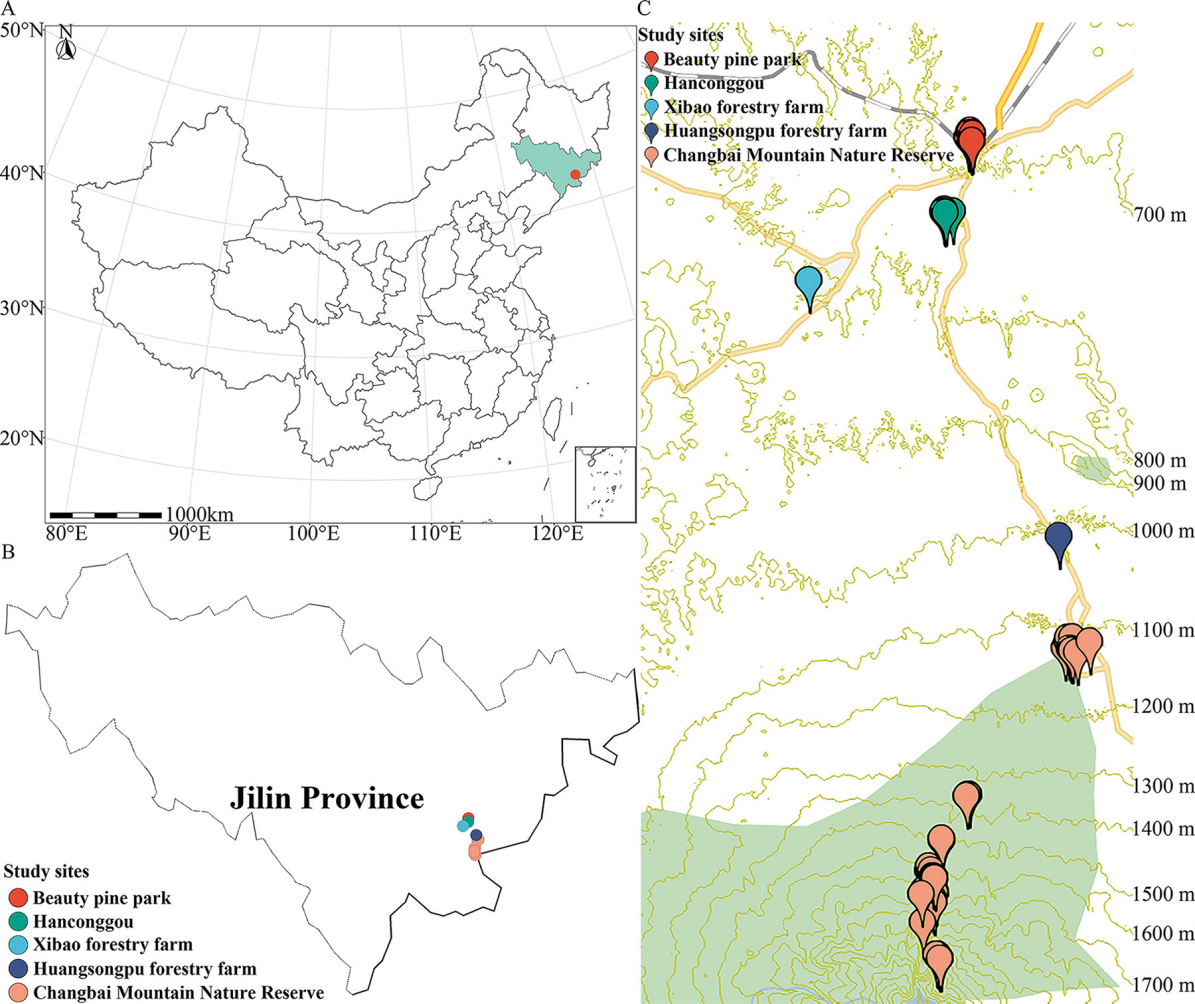
Maps of sampling sites on Changbai Mountain in Jilin Province of China. (A) Location of Changbai Mountain in Jilin Province with respect to the rest of China. (B) Map showing the collecting localities in Jilin Province. (C) Contour plot (ovitalMap, v9.1.6) showing the sampling sites on Changbai Mountain.

Changbai Mountain (127°40' to 128°16' E and 41°35' to 42°25' N; elevation 2,749 m), the highest mountain in the northeast of China, is located in the southeast position of Jilin Province, which places it near the border between China and North Korea (Fig. 1). Changbai Mountain has a wide range of soils, vegetation, and climatic conditions along the gradient of elevation (20). However, only three previous papers have reported data on the dictyostelids of Changbai Mountain (21–23). In the study reported herein, the dictyostelids of Changbai Mountain were studied during the period of 2016 to 2017 in an effort to document these organisms more completely.

Tang et al. (24) indicated that elevation, rather than vegetation, is the primary predictor of rhizosphere microbial community structure in areas of alpine tundra on Changbai Mountain. Liu et al. (25) reported that at higher elevations on the Qinghai–Tibet Plateau, dictyostelids appear to be relatively common. As such, in the present study soil samples were collected over a wide range of elevations (675 to 2,636 m) to obtain new isolates of dictyostelids and to determine their patterns of diversity at high elevations on Changbai Mountain.

For most of their history, dictyostelids have been distinguished on the basis of morphological features, but more recently this has been replaced with the use of small subunit rRNA (SSU rDNA) sequences, with some studies also based on the internal transcribed spacer (ITS) region of rDNA (26–30). In the previously mentioned study, Sheikh et al. (9) subdivided dictyostelids into the 12 genera based on a SSU rDNA molecular phylogeny. However, preliminary experiments carried out in our laboratory have indicated that neither SSU nor ITS sequences produce a clearly resolved molecular phylogeny in some genera such as *Dictyostelium*. Consequently, we evaluated data obtained from the ATPase subunit 1 gene (*atp1*) of mitochondrial genome sequences to determine its possible use in studies of dictyostelids.

## RESULTS

### Taxonomy and molecular phylogeny.

**FIG 2:**
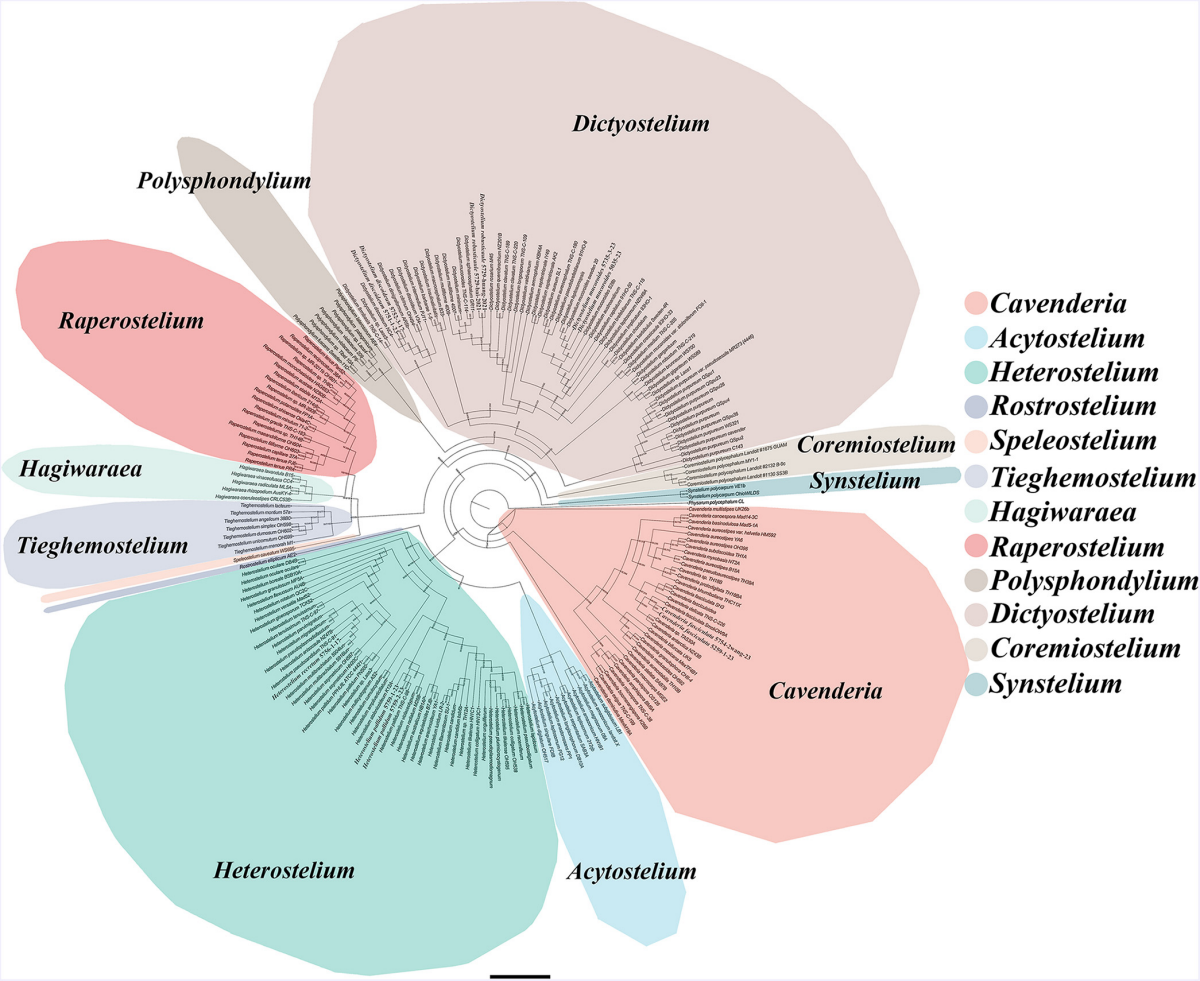
Phylogenetic tree of all known dictyostelids based on SSU rDNA. Numbers in parentheses are SH-aLRT support (%)/ultrafast bootstrap support (%). Newly generated sequences are indicated in bold. Scale bar = 9.0.

**FIG 3:**
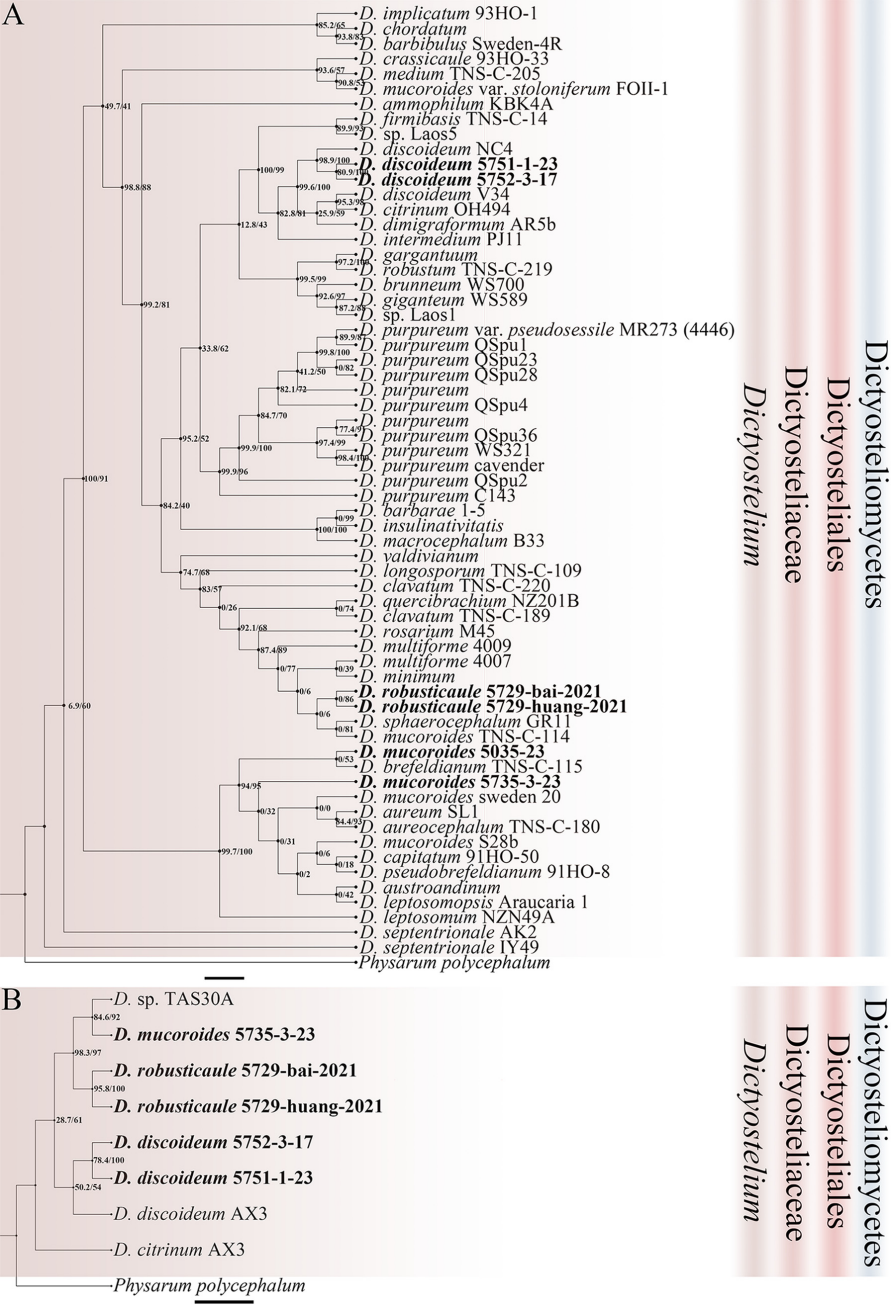
Phylogenetic trees of *Dictyostelium* sequences. (A) Position of *Dictyostelium* species of this paper in the SSU rDNA phylogeny. (B) Position of *Dictyostelium* species of this paper in the *atp1* phylogeny. Numbers in parentheses are SH-aLRT support (%)/ultrafast bootstrap support (%). Newly generated sequences are indicated in bold. Scale bar: A = 2.0; B = 3.0.

**FIG 4:**
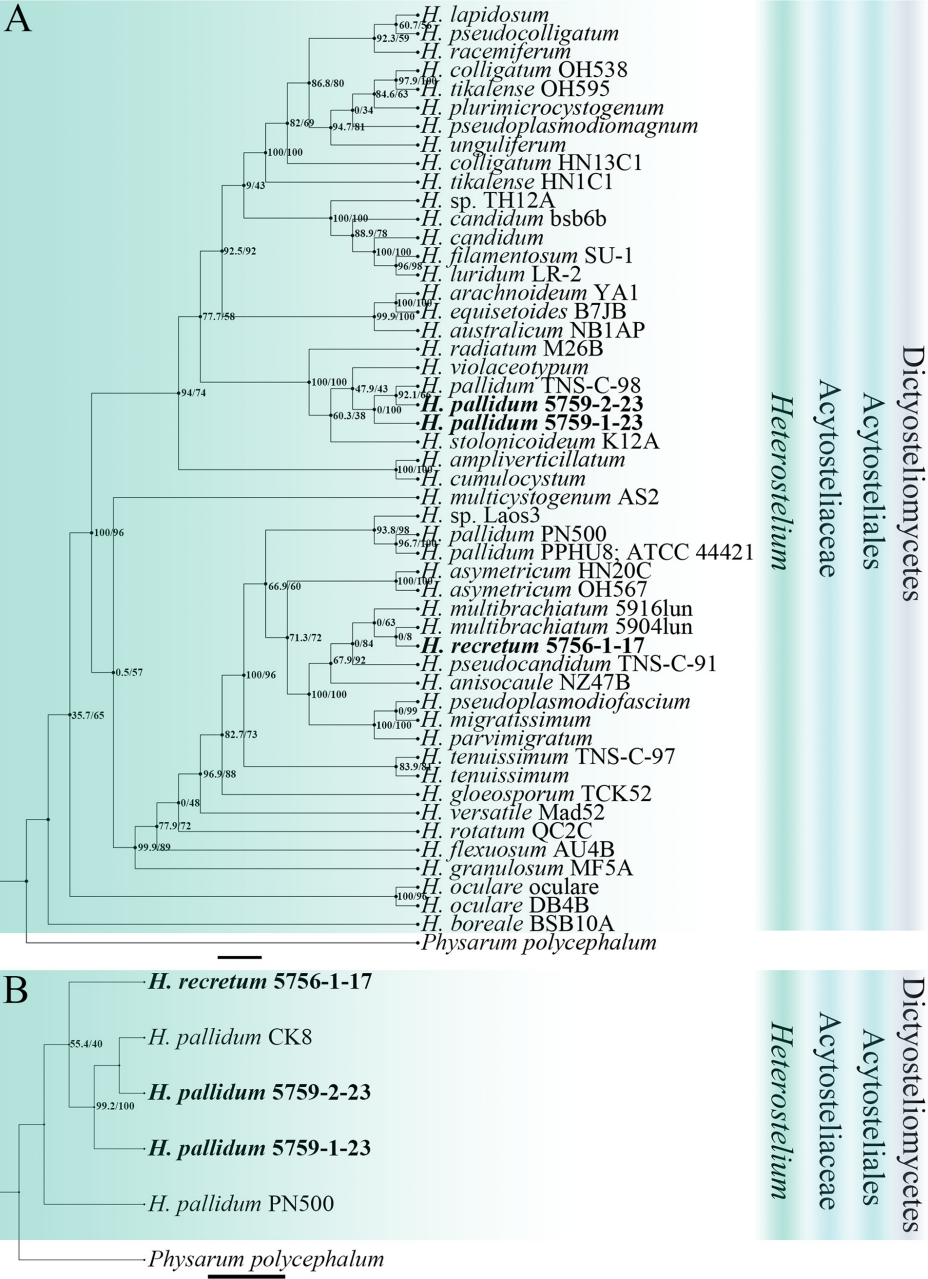
Phylogenetic trees of *Heterostelium* sequences. (A) Position of *Heterostelium* species of this paper in the SSU rDNA phylogeny. (B) Position of *Heterostelium* species of this paper in the *atp1* phylogeny. Numbers in parentheses are SH-aLRT support (%)/ultrafast bootstrap support (%). Newly generated sequences are indicated in bold. Scale bar: A = 2.0; B = 3.0.

**FIG 5:**
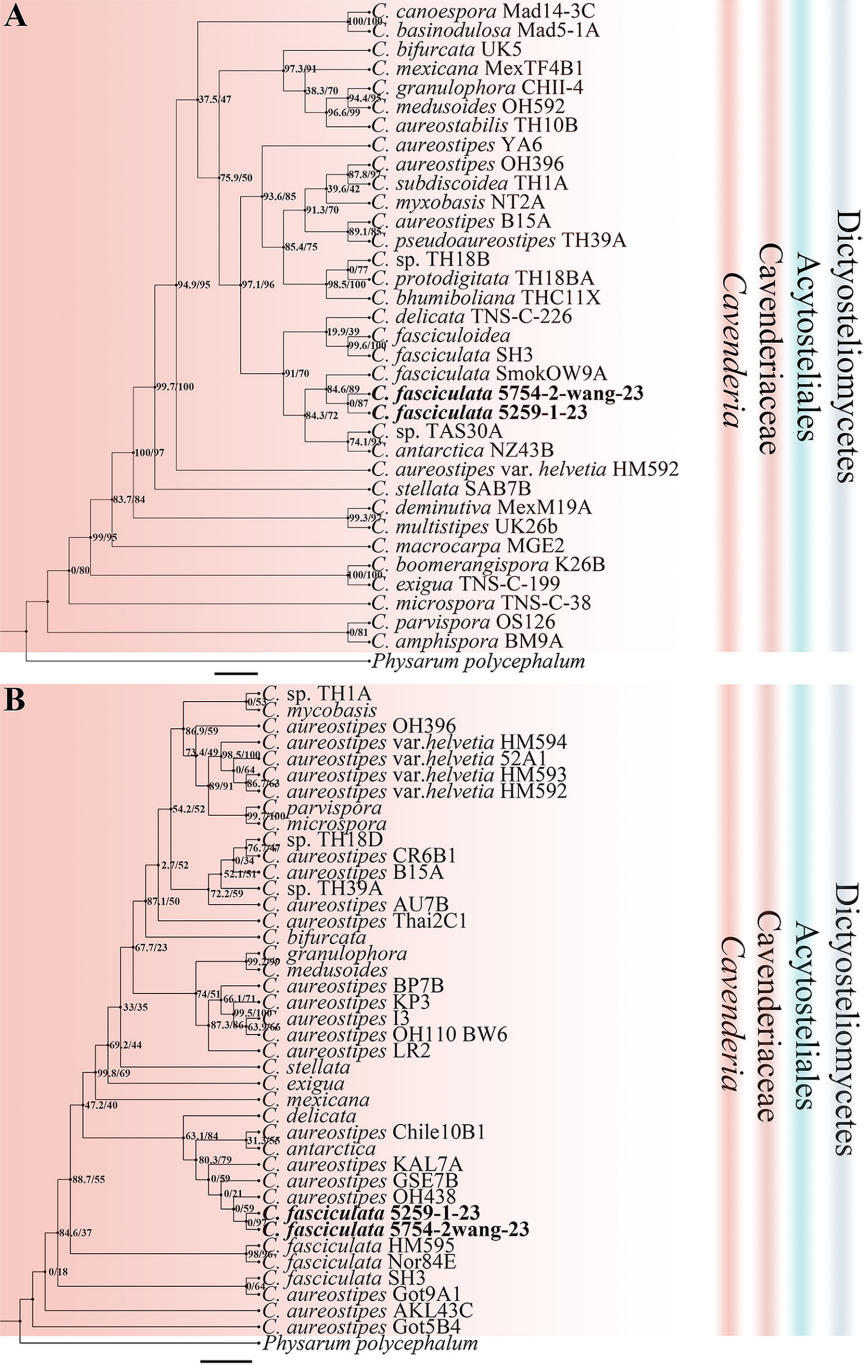
Phylogenetic trees of *Cavenderia* sequences. (A) Position of *Cavenderia* species of this paper in the SSU rDNA phylogeny. (B) Position of *Cavenderia* species of this paper in the *atp1* phylogeny. Numbers in parentheses are SH-aLRT support (%)/ultrafast bootstrap support (%). Newly generated sequences are indicated in bold. Scale bar: A = 2.0; B = 4.0.

Eleven isolates representing six species (Table 1) were obtained from the soil samples collected on Changbai Mountain in 2016 to 2017, including two species (Dictyostelium discoideum and *D. mucoroides*) common in Jilin Province (31). *Cavenderia faciculata* and *Heterostelium pallidum* were not previously known from Jilin Province. Two species new to science (*D. robusticaule* and *H. recretum*) were isolated. These are described below. Phylogenetic studies of the SSU rDNA data revealed the two new species are members of genera *Dictyostelium* and *Heterostelium* (Fig. 2–4; Table S2), based on the concepts of Schaap et al. (32), Romeralo et al. (29), and Sheikh et al. (9). Phylogenetic studies of the *atp1* data showed that the three species D. discoideum, *D. mucoroides*, and *D. robusticaule* clustered within the clade containing the other *Dictyostelium* sequences, whereas the sequences for *H. pallidum* and *H. recretum* clustered with other species of *Heterostelium*. The other species (*C. faciculata*) clustered with the other *Cavenderia* sequences (Fig. 5; Table S3).

**TABLE 1:** Six species of dictyostelids isolated from soil samples collected on Changbai Mountain in current study

Species	Strain no.	Soil no.	Location	Coordinates	Elevation (m)	Habitat
Dictyostelium discoideum	HMJAU MR302 (strain 5751-1-23)	5751	Hanconggou	42°24'06''93N, 128°05'52''43E	776	Broadleaf forest
	HMJAU MR302 (strain 5752-3-17)	5752	Hanconggou	42°24'07''72N, 128°05'52''14E	774	Broadleaf forest
*Dictyostelium mucoroides*	HMJAU MR303 (strain 5035-23)	5035	Changbai Mountain Nature Reserve	42°03'14''09.6N, 128°03'59''52.8E	2,038	Tundra
	HMJAU MR303 (strain 5735-3-23)	5735	Changbai Mountain Nature Reserve	42°04'13''76N, 128°03'56''95E	1,720	Mixed broadleaf-conifer forest
*Dictyostelium robusticaule* ^*a*^	HMJAU MR305 (strain 5729-huang-2021)	5729	Xibao forestry farm	42°22'17''84N, 128°00'13''74E	828	Mixed broadleaf-conifer forest
	HMJAU MR305 (strain 5729-bai-2021)	5729	Xibao forestry farm	42°22'17''84N, 128°00'13''74E	828	Mixed broadleaf-conifer forest
*Cavenderia fasciculata*	HMJAU MR313 (strain 5754-2wang-23)	5754	Hanconggou	42°24'11''92N, 128°05'53''34E	787	Mixed broadleaf-conifer forest
	HMJAU MR313 (strain 5259-1-23)	5759	Changbai Mountain Nature Reserve	42°06'25''54N, 128°05'34''13E	1,438	Coniferous forest
*Heterostelium pallidum*	HMJAU MR314 (strain 5759-1-23)	5759	Changbai Mountain Nature Reserve	42°06'25''54N, 128°05'34''13E	1,438	Coniferous forest
	HMJAU MR314 (strain 5759-2-23)	5759	Changbai Mountain Nature Reserve	42°06'25''54N, 128°05'34''13E	1,438	Coniferous forest
*Heterostelium recretum* ^*a*^	HMJAU MR315 (strain 5756-1-17)	5756	Hanconggou	42°24'06''81N, 128°05'54''86E	768	Mixed broadleaf-conifer forest

a New species obtained in this study.

**FIG 6:**
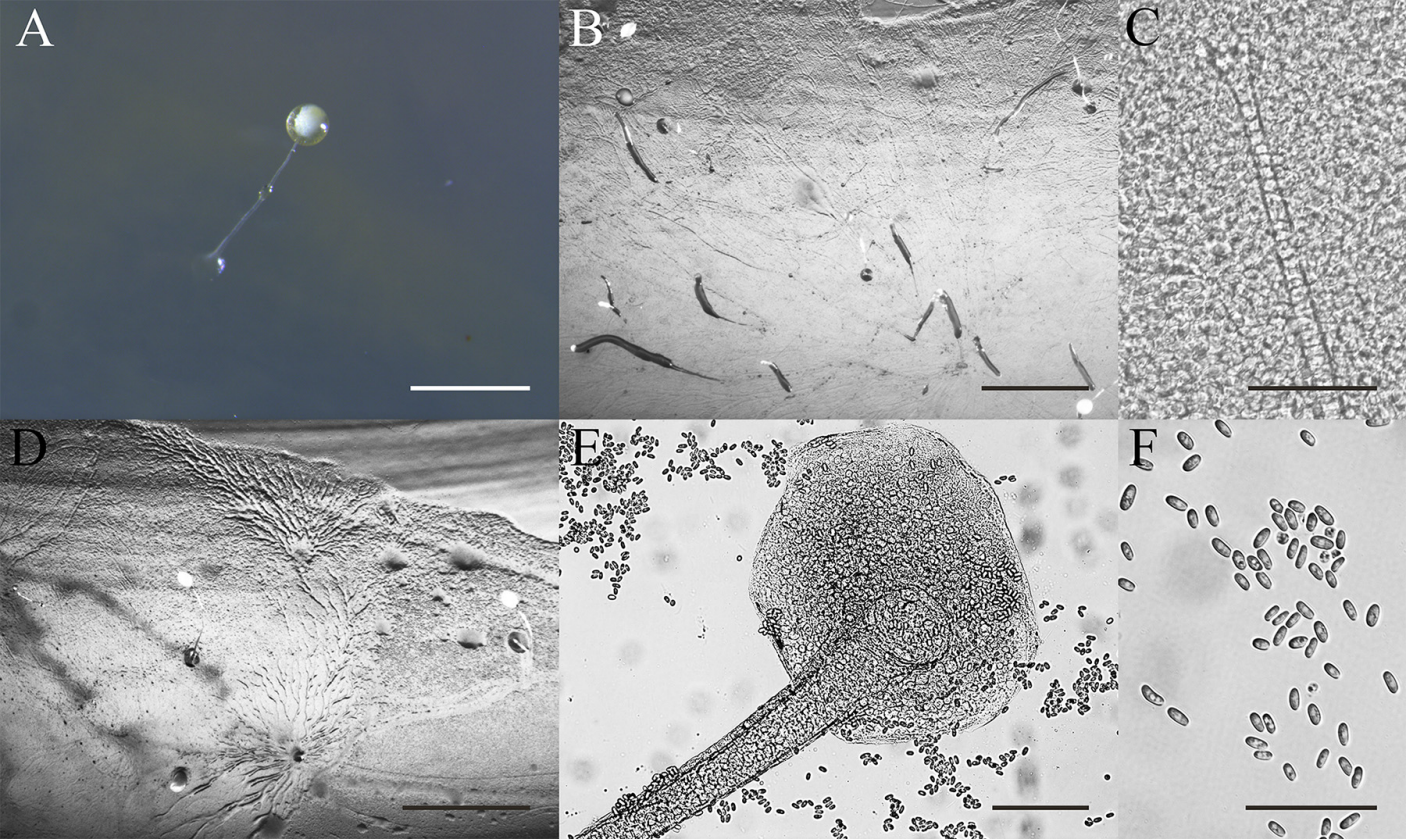
Morphological features of Dictyostelium discoideum. (A) Sorocarps. (B) Pseudoplasmodia. (C) Tip of sorophore. (D) Aggregations. (E) Base of sorophore. (F) Spores. Scale bars: A = 1 mm; B and D = 2 mm; C = 60 μm; E = 100 μm; F = 50 μm.

Dictyostelium discoideum Raper, J. Agr. Res. 50: 135–147 (1935) (Fig. 6).

When cultured at 17°C or 23°C on nonnutrient agar with Escherichia coli, sorocarps erect, solitary, strong, 3.1 to 3.3 mm high. Sorophores consisting of several tiers of cells. Tips capitate with one or two tiers of cells, 8.0 to 13.9 μm wide. Bases with basal disks, consisting of several tiers of cells, 51.8 to 78.9 μm wide. Sori globose, golden yellow, 323 to 535 μm in diameter. Spores are elliptical-oblong, 8.8 to 10.9 × 3.7 to 4.8 μm, rarely longer and up to 13.4 to 14.1 × 4.6 to 5.0 μm, without polar granules but sometimes with dispersed solid granules. Aggregations are radiate and pseudoplasmodia migrate without stalks.

Cultures examined: HMJAU MR302 (strain 5751-1-23, strain 5752-3-17) was isolated from two soil samples (5751, elevation 776 m, coordinates 42°24'06''93 N, 128°05'52''43 E; 5752, elevation 774 m, coordinates 42°24'07''72 N, 128°05'52''14 E), collected on September 17, 2017 in a broadleaf deciduous forest, China, Jilin Province, Hanconggou.

GenBank accession number: SSU sequence: MT125951 (5751-1-23), MT125952 (5752-3-17), *atp1* sequence: MZ318395 (5751-1-23), MZ318394 (5752-3-17).

Known distribution: China, India, Japan, numerous localities throughout North America, and Pakistan.

This species was isolated and cultured both at 23°C (5751-1-23) and 17°C (5752-3-17) and is a common species throughout the world as a model organism for research in each field of biology (33). This species tapers from the bases to tips and can be distinguished easily on the basis of the stout stalks, golden-yellow sori, and the basal disks (34).

**FIG 7:**
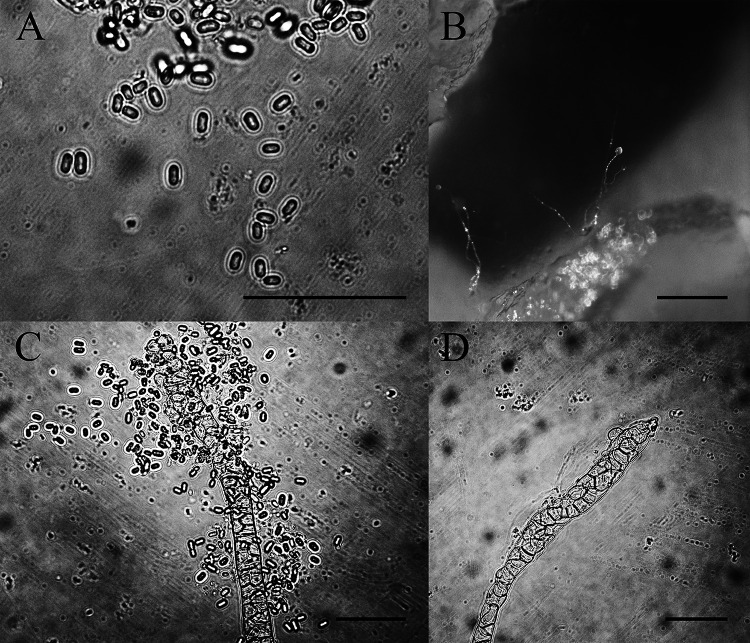
Morphological features of *Dictyostelium mucoroides*. (A) Spores. (B) Sorocarps. (C) Tip of sorophore. (D) Base of sorophore. Scale bars: A = 50 μm; B = 1 mm; C to D = 50 μm.

*Dictyostelium mucoroides* Bref., Abh. senckenb. naturforsch. Ges. 7: 85 (1869) (Fig. 7).

When cultured at 23°C on nonnutrient agar with E. coli, sorocarps erect or prone, solitary or clustered, without branches or occasionally with a few branches, not phototropic, 1.6 to 7.0 mm high. Stalks consisting of several tiers of cells are 15.5 to 30.8 μm wide. Tips capitate or clavate, 16.6 μm wide. Bases clavate, 21.2 to 31.8 μm wide. Sori globose or lemon-shaped, white at first and then darker with time until reaching a cream color and are 110 to 214 μm in diameter. Spores are oblong, 5.9 to 8.4 × 3.0 to 4.1 μm, without polar granules. Aggregations are mound-like and pseudoplasmodia are not migrating without sorophore formation. Microcysts and macrocysts are absent.

Cultures examined: HMJAU MR303 (strain 5035-23, strain 5735-3-23) was isolated from two soil samples from China, Jilin Province, Changbai Mountain, one (5035, elevation 2,038 m, coordinates 42°03'236'' N, 128°03'998'' E) collected on August 29, 2016 in mountain tundra, the other (5735, elevation 1,720 m, coordinates 42°04'13''76 N, 128°03'56''95 E) collected on September 16, 2017 in a mixed broadleaf-conifer forest.

GenBank accession number: SSU sequence: MW660746 (5035-23), MW660747 (5735-3-23), *atp1* sequence: MZ318393 (5735-3-23).

Known distribution: United States, Canada, China, Denmark, Germany, India, Japan, Nepal, Netherlands, New Zealand, Oman, Switzerland, United Kingdom, and Uganda.

This species was isolated and cultured at 23°C and is a common species throughout the world. There has been considerable research on *Dictyostelium mucoroides* as the first dictyostelid to be described (6, 13, 35, 36). In one report, *D. mucoroides* was described as having basal disks (36), but this was not observed in the present study.

**FIG 8:**
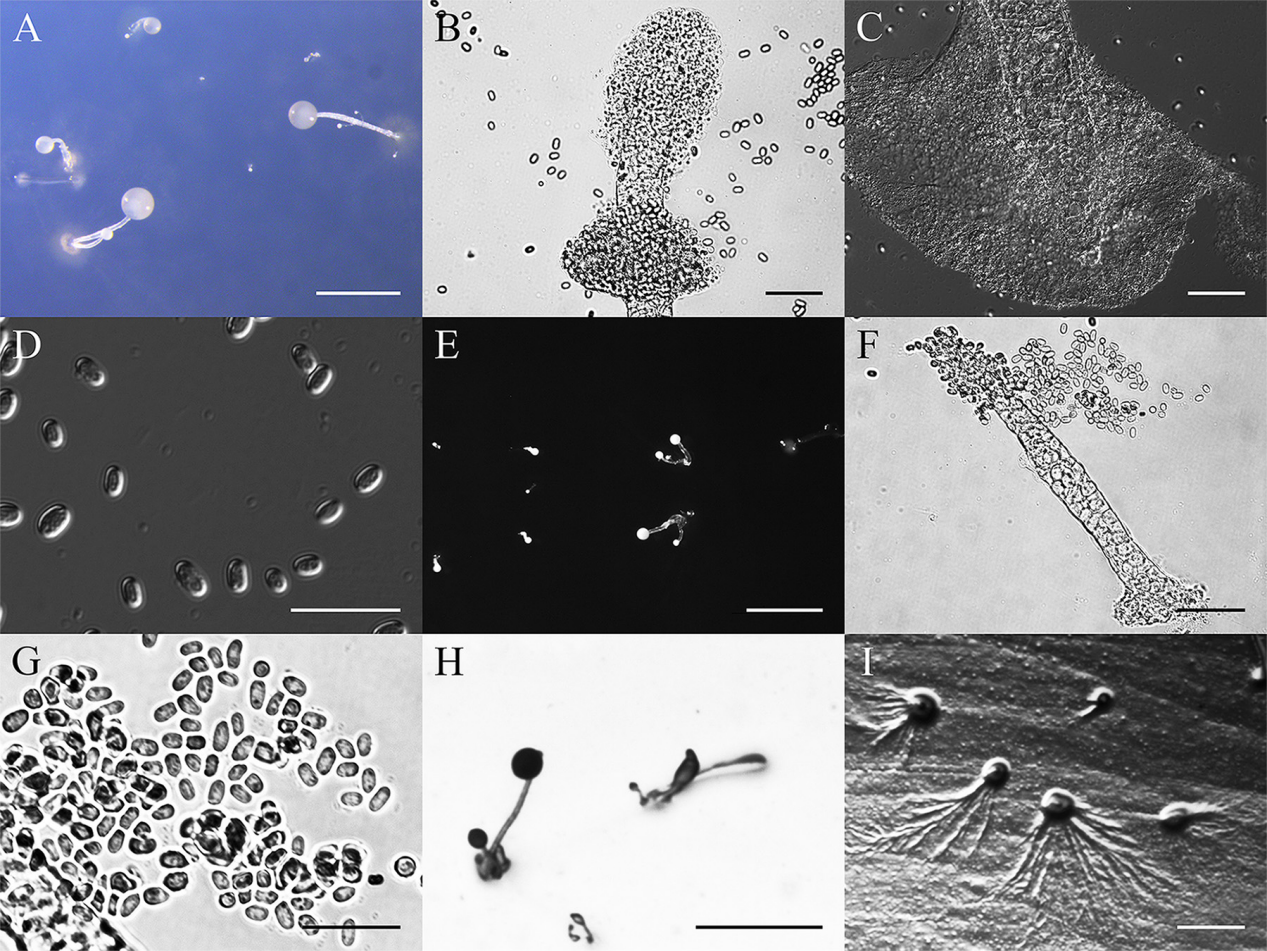
Morphological features of *Dictyostelium robusticaule*. (A) Larger sorocarps. (B) Tip of bigger sorophore. (C) Base of larger sorophore. (D) Larger spores. (E) Smaller sorocarps. (F) Smaller sorophore. (G) Smaller spores. (H) Pseudoplasmodia. (I) Aggregations. Scale bars: A = 1 mm; B and C = 40 μm; D = 20 μm; E = 1 mm; F = 40 μm; G = 20 μm; H to I = 1 mm.

*Dictyostelium robusticaule* Y. Li, P. Liu et Y. Zou, sp. nov. (Fig. 8).

MycoBank accession number: MB830534.

When cultured at 23°C on nonnutrient agar with E. coli, sorocarps solitary. Sorophores stout, generally erect, inclined, semierect or prone, unbranched or with one to five irregular branches near the base, branches of different sizes. There are two sizes of sorocarps, the larger ones are 0.3 to 2.4 mm in length, with very stout sorophores that are white or hyaline. Sorophores with obtuse tips and a collar, tips with many tiers of cells and are mostly 24.0 to 53.6 μm in diameter. Bases clavate, with many tiers of cells, 35.5 to 51.8 μm, always with slime around the base (basal disks). Sori globose, present white to off-white with abundant slime at first but over time some sori tend to dry out and spores remain strongly attached to one another; other sori do not dry out but tend to become yellowish in age and are 121 to 520 μm. Spores are elliptical or oblong, 5.0 to 7.2 × 3.2 to 4.7 μm with polar granules. The smaller sorocarps are 0.1 to 0.2 mm in length with tapering sorophores. Tips of the sorophores are obtuse with two tiers of cells, 7.7 to 10.0 μm in diameter. Slightly expanded bases have many tiers of cells, bases are clavate or obtuse, 19.3 to 23.3 μm in diameter. Sori are small, globose, hyaline or white, 27 to 47 μm in diameter. Slightly smaller spores in the small sori are 4.7 to 6.5 × 3.0 to 4.3 μm, with polar granules. Aggregations are radiate. This species belongs to the *Dictyostelium* clade in a SSU rDNA phylogeny.

Etymology: the name refers to the robust sorophores.

Holotype: HMJAU MR305 (strain 5729-huang-2021) was isolated from a soil sample of China, Jilin Province, Xibao forestry farm (5729, elevation 828 m, coordinates 42°22'17''84 N, 128°00'13''74 E) collected on September 17, 2017 in a mixed broadleaf-conifer forest.

GenBank accession number: SSU sequence: MW931856, *atp1* sequence: MZ318392.

Morphologically, *Dictyostelium robusticaule* is characterized by two types of sorocarps and the stout sorophores with a collar. It is similar to *D. gargantuum*, *D. multiforme*, *D. septentrionale*, and *D. sphaerocephalum* in the structure of the collar, but these species do not have polar granules in the spores except for *D. robusticaule*, and the two types of sorocarps are also absent in the latter species (13, 25, 37, 38).

Other specimens examined: HMJAU MR305 (strain 5729-bai-2021) was also examined and isolated from a soil sample of China, Jilin Province, Xibao forestry farm (5729, elevation 828 m, coordinates 42°22’17''84 N, 128°00’13''74 E) collected on September 17, 2017 in a mixed broadleaf-conifer forest.

GenBank accession number: SSU sequence: MW931857, *atp1* sequence: MZ318391.

**FIG 9:**
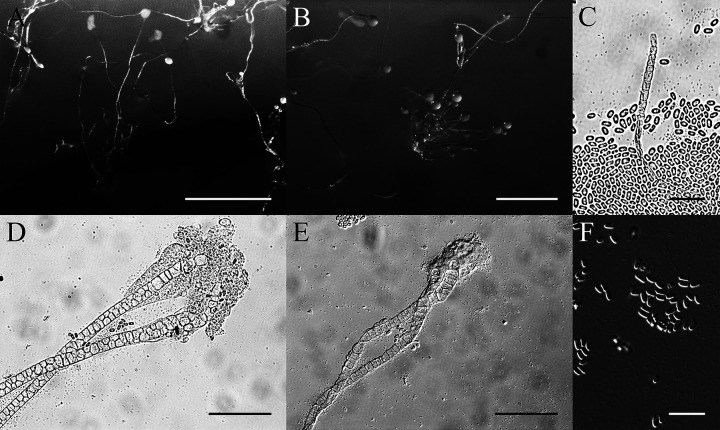
Morphological features of *Cavenderia fasciculata*. (A and B) Sorocarps. (C) Tip of sorophore. (D and E) Bases of sorophores. (F) Spores. Scale bars: A = 2 mm; B = 1 mm; C = 20 μm; D and E = 60 μm; F = 20 μm.

*Cavenderia fasciculata* (F. Traub et al.) S. Baldauf, S. Sheikh & Thulin, Protist 169:19 (2018) (Fig. 9).

Basionym. *Dictyostelium fasciculatum* F. Traub, H.R. Hohl & Cavender, Am. J. Bot. 68(2): 166 (1981).

When cultured at 23°C on nonnutrient agar with E. coli, sorocarps are solitary, clustered or gregarious, prostrate or semierect, occasionally with several irregular branches, 3.0 to 5.3 mm high. Stalks consist of several tiers of cells. Tips are obtuse, 12 to 18 μm in wide. Bases clavate, with several tiers of cells, 19 to 25 μm wide. Sori are globose, white, 145 to 200 μm in diameter. Spores are elliptical to oblong, 4.8 to 8.3 × 2.7 to 3.5 μm with polar granules. Aggregations are radiate. Microcysts and macrocysts are absent.

Cultures examined: HMJAU MR313 (strain 5754-2wang-23, strain 5259-1-23) was isolated from two soil samples, one of China, Jilin Province, Hanconggou (5754, elevation 787 m, coordinates 42°24'11''92 N, 128°05'53''34 E) collected on September 17, 2017 in mixed broadleaf-conifer forest, the other sample of China, Jilin Province, Changbai Mountain (5759, elevation 1,438 m, coordinates 42°06'25''54 N, 128°05'34''13 E) collected on September 18, 2017 in a coniferous forest.

GenBank accession number: SSU sequence: MW660750 (5754-2wang-23), MW660751 (5259-1-23), *atp1* sequence: MZ318390 (5754-2wang-23), MZ318389 (5259-1-23).

Known distribution: China, Denmark, France, New Zealand, Russia, Switzerland, and the United States.

*Cavenderia fasciculata* was isolated at 23°C in the present study. This species typically has clustered sorocarps, some with irregular branches near the bases, and spores with conspicuous polar granules (39).

**FIG 10:**
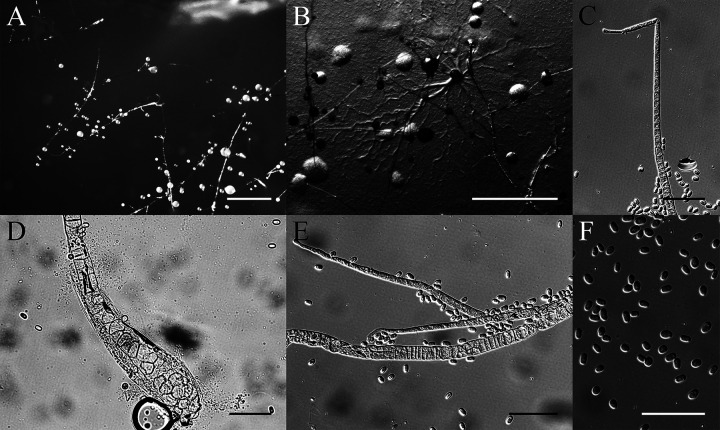
Morphological features of *Heterostelium pallidum*. (A) Sorocarps. (B) Aggregation. (C) Tip of sorophore. (D) Base of sorophore. (E) Branches of sorophore. (F) Spores. Scale bars: A and B = 1 mm; C to F = 40 μm.

*Heterostelium pallidum* (Olive) S. Baldauf, S. Sheikh & Thulin. Protist, 169: 18 (2018) (Fig. 10).

Basionym. *Polysphondylium pallidum* Olive, Proc. Amer. Acad. Arts & Sci. 37(12): 341 (1901).

When cultured at 23°C on nonnutrient agar with E. coli, sorocarps solitary or clustered, 2.5 to 5.9 mm in length, prone, prostrate, or semierect, with regular whorls of branches, some with secondary branches on the lower part. The secondary branches are also with regular whorls of branches. Sorophores consisting of one or two tiers of cells, tips clavate or acuminate with one tier of cells, 3.4 to 5.1 μm wide. Bases clavate with two tiers of cells, sometimes with a dense slime sheath, 18.3 to 27.2 μm wide. Sorophores have one to six whorls, each whorl with one to five branches; length is generally 174 to 420 μm. Sori are globose, white; terminal sori are larger than lateral sori. Terminal sori are 183 to 220 μm in diameter, and lateral sori are smaller at 99 to 153 μm diameter. Spores are oblong, 7.0 to 8.8 × 3.5 to 4.5 μm, with unconsolidated polar granules.

Cultures examined: HMJAU MR314 (strain 5759-1-23, strain 5759-2-23) was isolated from one soil sample of China, Jilin Province, Changbai Mountain (5759, elevation 1,438 m, coordinates 42°06'25''54 N, 128°05'34''13 E) collected on September 18, 2017 in a coniferous forest.

GenBank accession number: SSU sequence: MW660748 (5759-1-23), MW660749 (5759-2-23), *atp1* sequences: MZ318388 (5759-1-23), MZ318387 (5759-2-23).

Known distribution: United States, Canada, China, France, India, Indonesia, Italy, Japan, Liberia, Malaysia, Nepal, Philippines, and Tanzania.

*Heterostelium pallidum* was isolated at 23°C. This species has widespread distribution in the world. The longer terminal segment of the sorophores and secondary branches of whorls are both distinguishing features (40). *Heterostelium pallidum* is similar to *H. arachnoideum*, but the upper stalks of *H. arachnoideum* produce a delicate spiderweb-like trama, sometimes without sori. This feature distinguishes it from *H. pallidum* (41).

**FIG 11:**
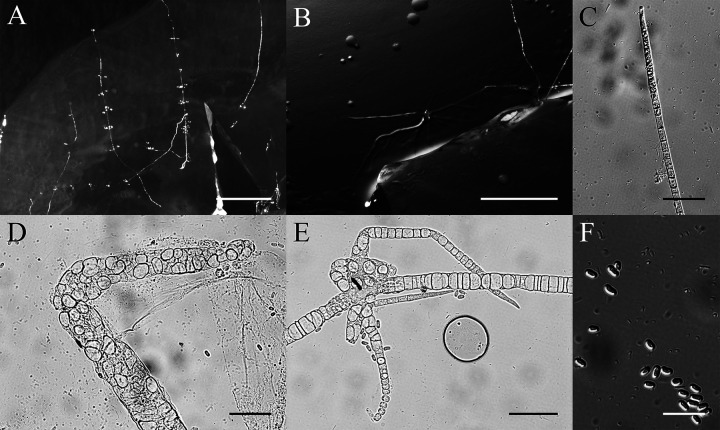
Morphological features of *Heterostelium recretum*. (A) Sorocarps. (B) Aggregation. (C) Tip of sorophore. (D) Base of sorophore. (E) Branches of sorophore. (F) Spores. Scale bars: A and B = 2 mm; C to E = 40 μm; F = 20 μm.

*Heterostelium recretum* Y. Li, P. Liu et Y. Zou, sp. nov. (Fig. 11).

MycoBank Accession Number. MB830541.

When cultured at 17°C on hay infusion agar and cultured at 23°C on nonnutrient agar with E. coli, sorocarps solitary or gregarious, semierect or prostrate, white, with many whorls of branches, and phototropic. Sorophores consisting of one or two tiers of cells except at the base, and are mostly 2.6 to 12.2 mm in length. Occasionally, terminal sori of prostrate-creeping sorocarps collapse and regrow, starting anew, sometimes growing up to 20.5 mm. Sorophores are slightly tapered from near the base to the tip. Bases clavate, consisting of several tiers of cells, 19.5 to 24.6 μm wide, sometimes with adherent dense layer of slime. Tips consisting of one tier of cells, acuminate, 6.0 to 9.4 μm wide. Sorophores commonly have two to eight whorls, sometimes up to 11 whorls when prostrate, each whorl with three to six branches. Branches are generally 121.5 to 235.0 μm in length. Nodes are regularly spaced, 522 to 788 μm, terminal segments are 478 to 712 μm long. Sori are white, semihyaline, globose. Terminal sori are 69 to 155 μm in diameter, lateral sori are 50 to 129 μm in diameter. Spores are elliptical, mostly 5.8 to 7.5 × 3.2 to 4.2 μm, with inconspicuous polar granules. Aggregations are radiate. This species belongs to the *Heterostelium* clade in a SSU rDNA phylogeny.

Etymology: The name *recretum* refers to the regrowing phenomenon of the sorophores.

Holotype: HMJAU MR315 (strain 5756-1-17) was isolated from a soil sample of China, Jilin Province, Hanconggou (5756, elevation 768 m, coordinates 42°24'06''81 N, 128°05'54''86 E) collected on September 17, 2017 in a mixed broadleaf-conifer forest.

GenBank accession number: SSU sequence: MW857293, *atp1* sequence: MZ318386.

This species is very similar to several other species. There are six species that cluster near this species in a SSU phylogenetic tree. These are *Heterostelium anisocaule*, *H. migratissimum*, *H. multibrachiatum*, *H. parvimigratum*, *H. pseudocandidum*, and *H. pseudoplasmodiofascium* (35, 36, 42, 43). In *H. anisocaule*, the number of branches in whorls, the number of nodes, and length of sorocarps are all less than in this species. *Heterostelium migratissimum* has shorter sorocarps and a shorter terminal segment, whereas *H. multibrachiatum* has shorter branches and a shorter terminal segment. Both *H. pseudocandidum* and *H. parvimigratum* have fewer nodes and shorter sorocarps, while *H. pseudoplasmodiofascium* has smaller sori and larger spores.

In terms of morphology, *Heterostelium stolonicoideum* has features similar to this species. Both of them regrow sorocarps after sori collapse, but this species has shorter branches, wider bases, and longer sorocarps (44).

### Key to dictyostelid species on Changbai Mountain.

The key given below is designed for identifying any of the species of dictyostelids known to occur on Changbai Mountain. Nomenclature for species other than those described herein follows Raper (13), Hagiwara (36), and Sheikh et al. (9).

- 1. Sorocarps with regular branches....................2
- 1. Sorocarps mostly unbranched but sometimes with irregular branches...................5
- 2. Sori pigmented violet/lavender/purple, often darkening with age.................*Polysphondylium violaceum*
- 2. Sori white-hyaline......................3
- 3. Terminal segments of sorophores often quite long with terminal sori usually minute or lacking.............................*Heterostelium candidum*
- 3. Terminal segments of sorophores not elongate; terminal sori comparatively large....................4
- 4. Longer sorophores have shorter branches and smaller terminal sori...........................*H. recretum*
- 4. Shorter sorophores have longer branches and larger terminal sori...........................*H. pallidum*
- 5. Stalk often has a basal support disk........................6
- 5. Stalk bases clavate without expanded............................8
- 6. Sorocarps gigantic (−10 mm or more)............................*Dictyostelium firmibasis*
- 6. Sorocarps medial (−3.5 mm)......................7
- 7. Sorocarps with two types, and the stout sorophore has a collar...........................*D. robusticaule*
- 7. Sorocarps have only one type, and sorophores without collars...........................D. discoideum
- 8. Polar granules absent, or PG (−)..............................*D. mucoroides*
- 8. Polar granules present, or PG (+)...............................*Cavenderia fasciculata*

### Diversity.

Eleven isolates of dictyostelids representing six species from three genera, three families, and two orders were cultured from eight of 254 soil samples collected in this study (Table 1; Table S4). However, these soil samples were not equally productive for dictyostelids. Soil sample (No. 5759) yielded the highest numbers of isolates (strain 5259-1-23, strain 5759-1-23, and strain 5759-2-23) and species (*Cavenderia fasciculata* and *Heterostelium pallidum*). Two isolates (strain 5729-huang-2021 and strain 5729-bai-2021) representing the same species (*Dictyostelium robusticaule*) were isolated from one soil sample (No. 5729). The other soil samples all yielded just one isolate and one species of dictyostelid.

Soil samples for isolation of dictyostelids in this study were collected from 11 study sites on Changbai Mountain in Jilin Province of China. However, soil samples from the different study sites yielded different numbers of isolates and species of dictyostelids, including the three study sites of Hanconggou, Changbai Mountain Nature Reserve, and the Xibao forestry farm. Dictyostelium discoideum (strain 5751-1-23 and strain 5752-3-17), *C. fasciculata* (strain 5754-2wang-23), and *H. recretum* (strain 5756-1-17) were isolated from soil samples collected in Hanconggou. *Dictyostelium mucoroides* (strain 5035-23 and strain 5735-3-23), *C. fasciculata* (strain 5259-1-23), and *H. pallidum* (strain 5759-1-23 and strain 5759-2-23) were isolated from soil samples collected in the Changbai Mountain Nature Reserve. *Dictyostelium robusticaule* (strain 5729-huang-2021 and strain 5729-bai-2021) was isolated from soil samples collected in the Xibao forestry farm.

*Dictyostelium mucoroides* (strain 5735-3-23), *D. robusticaule* (strain 5729-huang-2021 and strain 5729-bai-2021), *C. fasciculata* (strain 5754-2wang-23), and *H. recretum* (strain 5756-1-17) were isolated from mixed broadleaf-conifer forest soil, *C. fasciculata* (strain 5259-1-23) and *H. pallidum* (strain 5759-1-23 and strain 5759-2-23) from coniferous forest soil, D. discoideum (strain 5751-1-23 and strain 5752-3-17) from broadleaf deciduous forest soil, and *D. mucoroides* (strain 5035-23) from mountain tundra soil. Consequently, mixed broadleaf-conifer forests had the highest number of isolates, whereas the lowest numbers were recorded for tundra and broadleaf forests in this study.

Dictyostelium discoideum (strain 5751-1-23 and strain 5752-3-17), *D. robusticaule* (strain 5729-huang-2021 and strain 5729-bai-2021), *C. fasciculate* (strain 5754-2wang-23), and *H. recretum* (strain 5756-1-17) were isolated from soil samples collected at elevations below 1,000 m. *Dicytostelium mucoroides* (strain 5035-23 and strain 5735-3-23), *C. fasciculata* (strain 5259-1-23) and *H. pallidum* (strain 5759-1-23 and strain 5759-2-23) were isolated from soil samples collected at elevations from 1,000 to 2,100 m.

## DISCUSSION

### Molecular phylogeny.

*Dictyostelium robusticaule* appears to be closest to *D. mucoroides* and *D. multiforme* in *Dictyostelium* based on a rDNA SSU phylogeny (Fig. 3). These three species share some morphological characteristics, including solitary and erect sorocarps, but neither *D. mucoroides* nor *D. multiforme* have polar granules in their spores (13, 25). Moreover, *D. robusticaule* has the specific character of producing two types of sorocarps.

*Heterostelium recretum* has sorophores that regrow with whorled branches. The rDNA SSU phylogeny suggests that *H. recretum* is most closely related to *H. multibrachiatum* in *Heterostelium* (Fig. 4). These two species are described from two different localities in the world. *Heterostelium multibrachiatum* was described recently from a mixed forest in the Far East of Russia (43). There are also some morphological differences between the two species. Both have whorled branches on the sorophores. However, *H. recretum* has 2 to 8 nodes, sometimes up to 11, with 3 to 6 branches on each node, whereas *H. multibrachiatum* has 1 to 13 nodes, with 2 to 8 branches (or sometimes as many as 12) on the sorophores. *Heterostelium recretum* has branches that are 121.5 to 235.0 μm long, whereas *H. multibrachiatum* has branches 64.6 to 109.3 μm long. *Heterostelium recretum* has sorophores 2.6 to 12.2 mm long, and *H. multibrachiatum* is only 0.7 to 7.9 mm tall. In addition, *H. recretum* can regrow from terminal collapsed sori, but there are no records of this occurring in *H. multibrachiatum*.

In this paper, we also obtained *atp1* sequences from dictyostelids to assess their use for species identification (Fig. 3–5). The mitochondrial *atp1* gene phylogeny supports the results of the SSU rDNA phylogeny. In the mitochondrial *atp1* gene phylogeny tree, Dictyostelium discoideum, *D. robusticaule*, *Heterostelium pallidum*, and *Cavenderia fasciculata* formed a tight cluster. All *atp1* sequences we obtained in the present study clustered near other sequences of same genus. On the other hand, the *atp1* gene of dictyostelids has about 570 bp, less than the SSU rDNA sequence with about 1,800 bp, could help to reduce the costs of DNA sequencing. Consequently, the *atp1* gene could be helpful to identify dictyostelids with a complement of SSU rDNA sequence.

### Potential diversity and its environmental effects.

Only three other species of dictyostelids (*Dictyostelium firmibasis*, *Heterostelium candidum*, and *Polysphondylium violaceum*) have been previously recorded from Changbai Mountain (21–23). The present study increased the number to nine, including the two new species (*D. robusticaule* and *H. recretum*). The other species already known from Changbai Mountain have been recorded fewer than three times in China except for *H. pallidum* (21–23, 25, 31, 45–68), and three truly cosmopolitan species (*Cavenderia aureostipes*, *D. brefeldianum*, and *D. sphaerocephalum*) are not yet known from this location.

### Effects of latitude on the diversity of dictyostelids.

**FIG 12:**
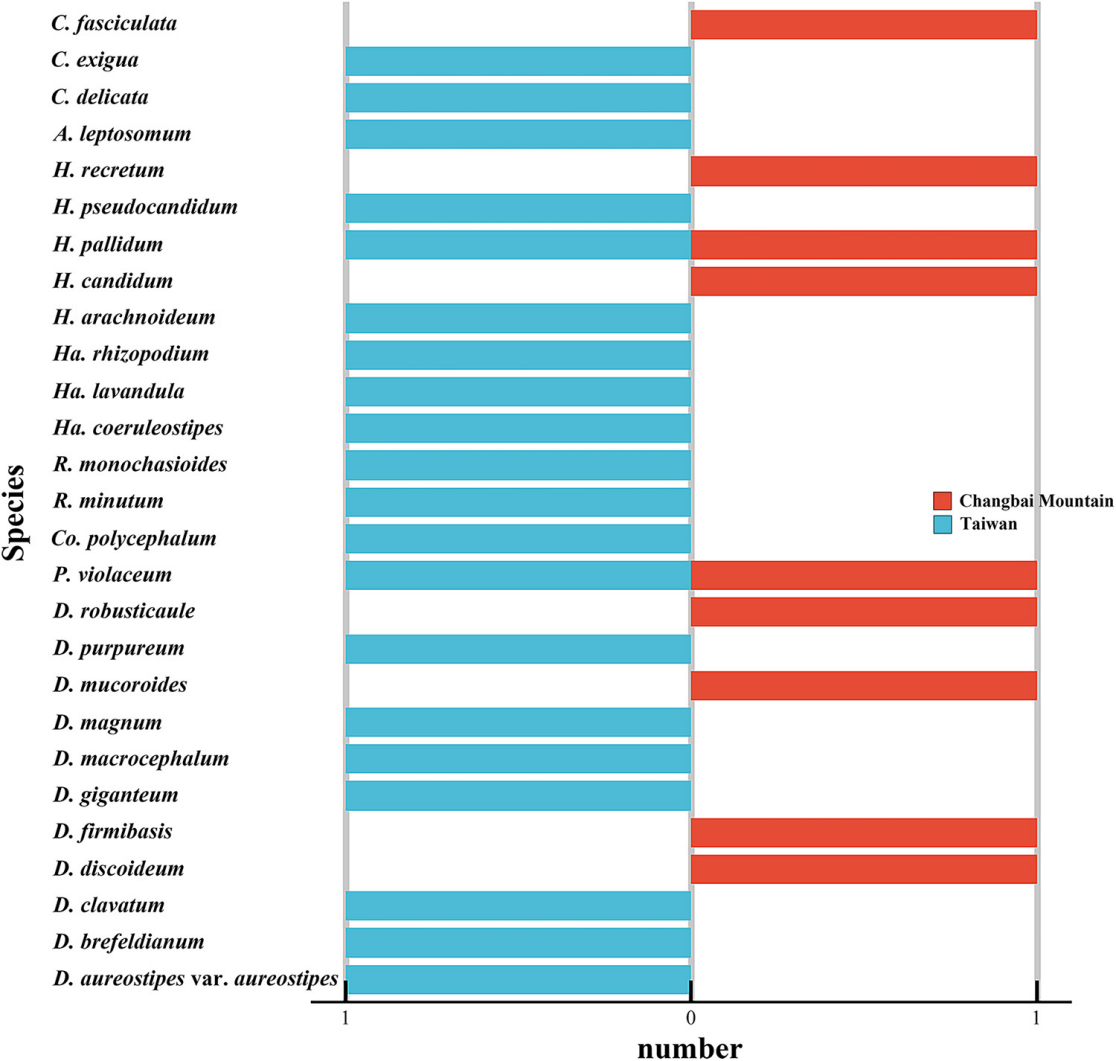
Species that have been recorded in Taiwan and on Changbai Mountain. *A*, *Acytostelium*; *C*, *Cavenderia*; *Co*, *Coremiostelium*; *D*, *Dictyostelium*; *H*, *Heterostelium*; *Ha*, *Hagiwaraea*; *P*, *Polysphondylium*; *R*, *Raperostelium*.

Changbai Mountain (127°40' to 128°16' E, 41°35' to 42°25' N) is located at a similar longitude but at a very different latitude from the island of Taiwan (120°08' to 122°01' E, 21°53' to 25°18' N), yet studies have revealed an appreciable difference in the diversity of dictyostelids for the two localities. Twenty species of dictyostelids have been recorded from Taiwan, compared with just nine species recorded from Changbai Mountain (Table S4; Fig. 12) (21–23, 45–48, 51, 53, 63–65). The higher latitude of Changbai Mountain may account, at least in part for this difference in diversity, based on data reported by Perrigo et al. (69) in Sweden.

**FIG 13:**
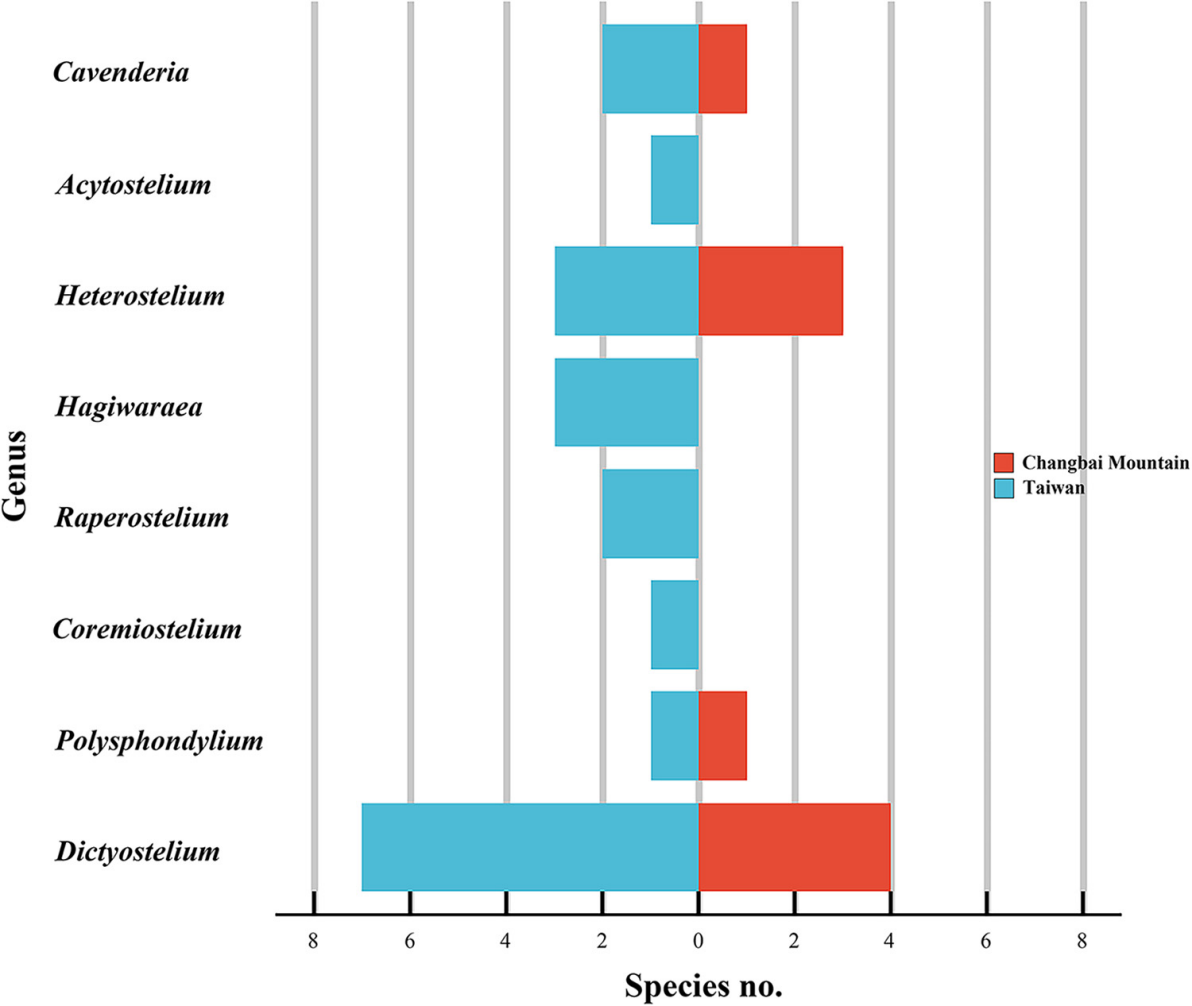
Numbers of species of dictyostelids in the different genera that have been recorded in Taiwan and on Changbai Mountain.

**FIG 14:**
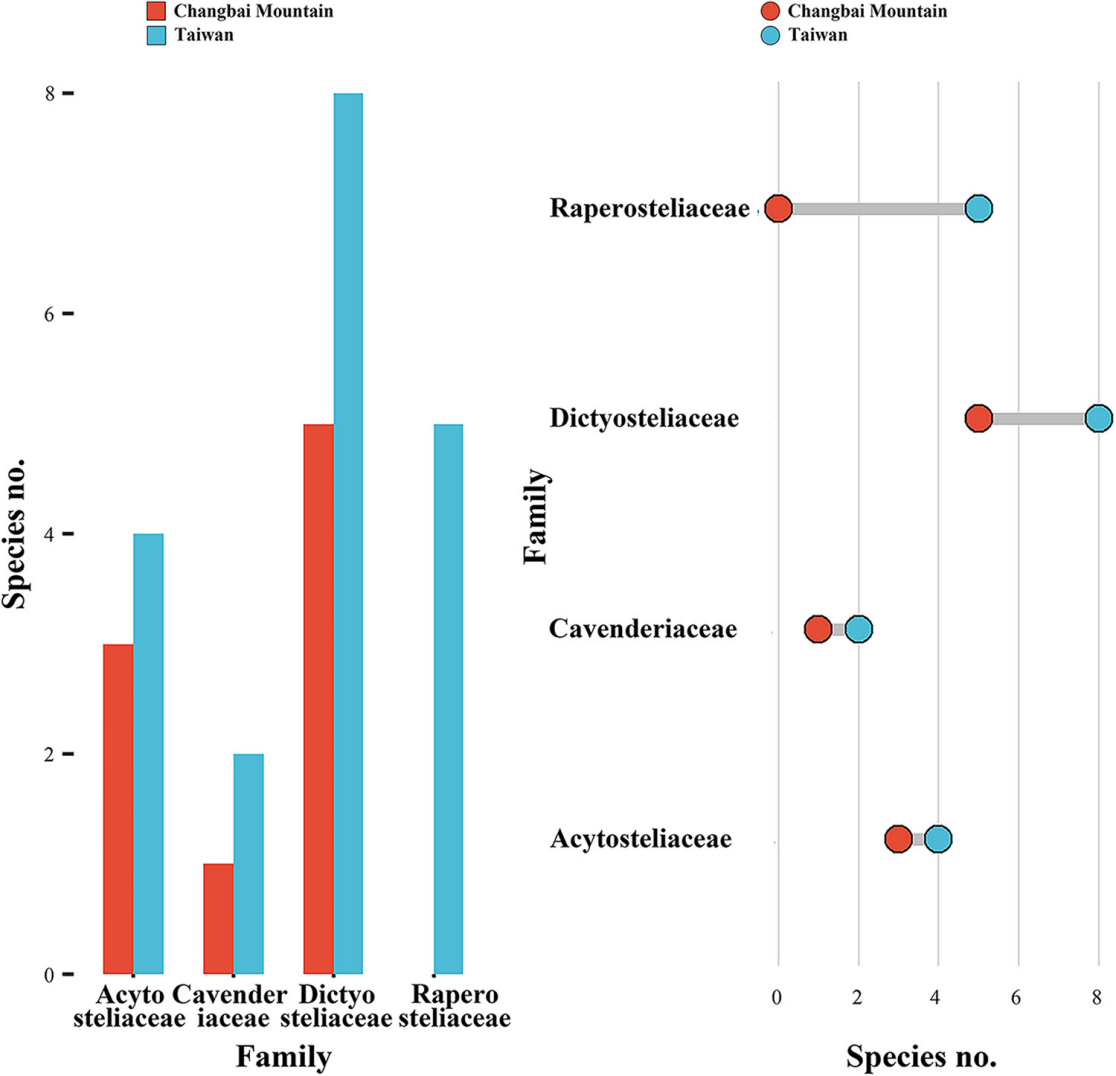
Numbers of species of dictyostelids in the different families that have been recorded in Taiwan and on Changbai Mountain.

Eight genera have been recorded from Taiwan, and half of these appeared in samples collected on Changbai Mountain (Fig. 13). In previous research, these four genera shared in common have encompassed more than 85% species the species recorded from China, and this proportion has increased as a result of the data reported herein (70). The absence of *Raperostelium* and *Hagiwarea* on Changbai Mountain is interesting, because members of these two represent a quarter of all dictyostelids known in Taiwan and 11% of those known in all of China. One hypothesis to account for the absence of *Raperostelium* or *Hagiwarea* is that they do not tolerate the environmental conditions associated with high latitude habitats. On the other hand, *Raperostelium* or *Hagiwarea* are difficult to find in traditional cultures (71). Numbers of species in the four families represented in the isolates from Changbai Mountain were all less than the corresponding numbers for Taiwan (Fig. 14), which suggests generally lower numbers of species at high latitudes. These data display a pattern of diversity at the family level that might be related to differences in latitude.

### Effects of vegetation type on the diversity of dictyostelids.

**FIG 15:**
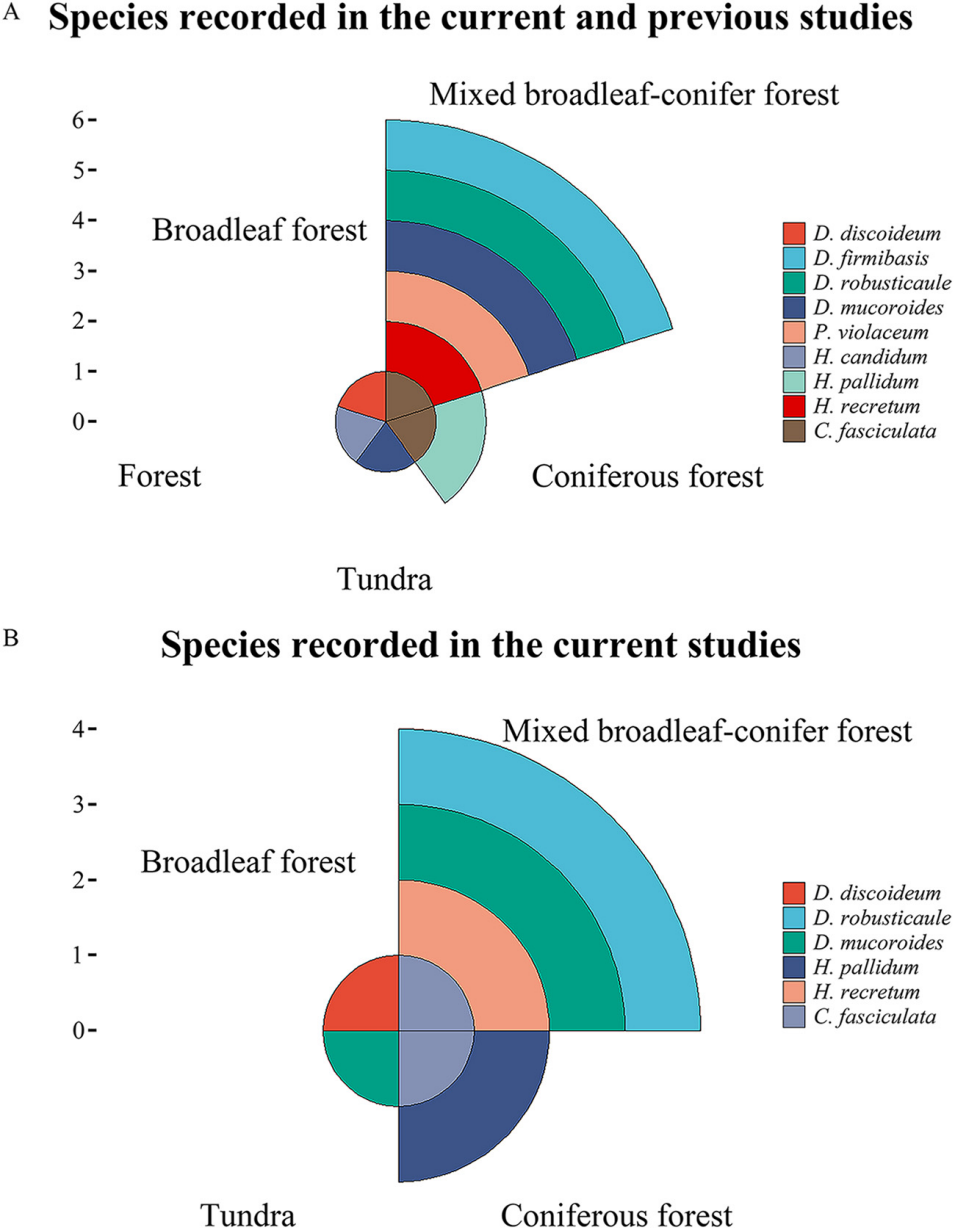
Species recorded on Changbai Mountain. (A) Species recorded in the current and previous studies on Changbai Mountain. (B) Species recorded in the current studies on Changbai Mountain. *C*, *Cavenderia*; *D*, *Dictyostelium*; *H*, *Heterostelium*; *P*, *Polysphondylium*.

Liu et al. (70) reported that broadleaf forests are characterized by the highest number of species of dictyostelids known to occur in China, but the results of this study and a previous study (21–23) indicated that the maximum number of species of dictyostelids on Changbai Mountain occurs in mixed broadleaf-conifer forests (Table S5; Fig. 15). The difference is possibly due to two reasons. First, the more expansive areas of mixed broadleaf-conifer forest on Changbai Mountain could account for higher species richness. Second, the relatively higher latitude may affect patterns of diversity.

In the present study, four species were isolated from mixed broadleaf-conifer forest soil, two species from coniferous forest soil, one species from broadleaf deciduous forest soil, and one species from mountain tundra soil (Fig. 15). As such, mixed broadleaf-conifer forest soil on Changbai Mountain had the highest species richness, which differs from the data obtained by Liu et al. (70). However, Liu et al. (70) discussed dictyostelid diversity for all of China, whereas the present study investigated only Changbai Mountain, which occurs at a higher latitude than most other areas of China. Liu et al. (25) and Romeralo and Lado (72) discussed some species of dictyostelids that are associated with different types of forests. Romeralo et al. (73) showed that a combination of climatic conditions (temperature and water availability), physical factors (pH), and vegetation (species richness) factors favor dictyostelid species richness. This warrants further study.

**FIG 16:**
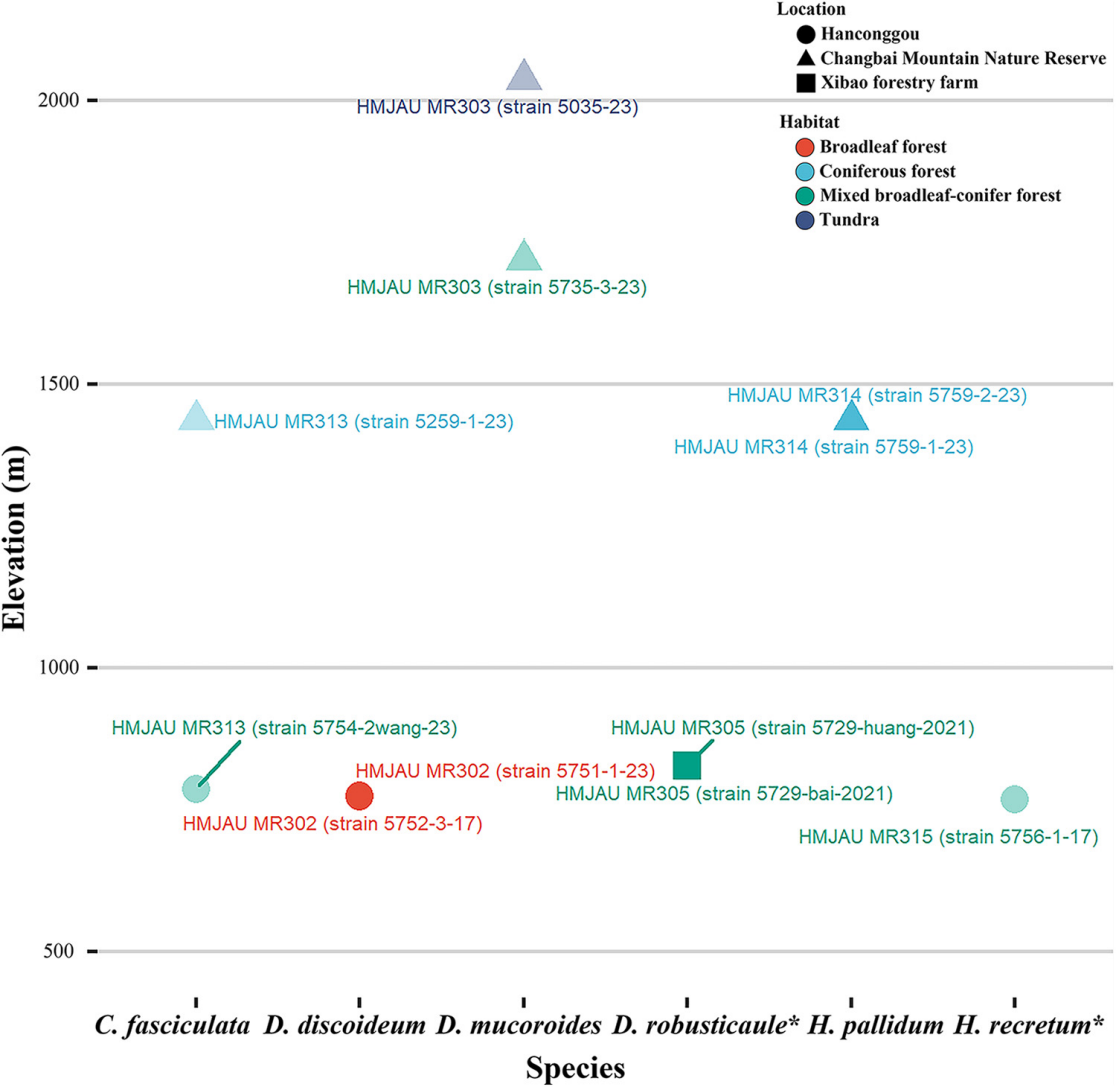
Relationships of elevation, species, isolates, habitat, and location in the present study. Habitats are shown in different colors. Localities are indicated by different shapes. Isolates are on display with names near the points. Species with one isolate are semihyaline, species with two isolates are nontransparent. New species are indicated with asterisks. *C*, *Cavenderia*; *D*, *Dictyostelium*; *H*, *Heterostelium*.

At same elevations (500 to 1,000 m elevation) on Changbai Mountain, mixed broadleaf-conifer forests yielded more isolates (four compared with two) and species (three compared with one) than broadleaf forests (Fig. 16). Mixed broadleaf-conifer forests were also found to have the highest species richness of dictyostelids. Consequently, at the same latitude, vegetation type might possess more impact than elevation on dictyostelid diversity.

### Effects of elevation on the diversity of dictyostelids.

In the present study, four species of dictyostelids were isolated from soil samples collected at elevations below 1,000 m. Three species were isolated from soil samples collected at elevations from 1,000 to 2,100 m (Table 1; Fig. 16). These data, albeit limited, suggest a pattern of slightly decreasing diversity with increasing elevation. This pattern fits the results reported by Landolt at al. (74), but not with what was reported by Paillet and Satre (75). Liu et al. (25) investigated dictyostelid biodiversity in mountains at elevations >2,000 m and indicated that dictyostelids are probably common at higher elevations. But the relationship of elevation and biodiversity needs to be investigated further.

*Dictyostelium mucoroides* was the only species recorded at an elevation above 1,500 m (Fig. 16). This species was found in both tundra and mixed broadleaf-conifer forests above 1,500 m, which suggest that *D*. *mucoroides* is well adapted for the environmental conditions associated with high elevations.

As a general pattern, study sites at lower elevations produced higher numbers of species as well as isolates. Four of six species and six of 11 isolates were recorded at elevations below 1,000 m (Fig. 16). In mixed broadleaf-conifer forests below 1,000 m elevation, there were three species and four isolates, but only one species and isolate above 1,000 m. Two species and three isolates were recorded below 1,500 m elevation, and one species with two isolates above 1,500 m. This suggests that environmental conditions at lower elevations appear to be more suitable for dictyostelids.

### Limitations of the present study.

A few limitations should be considered with respect to the present study. First, we investigated species richness of dictyostelids only on a spatial scale, not considering either the temporal scale or the phylogenetic scale. Estes et al. (76) suggested that it is necessary to observe the behavior of an organism across multiple spatial and temporal scales to understand ecological phenomena, and Ladau and Eloe-Fadrosh (77) indicated that the spatial scale, the temporal scale, and the phylogenetic scale must be studied specifically for microbial ecology. Therefore, future research could focus on the temporal scale and phylogenetic scale. Second, the present study investigated dictyostelids with the traditional morphology-based isolation and culture technique. However, McCaig et al. (78) used molecular method to show that only about 1% of the total bacteria population could be cultured under a restricted range of media and cultivation conditions. Furthermore, Mora et al. (79) showed there are numerous protists yet to be discovered. Consequently, there are likely numerous species of dictyostelids in our samples that could not be cultured with the techniques we used. High-throughput sequencing techniques could be used in a more systematic and exhaustive ecological study as the next step. Recently, new species and one new variant have been reported from other studies carried out in China (25, 54, 59, 68). The relatively small number of scientists studying dictyostelids limits the amount of data available for many regions of the world; therefore, there is the potential for many new species that have yet to be discovered.

## MATERIALS AND METHODS

### Isolation, cultivation, and morphological assessment.

A total of 254 soil samples for isolation of dictyostelids were collected from 11 study sites on Changbai Mountain in Jilin Province of China in 2016 and 2017 (Fig. 1; Table S1). These samples were collected in broadleaf deciduous forests (elevation 700 m to 800 m), mixed broadleaf-conifer forests (elevations <1,000 m), coniferous forests (elevation 1,000 to 1,800 m), alpine birch forests (elevations between 1,800 to 2,000 m), and areas of mountain tundra (elevations >2,000 m) (Fig. 1). Each sample (approximately 50 g) was labeled with the date, vegetation type, elevation, and latitude/longitude. All samples were preserved at 4°C as soon as possible after being collected.

The isolation and culturing methods used followed those described by Cavender and Raper (80) with a few minor modifications. A dilute organic medium, which has been found to stimulate growth and development of dictyostelids but which is too dilute to allow appreciable growth of bacteria or fungi was made from leached, dried hay (largely *Setaria* spp.) weathered for several weeks and collected from fields or roadsides. An infusion was made from 10 g of hay per L of distilled water by autoclaving at 120°C for 20 min, filtering the infusion through cheesecloth and cotton, and then bringing it up to volume with distilled water. The infusion was buffered with 1.5 g KH_2_PO_4_ and 0.62 g Na_2_HPO_4_·7H_2_O per L to yield pH 6.0 ±. Fifteen grams of agar were added per L.

Ten grams of soil were measured into flasks (500 mL) containing 90 mL of sterile distilled water, giving an initial dilution of 1:10. Flasks were then placed on a rotary shaker at 280 rpm for 2 min to break up soil particles and to distribute spores and myxamoebae. A dilution of 1:25 was made by adding 5 mL of the above suspension to 7.5 mL sterile water, and 0.5 mL of this dilution was added to each culture plate, giving a final dilution of 1/50 g soil/plate. At the same time, ca 0.4 mL of a heavy suspension of the pregrown bacterium E. coli was added to provide nutrition for the dictyostelids, which provided sufficient nutrition for preparing six replicates per soil sample. E. coli was grown on a LB broth medium (Bopebio, China) in conical flasks for a period of 200 r/min, 12 to 24 h at 30°C. The bacterial and soil suspensions were mixed together and distributed over the surface of a plate by tilting it back and forth. Plates were poured several days in advance in order for free water to evaporate, making the sufficiently agar dry enough to absorb the excess liquid. Plates were then incubated at 23°C and 17°C with a 12 h light and dark cycle, respectively. Each plate was examined at least once a day for 2 weeks after the appearance of initial aggregations. Each isolate was subcultured for taxonomic studies and preserved on nonnutrient water agar plates with E. coli pregrown for 12 to 24 h.

Dictyostelid isolates were initially identified with the use of the descriptions provided by Raper (13) and Hagiwara (36), whose nomenclature was also followed except for those species recently assigned to new genera in the system of classification proposed by Sheikh et al. (9). The characteristic stages in the life cycle, including cell aggregation, pseudoplasmodia, and sorocarps, were observed under a Zeiss dissecting microscope (Axio Zoom V16, Carl Zeiss Microscopy GmbH, Göttingen, Germany) with a 1.5× objective and 10× ocular. Slides with sorocarps were prepared with water as the mounting medium. Features of spores, sorophores, and sorocarps were observed and measured on the slides by using a Zeiss light microscope (Axio Imager A2, Carl Zeiss Microscopy GmbH, Göttingen, Germany), with 10× ocular and 40× objectives. Photographs were taken with Zeiss Axiocam 506 color microscope camera (Carl Zeiss Microscopy GmbH, Göttingen, Germany). All isolates were deposited in HL-5 media (81), which was frozen at −80°C and deposited in the herbarium of the Mycological Institute of Jilin Agricultural University, Changchun, China (HMJAU).

### Phylogenetic analyses.

DNA was extracted from resulting isolates following the methods described by Liu et al. (5). Sorocarps were collected with a sterile tip and mixed with the lysis buffer of the MiniBEST Universal Genomic DNA Extraction Kit Ver.5.0 (TaKaRa Bio Inc., Kusatsu, Japan) according to the manufacturer’s protocol.

The ribosomal SSU and *atp1* were amplified by using the PCR, following the protocol described in Perrigo (82). The rDNA SSU sequence amplification used the primers 18S-FA (5′→ 3′: AACCTGGTTGATCCTGCCAG) and 18S-RB (5′→ 3′: TGATCCTTCTGCAGGTTCAC) (83), along with D542F (5′→ 3′: ACAATTGGAGGGCAAGTCTG) and D134R (5′→ 3′: TCGAGGTCTCGTCCGTTATC) (32). The *atp1* sequence amplification was carried out using the primers ATP_352F (5′→ 3′: TGTTAGGAMGAGTAGTWGATGTATTAGG) and ATP_897R (5′→ 3′: TCTCCTGGRTAYGCTTCTCKTCCTGG) (82). PCR products were sent to Genewiz (Tianjin, China) for sequencing.

The sequences obtained were deposited in the GenBank database. The sequence data for all closely related species were downloaded from GenBank for phylogenetic analysis (Tables S2 and S3). The *atp1* and SSU sequences were aligned and compared separately by using the program ClustalW Multiple alignment version 2.1 (84), then manually adjusted in BioEdit version 7.0.9.0 (85). Maximum likelihood analyses (ML analyses) were performed using IQTREE v.1.6.12 (86), with 10,000 replicates of ultrafast–likelihood bootstrapping to obtain node support values by “-bb 10,000” option, and further optimized using a hill-climbing nearest-neighbor interchange (NNI) by the “-bnni” option (87, 88). We also used the SH-aLRT test to obtain the confidence limit of the topology by the “-alrt 1,000” option (89). The “-nt AUTO” option was used to automatically determine the best number of cores given the current data. We used ModelFinder as implemented within IQ-TREE to determine the best substitution model based on Bayesian information criteria (BIC) (90). In the ML analyses of *Cavenderia* SSU and *atp1* sequences, the model of “TIM2+F+I+G4” was selected, and using a myxomycete sequence (Physarum polycephalum, Accession no. X13160.1/NC_002508.1) as the outgroup with the “-o X13160.1”/“-o NC_002508.1” option. In the ML analyses of *Dictyostelium* SSU and *atp1* sequence, the model of “TIM2+F+G4” was selected, and using a myxomycete sequence (Physarum polycephalum, Accession no. X13160.1/NC_002508.1) as the outgroup with the “-o X13160.1”/“-o NC_002508.1” option. In the ML analyses of *Heterostelium* SSU and *atp1* sequence, the model of “TPM2u+F+G4” was selected, and using a myxomycete sequence (Physarum polycephalum, Accession no. X13160.1/NC_002508.1) as the outgroup with the “-o X13160.1”/“-o NC_002508.1” option.

### Data availability.

The isolates and the NCBI GenBank accession numbers of SSU and *atp1* DNA sequences considered in the present study are listed in Table S2 and S3. Sequence data are available in GenBank (Accession Numbers MT125951, MT125952, MW660746, MW660747, MW660748, MW660749, MW660750, MW660751, MW857293, MW931856, MW931857, MZ318386, MZ318387, MZ318388, MZ318389, MZ318390, MZ318391, MZ318392, MZ318393, MZ318394, and MZ318395). The nomenclature of the new species in the present study is available in MycoBank (MB830534 and MB830541).
