# Supplementary material for: Diversity of Dictyostelid Cellular Slime Molds, Including Two Species New to Science, in Forest Soils of Changbai Mountain, China
Source: Microbiol Spectr. 2022 Oct 3;10(5):e02402-22. doi: 10.1128/spectrum.02402-22 (PMC9620775; doi:10.1128/spectrum.02402-22)
Supplement: Supplemental Material 1 — Tables S1 to S5 and Texts S1 to S4. Download spectrum.02402-22-s0001.pdf, PDF file, 0.6 MB [file spectrum.02402-22-s0001.pdf]

Supplementary Files

Table S1. The 254 soil samples for isolation of dictyostelids collected from eleven study sites on Changbai Mountain in the Jilin Province of China in 2016 and 2017.

Number	Data	Location	Habitat	Collection people	Latitude (north)	Longitude (east)	Altitude (m)
4905	2016.8.28	Changbai Mountain Nature Reserve	Mixed broadleaf-conifer forest	Y Zou, JG Hou	42° 10' 47" 52.8	128° 10' 02" 49.2	1155
4906	2016.8.28	Changbai Mountain Nature Reserve	Mixed broadleaf-conifer forest	Y Zou, JG Hou	42° 10' 47" 52.8	128° 10' 02" 49.2	1155
4907	2016.8.28	Changbai Mountain Nature Reserve	Mixed broadleaf-conifer forest	Y Zou, JG Hou	42° 10' 47" 52.8	128° 10' 02" 49.2	1155
4908	2016.8.28	Changbai Mountain Nature Reserve	Mixed broadleaf-conifer forest	Y Zou, JG Hou	42° 10' 47" 52.8	128° 10' 02" 49.2	1155
4909	2016.8.28	Changbai Mountain Nature Reserve	Mixed broadleaf-conifer forest	Y Zou, JG Hou	42° 10' 47" 52.8	128° 10' 02" 49.2	1155
4910	2016.8.28	Changbai Mountain Nature Reserve	Mixed broadleaf-conifer forest	Y Zou, JG Hou	42° 10' 46" 48	128° 09' 53" 27.6	1133
4911	2016.8.28	Changbai Mountain Nature Reserve	Mixed broadleaf-conifer forest	Y Zou, JG Hou	42° 10' 46" 48	128° 09' 53" 27.6	1133
4912	2016.8.28	Changbai Mountain Nature Reserve	Mixed broadleaf-conifer forest	Y Zou, JG Hou	42° 10' 46" 48	128° 09' 53" 27.6	1133
4913	2016.8.28	Changbai Mountain Nature Reserve	Mixed broadleaf-conifer forest	Y Zou, JG Hou	42° 10' 46" 48	128° 09' 53" 27.6	1133
4914	2016.8.28	Changbai Mountain Nature Reserve	Mixed broadleaf-conifer forest	Y Zou, JG Hou	42° 10' 46" 48	128° 09' 53" 27.6	1133
4915	2016.8.28	Changbai Mountain Nature Reserve	Mixed broadleaf-conifer forest	Y Zou, JG Hou	42° 10' 56" 38.4	128° 10' 00" 54	1133
4916	2016.8.28	Changbai Mountain Nature Reserve	Mixed broadleaf-conifer forest	Y Zou, JG Hou	42° 10' 56" 38.4	128° 10' 00" 54	1133
4917	2016.8.28	Changbai Mountain Nature Reserve	Mixed broadleaf-conifer forest	Y Zou, JG Hou	42° 10' 56" 38.4	128° 10' 00" 54	1133

4918	2016.8.28	Changbai Mountain Nature Reserve	Mixed broadleaf-conifer forest	Y Zou, JG Hou	42° 10' 56" 38.4	128° 10' 00" 54	1133
4919	2016.8.28	Changbai Mountain Nature Reserve	Mixed broadleaf-conifer forest	Y Zou, JG Hou	42° 10' 56" 38.4	128° 10' 00" 54	1133
4920	2016.8.28	Changbai Mountain Nature Reserve	Mixed broadleaf-conifer forest	Y Zou, JG Hou	42° 11' 04" 19.2	128° 10' 04" 26.4	1200
4921	2016.8.28	Changbai Mountain Nature Reserve	Mixed broadleaf-conifer forest	Y Zou, JG Hou	42° 11' 04" 19.2	128° 10' 04" 26.4	1200
4922	2016.8.28	Changbai Mountain Nature Reserve	Mixed broadleaf-conifer forest	Y Zou, JG Hou	42° 11' 04" 19.2	128° 10' 04" 26.4	1200
4923	2016.8.28	Changbai Mountain Nature Reserve	Mixed broadleaf-conifer forest	Y Zou, JG Hou	42° 11' 04" 19.2	128° 10' 04" 26.4	1200
4924	2016.8.28	Changbai Mountain Nature Reserve	Mixed broadleaf-conifer forest	Y Zou, JG Hou	42° 11' 04" 19.2	128° 10' 04" 26.4	1200
4925	2016.8.28	Changbai Mountain Nature Reserve	Mixed broadleaf-conifer forest	Y Zou, JG Hou	42° 11' 04" 26.4	128° 10' 12" 25.2	1134
4926	2016.8.28	Changbai Mountain Nature Reserve	Mixed broadleaf-conifer forest	Y Zou, JG Hou	42° 11' 04" 26.4	128° 10' 12" 25.2	1134
4927	2016.8.28	Changbai Mountain Nature Reserve	Mixed broadleaf-conifer forest	Y Zou, JG Hou	42° 11' 04" 26.4	128° 10' 12" 25.2	1134
4928	2016.8.28	Changbai Mountain Nature Reserve	Mixed broadleaf-conifer forest	Y Zou, JG Hou	42° 11' 04" 26.4	128° 10' 12" 25.2	1134
4929	2016.8.28	Changbai Mountain Nature Reserve	Mixed broadleaf-conifer forest	Y Zou, JG Hou	42° 11' 04" 26.4	128° 10' 12" 25.2	1134
4930	2016.8.28	Changbai Mountain Nature Reserve	Mixed broadleaf-conifer forest	Y Zou, JG Hou	42° 10' 45" 14.4	128° 10' 06" 21.6	1151
4931	2016.8.28	Changbai Mountain Nature Reserve	Mixed broadleaf-conifer forest	Y Zou, JG Hou	42° 10' 45" 14.4	128° 10' 06" 21.6	1151
4932	2016.8.28	Changbai Mountain Nature Reserve	Mixed broadleaf-conifer forest	Y Zou, JG Hou	42° 10' 45" 14.4	128° 10' 06" 21.6	1151
4933	2016.8.28	Changbai Mountain Nature Reserve	Mixed broadleaf-conifer forest	Y Zou, JG Hou	42° 10' 45" 14.4	128° 10' 06" 21.6	1151

4934	2016.8.28	Changbai Mountain Nature Reserve	Mixed broadleaf-conifer forest	Y Zou, JG Hou	42° 10' 45" 14.4	128° 10' 06" 21.6	1151
4935	2016.8.28	Changbai Mountain Nature Reserve	Mixed broadleaf-conifer forest	Y Zou, JG Hou	42° 10' 40" 44.4	128° 10' 08" 13.2	1101
4936	2016.8.28	Changbai Mountain Nature Reserve	Mixed broadleaf-conifer forest	Y Zou, JG Hou	42° 10' 40" 44.4	128° 10' 08" 13.2	1101
4937	2016.8.28	Changbai Mountain Nature Reserve	Mixed broadleaf-conifer forest	Y Zou, JG Hou	42° 10' 40" 44.4	128° 10' 08" 13.2	1101
4938	2016.8.28	Changbai Mountain Nature Reserve	Mixed broadleaf-conifer forest	Y Zou, JG Hou	42° 10' 40" 44.4	128° 10' 08" 13.2	1101
4939	2016.8.28	Changbai Mountain Nature Reserve	Mixed broadleaf-conifer forest	Y Zou, JG Hou	42° 10' 41" 06	128° 10' 11" 02.4	1161
4940	2016.8.28	Changbai Mountain Nature Reserve	Mixed broadleaf-conifer forest	Y Zou, JG Hou	42° 10' 41" 06	128° 10' 11" 02.4	1161
4941	2016.8.28	Changbai Mountain Nature Reserve	Mixed broadleaf-conifer forest	Y Zou, JG Hou	42° 10' 41" 06	128° 10' 11" 02.4	1161
4942	2016.8.28	Changbai Mountain Nature Reserve	Mixed broadleaf-conifer forest	Y Zou, JG Hou	42° 10' 41" 06	128° 10' 11" 02.4	1161
4943	2016.8.28	Changbai Mountain Nature Reserve	Mixed broadleaf-conifer forest	Y Zou, JG Hou	42° 10' 41" 06	128° 10' 11" 02.4	1161
4944	2016.8.28	Changbai Mountain Nature Reserve	Mixed broadleaf-conifer forest	Y Zou, JG Hou	42° 10' 36" 50.4	128° 10' 22" 12	1160
4945	2016.8.28	Changbai Mountain Nature Reserve	Mixed broadleaf-conifer forest	Y Zou, JG Hou	42° 10' 36" 50.4	128° 10' 22" 12	1160
4946	2016.8.28	Changbai Mountain Nature Reserve	Mixed broadleaf-conifer forest	Y Zou, JG Hou	42° 10' 36" 50.4	128° 10' 22" 12	1160
4947	2016.8.28	Changbai Mountain Nature Reserve	Mixed broadleaf-conifer forest	Y Zou, JG Hou	42° 10' 36" 50.4	128° 10' 22" 12	1160
4948	2016.8.28	Changbai Mountain Nature Reserve	Mixed broadleaf-conifer forest	Y Zou, JG Hou	42° 10' 36" 50.4	128° 10' 22" 12	1160
4949	2016.8.29	Changbai Mountain Nature Reserve	Mixed broadleaf-conifer forest	Y Zou, JG Hou	42° 04' 13" 44.4	128° 03' 49" 55.2	1670

4950	2016.8.29	Changbai Mountain Nature Reserve	Mixed broadleaf-conifer forest	Y Zou, JG Hou	42° 04' 13" 44.4	128° 03' 49" 55.2	1670
4951	2016.8.29	Changbai Mountain Nature Reserve	Mixed broadleaf-conifer forest	Y Zou, JG Hou	42° 04' 13" 44.4	128° 03' 49" 55.2	1670
4952	2016.8.29	Changbai Mountain Nature Reserve	Mixed broadleaf-conifer forest	Y Zou, JG Hou	42° 04' 13" 44.4	128° 03' 49" 55.2	1670
4953	2016.8.29	Changbai Mountain Nature Reserve	Mixed broadleaf-conifer forest	Y Zou, JG Hou	42° 04' 13" 44.4	128° 03' 49" 55.2	1670
4954	2016.8.29	Changbai Mountain Nature Reserve	Mixed broadleaf-conifer forest	Y Zou, JG Hou	42° 04' 10" 44.4	128° 03' 55" 30	1724
4955	2016.8.29	Changbai Mountain Nature Reserve	Mixed broadleaf-conifer forest	Y Zou, JG Hou	42° 04' 10" 44.4	128° 03' 55" 30	1724
4956	2016.8.29	Changbai Mountain Nature Reserve	Mixed broadleaf-conifer forest	Y Zou, JG Hou	42° 04' 10" 44.4	128° 03' 55" 30	1724
4957	2016.8.29	Changbai Mountain Nature Reserve	Mixed broadleaf-conifer forest	Y Zou, JG Hou	42° 04' 10" 44.4	128° 03' 55" 30	1724
4958	2016.8.29	Changbai Mountain Nature Reserve	Mixed broadleaf-conifer forest	Y Zou, JG Hou	42° 04' 10" 44.4	128° 03' 55" 30	1724
4959	2016.8.29	Changbai Mountain Nature Reserve	Mixed broadleaf-conifer forest	Y Zou, JG Hou	42° 04' 10" 44.4	128° 03' 55" 30	1724
4960	2016.8.29	Changbai Mountain Nature Reserve	Mixed broadleaf-conifer forest	Y Zou, JG Hou	42° 04' 10" 44.4	128° 03' 55" 30	1724
4961	2016.8.29	Changbai Mountain Nature Reserve	Mixed broadleaf-conifer forest	Y Zou, JG Hou	42° 04' 10" 44.4	128° 03' 55" 30	1724
4962	2016.8.29	Changbai Mountain Nature Reserve	Coniferous forest	Y Zou, JG Hou	42° 04' 06" 43.2	128° 03' 56" 45.6	1745
4963	2016.8.29	Changbai Mountain Nature Reserve	Coniferous forest	Y Zou, JG Hou	42° 04' 06" 43.2	128° 03' 56" 45.6	1745
4964	2016.8.29	Changbai Mountain Nature Reserve	Coniferous forest	Y Zou, JG Hou	42° 04' 06" 43.2	128° 03' 56" 45.6	1745
4965	2016.8.29	Changbai Mountain Nature Reserve	Coniferous forest	Y Zou, JG Hou	42° 04' 06" 43.2	128° 03' 56" 45.6	1745

4966	2016.8.29	Changbai Mountain Nature Reserve	Coniferous forest	Y Zou, JG Hou	42° 04' 06" 43.2	128° 03' 56" 45.6	1745
4967	2016.8.29	Changbai Mountain Nature Reserve	Coniferous forest	Y Zou, JG Hou	42° 04' 06" 43.2	128° 03' 56" 45.6	1745
4968	2016.8.29	Changbai Mountain Nature Reserve	Coniferous forest	Y Zou, JG Hou	42° 04' 06" 43.2	128° 03' 56" 45.6	1745
4969	2016.8.29	Changbai Mountain Nature Reserve	Coniferous forest	Y Zou, JG Hou	42° 04' 06" 43.2	128° 03' 56" 45.6	1745
4970	2016.8.29	Changbai Mountain Nature Reserve	Coniferous forest	Y Zou, JG Hou	42° 04' 04" 26.4	128° 03' 59" 34.8	1780
4971	2016.8.29	Changbai Mountain Nature Reserve	Coniferous forest	Y Zou, JG Hou	42° 04' 04" 26.4	128° 03' 59" 34.8	1780
4972	2016.8.29	Changbai Mountain Nature Reserve	Coniferous forest	Y Zou, JG Hou	42° 04' 04" 26.4	128° 03' 59" 34.8	1780
4973	2016.8.29	Changbai Mountain Nature Reserve	Coniferous forest	Y Zou, JG Hou	42° 04' 04" 26.4	128° 03' 59" 34.8	1780
4974	2016.8.29	Changbai Mountain Nature Reserve	Coniferous forest	Y Zou, JG Hou	42° 04' 04" 26.4	128° 03' 59" 34.8	1780
4975	2016.8.29	Changbai Mountain Nature Reserve	Coniferous forest	Y Zou, JG Hou	42° 04' 04" 26.4	128° 03' 59" 34.8	1780
4976	2016.8.29	Changbai Mountain Nature Reserve	Coniferous forest	Y Zou, JG Hou	42° 04' 04" 26.4	128° 03' 59" 34.8	1780
4977	2016.8.29	Changbai Mountain Nature Reserve	Coniferous forest	Y Zou, JG Hou	42° 04' 04" 26.4	128° 03' 59" 34.8	1780
4978	2016.8.29	Changbai Mountain Nature Reserve	Mix birch-conifer forest	Y Zou, JG Hou	42° 04' 03" 50.4	128° 03' 59" 52.8	1807
4979	2016.8.29	Changbai Mountain Nature Reserve	Mix birch-conifer forest	Y Zou, JG Hou	42° 04' 03" 50.4	128° 03' 59" 52.8	1807
4980	2016.8.29	Changbai Mountain Nature Reserve	Mix birch-conifer forest	Y Zou, JG Hou	42° 04' 03" 50.4	128° 03' 59" 52.8	1807
4981	2016.8.29	Changbai Mountain Nature Reserve	Mix birch-conifer forest	Y Zou, JG Hou	42° 04' 03" 50.4	128° 03' 59" 52.8	1807

4982	2016.8.29	Changbai Mountain Nature Reserve	Mix birch-conifer forest	Y Zou, JG Hou	42° 04' 03" 50.4	128° 03' 59" 52.8	1807
4983	2016.8.29	Changbai Mountain Nature Reserve	Mix birch-conifer forest	Y Zou, JG Hou	42° 04' 03" 50.4	128° 03' 59" 52.8	1807
4984	2016.8.29	Changbai Mountain Nature Reserve	Mix birch-conifer forest	Y Zou, JG Hou	42° 04' 03" 50.4	128° 03' 59" 52.8	1807
4985	2016.8.29	Changbai Mountain Nature Reserve	Mix birch-conifer forest	Y Zou, JG Hou	42° 04' 03" 50.4	128° 03' 59" 52.8	1807
4986	2016.8.29	Changbai Mountain Nature Reserve	Alpine birch forest	Y Zou, JG Hou	42° 04' 00" 43.2	128° 04' 03" 50.4	1840
4987	2016.8.29	Changbai Mountain Nature Reserve	Alpine birch forest	Y Zou, JG Hou	42° 04' 00" 43.2	128° 04' 03" 50.4	1840
4988	2016.8.29	Changbai Mountain Nature Reserve	Alpine birch forest	Y Zou, JG Hou	42° 04' 00" 43.2	128° 04' 03" 50.4	1840
4989	2016.8.29	Changbai Mountain Nature Reserve	Alpine birch forest	Y Zou, JG Hou	42° 04' 00" 43.2	128° 04' 03" 50.4	1840
4990	2016.8.29	Changbai Mountain Nature Reserve	Alpine birch forest	Y Zou, JG Hou	42° 04' 00" 43.2	128° 04' 03" 50.4	1840
4991	2016.8.29	Changbai Mountain Nature Reserve	Alpine birch forest	Y Zou, JG Hou	42° 04' 00" 43.2	128° 04' 03" 50.4	1840
4992	2016.8.29	Changbai Mountain Nature Reserve	Alpine birch forest	Y Zou, JG Hou	42° 04' 00" 43.2	128° 04' 03" 50.4	1840
4993	2016.8.29	Changbai Mountain Nature Reserve	Alpine birch forest	Y Zou, JG Hou	42° 04' 00" 43.2	128° 04' 03" 50.4	1840
4994	2016.8.29	Changbai Mountain Nature Reserve	Alpine birch forest	Y Zou, JG Hou	42° 03' 47" 20.4	128° 04' 02" 45.6	1896
4995	2016.8.29	Changbai Mountain Nature Reserve	Alpine birch forest	Y Zou, JG Hou	42° 03' 47" 20.4	128° 04' 02" 45.6	1896
4996	2016.8.29	Changbai Mountain Nature Reserve	Alpine birch forest	Y Zou, JG Hou	42° 03' 47" 20.4	128° 04' 02" 45.6	1896
4997	2016.8.29	Changbai Mountain Nature Reserve	Alpine birch forest	Y Zou, JG Hou	42° 03' 47" 20.4	128° 04' 02" 45.6	1896

4998	2016.8.29	Changbai Mountain Nature Reserve	Alpine birch forest	Y Zou, JG Hou	42° 03' 47" 20.4	128° 04' 02" 45.6	1896
4999	2016.8.29	Changbai Mountain Nature Reserve	Alpine birch forest	Y Zou, JG Hou	42° 03' 47" 20.4	128° 04' 02" 45.6	1896
5000	2016.8.29	Changbai Mountain Nature Reserve	Alpine birch forest	Y Zou, JG Hou	42° 03' 47" 20.4	128° 04' 02" 45.6	1896
5001	2016.8.29	Changbai Mountain Nature Reserve	Alpine birch forest	Y Zou, JG Hou	42° 03' 47" 20.4	128° 04' 02" 45.6	1896
5002	2016.8.29	Changbai Mountain Nature Reserve	Alpine birch forest	Y Zou, JG Hou	42° 03' 34" 15.6	128° 04' 04" 40.8	1958
5003	2016.8.29	Changbai Mountain Nature Reserve	Alpine birch forest	Y Zou, JG Hou	42° 03' 34" 15.6	128° 04' 04" 40.8	1958
5004	2016.8.29	Changbai Mountain Nature Reserve	Alpine birch forest	Y Zou, JG Hou	42° 03' 34" 15.6	128° 04' 04" 40.8	1958
5005	2016.8.29	Changbai Mountain Nature Reserve	Alpine birch forest	Y Zou, JG Hou	42° 03' 34" 15.6	128° 04' 04" 40.8	1958
5006	2016.8.29	Changbai Mountain Nature Reserve	Alpine birch forest	Y Zou, JG Hou	42° 03' 34" 15.6	128° 04' 04" 40.8	1958
5007	2016.8.29	Changbai Mountain Nature Reserve	Alpine birch forest	Y Zou, JG Hou	42° 03' 34" 15.6	128° 04' 04" 40.8	1958
5008	2016.8.29	Changbai Mountain Nature Reserve	Alpine birch forest	Y Zou, JG Hou	42° 03' 34" 15.6	128° 04' 04" 40.8	1958
5009	2016.8.29	Changbai Mountain Nature Reserve	Alpine birch forest	Y Zou, JG Hou	42° 03' 34" 15.6	128° 04' 04" 40.8	1958
5010	2016.8.29	Changbai Mountain Nature Reserve	Alpine shrubland	Y Zou, JG Hou	42° 03' 32" 06	128° 04' 05" 13.2	1979
5011	2016.8.29	Changbai Mountain Nature Reserve	Alpine shrubland	Y Zou, JG Hou	42° 03' 32" 06	128° 04' 05" 13.2	1979
5012	2016.8.29	Changbai Mountain Nature Reserve	Alpine shrubland	Y Zou, JG Hou	42° 03' 32" 06	128° 04' 05" 13.2	1979
5013	2016.8.29	Changbai Mountain Nature Reserve	Alpine shrubland	Y Zou, JG Hou	42° 03' 32" 06	128° 04' 05" 13.2	1979

5014	2016.8.29	Changbai Mountain Nature Reserve	Alpine shrubland	Y Zou, JG Hou	42° 03' 32" 06	128° 04' 05" 13.2	1979
5015	2016.8.29	Changbai Mountain Nature Reserve	Alpine shrubland	Y Zou, JG Hou	42° 03' 32" 06	128° 04' 05" 13.2	1979
5016	2016.8.29	Changbai Mountain Nature Reserve	Alpine shrubland	Y Zou, JG Hou	42° 03' 32" 06	128° 04' 05" 13.2	1979
5017	2016.8.29	Changbai Mountain Nature Reserve	Alpine shrubland	Y Zou, JG Hou	42° 03' 32" 06	128° 04' 05" 13.2	1979
5018	2016.8.29	Changbai Mountain Nature Reserve	Alpine grassland	Y Zou, JG Hou	42° 03' 22" 44.4	128° 04' 00" 57.6	2006
5019	2016.8.29	Changbai Mountain Nature Reserve	Alpine grassland	Y Zou, JG Hou	42° 03' 22" 44.4	128° 04' 00" 57.6	2006
5020	2016.8.29	Changbai Mountain Nature Reserve	Alpine grassland	Y Zou, JG Hou	42° 03' 22" 44.4	128° 04' 00" 57.6	2006
5021	2016.8.29	Changbai Mountain Nature Reserve	Alpine grassland	Y Zou, JG Hou	42° 03' 22" 44.4	128° 04' 00" 57.6	2006
5022	2016.8.29	Changbai Mountain Nature Reserve	Alpine grassland	Y Zou, JG Hou	42° 03' 22" 44.4	128° 04' 00" 57.6	2006
5023	2016.8.29	Changbai Mountain Nature Reserve	Small broadleaf forest	Y Zou, JG Hou	42° 03' 19" 15.6	128° 03' 58" 15.6	2018
5024	2016.8.29	Changbai Mountain Nature Reserve	Small broadleaf forest	Y Zou, JG Hou	42° 03' 19" 15.6	128° 03' 58" 15.6	2018
5025	2016.8.29	Changbai Mountain Nature Reserve	Small broadleaf forest	Y Zou, JG Hou	42° 03' 19" 15.6	128° 03' 58" 15.6	2018
5026	2016.8.29	Changbai Mountain Nature Reserve	Small broadleaf forest	Y Zou, JG Hou	42° 03' 19" 15.6	128° 03' 58" 15.6	2018
5027	2016.8.29	Changbai Mountain Nature Reserve	Small broadleaf forest	Y Zou, JG Hou	42° 03' 19" 15.6	128° 03' 58" 15.6	2018
5028	2016.8.29	Changbai Mountain Nature Reserve	Small broadleaf forest	Y Zou, JG Hou	42° 03' 19" 15.6	128° 03' 58" 15.6	2018
5029	2016.8.29	Changbai Mountain Nature Reserve	Small broadleaf forest	Y Zou, JG Hou	42° 03' 19" 15.6	128° 03' 58" 15.6	2018

5030	2016.8.29	Changbai Mountain Nature Reserve	Small broadleaf forest	Y Zou, JG Hou	42° 03' 19" 15.6	128° 03' 58" 15.6	2018
5031	2016.8.29	Changbai Mountain Nature Reserve	Tundra	Y Zou, JG Hou	42° 03' 14" 09.6	128° 03' 59" 52.8	2038
5032	2016.8.29	Changbai Mountain Nature Reserve	Tundra	Y Zou, JG Hou	42° 03' 14" 09.6	128° 03' 59" 52.8	2038
5033	2016.8.29	Changbai Mountain Nature Reserve	Tundra	Y Zou, JG Hou	42° 03' 14" 09.6	128° 03' 59" 52.8	2038
5034	2016.8.29	Changbai Mountain Nature Reserve	Tundra	Y Zou, JG Hou	42° 03' 14" 09.6	128° 03' 59" 52.8	2038
5035	2016.8.29	Changbai Mountain Nature Reserve	Tundra	Y Zou, JG Hou	42° 03' 14" 09.6	128° 03' 59" 52.8	2038
5036	2016.8.29	Changbai Mountain Nature Reserve	Tundra	Y Zou, JG Hou	42° 03' 14" 09.6	128° 03' 59" 52.8	2038
5037	2016.8.29	Changbai Mountain Nature Reserve	Tundra	Y Zou, JG Hou	42° 03' 14" 09.6	128° 03' 59" 52.8	2038
5038	2016.8.29	Changbai Mountain Nature Reserve	Tundra	Y Zou, JG Hou	42° 03' 14" 09.6	128° 03' 59" 52.8	2038
5039	2016.8.29	Changbai Mountain Nature Reserve	Mixed broadleaf-conifer forest	Y Zou, JG Hou	42° 05' 07" 33.6	128° 04' 28" 08.4	1613
5040	2016.8.29	Changbai Mountain Nature Reserve	Mixed broadleaf-conifer forest	Y Zou, JG Hou	42° 05' 07" 33.6	128° 04' 28" 08.4	1613
5041	2016.8.29	Changbai Mountain Nature Reserve	Mixed broadleaf-conifer forest	Y Zou, JG Hou	42° 05' 07" 33.6	128° 04' 28" 08.4	1613
5042	2016.8.29	Changbai Mountain Nature Reserve	Mixed broadleaf-conifer forest	Y Zou, JG Hou	42° 05' 07" 33.6	128° 04' 28" 08.4	1613
5043	2016.8.29	Changbai Mountain Nature Reserve	Mixed broadleaf-conifer forest	Y Zou, JG Hou	42° 05' 07" 33.6	128° 04' 28" 08.4	1613
5044	2016.8.29	Changbai Mountain Nature Reserve	Mixed broadleaf-conifer forest	Y Zou, JG Hou	42° 05' 07" 33.6	128° 04' 28" 08.4	1613
5045	2016.8.29	Changbai Mountain Nature Reserve	Mixed broadleaf-conifer forest	Y Zou, JG Hou	42° 05' 07" 33.6	128° 04' 28" 08.4	1613

5046	2016.8.29	Changbai Mountain Nature Reserve	Mixed broadleaf-conifer forest	Y Zou, JG Hou	42° 05' 07" 33.6	128° 04' 28" 08.4	1613
5047	2016.8.30	Beauty pine park	Mixed broadleaf-conifer forest	Y Zou, JG Hou	42° 26' 18" 18	128° 07' 03" 00	709
5048	2016.8.30	Beauty pine park	Mixed broadleaf-conifer forest	Y Zou, JG Hou	42° 26' 18" 18	128° 07' 03" 00	709
5049	2016.8.30	Beauty pine park	Mixed broadleaf-conifer forest	Y Zou, JG Hou	42° 26' 18" 18	128° 07' 03" 00	709
5050	2016.8.30	Beauty pine park	Mixed broadleaf-conifer forest	Y Zou, JG Hou	42° 26' 18" 18	128° 07' 03" 00	709
5051	2016.8.30	Beauty pine park	Mixed broadleaf-conifer forest	Y Zou, JG Hou	42° 26' 18" 18	128° 07' 03" 00	709
5052	2016.8.30	Beauty pine park	Mixed broadleaf-conifer forest	Y Zou, JG Hou	42° 26' 18" 18	128° 07' 03" 00	709
5053	2016.8.30	Beauty pine park	Mixed broadleaf-conifer forest	Y Zou, JG Hou	42° 26' 18" 18	128° 07' 03" 00	709
5054	2016.8.30	Beauty pine park	Mixed broadleaf-conifer forest	Y Zou, JG Hou	42° 26' 18" 18	128° 07' 03" 00	709
5055	2016.8.30	Beauty pine park	Mixed broadleaf-conifer forest	Y Zou, JG Hou	42° 26' 19" 15.6	128° 07' 03" 36	720
5056	2016.8.30	Beauty pine park	Mixed broadleaf-conifer forest	Y Zou, JG Hou	42° 26' 19" 15.6	128° 07' 03" 36	720
5057	2016.8.30	Beauty pine park	Mixed broadleaf-conifer forest	Y Zou, JG Hou	42° 26' 19" 15.6	128° 07' 03" 36	720
5058	2016.8.30	Beauty pine park	Mixed broadleaf-conifer forest	Y Zou, JG Hou	42° 26' 19" 15.6	128° 07' 03" 36	720
5059	2016.8.30	Beauty pine park	Mixed broadleaf-conifer forest	Y Zou, JG Hou	42° 26' 19" 15.6	128° 07' 03" 36	720
5060	2016.8.30	Beauty pine park	Mixed broadleaf-conifer forest	Y Zou, JG Hou	42° 26' 19" 15.6	128° 07' 03" 36	720
5061	2016.8.30	Beauty pine park	Mixed broadleaf-conifer forest	Y Zou, JG Hou	42° 26' 19" 15.6	128° 07' 03" 36	720
5062	2016.8.30	Beauty pine park	Mixed broadleaf-conifer forest	Y Zou, JG Hou	42° 26' 19" 15.6	128° 07' 03" 36	720
5063	2016.8.30	Beauty pine park	Mixed broadleaf-conifer forest	Y Zou, JG Hou	42° 26' 23" 20.4	128° 07' 1" 44.4	721
5064	2016.8.30	Beauty pine park	Mixed broadleaf-conifer forest	Y Zou, JG Hou	42° 26' 23" 20.4	128° 07' 1" 44.4	721
5065	2016.8.30	Beauty pine park	Mixed broadleaf-conifer forest	Y Zou, JG Hou	42° 26' 23" 20.4	128° 07' 1" 44.4	721
5066	2016.8.30	Beauty pine park	Mixed broadleaf-conifer forest	Y Zou, JG Hou	42° 26' 23" 20.4	128° 07' 1" 44.4	721
5067	2016.8.30	Beauty pine park	Mixed broadleaf-conifer forest	Y Zou, JG Hou	42° 26' 23" 20.4	128° 07' 1" 44.4	721
5068	2016.8.30	Beauty pine park	Mixed broadleaf-conifer forest	Y Zou, JG Hou	42° 26' 23" 20.4	128° 07' 1" 44.4	721
5069	2016.8.30	Beauty pine park	Mixed broadleaf-conifer forest	Y Zou, JG Hou	42° 26' 23" 20.4	128° 07' 1" 44.4	721
5070	2016.8.30	Beauty pine park	Mixed broadleaf-conifer forest	Y Zou, JG Hou	42° 26' 23" 20.4	128° 07' 1" 44.4	721
5071	2016.8.30	Beauty pine park	Mixed broadleaf-conifer forest	Y Zou, JG Hou	42° 26' 22" 08.4	128° 07' 00" 25.2	726
5072	2016.8.30	Beauty pine park	Mixed broadleaf-conifer forest	Y Zou, JG Hou	42° 26' 22" 08.4	128° 07' 00" 25.2	726
5073	2016.8.30	Beauty pine park	Mixed broadleaf-conifer forest	Y Zou, JG Hou	42° 26' 22" 08.4	128° 07' 00" 25.2	726
5074	2016.8.30	Beauty pine park	Mixed broadleaf-conifer forest	Y Zou, JG Hou	42° 26' 22" 08.4	128° 07' 00" 25.2	726
5075	2016.8.30	Beauty pine park	Mixed broadleaf-conifer forest	Y Zou, JG Hou	42° 26' 22" 08.4	128° 07' 00" 25.2	726
5076	2016.8.30	Beauty pine park	Mixed broadleaf-conifer forest	Y Zou, JG Hou	42° 26' 22" 08.4	128° 07' 00" 25.2	726
5077	2016.8.30	Beauty pine park	Mixed broadleaf-conifer forest	Y Zou, JG Hou	42° 26' 22" 08.4	128° 07' 00" 25.2	726

5078	2016.8.30	Beauty pine park	Mixed broadleaf-conifer forest	Y Zou, JG Hou	42° 26' 22" 08.4	128° 07' 00" 25.2	726
5079	2016.8.30	Beauty pine park	Mixed broadleaf-conifer forest	Y Zou, JG Hou	42° 26' 31" 55.2	128°07' 06" 18	717
5080	2016.8.30	Beauty pine park	Mixed broadleaf-conifer forest	Y Zou, JG Hou	42° 26' 31" 55.2	128°07' 06" 18	717
5081	2016.8.30	Beauty pine park	Mixed broadleaf-conifer forest	Y Zou, JG Hou	42° 26' 31" 55.2	128°07' 06" 18	717
5082	2016.8.30	Beauty pine park	Mixed broadleaf-conifer forest	Y Zou, JG Hou	42° 26' 31" 55.2	128°07' 06" 18	717
5083	2016.8.30	Beauty pine park	Mixed broadleaf-conifer forest	Y Zou, JG Hou	42° 26' 31" 55.2	128°07' 06" 18	717
5084	2016.8.30	Beauty pine park	Mixed broadleaf-conifer forest	Y Zou, JG Hou	42° 26' 31" 55.2	128°07' 06" 18	717
5085	2016.8.30	Beauty pine park	Mixed broadleaf-conifer forest	Y Zou, JG Hou	42° 26' 31" 55.2	128°07' 06" 18	717
5086	2016.8.30	Beauty pine park	Mixed broadleaf-conifer forest	Y Zou, JG Hou	42° 26' 31" 55.2	128°07' 06" 18	717
5087	2016.8.30	Beauty pine park	Mixed broadleaf-conifer forest	Y Zou, JG Hou	42° 26' 32" 27.6	128° 07' 8" 52.8	691
5088	2016.8.30	Beauty pine park	Mixed broadleaf-conifer forest	Y Zou, JG Hou	42° 26' 32" 27.6	128° 07' 8" 52.8	691
5089	2016.8.30	Beauty pine park	Mixed broadleaf-conifer forest	Y Zou, JG Hou	42° 26' 32" 27.6	128° 07' 8" 52.8	691
5090	2016.8.30	Beauty pine park	Mixed broadleaf-conifer forest	Y Zou, JG Hou	42° 26' 32" 27.6	128° 07' 8" 52.8	691
5091	2016.8.30	Beauty pine park	Mixed broadleaf-conifer forest	Y Zou, JG Hou	42° 26' 32" 27.6	128° 07' 8" 52.8	691
5092	2016.8.30	Beauty pine park	Mixed broadleaf-conifer forest	Y Zou, JG Hou	42° 26' 32" 27.6	128° 07' 8" 52.8	691
5093	2016.8.30	Beauty pine park	Mixed broadleaf-conifer forest	Y Zou, JG Hou	42° 26' 32" 27.6	128° 07' 8" 52.8	691
5094	2016.8.30	Beauty pine park	Mixed broadleaf-conifer forest	Y Zou, JG Hou	42° 26' 32" 27.6	128° 07' 8" 52.8	691
5095	2016.8.30	Beauty pine park	Mixed broadleaf-conifer forest	Y Zou, JG Hou	42° 26' 29" 09.6	128° 07' 08" 09.6	675
5096	2016.8.30	Beauty pine park	Mixed broadleaf-conifer forest	Y Zou, JG Hou	42° 26' 29" 09.6	128° 07' 08" 09.6	675
5097	2016.8.30	Beauty pine park	Mixed broadleaf-conifer forest	Y Zou, JG Hou	42° 26' 29" 09.6	128° 07' 08" 09.6	675
5098	2016.8.30	Beauty pine park	Mixed broadleaf-conifer forest	Y Zou, JG Hou	42° 26' 29" 09.6	128° 07' 08" 09.6	675
5099	2016.8.30	Beauty pine park	Mixed broadleaf-conifer forest	Y Zou, JG Hou	42° 26' 29" 09.6	128° 07' 08" 09.6	675
5100	2016.8.30	Beauty pine park	Mixed broadleaf-conifer forest	Y Zou, JG Hou	42° 26' 29" 09.6	128° 07' 08" 09.6	675
5101	2016.8.30	Beauty pine park	Mixed broadleaf-conifer forest	Y Zou, JG Hou	42° 26' 29" 09.6	128° 07' 08" 09.6	675
5102	2016.8.30	Beauty pine park	Mixed broadleaf-conifer forest	Y Zou, JG Hou	42° 26' 28" 30	128° 07' 11" 56.4	712
5103	2016.8.30	Beauty pine park	Mixed broadleaf-conifer forest	Y Zou, JG Hou	42° 26' 28" 30	128° 07' 11" 56.4	712
5104	2016.8.30	Beauty pine park	Mixed broadleaf-conifer forest	Y Zou, JG Hou	42° 26' 28" 30	128° 07' 11" 56.4	712
5105	2016.8.30	Beauty pine park	Mixed broadleaf-conifer forest	Y Zou, JG Hou	42° 26' 28" 30	128° 07' 11" 56.4	712
5106	2016.8.30	Beauty pine park	Mixed broadleaf-conifer forest	Y Zou, JG Hou	42° 26' 28" 30	128° 07' 11" 56.4	712
5107	2016.8.30	Beauty pine park	Mixed broadleaf-conifer forest	Y Zou, JG Hou	42° 26' 28" 30	128° 07' 11" 56.4	712
5108	2016.8.30	Beauty pine park	Mixed broadleaf-conifer forest	Y Zou, JG Hou	42° 26' 28" 30	128° 07' 11" 56.4	712
5109	2016.8.30	Beauty pine park	Mixed broadleaf-conifer forest	Y Zou, JG Hou	42° 26' 28" 30	128° 07' 11" 56.4	712
5110	2016.8.30	Beauty pine park	Mixed broadleaf-conifer forest	Y Zou, JG Hou	42° 26' 13" 55.2	128° 07' 08" 13.2	715

5111	2016.8.30	Beauty pine park	Mixed broadleaf-conifer forest	Y Zou, JG Hou	42° 26' 13" 55.2	128° 07' 08" 13.2	715
5112	2016.8.30	Beauty pine park	Mixed broadleaf-conifer forest	Y Zou, JG Hou	42° 26' 13" 55.2	128° 07' 08" 13.2	715
5113	2016.8.30	Beauty pine park	Mixed broadleaf-conifer forest	Y Zou, JG Hou	42° 26' 13" 55.2	128° 07' 08" 13.2	715
5114	2016.8.30	Beauty pine park	Mixed broadleaf-conifer forest	Y Zou, JG Hou	42° 26' 13" 55.2	128° 07' 08" 13.2	715
5115	2016.8.30	Beauty pine park	Mixed broadleaf-conifer forest	Y Zou, JG Hou	42° 26' 13" 55.2	128° 07' 08" 13.2	715
5116	2016.8.30	Beauty pine park	Mixed broadleaf-conifer forest	Y Zou, JG Hou	42° 26' 13" 55.2	128° 07' 08" 13.2	715
5117	2016.8.30	Beauty pine park	Mixed broadleaf-conifer forest	Y Zou, JG Hou	42° 26' 13" 55.2	128° 07' 08" 13.2	715
5118	2016.8.30	Beauty pine park	Mixed broadleaf-conifer forest	Y Zou, JG Hou	42° 26' 15" 25.2	128° 07' 9" 57.6	721
5119	2016.8.30	Beauty pine park	Mixed broadleaf-conifer forest	Y Zou, JG Hou	42° 26' 15" 25.2	128° 07' 9" 57.6	721
5120	2016.8.30	Beauty pine park	Mixed broadleaf-conifer forest	Y Zou, JG Hou	42° 26' 15" 25.2	128° 07' 9" 57.6	721
5121	2016.8.30	Beauty pine park	Mixed broadleaf-conifer forest	Y Zou, JG Hou	42° 26' 15" 25.2	128° 07' 9" 57.6	721
5122	2016.8.30	Beauty pine park	Mixed broadleaf-conifer forest	Y Zou, JG Hou	42° 26' 15" 25.2	128° 07' 9" 57.6	721
5123	2016.8.30	Beauty pine park	Mixed broadleaf-conifer forest	Y Zou, JG Hou	42° 26' 15" 25.2	128° 07' 9" 57.6	721
5124	2016.8.30	Beauty pine park	Mixed broadleaf-conifer forest	Y Zou, JG Hou	42° 26' 15" 25.2	128° 07' 9" 57.6	721
5125	2016.8.30	Beauty pine park	Mixed broadleaf-conifer forest	Y Zou, JG Hou	42° 26' 15" 25.2	128° 07' 9" 57.6	721
5728	2017.9.16	Hanconggou	Mixed broadleaf-conifer forest	Y Zou	42° 24' 08" 33	128° 06' 12" 56	725
5729	2017.9.16	Xibao forestry farm	Mixed broadleaf-conifer forest	Y Zou	42° 22' 17" 84	128° 00' 13" 74	828
5730	2017.9.16	Huangsongpu forestry farm	Mixed broadleaf-conifer forest	Y Zou	42° 14' 08" 77	128° 09' 52" 81	1040
5731	2017.9.16	Changbai Mountain Nature Reserve	Mixed broadleaf-conifer forest	Y Zou	42° 10' 54" 73	128° 10' 54" 44	1136
5732	2017.9.16	Changbai Mountain Nature Reserve	Mixed broadleaf-conifer forest	Y Zou	42° 05' 11" 04	128° 04' 27" 05	1595
5733	2017.9.16	Changbai Mountain Nature Reserve	Moss under alpine birch forest	Y Zou	42° 03' 38" 63	128° 03' 38" 41	1743
5734	2017.9.16	Changbai Mountain Nature Reserve	Mixed broadleaf-conifer forest	Y Zou	42° 04' 21" 52	128° 03' 53" 63	1700
5735	2017.9.16	Changbai Mountain Nature Reserve	Mixed broadleaf-conifer forest	Y Zou	42° 04' 13" 76	128° 03' 56" 95	1720
5736	2017.9.16	Changbai Mountain Nature Reserve	Mixed broadleaf-conifer forest	Y Zou	42° 04' 03" 34	128° 03' 59" 80	1790
5737	2017.9.16	Changbai Mountain Nature Reserve	Alpine birch forest	Y Zou	42° 04' 02" 38	128° 04' 02" 13	1817

5738	2017.9.16	Changbai Mountain Nature Reserve	Alpine birch forest	Y Zou	42° 04' 01" 27	128° 04' 03" 97	1824
5739	2017.9.16	Changbai Mountain Nature Reserve	Alpine birch forest	Y Zou	42° 03' 59" 68	128° 04' 04" 96	1830
5740	2017.9.16	Changbai Mountain Nature Reserve	Alpine birch forest	Y Zou	42° 02' 39" 99	128° 03' 29" 66	1858
5741	2017.9.16	Changbai Mountain Nature Reserve	Tundra	Y Zou	42° 01' 32" 62	128° 04' 08" 17	2615
5742	2017.9.16	Changbai Mountain Nature Reserve	Tundra	Y Zou	42° 01' 36" 98	128° 04' 03" 34	2620
5743	2017.9.16	Changbai Mountain Nature Reserve	Tundra	Y Zou	42° 01' 40" 06	128° 04' 00" 19	2625
5744	2017.9.16	Changbai Mountain Nature Reserve	Tundra	Y Zou	42° 01' 32" 40	128° 04' 07" 80	2626
5745	2017.9.16	Changbai Mountain Nature Reserve	Tundra	Y Zou	42° 01' 40" 29	128° 04' 01" 98	2619
5746	2017.9.16	Changbai Mountain Nature Reserve	Tundra	Y Zou	42° 01' 36" 51	128° 04' 03" 68	2636
5747	2017.9.16	Changbai Mountain Nature Reserve	tundra under stone	Y Zou	42° 01' 34" 29	128° 04' 04" 45	2620
5748	2017.9.16	Changbai Mountain Nature Reserve	Tundra	Y Zou	42° 01' 40" 39	128° 04' 00" 31	2625
5749	2017.9.16	Changbai Mountain Nature Reserve	Volcanics	Y Zou	42° 01' 32" 35	128° 04' 07" 83	2626
5750	2017.9.17	Hanconggou	snail shell in broadleaf forest	Y Zou	42° 24' 06" 93	128° 05' 52" 43	776
5751	2017.9.17	Hanconggou	Broadleaf forest	Y Zou	42° 24' 06" 93	128° 05' 52" 43	776
5752	2017.9.17	Hanconggou	Broadleaf forest	Y Zou	42° 24' 07" 72	128° 05' 52" 14	774
5753	2017.9.17	Hanconggou	Broadleaf forest	Y Zou	42° 24' 05" 65	128° 05' 52" 25	786
5754	2017.9.17	Hanconggou	Mixed broadleaf-conifer forest	Y Zou	42° 24' 11" 92	128° 05' 53" 34	787
5755	2017.9.17	Hanconggou	Mixed broadleaf-conifer forest	Y Zou	42° 24' 08" 40	128° 05' 51" 38	809
5756	2017.9.17	Hanconggou	Mixed broadleaf-conifer forest	Y Zou	42° 24' 06" 81	128° 05' 54" 86	768
5757	2017.9.18	Changbai Mountain Nature Reserve	Coniferous forest	Y Zou	42° 06' 26" 88	128° 05' 38" 72	1430

5758	2017.9.18	Changbai Mountain Nature Reserve	Coniferous forest	Y Zou	42° 06' 27" 41	128° 05' 37" 56	1403
5759	2017.9.18	Changbai Mountain Nature Reserve	Coniferous forest	Y Zou	42° 06' 25" 54	128° 05' 34" 13	1438
5760	2017.9.18	Changbai Mountain Nature Reserve	Alpine birch forest	Y Zou	42° 03' 30" 74	128° 03' 29" 04	1759

Table S2. NCBI GenBank accession information for sequences of all dictyostelids and an outgroup (*Physarum polycephalum*) included in the SSU phylogenetic analysis. Newly generated sequences are indicated with asterisks.

Taxon	Isolate no./ Strain no.	Accession no.	Gene
Physarum polycephalum	CL	X13160.1	SSU
*Cavenderia fasciculata**	5754-2wang-23 (HMJAU MR313)	MW660750	SSU
*C. fasciculata**	5259-1-23 (HMJAU MR313)	MW660751	SSU
C. basinodulosa	Mad5-1A	MN338955.1	SSU
C. canoespora	Mad14-3C	MN338956.1	SSU
C. protodigitata	TH18BA	MH745572.1	SSU
C. aureostabilis	TH10B	MH745571.1	SSU
C. multistipes	UK26b	AM168070.1	SSU
C. deminutiva	MexM19A	AM168092.1	SSU
C. stellata	SAB7B	AM168081.1	SSU
C. mexicana	MexTF4B1	AM168089.1	SSU
C. bifurcata	UK5	AM168084.1	SSU
C. granulophora	CHII-4	AM168072.1	SSU
C. medusoides	OH592	AM168088.1	SSU
C. aureostipes var. *helvetia*	HM592	KF662214.1	SSU
C. aureostipes	YA6	AM168083.1	SSU
C. myxobasis	NT2A	HQ141522.1	SSU
C. subdiscoidea	TH1A	HQ141515.1	SSU
C. aureostipes	OH396	KF662201.1	SSU
C. aureostipes	B15A	KF662199.1	SSU
C. pseudoaureostipes	TH39A	HQ141518.1	SSU
C. sp. TH18B	TH18B	HQ141517.1	SSU
C. bhumiboliana	THC11X	HQ141523.1	SSU
C. fasciculata	SH3	AM168087.1	SSU
C. fasciculoidea		GQ496157.1	SSU
C. delicata	TNS-C-226	AM168093.1	SSU
C. fasciculata	SmokOW9A	AM168086.1	SSU
C. sp. TAS30A	TAS30A	HQ141516.1	SSU

C. antarctica	NZ43B	AM168080.1	SSU
C. macrocarpa	MGE2	HQ141519.1	SSU
C. parvispora	OS126	AM168091.1	SSU
C. amphispora	BM9A	HQ141521.1	SSU
C. microspora	TNS-C-38	AM168090.1	SSU
C. boomerangispora	K26B	HQ141520.1	SSU
C. exigua	TNS-C-199	AM168085.1	SSU
Acytostelium amazonicum	HN1B1	HQ141511.1	SSU
A. subglobosum	LB1	AM168110.1	SSU
A. magnisorum	08A	HQ141513.1	SSU
A. amazonicum	landolt X	HQ141510.1	SSU
A. leptosomum	212rjb	HQ141512.1	SSU
A. serpentarium	SAB3A	AM168113.1	SSU
A. digitatum	OH517	AM168114.1	SSU
A. singulare	FDIB	HQ141514.1	SSU
A. longisorophorum	DB10A	AM168109.1	SSU
A. anastomosans	PP1	AM168115.1	SSU
A. leptosomum	FG12	AM168111.1	SSU
Rostrostelium ellipticum	AE2	AM168112.1	SSU
*Heterostelium pallidum**	5759-1-23 (HMJAU MR314)	MW660748	SSU
*H. pallidum**	5759-2-23 (HMJAU MR314)	MW660749	SSU
*H. recretum**	5756-1-17 (HMJAU MR315)	MW857293	SSU
H. radiatum	M26B	MN338953.1	SSU
H. multibrachiatum	5916lun	MN752217.1	SSU
H. multibrachiatum	5904lun	MN752218.1	SSU
H. stolonicoideum	K12A	HQ141507.1	SSU
H. versatile	Mad52	MN338954.1	SSU
H. violaceotypum		KP167478.1	SSU
H. pallidum	TNS-C-98	AM168103.1	SSU
H. candidum	bsb6b	HQ141498.1	SSU
H. sp. TH12A	TH12A	HQ141504.1	SSU
H. candidum		AY040337.1	SSU
H. filamentosum	SU-1	AM168100.1	SSU

H. luridum	LR-2	AM168101.1	SSU
H. lapidosum		KP167477.1	SSU
H. pseudocolligatum		KP167474.1	SSU
H. racemiferum		KP167476.1	SSU
H. colligatum	OH538	AM168098.1	SSU
H. tikalense	OH595	AM168106.1	SSU
H. colligatum	HN13C1	HQ141505.1	SSU
H. tikalense	HN1C1	HQ141509.1	SSU
H. plurimicrocystogenum		KP167475.1	SSU
H. pseudoplasmodiomagnum		KP167472.1	SSU
H. unguliferum		KP167473.1	SSU
H. australicum	NB1AP	HQ141508.1	SSU
H. equisetoides	B7JB	AM168099.1	SSU
H. arachnoideum	YA1	AM168102.1	SSU
H. cumulocystum		KP167479.1	SSU
H. ampliverticillatum		KP167480.1	SSU
H. multicystogenum	AS2	HQ141506.1	SSU
H. parvimigratum		KP167483.1	SSU
H. pseudoplasmodiofascium		KP167482.1	SSU
H. migratissimum		KP167481.1	SSU
H. anisocaule	NZ47B	AM168096.1	SSU
H. pseudocandidum	TNS-C-91	AM168107.1	SSU
H. asymetricum	OH567	AM168097.1	SSU
H. asymetricum	HN20C	HQ141503.1	SSU
H. sp. Laos3	Laos3	HQ141496.1	SSU
H. pallidum	PN500	AM168104.1	SSU
H. pallidum	PPHU8; ATCC 44421	EU004605.1	SSU
H. tenuissimum	TNS-C-97	AM168105.1	SSU
H. tenuissimum		AY040339.1	SSU
H. gloeosporum	TCK52	AM168074.1	SSU
H. granulosum	MF5A	HQ141502.1	SSU
H. flexuosum	AU4B	HQ141500.1	SSU
H. rotatum	QC2C	HQ141501.1	SSU

H. boreale	BSB10A	HQ141499.1	SSU
H. oculare	oculare	HQ141497.1	SSU
H. oculare	DB4B	AM168079.1	SSU
Speleostelium caveatum	WS695	AM168077.1	SSU
Tieghemostelium menorah	M1	AM168073.1	SSU
T. unicornutum	OH599	JF892725.1	SSU
T. lacteum		AM168045.1	SSU
T. angelicum	38B0	JF892716.1	SSU
T. montium	57a	JF892717.1	SSU
T. dumosum	OH602	JF892722.1	SSU
T. simplex	OH598	JF892720.1	SSU
Hagiwaraea coeruleostipes	CRLC53B	AM168036.1	SSU
Ha. rhizopodium	AusKY-4	AM168063.1	SSU
Ha. radiculata	ML5A	HQ141494.1	SSU
Ha. vinaceofusca	CC4	AM168062.1	SSU
Ha. lavandula	B15	AM168047.1	SSU
Raperostelium stabile	M12A	MN338957.1	SSU
R. minutum	71-2	AM168051.1	SSU
R. ohioense	Okla4C	HQ141493.1	SSU
R. sp. TH14B	TH14B	HQ141491.1	SSU
R. gracile	TNS-C-183	AM168078.1	SSU
R. tenue	PR4	AM168075.1	SSU
R. tenue	PJ6	AM168094.1	SSU
R. filiforme	OH603	JF892724.1	SSU
R. capillare	37A	JF892721.1	SSU
R. maeandriforme	OH604	JF892719.1	SSU
R. reciprocatum var. *transitum*	OH601	JF892723.1	SSU
R. tenue	Pan52	AM168076.1	SSU
R. reciprocatum	38A	JF892718.1	SSU
R. australe	NZ80B	AM168029.1	SSU
R. sp. TH8C	TH8C	HQ141492.1	SSU
R. monochasioides	HAG653	AM168052.1	SSU
R. potamoides	FP1A	AM168069.1	SSU

R. sp. MR-2008		EU672875.1	SSU
R. ibericum	214rjb	HQ141495.1	SSU
Coremiostelium polycephalum	Landolt #1675 GUAM	HQ141490.1	SSU
Co. polycephalum	MY1-1	AM168056.1	SSU
Co. polycephalum	Landolt #1130 SS3B	HQ141488.1	SSU
Co. polycephalum	Landolt #2132 B-9c	HQ141489.1	SSU
Polysphondylium fuscans	Sweden-11D	JX173877.1	SSU
P. sp. Tibet 10A	Tibet 10A	HQ141487.1	SSU
P. laterosorum	AE4	AM168046.1	SSU
P. violaceum	P6	AM168108.1	SSU
P. sp. Laos4	Laos4	HQ141485.1	SSU
P. patagonicum		GQ496156.1	SSU
P. violaceum	209	HQ141486.1	SSU
*Dictyostelium discoideum**	5751-1-23 (HMJAU MR302)	MT125951	SSU
*D. discoideum**	5752-3-17 (HMJAU MR302)	MT125952	SSU
*D. mucoroides**	5035-23 (HMJAU MR303)	MW660746	SSU
*D. mucoroides**	5735-3-23 (HMJAU MR303)	MW660747	SSU
*D. robusticaule**	5729-huang-2021 (HMJAU MR305)	MW931856	SSU
*D. robusticaule**	5729-bai-2021 (HMJAU MR305)	MW931857	SSU
D. barbarae	1-5	MK322959.1	SSU
D. insulinativitatis		MK322958.1	SSU
D. multiforme	4009	MG490371.1	SSU
D. multiforme	4007	MG490370.1	SSU
D. minimum		MG490369.1	SSU
D. gargantuum		GQ496161.1	SSU
D. robustum	TNS-C-219	AM168064.1	SSU
D. brunneum	WS700	AM168031.1	SSU
D. giganteum	WS589	AM168042.1	SSU
D. sp. Laos1	Laos1	HQ141483.1	SSU
D. purpureum var. *pseudosessile*	MR273 (4446)	MH280023.1	SSU
D. purpureum	QSpu1	FJ424829.1	17S ribosomal RNA gene, internal transcribed spacer 1, 5.8S ribosomal RNA gene, and internal transcribed spacer 2, complete sequence; and 26S ribosomal RNA gene, partial sequence
D. purpureum	QSpu2	FJ424839.1	17S ribosomal RNA gene, partial sequence; internal transcribed spacer 1, 5.8S ribosomal RNA gene, and internal transcribed spacer 2, complete

			sequence; and 26S ribosomal RNA gene, partial sequence
D. purpureum	QSpu23	FJ424832.1	7S ribosomal RNA gene, internal transcribed spacer 1, 5.8S ribosomal RNA gene, internal transcribed spacer 2, and 26S ribosomal RNA gene, complete sequence
D. purpureum	QSpu28	FJ424836.1	17S ribosomal RNA gene, internal transcribed spacer 1, 5.8S ribosomal RNA gene, and internal transcribed spacer 2, complete sequence; and 26S ribosomal RNA gene, partial sequence
D. purpureum		DQ340386.1	17S ribosomal RNA, 5.8S ribosomal RNA, 26S ribosomal RNA, and 5S ribosomal RNA genes, complete sequence
D. purpureum	QSpu4	FJ424826.1	17S ribosomal RNA gene, partial sequence; internal transcribed spacer 1, 5.8S ribosomal RNA gene, and internal transcribed spacer 2, complete sequence; and 26S ribosomal RNA gene, partial sequence
D. purpureum		AY040335.1	SSU
D. purpureum	QSpu36	FJ424828.1	17S ribosomal RNA gene, internal transcribed spacer 1, 5.8S ribosomal RNA gene, and internal transcribed spacer 2, complete sequence; and 26S ribosomal RNA gene, partial sequence
D. purpureum	WS321	AM168061.1	SSU
D. purpureum	cavender	HQ141481.1	SSU
D. septentrionale	IY49	AM168066.1	SSU
D. septentrionale	AK2	AM168067.1	SSU
D. purpureum	C143	AM168060.1	SSU
D. discoideum	NC4	AM168071.1	SSU
D. dimigraformum	AR5b	AM168038.1	SSU
D. citrinum	OH494	AM168033.1	SSU
D. discoideum	V34	AM168039.1	SSU
D. firmibasis	TNS-C-14	AM168041.1	SSU
D. intermedium	PJ11	AM168044.1	SSU
D. sp. Laos5	Laos5	HQ141484.1	SSU
D. crassicaule	93HO-33	AM168037.1	SSU
D. aureocephalum	TNS-C-180	AM167876.1	SSU
D. aureum	SL1	AM168028.1	SSU
D. leptosomum	NZN49A	HQ141480.1	SSU
D. capitatum	91HO-50	AM168032.1	SSU
D. pseudobrefeldianum	91HO-8	AM168059.1	SSU
D. austroandinum		GQ496158.1	SSU
D. leptosomopsis	Araucaria 1	HM159992.1	SSU
D. mucoroides	S28b	AM168054.1	SSU
D. brefeldianum	TNS-C-115	AM168030.1	SSU

D. mucoroides	sweden 20	HQ141482.1	SSU
D. macrocephalum	B33	AM168049.1	SSU
D. medium	TNS-C-205	AM168050.1	SSU
D. mucoroides var. *stoloniferum*	FOII-1	AM168055.1	SSU
D. rosarium	M45	AM168065.1	SSU
D. sphaerocephalum	GR11	AM168068.1	SSU
D. mucoroides	TNS-C-114	AM168053.1	SSU
D. quercibrachium	NZ201B	HQ141479.1	SSU
D. clavatum	TNS-C-189	AM168034.1	SSU
D. clavatum	TNS-C-220	AM168035.1	SSU
D. longosporum	TNS-C-109	AM168048.1	SSU
D. ammophilum	KBK4A	HQ141478.1	SSU
D. valdivianum		GQ496155.1	SSU
D. implicatum	93HO-1	AM168043.1	SSU
D. chordatum		GQ496159.1	SSU
D. barbibulus	Sweden-4R	JX173878.1	SSU
Synstelium polycarpum	VE1b	AM168057.1	SSU
S. polycarpum	OhioWILDS	AM168058.1	SSU

Table S3. NCBI GenBank accession information for sequences of all dictyostelids and an outgroup (*Physarum polycephalum*) included in the atp1 phylogenetic analysis. Newly generated sequences are indicated with asterisks.

Taxon	Isolate no./ Strain no.	Accession no.	Gene
Physarum polycephalum		NC_002508.1	mitochondrion, complete genome
*Dictyostelium discoideum**	5751-1-23 (HMJAU MR302)	MZ318395	*atp1*
*D. discoideum**	5752-3-17 (HMJAU MR302)	MZ318394	*atp1*
*D. mucoroides**	5735-3-23 (HMJAU MR303)	MZ318393	*atp1*
*D. robusticaule**	5729-huang-2021 (HMJAU MR305)	MZ318392	*atp1*
*D. robusticaule**	5729-bai-2021 (HMJAU MR305)	MZ318391	*atp1*
D. sp. TAS30A	TAS30A	KF662068.1	*atp1*
D. discoideum	AX3	AB000109.1	mitochondrial DNA, complete genome
D. citrinum		DQ336395.4	mitochondrion, complete genome
*Heterostelium pallidum**	5759-1-23 (HMJAU MR314)	MZ318388	*atp1*
*H. pallidum**	5759-2-23 (HMJAU MR314)	MZ318387	*atp1*
*H. recretum**	5756-1-17 (HMJAU MR315)	MZ318386	*atp1*
H. pallidum	CK8	AY700145.1	mitochondrion DNA, complete sequence
H. pallidum	PN500	EU275726.1	mitochondrion, complete genome
*Cavenderia fasciculata**	5754-2wang-23 (HMJAU MR313)	MZ318390	*atp1*
*C. fasciculata**	5259-1-23 (HMJAU MR313)	MZ318389	*atp1*
C. granulophora	CH11-4	KF662075.1	*atp1*
C. sp. TH18D	TH18D	KF662113.1	*atp1*
C. aureostipes	AU7B	KF662112.1	*atp1*
C. sp. TH1A	TH1A	KF662123.1	*atp1*
C. aureostipes	B15A	KF662116.1	*atp1*
C. sp. TH39A	TH39A	KF662117.1	*atp1*
C. myxobasis	NT2A	KF662122.1	*atp1*
C. aureostipes	Thai2C1	KF662118.1	*atp1*
C. stellata	SAB7B	KF662110.1	*atp1*
C. aureostipes	OH396	KF662120.1	*atp1*
C. aureostipes var. *helvetia*	HM594	KF662109.1	*atp1*

C. aureostipes var. *helvetia*	52A1	KF662100.1	*atp1*
C. aureostipes var. *helvetia*	HM593	KF662099.1	*atp1*
C. aureostipes	CR6B1	KF662115.1	*atp1*
C. bifurcata	UK5	KF662111.1	*atp1*
C. fasciculata	SH3	EU275727.1	mitochondrion, complete genome
C. aureostipes	KAL7A	KF662084.1	*atp1*
C. aureostipes	Chile10B1	KF662082.1	*atp1*
C. aureostipes	GSE7B	KF662083.1	*atp1*
C. mexicana	TF4B1	KF662073.1	*atp1*
C. delicata	TNS 266	KF662080.1	*atp1*
C. aureostipes	BP7B	KF662127.1	*atp1*
C. aureostipes	Got5B4	KF662094.1	*atp1*
C. antarctica	NZ43B	KF662081.1	*atp1*
C. aureostipes	LR2	KF662124.1	*atp1*
C. aureostipes	AKL43C	KF662095.1	*atp1*
C. aureostipes	KP3	KF662130.1	*atp1*
C. aureostipes	Got9A1	KF662092.1	*atp1*
C. fasciculata	HM595	KF662076.1	*atp1*
C. aureostipes	I3	KF662133.1	*atp1*
C. aureostipes	OH110 BW6	KF662131.1	*atp1*
C. fasciculata	Nor84E	KF662077.1	*atp1*
C. aureostipes	OH438	KF662088.1	*atp1*
C. medusoides	OH592	KF662074.1	*atp1*
C. aureostipes var. *helvetia*	HM592	KF662098.1	*atp1*
C. exigua	KP94	KF662096.1	*atp1*
C. parvispora	OS126	KF662071.1	*atp1*
C. microspora	Hagiwara143	KF662072.1	*atp1*

Table S4. Dictyostelids have been reported in Taiwan and on Changbai Mountain.

Order	Family	Genus	Taiwan	References	Changbai Mountain	References
Dictyosteliales	Dictyosteliaceae	*Dictyostelium*	*Dictyostelium aureostipes* var. *aureostipes*	Hagiwara et al. 1992(1)		
Dictyosteliales	Dictyosteliaceae	*Dictyostelium*	*D. brefeldianum*	Yeh & Chien 1983(2)		
Dictyosteliales	Dictyosteliaceae	*Dictyostelium*	*D. clavatum*	Fan et al. 2002(3)		
Dictyosteliales	Dictyosteliaceae	*Dictyostelium*			*D. discoideum*	This paper
Dictyosteliales	Dictyosteliaceae	*Dictyostelium*			*D. firmibasis*	An et al. 2018(4)
Dictyosteliales	Dictyosteliaceae	*Dictyostelium*	*D. giganteum*	Yeh & Chien 1983(2)		
Dictyosteliales	Dictyosteliaceae	*Dictyostelium*	*D. macrocephalum*	Hagiwara et al. 1985(5)		
Dictyosteliales	Dictyosteliaceae	*Dictyostelium*	*D. magnum*	Hagiwara et al. 1992(1)		
Dictyosteliales	Dictyosteliaceae	*Dictyostelium*			*D. mucoroides*	This paper
Dictyosteliales	Dictyosteliaceae	*Dictyostelium*	*D. purpureum*	Hagiwara et al. 1992(1)		
Dictyosteliales	Dictyosteliaceae	*Dictyostelium*			*D. robusticaule*	This paper
Dictyosteliales	Dictyosteliaceae	*Polysphondylium*	*Polysphondylium violaceum*	Yeh & Chien 1983(2)	*P. violaceum*	Liu & Li 2014(6)
Dictyosteliales		*Coremiostelium*	*Coremiostelium polycephalum*	Hagiwara et al. 1992(1)		
Dictyosteliales	Raperosteliaceae	*Raperostelium*	*Raperostelium minutum*	Yeh 2003(7)		
Dictyosteliales	Raperosteliaceae	*Raperostelium*	*R. monochasioides*	Hagiwara et al. 1992(1)		
Dictyosteliales	Raperosteliaceae	*Hagiwaraea*	*Hagiwaraea coeruleostipes*	Fan & Yeh 2001(8)		
Dictyosteliales	Raperosteliaceae	*Hagiwaraea*	*Ha. lavandula*	Hagiwara et al. 1992(1)		
Dictyosteliales	Raperosteliaceae	*Hagiwaraea*	*Ha. rhizopodium*	Hagiwara et al. 1992(1)		
Acytosteliales	Acytosteliaceae	*Heterostelium*	*Heterostelium arachnoideum*	Liu et al. 2019(9)		
Acytosteliales	Acytosteliaceae	*Heterostelium*			*H. candidum*	He & Li 2008(10)
Acytosteliales	Acytosteliaceae	*Heterostelium*	*H. pallidum*	Yeh & Chien 1983(2)	*H. pallidum*	This paper
Acytosteliales	Acytosteliaceae	*Heterostelium*	*H. pseudocandidum*	Lin & Yeh 1999(11)		
Acytosteliales	Acytosteliaceae	*Heterostelium*			*H. recretum*	This paper
Acytosteliales	Acytosteliaceae	*Acytostelium*	*Acytostelium leptosomum*	Hagiwara et al. 1992(1)		
Acytosteliales	Cavenderiaceae	*Cavenderia*	*Cavenderia delicata*	Hsu et al. 2001(12)		
Acytosteliales	Cavenderiaceae	*Cavenderia*	*C. exigua*	Yeh & Chen 2004(13)		
Acytosteliales	Cavenderiaceae	*Cavenderia*			*C. fasciculata*	This paper

Table S5. Dictyostelids have been reported with habitat on Changbai Mountain.

Changbai Mountain	References	Habitat
Dictyostelium discoideum	This paper	Broadleaf forest
D. firmibasis	An et al. 2018(4)	Mixed broadleaf-conifer forest
D. mucoroides	This paper	Tundra
D. mucoroides	This paper	Mixed broadleaf-conifer forest
D. robusticaule	This paper	Mixed broadleaf-conifer forest
Polysphondylium violaceum	Liu & Li 2014(6)	Mixed broadleaf-conifer forest
Heterostelium candidum	He & Li 2008a(10)	Forest
H. pallidum	This paper	Coniferous forest
H. recretum	This paper	Mixed broadleaf-conifer forest
Cavenderia fasciculata	This paper	Mixed broadleaf-conifer forest
C. fasciculata	This paper	Coniferous forest

Text S1. Alignment of SSU sequences

>MN338956.1_Cavenderia_canoespora_Mad14_3C
-------TATAAGCTCTTG-TACGGCTA-GA---CTGCAGACGGCTCATT--ACAA--CG
GTTGT-----AT---CTTACAGGGCATCCGG-GTCGCA---AGACCTTCT-GGATAACCG
CAGTAAATCGGGGCTAATACATATAAA-CGGAGGGATGGAGAGGGC----AACCTTGAAG
TTTCTGCGATGGACATTTA-GCTATTC--GA-CCAACCC--CG-CAA-GGGAACG--GTT
GGAACCGGTTCATATTGCTAATCGACT-CTAGCTTGCTAGTAGTCTGATGAGCTCTATAG
ACAACCGCCCTATCAACTT--GATGGTAAGGTTTTGGCTTACCATGGTTGTAACGGGTAA
CGGGGAATCAGGGTTCGATTCCGGAGAGGACGCCTGAGAAACGGCGTCCACATCTACGGG
TGGCAGCAGGCGCGTAAATT-GCCCAATCTCAACAGAGAGGAGGCGGTGACAATAAATCC
CGATGGCTTTGGGGGCAACCCCAGG--CCAATCAGAATAAGTACACATTAAATCCCTTAA
CC-AATATAATTGGAGGGCAAGTCTGGTGCCAGCAGCCGCGGTAATTCCAGCTCCAA-TA
GCATATACTAATGTTGTTGCAGTTAAAA-CGC-TCGTAGCT-----GAATATCTTTT--G
AG-CTAC------TATCGACT-ACAT-TCC----TTCAAA-G----GTCGCAAGACT-GA
----TGAAGA--TGT-GGT-CGGCTCAAACAC-TGTAGGTGATGATA-TTGG-----TAG
CAATA-CTAAT--AT-----------CATAA---TCGACTGTGCATAAA-CCTTGATGCT
CAAGGTA--GG-CCT-TT---ATAGGGTAGATACACAGT-GCATGGCATTGTGGAACAAG
GCAT--CTCGCGGCTTAGTTGGT-GGGCC-------GCGGGGGCAATGATTAATAGGGAG
GAGCGGGGGCCTTCATATTGCAGGGCGAGAGGTGAAATTCGTTGACCCTTGCAAGATGTC
CGACAGCGAAAGCATTGGCCAAGTGCCTCTCCATTAGTCAAGAACGAAAGTTTGGGGATC
AAAGACGATCAGATACCGTCGTAGTCCAAACCATAAACTATGTCGACCAGCGATTAGGCG
GGCTACCTTCTTCGAGAGCTGCCTAGCAGCTT-GTGGGAAACCAT-GAGTGCTTGGACTC
TGGGGGGAGTATGGTCGCAAGGCTGAAACTTAAAGGAATTGACGGAAGGGCACACCATGG
AGTGGAG-CCTGCGGCTTAATTT-GACTCAACACGGGAAA-GCTTACCAAGCTCAGATAT
GATAAGGATTGACAGA-CTAA-AAGATCTTTCATGATCTCATAAGTGGTGGTGCATGGTC
GTTCTTAG-TTGGTGGAGTGATTTGTCAGGTCAATTCCGGTAACGGACGAGACCTCGACC
TGCTAACTAGTGGGATTCATTCTTTCG--ATTGACGAGGCGGGTATTGC-TTTGATTATG
A------GGGGCAACCTT-----TATAGTCGGG-TAATATTTGTAG-TAGTCTGGAGGAG
TGG--GTTTCAGACAATTTAATT---AATCTTCCTAGAGGTACTTCTG-GCTCTAAGCCA
GAGG-AAGTCCGAGGCAATAACAGGTCTGTGATGCCCTTAGATATCTT-GGGCCGCACGC
GTGCTACAATGTAGGCGCTAATGAGTCG--TAACAATTTCC--AGCTCCGT-AAG---GA
GTCTGGTAATC-TTGGAATCACCTGCGTGATTGGGCTTG-ACTACTGTAACTGT-GGTCA
TCAACGAGGAATTCCTTGTATGCGCGAGTCACTATCTCGCGCAGAATCTGTCCCTGCCCT
TTGTACACACCGCCCGTCGCTCCTACCGATCGAACGATCAGGTAAAGTGGACAGATTA--
--------------GGA-ACCG-C-AAG-G-AT-C---------TT-TAG--AAGT-CC-
A--------------------------
>MN338955.1_Cavenderia_basinodulosa_Mad5_1A
TCTAAG-T--AAGCTCTTGCTACTGTTATGA---CTGCAGACGGCTCATT--ATCATATC
TGTGTATGT-AT---CTTACAGGGCATCCGG-GTCGCA---AGACCTTCT-GGATAACCG
CAGTAAATCGGGGCTAATACATATAAA-CGGAGGGATGGAGAGGGC----TACCTTGAAG
TTTCTGCGATGGACATTTA-GCTATTC--GA-CCTCCCC--CG-CAA-GGGAACG--GTT
GGAACCGGTTCATATTGCTAATCGACT-CTAGCTTGCTAGTTTTCTGATGAGCTCTATAG
ACAACCGCCCTATCAACTT--GATGGTAAGGTTTTGGCTTACCTTGGTTGTATCGGGTAA
CGGGGAATCAGGGTTCGATTCCGGAGAGGACGCCTGACAAACGGCGTCCACATCTACGGG
TGGCAGCAGGCGCGTAAATT-GCCCAATCTCAACAGAGAGGAGGCGGTGACAATAAATCC
CGATGGCTTTGGGGGCAACCCCAGG--CCAATCAGAATAAGTACACATTAAATCCCTTAA
CC-AATATAATTGGAGGGCAAGTCTGGTGCCAGCAGCCGCGGTAATTCCAGCTCCAA-TA
GCATATACTAATGTTGTTGCAGTTAAAA-CGC-TCGTAGCT-----GAATATCTTTT--G
AG-CTAC------TATCGACT-ACAT-TCC-----TTCAAA-G----GTCGCAAGACT-GA
----TGAAGA--TGT-GGT-CGGCTCAAACAC-TGTAGGTGATGATA-TTGG-----TAG
CAATA-CTAAT--AT-----------CATAA---TCGACTGTGCATAAA-CCTTGATGCT
CAAGGTA--GG-CCT-TT---ATAGGGTAGATACACAGT-GCATGGCATTGTGGAACAAG
GCAT--CTCGCGGCTTAGTTGGT-GGGCC-------GCGGGGGCAATGATTAATAGGGAG
GAGCGGGGGCCTTCATATTGCAGGGCGAGAGGTGAAATTCGTTGACCCTTGCAAGATGTC
CGACAGCGAAAGCATTGGCCAAGTGCCTCTCCATTAGTCAAGAACGAAAGTTTGGGGATC
AAAGACGATCAGATACCGTCGTAGTCCAAACCATAAACTATGTCGACCAGCGATTAGGCG
GGCTACCTTCTTCGAGAGCTGCCTAGCAGCTT-GTGGGAAACCAT-GAGTGCTTGGACTC
TGGGGGGAGTATGGTCGCAAGGCTGAAACTTAAAGGAATTGACGGAAGGGCACACCATGG
AGTGGAG-CCTGCGGCTTAATTT-GACTCAACACGGGAAA-GCTTACCAAGCTCAGATAT
GATAAGGATTGACAGA-CTAA-AAGATCTTTCATGATCTCATAAGTGGTGGTGCATGGTC
GTTCTTAG-TTGGTGGAGTGATTTGTCAGGTCAATTCCGGTAACGGACGAGACCTCGACC
TGCTAACTAGTGGGATTCATTCTTTCG--ATTGACGAGGCGGGTATTGC-TTTGATTATG
A------GGGGCAACCTT-----TATAGTCGGG-TAATATTTGTAG-TAGTCTGGAGGAG
TGG--GTTTCAGACAATTTAATT---AATCTTCCTAGAGGTACTTCTG-GCTCTAAGCCA
GAGG-AAGTCCGAGGCAATAACAGGTCTGTGATGCCCTTAGATATCTT-GGGCCGCACGC
GTGCTACAATGTAGGCGCTAATGAGTCG--TAACAATTTCC--AGCTCCGT-AAG---GA
GTCTGGTAATC-TTGGAATCACCTGCGTGATTGGGCTTG-ACTACTGTAACTGT-GGTCA
TCAACGAGGAATTCCTTGTATGCGCGAGTCACTATCTCGCGCAGAATCTGTCCCTGCCCT
TTGTACACACCGCCCGTCGCTCCTACCGATCGAACGATCAGGTAAAATGGACA--TT---
--------------G-ATACCCTC-CGC---A-G-AGTC--C--TC-CCG--ATGT-AA-

T-A-CGGTTAT--A----A-----TT-
>AM168092.1_Cavenderia_deminutiva_MexM19A
TCTAAG-TATAAGTCCTTG-TACGACTA-GA---CTGCAGACGGCTCATT--ACAA--CA
GTTGT-----TG---TCTACAGGACATCCGC-G-CTTT---TG-CGTTTT-GGATAACCG
CAGTAAATCGGGGCTAATACATATAAA-CGAAGGAAT-GACTGG-T----AAC--GGAAG
TTTCTGCGATGGA--TTTA-GCTATTC--AA-CCGGCCT--TG-T-----GTTT---GTT
GGAACCGATCCATATTGCTAATCGGCTATTAGTTTACTAATAGCCTGATGAGTTCTATGA
ACAACTGCCCTATCAACTT--GATGGTAAGGTATTGGCTTACCATGGTTGTAACGGGTAA
CGGGGAATCAGGGTTCGATTCCGGAGAGGACGCCTGAGAAACGGCGTCCACATCTACGGG
TGGCAGCAGGCGCGTAAATT-ACCCAATCTCAATAGAGAGGAGGTGGCGACAATAAATCC
TGATGCCTATAGGCTCACGCCTAGG--GCAATTAGAATAAGTACAAAATAAATCCATTAA
CC-AATGTAATTGGAGGGCAAGTCTGGTGCCAGCAGCCGCGGTAATTCCAGCTCCAA-TA
GCGTATACTAATGTTGTTGCAGTTAAAA-CGC-TCGTAGCT--------TA--------A
AA-ACAC------GGATCATT-TTTT-AGCTAAATCGATT-G----CCACCTGGA-AACA
--GGTGAGGTGATTT-ATT-TGGCAT-GGCACGTGCTGGT--TGGGT-TTGTTG--GT-T
CGCT--CTAGCA-AG-----------CTTAG---TCAACTGTGCATAAA-CCTTGATGCT
CAAGGTAAAGA-CCG-TAGGTCT--GT--GATACTAAGT-GCATGGTATTGTAGAACATG
GTAA--CAGGTGGCTTTGTTGGTTGGGTC-------ACTAGTGCAATGATTAATAGGGAG
GAGCGGGGGCCTTCATATTGATGGGCGAGAGGTGAAATTCGTTGACCCTATCAAGATGTC
CTACAGCGAAAGCATTGGCCAAGTGCCTCTCCATTAGTCAAGAACGATAGTTTGGGGATC
AAAGACGATCAGATACCGTCGTAGTCCAAACCATAAACGATGTCGACCAGTGATTAGGCA
CGTCACCTTTCTAGAGAACTGCCTAGGAACTTAGTGGGAAACCATTGAGTTTTTGGACTC
TGGGGGGAGTATGGTCGCAAGGCTGAAACTTAAAGGAATTGACGGAAGGGCACACCATGG
AGTGGAG-CCTGCGGCTTAATTT-GACTCAACACGGGAAA-ACTTACCAAGCTCTGATAT
GATAAGGATTGACAGA-CTAA-AAGATCTTTCATGATCGCATAAGTGGTGGTGCATGGTC
GTTCTTAG-TTGGTGGAGTGATTTGTCAGGTCAATTCCGGTAACGGACGAGACCTCGACC
TGCTAACTAGTGACAATTAGATTGTCGT-ATGGGCGAAGTTAGTTCGGCAGCTACTTAAT
G--------GGCAACTGT--TA-GGTAGTTGACGTATTGATTGTAG----CCTGGCAATG
TAG--TTGTTATA---TT---TT-----CCTTCTTAGAGGGACTTCCA-GCTTTAAGTTG
GAGG-AAGTCCGAGGCAATAACAGGTCTGTGATGCCCTTAGATATCTT-GGGCCGCACGC
GTGCTACAATGTAGGCGCTAAT-AGGC----ATTT-TACCC---TTTACCT-AGA---GG
TAGAGGTAACC-TA-CAATCCCCTACGTGATGGGGATTG-ACCACTGTAACTGT-GGTCA
TCAACGAGGAATTCCTTGTAGGCGTGCGTCACTATCGCACGCCGAATCTGTCCCTGCCCT
CTGTACACACCGCCCGTCGCTCCTACCGATCGAACGTCCAGGTAAAGTCGACGGACT---
--------------GTC-TTCG---AA--AGTTG----------AT-AGA--AAGT-CC-
A-T-TGAACCT--C----GTC--GTTT
>AM168070.1_Cavenderia_multistipes_UK26b
TCTAAG-TATA-G-TCCTC--ACGGACGAAA---CTGCAGACGGCTCATT--ACAA--CG
GTCGC-----TG---GTCACAGGACACGCGGCGGGGCA---ACTCGCTGT-GGATAACCG
CATTAAATCGGGGCTAATACATACAAAACCGAGTGGACGACTGGGC----AAC-TGGAAG
TTCGCGCGATGGA--TTAA-CCC-TTC--GA-CTGACCT--CT-T----GGTTTG--GTT
GGAACCGATTCATGTTGTTAGTCGGC-----GCTT-C--GGCGCC-GATTTGTTCTAGAC
GCGACTGCCCTATCAGCTT--GACGGCAAGGTCTTGGCTTGCCGTGGCTGTAACGGGTAA
CGGAGAATTAGGGTTCGATTCCGGAGAGGACGCCTGAGAAACGGCGTCCACACCTACGGG
TGGCAGCAGGCACGTAAATT-TCCCAATGTCAATAC-GATGAGGGAGCGACAATAAATAC
TTTTAGTCCTGGTCGCAAGATCAGGACTCAACAAGATTAAGCACAAAGTAAATAAATTAG
CC-AGTATTATTGGAGGGCAAGTCTGGTGCCAGCAGCCGCGGTAATTCCAGCTCCAA-CA
GTATATACTAAAGTTGTTGCAGTTAAAA-CGT-TCGTAGCT--------TA---------
-----A-------ATACAGGTCTGTG----TAA-TTGCTT-G----TCACGTCGTTCGC-
--GG--------CGT-GAC-AAGCGCAGGCGC-GGTAGATTGTCATGTCTAG-----TCG
GCAAC-GGCTA--GG-----------CGCGGCGTTCAACTGTGCATAAA-TCTTGATGCT
CAAGGTG--AGTCC---AGGCGT-GGACGGGAAAACAGT-GCATGGTATTGTAGAAGAAG
ACGTG-GCAACGTCTAGGTTGGT-TGGTT-------GTCCAGGTAATGATTAATAGGGAG
GAGCGGGGGCTGTCGTATTGACGGGCGAGAGGTGAAATTCGTTGACCCTGTCAAGACGCA
CAACGGCGGAAGCAGCAGCCAAGTGCCTCTCCATTAGTCAAGAACGATAGTCTAGGGATC
AAAGACGATCAGATACCGTCGTAGTCTAGACCATAAACTATGTCGGCTAACACTT-GGCG
ACGT-GC-TCTCCGAGGCGTCTCCAGGAGTTT-GTGGGAAACCAT-AAGCTTTTGGACTC
TGGGGGGAGTATGGCCGCAAGACTGAAACTTAAAGGAATTGACGGAAGGGCACACCATGG
CGTGGAG-CCTGCGGCTTAATTT-GACTCAACACGGGAAA-CCTTACCAAGCTCAGATAT
GTATAGGATTGACAGA-CTAA-TAGATTTTTCATGATCGCATAAGTAGTGGTGCATGGCC
GTTCTTAG-TTGGTGGAGTGATTTGTCAGGTCAATTCCGGTAACGGACGAGACCTCCATT
TGCTAACTAGTCGCGGCGACGGGTTCG--ACTGGCGAGGGTCGGACGGC-GCGGCTGCCT
G------GTCGAAAGGCTGGG--TAAGTGCGTCGTACGGTTAGTAG----CCTGGGCTCG
TCT--TCGCGGAATAAATAAAGT---AA-CTTCTTAGAAATACTTCCG-ACACCAAGCCG
GAGG-AAGTTGGAGGCAATAACAGGTCTGTGATGCCCTTAGATATCTT-GGGCCGCACGC
GTGCTACAATGTAGACGCGAATAAG-C---TCTAAAAATGC-GCGGCGCGA-AA----GC
GTCGCGGAAAC--AGCAATCGTCTACGTAACAGGGACTG-ACGGTTGCAATAAC-GATCA
CGAACGAGGAATGCCTTGTAGGCGCGAGTCATCATCTCGCGCCGAATCTGTCCCTGCCCT
TTGTACACACCGCCCGTCGCTCCTACCGATCGGACGGCGAGGTGAAGACGACGGACT---
--------------GTA-TCCTTC-ACG-GGAC-----------GC-GGA--AAGT-CG-
T-T-TAAACCT--T----GTT--GTCT
>AM168081.1_Cavenderia_stellata_SAB7B
TCTAAG-TATAAGCTCTTG-TACGGCTA-GA---CTGCAGACGGCTCATT--ACAA--CG

GTTGT-----AG---CTTCCAGGACATCCGG-GTCGCA---AGGCCTTCT-GGATAACCG
CAGTAAATCGGGGCTAATACATATAAA-CGGAGGGGTAGAGGGGGC----AACCTTGAAG
CTTCTGCGATGGACACTTA-GCTATTC--GA-CCAACCC--CG-CAA-GGGAGTG--GTT
GGAACCGGTTCATATTGCTAATCGACT-CTAGCTTGCTAGTAGTCTGATAAGTTCTATAG
ACAACTGCCCTATCAACTT--GATGGTAAGGTTTTGGCTTACCATGGTTATAACGGGTAA
CGGGGAATCAGGGTTCGATTCCGGAGAGGACGCCTGAGAAACGGCGTCCACATCTACGGG
TGGCAGCAGGCGCGTAAATT-GCCCAATCTCAACAGAGAGGAGGCGGTGACAATAAATCC
CGATGGCTTTGGGGGCAACCCCAGG--CCAATCAGAATAAGTACACATTAAATCCCCTAA
CC-AATATAATTGGAGGGCAAGTCTGGTGCCAGCAGCCGCGGTAATTCCAGCTCCAA-TA
GCATATACTAATGTTGTTGCAGTTAAAA-CGC-TCGTAGCT-----CAATATCTTTT--G
AG-CTAT------TTACGATCGTGT--CACTCACCTGATG-G----CCCTTAAAGCTTGA
G-GGTGAG----CGC-GGT-CGGCTCGAACAC-TGTAGGTCACGGTG-GTCT-----TAG
CAATA-GGGCC--AT-----------CGTAA---TCGACTGTGCATAAA-CCTTGATGCT
CAAGGTA--GG-CCT-CTGG----GGGTAGATACGCAGT-GCATGGCATTGTGGAACAAG
GCAT--CTCGCGGCTTAGTTGGT-GGGCC-------GCGGGGGCAATGATTAATAGGGAG
GAGCGGGGGCCTTCATATTGCAGGGCGAGAGGTGAAATTCGTTGACCCTTGCAAGATGTC
CTACAGCGAAAGCATTGGCCAAGTGCCTCTCCATTAGTCAAGAACGAAAGTTTGGGGATC
AAAGACGATCAGATACCGTCGTAGTCCAAACCATAAACTATGTCGACCAGTGATTAGGCG
GGCTACCTTCTTCGAGAGCTGCCTAGCAACTT-GTGGGAAACCAT-GAGTGCTTGGACTC
TGGGGGGAGTATGGTCGCAAGGCTGAAACTTAAAGGAATTGACGGAAGGGCACACCATGG
AGTGGAG-CCTGCGGCTTAATTT-GACTCAACACGGGAAA-GCTTACCAAGCTCAGATAT
GATAAGGATTGACAGA-CTAA-AAGATCTTTCATGATCTCATAAGTGGTGGTGCATGGTC
GTTCTTAG-TTGGTGGAGTGATTTGTCAGGTCAATTCCGGTAACGGACGAGACCTCGACC
TGCTAACTAGTGGGGCTCATTGGTTCA--AATGGCGAAGCGAATTTTGC-TTTTACTATG
G-----GTGGGCAACCAT---T-CATAGTGGGGGTAGGGAGCGTATGTAGCCTGGGCCAG
TGG--GTTTCAAAAATTAAAATT---AATCTTCCTAGAGGTACTTCTG-GCTCTAAGCCA
GAGG-AAGTCCGAGGCAATAACAGGTCTGTGATGCCCTTAGATATCTT-GGGCCGCACGC
GTGCTACAATGTAGGCGCTAATGAGTCG--TAACA-T-TCC--AGCACCGC-AAG---GT
GTCTGGTAATC-TTGGAATCCCCTGCGTGATTGGGCTTG-ACCACTGTAACTGT-GGTCA
TCAACGAGGAATTCCTTGTATGCGCGAGTCACTATCTCGCGCAGAATCTGTCCCTGCCCT
TTGTACACACCGCCCGTCGCTCCTACCGATCGAACGATCAGGTAAAGTGGACAGACA---
--------------GAGACCCG-C-AAG-GGCT-----------TT-TGA--AAGT-CC-
A-T-TGAACCT--C----GCC--GTTT
>KF662214.1_Cavenderia_aureostipes_var._helvetia_HM592

--TGT-----AT---CTTACAGGGCATCCGG-GTCGCA---AGACCTTCT-GGATAACCG
CAGTAAATCGGGGCTAATACATACAAA-CGGAGGAATGGAGGGGGC----AACCTTGAAG
TTTCTGCGATGGACATTTA-GCTATTC--GA-CCAACCC--CG-CAA-GGGAATG--GTT
GGAACCGGTTCATATTGCTAATCGACT-CTAGCTTGCTAGTAGTCTGATAAGCCCTATAG
ACAACCGCCCTATCAACTT--GATGGTAAGGTTTTGGCTTACCATGGTTGTAACGGGTAA
CGGGGAATCAGGGTTCGATTCCGGAGAGGACGCCTGAGAAACGGCGTCCACATCTACGGG
TGGCAGCAGGCGCGTAAATT-GCCCAATCTCAACAGAGAGGAGGCGGTGACAATAAATCC
CGATGGCTTTGGGGGCAACCCCAGG--CTAATCAGAATAAGTACACATTAAATCCCCTAA
CC-AATATAATTGGAGGGCAAGTCTGGTGCCAGCAGCCGCGGTAATTCCAGCTCCAA-TA
GCATATACTAATGTTGTTGCAGTTAAAA-CGC-TCGTAGCT-----GAATATCTTTT--G
AG-CTAC------TATCGGTTGTGT--T------CTATTG-A----CCCGCAAGG--GGA
--GGT-AG----CGC-AGC-CGGCTCAAACAC-TGTAGGTCATGGCG-TTGG-----TAG
TAATA-CTGAT--GT-----------CATAA---TCGACTGTGCATAAA-CCTTGATGCT
CAAGGTA--GG-CCT-TT---ATAGGGTAGATACACAGT-GCATGGCATTGTGGAACAAG
GCAT--CTCGCGGCTTAGTTGGT-GGGCC-------GCGGGGGCAATGATTAATAGGGAG
GAGCGGGGGCCTTCATATTGCAGGGCGAGAGGTGAAATTCGTTGACCCTTGCAAGATGTC
CTACAGCGAAAGCATTGGCCAAGTGCCTCCCCATTAGTCAAGAACGAAAGTTTGGGGATC
AAAGACGATCAGATACCGTCGTAGTCCAAACCATAAACTATGTCGACCAGCGATTAGGCG
GGCTACCTTCTTCGAGAGCTGCCTAGCAGCTT-GTGGGAAACCAT-GAGTGCTTGGACTC
TGGGGGGAGTATGGTCGCAAGGCTGAAACTTAAAGGAATTGACGGAAGGGCACACCATGG
AGTGGAG-CCTGCGGCTTAATTT-GACTCAACACGGGAAA-GCTTACCAAGCTCAGATAT
GATAAGGATTGACAGA-CTAA-AAGATCTTTCATGATCTCATAAGTGGTGGTGCATGGTC
GTTCTTAG-TTGGTGGAGTGATTTGTCAGGTCAATTCCGGTAACGGACGAGACCTCGACC
TGCTAACTAGTGGGATTCATTCCTTCG--CTCGACGAGGCAGGCTTTGC-TTTGATTATA
G------GGGGCAACCTT-----TATAGTCGGG-TAAGGTTTGTAT-TAGTCTGGGGGAG
TGG--GTTTCATATAATTTAATT---AATCTTCCTAGAGGTACTTCTG-GCTCTAAGCCA
GAGG-AAGTCCGAGGCAATAACAGGTCTGTGATGCCCTTAGATATCTT-GGGCCGCACGC
GTGCTACAATGTAGGCGCTAATGAGTCT--TTATT-TATCC--AGCTCCGT-AAG---GA
GTCTGGTAATC-TTGGAATCCCCTGCGTGATTGGGCTTG-ACCACTGTAACTGT-GGTCA
TCAACGAGGAATTCCTTGTATGCGCGAGTCACTATCTCGCGCAGAATCTGTCCCTGCCCT
TTGTACACACCGCCCGTCGCTCCTACCGATCGAACGATCAGGTAAAGTGGACAGACT---
--------------TCGACTCG-C-AAG-AGTT-----------T---------------

>AM168084.1_Cavenderia_bifurcata_UK5
TCTAAG-TATAAGCTCTTG-TACGGCTA-GA---CTGCAGACGGCTCATT--ACAA--CG
GTTGT-----AG---CTTCCAGGACATCCGG-GTCGCA---AGACCTTCT-GGATAACCG
CAGTAAATCGGGGCTAATACATACAAA-CGGAGGGATGGATAGGGC----AACCTTGAAG
TTTCTGCGATGGACACTTA-GCTATTC--GA-CCAGCCC--TGGCAACAGGAATG--GTT

GGAACCGGTTCATATTGCTAATCGACT-CTAGCTTGCTAGCAGTCTGATAAGTCCTATAG
ACAACCGCCCTATCAACTT--GATGGTAAGGTTTTGGCTTACCATGGTTGTAACGGGTAA
CGGGGAATCAGGGTTCGATTCCGGAGAGGACGCCTGAGAAACGGCGTCCACATCTACGGG
TGGCAGCAGGCGCGTAAATT-GCCCAATCTCAACAGAGAGGAGGCGGTGACAATAAATCC
CGATGGCTTTGGGGGCAACCCCAGG--CCAATCAGAATAAGTACACATTAAATCCCTTAA
CC-AATATAATTGGAGGGCAAGTCTGGTGCCAGCAGCCGCGGTAATTCCAGCTCCAA-TA
GCATATACTAATGTTGTTGCAGTTAAAA-CGC-TCGTAGCT-----CAATTACTTTT--G
AG-CTTA------TTACGGTTGTTCATTAGTTTGCTTAAA-G----GCCTCACGGCTAAT
A-GGTAAATTGATAC-GAC-CGGCTCAAACAC-TGTAGGTCATGGCA-TTAG----GGAG
CAATTTCTAGT--GT-----------CATAA---TCGACTGTGCATAAA-CCTTGATGCT
CAAGGTA--GG-CCT-TT--TATAGGGCAGATACACAGT-GCATGGCATTGTGGAACAAG
GCAT--CTCGCGGCTTAGTTGGT-GGGCC-------GCGGGGGCAATGATTAATAGGGAG
GAGCGGGGGCCTTCATATTGCAGGGCGAGAGGTGAAATTCGTTGACCCTTGCAAGATGTC
CGACAGCGAAAGCATTGGCCAAGTGCCTCTCCATTAGTCAAGAACGAAAGTTTGGGGATC
AAAGACGATCAGATACCGTCGTAGTCCAAACCATAAACTATGTCGACCAGCGATTAGGCG
CGCTACCTTCTTCGAGAGCTGCCTAGCAGCTT-GTGGGAAACCAT-GAGTGCTTGGACTC
TGGGGGGAGTATGGTCGCAAGTCTGAAACTTAAAGGAATTGACGGAAGGGCACACCATGG
AGTGGAG-CCTGCGGCTTAATTT-GACTCAACACGGGAAA-GCTTACCAAGCTCAGATAT
GATAAGGATTGACAGA-CTAA-AAGATCTTTCATGATCTCATAAGTGGTGGTGCATGGTC
GTTCTTAG-TTGGTGGAGTGATTTGTCAGGTCAATTCCGGTAACGGACGAGACCTCGACC
TGCTAACTAGTGGGGTTCATTCTTTCG--CTTGACGAGGCAGGTTTTGCCTTTGATTACA
GAAG--GGGGGCAACTTTCTTT-TGTAGTTGGGGTAGAGTTTGTAG-TAGTCTGGAGGAG
TGG--ACTTCAAAAATTAAAAAT---AATCTTCCTAGAGGTACTTCTG-GCTCTAAGTCA
GAGG-AAGTCCGAGGCAATAACAGGTCTGTGATGCCCTTAAATATCTT-GGGCCGCACGC
GTGCTACAATGTAGGCGCTAGAAAGTCA--TAATTAAAACC--ATCTCCGC-AAG---GA
GTATGGTAATC-TTATAATCACCTGCGTGATTGGGCTTG-ACCACTGTAACTGT-GGTCA
TCAACGAGGAATTCCTTGTATGCGCGAGTCACTATCTCGCGCAGAATCTGTCCCTGCCCT
TTGTACACACCGCCCGTCGCTCCTACCGATCGAACGATCAGGTAAAGTGGACAGACA---
----C---------GAAGCTCG-C-AAG-AGCT-----------TT-TGG--AAGT-CC-
A-T-TGAACCT--C----GCC--GTTT
>AM168089.1_Cavenderia_mexicana_MexTF4B1
TCTAAG-TATAAGCTCTTG-TACGGCTA-GA---CTGCAGACGGCTCATT--ACAA--CG
GTTGT-----AT---CTTCCAGGACATCCGG-GTCGAA---AGACCTTCT-GGATAACCG
CAGTAAATCGGGGCTAATACATACAAA-CGGAGGGGTGGATAGGGC----AACCTTGAAG
CTTCTGCGATGGACACTTA-GCTATTC--GA-CCAGCCC--CG-CAA-GGGAACG--GTT
GGAACCGGTTCATATTGCTAATCGACT-CTGGCTTGCCACGAGTCTGATAAGTCCTATAG
ACAACCGCCCTATCAACTT--GATGGTAAGGTTTTGGCTTACCATGGTTGTAACGGGTAA
CGGGGAATCAGGGTTCGATTCCGGAGAGGACGCCTGAGAAACGGCGTCCACATCTACGGG
TGGCAGCAGGCGCGTAAATT-GCCCAATCTCAACAGAGAGGAGGCGGTGACAATAAATCC
CGATGGCTTTGGGGGCAACCCCAGG--CCAATCAGAATAAGTACACATTAAATCCCTTAA
CC-AATATAATTGGAGGGCAAGTCTGGTGCCAGCAGCCGCGGTAATTCCAGCTCCAA-TA
GCATATACTAATGTTGTCGCAGTTAAAAACGCCTCGTAGCT-----TAATTACTTTTT-G
AG-CTAC------TTACGGTTGTCCA-AC-TTTGCG-AAA-G-----ACTTAACTTTTCCT
A-GTGAAG----AAC-AAC-CGGCTCAGACAC-TGTAGGTCGTGGCA-TTGG----GG-G
TAACT-CTAGT--GT-----------CACTA---TCGACTGTGCATAAA-CCTTGATGCT
CAAGGTA--GG-CCT-TT---ATAGGGTAGATACACAGT-GCATGGCATTGTGGAACAAG
GCAT--CTCGCGGCTTAGTTGGT-GGGCC-------GCGGGGGCAATGATTAATAGGGAG
GAGCGGGGACCTTCATATTGCAGGGCGAGAGGTGAAATTCGTTGACCCTTGCAAGATGAC
CGACAGCGAAAGCATTGGTCAAGTGCCTTCCCATTAGTCAAGAACGAAAGTTTGGGGATC
AAAGACGATCAGATACCGTCGTAGTCCAAACCATAAACTATGTCGACCAGCGATTAGGCG
GGCTACCTTCTTCGAGAGCTGCCTAGCAGCTT-GTGGGAAACCAT-GAGTGCTTGGACTC
TGGGGGGAGTATGGTCGCAAGGCTGAAACTTAAAGGAATTGACGGAAGGGCACACCATGG
AGTGGAG-CCTGCGGCTTAATTT-GACTCAACACGGGAAA-GCTTACCAAGCTCAGATAT
GATAAGGATTGACAGA-CTAA-AAGATCTTTCATGATCTCATAAGTGGTGGTGCATGGTC
GTTCTTAG-TTGGTGGAGTGATTTGTCAGGTCAATTCCGGTAACGGACGAGACCTCGACC
TGCTAACTAGTGGGGTTCATTCCTTCG--CTCGATGAGGCAAGGTCTGC-TTTTATTGCA
G------GGGGTAACTCT-----TGTAGTAGGG-TAGGCTTTGTAT-TAGTCTGGGGGAG
TGG--ACTTC-CA--ATATAATTTATAATCTTCCTAGAGGTACTTCTG-GCTCTAAGCCA
GAGG-AAGTCCGAGGCAATAACAGGTCTGTGATGCCCTTAGATATCTT-GGGCCGCACGC
GTGCTACAATGTAGGCGCTAATGAGTC---GTAACATATCC--AGCTCCGT-AAG---GA
GTCTGGTAATC-TTGGAATCCCCTGCGTGATTGGGCTTG-ACTACTGTAACTGT-GGTCA
TCAACGAGGAATTCCTTGTATGCGTGAGTCACTATCTCACGCAGAATCTGTCCCTGCCCT
TTGTACACACCGCCCGTCGCTCCTACCGATCGAACGATCAGGTAAAGTGGACAGACT---
--------------TTAGCCCG-C-AAG-GGTT-----------TT-GGG--AAGT-CC-
A-T-TGAACCT--C----GCC--GTTT
>AM168072.1_Cavenderia_granulophora_CHII_4
TCTAAG-TATAAGCTCTTG-TACGGCTA-GA---CTGCAGACGGCTCATT--ACAA--CG
GTTGT-----AT---CTTCCAGGACATCCGG-GTCGCA---AGATCTTCT-GGATAACCG
CAGTAAATCGGGGCTAATACATACAAA-CGAAGGGGCAGAGAGGGC----AACCTTGAAG
CTTCTGCGATGGACACTTA-GCTATTC--GA-CCAACCC--CG-CAA-GGGAATG--GTT
GGAACCGGTTCATATTGCTAATCGACT-CTGGTTTACCATGAGTC-GATAAGTCCTATAG
ACAACCGCCCTATCAACTT--GATGGTAAGGTTTTGGCTTACCATGGTTGTAACGGGTAA
CGGGGAATCAGGGTTCGATTCCGGAGAGGACGCCTGAGAAACGGCGTCCACATCTACGGG

TGGCAGCAGGCGCGTAAATT-GCCCAATCTCAACAGAGAGGAGGCGGTGACAATAAATCC
CGATGGCTTTGGGGGCAACCCCAGG--CCAATCAGAATAAGTACACATTAAATCCCTTAA
CC-AATATAATTGGAGGGCAAGTCTGGTGCCAGCAGCCGCGGTAATTCCAGCTCCAA-TA
GCATATACTAATGTTGTTGCAGTTAAAA-CGC-TCGTAGCT-----TAATTTCTTTT--G
AG-CTAC------TTACGGTTGTC---ACC-CTGTG--AA-G----TCTTCGGACCTAGT
A-GG--------AAC-AGC-CGGCTCAAACAC-TGTAGGTCATGGT-----GC----TAG
CAATA-GC-----AT-----------CATAA---TCGACTGTGCATAAA-CCTTGATGCT
CAAGGTA--GG-CCT-TT---TTAGGGTAGATACACAGT-GCATGGCATTGTGGAACAAG
GCAT--CTCGCGGCTTAGTTGGT-GGGCC-------GCGGGGGCAATGATTAATAGGGAG
GAGCGGGGGCCTTCATATTGCAGGGCGAGAGGTGAAATTCGTTGACCCTTGCAAGATGTC
CGACAGCGAAAGCATTGGCCAAGTGCCTCCCCATTAGTCAAGAACGAAAGTTTGGGGATC
AAAGACGATCAGATACCGTCGTAGTCCAGACCATAAACTATGTCGACCAGCGATTAGGCG
GGCTACCTTCTTCGAGAGCTGCCTAGCAGCTT-GTGGGAAACCAT-GAGTGCTTGGACTC
TGGGGGGAGTATGGTCGCAAGGCTGAAACTTAAAGGAATTGACGGAAGGGCACACCATGG
AGTGGAG-CCTGCGGCTTAATTT-GACTCAACACGGGAAA-GCTTACCAAGCTCAGATAT
GATAAGGATTGACAGA-CTAA-AAGATCTTTCATGATCTCATAAGTGGTGGTGCATGGTC
GTTCTTAG-TTGGTGGAGTGATTTGTCAGGTCAATTCCGGTAACGGACGAGACCTCGACC
TGCTAACTAGTGGGATTCATTCCTTCT--CTCGACGAGGCAGGTTCTGC-TTTTATTGTA
A------GGG-CAACT-T-----TGCAGTAGGG-TAGGATTTGTAT-TAGTCTGGGGGAG
TGG--ATTTCATA--ATTTAATT---AATCTTCCTAGAGGTACTTCTG-GCTCTAAGCCA
GAGG-AAGTTCGAGGCAATAACAGGTCTGTGATGCCCTTAGATATCTT-GGGCCGCACGC
GTGCTACAATGTAGGCGCTAATGAGTC---TTTTTACATCC--AGCTCCGT-AAG---GA
GTCTGGTAATC-TTGGAATCCCCTGCGTGATTGGGCTTG-ACTACTGTAACTGT-GGTCA
TCAACGAGGAATTCCTTGTATGCGCGAGTCACTATCTCGCGCAGAATCTGTCCCTGCCCT
TTGTACACACCGCCCGTCGCTCCTACCGATCGAACGATCAGGTAAAGTGGACAGACT---
--------------AGAGGCCG-C-AAG-GCAT-----------TT-GGA--AAGT-CC-
A-T-TGAACCT--C----GCC--GTTT
>AM168088.1_Cavenderia_medusoides_OH592
TCTAAG-TATAAGCTCTTG-TACGGCTA-GA---CTGCAGACGGCTCATT--ACAA--CG
GTTGT-----AT---CTTCCAGAACGTCCGG-GTCGCA---AGATCTTCT-GGATAACCG
CAGTAAATCGGGGCTAATACATACAAA-CGAAGGGGTGGAGAGGGC----AACCTTGAAG
CTTCTGCGATGGACACTTA-GCTATTC--GA-CCAACCC--CG-CAA-GGGAATG--GTT
GGAACCGGTTCATATTGCTAATCGACT-CTGGTTTACCATGAGTC-GATAAGTTCTATAG
ACAACCGCCCTATCAACTT--GATGGTAAGGTTTTGGCTTACCATGGTTGTAACGGGTAA
CGGGGAATCAGGGTTCGATTCCGGAGAGGACGCCTGAGAAACGGCGTCCACATCTACGGG
TGGCAGCAGGCGCGTAAATT-GCCCAATCTCAACAGAGAGGAGGCGGTGACAATAAATCC
CGATGGCTTTGGGGGCAACCCCAGG--CCAATCAGAATAAGTACACATTAAATCCCTTAA
CC-AATATAATTGGAGGGCAAGTCTGGTGCCAGCAGCCGCGGTAATTCCAGCTCCAA-TA
GCATATACTAATGTTGTTGCAGTTAAAA-CGC-TCGTAGCT-----TAATTTCTTTT--G
AG-CTAC------TTACGGTTGTC---ACC-CTGTG--AA-G----TCTTCGGACCTAAT
A-GG--------AAC-AGC-CGGCTCAAACAC-TGTAGGTCATGGTA-CTGG-----TA-
------ACAGT--AT----------CATAA---TCGACTGTGCATAAA-CCTTGATGCT
CAAGGTA--GG-CCT-TT---TTAGGGTAGATACACAGT-GCATGGCATTGTGGAACAAG
GCAT--CTCGCGGCTTAGTTGGT-GGGCC-------GCGGGGGCAATGATTAATAGGGAG
GAGCGGGGGCCTTCATATTGCAGGGCGAGAGGTGAAATTCGTTGACCCTTGCAAGATGTC
CGACAGCGAAAGCATTGGCCAAGTGCCTCCCCATTAGTCAAGAACGAAAGTTTGGGGATC
AAAGACGATCAGATACCGTCGTAGTCCAAACCATAAACTATGTCGACCAGCGATTAGGCG
GGCTACCTTCTTCGAGAGCTGCCTAGCAGCTT-GTGGGAAACCAT-GAGTGCTTGGACTC
TGGGGGGAGTATGGTCGCAAGGCTGAAACTTAAAGGAATTGACGGAAGGGCACACCATGG
AGTGGAG-CCTGCGGCTTAATTT-GACTCAACACGGGAAA-GCTTACCAAGCTCAGATAT
GATAAGGATTGACAGA-CTAA-AAGATCTTTCATGATCTCATAAGTGGTGGTGCATGGTC
GTTCTTAG-TTGGTGGAGTGATTTGTCAGGTCAATTCCGGTAACGGACGAGACCTCGACC
TGCTAACTAGTGGGATTCATTCCTTCG--CTCGACGAGGCAGGTTCTGC-TTTTATTGTA
G------GGG-CAACT-T-----TACAGTAGGG-TAGGATTTGTAT-TAGTCTGGGGGAG
TGG--ATTTCATA--ATTTAATT---AATCTTCCTAAAGGTACTTCTG-GCTCTAAGCCA
GAGG-AAGTTCGAGGCAATAACAGGTCTGTGATGCCCTTAGATATCTT-GGGCCGCACGC
GTGCTACAATGTAGGCGCTAATGAGTC---TTTTTACATCC--AGCTCCGT-AAG---GA
GTCTGGTAATC-TTGGAATCCCCTGCGTGATTGGGCTTG-ACTACTGTAACTGT-GGTCA
TCAACGAGGAATTCCTTGTATGCGCGAGTCACTATCTCGCGCAGAATCTGTCCCTGCCCT
TTGTACACACCGCCCGTCGCTCCTACCGATCGAACGATCAGGTAAAGTGGACAGACT---
--------------AGAAGCCG-C-AAG-GTTT-----------TT-GGA--AAGT-CC-
A-T-TGAACCT--C----GCC--GTTT
>AM168083.1_Cavenderia_aureostipes_YA6
TCTAAG-TATAAGCTCTTG-TACGGCTA-GA---CTGCAGACGGCTCATT--ACAA--CG
GTTGT-----AT---CTTCCAGGACATCCGG-GTCGCA---AGACCTTCT-GGATAACCG
CAGTAAATCGGGGCTAATACATACAAA-CGGAGGGGTAGAGAGGGC----AACCTTGAAG
CTTCTGCGATGGACAATTA-GTTATTC--GA-CCAACCC--CG-CAA-GGGAATG--GTT
GGAACCGGTTCATATTGCTAATCGACT-CTAGCTTGCTAGTAGTCTGATAAGTCCTATAG
ACAACCGCCCTATCAACTT--GATGGTAAGGTTTTGGCTTACCATGGTTGTAACGGGTAA
CGGGGAATCAGGGTTCGATTCCGGAGAGGACGCCTGAGAAACGGCGTCCACATCTACGGG
TGGCAGCAGGCGCGTAAATT-GCCCAATCTCAACAGAGAGGAGGCGGCGACAATAAATCC
CGATGGCTTTGGGGGCAACCCCAGG--CCAATCAGAATAAGTACACATTAAATCCCTTAA
CC-AATATAATTGGAGGGCAAGTCTGGTGCCAGCAGCCGCGGTAATTCCAGCTCCAA-TA

GCATATACTAATGTTGTTGCAGTTAAAA-CGC-TCGTAGCT-----CAATATCTTTT--G
AG-C-GC------TTTCGGCCGTT---CTCTTTAGC--TA------CCGCA--AGGCTGT
TAAAGA------ACC-GGT-CGGCTCAAACAC-TGTAGGTCATGGTA-AAGT-----TAG
CAATA-GCTTT--AT-----------CATAA---TCGACTGTGCATAAA-CCTTGATGCT
CAAGGTA--GG-CCT-TT--TATAGGGTAGATACACAGT-GCATGGCATTGTGGAACAAG
GCAT--CTCGCGGCTTAGTTGGT-GGGCC-------GCGGGGGCAATGATTAATAGGGAG
GAGCGGGGGCCTTCATATTGCAGGGCGAGAGGTGAAATTCGTTGACCCTTGCAAGATGTC
CTACAGCGAAAGCATTGGCCAAGTGCCTCCCCATTAGTCAAGAACGAAAGTTTGGGGATC
AAAGACGATCAGATACCGTCGTAGTCCAAACCATAAACTATGTCGACCAGCGATTAGGCG
GGCTACCTTCTTCGAGAGCTGCCTAGCAGCTT-GTGGGAAACCAT-GAGTGCTTGGACTC
TGGGGGGAGTATGGTCGCAAGGCTGAAACTTAAAGGAATTGACGGAAGGGCACACCATGG
AGTGGAG-CCTGCGGCTTAATTT-GACTCAACACGGGAAA-GCTTACCAAGCTCAGATAT
GATTAGGATTGACAGA-CTAA-AAGATCTTTCATGATCTCATAAGTGGTGGTGCATGGTC
GTTCTTAG-TTGGTGGAGTGATTTGTCAGGTCAATTCCGGTAACGGACGAGACCTCGACC
TGCTAACTAGTGGGATTCATTCTTTCG--ATTGACGAGGCAGGTTTTGC-TTTGATTATA
G------GGGGCAACTTC-----TATAGTCGGG-TAGAGCTTGTAT-TAGTCTGGGAGAG
TGG--GTTTCAAAAATTAAAATT---AATCTTCCTAGAGGTACTTCTG-GCTCTAAGCCA
GAGG-AAGTCCGAGGCAATAACAGGTCTGTGATGCCCTTAGATATCTT-GGGCCGCACGC
GTGCTACAATGTAGGCGCTAATGAGTT---TATTTACATCC--ATCTCCGC-AAG---GA
GTATGGTAATC-TTGGAATCCCCTGCGTGATTGGGCTTG-ACCACTGTAACTGT-GGTCA
TCAACGAGGAATTCCTTGTATGCGCGAGTCACTATCTCGCGCAGAATCTGTCCCTGCCCT
TTGTACACACCGCCCGTCGCTCCTACCGATCGAACGATCAGGTAAAGTGGACAGACT---
--------------GGAACTCG-C-AAG-AGTT-----------CT-TGG--AAGT-CC-
A-T-TGAACCT--C----GCC--GTTT
>KF662201.1_Cavenderia_aureostipes_OH396

--TGT-----AT---CTTCCAGGGCATCCGG-GTCGCA---AGACCTTCT-GGATAACCG
CAGTAAATCGGGGCTAATACATATAAA-CGGAGGGATGGAGAGGGC----AACCTTGAAG
TTTCTGCGATGGACAATTA-GTTATTC--GA-CCAACCC--CG-CAA-GGGAATG--GTT
GGAACCGGTTCATATTGCTAATCGACT-CTAGCTTGCTAGTAGTCTGATAAGCCCTATAG
ACAACCGCCCTATCAACTT--GATGGTAAGGTTTTGGCTTACCATGGTTGTAACGGGTAA
CGGGGAATCAGGGTTCGATTCCGGAGAGGACGCCTGAGAAACGGCGTCCACATCTACGGG
TGGCAGCAGGCGCGTAAATT-GCCCAATCTCAACAGAGAGGAGGCGGTGACAATAAATCC
CGATGGCTTTGGGGGCAACCCCAGG--CTAATCAGAATAAGTACACATTAAATCCCTTAA
CC-AATATAATTGGAGGGCAAGTCTGGTGCCAGCAGCCGCGGTAATTCCAGCTCCAA-TA
GCATATACTAATGTTGTTGCAGTTAAAA-CGC-TCGTAGCT-----GAATATCTTTT--G
AG-CTAC------TATCGACTGTT---TTCCTTCTG--GA------TCTTC--GGAAAAG
GGAGG-------GGC-AGT-CGGCTCAAACAC-TGTAGGTCATG-----CGGC----TAG
CAATA-GTTG----------------CATAA---TCGACTGTGCATAAA-CCTTGATGCT
CAAGGTA--GG-CCT-TT---ATAGGGTAGATACACAGT-GCATGGCATTGTGGAACAAG
GCAT--CTCGCGGCTTAGTTGGT-GGGCC-------GCGGGGGCAATGATTAATAGGGAG
GAGCGGGGGCCTTCATATTGCAGGGCGAGAGGTGAAATTCGTTGACCCTTGCAAGATGTC
CTACAGCGAAAGCATTGGCCAAGTGCCTCCCCATTAGTCAAGAACGAAAGTTTGGGGATC
AAAGACGATCAGATACCGTCGTAGTCCAAACCATAAACTATGTCGACCAGCGATTAGGCG
GGCTACCTTCTTCGAGAGCTGCCTAGCAGCTT-GTGGGAAACCAT-GAGTGCTTGGACTC
TGGGGGGAGTATGGTCGCAAGGCTGAAACTTAAAGGAATTGACGGAAGGGCACACCATGG
AGTGGAG-CCTGCGGCTTAATTT-GACTCAACACGGGAAA-GCTTACCAAGCTCAGATAT
GATTAGGATTGACAGA-CTAA-AAGATCTTTCATGATCTCATAAGTGGTGGTGCATGGTC
GTTCTTAG-TTGGTGGAGTGATTTGTCAGGTCAATTCCGGTAACGGACGAGACCTCGACC
TGCTAACTAGTGGGATTCATTCTTTCG--ATTGACGAGGCAGGTCTTGC-TTTGATTATA
G------GGGGCAACTTC-----TATAGTCGGG-TAGGGTTTGTAG-TAGTCTGGAGGAG
TGG--ATTTCAAA-AATTAAATT---AATCTTCCTAGAGGTACTTCTG-GCTCTAAGCCA
GAGG-AAGTCCGAGGCAATAACAGGTCTGTGATGCCCTTAGATATCTT-GGGCCGCACGC
GTGCTACAATGTAGGCGCTAATGAGTT---TATTTACATCC--ATCTCCGC-AAG---GA
GTATGGTAATC-TTGGAATCACCTGCGTGATTGGGCTTG-ACCACTGTAACTGT-GGTCA
TCAACGAGGAATTCCTTGTATGCGCGAGTCACTATCTCGCGCAGAATCTGTCCCTGCCCT
TTGTACACACCGCCCGTCGCTCCTACCGATCGAACGATCAGGTAAAGTGGACAGACT---
--------------GGAACCCG-C-AAG-GGTA-----------CT-TG-----------

>KF662199.1_Cavenderia_aureostipes_B15A
TCTAAG-TATAAGCTCTTG-TACGGCTA-GA---CTGCAGACGGCTCATT--ACAA--CG
GTTGT-----AT---CTTCCAGGGCATCCGG-GTCGCA---AGACCTTCT-GGATAACCG
CAGTAAATCGGGGCTAATACATACAAA-CGGAGGGATGGAGAGGGC----AACCTTGAAG
TTTCTGCGATGGACAATTA-GTTATTC--GA-CCAACCC--CG-CAA-GGGAACG--GTT
GGAACCGGTTCATATTGCTAATCGACT-CTAGCTTGCTAGTAGTCTGATAAGCCCTATAG
ACAACCGCCCTATCAACTT--GATGGTAAGGTTTTGGCTTACCATGGTTGTAACGGGTAA
CGGGGAATCAGGGTTCGATTCCGGAGAGGACGCCTGAGAAACGGCGTCCACATCTACGGG
TGGCAGCAGGCGCGTAAATT-GCCCAATCTCAACAGAGAGGAGGCGGTGACAATAAATCC
CGATGGCTTTGGGGGCAACCCCAGG--CTAATCAGAATAAGTACACATTAAATCCCTTAA
CC-AATATAATTGGAGGGCAAGTCTGGTGCCAGCAGCCGCGGTAATTCCAGCTCCAA-TA
GCATATACTAATGTTGTTGCAGTTAAAA-CGC-TCGTAGCT-----TAATATCTTTT--G
AG-CTAC------TATCGATTGTT---ATTCCTTTG--GA------TCCTCACGGTGAAG
GAGGGA------GGC-AAT-CGGCTCAAACAC-TGTAGGTCATG-----CGGC----TAG

CAATA-GTTG----------------CATAA---TCGACTGTGCATAAA-CCTTGATGCT
CAAGGTA--GG-CCT-TT---ATAGGGTAGATACACAGT-GCATGGCATTGTGGAACAAG
GCAT--CTCGCGGCTTAGTTGGT-GGGCC-------GCGGGGGCAATGATTAATAGGGAG
GAGCGGGGGCCTTCATATTGCAGGGCGAGAGGTGAAATTCGTTGACCCTTGCAAGATGTC
CTACAGCGAAAGCATTGGCCAAGTGCCTCCCCATTAGTCAAGAACGAAAGTTTGGGGATC
AAAGACGATCAGATACCGTCGTAGTCCAAACCATAAACTATGTCGACCAGCGATTAGGCG
GGCTACCTTCTTCGAGAGCTGCCTAGCAGCTT-GTGGGAAACCAT-GAGTGCTTGGACTC
TGGGGGGAGTATGGTCGCAAGGCTGAAACTTAAAGGAATTGACGGAAGGGCACACCATGG
AGTGGAG-CCTGCGGCTTAATTT-GACTCAACACGGGAAA-GCTTACCAAGCTCAGATAT
GATTAGGATTGACAGA-CTAA-AAGATCTTTCATGATCTCATAAGTGGTGGTGCATGGTC
GTTCTTAG-TTGGTGGAGTGATTTGTCAGGTCAATTCCGGTAACGGACGAGACCTCGACC
TGCTAACTAGTGGGATTCATTCTTTCG--ATTGACGAGGCAGGTCTTGC-TTTGATTATA
G------GGGGCAACTTT-----TATAGTCGGG-TACGGTTTGTAG-TAGTCTGGAGGAG
TGG--ATTTCAAA-AATTAAATT---AATCTTCCTAGAGGTACTTCTG-GCTCTAAGCCA
GAGG-AAGTCCGAGGCAATAACAGGTCTGTGATGCCCTTAGATATCTT-GGGCCGCACGC
GTGCTACAATGTAGGCGCTAATGAGTT---TATTTACATCC--ATCTCCGC-AAG---GA
GTATGGTAATC-TTGGAATCACCTGCGTGATTGGGCTTG-ACCACTGTAACTGT-GGTCA
TCAACGAGGAATTCCTTGTATGCGCGAGTCACTATCTCGCGCAGAATCTGTCCCTGCCCT
TTGTACACACCGCCCGTCGCTCCTACCGATCGAACGATCAGGTAAAGTGGACAGACT---
--------------GGAGCCCG-C-AAG-GGCA-----------CT-TGG--AAGT-CC-
A-T-TGAACCT--C----GCC--GTT-
>HQ141515.1_Cavenderia_subdiscoidea_TH1A
TCTAAG-TATAAGCTCTTG-TACGGCTA-GA---CTGCAGACGGCTCATT--ACAA--CG
GTTGT-----AT---CTTCCAGGGCATCCGG-GTCGCA---AGACCTTCT-GGATAACCG
CAGTAAATCGGGGCTAATACATACAAA-CGGAGGGATGGAGAGGGC----AACCTTGAAG
TTTCTGCGATGGACAATTA-GTTATTC--GA-CCAACCC--CG-CAA-GGGAATG--GTT
GGAACCGGTTCATATTGCTAATCGACT-CTAGCTTGCTAGTAGTCTGATAAGCCCTATAG
ACAACCGCCCTATCAACTT--GATGGTAAGGTTTTGGCTTACCATGGTTGTAACGGGTAA
CGGGGAATCAGGGTTCGATTCCGGAGAGGACGCCTGAGAAACGGCGTCCACATCTACGGG
TGGCAGCAGGCGCGTAAATT-GCCCAATCTCAACAGAGAGGAGGCGGTGACAATAAATCC
CGATGGCTTTGGGGGCAACCCCAGG--CTAATCAGAATAAGTACACATTAAATCCCTTAA
CC-AATATAATTGGAGGGCAAGTCTGGTGCCAGCAGCCGCGGTAATTCCAGCTCCAA-TA
GCATATACTAATGTTGTTGCAGTTAAAA-CGC-TCGTAGCT-----TAATATCTTTT--G
AG-CTAC------TATCGACTGTT---TTCCTCCTG--GA------TCTTC--GGAAAAG
GGAGG-------GGC-AGT-CGGCTCAAACAC-TGTAGGTCATG-----CGGC----TAG
CAATA-GTTG----------------CATAA---TCGACTGTGCATAAA-CCTTGATGCT
CAAGGTA--GG-CCT-TT---ATAGGGTAGATACACAGT-GCATGGCATTGTGGAACAAG
GCAT--CTCGCGGCTTAGTTGGT-GGGCC-------GCGGGGGCAATGATTAATAGGGAG
GAGCGGGGGCCTTCATATTGCAGGGCGAGAGGTGAAATTCGTTGACCCTTGCAAGATGTC
CTACAGCGAAAGCATTGGCCAAGTGCCTCCCCATTAGTCAAGAACGAAAGTTTGGGGATC
AAAGACGATCAGATACCGTCGTAGTCCAAACCATAAACTATGTCGACCAGCGATTAGGCG
GGCTACCTTCTTCGAGAGCTGCCTAGCAGCTT-GTGGGAAACCAT-GAGTGCTTGGACTC
TGGGGGGAGTATGGTCGCAAGGCTGAAACTTAAAGGAATTGACGGAAGGGCACACCATGG
AGTGGAG-CCTGCGGCTTAATTT-GACTCAACACGGGAAA-GCTTACCAAGCTCAGATAT
GATTAGGATTGACAGA-CTAA-AAGATCTTTCATGATCTCATAAGTGGTGGTGCATGGTC
GTTCTTAG-TTGGTGGAGTGATTTGTCAGGTCAATTCCGGTAACGGACGAGACCTCGACC
TGCTAACTAGTGGGATTCATTCTTTCG--ATTGACGAGGCAGGTCTTGC-TTTGATTATA
G------GGGGCAACTTC-----TATAGTCGGG-TAGGTTTTGTAG-TAGTCTGGAGGAG
TGG--ATTTCAAA-AATTAAATT---AATCTTCCTAGAGGTACTTCTG-GCTCTAAGCCA
GAGG-AAGTCCGAGGCAATAACAGGTCTGTGATGCCCTTAGATATCTT-GGGCCGCACGC
GTGCTACAATGTAGGCGCTAATGAGTT---TATTTACATCC--ATCTCCGC-AAG---GA
GTATGGTAATC-TTGGAATCACCTGCGTGATTGGGCTTG-ACCACTGTAACTGT-GGTCA
TCAACGAGGAATTCCTTGTATGCGCGAGTCACTATCTCGCGCAGAATCTGTCCCTGCCCT
TTGTACACACCGCCCGTCGCTCCTACCGATCGAACGATCAGGTAAAGTGGACAGACT---
--------------GGAACCCG-C-AAG-GGTA-----------CT-TGG--AAGT-CC-
A-T-TGAACCT--C----GCC--GTT-
>HQ141518.1_Cavenderia_pseudoaureostipes_TH39A
TCTAAG-TATAAGCTCTTG-TACGGCTA-GA---CTGCAGACGGCTCATT--ACAA--CG
GTTGT-----AT---CTTCCAGGGCATCCGG-GTCGCA---AGACCTTCT-GGATAACCG
CAGTAAATCGGGGCTAATACATACAAA-CGGAGGGATGGAGAGGGC----AACCTTGAAG
TTTCTGCGATGGACAATTA-GTTATTC--GA-CCAACCC--CG-CAA-GGGAATG--GTT
GGAACCGGTTCATATTGCTAATCGACT-CTAGCTTGCTAGTAGTCTGATAAGCCCTATAG
ACAACCGCCCTATCAACTT--GATGGTAAGGTTTTGGCTTACCATGGTTGTAACGGGTAA
CGGGGAATCAGGGTTCGATTCCGGAGAGGACGCCTGAGAAACGGCGTCCACATCTACGGG
TGGCAGCAGGCGCGTAAATT-GCCCAATCTCAACAGAGAGGAGGCGGTGACAATAAATCC
CGATGGCTTTGGGGGCAACCCCAGG--CTAATCAGAATAAGTACACATTAAATCCCTTAA
CC-AATATAATTGGAGGGCAAGTCTGGTGCCAGCAGCCGCGGTAATTCCAGCTCCAA-TA
GCATATACTAATGTTGTTGCAGTTAAAA-CGC-TCGTAGCT-----TAATATCTTTT--G
AG-CTAC------TATCGACTGTT---ATTTCTGTG--GA------TCCGCAAGGTGAAT
CAGGGA------GGC-AGT-CGGCTCAAACAC-TGTAGGTCATG-----CGGC----TAG
CAATA-GTTG----------------CATAA---TCGACTGTGCATAAA-CCTTGATGCT
CAAGGTA--GG-CCT-TT---ATAGGGTAGATACACAGT-GCATGGCATTGTGGAACAAG
GCAT--CTCGCGGCTTAGTTGGT-GGGCC-------GCGGGGGCAATGATTAATAGGGAG

GAGCGGGGGCCTTCATATTGCAGGGCGAGAGGTGAAATTCGTTGACCCTTGCAAGATGTC
CTACAGCGAAAGCATTGGCCAAGTGCCTCCCCCTTAGTCAAGAACGAAAGTTTGGGGATC
AAAGACGATCAGATACCGTCGTAGTCCAAACCATAAACTATGTCGACCAGCGATTAGGCG
GGCTACCTTCTTCGAGAGCTGCCTAGCAGCTT-GTGGGAAACCAT-GAGTGCTTGGACTC
TGGGGGGAGTATGGTCGCAAGGCTGAAACTTAAAGGAATTGACGGAAGGGCACACCATGG
AGTGGAG-CCTGCGGCTTAATTT-GACTCAACACGGGAAA-GCTTACCAAGCTCAGATAT
GATTAGGATTGACAGA-CTAA-AAGATCTTTCATGATCTCATAAGTGGTGGTGCATGGTC
GTTCTTAG-TTGGTGGAGTGATTTGTCAGGTCAATTCCGGTAACGGACGAGACCTCGACC
TGCTAACTAGTGGGATTCATTCTTTCG--ATTGACGAGGCAGGTCTTGC-TTTGATTACA
G------GGGGCAACTTC-----TGTAGTCGGG-TACGGTTTGTAG-TAGTCTGGAGGAG
TGG--GTTTCAAA-AATTAAATT---AATCTTCCTAGAGGTACTTCTG-GCTCTAAGCCA
GAGG-AAGTCCGAGGCAATAACAGGTCTGTGATGCCCTTAGATATCTT-GGGCCGCACGC
GTGCTACAATGTAGGCGCTAATGAGTT---TATTTACATCC--ATCTCCGC-AAG---GA
GTATGGTAATC-TTGGAATCACCTGCGTGATTGGGCTTG-ACCACTGTAACTGT-GGTCA
TCAACGAGGAATTCCTTGTATGCGCGAGTCACTATCTCGCGCAGAATCTGTCCCTGCCCT
TTGTACACACCGCCCGTCGCTCCTACCGATCGAACGATCAGGTAAAGTGGACAGACT---
--------------GGAACCCG-C-AAG-GGTA-----------CT-TGG--AAGT-CC-
A-T-TGAACCT--C----GCC--GTT-
>HQ141517.1_Cavenderia_sp._TH18B
TCTAAG-TATAAGCTCTTG-TACGGCTA-GA---CTGCAGACGGCTCATT--ACAA--CG
GTTGT-----AT---CTTCCAGGGCATCCGG-GTCGCA---AGACCTTCT-GGATAACCG
CAGTAAATCGGGGCTAATACATACAAA-CGGAGGGATGGAGAGGGC----AACCTTGAAG
TTTCTGCGATGGACAATTA-GTTATTC--GA-CCAACCC--CG-CAA-GGGAATG--GTT
GGAACCGGTTCATATTGCTAATCGACT-CTAGCTTGCTAGTAGTCTGATAAGCCCTATAG
ACAACCGCCCTATCAACTT--GATGGTAAGGTTTTGGCTTACCATGGTTGTAACGGGTAA
CGGGGAATCAGGGTTCGATTCCGGAGAGGACGCCTGAGAAACGGCGTCCACATCTACGGG
TGGCAGCAGGCGCGTAAATT-GCCCAATCTCAACAGAGAGGAGGCGGTGACAATAAATCC
CGATGGCTTTGGGGGCAACCCCAGG--CTAATCAGAATAAGTACACATTAAATCCCTTAA
CC-AATATAATTGGAGGGCAAGTCTGGTGCCAGCAGCCGCGGTAATTCCAGCTCCAA-TA
GCATATACTAATGTTGTTGCAGTTAAAA-CGC-TCGTAGCT-----TAATATCTTTT--G
AG-CTAC------TATCGATTGTT---ATCCTGCTG--AC------CCGAA--AGGTGAT
GTAGGA------GGC-AGT-CGGCTCAAACAC-TGTAGGTCATG-----CGGC----TAG
CAATA-GTTG----------------CATAA---TCGACTGTGCATAAA-CCTTGATGCT
CAAGGTA--GG-CCT-TT---ATAGGGTAGATACACAGT-GCATGGCATTGTGGAACAAG
GCAT--CTCGCGGCTTAGTTGGT-GGGCC-------GCGGGGGCAATGATTAATAGGGAG
GAGCGGGGGCCTTCATATTGCAGGGCGAGAGGTGAAATTCGTTGACCCTTGCAAGATGTC
CTACAGCGAAAGCATTGGCCAAGTGCCTCCCCATTAGTCAAGAACGAAAGTTTGGGGATC
AAAGACGATCAGATACCGTCGTAGTCCAAACCATAAACTATGTCGACCAGCGATTAGGCG
GGCTACCTTCTTCGAGAGCTGCCTAGCAGCTT-GTGGGAAACCAT-GAGTGCTTGGACTC
TGGGGGGAGTATGGTCGCAAGGCTGAAACTTAAAGGAATTGACGGAAGGGCACACCATGG
AGTGGAG-CCTGCGGCTTAATTT-GACTCAACACGGGAAA-GCTTACCAAGCTCAGATAT
GATTAGGATTGACAGA-CTAA-AAGATCTTTCATGATCTCATAAGTGGTGGTGCATGGTC
GTTCTTAG-TTGGTGGAGTGATTTGTCAGGTCAATTCCGGTAACGGACGAGACCTCGACC
TGCTAACTAGTGGGATTCATTCTTTCG--ATTGACGAGGCAGGTCTTGC-TTTGATTATA
G------GGGGCAACTTC-----TATAGTCGGG-TACGGTTTGTAG-TAGTCTGGAGGAG
TGG--ATTTCAAA-AATTAAATT---AATCTTCCTAGAGGTACTTCTG-GCTCTAAGCCA
GAGG-AAGTCCGAGGCAATAACAGGTCTGTGATGCCCTTAGATATCTT-GGGCCGCACGC
GTGCTACAATGTAGGCGCTAATGAGTT---TATTTACATCC--ATCTCCGC-AAG---GA
GTATGGTAATC-TTGGAATCACCTGCGTGATTGGGCTTG-ACCACTGTAACTGT-GGTCA
TCAACGAGGAATTCCTTGTATGCGCGAGTCACTATCTCGCGCAGAATCTGTCCCTGCCCT
TTGTACACACCGCCCGTCGCTCCTACCGATCGAACGATCAGGTAAAGTGGACAGACT---
--------------GGAGCCCG-C-AAG-GGTT-----------CT-TGG--AAGT-CC-
A-T-TGAACCT--C----GCC--GTT-
>HQ141523.1_Cavenderia_bhumiboliana_THC11X
TCTAAG-TATAAGCTCTTG-TACGGCTA-GA---CTGCAGACGGCTCATT--ACAA--CG
GTTGT-----AT---CTTCCAGGGCATCCGG-GTCGCA---AGACCTTCT-GGATAACCG
CAGTAAATCGGGGCTAATACATACAAA-CGGAGGGATGGAGAGGGC----AACCTTGAAG
TTTCTGCGATGGACAATTA-GTTATTC--GA-CCAACCC--CG-CAA-GGGAATG--GTT
GGAACCGGTTCATATTGCTAATCGACT-CTAGCTTGCTAGTAGTCTGATAAGCCCTATAG
ACAACCGCCCTATCAACTT--GATGGTAAGGTTTTGGCTTACCATGGTTGTAACGGGTAA
CGGGGAATCAGGGTTCGATTCCGGAGAGGACGCCTGAGAAACGGCGTCCACATCTACGGG
TGGCAGCAGGCGCGTAAATT-GCCCAATCTCAACAGAGAGGAGGCGGTGACAATAAATCC
CGATGGCTTTGGGGGCAACCCCAGG--CTAATCAGAATAAGTACACATTAAATCCCTTAA
CC-AATATAATTGGAGGGCAAGTCTGGTGCCAGCAGCCGCGGTAATTCCAGCTCCAA-TA
GCATATACTAATGTTGTTGCAGTTAAAA-CGC-TCGTAGCT-----TAATATCTTTT--G
AG-CTAC------TATCGATTGTT---ATCCTGCTG--AC------CCGAA--AGGTGAT
GTAGGA------GGC-AGT-CGGCTCAAACAC-TGTAGGTCATG-----CGGC----TAG
CAATA-GTTG----------------CATAA---TCGACTGTGCATAAA-CCTTGATGCT
CAAGGTA--GG-CCT-TT---ATAGGGTAGATACACAGT-GCATGGCATTGTGGAACAAG
GCAT--CTCGCGGCTTAGTTGGT-GGGCC-------GCGGGGGCAATGATTAATAGGGAG
GAGCGGGGGCCTTCATATTGCAGGGCGAGAGGTGAAATTCGTTGACCCTTGCAAGATGTC
CTACAGCGAAAGCATTGGCCAAGTGCCTCCCCATTAGTCAAGAACGAAAGTTTGGGGATC
AAAGACGATCAGATACCGTCGTAGTCCAAACCATAAACTATGTCGACCAGCGATTAGGCG

GGCTACCTTCTTCGAGAGCTGCCTAGCAGCTT-GTGGGAAACCAT-GAGTGCTTGGACTC
TGGGGGGAGTATGGTCGCAAGGCTGAAACTTAAAGGAATTGACGGAAGGGCACACCATGG
AGTGGAG-CCTGCGGCTTAATTT-GACTCAACACGGGAAA-GCTTACCAAGCTCAGATAT
GATTAGGATTGACAGA-CTAA-AAGATCTTTCATGATCTCATAAGTGGTGGTGCATGGTC
GTTCTTAG-TTGGTGGAGTGATTTGTCAGGTCAATTCCGGTAACGGACGAGACCTCGACC
TGCTAACTAGTGGGATTCATTCTTTCG--ATTGACGAGGCAGGTCTTGC-TTTGATTATA
G------GGGGCAACTTC-----TATAGTCGGG-TACGGTTTGTAG-TAGTCTGGAGGAG
TGG--ATTTCAAA-AATTAAATT---AATCTTCCTAGAGGTACTTCTG-GCTCTAAGCCA
GAGG-AAGTCCGAGGCAATAACAGGTCTGTGATGCCCTTAGATATCTT-GGGCCGCACGC
GTGCTACAATGTAGGCGCTAATGAGTT---TATTTACATCC--ATCTCCGC-AAG---GA
GTATGGTAATC-TTGGAATCACCTGCGTGATTGGGCTTG-ACCACTGTAACTGT-GGTCA
TCAACGAGGAATTCCTTGTATGCGCGAGTCACTATCTCGCGCAGAATCTGTCCCTGCCCT
TTGTACACACCGCCCGTCGCTCCTACCGATCGAACGATCAGGTAAAGTGGACAGACT---
--------------GGAGCCCG-C-AAG-GGTT-----------CT-TGG--AAGT-CC-
A-T-TGAACCT--C----GCC--GTTT
>MH745572.1_Cavenderia_protodigitata_TH18BA
TCTAAG-TATAAGCTCTTG-TACGGCTA-GA---CTGCAGACGGCTCATT--ACAA--CG
GTTGT-----AT---CTTCCAGGGCATCCGG-GTCGCA---AGACCTTCT-GGATAACCG
CAGTAAATCGGGGCTAATACATACAAA-CGGAGGGATGGAGAGGGC----AACCTTGAAG
TTTCTGCGATGGACAATTA-GTTATTC--GA-CCAACCC--CG-CAA-GGGAATG--GTT
GGAACCGGTTCATATTGCTAATCGACT-CTAGCTTGCTAGTAGTCTGATAAGCCCTATAG
ACAACCGCCCTATCAACTT--GATGGTAAGGTTTTGGCTTACCATGGTTGTAACGGGTAA
CGGGGAATCAGGGTTCGATTCCGGAGAGGACGCCTGAGAAACGGCGTCCACATCTACGGG
TGGCAGCAGGCGCGTAAATT-GCCCAATCTCAACAGAGAGGAGGCGGTGACAATAAATCC
CGATGGCTTTGGGGGCAACCCCAGG--CTAATCAGAATAAGTACACATTAAATCCCTTAA
CC-AATATAATTGGAGGGCAAGTCTGGTGCCAGCAGCCGCGGTAATTCCAGCTCCAA-TA
GCATATACTAATGTTGTTGCAGTTAAAA-CGC-TCGTAGCT-----TAATATCTTTT--G
AG-CTAC------TATCGATTGTT---ATCCTGCTG--AC------CCGAA--AGGTGAT
GTAGGA------GGC-AGT-CGGCTCAAACAC-TGTAGGTCATG-----CGGC----TAG
CAATA-GTTG----------------CATAA---TCGACTGTGCATAAA-CCTTGATGCT
CAAGGTA--GG-CCT-TT---ATAGGGTAGATACACAGT-GCATGGCATTGTGGAACAAG
GCAT--CTCGCGGCTTAGTTGGT-GGGCC-------GCGGGGGCAATGATTAATAGGGAG
GAGCGGGGGCCTTCATATTGCAGGGCGAGAGGTGAAATTCGTTGACCCTTGCAAGATGTC
CTACAGCGAAAGCATTGGCCAAGTGCCTCCCCATTAGTCAAGAACGAAAGTTTGGGGATC
AAAGACGATCAGATACCGTCGTAGTCCAAACCATAAACTATGTCGACCAGCGATTAGGCG
GGCTACCTTCTTCGAGAGCTGCCTAGCAGCTT-GTGGGAAACCAT-GAGTGCTTGGACTC
TGGGGGGAGTATGGTCGCAAGGCTGAAACTTAAAGGAATTGACGGAAGGGCACACCATGG
AGTGGAG-CCTGCGGCTTAATTT-GACTCAACACGGGAAA-GCTTACCAAGCTCAGATAT
GATTAGGATTGACAGA-CTAA-AAGATCTTTCATGATCTCATAAGTGGTGGTGCATGGTC
GTTCTTAG-TTGGTGGAGTGATTTGTCAGGTCAATTCCGGTAACGGACGAGACCTCGACC
TGCTAACTAGTGGGATTCATTCTTTCG--ATTGACGAGGCAGGTCTTGC-TTTGATTATA
G------GGGGCAACTTC-----TATAGTCGGG-TACGGTTTGTAG-TAGTCTGGAGGAG
TGG--ATTTCAAA-AATTAAATT---AATCTTCCTAGAGGTACTTCTG-GCTCTAAGCCA
GAGG-AAGTCCGAGGCAATAACAGGTCTGTGATGCCCTTAGATATCTT-GGGCCGCACGC
GTGCTACAATGTAGGCGCTAATGAGTT---TATTTACATCC--ATCTCCGC-AAG---GA
GTATGGTAATC-TTGGAATCACCTGCGTGATTGGGCTTG-ACCACTGTAACTGT-GGTCA
TCAACGAGGAATTCCTTGTATGCGCGAGTCACTATCTCGCGCAGAATCTGTCCCTGCCCT
TTGTACACACCGCCCGTCGCTCCTACCGATCGAACGATCAGGTAAAGTGGACAGACT---
--------------GGAGCCCG-C-AAG-GGTT-----------CT-TGG--AAGT-CC-
A-T-TGAACCT--C----GCC--GTT-
>HQ141522.1_Cavenderia_myxobasis_NT2A
TCTAAG-TATAAGCTCTTG-TACGGCTA-GA---CTGCAGACGGCTCATT--ACAA--CG
GTTGT-----AT---CTTCCAGGGCATCCGG-GTCGCA---AGACCTTCT-GGATAACCG
CAGTAAATCGGGGCTAATACATACAAA-CGGAGGGATGGAGAGGGC----AACCTTGAAG
TTTCTGCGATGGACAATTA-GTTATTC--TA-CCAACCC--CG-CAA-GGGAATG--GTT
GGAACCGGTTCATATTGCTAATCGACT-CTAGCTTGCTAGTAGTCTGATAAGCCCTATAG
ACAACCGCCCTATCAACTT--GATGGTAAGGTTTTGGCTTACCATGGTTGTAACGGGTAA
CGGGGAATCAGGGTTCGATTCCGGAGAGGACGCCTGAGAAACGGCGTCCACATCTACGGG
TGGCAGCAGGCGCGTAAATT-GCCCAATCTCAACAGAGAGGAGGCGGTGACAATAAATCC
CGATGGCTTTGGGGGCAACCCCAGG--CTAATCAGAATAAGTACACATTAAATCCCTTAA
CC-AATATAATTGGAGGGCAAGTCTGGTGCCAGCAGCCGCGGTAATTCCAGCTCCAA-TA
GCATATACTAATGTTGTTGCAGTTAAAA-CGC-TCGTAGCT-----TAATATCTTTT--G
AG-CTAC------TATCGACTGTT---TTCCTTCTA--AC------TCTTC--GGAGGAG
GAAGG-------GGC-AGT-CGGCTCAAACAC-TGTAGGTCATG-----CGGC----TAG
CAATA-GTTG----------------CATAA---TCGACTGTGCATAAA-CCTTGATGCT
CAAGGTA--GG-CCT-TT---ATAGGGTAGATACACAGT-GCATGGCATTGTGGAACAAG
GCAT--CTCGCGGCTTAGTTGGT-GGGCC-------GCGGGGGCAATGATTAATAGGGAG
GAGCGGGGGCCTTCATATTGCAGGGCGAGAGGTGAAATTCGTTGACCCTTGCAAGATGTC
CTACAGCGAAAGCATTGGCCAAGTGCCTCCCCATTAGTCAAGAACGAAAGTTTGGGGATC
AAAGACGATCAGATACCGTCGTAGTCCAAACCATAAACTATGTCGACCAGCGATTAGGCG
GGCTACCTTCTTCGAGAGCTGCCTAGCAGCTT-GTGGGAAACCAT-GAGTGCTTGGACTC
TGGGGGGAGTATGGTCGCAAGGCTGAAACTTAAAGGAATTGACGGAAGGGCACACCATGG
AGTGGAG-CCTGCGGCTTAATTT-GACTCAACACGGGAAA-GCTTACCAAGCTCAGATAT

GATTAGGATTGACAGA-CTAA-AAGATCTTTCATGATCTCATAAGTGGTGGTGCATGGTC
GTTCTTAG-TTGGTGGAGTGATTTGTCAGGTCAATTCCGGTAACGGACGAGACCTCGACC
TGCTAACTAGTGGGATTCATTCTTTCG--ATTGACGAGGCAGGTATTGC-TTTGATTATA
G------GGGGCAACTTC-----TATAGTCGGG-TAGTGCTTGTAG-TAGTCTGGAGGAG
TGG--ATTTCAAA-AATTAAATT---AATCTTCCTAGAGGTACTTCTG-GCTCTAAGCCA
GAGG-AAGTCCGAGGCAATAACAGGTCTGTGATGCCCTTAGATATCTT-GGGCCGCACGC
GTGCTACAATGTAGGCGCTAATGAGTT---TATTTACATCC--ATCTCCGC-AAGT--GA
GTATGGTAATC-TTGGAATCACCTGCGTGATTGGGCTTG-ACCACTGTAACTGT-GGTCA
TCAACGAGGAATTCCTTGTATGCGCGAGTCACTATCTCGCGCAGAATCTGTCCCTGCCCT
TTGTACACACCGCCCGTCGCTCCTACCGATCGAACGATCAGGTAAAGTGGACAGACT---
--------------GGAACCCG-C-AAG-GGTA-----------CT-TGG--AAGT-CC-
A-T-TGAACCT--C----GCC--GTTT
>AM168087.1_Cavenderia_fasciculata_SH3
TCTAAG-TATAAGCTCTTG-TACGGCTA-GA---CTGCAGACGGCTCATT--ACAA--CG
GTTGT-----AT---CTTACAGGGCATCCGG-GTCGCA---AGACCTTCT-GGATAACCG
CAGTAAATCGGGGCTAATACATATAAA-CGGAGGGATGGAGAGGGT----AACCTTGAAG
TTTCTGCGATGGACATTTA-GCTATTC--GA-CCAACCC--CG-CAA-GGGAATG--GTT
GGAACCGGTTCATATTGCTAATCGACT-CTAGCTTGCTAGTAGTCTGATAAGCCCTATAG
ACAACCGCCCTATCAACTT--GATGGTAAGGTTTTGGCTTACCATGGTTGTAACGGGTAA
CGGGGAATCAGGGTTCGATTCCGGAGAGGACGCCTGAGAAACGGCGTCCACATCTACGGG
TGGCAGCAGGCGCGTAAATT-GCCCAATCTCAACAGAGAGGAGGCGGTGACAATAAATCC
CGATGGCTTTGGGGGCAACCCCAGG--CTAATCAGAATAAGTACACATTAAATCCCTTAA
CC-AATATAATTGGAGGGCAAGTCTGGTGCCAGCAGCCGCGGTAATTCCAGCTCCAA-TA
GCATATACTAATGTTGTTGCAGTTAAAA-CGC-TCGTAGCT-----TAATATCTTTT--G
AG-CTAC------TATCGACTGTC---ATCCTCTCTTG-ATC----CGTTAAAAG-TGAG
AGGGAG------GACCAGT-CGGCTCAAACAC-TGTAGGTCATG-----CGGC----TAG
CAATA-GTTG----------------CATAA---TCGACTGTGCATAAA-CCTTGATGCT
CAAGGTA--GG-CCT-TT---ATAGGGTAGATACACAGT-GCATGGCATTGTGGAACAAG
GCAT--CTCGCGGCTTAGTTGGT-GGGCC-------GCGGGGGCAATGATTAATAGGGAG
GAGCGGGGGCCTTCATATTGCAGGGCGAGAGGTGAAATTCGTTGACCCTTGCAAGATGTC
CTACAGCGAAAGCATTGGCCAAGTGCCTCCCCATTAGTCAAGAACGAAAGTTTGGGGATC
AAAGACGATCAGATACCGTCGTAGTCCAAACCATAAACTATGTCGACCAGCGATTAGGCG
GGCTACCTTCTTCGAGAGCTGCCTAGCAGCTT-GTGGGAAACCAT-GAGTGCTTGGACTC
TGGGGGGAGTATGGTCGCAAGGCTGAAACTTAAAGGAATTGACGGAAGGGCACACCATGG
AGTGGAG-CCTGCGGCTTAATTT-GACTCAACACGGGAAA-GCTTACCAAGCTCAGATAT
GATTAGGATTGACAGA-CTAA-AAGATCTTTCATGATCTCATAAGTGGTGGTGCATGGTC
GTTCTTAG-TTGGTGGAGTGATTTGTCAGGTCAATTCCGGTAACGGACGAGACCTCGACC
TGCTAACTAGTGGGATTCATTCTTTCG--ATTGACGAGGCAGGTATTGC-TTTGATTATA
G------GGGGCAACTTT-----TATAGTCGGG-TAGTGCTTGTAT-TAGTCTGGGAGAG
TGG--GTTTCAAA-AATTTAATT---AATCTTCCTAGAGGTACTTCTG-GCTCTAAGCCA
GAGG-AAGTCCGAGGCAATAACAGGTCTGTGATGCCCTTAGATATCTT-GGGCCGCACGC
GTGCTACAATGTAGGCGCTAATGAGTT---TATTTACATCC--ATCTCCGC-AAG---GA
GTATGGTAATC-TTGGAATCCCCTGCGTGATTGGGCTTG-ACCACTGTAACTGT-GGTCA
TCAACGAGGAATTCCTTGTATGCGCGAGTCACTATCTCGCGCAGAATCTGTCCCTGCCCT
TTGTACACACCGCCCGTCGCTCCTACCGATCGAACGATCAGGTAAAGTGGACAGACT---
--------------TTGGCCCG-C-AAG-GGTT-----------GT-TGG--AAGT-CC-
A-T-TGAACCT--C----GCC--GTTT
>GQ496157.1_Cavenderia_fasciculoidea
TCTAAG-TATAAGCTCTTG-TACGGCTA-GA---CTGCAGACGGCTCATT--ACAA--CG
GTTGT-----AT---CTTACAGGGCATCCGG-GTCGCA---AGACCTTCT-GGATAACCG
CAGTAAATCGGGGCTAATACATATAAA-CGGAGGGATGGAGAGGGT----AACCTTGAAG
TTTCTGCGATGGACATTTA-GCTATTC--GA-CCAACCC--CG-CAA-GGGAATG--GTT
GGAACCGGTTCATATTGCTAATCGACT-CTAGCTTGCTAGTAGTCTGATAAGCCCTATAG
ACAACCGCCCTATCAACTT--GATGGTAAGGTTTTGGCTTACCATGGTTGTAACGGGTAA
CGGGGAATCAGGGTTCGATTCCGGAGAGGACGCCTGAGAAACGGCGTCCACATCTACGGG
TGGCAGCAGGCGCGTAAATT-GCCCAATCTCAACAGAGAGGAGGCGGTGACAATAAATCC
CGATGGCTTTGGGGGCAACCCCAGG--CTAATCAGAATAAGTACACATTAAATCCCTTAA
CC-AATATAATTGGAGGGCAAGTCTGGTGCCAGCAGCCGCGGTAATTCCAGCTCCAA-TA
GCATATACTAATGTTGTTGCAGTTAAAA-CGC-TCGTAGCT-----TAATATCTTTT--G
AG-CTAC------TATCGACTGTC---ATCCTCTCTTG-ATC----CGTTAAAAG-TGAG
AGGGAG------GACCAGT-CGGCTCAAACAC-TGTAGGTCATG-----CGGC----TAG
CAATA-GTTG----------------CATAA---TCGACTGTGCATAAA-CCTTGATGCT
CAAGGTA--GG-CCT-TT---ATAGGGTAGATACACAGT-GCATGGCATTGTGGAACAAG
GCAT--CTCGCGGCTTAGTTGGT-GGGCC-------GCGGGGGCAATGATTAATAGGGAG
GAGCGGGGGCCTTCATATTGCAGGGCGAGAGGTGAAATTCGTTGACCCTTGCAAGATGTC
CTACAGCGAAAGCATTGGCCAAGTGCCTCCCCATTAGTCAAGAACGAAAGTTTGGGGATC
AAAGACGATCAGATACCGTCGTAGTCCAAACCATAAACTATGTCGACCAGCGATTAGGCG
GGCTACCTTCTTCGAGAGCTGCCTAGCAGCTT-GTGGGAAACCAT-GAGTGCTTGGACTC
TGGGGGGAGTATGGTCGCAAGGCTGAAACTTAAAGGAATTGACGGAAGGGCACACCATGG
AGTGGAG-CCTGCGGCTTAATTT-GACTCAACACGGGAAA-GCTTACCAAGCTCAGATAT
GATTAGGATTGACAGA-CTAA-AAGATCTTTCATGATCTCATAAGTGGTGGTGCATGGTC
GTTCTTAG-TTGGTGGAGTGATTTGTCAGGTCAATTCCGGTAACGGACGAGACCTCGACC
TGCTAACTAGTGGGATTCATTCTTTCG--ATTGACGAGGCAGGTATTGC-TTTGATTATA

G------GGGGCAACTTT-----TATAGTCGGG-TAGTGCTTGTAT-TAGTCTGGGAGAG
TGG--GTTTCAAA-AATTTAATT---AATCTTCCTAGAGGTACTTCTG-GCTCTAAGCCA
GAGG-AAGTCCGAGGCAATAACAGGTCTGTGATGCCCTTAGATATCTT-GGGCCGCACGC
GTGCTACAATGTAGGCGCTAATGAGTT---TATTTACATCC--ATCTCCGC-AAG---GA
GTATGGTAATC-TTGGAATCCCCTGCGTGATTGGGCTTG-ACCACTGTAACTGT-GGTCA
TCAACGAGGAATTCCTTGTATGCGCGAGTCACTATCTCGCGCAGAATCTGTCCCTGCCCT
TTGTACACACCGCCCGTCGCTCCTACCGATCGAACGATCAGGTAAAGTGGACAGACT---
--------------TTGGCCCG-C-AAG-GGTT-----------GT-TGG--AAGT-CC-
A-T-TGAACCT--C----GCC--GTT-
>AM168093.1_Cavenderia_delicata_TNS_C_226
TCTAAG-TATAAGCTCTTG-TACGGCTA-GA---CTGCAGACGGCTCATT--ACAA--CG
GTTGT-----AT---CTTACAGGGCATCCGG-GTCGCA---AGACCTTCT-GGATAACCG
CAGTAAATCGGGGCTAATACATACAAA-CGGAGGGATGGAGAGGGT----AACCTTGAAG
TTTCTGCGATGGACATTTA-GCTATTC--GA-CCAACCC--CG-CAA-GGGAATG--GTT
GGAACCGGTTCATATTGCTAATCGACT-CTAGCTTGCTAGTAGTCTGATAAGCCCTATAG
ACAACCGCCCTATCAACTT--GATGGTAAGGTTTTGGCTTACCATGGTTGTAACGGGTAA
CGGGGAATCAGGGTTCGATTCCGGAGAGGACGCCTGAGAAACGGCGTCCACATCTACGGG
TGGCAGCAGGCGCGTAAATT-GCCCAATCTCAACAGAGAGGAGGCGGTGACAATAAATCC
CGATGGCTTTGGGGGCAACCCCAGG--CTAATCAGAATAAGTACACATTAAATCCCTTAA
CC-AATATAATTGGAGGGCAAGTCTGGTGCCAGCAGCCGCGGTAATTCCAGCTCCAA-TA
GCATATACTAATGTTGTTGCAGTTAAAA-CGC-TCGTAGCT-----TAATATCTTTT--G
AG-CTAC------TATCGACTGTC---ATCCTTTTGTG-GCG----CCTTCGGGT-TAAG
CAGGAG------GACCGGT-CGGCTCAAACAC-TGTAGGTCATG-----CGGC----TAG
CAATA-GTTG----------------CATAA---TCGACTGTGCATAAA-CCTTGATGCT
CAAGGTA--GG-CCT-TT---ATAGGGTAGATACACAGT-GCATGGCATTGTGGAACAAG
GCAT--CTCGCGGCTTAGTTGGT-GGGCC-------GCGGGGGCAATGATTAATAGGGAG
GAGCGGGGGCCTTCATATTGCAGGGCGAGAGGTGAAATTCGTTGACCCTTGCAAGATGTC
CTACAGCGAAAGCATTGGCCAAGTGCCTCCCCATTAGTTAAGAACGAAAGTTTGGGGATC
AAAGACGATCAGATACCGTCGTAGTCCAAACCATAAACTATGTCGACCAGCGATTAGGCG
GGCTACCTTCTTCGAGAGCTGCCTAGCAGCTT-GTGGGAAACCAT-GAGTGCTTGGACTC
TGGGGGGAGTATGGTCGCAAGGCTGAAACTTAAAGGAATTGACGGAAGGGCACACCATGG
AGTGGAG-CCTGCGGCTTAATTT-GACTCAACACGGGAAA-GCTTACCAAGCTCAGATAT
GATTAGGATTGACAGA-CTAA-AAGATCTTTCATGATCTCATAAGTGGTGGTGCATGGTC
GTTCTTAG-TTGGTGGAGTGATTTGTCAGGTCAATTCCGGTAACGGACGAGACCTCGACC
TGCTAACTAGTGGGATTCATTCTTTCG--ATTGACGAGGCAGGTATTGC-TTTGATTATA
G------GGGGCAACTTT-----TATAGTCGGG-TAGTGCTTGTAT-TAGTCTGGGAGAG
TGG--GTTTCAAA-AATTTAATT---AATCTTCCTAGAGGTACTTCTG-GCTCTAAGCCA
GAGG-AAGTCCGAGGCAATAACAGGTCTGTGATGCCCTTAGATATCTT-GGGCCGCACGC
GTGCTACAATGTAGGCGCTAATGAGTT---TATTTACATCC--ATCTCCGC-AAG---GA
GTATGGTAATC-TTGGAATCCCCTGCGTGATTGGGCTTG-ACCACTGTAACTGT-GGTCA
TCAACGAGGAATTCCTTGTATGCGCGAGTCACTATCTCGCGCAGAATCTGTCCCTGCCCT
TTGTACACACCGCCCGTCGCTCCTACCGATCGAACGATCAGGTAAAGTGGACAGACT---
--------------GAGACTCG-C-AAG-AGTT-----------TT-TGG--AAGT-CC-
A-T-TGAACCT--C----GCC--GTTT
>AM168086.1_Cavenderia_fasciculata_SmokOW9A
TCTAAG-TATAAGCTCTTG-TACGGCTA-GA---CTGCAGACGGCTCATT--ACAA--CG
GTTGT-----AT---CTTACAGGGCATCCGG-GTCGCA---AGACCTTCT-GGATAACCG
CAGTAAATCGGGGCTAATACATACAAA-CGGAGGGATGGAGAGGGC----AACCTTGAAG
TTTCTGCGATGGACATTTA-GCTATTC--GA-CCAACCC--CG-CAA-GGGAATG--GTT
GGAACCGGTTCATATTGCTAATCGACT-CTAGCTTGCTAGTAGTCTGATAAGCCCTATAG
ACAACCGCCCTATCAACTT--GATGGTAAGGTTTTGGCTTACCATGGTTGTAACGGGTAA
CGGGGAATCAGGGTTCGATTCCGGAGAGGACGCCTGAGAAACGGCGTCCACATCTACGGG
TGGCAGCAGGCGCGTAAATT-GCCCAATCTCAACAGAGAGGAGGCGGTGACAATAAATCC
CGATGGCTTTGGGGGCAACCCCAGG--CTAATCAGAATAAGTACACATTAAATCCCTTAA
CC-AATATAATTGGAGGGCAAGTCTGGTGCCAGCAGCCGCGGCAATTCCAGCTCCAA-TA
GCATATACTAATGTTGTTGCAGTTAAAAACGC-TCGTAGCT-----TAATATCTTTT--G
AG-CTAC------TATCGACTGTC---ATCCTCTTATGT-AG----CCGCAAGGT-GAAG
TAGGAG------GGCCAGT-CGGCTCAAACAC-TGTAGGTCATG-----CGGC----TAG
CAATA-GTTG----------------CATAA---TCGACTGTGCATAAA-CCTTGATGCT
CAAGGTA--GG-CCT-TT---ATAGGGTAGATACACAGT-GCATGGCATTGTGGAACAAG
GCAT--CTCGCGGCTTAGTTGGT-GGGCC-------GCGGGGGCAATGATTAATAGGGAG
GAGCGGGGGCCTTCATATTGCAGGGCGAGAGGTGAAATTCGTTGACCCTTGCAAGATGTC
CTACAGCGAAAGCATTGGCCAAGTGCCTCCCCATTAGTCAAGAACGAAAGTTTGGGGATC
AAAGACGATCAGATACCGTCGTAGTCCAAACCATAAACTATGTCGACCAGCGATTAGGCG
GGCTACCTTCTTCGAGAGCTGCCTAGCAGCTT-GTGGGAAACCAT-GAGTGCTTGGACTC
TGGGGGGAGTATGGTCGCAAGGCTGAAACTTAAAGGAATTGACGGAAGGGCACACCATGG
AGTGGAG-CCTGCGGCTTAATTTTGACTCAACACGGGAAAAGCTTACCAAGCTCAGATAT
GATTAGGATTGACAGAGCTAA-AAGATCTTTCATGATCTCATAAGTGGTGGTGCATGGTC
GTTCTTAGGTTGGTGGAGTGATTTGTCAGGTCAATTCCGGTAACGGACGAGACCTCGACC
TGCTAACTAGTGGGATTCATTCTTTCG--ATTGACGAGGCAGGTATTGC-TTTGATTATA
G------GGGGCAACTTT-----TATAGTCGGG-TAGTGCTTGTAT-TAGTCTGGGAGAG
TGG--GTTTCAAA-AATTTAATT---AATCTTCCTAGAGGTACTTCTG-GCTCTAAGCCA
GAGG-AAGTCCGAGGCAATAACAGGTCTGTGATGCCCTTAGATATCTT-GGGCCGCACGC

GTGCTACAATGTAGGCGCTAATGAGTT---TATTTACATCC--ATCTCCGC-AAG---GA
GTATGGTAATC-TTGGAATCCCCTGCGTGATTGGGCTTG-ACCACTGTAACTGT-GGTCA
TCAACGAGGAATTCCTTGTATGCGCGAGTCACTATCTCGCGCAGAATCTGTCCCTGCCCT
TTGTACACACCGCCCGTCGCTCCTACCGATCGAACGATC-GGTAAAGTGGACAGACT---
--------------GAAACCCG-C-AAG-GGTT-----------TT-TGG--AAGT-CCT
A-T-TGAACCT--C----GCC--GTTT
>HQ141516.1_Cavenderia_sp._TAS30A
TCTAAG-TATAAGCTCTTG-TACGGCTA-GA---CTGCAGACGGCTCATT--ACAA--CG
GTTGT-----AT---CTTACAGGGCATCCGG-GTCGCA---AGACCTTCT-GGATAACCG
CAGTAAATCGGGGCTAATACATACAAA-CGGAGGGATGGAGAGGGC----AACCTTGAAG
TTTCTGCGATGGACATTTA-GCTATTC--GA-CCAACCC--CG-CAA-GGGAATG--GTT
GGAACCGGTTCATATTGCTAATCGACT-CTAGCTTGCTAGTAGTCTGATAAGCCCTATAG
ACAACCGCCCTATCAACTT--GATGGTAAGGTTTTGGCTTACCATGGTTGTAACGGGTAA
CGGGGAATCAGGGTTCGATTCCGGAGAGGACGCCTGAGAAACGGCGTCCACATCTACGGG
TGGCAGCAGGCGCGTAAATT-GCCCAATCTCAACAGAGAGGAGGCGGTGACAATAAATCC
CGATGGCTTTGGGGGCAACCCCAGG--CTAATCAGAATAAGTACACATTAAATCCCTTAA
CC-AATATAATTGGAGGGCAAGTCTGGTGCCAGCAGCCGCGGTAATTCCAGCTCCAA-TA
GCATATACTAATGTTGTTGCAGTTAAAA-CGC-TCGTAGCT-----TAATATCTTTT--G
AG-CTAC------TATCGACTGTC---ATCCTCTTATGTAAG----CCGCAAGGTCCAAG
TAAGAG------GACCAGT-CGGCTCAAACAC-TGTAGGTCATG-----CGGC----TAG
CAATA-GTTG----------------CATAA---TCGACTGTGCATAAA-CCTTGATGCT
CAAGGTA--GG-CCT-TT---ATAGGGTAGATACACAGT-GCATGGCATTGTGGAACAAG
GCAT--CTCGCGGCTTAGTTGGT-GGGCC-------GCGGGGGCAATGATTAATAGGGAG
GAGCGGGGGCCTTCATATTGCAGGGCGAGAGGTGAAATTCGTTGACCCTTGCAAGATGTC
CTACAGCGAAAGCATTGGCCAAGTGCCTCCCCATTAGTCAAGAACGAAAGTTTGGGGATC
AAAGACGATCAGATACCGTCGTAGTCCAAACCATAAACTATGTCGACCAGCGATTAGGCG
GGCTACCTTCTTCGAGAGCTGCCTAGCAGCTT-GTGGGAAACCAT-GAGTGCTTGGACTC
TGGGGGGAGTATGGTCGCAAGGCTGAAACTTAAAGGAATTGACGGAAGGGCACACCATGG
AGTGGAG-CCTGCGGCTTAATTT-GACTCAACACGGGAAA-GCTTACCAAGCTCAGATAT
GATTAGGATTGACAGA-CTAA-AAGATCTTTCATGATCTCATAAGTGGTGGTGCATGGTC
GTTCTTAG-TTGGTGGAGTGATTTGTCAGGTCAATTCCGGTAACGGACGAGACCTCGACC
TGCTAACTAGTGGGATTCATTCTTTCG--ATTGACGAGGCAGGTATTGC-TTTGATTATA
G------GGGGCAACTTT-----TATAGTCGGG-TAGTGCTTGTAT-TAGTCTGGGAGAG
TGG--GTTTCAAA-AATTTAATT---AATCTTCCTAGAGGTACTTCTG-GCTCTAAGCCA
GAGG-AAGTCCGAGGCAATAACAGGTCTGTGATGCCCTTAGATATCTT-GGGCCGCACGC
GTGCTACAATGTAGGCGCTAATGAGTT---TATTTACATCC--ATCTCCGC-AAG---GA
GTATGGTAATC-TTGGAATCCCCTGCGTGATTGGGCTTG-ACCACTGTAACTGT-GGTCA
TCAACGAGGAATTCCTTGTATGCGCGAGTCACTATCTCGCGCAGAATCTGTCCCTGCCCT
TTGTACACACCGCCCGTCGCTCCTACCGATCGAACGATCAGGTAAAGTGGACAGACT---
--------------GAAACCCG-C-AAG-GGTT-----------TT-TGG--AAGT-CC-
A-T-TGAACCT--C----GCC--GTT-
>AM168080.1_Cavenderia_antarctica_NZ43B
TCTAAG-TATAAGCTCTTG-TACGGCTA-GA---CTGCAGACGGCTCATT--ACAA--CG
GTTGT-----AT---CTTACAGGGCATCCGG-GTCGCA---AGACCTTCT-GGATAACCG
CAGTAAATCGGGGCTAATACATACAAA-CGGAGGGATGGAGAGGGC----AACCTTGAAG
TTTCTGCGATGGACATTTA-GCTATTC--GA-CCAACCC--CG-CAA-GGGAATG--GTT
GGAACCGGTTCATATTGCTAATCGACT-CTAGCTTGCTAGTAGTCTGATAAGCCCTATAG
ACAACCGCCCTATCAACTT--GATGGTAAGGTTTTGGCTTACCATGGTTGTAACGGGTAA
CGGGGAATCAGGGTTCGATTCCGGAGAGGACGCCTGAGAAACGGCGTCCACATCTACGGG
TGGCAGCAGGCGCGTAAATT-GCCCAATCTCAACAGAGAGGAGGCGGTGACAATAAATCC
CGATGGCTTTGGGGGCAACCCCAGG--CTAATCAGAATAAGTACACATTAAATCCCTTAA
CC-AATATAATTGGAGGGCAAGTCTGGTGCCAGCAGCCGCGGTAATTCCAGCTCCAA-TA
GCATATACTAATGTTGTTGCAGTTAAAA-CGC-TCGTAGCT-----TAATATCTTTT--G
AG-CTAC------TATCGACTGTC---ATCCTCTTATGTAAG----CCGCAAGGTCCAAG
TAAGAG------GACCAGT-CGGCTCAAACAC-TGTAGGTCATG-----CGGC----TAG
CAATA-GTTG----------------CATAA---TCGACTGTGCATAAA-CCTTGATGCT
CAAGGTA--GG-CCT-TT---ATAGGGTAGATACACAGT-GCATGGCATTGTGGAACAAG
GCAT--CTCGCGGCTTAGTTGGT-GGGCC-------GCGGGGGCAATGATTAATAGGAAG
GAGCGGGGGCCTTCATATTGCAGGGCGAGAGGTGAAATTCGTTGACCCTTGCAAGATGTC
CTACAGCGAAAGCATTGGCCAAGTGCCTCCCCATTAGTCAAGAACGAAAGTTTGGGGATC
AAAGACGATCAGATACCGTCGTAGTCCAAACCATAAACTATGTCGACCAGCGATTAGGCG
GGCTACCTTCTTCGAGAGCTGCCTAGCAGCTT-GTGGGAAACCAT-GAGTGCTTGGACTC
TGGGGGGAGTATGGTCGCAAGGCTGAAACTTAAAGGAATTGACGGAAGGGCACACCATGG
AGTGGAG-CCTGCGGCTTAATTT-GACTCAACACGGGAAA-GCTTACCAAGCTCAGATAT
GATTAGGATTGACAGA-CTAA-AAGATCTTTCATGATCTCATAAGTGGTGGTGCATGGTC
GTTCTTAG-TTGGTGGAGTGATTTGTCAGGTCAATTCCGGTAACGGACGAGACCTCGACC
TGCTAACTAGTGGGATTCATTCTTTCG--ATTGACGAGGCAGGTATTGC-TTTGATTATA
G------GGGGCAACTTT-----TATAGTCGGG-TAGTGCTTGTAT-TAGTCTGGGAGAG
TGG--GTTTCAAA-AATTTAATT---AATCTTCCTAGAGGTACTTCTG-GCTCTAAGCCA
GAGG-AAGTCCGAGGCAATAACAGGTCTGTGATGCCCTTAGATATCTT-GGGCCGCACGC
GTGCTACAATGTAGGCGCTAATGAGTT---TATTTACATCC--ATCTCCGC-AAG---GA
GTATGGTAATC-TTGGAATCCCCTGCGTGATTGGGCTTG-ACCACTGTAACTGT-GGTCA
TCAACGAGGAATTCCTTGTATGCGCGAGTCACTATCTCGCGCAGAATCTGTCCCTGCCCT

TTGTACACGCCGCCCGTCGCTCCTACCGATCGAACGATCAGGTAAAGTGGACAGACT---
--------------GAAACCCG-C-AAG-GGTT-----------TT-TGG--AAGT-CC-
A-T-TGAACCT--C----GCC--GTTT
>MH745571.1_Cavenderia_aureostabilis_TH10B
TCTAAG-TATAAGCTCTTG-TACGGCTA-GA---CTGCAGACGGCTCATT--ACAA--CG
GTTGT-----AT---CTTCCAGGACATCCGG-GTCGCA---AGATCTTCT-GGATAACCG
CAGTAAATCGGGGCTAATACATACAAA-CGAAGGGGTGGAGAGGGC----AACCTTGAAG
CTTCTGCGATGGACACTTA-GCTATTC--GA-CCAACCC--CG-CAA-GGGAGTG--GTT
GGAACCGGTTCATATTGCTAATCGACT-CTGGCTTGCCATGAGTC-GATAAGTCCTATAG
ACAACCGCCCTATCAACTT--GATGGTAAGGTTTTGGCTTACCATGGTTGTAACGGGTAA
CGGGGAATCAGGGTTCGATTCCGGAGAGGACGCCTGAGAAACGGCGTCCACATCTACGGG
TGGCAGCAGGCGCGTAAATT-GCCCAATCTCAACAGAGAGGAGGCGGTGACAATAAATCC
CGATGGCTTTGGGGGCAACCCCAGG--CTAATCAGAATAAGTACACATTAAATCCCTTAA
CC-AATATAATTGGAGGGCAAGTCTGGTGCCAGCAGCCGCGGTAATTCCAGCTCCAA-TA
GCATATACTAATGTTGTTGCAGTTAAAA-CGC-TCGTAGCT-----TAATTTCTTTT--G
AG-CTAC------TTACGGTTGTCA--AACCTG----CGAAG----TCTTCGG-ACCTAG
TAGGA--------ACAAC--CGGCTCAAACAC-TGTAGGTCATG------GGC---TTAG
CAATA-AGTT----------------CATAA---TCGACTGTGCATAAA-CCTTGATGCT
CAAGGTA--GG-CCT-TT---ATAGGGTAGATACACAGT-GCATGGCATTGTGGAACAAG
GCAT--CTCGCGGCTTAGTTGGT-GGGCC-------GCGGGGGCAATGATTAATAGGGAG
GAGCGGGGGCCTTCATATTGCAGGGCGAGAGGTGAAATTCGTTGACCCTTGCAAGATGTC
CGACAGCGAAAGCATTGGCCAAGTGCCTCCCCATTAGTCAAGAACGAAAGTTTGGGGATC
AAAGACGATCAGATACCGTCGTAGTCCAAACCATAAACTATGTCGACCAGCGATTAGGCG
GGCTACCTTCTTCGAGAGCTGCCTAGCAGCTT-GTGGGAAACCAT-GAGTGCTTGGACTC
TGGGGGGAGTATGGTCGCAAGGCTGAAACTTAAAGGAATTGACGGAAGGGCACACCATGG
AGTGGAG-CCTGCGGCTTAATTT-GACTCAACACGGGAAA-GCTTACCAAGCTCAGATAT
GATAAGGATTGACAGA-CTAA-AAGATCTTTCATGATCTCATAAGTGGTGGTGCATGGTC
GTTCTTAG-TTGGTGGAGTGATTTGTCAGGTCAATTCCGGTAACGGACGAGACCTCGACC
TGCTAACTAGTGGGATTCATTCCTTCG--CTCGACGAGGCGGGCTCTGC-TTTTATTATG
A------GGG-CAACT-T-----CATAGTAGGG-TAGGGTTCGTAT-TAGTCTGGGGGAG
TGG--GTTTCAAA--ATTTAATTT--AATCTTCCTAGAGGTACTTCTG-GCTCTAAGCCA
GAGG-AAGTTCGAGGCAATAACAGGTCTGTGATGCCCTTAGATATCTT-GGGCCGCACGC
GTGCTACAATGTAGGCGCTAATGAGTC---TTAATACATCC--AGCTCCGT-AAG---GA
GTCTGGTAATC-TTGGAATCCCCTGCGTGATTGGGCTTG-ACTACTGTAACTGT-GGTCA
TCAACGAGGAATTCCTTGTATGCGCGAGTCACTATCTCGCGCAGAATCTGTCCCTGCCCT
TTGTACACACCGCCCGTCGCTCCTACCGATCGAACGATCAGGTAAAGTGGACAGACT---
--------------AGAGGCCG-C-AAG-GCAT-----------TT-GGA--AAGT-CC-
A-T-TGAACCT--C----GCC--GTTT
>HQ141519.1_Cavenderia_macrocarpa_MGE2
TCTAAG-TATAAGCCCTTG-TACGGCTA-GA---CTGCAGACGGCTCATT--ACAA--CG
GTTGC-----AT---CTTCCAGAGCATCCGG-GTCGCA---AGACCTTCT-GGATAACCG
CAGTAAATCGGGGCTAATACATACAAA-CGGAGGGGTGGACCAGGC----AACTGGGAAA
CTTCTGCGATGGACACTTA-GCTATTC--GA-CTGACCC--TT-TCG-GGGAATG--GTT
GGAACCGGTTCATATTGCTAATCGATTTGTGGCTTGCCACAAGTCTGATAAGCTCTATAG
GCAACCGCCCTATCAACTT--GATGGTAAGGTTTTGGCTTACCATGGTTGTAACGGGTAA
CGGGGAATCAGGGTTCGATTCCGGAGAGGACGCCTGAGAAACGGCGTCCACATCTACGGG
TGGCAGCAGGCGCGTAAATT-GCCCAATCTCAATAGAGAGGAGGCGGTGACAATAAATCC
CAATGACCATGGGGGCAACCCCGGG--TCAATTAGAATAAGTACACATTAAATACCCTAA
CCCAATATAATTGGAGGGCAAGTCTGGTGCCAGCAGCCGCGGTAATTCCAGCTCCAA-TA
GCGTATACTAATGTTGTTGCAGTTAAAA-CGC-TCGTAGCT-----AAAGATCTTGATGG
CAAATTC------TTGTGAAGACTCCATAGTCCTCTGGGGAAA---CCCATTGTGACTTT
TGGAC--------TTTAC--AGGC-CA-GCAA--GTAGGTAGTCCTG--GGCAT--GAGG
GTAAC-CTTGTGTTT-----------AGCGACGATCGACTGTGCATAAA-CCTTGATGCT
CAAGGTAAACA-AAA-TC--TTCTTGAAAGATACACGGT-GCATGGCATTGTGGAATATG
GCGTC-TTTGTGTCCTTGTTGGTTGGGAC-------ACAAGAGCAATGATTAATAGGGAG
GAGCGGGGGCCTTCATATTGCCGGGCGAGAGGTGAAATTCGTTGACCCTGGCAAGATGTC
CTACAGCGAAAGCATTGGCCAAGTGCCTCTCCATTAGTCAAGAACGAAAGTTTGGGGATC
AAAGACGATCAGATACCGTCGTAGTCCAAACCATAAACTATGTCGACCAGCGATTAGGTT
CGCCACCTTCTTCGAGAGCAACCTGGCAGCTT-GTGGGAAACCAT-GAGTTCTTGGACTC
TGGGGGGAGTATGGTCGCAAGGCTGAAACTTAAAGGAATTGACGGAAGGGCACACCATGG
AGTGGAG-CCTGCGGCTTAATTT-GACTCAACACGGGAAA-GCTTACCAAGCTCAGATAT
GATAAGGATTGACAGA-CCAA-AAGATCTTTCATGATCTCATAAGTGGTGGTGCATGGCC
GTTCTTAG-TTGGTGGAGTGATTTGTCAGGTCAATTCCGGTAACGGACGAGACCTCGACC
TGCTAACTAGTGGCAGTTGTCTTTTCG--ATCGGCGAGGCGTCGTGGGCATGGAACTTCT
A--T--AGGGGCAACTCTATGG-CTTGGGAAGTGTCCACGATGTAG-TAGTCTGGAGAGG
CAG--CTGTCCCCAAA--AATAT---AATCTTCCTAGAGGTACTTCCA-GCTCTAAGTTG
GAGG-AAGTTCGAGGCAATAACAGGTCTGTGATGCCCTTAGATATCTT-GGGCCGCACGC
GTGCTACAATGTAGGCGCTAATAAGAAAAACATTT-TATCCCCGGCACCGCCAAG---GT
GTCCGGTAATC-CA-CAATCACCTGCGTGATTGGGATTG-GTCACTGTAACTGT-GACCA
TCAACGAGGAATTCCTTGTATGCGCGGGTCACTATCCCGCGCAGAATCTGTCCCTGCCCT
TTGTACACACCGCCCGTCGCTCCTACCGATCGAACGGTAAGGTAAAGTGGACAGACA---
-------------G-T-CCCT-C-T-T-TATTG-AGGA-----GC-TGA--AAGT-CC-
A-T-TGAACCT--C----GTC--GTT-

>AM168091.1_Cavenderia_parvispora_OS126
TCTAAG-TATAAGCCCTAG-TACGGCTA-GA---CTGCAGACGGCTCATT--ACAA--CG
GTTGC-----AG---CTTACAGAGCATCCGG-GTCGCA---AGACCTTCT-GGATAACCG
CAGTAAATCGGGGCTAATACATACAAA-CGGAGGGGCGGAGCGGGC----AACCGCGAAG
CTTCTGCGATGGACACTTA-GCTATTC--GA-CTGACCC--CT-CAC-GGGAATG--GTT
GGAACCGGTTCATATTGCTAATCGACTTGTGGCTTGCCTCAAGTCTGATAAGCTCTATAG
GCAACCGCCCTATCAACTT--GATGGTAAGGTATTGGCTTACCATGGTTGTAACGGGTAA
CGGGGAATCAGGGTTCGATTCCGGAGAGGACGCCTGAGAAACGGCGTCCACATCTACGGG
TGGCAGCAGGCGCGTAAATT-GCCCAATCTCAATAGAGAGGAGGCGGTGACAATAAATCC
CGATGGCCATGGGGGCAACCCCGGG--CCAATCAGAATAAGTACACATTAAATACCCTAA
CC-AATATAATTGGAGGGCAAGTCTGGTGCCAGCAGCCGCGGTAATTCCAGCTCCAA-TA
GCGTATACTAATGTTGTTGCAGTTAAAA-CGC-TCGTAGCT-----GAACAACTTGATGG
CAAATTC------AGGTCAATGTGGCACGTCCATGGGGGGAAA---CCTCTATGCGGCAG
TGTCC--------TTGAC--CGGC-TAAGCAG--GTAGGTAGTTTGG--CGTGT--GTAG
TAATA-CATGCGTCA-----------GGCGA---TCGACTGTGCATAAA-CCTTGATGCT
CAAGGTAAAGA-CGA-CTC--TAGTCAAAGATACGCAGT-GCATGGCATTGTGGAACAAG
GCGA--TCCGTAGCTTGGTTGGT-GAGCT-------ACGGGTGCAATGATTAATAGGGAG
GAGCGGGGGCCTTCATATTGCCGGGCGAGAGGTGAAATTCGTTGACCCTGGCAAGATGTC
CTACAGCGAAAGCATTGGCCAAGTGCCTCTCCATTAGTCAAGAACGAAAGTTTGGGGATC
AAAGACGATCAGATACCGTCGTAGTCCAAACCATAAACTATGTCGACCAGCGATTAGGTT
CGCCACCTTCTTCGAGAGCAACCTGGCAGCTT-GTGGGAAACCAT-GAGTTCTTGGACTC
TGGGGGGAGTATGGTCGCAAGGCTGAAACTTAAAGGAATTGACGGAAGGGCACACCATGG
AGTGGAG-CCTGCGGCTTAATTT-GACTCAACACGGGAAA-GCTTACCAAGCTCAGATAT
GATAAGGATTGACAGA-CCAA-AAGATCTTTCATGATCTCATAAGTGGTGGTGCATGGCC
GTTCTTAG-TTGGTGGAGTGATTTGTCAGGTCAATTCCGGTAACGGACGAGACCTCGACC
TGCTAACTAGTAGCAGTTGTCTTTTCG--CTCGGCGAGGCGTTTTGGGCGTGGAACTTTG
A--T--AGGGGCAACTCTATC--TTTGGGAAACGAACGAGGCGTAG-TAGTCTGGAGAGG
CAG--CTGTTCCCATATTAAAAT---AATCTTCCTAGAGGTACTTCCA-GCTCTAAGTTG
GAAG-AAGTCCGAGGCAATAACAGGTCTGTGATGCCCTTAAATATCTT-GGGCCGCACGC
GTGCTACAATGTAGGCGCTAATGAGTCGT-TCTTAATATCC--GGCACCGC-AAG---GT
GTCTGGTAATC-CC-CAATCCCCTGCGTGATTGGGATTG-GTCACTGTAACTGT-GACCA
TCAACGAGGAATTCCTTGTATGCGCGGGTCACTATCCCGCGCAGAATCTGTCCCTGCCCT
TTGTACACACCGCCCGTCGCTCCTACCGATCGAACGGTAAGGTAAAGTGGACGGACA---
--------------GCTGTCCT-C-ACG-GATG-----------GC-TGG--AAGT-CC-
A-T-TGAACCT--C----GTC--GTTT
>HQ141521.1_Cavenderia_amphispora_BM9A
TCTAAG-TATAAGCCCTAG-TACGGCTA-GA---CTGCAGACGGCTCATT--ACAA--CG
GTTGC-----AG---CTTACAGAGCATCCGG-GTCGCA---AGACCTTCT-GGATAACCG
CAGTAAATCGGGGCTAATACATACAAA-CGGAGGGGCGGAGCGGGC----AACCGCGAAG
CTTCTGCGATGGACACTTA-GCTATTC--GA-CTGACCC--CT-CAC-GGGAATG--GTT
GGAACCGGTTCATATTGCTAATCGACTTGTGGCTTGCC-CAAGTCTGATAAGCTCTATAG
GCAACCGCCCTATCAACTT--GATGGTAAGGTATTGGCTTACCATGGTTGTAACGGGTAA
CGGGGAATCAGGGTTCGATTCCGGAGAGGACGCCTGAGAAACGGCGTCCACATCTACGGG
TGGCAGCAGGCGCGTAAATT-GCCCAATCTCAATAGAGAGGAGGCGGTGACAATAAATCC
CGATGGCCATGGGGGCAACCCCGGG--CCAATCAGAATAAGTACACATTAAATACCCTAA
CC-AATATAATTGGAGGGCAAGTCTGGTGCCAGCAGCCGCGGTAATTCCAGCTCCAA-TA
GCGTATACTAATGTTGTTGCAGTTAAAA-CGC-TCGTAGCT-----GAACAACTTGATGG
CAAATTC------AGGTCAATGTGGCACGTCCATGGGGGGAAA---CCTTTATGCGGCAG
TGTCC--------TTGAC--CGGC-TAAGCAG--GTAGGTAGTTTGG--CGTGT--GTAG
CAATA-CATGCGTCA-----------GGCGA---TCGACTGTGCATAAA-CCTTGATGCT
CAAGGTAAAGA-CGA-CTC--TAGTCAAAGATACGCAGT-GCATGGCATTGTGGAACAAG
GCGA--TCCGTAGCTTGGTTGGT-GAGCT-------ACGGGTGCAATGATTAATAGGGAG
GAGCGGGGGCCTTCATATTGCCGGGCGAGAGGTGAAATTCGTTGACCCTGGCAAGATGTC
CTACAGCGAAAGCATTGGCCAAGTGCCTCTCCATTAGTCAAGAACGAAAGTTTGGGGATC
AAAGACGATCAGATACCGTCGTAGTCCAAACCATAAACTATGTCGACCAGCGATTAGGTT
CGCCACCTTCTTCGAGAGCAACCTGGCAGCTT-GTGGGAAACCAT-GAGTTCTTGGACTC
TGGGGGGAGTATGGTCGCAAGGCTGAAACTTAAAGGAATTGACGGAAGGGCACACCATGG
AGTGGAG-CCTGCGGCTTAATTT-GACTCAACACGGGAAA-GCTTACCAAGCTCAGATAT
GATAAGGATTGACAGA-CCAA-AAGATCTTTCATGATCTCATAAGTGGTGGTGCATGGCC
GTTCTTAG-TTGGTGGAGTGATTTGTCAGGTCAATTCCGGTAACGGACGAGACCTCGACC
TGCTAACTAGTGGCAGTTGTCTTTTCG--CTCGGCGAGGCGTTTTGGGCGTGGAACTTTG
A--T--AGGGGCAACTCTATC--TTTGGGAAGCGAACGAGGC-TAG-TAGTCTGGAGAGG
CAG--CTGTCCCCATATTAAAAT---AATCTTCCTAGAGGTACTTCCA-GCTCTAAGTTG
GAGG-AAGTCCGAGGCAATAACAGGTCTGTGATGCCCTTAGATATCTT-GGGCCGCACGC
GTGCTACAATGTAGGCGCTAATGAGTCGT-TCTTAATATCCC-GGCACCGCCAAG---GT
GTCTGGTAATC-CC-CAATCCCCTGCGTGATTGGGATTG-GTCACTGTAACTGT-GACCA
TCAACGAGGAATTCCTTGTATGCGCGGGTCACTATCCCGCGCAGAATCTGTCCCTGCCCT
TTGTACACACCGCCCGTCGCTCCTACCGATCGAACGGTAAGGTAAAGTGGACGGACA---
--------------GCTGTCCTTC-ACG-GATG-----------GC-TGG--AAGT-CCC
A-T-TGAACCTT-C----GTC--GTT-
>AM168090.1_Cavenderia_microspora_TNS_C_38
TCTAAG-TATAAGCCCTAG-TACGGCTA-GA---CTGCAGACGGCTCATT--ACAA--CG
GTTGC-----AG---CTTACAGAGCATCCGG-GTCGCA---AGACCTTCT-GGATAACCG

CAGTAAATCGGGGCTAATACATACAAA-CGGAGGGGCGGAGCGGGC----AACCGCGAAG
CTTCTGCGATGGACACTTA-GCTATTC--GA-CTGACCC--CT-CAC-GGGAATG--GTT
GGAACCGGTTCATATTGCTAATCGACTTGTGGCTTGCCTCAAGTCTGATAAGCTCTATAG
GCAACCGCCCTATCAACTT--GATGGTAAGGTATTGGCTTACCATGGTTGTAACGGGTAA
CGGGGAATCAGGGTTCGATTCCGGAGAGGACGCCTGAGAAACGGCGTCCACATCTACGGG
TGGCAGCAGGCGCGTAAATT-GCCCAATCTCAATAGAGAGGAGGCGGTGACAATAAATCC
CGATGGCCATGGGGGCAACCCCGGG--CCAATCAGAATAAGTACACATTAAATACCCTAA
CC-AATATAATTGGAGGGCAAGTCTGGTGCCAGCAGCCGCGGTAATTCCAGCTCCAA-TA
GCGTATACTAATGTTGTTGCAGTTAAAA-CGC-TCGTAGCT-----GAACAACTTGATGG
CAAATTC------AGGTCAATGTGGCACGTCCATGGGGGGAAA---CCTTTATGCGGCAG
TGTCC--------TTGAC--CGGC-TAAGCAG--GTAGGTAGTTTGG--CG-GT--GTAG
CAATA-CAT-CGTCA-----------GGCGA---TCGACTGTGCATAAA-CCTTGATGCT
CAAGGTAAAGA-CGA-TTC--TAGTCAAAGATACGCAGT-GCATGGCATTGTGGAACAAG
GCGA--TCCGTAGCTTGGTTGGT-GAGCT-------ACGGGTGCAATGATTAATAGGGAG
GAGCGGGGGCCTTCATATTGCCGGGCGAGAGGTGAAATTCGTTGACCCTGGCAAGATGTC
CTACAGCGAAAGCATTGGCCAAGTGCCTCTCCATTAGTCAAGAACGAAAGTTTGGGGATC
AAAGACGATCAGATACCGTCGTAGTCCAAACCATAAACTATGTCGACCAGCGATTAGGTT
CGCCACCTTCTTCGAGAGCAACCTGGCAGCTT-GTGGGAAACCAT-GAGTTCTTGGACTC
TGGGGGGAGTATGGTCGCAAGGCTGAAACTTAAAGGAATTGACGGAAGGGCACACCATGG
AGTGGAG-CCTGCGGCTTAATTT-GACTCAACACGGGAAA-GCTTACCAAGCTCAGATAT
GATAAGGATTGACAGA-CCAA-AAGATCTTTCATGATCTCATAAGTGGTGGTGCATGGCC
GTTCTTAG-TTGGTGGAGTGATTTGTCAGGTCAATTCCGGTAACGGACGAGACCTCGACC
TGCTAACTAGTGGCAGTTGTCTTTTCG--CTCGGCGAGGCGTTTTGGGCGTGGAACTTTG
G--T--AGGGGCAACTCTATC--TTTGGGAAGCGAACGAGGCGTAG-TAGTCTGGAGAGG
CAG--CTGTCCCCATATTAAAAT---AATCTTCCTAGAGGTACTTCCA-GCTCTAAGTTG
GAGG-AAGTCCGAGGCAATAACAGGTCTGTGATGCCCTTAGATATCTT-GGGCCGCACGC
GTGCTACAATGTAGGCGCTAATGAGTCGT-TCTTAATATCC--GGCACCGC-AAG---GT
GTCTGGTAATC-CC-CAATCCCCTGCGTGATTGGGATTG-GTCACTGTAACTGT-GACCA
TCAACGAGGAATTCCTTGTATGCGCGGGTCACTATCCCGCGCAGAATCTGTCCCTGCCCT
TTGTACACACCGCCCGTCGCTCCTACCGATCGAACGGTAAGGTAAAGTGGACGGACA---
--------------GCTGTCCT-C-ACG-GATG-----------GC-TGG--AAGT-CC-
A-T-TGAACCTG-C----GTC--GTTT
>HQ141520.1_Cavenderia_boomerangispora_K26B
TCTAAG-TATAAGCCCTTG-TACGGCTA-GA---CTGCAGACGGCTCATT--ACAA--CG
GTTGT-----AG---CTTACAGAGCATCCGG-G-CGCA---AG-CCTTCT-GGATAACCG
CAGTAAATCGGGGCTAATACATACAAA-CGGAGGGGTGGAGCGGGC----AACCGCAAAG
CTTCTGCGATGGACACTTA-GCTATTC--GA-CTAACCC---T--AC-GGGTCAG--GTT
GGAACCGGTTCATATTGCCAATCGACT-ATGGCTTGCCATA-GTCTGATAAGCTCTATAG
ACAACCGCCCTATCAACTT--GATGGTAAGGTATTGGCTTACCATGGTTGTAACGGGTAA
CGGGGAATCAGGGTTCGATTCCGGAGAGGACGCCTGAGAAACGGCGTCCACATCTACGGG
TGGCAGCAGGCGCGTAAATT-GCCCAATCTCAATAGAGAGGAGGCGGCGACAATAAATCC
CGATGGCTATGGGGGCAACCCCGGG--CCAATCAGAATAAGTACACAATAAATACCCTAA
CC-AATATAATTGGAGGGCAAGTCTGGTGCCAGCAGCCGCGGTAATTCCAGCTCCAA-TA
GCATATACTAACGTTGTTGCAGTTAAAA-CGC-TCGTAGCT-----GAACAACTTGATGG
CAAATTC------AGGGCAGTGTCAAGAGTAC-TGGGGTCAAA---CCTT--GGATACAT
CTTGC--------CTGTC--CGGC-CAAGCAA--GTAGGTAGTTTGG--AGTA---GGGG
CAACT-C-TGCTTCA-----------GGCGA---TCGACTGTGCATAAA-CCTTGATGCT
CAAGGTAAAGA-CGA-CTC--TAGTCAAAGATACGCAGT-GCATGGCATTGTGGAACAAG
GCGA--TCCGTAGCTTAGTTAGT-GGGCT-------GCGGGTGCAATGATTAATAGGGAG
GAGCGGGGGCCTTCATATTGCCGGGCGAGAGGTGAAATTCGTTGACCCTGGCAAGATGTC
CTACAGCGAAAGCATTGGCCAAGTGCCTCTCCATTAGTCAAGAACGAAAGTTTGGGGATC
AAAGACGATCAGATACCGTCGTAGTCCAAACCATAAACGATGTCGACCAGCGATTAGGTT
CGCCACCTTCTTCGAGAGCAACCTGGCAGCTT-GTGGGAAACCAT-GAGTTCTTGGACTC
TGGGGGGAGTATGGTCGCAAGGCTGAAACTTAAAGGAATTGACGGAAGGGCACACCATGG
AGTGGAG-CCTGCGGCTTAATTT-GACTCAACACGGGAAA-GCTTACCAAGCTCAGATAT
GATAAGGATTGACAGA-CCAA-TAGATCTTTCATGATCT---------------------

>AM168085.1_Cavenderia_exigua_TNS_C_199
TCTAAG-TATAAGCCCTTG-TACGGCTA-GA---CTGCAGACGGCTCATT--ACAA--CG
GTTGT-----AG---CTTACAGAGCATCCGG-G-CGCA---AG-CCTTCT-GGATAACCG
CAGTAAATCGGGGCTAATACATACAAA-CGGAGGGGTGGAGCGGGC----AACCGCAAAG
CTTCTGCGATGGACACTTA-GCTATTC--GA-CTAACCC--TT---C-GGGTCAG--GTT
GGAACCGGTTCATATTGCCAATCGACT-ATGGCTTGCCATA-GTCTGATAAGCTCTATAG

ACAACCGCCCTATCAACTT--GATGGTAAGGTATTGGCTTACCATGGTTGTAACGGGTAA
CGGGGAATCAGGGTTCGATTCCGGAGAGGACGCCTGAGAAACGGCGTCCACATCTACGGG
TGGCAGCAGGCGCGTAAATT-GCCCAATCTCAATAGAGAGGAGGCGGCGACAATAAATCC
CGATGGCTATGGGGGCAACCCCGGG--CCAATCAGAATAAGTACACAATAAATACCCTAA
CC-AATATAATTGGAGGGCAGGTCTGGTGCCAGCAGCCGCGGTAATTCCAGCTCCAA-TA
GCATATACTAACGTTGTTGCAGTTAAAA-CGC-TCGTAGCT-----GAACAACTTGATGG
CAAATTC------AGGGCT-TGTCACGAGTAC-CGCCTTAAAA---C-----GGATACAT
CGTGC--------TTGTC--CGGC-CAAGCAA--GTAGGTAGTTTGA--TGT----GAGG
CAACT-C--GCGTCA-----------GGCGA---TCGACTGTGCATAAA-CCTTGATGCT
TAAGGTAAAGA-CGA-TTC--TAGTCAAAGATACGCAGT-GCATGGCATTGTGGAACAAG
GCGA--TCCGTAGCTTAGTTAGT-GGGCT-------GCGGGTGCAATGATTAATAGGGAG
GAGCGGGGGCCTTCATATTGCCGGGCGAGAGGTGAAATTCGTTGACCCTGGCAAGATGTC
CTACAGCGAAAGCATTGGCCAAGTGCCTCTCCATTAGTCAAGAACGAAAGTTTGGGGATC
AAAGACGATCAGATACCGTCGTAGTCCAGACCATAAACGATGTCGACCAGCGATTAGGTT
CGCCACCTTCTTCGAGAGCAACCTGGCAGCTT-GTGGGAAACCAT-GAGTTCTTGGACTC
TGGGGGGAGTATGGTCGCAAGGCTGAAACTTAAAGGAATTGACGGAAGGGCACACCATGG
AGTGGAG-CCTGCGGCTTAATTT-GACTCAACACGGGAAA-GCTTACCAAGCTCAGATAT
GATAAGGATTGACAGA-CCAA-TAGATCTTTCATGATCTCATAAGTGGTGGTGCATGGCC
GTTCTTAG-TTGGTGGAGTGATTTGTCAGGTCAATTCCGGTAACGGACGAGACCTCGACC
TGCTAACTAGTGGCAGTTGTCTTTTCG--CTCGGCGAGGCGTCATTGGCATACGACTTTG
G--T--AGGGGCAACTCTATC--TTGGAAGTGTGACAGCGGCGTAG-TAGTCTGGAGAGG
CAG--CTGTCCCCAA---TAATT---AATCTTCCTAGAGGTACTTCCA-GCTCTAAGCTG
GAGG-AAGTCCGAGGCAATAACAGGTCTGTGATGCCCTTAGATATCTT-GGGCCGCACGC
GTGCTACAATGTAGGCGCTAATATGATAT-CCCAATTACCC--GGCACCGTCAAG---GT
GTC-GGTAATC-TC-CAATCACCTGCGTGATTGGGCTTG-GTCACTGTAACTGT-GACCA
TTAACGAGGAATTCCTTGTATACGCGAGTCACTATCTCGCATAGAATCTGTCCCTGCCCT
TTGTACACACCGCCCGTCGCTCCTACCGATCGAACGGTAAGGTAAAGTGGACAGA-----
---------CCG--CC-GCCCT-C-ATC-GGTA-----------GC-TGG--AAGT-CC-
A-T-TGAACCT--C-----TTC-GTTT
>AM168110.1_Acytostelium_subglobosum_LB1
TCTAAG-TATAAACCTCTA-TATGGTGAAA----CTGCAGACGGCTCA-TT-ACAA--CA
---GT----GATAAACTA-CAAGACTTTCGC-GCTTC-----GGCGTTAT-GGATAACCG
CAGTAAATCGGGGCTAATACATATAAA-CG-AAAGA-CGAGCAAG----CAATTGCG-AG
TTCTTGCGATTTTT----A-GCTACTA---A---A-ATCGACCCTTTT--AGGTCGTGTT
GAAACCGAAAAATATTGCTGATCGAGA--------ATT-T---CTCGACAAGTCTTATGT
GTCACTGCCCTATCAACT--CGATGGTACGGTATTGGCCTACCATGGTTGCAACGGGTAA
CGGGGAATTAGGGTTCGATTCCGGAGAGGGAGCCTGAGAAATGGCTGCCACTTCTACGGA
AGGCAGCAGGCGCGCAAATT-ACTCAATTCCGATAC-GGAGAAGTAGTGACAATAAATAC
AAATGCTTTTCAATTT-TTTTGAGA--GCAATTTGAATAAGTACAACTTAAATCGCTTAG
CT-AAAGTGATTGGAGGGCAAGTCTGGTGCCAGCAGCCGCGGTAATTCCAGCTCCAA-TA
GCGTATACTAAATTTGTTGCAGTTAAAA-CGC-TCGTAGTTAAAGTAAAGGTTACTTG-G
AT-AAAC----AAGAATAATTTTTTTTA--GAATTATT------T-----AACGAATTT-
----TTAATTATTTTCAT-AGTTAAATTTTCA-CCAAGTTC-CTTATTTTTGG-ACT---
-----TAAAAATTTATT-----TTTTT-GTCTAGTCTACTGTGAGAAAA-TTGTAGTGTT
TAAAGCA--AG-C----T----ATCAGCTTGATCAATGCAGCATGGTATAGAAAAATATG
ACAC---TAAGTATTTTGTTGGTTT-AATA----C-TCTAGTGTAATGATTAATAGGGAA
TGGCGGGGCCGTTCGTATTGATGGGCGAGAGGTGAAATTCGTTGACCCTATCAAGACGTA
CAACAGCGAAAGCATTCGGCAAGTATTTCTCCATTAATCAAGAACGAAAGTTTGGGGATC
GAAGACGATCAGATACCGTCGTAGTCCAAACCATAAACAATGTCGACCAGGGATTGGACG
GAATTT-TTTTAAAAAACTCGCTCAGAACCTT-GTGAGAAATCAT-GAGTGTTTGGACTC
TGGGGGGAGTATGGTCGCAAGTCTGAAACTTAAAGGAATTGACGGAAGGGCACACAATGG
AGTGGAA-CCTGCGGCTTAATTT-GACTCAACTCGGGAAA-ACTTACCAAGCTCAGATAT
AATAAGGATTGACAGA-CTAA-AAGATCTTTCATGATTGTATAAGTGGTGGTGCATGGTC
GTTCTTAG-TTGGTGGAGTGATTTGTCTGGTCAATTCCGATAACGGACGAGACGTCTACT
TGCTAACTAGTAAAATTAATTTTTTCGC-TCGGG-GATAGTTAGT-TTGTATTGATCTTT
ATCA--T-CGCAA--GGT-G---GTAATTTTCGTATAGATTGATGTGTACCTTGGAAAAA
TAA--ATTTTTTCATAAACATTA---AA-CTTCTTAGAAGGACTACCT-ACCTCAAGTGG
GGGG-AAGTCGGACGCAATAACAGGTCTGTGATGCCCTTAGATACCTT-GGGCTGCACGC
GCGTTACAATGTAGAAAAGAAAAAGGC----TTCCC-------GGAGCCGT-AAG---G-
CTCTGGTAATCATATGAATTTTCTACGTAATAGGGATTG-ATCTTTGTAATTATCGATCA
TAAACGAGGAATTCCTTGTAAGCGTAAATCATTACTTTACGCTGAATATGTCCCTGCCCT
TTGTACACACCGCCCGTCGCTCCTACCGATCGAACGATTAGGTAAAATTGACGGACTT--
-TGACATA-CTA--G-----AA-A-TAG-CTTGT--------TTTT-G-A--AAG--TTA
T-T-TAAATCT--C----ATT--GTTT
>HQ141510.1_Acytostelium_amazonicum_landolt_X
TCTAAG-TATAAACCTCTA-TATGGTGAAC----CTGCAGAAGGATCAACT-ACAA--CA
--TGT----GATAAACTA-CAAGACTTTCGC-GCTTC-----GGCGTTAT-GGATAACCG
CAGTAAATCGGGGCTAATACATATAAA-CG-AAAGA-CGAGCAAG----CAATTGCG-AG
TTCTTGCGATTTTT----A-GCTACTA---A---A-ATCGACCCTTTT--AGGTCGTGTT
GAAACCGAAAAATATTGCTGATCGAGA--------ATT-T---CTCGACAAGTCTTATGT
GTCACTGCCCTATCAACT--CGATGGTACGGTATTGGCCTACCATGGTTGCAACGGGTAA
CGGGGAATTAGGGTTCGATTCCGGAGAGGGAGCCTGAGAAATGGCTGCCACTTCTACGGA
AGGCAGCAGGCGCGCAAATT-ACTCAATTCCGATAC-GGAGAAGTAGTGACAATAAATAC

AAATGCTTTTCAATTT-TTTTGAGA--GCAATTTGAATAAGTACAACTTAAATCGCTTAG
CT-AAAGTGATTGGAGGGCAAGTCTGGTGCCAGCAGCCGCGGTAATTCCAGCTCCAA-TA
GCGTATACTAAATTTGTTGCAGTTAAAA-CGC-TCGTAGTTAAAGTAAAGGTTACTTG-G
AT-AAAC----AAGAATAATTTTTTTTA--GAATTATT------T-----AACGAATTT-
----TTAATTATTTTCAT-AGTTAAATTTTCA-CCAAGTTC-CTTATTTTTGG-ACT---
-----TAAAAATTTATT-----TTTTT-GTCTAGTCTACTGTGAGAAAA-TTGTAGTGTT
TAAAGCA--AG-C----T----ATCAGCTTGATCAATGCAGCATGGTATAGAAAAATATG
ACAC---TAAGTATTTTGTTGGTTT-AATA----C-TCTAGTGTAATGATTAATAGGGAA
TGGCGGGGCCGTTCGTATTGATGGGCGAGAGGTGAAATTCGTTGACCCTATCAAGACGTA
CAACAGCGAAAGCATTCGGCAAGTATTTCTCCATTAATCAAGAACGAAAGTTTGGGGATC
GAAGACGATCAGATACCGTCGTAGTCCAAACCATAAACAATGTCGACCAGGGATTGGACG
GAATTT-TTTTAAAAAACTCGCTCAGAACCTT-GTGAGAAATCAT-GAGTGTTTGGACTC
TGGGGGGAGTATGGTCGCAAGTCTGAAACTTAAAGGAATTGACGGAAGGGCACACAATGG
AGTGGAA-CCTGCGGCTTAATTT-GACTCAACTCGGGAAA-ACTTACCAAGCTCAGATAT
AATAAGGATTGACAGA-CTAA-AAGATCTTTCATGATTGTATAAGTGGTGGTGCATGGTC
GTTCTTAG-TTGGTGGAGTGATTTGTCTGGTCAATTCCGATAACGGACGAGACGTCTACT
TGCTAACTAGTAAAATTAATTTTTTCGC-TCGGG-GATAGTTAGT-TTGTATTGATCTTT
ATCA--T-CGCAA--GGT-G---GTAATTTTCGTATAGATTGATGTGTACCTTGGAAAAA
TAA--ATTTTTTCATAAACATTA---AA-CTTCTTAGAAGGACTACCT-ACCTCAAGTGG
GGGG-AAGTCGGACGCAATAACAGGTCTGTGATGCCCTTAGATACCTT-GGGCTGCACGC
GCGTTACAATGTAGAAAAGAAAAAGGC----TTCCC-------GGAGCCGT-AAG---G-
CTCTGGTAATCATATGAATTTTCTACGTAATAGGGATTG-ATCTTTGTAATTATCGATCA
TAAACGAGGAATTCCTTGTAAGCGTAAATCATTACTTTACGCTGAATATGTCCCTGCCCT
TTGTACACACCGCCCGTCGCTCCTACCGATCGAACGATTAGGTAAAATTGACGGACTT--
-TGAC-TAACTC--A-----TG-A-GAT-ATGTAT--------GCCTGGGTGAACC--TG
C-AG-AAGGAT--C----A--C---C-
>HQ141513.1_Acytostelium_magnisorum_08A
TCTAAG-TATAAACCTCTA-TATGGTGAAC----CTGCAGAAGGATCAACT-ACGAG-CA
--TGT----GATAAACTA-CAAGACTTTCGC-GCTTC-----GGCGTTAT-GGATAACCG
CAGTAAATCGGGGCTAATACATATAAA-CG-AAAGA-CGAGCAAG----CAATTGCG-AG
TTCTTGCGATTTTT----A-GCTACTA---A---A-ATCGACCCTTTT--AGGTCGTGTT
GAAACCGAAAAATATTGCTGATCGAGA--------ATT-T---CTCGACAAGTCTTATGT
GTCACTGCCCTATCAACT--CGATGGTACGGTATTGGCCTACCATGGTTGCAACGGGTAA
CGGGGAATTAGGGTTCGATTCCGGAGAGGGAGCCTGAGAAATGGCTGCCACTTATACGGA
AGGCAGCAGGCGCGCAAATT-ACTCAATTCCGATAC-GGAGAAGTAGTGACAATAAATAC
AAATGCTTTTCAATTT-TTTTGAGA--GCAATTTGAATAAGTACAACTTAAATCGCTTAG
CT-AAAGTGATTGGAGGGCAAGTCTGGTGCCAGCAGCCGCGGTAATTCCAGCTCCAA-TA
GCGTATACTAAATTTGTTGCAGTTAAAA-CGC-TCGTAGTTAAAGTAAAGGTTACTTG-G
AT-AAAC----AAGAATAATTTTTTTTA--GAATTATT------T-----AACGAATTT-
----TTAATTATTTTCAT-AGTTAAATTTTCA-CCAAGTTC-CTTATTTTTGG-ACT---
-----TAAAAATTTATT-----TTTTT-GTCTAGTCTACTGTGAGAAAA-TTGTAGTGTT
TAAAGCA--AG-C----T----ATCAGCTTGATCAATGCAGCATGGTATAGAAAAATATG
ACAC---TAAGTATTTTGTTGGTTT-AATA----C-TCTAGTGTAATGATTAATAGGGAA
TGGCGGGGCCGTTCGTATTGATGGGCGAGAGGTGAAATTCGTTGACCCTATCAAGACGTA
CAACAGCGAAAGCATTCGGCAAGTATTTCTCCATTAATCAAGAACGAAAGTTTGGGGATC
GAAGACGATCAGATACCGTCGTAGTCCAAACCATAAACAATGTCGACCAGGGATTGGACG
GAATTT-TTTTAAAAAACTCGCTCAGAACCTT-GTGAGAAATCAT-GAGTGTTTGGACTC
TGGGGGGAGTATGGTCGCAAGTCTGAAACTTAAAGGAATTGACGGAAGGGCACACAATGG
AGTGGAA-CCTGCGGCTTAATTT-GACTCAACTCGGGAAA-ACTTACCAAGCTCAGATAT
AATAAGGATTGACAGA-CTAA-AAGATCTTTCATGATTGTATAAGTGGTGGTGCATGGTC
GTTCTTAG-TTGGTGGAGTGATTTTGTCTGGTCAATTCCGATAACGGACGAGACGTCTACT
TGCTAACTAGTAAAATTAATTTTTTCGC-TCGGG-GATAGTTAGT-TTGTATTGATCTTT
ATCA--T-CGCAA--TGT-G---GTAATTTTCGTATAGATTGATGTGTACCTTGGAAAAA
TAA--ATTTTTTCATAAACATTA---AA-CTTCTTAGAATGACTACCT-ACCTCAAGTGG
GGGG-AAGTCGGACGCAATAACAGGTCTGTGATGCCCTTAGATACCTT-GGGCTGCACGC
GCGTTACAATGTATAAAAGAAAAAGGC----TTCCC-------GGAGCCGT-AAG---G-
CTCTGGTAATCATATGAATTTTCTACGTAATAGGGATTG-ATCTTTGTAATTATCGATCA
TAAACGAGGAATTCCTTGTAAGCGTAAATCATTACTTTACGCTGAATATGTCCCTGCCCT
TTGTACACACCGCCCGTCGCTCCTACCGATCGAACGATTAGGTAAAATTGACGGACGG--
-TGA---ACCTG--C-----AG-A-AGG-AT---C------ATTT--GACT-AACCA-TG
--AGTT--GAT--C-------CTGCC-
>HQ141511.1_Acytostelium_amazonicum_HN1B1
TCTAAG-TATAAACCTTTA-TACGGTGAAC----CTGCAGAAGGATCAAT--ACAA--CA
--TGT----GATAAACTA-CAAGACTTTCGC-GCTTC-----GGCGTTTT-GGATAACCG
CAGTAAATCGGGGCTAATACATATAAA-CG-AAAGG-CGAGCAAG----TAATTGCG-AG
TCCTTGCGATTGTT----A-GCCATTT---T---A-ACCGACCCTCTTG-AGGTTGTGTT
GAACCCGAACAATATTGCTGATCGAGA--------ATT-TATTCTCGACAAGTCTTATGT
GTCACTGCCCTATCAACT--CGATGGTACGGTATTGGCCTACCATGGTTGCAACGGGTAA
CGGGGAATCAGGGTTCGATTCCGGAGAGGGAGCCTGAGAAATGGCTGCCACTTCTACGGA
AGGCAGCAGGCGCGCAAATT-ACTCAATCCCAATAC-GGGGAAGTAGTGACAATAAATAC
AAATGCCTTTCCATTT-TATGGAGG--GCAATTTGAATAAGTACAATCTAAATCGCTTAG
CC-AAAGTGATTGGAGGGCAAGTCTGGTGCCAGCAGCCGCGGTAATTCCAGCTCCAA-TA
GCGTATACTAAATTTGTTGCAGTTAAAA-CGC-TCGTAGTTGAAGTAGAAATTATTTG-G

AG-AA-T-----AGAAGGA-TCTTTTTAAAGGTCACTT------T-----AAAG-ACAC-
----TTTTCCACTTA-GT-GGTCAAGTTGTCA-CCAAATAT-TTCTTTAAGGGCAAT---
-----GGAATTTTTATT-----CATTT-GCCTTGTCTACTGTGAGAAAA-TTGCAGTGTT
CAAAGCA--AG-C----G----TAAAGCTTGATCAATGCAGCATGGTATAGAGAAACACG
ACAC---TAAATATTTGTTGGTT--AATA-----TTCTAGTGTAATGGTTAATAGGGAA
TGGCGGGGCCGTTCGTATTGATGGGCGAGAGGTGAAATTCGTTGACCCTATCAAGACGTA
CAACAGCGAAAGCATTCGGCAAGTAATTCTCCATTAATCAAGAACGAAAGTTTGGGGATC
GAAGACGATCAGATACCGTCGTAGTCCAAACTATAAACTATGTCGACCAGGGATTGGACG
GATCCT-TTTTAAAAAACCCGCTCAGAACCTT-GTGGGAAACCAT-GAGTGTTTGGACTC
TGGGGGGAGTATGGTCGCAAGTCTGAAACTTAAAGGAATTGACGGAAGGGCACACAATGG
AGTGGAA-CCTGCGGCTTAATTT-GACTCAACTCGGGAAA-ACTTACCAAGCTCAGATAT
AATAAGGATTGACAGA-CTAA-AAGATCTTTCATGATTGTATAAGTGGTGGTGCATGGTC
GTTCTTAG-TTGGTGGAGTGATTTGTCTGGTCAATTCCGATAACGGACGAGACGTCTACT
TGCTAACTAGTGTAAATTATTATGCCGA-ACGAGAGATAGCTAGT-TTGTATTGATTTTT
ATTG--A-CGCAA--G-TCG---GTAGACTTCGTATAGATTAGTGTGTATCTTGGTAAAA
TAA--TTTATATCATAAACTTTA---AA-CTTCTTAGAAGGACTACCT-ACCTCAAGTGG
GGGG-AAGTCGGACGCAATAACAGGTCTGTGATGCCCTTAGATACCTT-GGGCTGCACGC
GCGTTACAATGTAGAGTGGAAAAAGGC----TTCCC-------GTGACCGG-AAG---GT
C-ATGGTAATCATTTGAATACTCTACGTAATAGGGATTG-ATCTTTGTAATTATCGATCA
TAAACGAGGAATTCCTTGTAAGCGTAAATCATTACTTTACGCTGAATATGTCCCTGCCCT
TTGTACACACCGCCCGTCGCTCCTACCGATCGAACGATTAGGTAAAATTGACGGACGG--
-TGA---ACCTG--C-----AG-A-AGG-AT---C------AAAT--GCCT-AACC--TG
CGA-TT--GAT--C-------CTGCCG
>HQ141512.1_Acytostelium_leptosomum_212rjb
TCTAAG-TATAAACTTTAA-TACGGTGAAC----CTGCAGAAGGATCAAT--ACAA--CA
--TGT----GATAAAGTA-CAGAACTTTCGC-GCGTA----AG-CGTCTTTGGATAACCG
CAGTAAATCGGGGCTAATACATATAAA-CG-AAAGA-TTAATAGTTT--CTACTGTT-GA
T-TTTGCGATTGTT----A-GTCATTT-CAA---A-ACTAGCCTCTTCGGAGTTAGTGGT
GAATCCGAACAATATTGCTGATCGA-A--------ATT-TATTT-CGACGAGTTCTATCT
GTCACTGCCCTATCAACTTTCGATGGTACGGTATTGGCCTACCATGGTTGTAACGGGTAA
CGGGGAATCAGGGTTCGATTCCGGAGAGGGCGCCTGAGAAATGGCGACCACTTCTACGGA
AGGCAGCAGGCGCGCAAATT-ACTCAATCCCAATAC-GGGGAAGTAGTGACAATAAATAC
TAATTTCTTTCCATTT-TATGGAGG--AAAATTGGAATAAGTACAACGTAAATAGCTTAA
CC-AAAGTAATTGGAGGGCAAGTCTGGTGCCAGCAGCCGCGGTAATTCCAGCTCCAA-TA
GCGTATACTAAATTTGTTGCAGTTAAAA-AGC-TCGTAGTTCAGATAAAGGTTGTTCG-G
GT-CAAG-----------------------GTG-TCTTTA----T-AAAATAAGCATCTG
ATT-CTA-TCTAATTTATTAGTT-TAGTATCA-CCGACAAC-TTTATTTAGGG-TCT---
---GTTATCAATTTATT--GGTGATTT-GATCTATCCACTGTGAGAAAA-TTGTAGTGTT
TAAAGCA--AG-C---GT----TTCTGCTTGTTCAATGCAGCATGGTATGGTAAAATAGG
ACAC---TTAACAATT-GTTGGTTT-TTT-----GTTGAAGTGTAATGACTAATAGGGAA
GGGCGGGATCGTACATATTGATGGGCGAGAGGTGAAATTCGTTGACCCTATCAAGATGCA
CTACAGCGAAAGCATTCGATAAGTACTTCTCCATTAATCAAGAACGAAAGTTTGGGGATC
GAAGACGATCAGATACCGTCGTAGTCCAAACTATAAACTATGTCGACCAGGGATCGGATA
GATTAA-ATTTCTAACTCTAT-TCGGAACCTT-GTGAGAAATCAT-GAGTGTTTGGACTC
TGGGGGGAGTATGGTCGCAAGGCTGAAACTTAAAGGAATTGACGGAAGGGCACACAATGG
AGTGGAG-CCTGCGGCTTAATTT-GACTCAACTCGGGAAA-ACTTACCAAGCTCAGATAT
AATAAGGATTGACAGA-CTAA-AAGATCTTTCATGATTGTATAAGTGGTGGTGCATGGTC
GTTCTTAG-TTGGTGGAGTGATTTGTCTGGTCAATTCCGATAACGGACGAGACGTCTACT
TACTAAATAGTAGGATTTAT-GGGTCAATATGTGGGATAGGTCTT-AGGGGTTTGTA--T
ATAT--TTC-CAAAAGATTT---GTATGCTACCACT--ATGGCTGTGTAGCACGACTCTT
TAA--ATTCGAG-TAAAAAACTA---AA-CTTCTTAGAAGGACTACCC-ACCTCAAGTGG
GGGG-AAGTCGGACGCAATAACAGGTCTGTGATGCCCTTAGATACCTT-GGGCTGCACGC
GCGCTACAATGTAGAGATGAAAAAGGT----TTCCG-------GGTACCGG-AAG---GT
ATC-GGTAATCATTTGAATACTCTACGTGATAGGGATTG-ATCTTTGTAATTATCGATCA
TCAACGAGGAATTCCTTGTAAGCGTAAGTCATTACCTTACGCTGAATAGGTCCCTGCCCT
TTGTACACACCGCCCGTCGCTCCTACCGATCGAACGATTAGGTAAAACTGACGGACGG--
-TGA---ACCTG--C-----AG-A-AGG-ATCATT-------AGTC-AAC---AC---TC
G-TGTT--GAT--C-------CTGC-T
>AM168109.1_Acytostelium_longisorophorum_DB10A
TCTAAG-TATAAACTTTTA-TACGGTGAAA----CTGCAGACGGCTCATT--ACAA--CA
---GT----GATAAACTA-CAGAACTTTCGA-GCGT--------CTTTTT-GGATAACCG
CAGTAAATCGGGGCTAATACATATAAA-CG-GAAGG-CGATTG-----------AAG-AG
TCCTTGCGATTACT----A-GC---TT---A---A-ACCAACCT-------------GTT
GAAACCGAATAATATTGCTGATC-AGA--------A-----ATTCTGACAAGTTCTATGT
GTCACTGCCCTATCAACTT--GATGGTACGGTATTGGCCTACCATGGTTGTAACGGGTAA
CGGGGAATCAGGGTTCGATTCCGGAGAGGGAGCCTGAGAAATGGCTACCACTTCTACGGA
AGGCAGCAGGCGCGCAAATT-ACTCAATCCCAATAC-GGGGAA-TAGTGACAATAAATAC
TAATGTTCTTCC--TT-TATGGAGA--ACAATTGGAATAAGTACAACGTAAATAGCTTAG
CA-AAAGTAATGGGAGGGCAAGTCTGGTG------------------------------

-----------------------------TCAATCCACTGTGAGAAAA-TTGTGGTGTT

TAAAGCA--GG-C---GT----TC-AGTCTGATCAATGCAGCATGGTATGGTAAAATATG
ACAC---TT--CATTT-GTTGGTT---------------AGTGTAATGACTAATAGGGAA
GGGCGGGGCCGTTCATATTGATGGGCGAGAGGTGAAATTCGTTGACCCTATCAAGATGCA
CTACAGCGAAAGCATTCGGCAAGTACTTCTCCATTAATCAAGAACGAAAGTTTGGGGTTC
GAAGACGATCAGATACCGTCGTAGTCCAAACCATAAATGATGTCGATCAGGGATCGG-TA
G-----------TAAATTCTTATCGGAACCTT-GTGAGAAATCAT-GAATGTTTGGACTC
TGGGGGGAGTATGGTCGCAAGGCTGAAACTTAAAGGAATTGACGGAAGGGCACACAATGG
AGTGGAA-CCTGCGGCTTAATTT-GACTCAACTCGGGAAA-ACTTACCAAGCTCAGATAT
AATAAGGATTGACAGA-CTAA-AAGATCTTTCATGATTGTATAAGTGGTGGTGCATGGTC
GTTCTTAG-TTGGTGGAGTGATTTGTCTGGTCAATTCCGATAACGGACGAGACGTCTACT
TGCTAACTAGTAGGATTTATTTTTCCAAAT-GGGGGATAGTTAGT-TGGTGGT-------
----------------GTGG---GTA---TTCCATT-GATTAGTGTGTACTCTGGATTGA
TAG--AT---------------------CTTCTTAGAAGGACTACCT-ACCTCAAGTAG
GGGG-AAATCGGACGCAATAACAGGTCTGTGATGCCCTTAGATACCTT-GGGCTGCACGC
GCGCTACAATGCAGCATAGAAAAAGGC--------C-------GTTGCTGG-AAA---G-
CAATGGTAATCAAA--AATATACTACGTAATAGGGATTG-ATCTTTGTAATTATCGATCA
TAAACGAGGAATTCCTTGTAAGCGTAATTCATTACATTACGCTGAATATGTCCCTGCCCT
TTGTACACACCGCCCGTCGCTCCTACCGATCGAATGATTGGGTAAAACTGACAGAT----
---TA-GAAG--TTA
T-T-TAAATCT--C----ATT--GTTT
>AM168115.1_Acytostelium_anastomosans_PP1
TCTAAG-TATAAACTTTTA-TACGGTGAAA----CTGCAGACGGCTCATT--ACAA--CA
---GT----GATAAACTA-CAGAACTTTCGA-GCGTA----AG-CTTTTT-GGATAACCG
CAGTAAATCGGGGCTAATACATATAAA-CG-GAAGG-CGATTGGG----CAACCAAG-AG
TCCTTGCGATTACT----A-GC---TT-C-A---A-ACCAACCTCTATG-AGATTGAGTT
GAAACCGAATAATATTGCTGATC-AGA--------ATT-TAATTCTGACAAGTTCTATGT
GTCACTGCCCTATCAACTTTCGATGGTACGGTATTGGCCTACCATGGTTGTAACGGGTAA
CGGGGAATCAGGGTTCGATTCCGGAGAGGGAGCCTGAGAAATGGCTACCACTTCTACGGA
AGGCAGCAGGCGCGCAAATT-ACTCAATCCCAATAC-GGGGAAGTAGTGACAATAAATAC
TAATGTTCTTCCATTT-TATGGAGA--ACAATTGGAATAAGTACAACGTAAATAGCTTAG
CA-AAAGTAATTGGAGGGCAAGTCTGGTGCCAGCAGCCGCGGTAATTCCAGCTCCAA-TA
GCGTATACTAAATTTGTTGCAGTTAAAA-AGC-TCGTAGTTTAGGCAGAGGTCATTCG-G
AT-TCAT------TAATTTCACCTTCAAAAGTG-ACTT------T-----AAAGGCTAT-
ATCACGCTTCGGTTGTGTTTAT-----GGTCA-CCGATGACTTGTATTATGA--CGT-TA
TTTGTTAGTTTACTAGCT-TATAATC---GTCAATCCACTGTGAGAAAA-TTGTGGTGTT
TAAAGCA--GG-C---GT----TCTAGTCTGATCAATGCAGCATGGTATGGTAAAATATG
ACAC---TTAACATTT-GTTGGTTT-ACT---TTGTTATAGTGTAATGACTAATAGGGAA
GGGCGGGGCCGTTCATATTGATGGGCGAGAGGTGAAATTCGTTGACCCTATCAAGATGCA
CTACAGCGAAAGCATTCGGCAAGTACTTCTCCATTAATCAAGAACGAAAGTTTGGGGATC
GAAGACGATCAGATACCGTCGTAGTCCAAACCATAAATGATGTCGATCAGGGATCGG-TA
GGATTT-AATTGTAAATTCTTATCGGAACCTT-GTGAGAAATCAT-GAATGTTTGGACTC
TGGGGGGAGTATGGTCGCAAGGCTGAAACTTAAAGGAATTGACGGAAGGGCACACAATGG
AGTGGAA-CCTGCGGCTTAATTT-GACTCAACTCGGGAAA-ACTTACCAAGCTCAGATAT
AATAAGGATTGACAGA-CTAA-AAGATCTTTCATGATTGTATAAGTGGTGGTGCATGGTC
GTTCTTAG-TTGGTGGAGTGATTTGTCTGGTCAATTCCGATAACGGACGAGACGTCTACT
TGCTAACTAGTAGGATTTATTTTTCCAAAT-GGGGGATAGTTAGT-TGGTGGTAGTATTC
A-----TTCGCAAG-GGTGG---GTA---TTCCATT-GATTAGTGTGTACTCTGGATTGA
TAG--ATTTTAG-TAAAAACATA---AA-CTTCTTAGAAGGACTACCT-ACCTCAAGTAG
GGGG-AAGTCGGACGCAATAACAGGTCTGTGATGCCCTTAGATACCTT-GGGCTGCACGC
GCGTTACAATGCAGCATAGAAAAAGGC----TC--C-------GTTGCTGG-AAA---G-
CAATGGTAATCAAAAGAATATACTACGTAATAGGGATTG-ATCTTTGTAATTATCGATCA
TAAACGAGGAATTCCTTGTAAGCGTAATTCATTACATTACGCTGAATATGTCCCTGCCCT
TTGTACACACCGCCCGTCGCTCCTACCGATCGAATGATTAGGTAAAACTGACAGAT----
-------AAA-A--A-TTTTTGTA-AA--A-----------AAAT--GTA-GAAG--TTA
T-T-TAAATCT--C----ATT--GTTT
>AM168111.1_Acytostelium_leptosomum_FG12
TCTAAG-TATAAACTTTTA-TACGGTGAAA----CTGCAGACGGCTCATT--ACAA--CA
---GT----GATAAACTA-CAGAACTTTCGA-GCGTA----AG-CTTTTT-GGATAACCG
CAGTAAATCGGGGCTAATACATATAAA-CG-GAAGG-CGATTGGG----CAACCAAG-AG
TCCTTGCGATTACT----A-GC---TT-C-A---A-ACCAACCTCTATG-AGATTGAGTT
GAAACCGAATAATATTGCTGATC-AGA--------ATT-TAATTCTGACAAGTTCTATGT
GTCACTGCCCTATCAACTTTCGATGGTACGGTATTGGCCTACCATGGTTGTAACGGGTAA
CGGGGAATCAGGGTTCGATTCCGGAGAGGGAGCCTGAGAAATGGCTACCACTTCTACGGA
AGGCAGCAGGCGCGCAAATT-ACTCAATCCCAATAC-GGGGAAGTAGTGACAATAAATAC
TAATGTTCTTCCATTT-TATGGAGA--ACAATTGGAATAAGTACAACGTAAATAGCTTCG
CA-AAAGTAATTGGAGGGCAAGTCTGGTGCCAGCAGCCGCGGTAATTCCAGCTCCAA-TA
GCGTATACTAAATTTGTTGCAGTTAAAA-AGC-TCGTAGTTTAGGCAGAGGTCATTCG-G
AT-TCAT------TAATTTCACCTTCAAAAGTG-ACTT------T-----AAAGGCTAT-
ATCACGCTTCGGTTGTGTTTAT-----GGTCA-CCGATGACTTGTATTATGA--CGT-TA
TTTGTTAGTTTACTAGCT-TATAATC---GTCAATCCACTGTGAGAAAA-TTGTGGTGTT
TAAAGCA--GG-C---GT----TCTAGCCTGATCAATGCAGCATGGTATGGTAAAATATG
ACAC---TTAACATTT-GTTGGTTT-ACT---TTGTTATAGTGTAATGACTAATAGGGAA
GGGCGGGGCCGTTCATATTGATGGGCGAGAGGTGAAATTCGTTGACCCTATCAAGATGCA

CTACAGCGAAAGCATTCGGCAAGTACTTCTCCATTAATCAAGAACGAAAGTTTGGGGATC
GAAGCCGATCAGATACCGTCGTAGTCCAAACCATAAATGATGTCGATCAGGGATCGG-TA
GGATTT-AATTGTAAATTCTTATCGGAACCTT-GTGAGAAATCAT-GAATGTTTGGACTC
TGGGGGGAGTATGGTCGCAAGGCTGAAACTTAAAGGAATTGACGGAAGGGCACACAATGG
AGTGGAA-CCTGCGGCTTAATTT-GACTCAACTCGGGAAA-ACTTACCAAGCTCAGATAT
AATAAGGATTGACAGA-CTAA-AAGATCTTTCATGATTGTATAAGTGGTGGTGCATGGTC
GTTCTTAG-TTGGTGGAGTGATTTGTCTGGTCAATTCCGATAACGGACGAGACGTCTACT
TGCTAACTAGTAGGATTTATTTTTCCAAAT-GGGGGATAGTTAGT-TGGTGGTAGTATTC
A-----TTCGCAAG-GGTGG---GTA---TTCCATT-GATTAGTGTGTACTCTGGATTGA
TAG--ATTTTAG-TAAAAACATA---AA-CTTCTTAGAAGGACTACCT-ACCTCAAGTAG
GGGG-AAGTCGGACGCAATAACAGGTCTGTGATGCCCTTAGATACCTT-GGGCTGCACGC
GCGTTACAATGCAGCATAGAAAAAGGC----TC--C-------GTTGCTGG-AAA---G-
CAATGGTAATCAAAAGAATATACTACGTAATAGGGATTG-ATCTTTGTAATTATCGATCA
TAAACGAGGAATTCCTTGTAAGCGTAATTCATTACATTACGCTGAATATGTCCCTGCCCT
TTGTACACACCGCCCGTCGCTCCTACCGATCGAATGATTAGGTAAAACTGACAGAT----
-------AAA-A--A-TTTTTGTA-AA--A-----------AAAT--GTA-GAAG--TTA
T-T-TAAATCT--C----ATT--GTTT
>HQ141514.1_Acytostelium_singulare_FDIB
TCTAAG-TATAAACTTTTA-TACGGTGAAA----CTGCAGACGGATCAAT--ACAA--CA
---GT----GATAAACTA-CAGAACTTTCGA-GCGTA----AG-CTTTTT-GGATAACCG
CAGTAAATCGGGGCTAATACATATAAA-CG-GAAGG-CGATTGGG----CAACCAAG-AG
TCCTTGCGATTACT----A-GC---TT-C-A---A-ACCAACCTCTATG-AGATTGAGTT
GAAACCGAATAATATTGCTGATC-AGA--------ATT-TAATTCTGACAAGTTCTATGT
GTCACTGCCCTATCAACTTTCGATGGTACGGTATTGGCCTACCATGGTTGTAACGGGTAA
CGGGGAATCAGGGTTCGATTCCGGAGAGGGAGCCTGAGAAATGGCTACCACTTCTACGGA
AGGCAGCAGGCGCGCAAATT-ACTCAATCCCAATAC-GGGGAAGTAGTGACAATAAATAC
TAATGTTCTTCCATTT-TATGGAGA--ACAATTGGAATAAGTACAACGTAAATAGCTTAG
CA-AAAGTAATTGGAGGGCAAGTCTGGTGCCAGCAGCCGCGGTAATTCCAGCTCCAA-TA
GCGTATACTAAATTTGTTGCAGTTAAAA-AGC-TCGTAGTTTAGGCAGAGGTCATTCG-G
AT-TCAT------TAATTTCACCTTCAAAAGTG-ACTT------T-----AAAGGCTAT-
ATCACGCTTCGGTTGTGTTTAT-----GGTCA-CCGATGACTTGTATTATGA--CGT-TA
TTTGTTAGTTTACTAGCT-TATAATC---GTCAATCCACTGTGAGAAAA-TTGTGGTGTT
TAAAGCA--GG-C---GT----TCTAGTCTGATCAATGCAGCATGGTATGGTAAAATATG
ACAC---TTAACATTT-GTTGGTTT-ACT---TTGTTATAGTGTAATGACTAATAGGGAA
GGGCGGGGCCGTTCATATTGATGGGCGAGAGGTGAAATTCGTTGACCCTATCAAGATGCA
CTACAGCGAAAGCATTCGGCAAGTACTTCTCCATTAATCAAGAACGAAAGTTTGGGGATC
GAAGACGATCAGATACCGTCGTAGTCCAAACCATAAATGATGTCGATCAGGGATCGG-TA
GGATTT-AATTGTAAATTCTTATCGGAACCTT-GTGAGAAATCAT-GAATGTTTGGACTC
TGGGGGGAGTATGGTCGCAAGGCTGAAACTTAAAGGAATTGACGGAAGGGCACACAATGG
AGTGGAA-CCTGCGGCTTAATTT-GACTCAACTCGGGAAA-ACTTACCAAGCTCAGATAT
AATAAGGATTGACAGA-CTAA-AAGATCTTTCATGATTGTATAAGTGGTGGTGCATGGTC
GTTCTTAG-TTGGTGGAGTGATTTGTCTGGTCAATTCCGATAACGGACGAGACGTCTACT
TGCTAACTAGTAGGATTTATTTTTCCAAAT-GGGGGATAGTTAGT-TGGTGGTAGTATTC
A-----TTCGCAAG-GGTGG---GTA---TTCCATT-GATTAGTGTGTACTCTGGATTGA
TAG--ATTTTAG-TAAAAACATA---AA-CTTCTTAGAAGGACTACCT-ACCTCAAGTAG
GGGG-AAGTCGGACGCAATAACAGGTCTGTGATGCCCTTAGATACCTT-GGGCTGCACGC
GCGTTACAATGCAGCATAGAAAAAGGC----TT--C-------GTTGCTGG-AAA---G-
CAATGGTAATCAAAAGAATATACTACGTAATAGGGATTG-ATCTTTGTAATTATCGATCA
TAAACGAGGAATTCCTTGTAAGCGTAATTCATTACATTACGCTGAATATGTCCCTGCCCT
TTGTACACACCGCCCGTCGCTCCTACCGATCGAATGATTAGGTAAAACTGACAGATGG--
-TGA---ACCTG--C-----AG-A-AGG-ATCA-TAACATT--GTG-TTGTATACAC-AT
--AGAC---A---C------------T
>AM168114.1_Acytostelium_digitatum_OH517
TCTAAG-TATAAACCTTTA-TACGGTGAAA----CTGCGGACGGCTCATT--ACAA--CA
---GT----GATAAACTA-AAGAACTTTCGC-GCGAA----AG-CGTTTT-GGATAACCG
CAGTAAATCGGGGCTAATACATATAAA-CG-AAAGG-CGAATGGTTTTATTACCATA-AG
TTCTTGCGATTATT----A-GTCATTT-T-A---A-ACCAACCTCTTCGGAGTTTGTGTT
GAAACCGAATAATATTGCTGATCGAGA--------ATT-TATTCTCGACAAGTTCTATGT
GTCACTGCCCTATCAACTTTGGATGGTACGGTATTGGCCTACCATGGTTGTAACGGGTGA
CGGGGAATCAGGGTTCGATTCCGGAGAGGGAGCCTGAGAAATGGCTACCACTTCTACGGA
AGGCAGCAGGCGCGCAAATT-ACTCAATCCCAATAC-GGGGAAGTAGTGACAATAAATAC
TAATGTCTTTCCAAAT-TTTGGAGG--ACAATTGGAATAAGTACAATGTAAATAGCTTAA
CA-AAAGTAATTGGAGGGCAAGTCTGGTGCCAGCAGCCGCGGTAATTCCAGCTCCAA-TA
GCGTATACTAAATTTGTTGCAGTTAAAA-AGC-TCGTAGTTAAGGTTGAAATCTTTCG-G
AT-TAAA-----AGAGTTACTCTTACCAAAGAG-----------T---TCTCCTTAGATC
ATTGCTTATCTAATAAGTTAATC----GATCA-CCGAAAATTTTTTTAAG----GT-TA
CTTTATAGGCAACTATAA-TTTAA-----ACCAATCCACTGTGAGAAAA-TTGTAGTGTT
TAAAGCA--AG-C---TT----TTATGCTTGTTCAATGCAGCATGGTATGGTAAAATAGG
ACAC---TTAACATTT-GTTGGTTT-ACCAA-TTGTTTAAGTGTAATGACTAATAGGGAA
AGTCGGGGCCGTTCATATTGATGGGCGAGAGGTGAAATTCGTTGACCCTATCAAGATGCA
CTACAGCGAAAGCATTCGGCAAGTATTTCTCCATTAATCAAGAACGAAAGTTTGGGGATC
GAAGACGATCAGATACCGTCGTAGTCCAAACCATAAACGATGTCGACCAGAGATTGGGTA
GACAAT-AAATATAAATGCAT-TCAGAACCTT-GTGAGAAATCAT-GAGTGTTTGGACTC

TGGGGGGAGTATGGTCGCAAGGCTGAAACTTAAAGGAATTGACGGAAGGGCACACAATGG
AGTGGAA-CCTGCGGCTTAATTT-GACTCAACTCGGGAAA-ACTTACCAAGCTCAGATAT
AATAAGGATTGACAGA-CTAA-AAGATCTTTCATGATTGTATAAGTGGTGGTGCATGGTC
GTTCTTAG-TTGGTGGAGTGATTTGTCTGGTCAATTCCGATAACGGACGAGACGTCTACT
TACTAACTAGTGGGATTTATCTAGTCAATTTGGAGGATAAGTTTT-TAGTGCTTCTATTC
AAGT--CTTTCGGG-GTTTG---GTA-G-TT-CATT-GAAGGCTGTGTACTCTGATTAGA
TAG--ATTCTAG-TAAAAACATA---AA-CTTCTTAGAAGGACTACCT-ACCTCAAGTGG
GGGG-AAGTTGGACGCAATAACAGGTCTGTGATGCCCTTAGATACCTT-GGGCTGCACGC
GCGTTACAATGTAACAGAGAAAAAGGC----TTTCC-------ATGTCTGG-AAA---GA
C-ATGGTAATCAATTGAATCTACTACGTAATAGGGATTG-ATCTTTGTAATTATCGATCA
TCAACGAGGAATTCCTTGTAAGCGTAAGTCATTACCTTACGCTGAATATGTCCCTGCCCT
TTGTACACACCGCCCGTCGCTCCTACCGATCGAACGATTAGGTAAAATTGACAGATT---
-TGATTTA------A-----AGCAGCAA---TGTT---ATTAAATC-TTA-GAAG--TTA
T-T-TAAATCT--C----ATT--GTTT
>AM168113.1_Acytostelium_serpentarium_SAB3A
TCTAAG-TATAAACTTTAA-TACGGTGAAA----CTGCGGACGGCTCATT--ACAA--CA
---GT----GATAAAGTA-CAGAACTTTCGC-GCGTA----AG-CGTCTTTGGATAACCG
CAGTAAATCGGGGCTAATACATATAAA-CG-AAAGA-TTAATAGTTT--CTACTGTT-GA
T-TTTGCGATTGTT----A-GTCATTT-CAA---A-ACTAGCCTCTTCGGAGTTAGTGGT
GAATCCGAACAATATTGCTGATCGA-A--------ATT-TATTT-CGACGAGTTCTATCT
GTCACTGCCCTATCAACTTTCGATGGTACGGTATTGGCCTACCATGGTTGTAACGGGTAA
CGGGGAATCAGGGTTCGATTCCGGAGAGGGCGCCTGAGAAATGGCGACCACTTCTACGGA
AGGCAGCAGGCGCGCAAATT-ACTCAATCCCAATAC-GGGGAAGTAGTGACAATAAATAC
TAATTTCTTTCCATTT-TATGGAGG--AAAATTGGAATAAGTACAACGTAAATAGCTTAA
CC-AAAGTAATTGGAGGGCAAGTCTGGTGCCAGCAGCCGCGGTAATTCCAGCTCCAA-TA
GCGTATACTAAATTTGTTGCAGTTAAAA-AGC-TCGTAGTTCAGATAAAGGTTGTTCG-G
GT-CAAG----------------------GTG-TCTTTA----T-AAAATAAGCATCTG
ATT-CTA-TCTAATTTATTAGTC-TAGTATCA-CCGACAAC-TTTATTTAGGG-TCT---
---GTTATCAATTTATT--GGTGATTT-GATCTATCCACTGTGAGAAAA-TTGTAGTGTT
TAAAGCA--AG-C---GT----TTCTGCTTGTTCAATGCAGCATGGTATGGTAAAATAGG
ACAC---TTAACAATT-GTTGGTTT-TTT-----GTTGAAGTGTAATGACTAATAGGGAA
GGGCGGGATCGTACATATTGATGGGCGAGAGGTGAAATTCGTTGACCCTATCAAGATGCA
CTACAGCGAAAGCATTCGATAAGTACTTCTCCATTAATCAAGAACGAAAGTTTGGGGATC
GAAGACGATCAGATACCGTCGTAGTCCAAACTATAAACTATGTCGACCAGGGATCGGATA
GATTAA-ATTTCTAACTCTAT-TCGGAACCTT-GTGAGAAATCAT-GAGTGTTTGGACTC
TGGGGGGAGTATGGTCGCAAGGCTGAAACTTAAAGGAATTGACGGAAGGGCACACAATGG
AGTGGAG-CCTGCGGCTTAATTT-GACTCAACTCGGGAAA-ACTTACCAAGCTCAGATAT
AATAAGGATTGACAGA-CTAA-AAGATCTTTCATGATTGTATAAGTGGTGGTGCATGGTC
GTTCTTAG-TTGGTGGAGTGATTTGTCTGGTCAATTCCGATAACGGACGAGACGTCTACT
TACTAAATAGTAGGATTTAT-GGGTCAATATGTGGGATAGGTCTT-AGGGGTTTGTA--T
ATAT--TTC-CAAAAGATTT---GTATGCTACCACT--ATGGCTGTGTAGCACGACTCTT
TAA--ATTCGAG-TAAAAAACTA---AA-CTTCTTAGAAGGACTACCC-ACCTCAAGTGG
GGGG-AAGTCGGACGCAATAACAGGTCTGTGATGCCCTTAGATACCTT-GGGCTGCACGC
GCGCTACAATGTAGAGATGAAAAAGGT----TTCCG-------GGTACCGG-AAG---GT
ATC-GGTAATCATTTGAATACTCTACGTGATAGGGATTG-ATCTTTGTAATTATCGATCA
TCAACGAGGAATTCCTTGTAAGCGTAAGTCATTACCTTACGCTGAATAGGTCCCTGCCCT
TTGTACACACCGCCCGTCGCTCCTACCGATCGAACGATTAGGTAAAACTGACGGACT---
-TAG---ACCTA--CCTTTTGG-CATGG----------------TC-GGG-GAAG--TTA
T-T-TAAATCT--C----ATT--GTTT
>AM168112.1_Rostrostelium_ellipticum_AE2
TCTAAG-TATAAACCTTTA-TACGGTGAAA----CTGCAGACGGCTCATT--ACAA--CA
---GT----GATAAACTA-ATGGACTTTCGC-GCCTTTAAAAG-CGTCTT-GGATATCCG
CAGTAAATCGGGGCTAATACATGCAAA-CG-AAAGGGTGAGCGGG----TAACCGCG-AG
CCTTTGCAATTATTT--TA-GCT-TTTGCT------ACCAACCTCTTAGGAGTTTGTGTT
GAATCCGAATAATTTTGCTGATCGGAA--------TTT-TA-TTCCGACAAGTTCAAAGT
GTCACTGCCCTATCAACTTTCGATGGTACGGTATTGGCCTACCATGGTTGTAACGGGTAA
CGGGGAATCAGGGTTCGGTTCCGGAGAGGGCGCCTGAGAAATGGCGACCACTTCTACGGA
AGGCAGCAGGCGCGCAAATT-ACTCAATCCCAATAC-GGGGAAGTAGTGACAATAAATAC
TGATGCCTTTCCATTT-TATGGAGG--GTAATTAGAATAAGTACAATTTAAATCGCTTAG
CC-AAAGTGATTGGAGGGCAAGTCTGGTGCCAGCAGCCGCGGTAATTCCAGCTCCAA-TA
GCATATACTAAATTTGTTGCAGTTAAAA-AGC-TCGTAGTTTAGAATGAAATTTTTCG--
-----AG----GG-ATTTT-GATTTTACAATCTGAACCC--T-GT-CT-TAAATTGCCTT
A-CGGG--TTAATTTTTGA---C--TGT-----CTCGATAATTTCAATTTTG-CCT--T-
T-A---GGCATTT-ATG--TCTATTA--GGTAGGTCCACTGTGAGAAAA-TTGTGGTGTT
TAAAGCG--GA-C---GT----TTTTGTTTGTTCATTGCAGCATGGTATGGTAAAACAAG
ACAC---TGTTCACTC-GTTGGTTG-TGAA------T-CAGTGTAATGACTAATAGGGAA
GGACGGGGCCGTTCATATTGATGGGCGAGAGGTGAAATTCGTTGACCCTATCAAGATGCA
CTACAGCGAAAGCATTCGGCAAGTACTTCCCCATTAATCAAGAACGAAAGTTTGGGGATC
GAAGACGATCAGATACCGTCGTAGTCCAAACCATAAACTATGTCGACCAGGGACTGGACG
GATGAT-TTTTAAAAAACTCGCTCAGAACCTT-GTGGGAAACCAT-GAGTGTTTGGATTC
CGGGGGGAGTATGGTCGCAAGTCTGAAACTTAAAGGAATTGACGGAAGGGCACACAATGG
AGTGGAA-CCTGCGGCTTAATTT-GACTCAACTCGGGAAA-ACTTACCAAGCTCAGATAT
AGTAAGGATTGACAGA-CTAA-AAGATTTTTCATGATTCTATAAGTGGTGGTGCATGGTC

GTTCTTAG-TTGGTGGAGTGATTTGTCTGGTCAATTCCGATAACGGACGAGACCTCTACC
TACTAAATAGTGGTATTTATTTTGTCGTTATGGGGGATAGTTAGT-TGGCGAGAGTAGT-
AGGT--TTTCGGA---TTTA---TTATTTCT-CGTTGAT-TGGTGTGTACCTCGATAAGA
TAG--ATTACTTCTAAAAA--TA---A--CTTCTTAGAGGGACTACCC-ACCTCAAGTGG
GGGG-AAGTCGGAGGCAATAACAGGTCTGTGATGCCCTTAGATACCTT-GGGCTGCACGC
GCGTTACAATGAAAGCGTGAAAAAGGC----TCCC--------AGCACCGG-AGG---GT
GT-TGGAAATCATATGAATCACTTTCGTAATGGGGATTG-ACCTTTGTAATTATCGGTCA
TAAACGAGGAATTCCTTGTAAGCGCAAGTCATTACCTTGCGTTGAATATGTCCCTGCCCT
TTGTACACACCGCCCGTCGCTCCTACCGATCGAACGATTAGGTAAAACTGACAGATTG--
-------TTTTG--TAAGGTAG-C-AAT-ATTTTAC------AGAA-TGA-AAAG--TTA
T-T-TAAATCT--C----ATT--GTTT
>KP167477.1_Heterostelium_lapidosum
TCTAAG-TATAA-CCTTTA-TACGGTGAAC----CTGCAGAAGGATCAT----CCC--G-
---GTTT-AGATAAACTATAAGAACTTTCGC-GCTTC-----GGCGTCTT-GGATAACCG
CAGTAAATCGGGGCTAATACATGTAAA-CG-AGAGGATGAGCGGG----CAACTGCG-AG
TCTTTGCGATTGTT----A-GCT-ATT-CA---CACACCAACCTCTTCGGAGTTTGTGGT
GAGTCCGAACAATATTGCTGATCGGAA--------ATT-TATTTCCGACAAGTTCTTTGT
GTCACTGCCCTATCAACTTTCGATGGTAAGGTATTGGCTTACCATGGTTGTAACGGGTGA
CGGGGAATCAGGGTTCGATTCCGGAGAGGGAGCCTGAGAAATGGCTACCACTTCTATGGA
AGGCAGCAGGCGCGCAAATT-ACTCAATCCCAATAC-GGGGAAGTAGTGACAAAAAATAC
TAATGCCTTTCCATTTATATGGGGG--GCAATTGGAATAAGTACAACTTAAATCGCTTAG
CA-AAAGTGATTGGAGGGCAAGTCTGGTGCCAGCAGCCGCGGTAATTCCAGCTCCAA-TA
GCGTATACTAAATTTGTTGCAGTTAAAA-AGC-TCGTAGTTGAGATTGAGATTTCTTG-G
GT-TTAG--CAGGCATTGTCGCCTT--CGGGTTGGCA--A-TCGT-TTGTAAAGCGCTAT
AATGGGATTTC-ACTCTCA-TTATATGTGTTA-CCAAGAAATTTCCAACTGC-CCA--TG
TCAAGTGGCAACACTTG--TCTGATA--GGGTGATCTACTGTGAGAAAA-TTGTAGTGTT
CAAAGCA--GG-C---GTC---TTACGTTTGTTCAATGCAGCATGGTATAGTAAAATATG
ACAC---TAAATATAT-GTTGGTTG-TATA---TTCT-TAGTGTAATGACTAATAGGGAA
GGGCGGGGCCGTTCATATTGATGGGCGAGAGGTGAAATTCGTTGACCCTATCAAGATGCA
CTACAGCGAAAGCATTCGGCAAGTACTTCTCCATTAATCAAGAACGAAAGTTTGGGGATC
GAAGACGATCAGATACCGTCGTAGTCCAAACCATAAACTATGTCGACCAGGGATTGGACG
GATAAT-TTTTTAAAAACTCGCTCAGAACCTT-GTGAGAAATCAT-GAGTGTTTGGACTC
TGGGGGGAGTATGGTCGCAAGTCTGAAACTTAAAGGAATTGACGGAAGGGCACACAATGG
AGTGGAA-CCTGCGGCTTAATTT-GACTCAACTCGGGAAA-ACTTACCAAGCTCAGATAT
AATAAGGATTGACAGA-CTAA-AAGATCTTTCATGATTGTATAAGTGGTGGTGCATGGTC
GTTCTTAG-TTGGTGGAGTGATTTGTCTGGTCAATTCCGATAACGGACGAGACCTCTACC
TGCTAACTAGTGGTATTTATTTGGTCAATATGGGAGATAGCTATT-TGGTGTTGGCAGTC
AGG----GTCAAA---CTTG---ATTGTCCTTCATTGAG-TGGTGTGTATTCTGATCAGA
TAG--GTACTAACTAAAAAA-TA---AA-CTTCTTAGAGGGACTACCT-ACCTCAAGTGG
GGGG-AAGTCGGAGGCAATAACAGGTCTGTGATGCCCTTAGATACCTT-GGGCTGCACGC
GCGTTACAATGTAGACGAGAAAAAGGT----CTCC--------GGCACCGA-AAG---GT
GTC-GGTAATCATATGAATTGTCTACGTAATGGGGATTA-ATTTTTGTAATTATCGATTA
TCAACGAGGAATTCCTTGTAAGCGTAAATCATTACTTTGCGCTGAATATGTCCCTGCCCT
TTGTACACACCGCCCGTCGCTCCTACCGATCGAACGATTAGGTAAAACTGACAGACTGT-
--AAC-GGATCT--GT---AAC-C-TGG---TTGAT-------CC---------------
----------------------TGCC-
>KP167474.1_Heterostelium_pseudocolligatum
--------------------ACGGTGGACACCTCGGCAGACGGGACAC---AGCA--G-
---GTTATAGATAAACTA-AAGAACTTTCGC-GCTTC-----GGCGTCTT-GGATAACCG
CAGTAAATCGGGGCTAATACATGTAAA-CG-AGAGGATGAGCGGG----CAACTGCG-AG
TCTTTGCGATTGTT----A-GCT-ATT-CA---CACACCAACCTCTTCGGAGTTTGTGGT
GAGTCCGAACAATATTGCTGATCGGAA--------ATT-TATTTCCGACAAGTTCTTTGT
GTCACTGCCCTATCAACTTTCGATGGTAAGGTATTGGCTTACCATGGTTGTAACGGGTGA
CGGGGAATCAGGGTTCGATTCCGGAGAGGGAGCCTGAGAAATGGCTACCACTTCTATGGA
AGGCAGCAGGCGCGCAAATT-ACTCAATCCCAATAC-GGGGAAGTAGTGACAAAAAATAC
TAATGCCTTTCCATTTATATGGGGG--GCAATTGGAATAAGTACAACTTAAATCGCTTAG
CA-AAAGTGATTGGAGGGCAAGTCTGGTGCCAGCAGCCGCGGTAATTCCAGCTCCAA-TA
GCGTATACTAAATTTGTTGCAGTTAAAA-AGC-TCGTAGTTGAGATTGAGATTTCTTG-G
GT-TTAG--CAGGCATTGTCGCCTT--CGGGTTGGCA--A-TCGT-TTGTAAAGCGCTAT
AATGGAATTTC-ACTTTCA-TTATATGTGTTA-CCAAGAAATTTCCAACTGC-CCA--TG
TCAAGTGGCAACACTTG--TCTGATA--GGGTGATCTACTGTGAGAAAA-TTGTAGTGTT
CAAAGCA--GG-C---GTC---TTACGTTTGTTCAATGCAGCATGGTATAGTAAAATAAG
ACAC---TAAATATAT-GTTGGTTG-TATA---TTCT-TAGTGTAATGACTAATAGGGAA
GGGCGGGGCCGTTCATATTGATGGGCGAGAGGTGAAATTCGTTGACCCTATCAAGATGCA
CTACAGCGAAAGCATTCGGCAAGTACTTCTCCATTAATCAAGAACGAAAGTTTGGGGATC
GAAGACGATCAGATACCGTCGTAGTCCAAACCATAAACTATGTCGACCAGGGATTGGACG
GATAAT-TTTTTAAAAACTCGCTCAGAACCTT-GTGAGAAATCAT-GAGTGTTTGGACTC
TGGGGGGAGTATGGTCGCAAGTCTGAAACTTAAAGGAATTGACGGAAGGGCACACAATGG
AGTGGAA-CCTGCGGCTTAATTT-GACTCAACTCGGGAAA-ACTTACCAAGCTCAGATAT
AATAAGGATTGACAGA-CTAA-AAGATCTTTCATGATTGTATAAGTGGTGGTGCATGGTC
GTTCTTAG-TTGGTGGAGTGATTTGTCTGGTCAATTCCGATAACGGACGAGACCTCTACC
TGCTAACTAGTGGTATTTATTTGGTCAATATGGGAGATAGCTATT-TGGTGTTGGCAGTC
AGG----GTCAAA---CTTG---ATTGTCCTTCATTGAG-TGGTGTGTATTCTGATCAGA

TAG--GTACTAACTAAAAAA-TA---AA-CTTCTTAGAGGGACTACCT-ACCTCAAGTGG
GGGG-AAGTCGGAGGCAATAACAGGTCTGTGATGCCCTTAGATACCTT-GGGCTGCACGC
GCGTTACAATGTAGACGAGAAAAAGGT----CTCC--------GGCACCGA-AAG---GT
GTC-GGTAATCATATGAATTGTCTACGTAATGGGGATTA-ATTTTGTAATTATCGATTA
TCAACGAGGAATTCCTTGTAAGCGTAAATCATTACTTTGCGCTGAATATGTCCCTGCCCT
TTGTACACACCGCCCGTCGCTCCTACCGATCGAACGATTAGGTAAAACTGACAGACTG--
--AGT-GGCTCT--GT---AAC-CCTGG---TTGAT-------CCC--------------
----------------------TGCC-
>HQ141505.1_Heterostelium_colligatum_HN13C1
TCTAAG-TATAA-CCTTTA-TACGGTGAAC----CTGCAGAAGGATCAACT-ACAGAGCA
--TGT----GATAAACTA-AAGAACTTTCGC-GCTTC-----GGCGTCTT-GGATAACCG
CAGTAAATCGGGGCTAATACATGTAAA-CG-AGAGGATGAGCGGG----CAACTGCG-AG
TCTTTGCGATTGTT----A-GCT-ATT-CA---CACACCAACCTCTTCGGAGTTTGTGGT
GAGTCCGAACAATATTGCTGATCGGAA--------ATT-TATTTCCGACAAGTTCTTTGT
GTCACTGCCCTATCAACTTTCGATGGTAAGGTATTGGCTTACCATGGTTGTAACGGGTGA
CGGGGAATCAGGGTTCGATTCCGGAGAGGGAGCCTGAGAAATGGCTACCACTTCTATGGA
AGGCAGCAGGCGCGCAAATT-ACTCAATCCCAATAC-GGGGAAGTAGTGACAAAAAATAC
TAATGCCTTTCCATTTATATGGGGG--GCAATTGGAATAAGTACAACTTAAATCGCTTAG
CA-AAAGTGATTGGAGGGCAAGTCTGGTGCCAGCAGCCGCGGTAATTCCAGCTCCAA-TA
GCGTATACTAAATTTGTTGCAGTTAAAA-AGC-TCGTAGTTGAGATTGAGATTTCTTG-G
GT-TTAG--CAGGCATTGTCGCCTT--CGGGTTGGCA--A-TCGT-TTGTAAAGCGCTAT
AATGGAATTTC-ACTTTCA-TTATATGTGTTA-CCAAGAAATTTCCAACTGC-CCA--TG
TCAAGTGGCAACACTTG--TCTGATA--GGGTGATCTACTGTGAGAAAA-TTGTAGTGTT
CAAAGCA--GG-C---GTC---TTACGTTTGTTCAATGCAGCATGGTATAGTAAAATATG
ACAC---TAAATATAT-GTTGGTTG-TATA---TTCT-TAGTGTAATGACTAATAGGGAA
GGGCGGGGCCGTTCATATTGATGGGCGAGAGGTGAAATTCGTTGACCCTATCAAGATGCA
CTACAGCGAAAGCATTCGGCAAGTACTTCTCCATTAATCAAGAACGAAAGTTTGGGGATC
GAAGACGATCAGATACCGTCGTAGTCCAAACCATAAACTATGTCGACCAGGGATTGGACG
GATAAT-TTTTTAAAAACTCGCTCAGAACCTT-GTGAGAAATCAT-GAGTGTTTGGACTC
TGGGGGGAGTATGGTCGCAAGTCTGAAACTTAAAGGAATTGACGGAAGGGCACACAATGG
AGTGGAA-CCTGCGGCTTAATTT-GACTCAACTCGGGAAA-ACTTACCAAGCTCAGATAT
AATAAGGATTGACAGA-CTAA-AAGATCTTTCATGATTGTATAAGTGGTGGTGCATGGTC
GTTCTTAG-TTGGTGGAGTGATTTGTCTGGTCAATTCCGATAACGGACGAGACCTCTACC
TGCTAACTAGTGGTATTTATTTGGTCAATATGGGAGATAGCTATT-TGGTGTTGGCAGTC
AGG----GTCAAA---CTTG---ATTGTCCTTCATTGAG-TGGTGTGTATTCTGATCAGA
TAG--GTACTAACTAAAAAA-TA---AA-CTTCTTAGAGGGACTACCT-ACCTCAAGTGG
GGGG-AAGTCGGAGGCAATAACAGGTCTGTGATGCCCTTAGATACCTT-GGGCTGCACGC
GCGTTACAATGTAGACGAGAAAAAGGT----CTCC--------GGCACCGA-AAG---GT
GTC-GGTAATCATATGAATTGTCTACGTAATGGGGATTA-ATTTTGTAATTATCGATTA
TCAACGAGGAATTCCTTGTAAGCGTAAATCATTACTTTGCGCTGAATATGTCCCTGCCCT
TTGTACACACCGCCCGTCGCTCCTACCGATCGAACGATTAGGTAAAACTGACAGACTG--
--AGT-GACTTC---T---AAC-CATGG---TTGAT-------CCT-GGT-GAACC--TG
-CA-GAAGGAT--C----A----GCC-
>HQ141509.1_Heterostelium_tikalense_HN1C1
TCTAAG-TATAA-CCTTTA-TACGGTGAAC----CTGCAGAAGGATCAACT-ACGAG-CA
--TGT---AGATAAACTA-AAGAACTTTCGC-GCTTC-----GGCGTCTT-GGATAACCG
CAGTAAATCGGGGCTAATACATGTAAA-CG-AGAGGATGAGCGGG----CAACTGCG-AG
TCTTTGCGATTGTT----A-GCT-ATT-CA---CACACCAACCTCTTCGGAGTTTGTGGT
GAGTCCGAACAATATTGCTGATCGGAA--------ATT-TATTTCCGACAAGTTCTTTGT
GTCACTGCCCTATCAACTTTCGATGGTAAGGTATTGGCTTACCATGGTTGTAACGGGTGA
CGGGGAATCAGGGTTCGATTCCGGAGAGGGAGCCTGAGAAATGGCTACCACTTCTATGGA
AGGCAGCAGGCGCGCAAATT-ACTCAATCCCAATAC-GGGGAAGTAGTGACAAAAAATAC
TAATGCCTTTCCATTTATATGGGGG--GCAATTGGAATAAGTACAACTTAAATCGCTTAG
CA-AAAGTGATTGGAGGGCAAGTCTGGTGCCAGCAGCCGCGGTAATTCCAGCTCCA--TA
GCGTATACTAAATTTGTTGCAGTTAAAA-AGC-TCGTAGTTGAGATTGAGATTTCTTG-G
GT-TTAG--CAGGCATTGTCGCCTT--CGGGTTGGCA--A-TCGT-TTGTAAAGCGCTAT
AATGGAATTTC-ACTTTCA-TTATATGTGTTA-CCAAGAAATTTCCAACTGC-CCA--TG
TCAAGTGGCAACACTTG--TCTGATA--GGGTGATCTACTGTGAGAAAA-TTGTAGTGTT
CAAAGCA--GG-C---GTC---TTACGTTTGTTCAATGCAGCATGGTATAGTAAAATATG
ACAC---TAAATATAT-GTTGGTTG-TATA---TTCT-TAGTGTAATGACTAATAGGGAA
GGGCGGGGCCGTTCATATTGATGGGCGAGAGGTGAAATTCGTTGACCCTATCAAGATGCA
CTACAGCGAAAGCATTCGGCAAGTACTTCTCCATTAATCAAGAACGAAAGTTTGGGGATC
GAAGACGATCAGATACCGTCGTAGTCCAAACCATAAACTATGTCGACCAGGGATTGGACG
GATAAT-TTTTTAAAAACTCGCTCAGAACCTT-GTGAGAAATCAT-GAGTGTTTGGACTC
TGGGGGGAGTATGGTCGCAAGTCTGAAACTTAAAGGAATTGACGGAAGGGCACACAATGG
AGTGGAA-CCTGCGGCTTAATTT-GACTCAACTCGGGAAA-ACTTACCAAGCTCAGATAT
AATAAGGATTGACAGA-CTAA-AAGATCTTTCATGATTGTATAAGTGGTGGTGCATGGTC
GTTCTTAG-TTGGTGGAGTGATTTGTCTGGTCAATTCCGATAACGGACGAGACCTCTACC
TGCTAACTAGTGGTATTTATTTGGTCAATATGGGAGATAGCTATT-TGGTGTTGGCAGTC
AGG----GTCAAA---CTTG---ATTGTCCTTCATTGAG-TGGTGTGTATTCTGATCAGA
TAG--GTACTAACTAAAAAA-TA---AA-CTTCTTAGAGGGACTACCT-ACCTCAAGTGG
GGGG-AAGTCGGAGGCAATAACAGGTCTGTGATGCCCTTAGATACCTT-GGGCTGCACGC
GCGTTACAATGTAGACGAGAAAAAGGT----CTCC--------GGCACCGA-AAG---GT

GTC-GGTAATCATATGAATTGTCTACGTAATGGGGATTA-ATTTTTGTAATTATCGATTA
TCAACGAGGAATTCCTTGTAAGCGTAAATCATTACTTTGCGCTGAATATGTCCCTGCCCT
TTGTACACACCGCCCGTCGCTCCTACCGATCGAACGATTAGGTAAAACTGACAGACTG--
--AGT-GACTTC----------------------------------GGT-GAACC--TG
-CA-GAAGGAT--C----A--------
>KP167476.1_Heterostelium_racemiferum
TCTAAG-TATAA-CCTTTA-TACGGTGAAC----CTGCAGAAGGCTCATT--ACAA--CA
---GT----GATAAACTA-AAGAACTTTCGC-GCTTC-----GGCGTCTT-GGATAACCG
CAGTAAATCGGGGCTAATACATGTAAA-CG-AGAGGATGAGCGGG----CAACTGCG-AG
TCTTTGCGATTGTT----A-GCT-ATT-CA---CACACCAACCTCTTCGGAGTTTGTGGT
GAGTCCGAACAATATTGCTGATCGGAA--------ATT-TATTTCCGACAAGTTCTTTGT
GTCACTGCCCTATCAACTTTCGATGGTAAGGTATTGGCTTACCATGGTTGTAACGGGTGA
CGGGGAATCAGGGTTCGATTCCGGAGAGGGAGCCTGAGAAATGGCTACCACTTCTATGGA
AGGCAGCAGGCGCGCAAATT-ACTCAATCCCAATAC-GGGGAAGTAGTGACAAAAAATAC
TAATGCCTTTCCATTTATATGGGGG--GCAATTGGAATAAGTACAACTTAAATCGCTTAG
CA-AAAGTGATTGGAGGGCAAGTCTGGTGCCAGCAGCCGCGGTAATTCCAGCTCCAA-TA
GCGTATACTAAATTTGTTGCAGTTAAAA-AGC-TCGTAGTTGAGATTGAGATTTCTTG-G
GT-TTAG--CAGGCATTGTCGCCTT--CGGGTTGGCA--A-TCGT-TTGTAAAGCGCTAT
AATGGGATTTC-ACTCTCA-TTATATGTGTTA-CCAAGAAATTTCCAACTGC-CCA--TG
TCAAGTGGCAACACTTG--TCTGATA--GGGTGATCTACTGTGAGAAAA-TTGTAGTGTT
CAAAGCA--GG-C---GTC---TTACGTTTGTTCAATGCAGCATGGTATAGTAAAATATG
ACAC---TAAATATAT-GTTGGTTG-TATA---TTCT-TAGTGTAATGACTAATAGGGAA
GGGCGGGGCCGTTCATATTGATGGGCGAGAGGTGAAATTCGTTGACCCTATCAAGATGCA
CTACAGCGAAAGCATTCGGCAAGTACTTCTCCATTAATCAAGAACGAAAGTTTGGGGATC
GAAGACGATCAGATACCGTCGTAGTCCAAACCATAAACTATGTCGACCAGGGATTGGACG
GATAAT-TTTTTAAAAACTCGCTCAGAACCTT-GTGAGAAATCAT-GAGTGTTTGGACTC
TGGGGGGAGTATGGTCGCAAGTCTGAAACTTAAAGGAATTGACGGAAGGGCACACAATGG
AGTGGAA-CCTGCGGCTTAATTT-GACTCAACTCGGGAAA-ACTTACCAAGCTCAGATAT
AATAAGGATTGACAGA-CTAA-AAGATCTTTCATGATTGTATAAGTGGTGGTGCATGGTC
GTTCTTAG-TTGGTGGAGTGATTTGTCTGGTCAATTCCGATAACGGACGAGACCTCTACC
TGCTAACTAGTGGTATTTATTTGGTCAATATGGGAGATAGCTATT-TGGTGTTGGCAGTC
AGG----GTCAAA---CTTG---ATTGTCCTTCATTGAG-TGGTGTGTATTCTGATCAGA
TAG--GTACTAACTAAAAAA-TA---AA-CTTCTTAGAGGGACTACCT-ACCTCAAGTGG
GGGG-AAGTCGGAGGCAATAACAGGTCTGTGATGCCCTTAGATACCTT-GGGCTGCACGC
GCGTTACAATGTAGACGAGAAAAAGGT----CTCC--------GGCACCGA-AAG---GT
GTC-GGTAATCATATGAATTGTCTACGTAATGGGGATTA-ATTTTTGTAATTATCGATTA
TCAACGAGGAATTCCTTGTAAGCGTAAATCATTACTTTGCGCTGAATATGTCCCTGCCCT
TTGTACACACCGCCCGTCGCTCCTACCGATCGAACGATTAGGTAAAACTGACAGACTG--
--AAT-GGCTT--

>AM168098.1_Heterostelium_colligatum_OH538
TCTAAG-TATAA-CCTTTA-TACGGTGAAA----CTGCAGACGGCTCATT--ACAA--CA
---GT----GATAAACTA-AAGAACTTTCGC-GCTTC-----GGCGTCTT-GGATAACCG
CAGTAAATCGGGGCTAATACATGTAAA-CG-AGAGGATGAGCGGG----CAACTGCG-AG
TCTTTGCGATTGTT----A-GCT-ATT-CA---CACACCAACCTCTTCGGAGTTTGTGGT
GAGTCCGAACAATATTGCTGATCGGAA--------ATT-TATTTCCGACAAGTTCTTTGT
GTCACTGCCCTATCAACTTTCGATGGTAAGGTATTGGCTTACCATGGTTGTAACGGGTGA
CGGGGAATCAGGGTTCGATTCCGGAGAGGGAGCCTGAGAAATGGCTACCACTTCTATGGA
AGGCAGCAGGCGCGCAAATT-ACTCAATCCCAATAC-GGGGAAGTAGTGACAAAAAATAC
TAATGCCTTTCCATTTATATGGGGG--GCAATTGGAATAAGTACAACTTAAATCGCTTAG
CA-AAAGTGATTGGAGGGCAAGTCTGGTGCCAGCAGCCGCGGTAGTTCCAGCTCCAA-TA
GCGTATACTAAATTTGTTGCAGTTAAAA-AGC-TCGTAGTTGAGATTGAGATTTCTTG-G
GT-TTAG--CAGGCATTGTCGCCTT--CGGGTTGGCA--A-TCGT-TTGTAAAGCGCTAT
AATGGAATTTC-ACTTTCA-TTATATGTGTTA-CCAAGAAATTTCCAACTGC-CCA--TG
TCAAGTGGCAACACTTG--TCTGATA--GGGTGATCTACTGTGAGAAAA-TTGTAGTGTT
CAAAGCA--GG-C---GTC---TTACGTTTGTTCAATGCAGCATGGTATAGTAAAATATG
ACAC---TAAATATAT-GTTGGTTG-TATA---TTCT-TAGTGTAATGACTAATAGGGAA
GGGCGGGGCCGTTCATATTGATGGGCGAGAGGTGAAATTCGTTGACCCTATCAAGATGCA
CTACAGCGAAAGCATTCGGCAAGTACTTCTCCATTAATCAAGAACGAAAGTTTGGGGATC
GAAGACGATCAGATACCGTCGTAGTCCAAACCATAAACTATGTCGACCAGGGATTGGACG
GATAAT-TTTTTAAAAACTCGCTCAGAACCTT-GTGAGAAATCAT-GAGTGTTTGGACTC
TGGGGGGAGTATGGTCGCAAGTCTGAAACTTAAAGGAATTGACGGAAGGGCACACAATGG
AGTGGAA-CCTGCGGCTTAATTT-GACTCAACTCGGGAAA-ACTTACCAAGCTCAGATAT
AATAAGGATTGACAGA-CTAA-AAGATCTTTCATGATTGTATAAGTGGTGGTGCATGGTC
GTTCTTAG-TTGGTGGAGTGATTTGTCTGGTCAATTCCGATAACGGACGAGACCTCTACC
TGCTAACTAGTGGTATTTATTTGGTCAATATGGGAGATAGCTATT-TGGTGTTGGCAGTC
AGG----GTCAAA---CTTG---ATTGTCCTTCATTGAG-TGGTGTGTATTCTGATCAGA
TAG--GTACTAACTAAAAAA-TA---AA-CTTCTTAGAGGGACTACCT-ACCTCAAGTGG
GGGG-AAGTCGGAGGCAATAACAGGTCTGTGATGCCCTTAGATACCTT-GGGCTGCACGC
GCGTTACAATGTAGACGAGAAAAAGGT----CTCC--------GGCACCGA-AAG---GT
GTC-GGTAATCATATGAATTGTCTACGTAATGGGGATTA-ATTTTTGTAATTATCGATTA
TCAACGAGGAATTCCTTGTAAGCGTAAATCATTACTTTGCGCTGAATATGTCCCTGCCCT
TTGTACACACCGCCCGTCGCTCCTACCGATCGAACGATTAGGTAAAACTGACAGACTG--

--AGT-GACTTC--C----CGC-A-AGG-GATTGTT-------GTT-TGG-AAAG--TTA
-GT-TAAATCT--C----ATT--GTTT
>AM168106.1_Heterostelium_tikalense_OH595
TCTAAG-TATAA-CCTTTA-TACGGTGAAA----CTGCAGACGGCTCATT--ACAA--CA
---GT----GATAAACTA-AAGAACTTTCGC-GCTTC-----GGCGTCTT-GGATAACCG
CAGTAAATCGGGGCTAATACATGTAAA-CG-AGAGGATGAGCGGG----CAACTGCG-AG
TCTTTGCGATTGTT----A-GCT-ATT-CA---CACACCAACCTCTTCGGAGTTTGTGGT
GAGTCCGAACAATATTGCTGATCGGAA--------ATT-TATTTCCGACAAGTTCTTTGT
GTCACTGCCCTATCAACTTTCGATGGTAAGGTATTGGCTTACCATGGTTGTAACGGGTGA
CGGGGAATCAGGGTTCGATTCCGGAGAGGGAGCCTGAGAAATGGCTACCACTTCTATGGA
AGGCAGCAGGCGCGCAAATT-ACTCAATCCCAATAC-GGGGAAGTAGTGACAAAAAATAC
TAATGCCTTTCCATTTATATGGGGG--GCAATTGGAATAAGTACAACTTAAATCGCTTAG
CA-AAAGTGATTGGAGGGCAAGTCTGGTGCCAGCAGCCGCGGTAATACCAGCTCCAA-TA
GCGTATACTAAATTTGTTGCAGTTAAAA-AGC-TCGTAGTTGAGATTGAGATTTCTTG-G
GT-TTAG--CAGGCATTGTCGCCTT--CGGGTTGGCA--A-TCGT-TTGTAAAGCGCTAT
AATGGAATTTC-ACTTTCA-TTATATGTGTTA-CCAAGAAATTTCCAACTGC-CCA--TG
TCAAGTGGCAACACTTG--TCTGATA--GGGTGATCTACTGTGAGAAAA-TTGTAGTGTT
CAAAGCA--GG-C---GTC---TTACGTTTGTTCAATGCAGCATGGTATAGTAAAATATG
ACAC---TAAATATAT-GTTGGTTG-TATA---TTCT-TAGTGTAATGACTAATAGGGAA
GGGCGGGGCCGTTCATATTGATGGGCGAGAGGTGAAATTCGTTGACCCTATCAAGATGCA
CTACAGCGAAAGCATTCGGCAAGTACTTCTCCATTAATCAAGAACGAAAGTTTGGGGATC
GAAGACGATCAGATACCGTCGTAGTCCAAACCATAAACTATGTCGACCAGGGATTGGACG
GATAAT-TTTTTAAAAACTCGCTCAGAACCTT-GTGAGAAATCAT-GAGTGTTTGGACTC
TGGGGGGAGTATGGTCGCAAGTCTGAAACTTAAAGGAATTGACGGAAGGGCACACAATGG
AGTGGAA-CCTGCGGCTTAATTT-GACTCAACTCGGGAAA-ACTTACCAAGCTCAGATAT
AATAAGGATTGACAGA-CTAA-AAGATCTTTCATGATTGTATAAGTGGTGGTGCATGGTC
GTTCTTAG-TTGGTGGAGTGATTTGTCTGGTCAATTCCGATAACGGACGAGACCTCTACC
TGCTAACTAGTGGTATTTATTTGGTCAATATGGGAGATAGCTATT-TGGTGTTGGCAGTC
AGG----GTCAAA---CTTG---ATTGTCCTTCATTGAG-TGGTGTGTATTCTGATCAGA
TAG--GTACTAACTAAAAAA-TA---AA-CTTCTTAGAGGGACTACCT-ACCTCAAGTGG
GGGG-AAGTCGGAGGCAATAACAGGTCTGTGATGCCCTTAGATACCTT-GGGCTGCACGC
GCGTTACAATGTAGACGAGAAAAAGGT----CTCC--------GGCACCGA-AAG---GT
GTC-GGTAATCATATGAATTGTCTACGTAATGGGGATTA-ATTTTTGTAATTATCGATTA
TCAACGAGGAATTCCTTGTAAGCGTAAATCATTACTTTGCGCTGAATATGTCCCTGCCCT
TTGTACACACCGCCCGTCGCTCCTACCGATCGAACGATTAGGTAAAACTGACAGACTG--
--AGT-GACTTC--C----CGC-A-AGG-GATTGTT-------GTT-TGG-AAAG--TTA
-GT-TAAATCT--C----ATT--GTTT
>KP167475.1_Heterostelium_plurimicrocystogenum
TCTAAG-TATAA-CCTTTA-TACGGTGAAA----CTGCAGACGGCTCAAT--ACAA--CA
---GT----GATAAACTA-AAGAACTTTCGC-GCTTC-----GGCGTCTT-GGATAACCG
CAGTAAATCGGGGCTAATACATGTAAA-CG-AGAGGATGAGCGGG----CAACTGCG-AG
TCTTTGCGATTGTT----A-GCT-ATT-CA---CACACCAACCTCTTCGGAGTTTGTGGT
GAGTCCGAACAATATTGCTGATCGGAA--------ATT-TATTTCCGACAAGTTCTTTGT
GTCACTGCCCTATCAACTTTCGATGGTAAGGTATTGGCTTACCATGGTTGTAACGGGTGA
CGGGGAATCAGGGTTCGATTCCGGAGAGGGAGCCTGAGAAATGGCTACCACTTCTATGGA
AGGCAGCAGGCGCGCAAATT-ACTCAATCCCAATAC-GGGGAAGTAGTGACAAAAAATAC
TAATGCCTTTCCATTTATATGGGGG--GCAATTGGAATAAGTACAACTTAAATCGCTTAG
CA-AAAGTGATTGGAGGGCAAGTCTGGTGCCAGCAGCCGCGGTAATTCCAGCTCCAA-TA
GCGTATACTAAATTTGTTGCAGTTAAAA-AGC-TCGTAGTTGAGATTGAGATTTCTTG-G
GT-TTAG--CAGGCATTGTCGCCTT--CGGGTTGGCA--A-TCGT-TTGTAAAGCGCTAT
AATGGAATTTC-ACTTTCA-TTATATGTGTTA-CCAAGAAATTTCCAACTGC-CCA--TG
TCAAGTGGCAACACTTG--TCTGATA--GGGTGATCTACTGTGAGAAAA-TTGTAGTGTT
CAAAGCA--GG-C---GTC---TTACGTTTGTTCAATGCAGCATGGTATAGTAAAATAAG
ACAC---TAAATATAT-GTTGGTTG-TATA---TTCT-TAGTGTAATGACTAATAGGGAA
GGGCGGGGCCGTTCATATTGATGGGCGAGAGGTGAAATTCGTTGACCCTATCAAGATGCA
CTACAGCGAAAGCATTCGGCAAGTACTTCTCCATTAATCAAGAACGAAAGTTTGGGGATC
GAAGACGATCAGATACCGTCGTAGTCCAAACCATAAACTATGTCGACCAGGGATTGGACG
GATAAT-TTTTTAAAAACTCGCTCAGAACCTT-GTGAGAAATCAT-GAGTGTTTGGACTC
TGGGGGGAGTATGGTCGCAAGTCTGAAACTTAAAGGAATTGACGGAAGGGCACACAATGG
AGTGGAA-CCTGCGGCTTAATTT-GACTCAACTCGGGAAA-ACTTACCAAGCTCAGATAT
AATAAGGATTGACAGA-CTAA-AAGATCTTTCATGATTGTATAAGTGGTGGTGCATGGTC
GTTCTTAG-TTGGTGGAGTGATTTGTCTGGTCAATTCCGATAACGGACGAGACCTCTACC
TGCTAACTAGTGGTATTTATTTGGTCAATATGGGAGATAGCTATT-TGGTGTTGGCAGTC
AGG----GTCAAA---CTTG---ATTGTCCTTCATTGAG-TGGTGTGTATTCTGATCAGA
TAG--GTACTAACTAAAAAA-TA---AA-CTTCTTAGAGGGACTACCT-ACCTCAAGTGG
GGGG-AAGTCGGAGGCAATAACAGGTCTGTGATGCCCTTAGATACCTT-GGGCTGCACGC
GCGTTACAATGTAGACGAGAAAAAGGT----CTCC--------GGCACCGA-AAG---GT
GTC-GGTAATCATATGAATTGTCTACGTAATGGGGATTA-ATTTTTGTAATTATCGATTA
TCAACGAGGAATTCCTTGTAAGCGTAAATCATTACTTTGCGCTGAATATGTCCCTGCCCT
TTGTACACACCGCCCGTCGCTCCTACCGATCGAACGATTAGGTAAAACTGACAGACTG--
--AGT-GGCTTC--T--
-------------C-------------
>KP167472.1_Heterostelium_pseudoplasmodiomagnum

TCTAAG-TATAA-CCTTTA-TACGGTGAAA----CTGCAGACGGATCAAT--ACAA--CA
---GT----GATAAACTA-AAGAACTTTCGC-GCTTC-----GGCGTCTT-GGATAACCG
CAGTAAATCGGGGCTAATACATGTAAA-CG-AGAGGATGAGCGGG----CAACTGCG-AG
TCTTTGCGATTGTT----A-GCT-ATT-CA---CACACCAACCTCTTCGGAGTTTGTGGT
GAGTCCGAACAATATTGCTGATCGGAA--------ATT-TATTTCCGACAAGTTCTTTGT
GTCACTGCCCTATCAACTTTCGATGGTAAGGTATTGGCTTACCATGGTTGTAACGGGTGA
CGGGGAATCAGGGTTCGATTCCGGAGAGGGAGCCTGAGAAATGGCTACCACTTCTATGGA
AGGCAGCAGGCGCGCAAATT-ACTCAATCCCAATAC-GGGGAAGTAGTGACAAAAAATAC
TAATGCCTTTCCATTTATATGGGGG--GCAATTGGAATAAGTACAACTTAAATCGCTTAG
CA-AAAGTGATTGGAGGGCAAGTCTGGTGCCAGCAGCCGCGGTAATTCCAGCTCCAA-TA
GCGTATACTAAATTTGTTGCAGTTAAAA-AGC-TCGTAGTTGAGATTGAGATTTCTTG-G
GT-TTAG--CAGGCATTGTCGCCTT--CGGGTTGGCA--A-TCGT-TTGTAAAGCGCTAT
AATGGAATTTC-ACTTTCA-TTATATGTGTTA-CCAAGAAATTTCCAACTGC-CCA--TG
TCAAGTGGCAACACTTG--TCTGATA--GGGTGATCTACTGTGAGAAAA-TTGTAGTGTT
CAAAGCA--GG-C---GTC---TTACGTTTGTTCAATGCAGCATGGTATAGTAAAATAAG
ACAC---TAAATATAT-GTTGGTTG-TATA---TTCT-TAGTGTAATGACTAATAGGGAA
GGGCGGGGCCGTTCATATTGATGGGCGAGAGGTGAAATTCGTTGACCCTATCAAGATGCA
CTACAGCGAAAGCATTCGGCAAGTACTTCTCCATTAATCAAGAACGAAAGTTTGGGGATC
GAAGACGATCAGATACCGTCGTAGTCCAAACCATAAACTATGTCGACCAGGGATTGGACG
GATAAT-TTTTTAAAAACTCGCTCAGAACCTT-GTGAGAAATCAT-GAGTGTTTGGACTC
TGGGGGGAGTATGGTCGCAAGTCTGAAACTTAAAGGAATTGACGGAAGGGCACACAATGG
AGTGGAA-CCTGCGGCTTAATTT-GACTCAACTCGGGAAA-ACTTACCAAGCTCAGATAT
AATAAGGATTGACAGA-CTAA-AAGATCTTTCATGATTGTATAAGTGGTGGTGCATGGTC
GTTCTTAG-TTGGTGGAGTGATTTGTCTGGTCAATTCCGATAACGGACGAGACCTCTACC
TGCTAACTAGTGGTATTTATTTGGTCAATATGGGAGATAGCTATT-TGGTGTTGGCAGTC
AGG----GTCAAA---CTTG---ATTGTCCTTCATTGAG-TGGTGTGTATTCTGATCAGA
TAG--GTACTAACTAAAAAA-TA---AA-CTTCTTAGAGGGACTACCT-ACCTCAAGTGG
GGGG-AAGTCGGAGGCAATAACAGGTCTGTGATGCCCTTAGATACCTT-GGGCTGCACGC
GCGTTACAATGTAGACGAGAAAAAGGT----CTCC--------GGCACCGA-AAG---GT
GTC-GGTAATCATATGAATTGTCTACGTAATGGGGATTA-ATTTTTGTAATTATCGATTA
TCAACGAGGAATTCCTTGTAAGCGTAAATCATTACTTTGCGCTGAATATGTCCCTGCCCT
TTGTACACACCGCCCGTCGCTCCTACCGATCGAACGATTAGGTAAAACTGACAGACTG--
--AGT-GGCTTC--T---

>KP167473.1_Heterostelium_unguliferum
TCTAAG-TATAA-CCTTTA-TACGGTGAAA----CTGCAGACGGATCAAT--ACAA--CA
---GT----GATAAACTA-AAGAACTTTCGC-GCTTC-----GGCGTCTT-GGATAACCG
CAGTAAATCGGGGCTAATACATGTAAA-CG-AGAGGATGAGCGGG----CAACTGCG-AG
TCTTTGCGATTGTT----A-GCT-ATT-CA---CACACCAACCTCTTCGGAGTTTGTGGT
GAGTCCGAACAATATTGCTGATCGGAA--------ATT-TATTTCCGACAAGTTCTTTGT
GTCACTGCCCTATCAACTTTCGATGGTAAGGTATTGGCTTACCATGGTTGTAACGGGTGA
CGGGGAATCAGGGTTCGATTCCGGAGAGGGAGCCTGAGAAATGGCTACCACTTCTATGGA
AGGCAGCAGGCGCGCAAATT-ACTCAATCCCAATAC-GGGGAAGTAGTGACAAAAAATAC
TAATGCCTTTCCATTTATATGGGGG--GCAATTGGAATAAGTACAACTTAAATCGCTTAG
CA-AAAGTGATTGGAGGGCAAGTCTGGTGCCAGCAGCCGCGGTAATTCCAGCTCCAA-TA
GCGTATACTAAATTTGTTGCAGTTAAAA-AGC-TCGTAGTTGAGATTGAGATTTCTTG-G
GT-TTAG--CAGGCATTGTCGCCTT--CGGGTTGGCA--A-TCGT-TTGTAAAGCGCTAT
AATGGAATTTC-ACTTTCA-TTATATGTGTTA-CCAAGAAATTTCCAACTGC-CCA--TG
TCAAGTGGCAACACTTG--TCTGATA--GGGTGATCTACTGTGAGAAAA-TTGTAGTGTT
CAAAGCA--GG-C---GTC---TTACGTTTGTTCAATGCAGCATGGTATAGTAAAATAAG
ACAC---TAAATATAT-GTTGGTTG-TATA---TTCT-TAGTGTAATGACTAATAGGGAA
GGGCGGGGCCGTTCATATTGATGGGCGAGAGGTGAAATTCGTTGACCCTATCAAGATGCA
CTACAGCGAAAGCATTCGGCAAGTACTTCTCCATTAATCAAGAACGAAAGTTTGGGGATC
GAAGACGATCAGATACCGTCGTAGTCCAAACCATAAACTATGTCGACCAGGGATTGGACG
GATAAT-TTTTTAAAAACTCGCTCAGAACCTT-GTGAGAAATCAT-GAGTGTTTGGACTC
TGGGGGGAGTATGGTCGCAAGTCTGAAACTTAAAGGAATTGACGGAAGGGCACACAATGG
AGTGGAA-CCTGCGGCTTAATTT-GACTCAACTCGGGAAA-ACTTACCAAGCTCAGATAT
AATAAGGATTGACAGA-CTAA-AAGATCTTTCATGATTGTATAAGTGGTGGTGCATGGTC
GTTCTTAG-TTGGTGGAGTGATTTGTCTGGTCAATTCCGATAACGGACGAGACCTCTACC
TGCTAACTAGTGGTATTTATTTGGTCAATATGGGAGATAGCTATT-TGGTGTTGGCAGTC
AGG----GTCAAA---CTTG---ATTGTCCTTCATTGAG-TGGTGTGTATTCTGATCAGA
TAG--GTACTAACTAAAAAA-TA---AA-CTTCTTAGAGGGACTACCT-ACCTCAAGTGG
GGGG-AAGTCGGAGGCAATAACAGGTCTGTGATGCCCTTAGATACCTT-GGGCTGCACGC
GCGTTACAATGTAGACGAGAAAAAGGT----CTCC--------GGCACCGA-AAG---GT
GTC-GGTAATCATATGAATTGTCTACGTAATGGGGATTA-ATTTTTGTAATTATCGATTA
TCAACGAGGAATTCCTTGTAAGCGTAAATCATTACTTTGCGCTGAATATGTCCCTGCCCT
TTGTACACACCGCCCGTCGCTCCTACCGATCGAACGATTAGGTAAAACTGACAGACTG--
--AGT-GGCTTC--T---

>HQ141504.1_Heterostelium_sp._TH12A
TCTAAG-TATAA-CCTTTA-TACGGTGAAC----CTGCAGAAGGATCAAT--ACGAG-CA
---GT----GATAAACTA-AAGAACTTTCGC-GCTTC-----GGCGTCTT-GGATAACCG
CAGTAAATCGGGGCTAATACATGTAAA-CG-AGAGGATGAGCGGG----TAACCGCG-AG

TCTTTGCGATTGTT----A-GCT-TTTACA---CACACCAACCTCTTCGGAGATTGTGGT
GAGTCCGAACAATATTGCTGATCGGAA--------ATT-TATTTCCGACGAGTTCTTTGT
GTCACTGCCCTATCAACTTTCGATGGTAAGGTATTGGCTTACCATGGTTGTAACGGGTGA
CGGGGAATCAGGGTTCGATTCCGGAGAGGGAGCCTGAGAAATGGCTACCACTTCTATGGA
AGGCAGCAGGCGCGCAAATT-ACTCAATCCCAATAC-GGGGAAGTAGTGACAAAAAATAC
TAATGCCTTACCATTTATATGGGGG--GCAATTGGAATAAGTACAACCTAAATCGCTTAG
CA-AAAGTGATTGGAGGGCAAGTCTGGTGCCAGCAGCCGCGGTAATTCCAGCTCCAA-TA
GCGTATACTAAATTTGTTGCAGTTAAAA-AGC-TCGTAGTTGAGATTGAGATTTCTTG-G
GT-TTAG--CGTTCATTATTGCCTT--CGGGT-AATA--A-CCGT-TCGTAAAGCT-TAT
AATCGGGATTC--GTTTCG-TT-TATGAGTTA-CCAAGAAATTTCTATATGC-CCA--TG
TCAACTGGTAACAGTTG--TCTGATC--GGGTGATCTACTGTGAGAAAA-TTGTAGTGTT
CAAAGCA--GG-C---GTC---TTTCGCTTGTTCAATGCAGCATGGTATAGTAAAATAAG
ACAC---TAAATATAT-GTTGGTTG-TATA---TTCT--AGTGTAATGACTAATAGGGAA
GGGCGGGGCCGTTCATATTGATGGGCGAGAGGTGAAATTCGTTGACCCTATCAAGATGCA
CTACAGCGAAAGCATTCGGCAAGTACTTCTCCATTAATCAAGAACGAAAGTTTGGGGATC
GAAGACGATCAGATACCGTCGTAGTCCAAACCATAAACTATGTCGACCAGGGATTGGACG
GATAAT-TTTTTAAAAACTCGCTCAGAACCTT-GTGAGAAATCAT-GAGTGTTTGGACTC
TGGGGGGAGTATGGTCGCAAGTCTGAAACTTAAAGGAATTGACGGAAGGGCACACAATGG
AGTGGAA-CCTGCGGCTTAATTT-GACTCAACTCGGGAAA-ACTTACCAAGCTCAGATAT
AATAAGGATTGACAGA-CTAA-AAGATCTTTCATGATTGTATAAGTGGTGGTGCATGGTC
GTTCTTAG-TTGGTGGAGTGATTTGTCTGGTCAATTCCGATAACGGACGAGACCTCTACC
TACTAAATAGTGGTATTTATTTGGTCAACATGGAAGATAGTCATT-TGGTGTTGGC-GTT
AGGT---GTCAAA--GCTTA---GC-GTTCTTCATTGAG-TGGTGTGTATTTCGATCAGA
TAG--GTACTAACTAAAAAA-TA---AA-CTTCTTAGAGGGACTACCT-ACCTCAAGTGG
GGGG-AAGTCGGAGGCAATAACAGGTCTGTGATGCCCTTAGATACCTT-GGGCTGCACGC
GCGTTACAATGTAGACTAGAAAAAGGT----TTCC--------GACATCGA-AAG---GT
GCC-GGTAATCAATTGAATAGTCTACGTAATGGGGATTA-ATTTTTGTAATTATCGATTA
TCAACGAGGAATTCCTTGTAAGCGTAAATCATTACTTTACGCTGAATATGTCCCTGCCCT
TTGTACACACCGCCCGTCGCTCCTACCGATCGAACGATTAGGTAAAACTGACGGACTG--
--AAT-GACTTC--T----AAC-C-TGG---TTG--------------GT-GAACC--TG
-CA-GAAGGAT--C--AGATCCTGCC-
>HQ141498.1_Heterostelium_candidum_bsb6b
TCTAAG-TATAA-CCTTTA-TACGGTGAAC----CTGCAGAAGGATCAAC---CA---G-
---GTTT-AGATAAACTA-AAGAACTTTCGC-GCTTC-----GGCGTCTT-GGATAACCG
CAGTAAATCGGGGCTAATACATGTAAA-CG-AGAGGATGAGCGGG----TAACCGCG-AG
TCTTTGCGATTGTT----A-GCT-TTTACA---CACACCAACCTCTTCGGAGATTGTGGT
GAGTCCGAACAATATTGCTGATCGGAA--------ATT-TATTTCCGACGAGTTCTTTGT
GTCACTGCCCTATCAACTTTCGATGGTAAGGTATTGGCTTACCATGGTTGTAACGGGTGA
CGGGGAATCAGGGTTCGATTCCGGAGAGGGAGCCTGAGAAATGGCTACCACTTCTATGGA
AGGCAGCAGGCGCGCAAATT-ACTCAATCCCAATAC-GGGGAAGTAGTGACAAAAAATAC
TAATGCCTTACCATTTATATGGGGG--GCAATTGGAATAAGTACAACCTAAATCGCTTAG
CA-AAAGTGATTGGAGGGCAAGTCTGGTGCCAGCAGCCGCGGTAATTCCAGCTCCAA-TA
GCGTATACTAAATTTGTTGCAGTTAAAA-AGC-TCGTAGTTGAGATTGAGATTTCTTG-G
GT-TTAG--CGTTCATTATTGCCTT--CGGGT-AATA--A-CCGT-TCGTAAAGCT-TCT
AATCGGGATTC--GTTTCG-TT-TAGGAGTTA-CCAAGAAATTTCTATATGC-CCA--TG
TCAACTGGTAACAGTTG--TCTGATC--GGGTGATCTACTGTGAGAAAA-TTGTAGTGTT
CAAAGCA--GG-C---GTC---TTTCGCTTGTTCAATGCAGCATGGTATAGTAAAATAAG
ACAC---TAAATATAT-GTTGGTTG-TATA---TTCT--AGTGTAATGACTAATAGGGAA
GGGCGGGGCCGTTCATATTGATGGGCGAGAGGTGAAATTCGTTGACCCTATCAAGATGCA
CTACAGCGAAAGCATTCGGCAAGTACTTCTCCATTAATCAAGAACGAAAGTTTGGGGATC
GAAGACGATCAGATACCGTCGTAGTCCAAACCATAAACTATGTCGACCAGGGATTGGACG
GATAAT-TTTTTAAAAACTCGCTCAGAACCTT-GTGAGAAATCAT-GAGTGTTTGGACTC
TGGGGGGAGTATGGTCGCAAGTCTGAAACTTAAAGGAATTGACGGAAGGGCACACAATGG
AGTGGAA-CCTGCGGCTTAATTT-GACTCAACTCGGGAAA-ACTTACCAAGCTCAGATAT
AATAAGGATTGACAGA-CTAA-AAGATCTTTCATGATTGTATAAGTGGTGGTGCATGGTC
GTTCTTAG-TTGGTGGAGTGATTTGTCTGGTCAATTCCGATAACGGACGAGACCTCTACC
TACTAAATAGTGGTATTTATTTGGTCAACATGGAAGATAGTCATT-TGGTGTTGGC-GTT
AGGT---GTCAAA--GCTTA---GC-GTTCTTCATTGAG-TGGTGTGTATTTCGATCAGA
TAG--GTACTAACTAAAAAA-TA---AA-CTTCTTAGAGGGACTACCT-ACCTCAAGTGG
GGGG-AAGTCGGAGGCAATAACAGGTCTGTGATGCCCTTAGATACCTT-GGGCTGCACGC
GCGTTACAATGTAGACTAGAAAAAGGT----TTCC--------GACATCGA-AAG---GT
GCC-GGTAATCAATTGAATAGTCTACGTAATGGGGATTA-ATTTTTGTAATTATCGATTA
TCAACGAGGAATTCCTTGTAAGCGTAAATCATTACTTTACGCTGAATATGTCCCTGCCCT
TTGTACACACCGCCCGTCGCTCCTACCGATCGAACGATTAGGTAAAACTGACGGACTG--
--AAT-GAACTC--TT---AAC-C-TGG---TTGAT-------CC--GGT-GAACC--TG
-CA-GAAGGAT--C----A---TGCCC
>AY040337.1_Heterostelium_candidum
TCTAAG-TATAA-CCTTTA-TACGGTGAAA----CTGCAGACGGCTCATT--ACAA--CA
---GT----GATAAACTA-AAGAACTTTCGC-GCTTC-----GGCGTCTT-GGATAACCG
CAGTAAATCGGGGCTAATACATGTAAA-CG-AGAGGATGAGCGGG----TAACCGCG-AG
TCTTTGCGATTGTT----A-GCT-TTTACA---CACACCAACCTCTTCGGAGATTGTGGT
GAGTCCGAACAATATTGCTGATCGGAA--------ATT-TATTTCCGACGAGTTCTTTGT
GTCACTGCCCTATCAACTTTCGATGGTAAGGTATTGGCTTACCATGGTTGTAACGGGTGA

CGGGGAATCAGGGTTCGATTCCGGAGAGGGAGCCTGAGAAATGGCTACCACTTCTATGGA
AGGCAGCAGGCGCGCAAATT-ACTCAATCCCAATAC-GGGGAAGTAGTGACAAAAAATAC
TAATGCCTTACCATTTATATGGGGG--GCAATTGGAATAAGTACAACCTAAATCGCTTAG
CA-AAAGTGATTGGAGGGCAAGTCTGGTGCCAGCAGCCGCGGTAATTCCAGCTCCAA-TA
GCGTATACTAAATTTGTTGCAGTTAAAA--GC-TCGTAGTTGAGATTGAGATTTCTTG-G
GT-TTAG--CGTTCATTATTGCCTT--CGGGT-AATA--A-CCGT-TCGTAAAGCT-TCT
AATCGGGATTC--GTTTCG-TT-TAGGAGTTA-CCAAGAAATTTCTATATGC-CCA--TG
TCAACTGGTAACAGTTG--TCTGATC--GGGTGATCTACTGTGAGAAAA-TTGTAGTGTT
CAAAGCA--GG-C---GTC---TTTCGCTTGTTCAATGCAGCATGGTATAGTAAAATAAG
ACAC---TAAATATAT-GTTGGTTG-TATA---TTCT--AGTGTAATGACTAATAGGGAA
GGGCGGGGCCGTTCATATTGATGGGCGAGAGGTGAAATTCGTTGACCCTATCAAGATGCA
CTACAGCGAAAGCATTCGGCAAGTACTTCTCCATTAATCAAGAACGAAAGTTTGGGGATC
GAAGACGATCAGATACCGTCGTAGTCCAAACCATAAACTATGTCGACCAGGGATTGGACG
GATAAT-TTTTTAAAAACTCGCTCAGAACCTT-GTGAGAAATCAT-GAGTGTTTGGACTC
TGGGGGGAGTATGGTCGCAAGTCTGAAACTTAAAGGAATTGACGGAAGGGCACACAATGG
AGTGGAA-CCTGCGGCTTAATTT-GACTCAACTCGGGAAA-ACTTACCAAGCTCAGATAT
AATAAGGATTGACAGA-CTAA-AAGATCTTTCATGATTGTATAAGTGGTGGTGCATGGTC
GTTCTTAG-TTGGTGGAGTGATTTGTCTGGTCAATTCCGATAACGGACGAGACCTCTACC
TACTAAATAGTGGTATTTATTTGGTCAACATGGAAGATAGTCATT-TGGTGTTGGC-GTT
AGGT---GTCAAA--GCTTA---GC-GTTCTTCATTGAG-TGGTGTGTATTTCGATCAGA
TAG--GTACTAACTAAAAAA-TA---AA-CTTCTTAGAGGGACTACCT-ACCTCAAGTGG
GGGG-AAGTCGGAGGCAATAACAGGTCTGTGATGCCCTTAGATACCTT-GGGCTGCACGC
GCGTTACAATGTAGACTAGAAAAAGGT----TTCC--------GACATCGA-AAG---GT
GCC-GGTAATCAATTGAATAGTCTACGTAATGGGGATTA-ATTTTTGTAATTATCGATTA
TCAACGAGGAATTCCTTGTAAGCGTAAATCATTACTTTACGCTGAATATGTCCCTGCCCT
TTGTACACACCGCCCGTCGCTCCTACCGATCGAACGATTAGGTAAAACTGACGGACTG--
--AAT-GACTTC--T----CGC-A-AGG-GATTGTC-------GTT-TGG-AAAG--TTA
-GT-TAAATCT--C----ATT--GTTT
>AM168100.1_Heterostelium_filamentosum_SU_1
TCTAAG-TATAA-CCTTTA-TACGGTGAAA----CTGCAGACGGCTCATT--ACAA--CA
---GT----GATAAACTA-AAGAACTTTCGC-GCTTC-----GGCGTCTT-GGATAACCG
CAGTAAATCGGGGCTAATACATGTAAA-CG-AGAGGATGAGCGGG----CAACCGCG-AG
TCTTTGCGATTGTT----A-GCT-TTTACA---CACACCAACCTCCTCGGAGATTGTGGT
GAGTCCGAACAATATTGCTGATCGGAA--------ATT-TATTTCCGACGAGTTCTTTGT
GTCACTGCCCTATCAACTTTCGATGGTAAGGTATTGGCTTACCATGGTTGTAACGGGTGA
CGGGGAATCAGGGTTCGATTCCGGAGAGGGAGCCTGAGAAATGGCTACCACTTCTATGGA
AGGCAGCAGGCGCGCAAATT-ACTCAATCCCAATAC-GGGGAAGTAGTGACAAAAAATAC
TAATGCCTTACCATTT-TATGGGGG--GCAATTGGAATAAGTACAACCTAAATCGCTTAG
CA-AAAGTGATTGGAGGGCAAGTCTGGTGCCAGCAGCCGCGGTAATTCCAGCTCCAA-TA
GCGTATACTAAATTTGTTGCAGTTAAAA-AGC-TCGTAGTTGAGATTGAGATTTCTTG-G
GT-TTAG--CGTTCATTATTGCCT-CAC-GGT-AATA--A-CCGT-TCGTAAAGCTTT-T
AATCGGGACTC--GTTTCG-TT-TAAGAGTTA-CCAAGAAATTTCTATATGC-CCA--TG
TCAACTGGTAACAGTTG--TCTGATC--GGGTGATCTACTGTGAGAAAA-TTGTAGTGTT
CAAAGCA--GG-C---GTC---TTTCGCTTGTTCAATGCAGCATGGTATAGTAAAATAAG
ACAC---TAAATATAT-GTTGGTTG-TATA---TTCT--AGTGTAATGACTAATAGGGAA
GGGCGGGGCCGTTCATACTGATGGGCGAGAGGTGAAATTCGTTGACCCTATCAAGATGCA
CTACAGCGAAAGCATTCGGCAAGTACTTCTCCATTAATCAAGAACGAAAGTTTGGGGATC
GAAGACGATCAGATACCGTCGTAGTCCAAACCATAAACTATGTCGACCAGGGATTGGACG
GATAAT-TTTTTAAAAACTCGCTCGGAACCTT-GTGAGAAATCAT-GAGTGTTTGGACTC
TGGGGGGAGTATGGTCGCAAGTCTGAAACTTAAAGGAATTGACGGAAGGGCACACAATGG
AGTGGAA-CCTGCGGCTTAATTT-GACTCAACTCGGGAAA-ACTTACCAAGCTCAGATAT
AATAAGGATTGACAGA-CTAA-AAGATCTTTCATGATTGTATAAGTGGTGGTGCATGGTC
GTTCTTAG-TTGGTGGAGTGATTTGTCTGGTCAATTCCGATAACGGACGAGACCTCTACC
TACTAAATAGTGGTATTTATTTGGTCAACATGGAAGATAGTCATT-TGGTGTTGGC-GTT
AGGT---GTCAAA--GCTTA---GC-GTTCTTCATTGAG-TGGTGTGTATTTCGATCAGA
TAG--GTACTAACTAAAAAA-TA---AA-CTTCTTAGAGGGACTACCT-ACCTCAAGTGG
GGGG-GAGTCGGAGGCAATAACAGGTCTGTGATGCCCTTAGATACCTT-GGGCTGCACGC
GCGTTACAATGTAGACTAGAAAAAGGT----TTCC--------GACATCGA-AAG---GT
GCC-GGTAATCAATTGAATAGTCTACGTAATGGGGATTA-ATTTTTGTAATTATCGATTA
TCAACGAGGAATTCCTTGTAAGCGTAAATCATTACTTTACGCTGAATATGTCCCTGCCCT
TTGTACACACCGCCCGTCGCTCCTACCGATCGAACGATTAGGTAAAACTGACGGACTG--
--AAT-GACTTC--T----CGC-A-AGG-GATTGTC-------GTT-TGG-AAAG--TTA
-GT-TAAATCT--C----ATT--GTTT
>AM168101.1_Heterostelium_luridum_LR_2
TCTAAG-TATAA-CCTTTA-TACGGTGAAA----CTGCAGACGGCTCATT--ACAA--CA
---GT----GATAAACTA-AAGAACTTTCGC-GCTTC-----GGCGTCTT-GGATAACCG
CAGTAAATCGGGGCTAATACATGTAAA-CG-AGAGGATGAGCGGG----CAACCGCG-AG
TCTTTGCGATTGTT----A-GCT-TTTACA---CACACCAGCCTCCTCGGAGATTGTGGT
GAGTCCGAACAATATTGCTGATCGGAA--------ATT-TATTTCCGACGAGTTCTTTGT
GTCACTGCCCTATCAACTTTCGATGGTAAGGTATTGGCTTACCATGGTTGTAACGGGTGA
CGGGGAATCAGGGTTCGATTCCGGAGAGGGAGCCTGAGAAATGGCTACCACTTCTATGGA
AGGCAGCAGGCGCGCAAATT-ACTCAATCCCAATAC-GGGGAAGTAGTGACAAAAAATAC
TAATGCCTTACCATTT-TATGGGGG--GCAATTGGAATAAGTACAACCTAAATCGCTTAG

CA-AAAGTGATTGGAGGGCAAGTCTGGTGCCAGCAGCCGCGGTAATTCCAGCTCCAA-TA
GCGTATACTAAATTTGTTGCAGTTAAAA-AGC-TCGTAGTTGAGATTGAGATTTCTTG-G
GT-TTAG--CGTTCATTATTGCCT-CAC-GGT-AATA--A-CCGT-TCGTAAAGCTTT-T
AATCGGGACTC--GTTTCG-TT-TAAGAGTTA-CCAAGAAATTTCTATATGC-CCA--TG
TCAACTGGTAACAGTTG--TCTGATC--GGGTGATCTACTGTGAGAAAA-TTGTAGTGTT
CAAAGCA--GG-C---GTC---TTTCGCTTGTTCAATGCAGCATGGTATAGTAAAATAAG
ACAC---TAAATATAT-GTTGGTTG-TATA---TTCT--AGTGTAATGACTAATAGGGAA
GGGCGGGGCCGTTCATATTGATGGGCGAGAGGTGAAATTCGTTGACCCTATCAAGATGCA
CTACAGCGAAAGCATTCGGCAAGTACTTCTCCATTAATCAAGAACGAAAGTTTGGGGATC
GAAGACGATCAGATACCGTCGTAGTCCAAACCATAAACTATGTCGACCAGGGATTGGACG
GATAAT-TTTTTAAAAACTCGCTCAGAACCTT-GTGAGAAATCAT-GAGTGTTTGGACTC
TGGGGGGAGTATGGTCGCAAGTCTGAAACTTAAAGGAATTGACGGAAGGGCACACAATGG
AGTGGAA-CCTGCGGCTTAATTT-GACTCAACTCGGGAAA-ACTTACCAAGCTCAGATAT
AATAAGGATTGACAGA-CTAA-AAGATCTTTCATGATTGTATAAGTGGTGGTGCATGGTC
GTTCTTAG-TTGGTGGAGTGATTTGTCTGGTCAATTCCGATAACGGACGAGACCTCTACC
TACTAAATAGTGGTATTTATTTGGTCAACATGGAAGATAGTCATT-TGGTGTTGGC-GTT
AGGT---GTCAAA--GCTTA---GC-GTTCTTCATTGAG-TGGTGTGTATTTCGATCAGA
TAG--GTACTAACTAAAAAA-TA---AA-CTTCTTAGAGGGACTACCT-ACCTCAAGTGG
GGGG-AAGTCAGAGGCAATAACAGGTCTGTGATGCCCTTAGATACCTT-GGGCTGCACGC
GCGTTACAATGTAGACTAGAAAAAGGT----TTCC--------GACATCGA-AAG---GT
GCC-GGTAATCAATTGAATAGTCTACGTAATGGGGATTA-ATTTTTGTAATTATCGATTA
TCAACGAGGAATTCCTTGTAAGCGTAAATCATTACTTTACGCTGAATATGTCCCTGCCCT
TTGTACACACCGCCCGTCGCTCCTACCGATCGAACGATTAGGTAAAACTGACGGACTG--
--AAT-GACTTC--T----CGC-A-AGG-GATTGTC-------GTT-TGG-AAAG--TTA
-GT-TAAATCT--C----ATT--GTTT
>MN338953.1_Heterostelium_radiatum_M26B
TCTAAG-TATAA-CCTTTA-TACGGTGAA-----CTG-AGAGGGACACGC---CGG--CA
---GT---AGATAAACTA-AAGAACTTTCGC-GCTTC-----GGCGTCTT-GGATAACCG
CAGTAAATCGGGGCTAATACATATAAA-CG-AGAGGATGAGCAGG----CAACTGCG-AG
TCTTTGCGATTGTT----A-GCTATCTTTTT--CACACCAACCTCCTCGGAGTTTGTGGT
GAATCCGAACAATATTGCTGATCGAAA--------ATT-TATTTTCGACGAGTTCTTTGT
GTCACTGCCCTATCAACTTTCGATGGTAAGGTATTGGCTTACCATGGTTGTAACGGGTGA
CGGGGAATCAGGGTTCGATTCCGGAGAGGGAGCCTGAGAAATGGCTACCACTTCTACGGA
AGGCAGCAGGCGCGCAAATT-ACTCAATCCCAATAC-GGGGAAGTAGTGACAAAAAATAC
TAATGCCTTTCCATAT-TATGGGGG--GCAATTGGAATAAGTACAACTTAAATCGCTTAG
CA-AAAGTGATTGGAGGGCAAGTCTGGTGCCAGCAGCCGCGGTAATTCCAGCTCCAA-TA
GCGTATACTAAATTTGTTGCAGTTAAAA-AGC-TCGTAGTTGAGATTGAGATTTCTTG-G
GT-TTAAGCCAGTTATAGTAGCTTT--CGGGTTATTATAA-T--TCGGTTAAAGCTCTTG
AGTGGATTTTA-TTTTTCA-CTTTTAGAGTTA-CCAAGGAATTTCCAACTGC-CCA--TG
TAGACTGGCAACAGTTT--AC-AATC--GGGTGATCTACTGTGAGAAAA-TTGTAGTGTT
CAAAGCA--GG-C---GTC---TTAAGCTTGTTCAATGCAGCATGGTATAGTAAAATATG
ACAC---TAAATATAT-GTTGGTTG-TATA---TTCT-TAGTGTAATGACTAATAGGGAA
GGGCGGGGCCGTTCATATTGATGGGCGAGAGGTGAAATTCGTTGACCCTATCAAGATGCA
CTACAGCGAAAGCATTCGGCAAGTACTTCTCCATTAATCAAGAACGAAAGTTTGGGGATC
GAAGACGATCAGATACCGTCGTAGTCCAAACCATAAACTATGTCGACCAGGGATTGGACG
GATAAT-TTTTTAAAAACTCGCTCAGAACCTT-GTGAGAAATCAT-GAGTGTTTGGACTC
TGGGGGGAGTATGGTCGCAAGTCTGAAACTTAAAGGAATTGACGGAAGGGCACACAATGG
AGTGGAA-CCTGCGGCTTAATTT-GACTCAACTCGGGAAA-ACTTACCAAGCTCAGATAT
AATAAGGATTGACAGA-CTAATAAGATCTTTCATGATTGTATAAGTGGTGGTGCATGGTC
GTTCTTAG-TTGGTGGAGTGATTTGTCTGGTCAATTCCGATAACGGACGAGACCTCTACC
TGCTAACTAGTGGTATTTATTTGGTCAATATGGAAGATAGTCATT-TGGTGTTGGT-GTT
AGGT---GTCAAA--GCTTA---GC-ATTCTTCATTGAG-TGGTGTGTATTCTGGTCAGA
TAG--GTACTAACTAAAAAA-TA---AA-CTTCTTAGAGGGACTACCT-ACCTCAAGTGG
GGGG-AAGTCGGAGGCAATAACAGGTCTGTGATGCCCTTAGATACCTT-GGGCTGCACGC
GCGTTACAATGTAGATGAGAAAAAGGT----TTCC--------GACGTCGA-AAG---GC
GCC-GGTAATCAATTGAATTGTCTACGTAATGGGGATTA-ATTTTTGTAATTATCGATTA
TCAACGAGGAATTCCTTGTAAGCGTAAATCATTACTTTGCGCTGAATATGTCCCTGCCCT
TTGTACACACCGCCCGTCGCTCCTACCGATCGAACGATTAGGTAAAACTGACGGATTA--
--GAT-GATTTTC-TGGTGAAC-CCTGG-TTTGGAT------TCC---CG-C--CCGTTG
--T-T----C---C----ATTATGCC-
>KP167478.1_Heterostelium_violaceotypum
TCTAAG-TATAA-CCTTTA-TACGGTGAAA----CTGCAGACGGCTCAAT--ACAA--CA
---GT----GATAAACTA-AAGAACTTTCGC-GCTTC-----GGCGTCTT-GGATAACCG
CAGTAAATCGGGGCTAATACATATAAA-CG-AGAGGATGAGCAGG----CAACTGCG-AG
TCTTTGCGATTGTT----A-GCTATCTTTT---CACACCAACCTCTTCGGAGTTTGTGGT
GAATCCGAACAATATTGCTGATCGAAA--------ATT-TATTTTCGACGAGTTCTTTGT
GTCACTGCCCTATCAACTTTCGATGGTAAGGTATTGGCTTACCATGGTTGTAACGGGTGA
CGGGGAATCAGGGTTCGATTCCGGAGAGGGAGCCTGAGAAATGGCTACCACTTCTACGGA
AGGCAGCAGGCGCGCAAATT-ACTCAATCCCAATAC-GGGGAAGTAGTGACAAAAAATAC
TAATGCCTTTCCATAT-TATGGGGG--GCAATTGGAATAAGTACAACTTAAATCGCTTAG
CA-AAAGTGATTGGAGGGCAAGTCTGGTGCCAGCAGCCGCGGTAATTCCAGCTCCAA-TA
GCGTATACTAAATTTGTTGCAGTTAAAA-AGC-TCGTAGTTGAGATTGAGATTTCTTG-G
GT-TTAAGCCAGTCATAGTAGCTTT--CGGGTTATTATGG-T--TCGGTTAAAGCTTTTG

AGTGGATTTTA-TTTTTCA-CTTTTAAAGTTA-CCAAGGAATTTCCAACTGC-CCA--TG
TAGACTGGCAACAGTTT--AC-AATC--GGGTGATCTACTGTGAGAAAA-TTGTAGTGTT
CAAAGCA--GG-C---GTC---TTACGTTTGTTCAATGCAGCATGGTATAGTAAAATATG
ACAC---TAAATATAT-GTTGGTTG-TATA---TTCT-TAGTGTAATGACTAATAGGGAA
GGGCGGGGCCGTTCATATTGATGGGCGAGAGGTGAAATTCGTTGACCCTATCAAGATGCA
CTACAGCGAAAGCATTCGGCAAGTACTTCTCCATTAATCAAGAACGAAAGTTTGGGGATC
GAAGACGATCAGATACCGTCGTAGTCCAAACCATAAACTATGTCGACCAGGGATTGGACG
GATAAT-TTTTTAAAAACTCGCTCAGAACCTT-GTGAGAAATCAT-GAGTGTTTGGACTC
TGGGGGGAGTATGGTCGCAAGTCTGAAACTTAAAGGAATTGACGGAAGGGCACACAATGG
AGTGGAA-CCTGCGGCTTAATTT-GACTCAACTCGGGAAA-ACTTACCAAGCTCAGATAT
AATAAGGATTGACAGG-CTAA-AAGATCTTTCATGATTGTATAAGTGGTGGTGCATGGTC
GTTCTTAG-TTGGTGGAGTGAATTGTCTGGTCAATTCCGATAACGGACGAGACCTCTACC
TGCTAACTAGTGGTATTTATTTGGTCAATATGGAAGATAGTCATT-TGGTGTTGGT-GTC
AGGT---GTCAAA--GCTTG---GC-ATTCTTCATTGAG-TGGTGTGTATTCTGGTCAGA
TAG--GTACTAACTAAAAACATA---A--CTTCT-AGAGGGACTACC--ACCTCA-GTGG
GGGG-AA-TCG-AAGC-ATAACAGG-CTGTGA-GCCCT-AAA-ACCTT-GG-CTGC-CCC
GCGT-ACAATG-AGATGA-AAAAAGG-----TTCC--------GAC-TCGA-A-G---GT
GCC-G-TA-TCAAT-GGATTG-CTACG-AATGGGGAT-A-ATTTT-G-AA-TATCGAT-A
TCA-CGAGGA-TTC-TTG-AAGCGTAA--------------------------------

>HQ141507.1_Heterostelium_stolonicoideum_K12A
TCTAAG-TATAA-CCTTTA-TACGGTGAAC----CTGCAGAAGGATCAACTCACGG--CT
--TGT----GATAAACTA-AAGAACTTCCGC-GCTTC-----GGCGTCTT-GGATAACCG
CAGTAAATCGGGGCTAATACATATAAA-CG-AGAGGGTGAGCGGG----CAACTGCG-AA
CCTTTGCGATTGTT----A-GCTATCA-CT---CA--CCAACCTCTTCGGAGTTTGTGGT
GAATCCGAACAATATTGCTGATCGAAA--------ATT-TATTTTCGACGAGTTCTTTGT
GTCACTGCCCTATCAACTTTCGATGGTAAGGTATTGGCTTACCATGGTTGTAACGGGTGA
CGGGGAATCAGGGTTCGATTCCGGAGAGGGAGCCTGAGAAATGGCTACCACTTCTACGGA
AGGCAGCAGGCGCGCAAATT-ACTCAATCCCAATAC-GGGGAAGTAGTGACAAAAAATAC
TAATGCCTTTCCATAT-TATGGGGG--GCAATTGGAATAAGTACAACTTAAATCGCTTAG
CA-AAAGTGATTGGAGGGCAAGTCTGGTGCCAGCAGCCGCGGTAATTCCAGCTCCAA-TA
GCGTATACTAAATTTGTTGCAGTTAAAA-AGC-TCGTAGTTGAGATTGAGATTTCTTG-G
GT-TTAAGCCAGTCATAGTAGCTTT--CGGGTTATTATGA-T-TTCGGTTAAAGCTTTTG
AGTGGATTTTA-TTTTTCA-CTTTTAAAGTTA-CCAAGGGATTTCCAACTGC-CCA--TG
TAAGCTGGCAACAGTTT--AC-AATC--GGGTGATCTACTGTGAGAAAA-TTGTAGTGTT
CAAAGCA--GG-C---GTC---TTACGTTTGTTCAATGCAGCATGGTATAGTAAAATATG
ACAC---TAAATATAT-GTTGGTTG-TATA---TTCT-TAGTGTAATGACTAATAGGGAA
GGGCGGGGCCGTTCATATTGATGGGCGAGAGGTGAAATTCGTTGACCCTATCAAGATGCA
CTACAGCGAAAGCATTCGGCAAGTACTTCTCCATTAATCAAGAACGAAAGTTTGGGGATC
GAAGACGATCAGATACCGTCGTAGTCCAAACCATAAACTATGTCGACCAGGGATTGGACG
GATAAT-TTTTTAAAAACTCGCTCAGAACCTT-GTGAGAAATCAT-GAGTGTTTGGACTC
TGGGGGGAGTATGGTCGCAAGTCTGAAACTTAAAGGAATTGACGGAAGGGCACACAATGG
AGTGGAA-CCTGCGGCTTAATTT-GACTCAACTCGGGAAA-ACTTACCAAGCTCAGATAT
AATAAGGATTGACAGA-CTAA-AAGATCTTTCATGATTGTATAAGTGGTGGTGCATGGTC
GTTCTTAG-TTGGTGGAGTGATTTGTCTGGTCAATTCCGATAACGGACGAGACCTCTACC
TGCTAACTAGTGGTATTTATTTGGTCAATATGGAAGATAGTCATT-TGGTGTTGGT-GTC
AGG----GTCAAA---CTTG---GC-ATTCTTCATTGAG-TGGTGTGTATTCTGGTCAGA
TAG--GTACTAACTAAAAAAATA---AA-CTTCTTAGAGGGACTACCT-ACCTCAAGTGG
GGGG-AAGTCGGAGGCAATAACAGGTCTGTGATGCCCTTAGATACCTT-GGGCTGCACGC
GCGTTACAATGTAGATGAGAAAAAGGT----TTCC--------GACATCGA-AAG---GT
GCC-GGTAATCAATTGAATTGTCTACGTAATGGGGATTA-ATTTTTGTAATTATCGATTA
TCAACGAGGAATTCCTTGTAAGCGTAAATCATTACTTTACGCTGAATATGTCCCTGCCCT
TTGTACACACCGCCCGTCGCTCCTACCGATCGAACGATTAGGTAAAACTGACGGATTA--
--GAT-GATTTC--CT---AAC-C-TGG---TTGAT-------CCT-GGT-GAACC--TG
-CA-GAAGGAT--C----A----GCC-
>AM168103.1_Heterostelium_pallidum_TNS_C_98
TCTAAG-TATAA-CCTTTA-TACGGTGAAA----CTGCAGACGGCTCATT--ACAA--CA
---GT----GATAAACTA-AAGAACTTCCGC-GCTTC-----GGCGTCTT-GGATAACCG
CAGTAAATCGGGGCTAATACATATAAA-CG-AGAGGGTGAGCGGG----CAACTGCG-AA
CCTTTGCGATTGTT----A-GCTATCTTTTT--CA--CCAACCTCTTCGGAGTTTGTGGT
GAATCCGAACAATATTGCTGATCGAAA--------ATT-TATTTTCGACGAGTTCTTTGT
GTCACTGCCCTATCAACTTTCGATGGTAAGGTATTGGCTTACCATGGTTGTAACGGGTGA
CGGGGAATCAGGGTTCGATTCCGGAGAGGGAGCCTGAGAAATGGCTACCACTTCTACGGA
AGGCAGCAGGCGCGCAAATT-ACTCAATCCCAATAC-GGGGAAGTAGTGACAAAAAATAC
TAATGCCTTTCCATAT-TATGGGGG--GCAATTGGAATAAGTACAACTTAAATCGCTTAG
CA-AAAGTGATTGGAGGGCAAGTCTGGTGCCAGCAGCCGCGGTAATTCCAGCTCCAA-TA
GCGTATACTAAATTTGTTGCAGTTAAAA-AGC-TCGTAGTTGAGATTGAGATTTCTTG-G
GT-TTAAGCCAGTCATAGTAGCTTT--CGGGTTATTATGA-T-TTCGGTTAAAGCTTTTG
AGTGGATTTTA-TTTTTCA-CTTTTAAAGTTA-CCAAGGGATTTCCAACTGC-CCA--TG
TAAGCTGGCAACAGTTT--AC-AATC--GGGTGATCTACTGTGAGAAAA-TTGTAGTGTT
CAAAGCA--GG-C---GTC---TTACGTTTGTTCAATGCAGCATGGTATAGTAAAATATG

ACAC---TAAATATAT-GTTGGTTG-TATA---TTCT-TAGTGTAATGACTAATAGGGAA
GGGCGGGGCCGTTCATATTGATGGGCGAGAGGTGAAATTCGTTGACCCTATCAAGATGCA
CTACAGCGAAAGCATTCGGCAAGTACTTCTCCATTAATCAAGAACGAAAGTTTGGGGATC
GAAGACGATCAGATACCGTCGTAGTCCAAACCATAAACTATGTCGACCAGGGATTGGACG
GATAAT-TTTTTAAAAACTCGCTCAGAACCTT-GTGAGAAATCAT-GAGTGTTTGGACTC
TGGGGGGAGTATGGTCGCAAGTCTGAAACTTAAAGGAATTGACGGAAGGGCACACAATGG
AGTGGAA-CCTGCGGCTTAATTT-GACTCAACTCGGGAAA-ACTTACCAAGCTCAGATAT
AATAAGGATTGACAGA-CTAA-AAGATCTTTCATGATTGTATAAGTGGTGGTGCATGGTC
GTTCTTAG-TTGGTGGAGTGATTTGTCTGGTCAATTCCGATAACGGACGAGACCTCTACC
TGCTAACTAGTGGTATTTATTTGGTCAATATGGAAGATAGTCATT-TGGTGTTGGT-GTC
AGG----GTCAAA---CTTG---GC-ATTCTTCATTGAG-TGGTGTGTATTCTGGTCAGA
TAG--GTACTAACTAAAAAAATA---AA-CTTCTTAGAGGGACTACCT-ACCTCAAGTGG
GGGG-AAGTCGGAGGCAATAACAGGTCTGTGATGCCCTTAGATACCTT-GGGCTGCACGC
GCGTTACAATGTAGATGAGAAAAAGGT----TTCC--------GACATCGA-AAG---GT
GCC-GGTAATCAATTGAATTGTCTACGTAATGGGGATTA-ATTTTTGTAATTATCGATTA
TCAACGAGGAATTCCTTGTAAGCGTAAATCATTACTTTACGCTGAATATGTCCCTGCCCT
TTGTACACACCGCCCGTCGCTCCTACCGATCGAACGATTAGGTAAAACTGACGGATTA--
--GAT-GATTTC--CT---CGC-A-AGG-GGTTATC-------GTT-TGA-GAAG--TTA
-GT-TAAATCT--C----ATT--GTTT
>AM168102.1_Heterostelium_arachnoideum_YA1
TCTAAG-TATAA-CCTTTA-TACGGTGAAA----CTGCAGACGGCTCATT--ACAA--CA
---GT----GATAAACTA-AAGAACTTTCGC-GCTTC-----GGCGTCTT-GGATAACCG
CAGTAAATCGGGGCTAATACATGTAAA-CG-AGAGGATGAGCGGG----CAACTGCG-AG
TCTTTGCGATTGTT----A-GCT-TTTATT---CACACCAACCTCTTCGGAGTTTGTGGT
GAGTCCGAACAATATTGCTGATCGGAA--------ATT-TATTTCCGACGAGTTCTTTGT
GTCACTGCCCTATCAACTTTCGATGGTAAGGTATTGGCTTACCATGGTTGTAACGGGTGA
CGGGGAATCAGGGTTCGATTCCGGAGAGGGAGCCTGAGAAATGGCTACCACTTCTATGGA
AGGCAGCAGGCGCGCAAATT-ACTCAATCCCAATAC-GGGGAAGTAGTGACAAAAAATAC
TAATGCCTTTCCATTTATATGGGGG--GCAATTGGAATAAGTACAACTTAAATCGCTTAG
CA-AAAGTGATTGGAGGGCAAGTCTGGTGCCAGCAGCCGCGGTAATTCCAGCTCCAA-TA
GCGTATACTAAATTTGTTGCAGTTAAAA-AGC-TCGTAGTTGAGATTGAGATTTCTTG-G
GT-TTAG--CATGGACTATGGCCT-CAC-GGTTTATA--A-T-TT-ATGTCAAGCTTTAT
AGTAAGATTTT-ACTCTTA-TTATTTAAGTTA-CCAAGAAATTTCCAACTGC-CCA--TG
CCAGTTAGCAATAATTG--GCTGATC--GGGTGATCTACTGTGAGAAAA-TTGTAGTGTT
CAAAGCA--GG-C---GTC---TTTCGCTTGTTCAATGCAGCATGGTATAGTAAAATATG
ACAC---TAAACATAT-GTTGGTTG-TATG---TA-T-TAGTGTAATGACTAATAGGGAA
GGGCGGGGCCGTTCATATTGATGGGCGAGAGGTGAAATTCGTTGACCCTATCAAGATGCA
CTACAGCGAAAGCATTCGGCAAGTACTTCTCCATTAATCAAGAACGAAAGTTTGGGGATC
GAAGACGATCAGATACCGTCGTAGTCCAAACCATAAACTATGTCGACCAGGGATTGGACG
GATAAT-TTTTTAAAAACTCGCTCAGAACCTT-GTGAGAAATCAT-GAGTGTTTGGACTC
TGGGGGGAGTATGGTCGCAAGTCTGAAACTTAAAGGAATTGACGGAAGGGCACACAATGG
AGTGGAA-CCTGCGGCTTAATTT-GACTCAACTCGGGAAA-ACTTACCAAGCTCAGATAT
AATAAGGATTGACAGA-CTAA-AAGATCTTTCATGATTGTATAAGTGGTGGTGCATGGTC
GTTCTTAG-TTGGTGGAGTGATTTGTCTGGTCAATTCCGATAACGGACGAGACCTCTACC
TGCTAACTAGTGGTATTTATTTGGTCAA-ATGGGAGATAGTCATT-TGGTGTTGGCAGTT
AGGC--CGTTAAAA-GTTTA---ATTGTCCTTCATTGAG-TGGTGTGTATCCTGATCAGA
TAG--GTACTAACTAAAAAAATA---AA-CTTCTTAGAGGGACTACCT-ACCTCAAGTGG
GGGG-AAGTCGGAGGCAATAACAGGTCTGTGATGCCCTTAGATACCTT-GGGCTGCACGC
GCGTTACAATGTAGGCGAGAAAAAGGT----TTCC--------GACATCGA-AAG---GT
GCC-GGTAATCAATTGAATTGCCTACGTAATGGGGATTA-ATTTTTGTAATTATCGATTA
TCAACGAGGAATTCCTTGTAAGCGTAAATCATTACTTTGCGCTGAATATGTCCCTGCCCT
TTGTACACACCGCCCGTCGCTCCTACCGATCGAACGATTAGGTAAAACTGACAGACTG--
--GAC-GACTTC--T----CGC-A-AGG-GATTGTT-------GTT-TGG-AAAG--TTA
-GT-TAAATCT--C----ATT--GTTT
>HQ141508.1_Heterostelium_australicum_NB1AP
TCTAAG-TATAA-CCTTTA-TACGGTGAAC----CTGCAGAAGGATCAACT-ACGA--CT
-ATGT----GATAAACTA-AAGAACTTTCGC-GCTTC-----GGCGTCTT-GGATAACCG
CAGTAAATCGGGGCTAATACATGTAAA-CG-AGAGGATGAGCGGG----CAACTGCG-AG
TCTTTGCGATTGTT----A-GCT-TTTATT---CACACCAACCTCTTCGGAGTTTGTGGT
GAGTCCGAACAATATTGCTGATCGGAA--------ATT-TATTTCCGACGAGTTCTTTGT
GTCACTGCCCTATCAACTTTCGATGGTAAGGTATTGGCTTACCATGGTTGTAACGGGTGA
CGGGGAATCAGGGTTCGATTCCGGAGAGGGAGCCTGAGAAATGGCTACCACTTCTATGGA
AGGCAGCAGGCGCGCAAATT-ACTCAATCCCAATAC-GGGGAAGTAGTGACAAAAAATAC
TAATGCCTTTCCATTTATATGGGGG--GCAATTGGAATAAGTACAACTTAAATCGCTTAG
CA-AAAGTGATTGGAGGGCAAGTCTGGTGCCAGCAGCCGCGGTAATTCCAGCTCCAA-TA
GCGTATACTAAATTTGTTGCAGTTAAAA-AGC-TCGTAGTTGAGATTGAGATTTCTTG-G
GT-TTAG--CATTGATTATGACTTT--CGGGTTTATA--A-T-TT-ATGTCAAGCTTTAT
AATAAGATTTC-ACTCTTG-TTATTTAAGTTA-CCAAGAAATTTCCAACTGC-CCA--TG
CCAGTTAGCAATAATTG--GCTGATC--GGGTGATCTACTGTGAGAAAA-TTGTAGTGTT
CAAAGCA--GG-C---GTC---TTTCGCTTGTTCAATGCAGCATGGTATAGTAAAATATG
ACAC---TAAACATAT-GTTGGTTG-TATG---TA-T-TAGTGTAATGACTAATAGGGAA
GGGCGGGGCCGTTCATATTGATGGGCGAGAGGTGAAATTCGTTGACCCTATCAAGATGCA
CTACAGCGAAAGCATTCGGCAAGTACTTCTCCATTAATCAAGAACGAAAGTTTGGGGATC

GAAGACGATCAGATACCGTCGTAGTCCAAACCATAAACTATGTCGACCAGGGATTGGACG
GATAAT-TTTTTAAAAACTCGCTCAGAACCTT-GTGAGAAATCAT-GAGTGTTTGGACTC
TGGGGGGAGTATGGTCGCAAGTCTGAAACTTAAAGGAATTGACGGAAGGGCACACAATGG
AGTGGAA-CCTGCGGCTTAATTT-GACTCAACTCGGGAAA-ACTTACCAAGCTCAGATAT
AATAAGGATTGACAGA-CTAA-AAGATCTTTCATGATTGTATAAGTGGTGGTGCATGGTC
GTTCTTAG-TTGGTGGAGTGATTTGTCTGGTCAATTCCGATAACGGACGAGACCTCTACC
TGCTAACTAGTGGTATTTATTTGGTCAATACGGGAGATAGTCATT-TGGTGTTGGCAGTT
AGAC--CCTTAAAA-GTTTA---ATTGTCCTTCATTGAG-TGGTGTGTATCCTGATCAGA
TAG--GTACTAACTAAAAAA-TA---AA-CTTCTTAGAGGGACTACCT-ACCTCAAGTGG
GGGG-AAGTCGGAGGCAATAACAGGTCTGTGATGCCCTTAGATACCTT-GGGCTGCACGC
GCGTTACAATGTAGACGAGAAAAAGGT----TTCC--------GACATCGA-AAG---GT
GCC-GGTAATCAATTGAATTGTCTACGTAATGGGGATTA-ATTTTTGTAATTATCGATTA
TCAACGAGGAATTCCTTGTAAGCGTAAATCATTACTTTGCGCTGAATATGTCCCTGCCCT
TTGTACACACCGCCCGTCGCTCCTACCGATCGAACGATTAGGTAAAACTGACAGACTG--
--GAC-GACTTC--T----AAC-C-TGG---TTGAT-------CCT-GGT-GAACC--TG
-CA-GAAGGAT--C----A----GCC-
>AM168099.1_Heterostelium_equisetoides_B7JB
TCTAAG-TATAA-CCTTTA-TACGGTGAAA----CTGCAGACGGCTCATT--ACAA--CA
---GT----GATAAACTA-AAGAACTTTCGC-GCTTC-----GGCGTCTT-GGATAACCG
CAGTAAATCGGGGCTAATACATGTAAA-CG-AGAGGATAAGCGGG----CAACTGCG-AG
TCTTTGCGATTGTT----A-GCT-TTTATT---CACACCAACCTCTTCGGAGTTTGTGGT
GAGTCCGAACAATATTGCTGATCGGAA--------ACT-TGTTTCCGACGAGTTCTTTGT
GTCACTGCCCTATCAACTTTCGATGGTAAGGTATTGGCTTACCATGGTTGTAACGGGTGA
CGGGGAATCAGGGTTCGATTCCGGAGAGGGAGCCTGAGAAATGGCTACCACTTCTATGGA
AGGCAGCAGGCGCGCAAATT-ACTCAATCCCAATAC-GGGGAAGTAGTGACAAAAAATAC
TAATGCCTTTCCCTTTATAGGGGGG--GCAATTGGAATAAGTACAACTTAAATCGCTTAA
CA-AAAGTGATTGGAGGGCAAGTCTGGTGCCAGCAGCCGCGGTAATTCCAGCTCCAA-TA
GCGTATACTAAATTTGTTGCAGTTAAAA-AGC-TCGTAGTTGAGATTGAGATTTCTTG-G
GT-TTAG--CGTATTTTATTGCCTT--CGGGTTTATA--A-T--T-GCGTAAAGCTTTTT
AATAGGAT-TC-ACTCTTA-TT-TTAAAGTTA-CCAAGAAATTTCCAACTGC-CCA--TG
CCAGTTAGCAATAATTG--GCTGATC--GGGTGATCTACTGTGAGAAAA-TTGTAGTGTT
CAAAGCA--GG-C---GTT---TTAAGCTTGTTCAATGCAGCATGGTATAGTAAAATATG
ACAC---TAAACATAT-GTTGGTAG-TATG---TA-T-TAGTGTAATGACTAATAGGGAA
GGGCGGGGCCGTTCATATTGATGGGCGAGAGGTGAAATTCGTTGACCCTATCAAGATGCA
CTACAGCGAAAGCATTCGGCAAGTACTTCTCCATTAATCAAGAACGAAAGTTTGGGGATC
GAAGACGATCAGATACCGTCGTAGTCCAAACCATAAACTATGTCGACCAGGGATTGGACG
GATAAT-TTTTTAAAAACTCGCTCAGAACCTT-GTGAGAAATCAT-GAGTGTTTGGACTC
TGGGGGGAGTATGGTCGCAAGTCTGAAACTTAAAGGAATTGACGGAAGGGCACACAATGG
AGTGGAA-CCTGCGGCTTAATTT-GACTCAACTCGGGAAA-ACTTACCAAGCTCAGATAT
AATAAGGATTGACAGA-CTAA-AAGATCTTTCATGATTGTATAAGTGGTGGTGCATGGTC
GTTCTTAG-TTGGTGGAGTGATTTGTCTGGTCAATTCCGATAACGGACGAGACCTCTACC
TGCTAACTAGTGGTATTTATTTGGTCAATACGGGAGATAGTTATT-TGGTGTTGGCAGTT
AGGC--CGTTAAAA-GTTTA---ATTGTCCTTCATTGAG-TAGTGTGTATCCTGATCAGA
TAG--GTACTAACTAAAAAAATA---AA-CTTCTTAGAGGGACTACCT-ACCTCAAGTGG
GGGG-AAGTCGGAGGCAATAACAGGTCTGTGATGCCCTTAGATACCTT-GGGCTGCACGC
GCGTTACAATGTAGGCGAGAAAAAGGT----TTCC--------GACATCGA-AAG---GT
GCC-GGTAATCAATTGAATTGCCTACGTAATGGGGATTA-ATTTTTGTAATTATCGATTA
TCAACGAGGAATTCCTTGTAAGCGTAAATCATTACTTTGCGCTGAATATGTCCCTGCCCT
TTGTACACACCGCCCGTCGCTCCTACCGATCGAACGATTAGGTAAAACTGACAGACTG--
--GAT-GACTTC--T----CGC-A-AGG-GATTGTC-------GTT-TGG-AAAG--TTA
-GT-TAAATCT--C----ATT--GTTT
>HQ141506.1_Heterostelium_multicystogenum_AS2
TCTAAG-TATAA-CCTTTA-TACGGTGAAC----CTGCAGAAGGATCAACT-ACAAG-CA
--TGT---AGATAAACTA-AAGAACTTTCGC-GCTTC-----GGCGTTTT-GGATAACCG
CAGTAAATCGGGGCTAATACATATAAA-CG-AGAGGATGAGCGGG----CAACTGCG-AG
TCTTTGCGATTGTT----A-GCT-TTT-T----CA--CCAATCTCTTCGGAGTTTGTGGT
GAATCCCTACAATATTGCTGATCGGAA--------ACT-TGTTTCCGACGAGTTCTTTGT
GTCACTGCCCTATCAACTTTCGATGGTAAGGTATTGGCTTACCATGGTTGTAACGGGTGA
CGGGGAATCAGGGTTCGATTCCGGAGAGGGAGCCTGAGAAATGGCTACCACTTCTACGGA
AGGCAGCAGGCGCGCAAATT-ACTCAATCCCAATAC-GGGGAAGTAGTGACAAAAAATAC
TAATGCCTTTCCATTTATATGGGGG--GCAATTGGAATAAGTACAACTTAAATCGCTTAA
CA-AAAGTGATTGGAGGGCAAGTCTGGTGCCAGCAGCCGCGGTAATTCCAGCTCCAA-TA
GCGTATACTAAATTTGTTGCAGTTAAAA-AGC-TCGTAGTTGAGATTGAGATTCTTTG-G
GT-TTAA--CAGTCAGCATTGCCTTCACCGGTTAATGCTT-TGATCT-GTAAAGCTTTAT
AGCAGAATTTC-ATTTT-G-TTATTAAAGTTA-CCAAAGAATTTCTATCTGC-CCA--TG
GTAGCCAGCAATGGTTA--TC-AATC--GGGTGATCTACTGTGAGAAAA-TTGTAGTGTT
CAAAGCA--GG-C---GTC---TTACGTTTGTTCAATGCAGCATGGTATAGTAAAATATG
ACAC---TTAACATAT-GTTGGTTG-TATG---TT-T-TAGTGTAATGACTAATAGGGAA
GGGCGGGGCCGTTCATATTGATGGGCGAGAGGTGAAATTCGTTGACCCTATCAAGATGCA
CTACAGCGAAAGCATTCGGCAAGTACTTCTCCATTAATCAAGAACGAAAGTTTGGGGATC
GAAGACGATCAGATACCGTCGTAGTCCAAACCATAAACTATGTCGACCAGGGATTGGACG
GATAAT-TTTTTAAAAACTCGCTCAGAACCTT-GTGAGAAATCAT-GAGTGTTTGGACTC
TGGGGGGAGTATGGTCGCAAGTCTGAAACTTAAAGGAATTGACGGAAGGGCACACAATGG

AGTGGAA-CCTGCGGCTTAATTT-GACTCAACTCGGGAAA-ACTTACCAAGCTCAGATAT
AATAAGGATTGACAGA-CTAA-AAGATCTTTCATGATTGTATAAGTGGTGGTGCATGGTC
GTTCTTAG-TTGGTGGAGTGATTTGTCTGGTCAATTCCGATAACGGACGAGACCTCTACT
TACTAACTAGTGGTATTTATTTGGTCAATACGGGAGATAGTCATT-TGGTGTTGGTAGT-
AGGA--CGTCAAAA-TCTTA----TTATTCTTCATTGAG-TGGTGTGTATCCTGATCAGA
TAG--GTACTAACTTAAAAAATA---AA-CTTCTTAGAAGGACTACCT-ACCTCAAGTGG
GGGG-AAGTCGGAGGCAATAACAGGTCTGTGATGCCCTTAGATACCTT-GGGCTGCACGC
GCGTTACAATGTAGGCGAGAAAAAGGT----TTCC--------GGAATCGA-AAG---GT
TTC-GGTAATCATTTGAATTGCCTACGTAATGGGGATTA-ATTTTTGTAATTATCGATTA
TCAACGAGGAATTCCTTGTAAGCGTAAATCATTACTTTACGCTGAATATGTCCCTGCCCT
TTGTACACACCGCCCGTCGCTCCTACCGATCGAACGATTAGGTAAAACTGACGGACTA--
--GGC-GACTTT--T----AAC-CATGG---TTGAT-------CC--GGT-GAACC--TG
-CA-GAAGGAT--C----A---TGCC-
>HQ141496.1_Heterostelium_sp._Laos3
TCTAAG-TATAA-CCTTTA-TACGGTGAAC----CTGCAGAAGGATCATCTCACGAG-CA
-TAGT---AGATAAACTA-AAGAACTTTCGC-GCTTC-----GGCGTCTT-GGATAACCG
CAGTAAATCGGGGCTAATACATGTAAA-CG-AGAGGATGAGCAGG----CAACTGCG-AG
TCTTTGCGATTGTT----A-GCT-TTCATT---CA--CCAACCTCTTCGGAGTTTGTGGT
GAGTCCGAACAATATTGCTGATCGGAA--------ACT-TGTTTCCGACGAGTTCTTTGT
GTCACTGCCCTATCAACTTTCGATGGTAAGGTATTGGCTTACCATGGTTGTAACGGGTGA
CGGGGAATCAGGGTTCGATTCCGGAGAGGGAGCCTGAGAAATGGCTACCACTTCTACGGA
AGGCAGCAGGCGCGCAAATT-ACTCAATCCCAATAC-GGGGAAGTAGTGACAAAAAATAC
TAATGCCTTTCCATTTATATGGGGG--GCAATTGGAATAAGTACAACTTAAATCGCTTAG
CA-AAAGTGATTGGAGGGCAAGTCTGGTGCCAGCAGCCGCGGTAATTCCAGCTCCAA-TA
GCGTATACTAAATTTGTTGCAGTTAAAA-AGC-TCGTAGTTGAGATTGAGATTTCTTA-G
GT-TTAG---AGTCACC-TGGCCTT--CGGGTT-CAG-GT-TG-TCTCGTAAAGCTTTAT
AATGAAATTCC-ATTTT-G-TTATTAAAGTCA-CTAAGGAATTTCTATCTGC-CCA--TG
GTAGCTAGCAATAGTTA--TC-AATC--GGGTGATCTACTGTGAGAAAA-TTGTAGTGTT
CAAAGCA--GG-C---GTC---TTACGTTTGTTCAATGCAGCATGGTATAGTAAAATATG
ACAC---TAAATATAT-GTTGGTTG-TATA---TTCT-TAGTGTAATGACTAATAGGGAA
GGGCGGGGCCGTTCATATTGATGGGCGAGAGGTGAAATTCGTTGACCCTATCAAGATGCA
CTACAGCGAAAGCATTCGGCAAGTACTTCTCCATTAATCAAGAACGAAAGTTTGGGGATC
GAAGACGATCAGATACCGTCGTAGTCCAAACCATAAACTATGTCGACCAGGGATTGGACG
GATAAT-TTTTTAAAAACTCGCTCAGAACCTT-GTGAGAAATCAT-GAGTGTTTGGACTC
TGGGGGGAGTATGGTCGCAAGTCTGAAACTTAAAGGAATTGACGGAAGGGCACACAATGG
AGTGGAA-CCTGCGGCTTAATTT-GACTCAACTCGGGAAA-ACTTACCAAGCTCAGATAT
AATAAGGATTGACAGA-CTAA-AAGATCTTTCATGATTGTATAAGTGGTGGTGCATGGTC
GTTCTTAG-TTGGTGGAGTGATTTGTCTGGTCAATTCCGATAACGGACGAGACCTCTACT
TACTAACTAGTGGTATTTATTTGGTCAATATGGGAGATAGTCATT-TGGTGTTGGTAGTT
AGGC--CGTTAAAA-GTTTA---GCTATTCTTCATTGAG-TGGTGTGTATTCTGATCAGA
TAG--GTACTAATTTAAAAAATA---AA-CTTCTTAGAAGGACTACCT-ACCTCAAGTGG
GGGG-AAGTCGGAGGCAATAACAGGTCTGTGATGCCCTTAGATACCTT-GGGCTGCACGC
GCGTTACAATGTAGGCGAGAAAAAGGT----TTCC--------GGAATCGA-AAG---GT
TTC-GGTAATCATTTGAATTGCCTACGTAATGGGGATTA-ATTTTTGTAATTATCGATTA
TCAACGAGGAATTCCTTGTAAGCGTAAATCATTACTT----------------------

>AM168104.1_Heterostelium_pallidum_PN500
TCTAAG-TATAA-CCTTTA-TACGGTGAAA----CTGCAGACGGCTCATT--ACAA--CA
---GT----GATAAACTA-AAGAACTTTCGC-GCTTC-----GGCGTCTT-GGATAACCG
CAGTAAATCGGGGCTAATACATGTAAA-CG-AGAGGATGAGCAGG----TAACTGCG-AG
TCTTTGCGATTGTT----A-GCT-TTCATT---CACACCAACCTCTTCGGAGTTTGTGGT
GAGTCCGAACAATATTGCTGATCGGAA--------ACT-TGTTTCCGACGAGTTCTTTGT
GTCACTGCCCTATCAACTTTCGATGGTAAGGTATTGGCTTACCATGGTTGTAACGGGTGA
CGGGGAATCAGGGTTCGATTCCGGAGAGGGAGCCTGAGAAATGGCTACCACTTCTACGGA
AGGCAGCAGGCGCGCAAATT-ACTCAATCCCAATAC-GGGGAAGTAGTGACAAAAAATAC
TAATGCCTTTCCATTTATATGGGGG--GCAATTGGAATAAGTACAACTTAAATCGCTTAG
CA-AAAGTGATTGGAGGGCAAGTCTGGTGCCAGCAGCCGCGGTAATTCCAGCTCCAA-TA
GCGTATACTAATTTTGTTGCAGTTAAAA-AGC-TCGTAGTTGAGATTGAGATTTCTTA-G
GT-TTAG---AGTC-GCTTGGCCTT--CGGGTTTGAG-TT-TGAT-TCGTAAAGCTCTAT
AGTAGAATTCC-ATTTT-A-TTATTAGGGTTA-CTAAGAAATTTCTATCTGC-CCA--TG
GTAGCTAGCAATAGTTA--TC-AATC--GGGTGATCTACTGTGAGAAAA-TTGTAGTGTT
CAAAGCA--GG-C---GTC---TTACGTTTGTTCAATGCAGCATGGTATAGTAAAATATG
ACAC---TAAATATAT-GTTGGTTG-TATA---TTCT-TAGTGTAATGACTAATAGGGAA
GGGCGGGGCCGTTCATATTGATGGGCGAGAGGTGAAATTCGTAGACCCTATCAAGATGCA
CTACAGCGAAAGCATTCGGCAAGTACTTCTCCATTAATCAAGAACGAAAGTTTGGGGATC
AAAGACGATCAGATACCGTCGTAGTCCAAACCATAAACTATGTCGACCAGGGATTGGACG
GATAAT-TTTTTAAAAACTCGCTCAGAACCTT-GTGAGAAATCAT-GAGTGTTTGGACTC
TGGGGGGAGTATGGTCGCAAGTCTGAAACTTAAAGGAATTGACGGAAGGGCACACAATGG
AGTGGAA-CCTGCGGCTTAATTT-GACTCAACTCGGGAAA-ACTTACCAAGCTCAGATAT
AATAAGGATTGACAGA-CTAA-AGGATCTTTCATGATTGTATAAGTGGTGGTGCATGGTC
GTTCTTAG-TTGGTGGAGTGATTTGTCTGGTCAATTCCGATAACGGACGAGACCTCTACT

TACTAACTAGTGGTATTTATTTGGTCACTATGGGAGATAGTCATT-TGGTGTTGGTAGTT
AGGC--CGTTAAAA-GTTTA---GCTATTCTTCATTGAG-TGGTGTGTATTCTGATCAGA
TAG--GTACTAATTTAAAAAATA---AA-CTTCTTAGAAGGACTACCT-ACCTCAAGTGG
GGGG-AAGTCGGAGGCAATAACAGGTCTGTGATGCCCTTAGATACCTT-GGGCTGCACGC
GCGTTACAATGTAGGCGAGAAAAAGGT----TTCC--------GGAATCGA-AAG---GT
TTC-GGTAATCATTTGAATTGCCTACGTAATGGGGATTA-ATTTTTGTAATTATCGATTA
TCAACGAGGAATTCCTTGTAAGCGTAAATCATTACTTTACGCTGAATATGTCCCTGCCCT
TTGTACACACCGCCCGTCGCTCCTACCGATCGAACGATTAGGTAAAACTGACGGACTG--
--GAT-GACTTT--TT---CGC-A-AGG-ATTTGTC-------GTT-TGG-GAAG--TTA
-GT-TAAATCT--C----ATT--GTTT
>EU004605.1_Heterostelium_pallidum_PPHU8_ATCC_44421
TCTAAG-TATAA-CCTTTA-TACGGTGAAA----CTGCAGACGGCTCATT--ACAA--CA
---GT----GATAAACTA-AAGAACTTTCGC-GCTTC-----GGCGTCTT-GGATAACCG
CAGTAAATCGGGGCTAATACATGTAAA-CG-AGAGGATGAGCAGG----TAACTGCG-AG
TCTTTGCGATTGTT----A-GCT-TTCATT---CACACCAACCTCTTCGGAGTTTGTGGT
GAGTCCGAACAATATTGCTGATCGGAA--------ACT-TGTTTCCGACGAGTTCTTTGT
GTCACTGCCCTATCAACTTTCGATGGTAAGGTATTGGCTTACCATGGTTGTAACGGGTGA
CGGGGAATCAGGGTTCGATTCCGGAGAGGGAGCCTGAGAAATGGCTACCACTTCTACGGA
AGGCAGCAGGCGCGCAAATT-ACTCAATCCCAATAC-GGGGAAGTAGTGACAAAAAATAC
TAATGCCTTTCCATTTATATGGGGG--GCAATTGGAATAAGTACAACTTAAATCGCTTAG
CA-AAAGTGATTGGAGGGCAAGTCTGGTGCCAGCAGCCGCGGTAATTCCAGCTCCAA-TA
GCGTATACTAAATTTGTTGCAGTTAAAA-AGC-TCGTAGTTGAGATTGAGATTTCTTA-G
GT-TTAG---AGTC-GCTTGGCCTT--CGGGTTTGAG-TT-TGAT-TCGTAAAGCTCTAT
AGTAGAATTCC-ATTTT-A-TTATTAGGGTTA-CTAAGAAATTTCTATCTGC-CCA--TG
GTAGCTAGCAATAGTTA--TC-AATC--GGGTGATCTACTGTGAGAAAA-TTGTAGTGTT
CAAAGCA--GG-C---GTC---TTACGTTTGTTCAATGCAGCATGGTATAGTAAAATATG
ACAC---TAAATATAT-GTTGGTTG-TATA---TTCT-TAGTGTAATGACTAATAGGGAA
GGGCGGGGCCGTTCATATTGATGGGCGAGAGGTGAAATTCGTTGACCCTATCAAGATGCA
CTACAGCGAAAGCATTCGGCAAGTACTTCTCCATTAATCAAGAACGAAAGTTTGGGGATC
GAAGACGATCAGATACCGTCGTAGTCCAAACCATAAACTATGTCGACCAGGGATTGGACG
GATAAT-TTTTTAAAAACTCGCTCAGAACCTT-GTGAGAAATCAT-GAGTGTTTGGACTC
TGGGGGGAGTATGGTCGCAAGTCTGAAACTTAAAGGAATTGACGGAAGGGCACACAATGG
AGTGGAA-CCTGCGGCTTAATTT-GACTCAACTCGGGAAA-ACTTACCAAGCTCAGATAT
AATAAGGATTGACAGA-CTAA-AAGATCTTTCATGATTGTATAAGTGGTGGTGCATGGTC
GTTCTTAG-TTGGTGGAGTGATTTGTCTGGTCAATTCCGATAACGGACGAGACCTCTACT
TACTAACTAGTGGTATTTATTTGGTCACTATGGGAGATAGTCATT-TGGTGTTGGTAGTT
AGGC--CGTTAAAA-GTTTA---GCTATTCTTCATTGAG-TGGTGTGTATTCTGATCAGA
TAG--GTACTAATTTAAAAAATA---AA-CTTCTTAGAAGGACTACCT-ACCTCAAGTGG
GGGG-AAGTCGGAGGCAATAACAGGTCTGTGATGCCCTTAGATACCTT-GGGCTGCACGC
GCGTTACAATGTAGGCGAGAAAAAGGT----TTCC--------GGAATCGA-AAG---GT
TTC-GGTAATCATTTGAATTGCCTACGTAATGGGGATTA-ATTTTTGTAATTATCGATTA
TCAACGAGGAATTCCTTGTAAGCGTAAATCATTACTTTACGCTGAATATGTCCCTGCCCT
TTGTACACACCGCCCGTCGCTCCTACCGATCGAACGATTAGGTAAAACTGACGGACTG--
--GAT-GACTTT--TT---CGC-A-AGG-ATTTGTC-------GTT-TGG-GAAG--TTA
-GT-TAAATCT--C----ATT--GTTT
>AM168105.1_Heterostelium_tenuissimum_TNS_C_97
TCTAAG-TATAA-CCTTTA-TACGGTGAAA----CTGCAGACGGCTCATT--ACAA--CA
---GT----GATAAACTA-AAGAACTTTCGC-GCTTC-----GGCGTCTT-GGATAACCG
CAGTAAATCGGGGCTAATACATGTAAA-CG-AGAGGATGAGCAGG----CAACTGCG-AG
TCTTTGCGATTGTT----A-GCT-TTTCTT---CA--CCAACCTCTTCGGAGTTTGTGGT
GAGTCCGAACAATATTGCTGATCGGAA--------ACT-TGTTTCCGACGAGTTCTTTGT
GTCACTGCCCTATCAACTTTCGATGGTAAGGTATTGGCTTACCATGGTTGTAACGGGTGA
CGGGGAATCAGGGTTCGATTCCGGAGAGGGAGCCTGAGAAATGGCTACCACTTCTACGGA
AGGCAGCAGGCGCGCAAATT-ACTCAATCCCAATAC-GGGGAAGTAGTGACAAAAAATAC
TAATGCCTTTCCATTTATATGGGGG--GCAATTGGAATAAGTATAACTTAAATCGCTTAG
CA-AAAGTGATTGGAGGGCAAGTCTGGTGCCAGCAGCCGCGGTAATTCCAGCTCCAA-TA
GCGTATACTAAATTTGTTGCAGTTAAAA-AGC-TCGTAGTTGAGATTGAGATTT-TTA-G
G--TTAG---GGTCGGCATTGCCTT--CGGGTTAGTGCTT-TGAT-TCGTAAAGCTTTGT
AGTAGAATTTTTATTTT-A-TTATTAAAGTTA-CTAAGGAATTTCCAACTGC-CCAA-TT
GTAATTAGCAATAATTA---C-ATTTA-GGGTGATCTACTGTGAGAAAA-TTGTAGTGTT
CAAAGCA--GG-C---GTC---TTACGTTTGTTCAATGCAGCATGGTATAGTAAAATATG
ACAC---TAAATATAT-GGTGGTTG-TATA---TTCT-TAGTGTAATGACTAATAGGGAA
GGGCGGGGCCGTTCATATTGATGGGCGAGAGGTGAAATTCGTTGACCCTATCAAGATGCA
CTACAGCGAAAGCATTCGGCAAGTACTTCTCCATTAATCAAGAACGAAAGTTTGGGGATC
GAAGACGATCAGATACCCTCGTAGTCCAACCCATAAACTATGTCGACCAGGGATTGGACG
GATAAT-TTTTTAAAAACTCGCTCAGAACCTT-GTGAGAAATCAT-GAGTGTTTGGACTC
TGGGGGGAGTATGGTCGCAAGTCTGAAACTTAAAGGAATTGACGGAAGGGCACACAATGG
AGTGGAA-CCTGCGGCTTAATTT-GACTCAACTCGGGAAA-ACTTACCAAGCTCAGATAT
AATAAGGATTGACAGA-CTAA-AAGATCTTTCATGATTGTATAAGTGGTGGTGCATGGTC
GTTCTTAG-TTGGTGGAGTGATTTGTCTGGTCAATTCCGATAACGGACGAGACCTCTACT
TACTAACTAGTGGTATTTATTTGGTCAATATGGGAGATAGTCATT-TGGTGTTGGTAGTT
AGAC--CGTTAAAA-GTTTA---GCTATTCTTCATTGAG-TGGTGTGTATTCTGATCAGA
TAG--GTACTAACTTAAAAAATA---AA-CTTCTTAGAAGGACTACCT-ACCTCAAGTGG

GGGG-AAGTCGGAGGCAATAACAGGTCTGTGATGCCCTTAGATACCTT-GGGCTGCACGC
GCGTTACAATGTAGGCGAGAAAAAGGT----TTCC--------GGAATCGA-AAG---GT
TTC-GGTAATCATTTGAATTGCCTACGTAATGGGGATTA-ATTTTTGTAATTATCGATTA
TCAACGAGGAATTCCTTGTAAGCGTAAATCATTACTTTACGCTGAATATGTCCCTGCCCT
TTGTACACACCGCCCGTCGCTCCTACCGATCGAACGATTAGGTAAAACTGACGGACTA--
--GGC-GACTTT--TC---CGC-A-AGG-ATTTGTT-------GTT-TGG-GAAG--TTA
-GT-TAAATCT--C----ATT--GTTT
>AY040339.1_Heterostelium_tenuissimum
TCTAAG-TATAA-CCTTTA-TACGGTGAAA----CTGCAGACGGCTCATT--ACAA--CA
---GT----GATAAACTA-AAGAACTTTCGC-GCTTC-----GGCGTCTT-GGATAACCG
CAGTAAATCGGGGCTAATACATGTAAA-CG-AAAGGATGAGCAGG----CAACTGCG-AG
TCTTTGCGATTGTT----A-GCT-TTTCTT---CA--CCAACCTCTTCGGAGTTTGTGGT
GAATCCGAACAATATTGCTGATCGGAA--------ACT-TGTTTCCGACGAGTTCTTTGT
GTCACTGCCCTATCAACTTTCGATGGTAAGGTATTGGCTTACCATGGTTGTAACGGGTGA
CGGGGAATCAGGGTTCGATTCCGGAGAGGGAGCCTGAGAAATGGCTACCACTTCTACGGA
AGGCAGCAGGCGCGCAAATT-ACTCAATCCCAATAC-GGGGAAGTAGTGACAAAAAATAC
TAATGCCTTTCCATTTATATGGGGG--GCAATTGGAATAAGTACAACTTAAATCGCTTAG
CA-AAAGTGATTGGAGGGCAAGTCTGGTGCCAGCAGCCGCGG-AATTCCAGCTCCAA-TA
GCGTATACTAAATTTGTTGCAGTTAAAA-AGC-TCGTAGTTGAGACAGAGATTTCTTA-G
GT-TTAG---GGTCGGCATTGCCTT--CGGGTTAGTGCTT-TGAT-TCGTAAAGCTTTGT
AGTAGAATTTC-ATTTT-A-TTATTAAAGTTA-CTAAGGAATTTCCAACTGC-CCA--TG
GTAGCTAGCAATAGTTA--TC-AATC--GGGTGATCTACTGTGAGAAAA-TTGTAGTGTT
CAAAGCA--GG-CC--GTC---TTACGTTTGTTCAATGCAGCATGGTATAGTAAAATATG
ACAC---TAAATATAT-GTTGGTTG-TATA---TTCT-TAGTGTAATGACTAATAGGGAA
GGGCGGGGCCGTTCATATTGATGGGCGAGAGGTGAAATTCGTTGACCCTATCAAGATGCA
CTACAGCGAAAGCATTCGGCAAGTACTTCTCCATTAATCAAGAACGAAAGTTTGGGGATC
GAAGACGATCAGATACCGTCGTAGTCCAAACCATAAACTATGTCGACCAGGGATTGGACG
GATAAT-TTTTTAAAAACTCGCTCAGAACCTT-GTGAGAAATCAT-GAGTGTTTGGACTC
TGGGGGGAGTATGGTCGCAAGTCTGAAACTTAAAGGAATTGACGGAAGGGCACACAATGG
AGTGGAA-CCTGCGGCTTAATTT-GACTCAACTCGGGAAA-ACTTACCAAGCTCAGATAT
AATAAGGATTGACAGA-CTAA-AAGATCTTTCATGATTGTATAAGTGGTGGTGCATGGTC
GTTCTTAG-TTGGTGGAGTGATTTGTCTGGTCAATTCCGATAACGGACGAGACCTCTACT
TACTAACTAGTGGTATTTATTTGGTCAATATGGGAGATAGTCATT-TGGTGTTGGTAGTT
AGAC--CGTTAAAA-GTTTA---GCTATTCTTCATTGAG-TGGTGTGTATTCTGATCAGA
TAG--GTACTAACTTAAAAAATA---AA-CTTCTTAGAAGGACTACCT-AC-TCAAGTGG
GGGG-AAGTCGGAGGCAATAACAGGTCTGTGATGCCCTTAGATACCTT-GGGCTGCACGC
GCGTTACAATGTAGGCGAGAAAAAGGT----TTCC--------GGAATCGA-AAG---GT
TTC-GGTAATCATTTGAATTGCCTACGTAATGGGGATTA-ATTTTTGTAATTATCGATTA
TCAACGAGGAATTCCTTGTAAGCGTAAATCATTACTTTACGCTGAATATGTCCCTGCCCT
TTGTACACACCGCCCGTCGCTCCTACCGATCGAACGATTAGGTAAAACTGACGGATTA--
--GAT-GATTTC--CT---CGC-A-AGG-GGTTATT-------GTT-TGA-GAAG--TTA
-GT-TAAATCT--C----ATT--GTTT
>MN338954.1_Heterostelium_versatile_Mad52
TCTAAG-TATAA-CCTTTA-TACGGTGAAA----CTGCAGACGGCTCATT--ACAA--CA
---GT----GATAAACTA-TAGAACTTTCGC-GCTTC-----GGCGTCTT-GGATAACCG
CAGTAAATCGGGGCTAATACATGTAAA-CG-AAAGGATGAGCGGG----CAACTGCG-AA
TCTTTGCGATTGTT----A-GCT-TTCA-----CACACCAACCTCTTCGGAGTTTGTGGT
GAGTCCGAACAATATTGCTGATCGGAA--------ACT-TGTTTCCGACAAGTTCTTTGT
GTCACTGCCCTATCAACTTTCGATGGTAAGGTATTGGCTTACCATGGTTGTAACGGGTGA
CGGGGAATCAGGGTTCGATTCCGGAGAGGGAGCCTGAGAAATGGCTACCACTTCTACGGA
AGGCAGCAGGCGCGCAAATT-ACTCAATCCCAATAC-GGGGAAGTAGTGACAAAAAATAC
TAATGCCTTTCCATTTATATGGGGG--GCAATTGGAATAAGTACAACTTAAATCGCTTAG
CA-AAAGTGATTGGAGGGCAAGTCTGGTGCCAGCAGCCGCGGTAATTCCAGCTCCAA-TA
GCGTATACTAAATTTGTTGCAGTTAAAA-AGC-TCGTAGTTTAGACTGAGATTTCTTA-G
GT-TTAG--CGGTCTGCATTGCTTTAATCGGTTAATGTTT--GAT-TCGTAAAGCTTTAT
AGTAGAATTTC-ATTTT-A-TTATTAAAGTTA-CTAAGGAATTTCCAACTGC-CCA--TG
GTAGCCAGCAATGGTTA--TC-AATC--GGGCGATCCACTGTGAGAAAA-TTGTAGTGTT
CAAAGCA--GA-C---GTC---TT-CGTTTGTTCAATGCAGCATGGTATGGTAAAATATG
ACAC---TAAATGTAT-GTTGGTTG-TATA---TA-T-TAGTGTAATGACTAATAGGGAA
GGGCGGGGCCGTTTATATTGATGGGCGAGAGGTGAAATTCGTTGACCCTATCAAGATACA
CAACAGCGAAAGCATTCGGCAAGTACTTCTCCATTAATCAAGAACGAAAGTTTGGGGATC
GAAGACGATCAGATACCGTCGTAGTCCAAACCATAAACTATGTCGACCAGGGATTGGACG
GATAAT-TTTTTAAAAACTCGCTCAGAACCTT-GTGAGAAATCAT-GAGTGTTTGGACTC
TGGGGGGAGTATGGTCGCAAGTCTGAAACTTAAAGGAATTGACGGAAGGGCACACAATGG
AGTGGAAACCTGCGGCTTTAATT-------------------------------------

>HQ141500.1_Heterostelium_flexuosum_AU4B
TCTAAG-TATAA-CCTTTA-TACGGTGAAC----CTGCAGAAGGATCAACT-ACAA--CT
-ATGT----GATAAACTA-TAGAACTTTCGC-GCTTC-----GGCGTCTT-GGATAACCG
CAGTAAATCGGGGCTAATACATGTAAA-CG-AAAGGATGAGCGGG----CAACTGCG-AG
TCTTTGCGATTGTT----A-GCT-TTCA-----CACACCAACCTCTTCGGAGTTTGTGGT
GAGTCCGAACAATATTGCTGATCGGAA--------ACT-TGTTTCCGACAAGTTCTTTGT
GTCACTGCCCTATCAACTTTCGATGGTAAGGTATTGGCTTACCATGGTTGTAACGGGTGA
CGGGGAATCAGGGTTCGATTCCGGAGAGGGAGCCTGAGAAATGGCTACCACTTCTACGGA
AGGCAGCAGGCGCGCAAATT-ACTCAATCCCAATAC-GGGGAAGTAGTGACAAAAAATAC
TAATGCCTTTCCATTTATATGGGGG--GCAATTGGAATAAGTACAACTTAAATCGCTTAG
CA-AAAGTGATTGGAGGGCAAGTCTGGTGCCAGCAGCCGCGGTAATTCCAGCTCCAA-TA
GCGTATACTAAATTTGTTGCAGTTAAAA-AGC-TCGTAGTTTAGACTGAGATTTCTTA-G
GT-TTAG--CGGTCGGCATTGCCTTAACCGGTTAATGTTT-TGAT-TCGTAAAGCTTTAT
AGTAGAATTTC-ATTTT-A-TTATTAAAGTTA-CTAAGGAGTTTCCAACTGC-CCA--TG
GTAGCTAGCAATAGTTA--TC-AATC--GGGTGATCCACTGTGAGAAAA-TTGTAGTGTT
CAAAGCA--GA-C---GTC---TT-CGTTTGTTCAATGCAGCATGGTATGGTAAAATATG
ACAC---TAAATGTAT-GTTGGTTG-TATA---TA-T-TAGTGTAATGACTAATAGGGAA
GGGCGGGGCCGTTTATATTGATGGGCGAGAGGTGAAATTCGTTGACCCTATCAAGATACA
CAACAGCGAAAGCATTCGGCAAGTACTTCTCCATTAATCAAGAACGAAAGTTTGGGGATC
GAAGACGATCAGATACCGTCGTAGTCCAAACCATAAACTATGTCGACCAGGGATTGGACG
GATAAT-TTTTTAAAAACTCGCTCAGAACCTT-GTGAGAAATCAT-GAGTGTTTGGACTC
TGGGGGGAGTATGGTCGCAAGTCTGAAACTTAAAGGAATTGACGGAAGGGCACACAATGG
AGTGGAA-CCTGCGGCTTAATTT-GACTCAACTCGGGAAA-ACTTACCAAGCTCAGATAT
AATAAGGATTGACAGA-CTAA-AAGATCTTTCATGATTGTATAAGTGGTGGTGCATGGTC
GTTCTTAG-TTGGTGGAGTGATTTGTCTGGTCAATTCCGATAACGGACGAGACCTCTACT
TACTAACTAGTGGTATTTATTTGGTCAATATGGGAGATAGTCATT-TGGTGTTGGTAGT-
AGGC--CGTCAAAA-GTTT----GCTATTCTTCATTGAG-TGGTGTGTATTCTGATCAGA
TAG--GTACTAACAAAAAAAAATA---AA-CTTCTTAGAAGGACTACCT-ACCTCAAGTGG
GGGG-AAGTCGGAGGCAATAACAGGTCTGTGATGCCCTTAGATACCTT-GGGCTGCACGC
GCGTTACAATGTAGGCGAGAAAAAGGT----TTCC--------GGAATCGA-AAG---GT
TTC-GGTAATCATTTGAATTGCCTACGTAATGGGGATTA-ATTTTTGTAATTATCGATTA
TCAACGAGGAATTCCTTGTAAGCGTAAATCATTACTTTACGCTGAATATGTCCCTGCCCT
TTGTACACACCGCCCGTCGCTCCTACCGATCGAACGATTAGGTAAAACTGACGGACTA--
--GGC-GATTTT--TT---AAC-CATGG---TTGAT-------CCT-GGT-GAACC--TG
-CA-GAAGGAT--C----A----GCC-
>HQ141501.1_Heterostelium_rotatum_QC2C
TCTAAG-TATAA-CCTTTA-TACGGTGAAC----CTGCAGAAGGATCAACT-ACAA--CA
--TGT----GATAAACTA-TAGAACTTTCGC-GCTTC-----GGCGTCTT-GGATAACCG
CAGTAAATCGGGGCTAATACATGTAAA-CG-AAAGGATGAGCGGG----CAACTGCG-AG
TCTTTGCGATTGTT----A-GCT-TTCA-----CACACCAACCTCTTCGGAGTTTGTGGT
GAGTCCGAACAATATTGCTGATCGGAA--------ACT-TGTTTCCGACAAGTTCTTTGT
GTCACTGCCCTATCAACTTTCGATGGTAAGGTATTGGCTTACCATGGTTGTAACGGGTGA
CGGGGAATCAGGGTTCGATTCCGGAGAGGGAGCCTGAGAAATGGCTACCACTTCTACGGA
AGGCAGCAGGCGCGCAAATT-ACTCAATCCCAATAC-GGGGAAGTAGTGACAAAAAATAC
TAATGCCTTTCCATTTATATGGGGG--GCAATTGGAATAAGTACAACTTAAATCGCTTAG
CA-AAAGTGATTGGAGGGCAAGTCTGGTGCCAGCAGCCGCGGTAATTCCAGCTCCAA-TA
GCGTATACTAAATTTGTTGCAGTTAAAA-AGC-TCGTAGTTTAGACTGAGATTTCTTA-G
GT-TTAG--CGGTCGGCATTGCCTTAACCGGTTAATGTTT-TGAT-TCGTAAAGCTTTAT
AGTAGAATTTC-ATTTT-A-TTATTAAAGTTA-CTAAGGAGTTTCCAACTGC-CCA--TG
GTAGCTAGCAATAGTTA--TC-AATC--GGGTGATCCACTGTGAGAAAA-TTGTAGTGTT
CAAAGCA--GA-C---GTC---TT-CGTTTGTTCAATGCAGCATGGTATGGTAAAATATG
ACAC---TAAATGTAT-GTTGGTTG-TATA---TA-T-TAGTGTAATGACTAATAGGGAA
GGGCGGGGCCGTTTATATTGATGGGCGAGAGGTGAAATTCGTTGACCCTATCAAGATACA
CAACAGCGAAAGCATTCGGCAAGTACTTCTCCATTAATCAAGAACGAAAGTTTGGGGATC
GAAGACGATCAGATACCGTCGTAGTCCAAACCATAAACTATGTCGACCAGGGATTGGACG
GATAAT-TTTTTAAAAACTCGCTCAGAACCTT-GTGAGAAATCAT-GAGTGTTTGGACTC
TGGGGGGAGTATGGTCGCAAGTCTGAAACTTAAAGGAATTGACGGAAGGGCACACAATGG
AGTGGAA-CCTGCGGCTTAATTT-GACTCAACTCGGGAAA-ACTTACCAAGCTCAGATAT
AATAAGGATTGACAGA-CTAA-AAGATCTTTCATGATTGTATAAGTGGTGGTGCATGGTC
GTTCTTAG-TTGGTGGAGTGATTTGTCTGGTCAATTCCGATAACGGACGAGACCTCTACT
TACTAACTAGTGGTATTTATTTGGTCAATATGGGAGATAGTCATT-TGGTGTTGGTAGT-
AGGC--CGTCAAAA-GTTT----ACTATTCTTCATTGAG-TGGTGTGTATTCTGATCAGA
TAG--GTACTAACAAAAAAAAATA---AA-CTTCTTAGAAGGACTACCT-ACCTCAAGTGG
GGGG-AAGTCGGAGGCAATAACAGGTCTGTGATGCCCTTAGATACCTT-GGGCTGCACGC
GCGTTACAATGTAGGCGAGAAAAAGGT----TTCC--------GGAATCGA-AAG---GT
TTC-GGTAATCATTTGAATTGCCTACGTAATGGGGATTA-ATTTTTGTAATTATCGATTA
TCAACGAGGAATTCCTTGTAAGCGTAAATCATTACTTTACGCTGAATATGTCCCTGCCCT
TTGTACACACCGCCCGTCGCTCCTACCGATCGAACGATTAGGTAAAACTGACGGACTA--
--GGC-GATTTT--TT--AAGC-CATGG---TTGAT-------CCT-GGT-GAACC--TG

-CA-GAAGGAT--C----A----GCC-
>AM168074.1_Heterostelium_gloeosporum_TCK52
TCTAAG-TATAA-CCTTTA-TACGGTGAAA----CTGCAGACGGCTCATT--ACAA--CA
---GT----GATAAACTA-TAGAACTTTCGC-GCTTC-----GGCGTCTT-GGATAACCG
CAGTAAATCGGGGCTAATACATGTAAA-CG-AAAGGATGAGCGGG----CAACTGCG-AG
TCTTTGCGATTGTT----A-GCT-TTCA-----CACACCAACCTCTTCGGAGTTTGTGGT
GAGTCCGAACAATATTGCTGATCGGAA--------ACT-TGTTTCCGACAAGTTCTTTGT
GTCACTGCCCTATCAACTTTCGATGGTAAGGTATTGGCTTACCATGGTTGTAACGGGTGA
CGGGGAATCAGGGTTCGATTCCGGAGAGGGAGCCTGAGAAATGGCTACCACTTCTACGGA
AGGCAGCAGGCGCGCAAATT-GCTCAATCCCAATAC-GGGGAAGTAGTGACAAAAAATAC
TAATGCCTTTCCATTTATATGGGGG--GCAATTGGAATAAGTACAACTTAAATCGCTTAG
CA-AAAGTGATTGGAGGGCAAGTCTGGTGCCAGCAGCCGCGGTAATTCCAGCTCCAA-TA
GCGTATACTAAATTTGTTGCAGTTAAAA-AGC-TCGTAGTTTAGATTGAGATTTCTTA-G
GT-TTAG--CGGTCGGTATTGCCTTAACCGGTTAATACTT-TGAT-TCGTAAAGCTTTAT
AGTAGAATTTC-ATTTT-A-TTATTAAAGTTA-CTAAGGAGTTTCCAACTGC-CCA--TG
GTAGCTAGCAATAGTTA--TC-AATC--GGGTGATCCACTGTGAGAAAA-TTGTAGTGTT
CAAAGCA--GA-C---GTC---TT-CGTTTGTTCAATGCAGCATGGTATGGTAAAATATG
ACAC---TAAATGTAT-GTTGGTTG-TATA---TA-T-TAGTGTAATGACTAATAGGGAA
GGGCGGGGCCGTTTATATTGATGGGCGAGAGGTGAAATTCGTTGACCCTATCAAGATACA
CAACAGCGAAAGCATTCGGCAAGTACTTCTCCATTAATCAAGAACGAAAGTTTGGGGATC
GAAGACGATCAGATACCGTCGTAGTCCAAACCATAAACTATGTCGACCAGGGATTGGACG
GATAAT-TTTTTAAAAACTCGCTCAGAACCTT-GTGAGAAATCAT-GAGTGTTTGGACTC
TGGGGGGAGTATGGTCGCAAGTCTGAAACTTAAAGGAATTGACGGAAGGGCACACAATGG
AGTGGAA-CCTGCGGCTTAATTT-GACTCAACTCGGGAAA-ACTTACCAAGCTCAGATAT
AATAAGGATTGACAGA-CTAA-AAGATCTTTCATGATTGTATAAGTGGTGGTGCATGGTC
GTTCTTAG-TTGGTGGAGTGATTTGTCTGGTCAATTCCGATAACGGACGAGACCTCTACT
TACTAACTAGTGGTATTTATTTGGTCAATATGGGAGATAGTCATT-TGGTGTTGGTAGT-
AGGC--CGTTAAAA-GTTT----GCTATTCTTCATTGAG-TGGTGTGTATTCTGATCAGA
TAG--GTACTAACAAAAAAAATA---AA-CTTCTTAGAAGGACTACCT-ACCTCAAGTGG
GGGG-AAGTCGGAGGCAATAACAGGTCTGTGATGCCCTTAGATACCTT-GGGCTGCACGC
GCGTTACAATGTAGGCGAGAAAAAGGT----TTCC--------GGAATCGA-AAG---GT
TTC-GGTAATCATTTGAATTGCCTACGTAATGGGGATTA-ATTTTTGTAATTATCGATTA
TCAACGAGGAATTCCTTGTAAGCGTAAATCATTACTTTACGCTGAATATGTCCCTGCCCT
TTGTACACACCGCCCGTCGCTCCTACCGATCGAACGATTAGGTAAAACTGACGGACTA--
--AAC-GATTTT--TT---GGC--AACA-AATTTTT-------GTT-TGG-GAAG--TTA
-GT-TAAATCT--C----ATT--GTTT
>HQ141502.1_Heterostelium_granulosum_MF5A
TCTAAG-TATAA-CCTTTA-TACGGTGAAC----CTGCAGAAGGATCAACT-ACAA--CA
--TGT----GATAAACTA-TAGAACTTTCGC-GCTTC-----GGCGTCTT-GGATAACCG
CAGTAAATCGGGGCTAATACATGTAAA-CG-AAAGGATGAGCGGG----TAACTGCG-AG
TCTTTGCGATTGTT----A-GCT-TTCA-----CACACCAACCTCTTCGGAGTTTGTGGT
GAGTCCGAACAATATTGCTGATCGGAA--------ATT-TATTTCCGACAAGTTCTTTGT
GTCACTGCCCTATCAACTTTCGATGGTAAGGTATTGGCTTACCATGGTTGTAACGGGTGA
CGGGGAATCAGGGTTCGATTCCGGAGAGGGAGCCTGAGAAATGGCTACCACTTCTACGGA
AGGCAGCAGGCGCGCAAATT-ACTCAATCCCAATAC-GGGGAAGTAGTGACAAAAAATAC
TAATGCCTTTCCATTTATATGGGGG--GCAATTGGAATAAGTACAACTTAAATCGCTTAG
CA-AAAGTGATTGGAGGGCAAGTCTGGTGCCAGCAGCCGCGGTAATTCCAGCTCCAA-TA
GCGTATACTAAATTTGTTGCAGTTAAAA-AGC-TCGTAGTTTAGACTGAGATTTCTTG-G
GT-CTAG--CGGTCTGCATTGCCTTTACCGGTTAATGTTT--GAT-TCGTAAAGCTTTAT
AGTAGAATTTC-ATTTT-A-TTATTAAAGTTA-CCAAGGAGTTTCCAACTGC-CCA--TG
GTAGCTAGCAATAGTTA--TC-AATC--GGGTGATCCACTGTGAGAAAA-TTGTAGTGTT
CAAAGCA--GA-C---GT----TT-CGTTTGTTCAATGCAGCATGGTATGGTAAAATATG
ACAC---TAAATGTAT-GTTGGTTG-TATA---TA-T-TAGTGTAATGACTAATAGGGAA
GGGCGGGGCCGTTTATATTGATGGGCGAGAGGTGAAATTCGTTGACCCTATCAAGATACA
CAACAGCGAAAGCATTCGGCAAGTACTTCTCCATTAATCAAGAACGAAAGTTTGGGGATC
GAAGACGATCAGATACCGTCGTAGTCCAAACCATAAACTATGTCGACCAGGGATTGGACG
GATAAT-TTTTTAAAAACTCGCTCAGAACCTT-GTGAGAAATCAT-GAGTGTTTGGACTC
TGGGGGGAGTATGGTCGCAAGTCTGAAACTTAAAGGAATTGACGGAAGGGCACACAATGG
AGTGGAA-CCTGCGGCTTAATTT-GACTCAACTCGGGAAA-ACTTACCAAGCTCAGATAT
AATAAGGATTGACAGA-CTAA-AAGATCTTTCATGATTGTATAAGTGGTGGTGCATGGTC
GTTCTTAG-TTGGTGGAGTGATTTGTCTGGTCAATTCCGATAACGGACGAGACCTCTACT
TACTAACTAGTGGTATTTATTTGGTCAATATGGGAGATAGTCATT-TGGTGTTGGTAGT-
AGGC--CGTCAAAA-GCTT----GCTATTCTTCATTGAG-TGGTGTGTATTCTGATCAGA
TAG--GTACTAACAAAAAAAATA---AA-CTTCTTAGAAGGACTACCT-ACCTCAAGTGG
GGGG-AAGTCGGAGGCAATAACAGGTCTGTGATGCCCTTAGATACCTT-GGGCTGCACGC
GCGTTACAATGTAGGCGAGAAAAAGGT----TTCC--------GGAATCGA-AAG---GT
TTC-GGTAATCATTTGAATTGCCTACGTAATGGGGATTA-ATTTTTGTAATTATCGATTA
TCAACGAGGAATTCCTTGTAAGCGTAAATCATTACTTTACGCTGAATATGTCCCTGCCCT
TTGTACACACCGCCCGTCGCTCCTACCGATCGAACGATTAGGTAAAACTGACGGACTA--
--GGC-GATTTT--TT--AAGC-CATGG---TTGAT-------CCT-GGT-GAACC--TG
-CA-GAAGGAT--C----A----GCC-
>KP167480.1_Heterostelium_ampliverticillatum
TCCAAG-TATAA-CCTTTC-TACGGTGAAC----CTGCAGAAGGATCAAGT-ACAA--CA

--TGT---AGATAAACTA-AAGAACTTTCGC-GCTTC-----GGCGTTCT-GGATAACCG
CAGTAAATCGGGGCTAATACATGTAAA-CG-AGAGGATGAGCGGG----CAACTGCG-AG
TCTTTGCGATTGTT----A-GCT-TTTATT---CACACCAACCTCTTCGGAGTTTGTGGT
GAGTCCGAACAATATTGCTGATCGGAA--------ACT-TGTTTCCGACGAGTTCTTTGT
GTCACTGCCCTATCAACTTTCGATGGTAAGGTATTGGCTTACCATGGTTGTAACGGGTGA
CGGGGAATCAGGGTTCGATTCCGGAGAGGGAGCCTGAGAAATGGCTACCACTTCTATGGA
AGGCAGCAGGCGCGCAAATT-ACTCAATCCCAATAC-GGGGAAGTAGTGACAAAAAATAC
TAATGCCTTTCCATTTATATGGGGG--GCAATTGGAATAAGTACAATTTAAATCGCTTAG
CA-AAAGTGATTGGAGGGCAAGTCTGGTGCCAGCAGCCGCGGTAATTCCAGCTCCAA-TA
GCGTATACTAAATTTGTTGCAGTTAAAA-AGC-TCGTAGTTGAGATAGAGATTTCTCG-G
GT-TTAG--CGGTCGTTATTGCCTTCACGGGTTAATAATT-TGAT-TCGTAAAGCTTTAT
AGTAAGATTCC-A-TCTTA-TTATTAAAGTTA-CCGAGGAATTTCCAACTGC-CCA--TG
GTAACTGGTAACAGTTA--TC-AATC--GGGTGATCTACTGTGAGAAAA-TTGTAGTGTT
CAAAGCA--GG-C---GT----TTACGTTTGTTCAATGCAGCATGGTATAGTAAAATATG
ACAC---TAAATATAT-GTTGGTTG-TATA---TTCT-TAGTGTAATGACTAATAGGGAA
GGGCGGGGCCGTTCATATTGATGGGCGAGAGGTGAAATTCGTTGACCCTATCAAGATGCA
CTACAGCGAAAGCATTCGGCAAGTACTTCTCCATTAATCAAGAACGAAAGTTTGGGGATC
GAAGACGATCAGATACCGTCGTAGTCCAAACCATAAACTATGTCGACCAGGGATTGGACG
GATAAT-TTTTTAAAAACTCGCTCAGAACCTT-GTGAGAAATCAT-GAGTGTTTGGACTC
TGGGGGGAGTATGGTCGCAAGTCTGAAACTTAAAGGAATTGACGGAAGGGCACACAATGG
AGTGGAA-CCTGCGGCTTAATTT-GACTCAACTCGGGAAA-ACTTACCAAGCTCAGATAT
AATAAGGATTGACAGA-CTAA-AAGATCTTTCATGATTGTATAAGTGGTGGTGCATGGTC
GTTCTTAG-TTGGTGGAGTGATTTGTCTGGTCAATTCCGATAACGGACGAGACCTCTACC
TACTAACTAGTGGTATTTATTTGGTCATTATGGGAGATAGTCATT-TGGTGTTGGTAGT-
AGGC--CGTCAAAA-GTTTA----CTATTCTTCATTGAG-TGGTGTGTATCCTGATCAGA
TAG--GTACTATCTAAAAAAATA---AA-CTTCTTAGAGGGACTACCT-ACCTCAAGTGG
GGGG-AAGTCGGAGGCAATAACAGGTCTGTGATGCCCTTAGATACCTT-GGGCTGCACGC
GCGTTACAATGTAGACGAGAAAAAGGC----TTCC--------GACATCGA-AAG---GT
GCC-GGTAATCAATTGAATTGTCTACGTAATGGGGATTA-ATTTTTGTAATTATCGATTA
TCAACGAGGAATTCCTTGTAAGCGTAAATCATTACTTTACGCTGAATATGTCCCTGCCCT
TTGTACACACCGCCCGTCGCTCCTACCGATCGAACGATTAGGTAAAACTGACGGACTG--
--AAC-GACT----GT---AAC-C-TGG---TTGAT-------CCT-------------
----------------------GCC-
>KP167479.1_Heterostelium_cumulocystum
---AT---ACAA--CA
---GT----GATAAACTA-AAGAACTTTCGC-GCTTC-----GGCGTTCT-GGATAACCG
CAGTAAATCGGGGCTAATACATGTAAA-CG-AGAGGATGAGCGGG----CAACTGCG-AG
TCTTTGCGATTGTT----A-GCT-TTTATT---CACACCAACCTCTTCGGAGTTTGTGGT
GAGTCCGAACAATATTGCTGATCGGAA--------ACT-TGTTTCCGACGAGTTCTTTGT
GTCACTGCCCTATCAACTTTCGATGGTAAGGTATTGGCTTACCATGGTTGTAACGGGTGA
CGGGGAATCAGGGTTCGATTCCGGAGAGGGAGCCTGAGAAATGGCTACCACTTCTATGGA
AGGCAGCAGGCGCGCAAATT-ACTCAATCCCAATAC-GGGGAAGTAGTGACAAAAAATAC
TAATGCCTTTCCATTTATATGGGGG--GCAATTGGAATAAGTACAATTTAAATCGCTTAG
CA-AAAGTGATTGGAGGGCAAGTCTGGTGCCAGCAGCCGCGGTAATTCCAGCTCCAA-TA
GCGTATACTAAATTTGTTGCAGTTAAAA-AGC-TCGTAGTTGAGATAGAGATTTCTCG-G
GT-TTAG--CGGTCGTTATTGCCTTCACGGGTTAATAATT-TGAT-TCGTAAAGCTTTAT
AGTAAGATTCC-A-TCTTA-TTATTAAAGTTA-CCGAGGAATTTCCAACTGC-CCA--TG
GTAACTGGTAACAGTTA--TC-AATC--GGGTGATCTACTGTGAGAAAA-TTGTAGTGTT
CAAAGCA--GG-C---GT----TTACGTTTGTTCAATGCAGCATGGTATAGTAAAATATG
ACAC---TAAATATAT-GTTGGTTG-TATA---TTCT-TAGTGTAATGACTAATAGGGAA
GGGCGGGGCCGTTCATATTGATGGGCGAGAGGTGAAATTCGTTGACCCTATCAAGATGCA
CTACAGCGAAAGCATTCGGCAAGTACTTCTCCATTAATCAAGAACGAAAGTTTGGGGATC
GAAGACGATCAGATACCGTCGTAGTCCAAACCATAAACTATGTCGACCAGGGATTGGACG
GATAAT-TTTTTAAAAACTCGCTCAGAACCTT-GTGAGAAATCAT-GAGTGTTTGGACTC
TGGGGGGAGTATGGTCGCAAGTCTGAAACTTAAAGGAATTGACGGAAGGGCACACAATGG
AGTGGAA-CCTGCGGCTTAATTT-GACTCAACTCGGGAAA-ACTTACCAAGCTCAGATAT
AATAAGGATTGACAGA-CTAA-AAGATCTTTCATGATTGTATAAGTGGTGGTGCATGGTC
GTTCTTAG-TTGGTGGAGTGATTTGTCTGGTCAATTCCGATAACGGACGAGACCTCTACC
TACTAACTAGTGGTATTTATTTGGTCATTATGGGAGATAGTCATT-TGGTGTTGGTAGT-
AGGC--CGTCAAAA-GTTTA----CTATTCTTCATTGAG-TGGTGTGTATCCTGATCAGA
TAG--GTACTATCTAAAAAAATA---AA-CTTCTTAGAGGGACTACCT-ACCTCAAGTGG
GGGG-AAGTCGGAGGCAATAACAGGTCTGTGATGCCCTTAGATACCTT-GGGCTGCACGC
GCGTTACAATGTAGACGAGAAAAAGGC----TTCC--------GACATCGA-AAG---GT
GCC-GGTAATCAATTGAATTGTCTACGTAATGGGGATTA-ATTTTTGTAATTATCGATTA
TCAACGAGGAATTCCTTGTAAGCGTAAATCATTACTTTACGCTGAATATGTCCCTGCCCT
TTGTACACACCGCCCGTCGCTCCTACCGATCGAACGATTAGGTAAAACTGACGGACTG--
--AAC-GACT---

>HQ141503.1_Heterostelium_asymetricum_HN20C
TCTAAG-TATAA-CCTTTA-TACGGTGAAC----CTGCAGAAGGATCAACT-ACGG--CA
--TGT---AGATAAACTA-AAGAACTTTCGC-GCTTC-----CGGGTCTT-GGATAACCG
CAGTAAATCGGGGCTAATACATGTAAA-CG-AGAGGATGAGCAGG----CAACTGCG-AG
TCTTTGCGATTGTT----A-GCTATTCATT---CACACCAACCTCTTCGGAGTTTGTGGT

GAGTCCGAACAATATTGCTGATCGGAA--------ACT-TGTTTCCGACGAGTTCTTTGT
GTCACTGCCCTATCAACTTTCGATGGTAAGGTATTGGCTTACCATGGTTGTAACGGGTGA
CGGGGAATCAGGGTTCGATTCCGGAGAGGGAGCCTGAGAAATGGCTACCACTTCTACGGA
AGGCAGCAGGCGCGCAAATT-ACTCAATCCCAATAC-GGGGAAGTAGTGACAAAAAATAC
TAATGCCTTTCCATTTATATGGGGG--GCAATTGGAATAAGTACAACTTAAATCGCTTAA
CG-AAAGTGATTGGAGGGCAAGTCTGGTGCCAGCAGCCGCGGTAATTCCAGCTCCAA-TA
GCGTATACTAAATTTGTTGCAGTTAAAA-AGC-TCGTAGTTGAGATTGAGATTTCTTG-G
GT-TTAG--CGTCCAAGATTGCCTTCACGGGTTAATCTTT-TGGT-ACGTAAAGCTTTTC
AGTGGAATTCT-ATTT-CA-TTGTTAAAGTTA-CCAAGGAATTTCTATCTGC-CCA--TT
TAAGGTGGCAACACTT---TA-AATTTAGGGTGATCTACTGTGAGAAAA-TTGTAGTGTT
CAAAGCA--GG-C---GTT---TT-CGCTTGTTCAATGCAGCATGGTATAGTAAAATATG
ACAC---TAAATATAT-GTTGGTTG-TATA---TT-T-TAGTGTAATGACTAATAGGGAA
GGGCGGGGCCGTTCATATTGATGGGCGAGAGGTGAAATTCGTTGACCCTATCAAGATGCA
CTACAGCGAAAGCATTCGGCAAGTACTTCTCCATTAATCAAGAACGAAAGTTTGGGGATC
GAAGACGATCAGATACCGTCGTAGTCCAAACCATAAACTATGTCGACCAGGGATTGGACG
GATAAT-TTTTTAAAAACTCGCTCAGAACCTT-GTGAGAAATCAT-GAGTGTTTGGACTC
TGGGGGGAGTATGGTCGCAAGTCTGAAACTTAAAGGAATTGACGGAAGGGCACACAATGG
AGTGGAA-CCTGCGGCTTAATTT-GACTCAACTCGGGAAA-ACTTACCAAGCTCAGATAT
AATAAGGATTGACAGA-CTAA-AAGATCTTTCATGATTGTATAAGTGGTGGTGCATGGTC
GTTCTTAG-TTGGTGGAGTGATTTGTCTGGTCAATTCCGATAACGGACGAGACCTCTACT
TACTAACTAGTGGTATTTATTTGGTCAATATGGGAGATAGCTATT-TGGTGTTGGT-GTT
AGGC--CGTCAAAA-GTTTA---GC-ATTCTTCATTGAG-TGGTGTGTATTCTGATCAGA
TAG--GTACTAAAATTTTAAATA---AA-CTTCTTAGAAGGACTACCT-ACCTCAAGTGG
GGGG-AAGTCGGAGGCAATAACAGGTCTGTGATGCCCTTAGATACCTT-GGGCTGCACGC
GCGTTACAATGTAGGCGAGAAAAAGGT----TTCC--------GGAACCGA-AAG---GT
TTC-GGTAATCATTTGAATTGCCTACGTAATGGGGATTA-ATTTTTGTAATTATCGATTA
TCAACGAGGAATTCCTTGTAAGCGTAAATCATTACTTTACGCTGAATATGTCCCTGCCCT
TTGTACACACCGCCCGTCGCTCCTACCGATCGAACGATTAGGTAAAACTGACGGACTA--
--GGC-GACTTT--C----AAC-C-TGGTTGATCCT-------GC--GGT-GAACC--TG
-CA-GAAGGAT--C----A-----C--
>AM168097.1_Heterostelium_asymetricum_OH567
TCTAAG-TATAA-CCTTTA-TACGGTGAAA----CTGCAGACGGCTCATT--ACAA--CA
---GT----GATAAACTA-AAGAACTTTCGC-GCTTC-----GGCGTCTT-GGATAACCG
CAGTAAATCGGGGCTAATACATGTAAA-CG-AGAGGATGAGCAGG----CAACTGCG-AG
TCTTTGCGATTGTT----A-GCTATTCATT---CACACCAACCTCTTCGGAGTTTGTGGT
GAGTCCGAACAATATTGCTGATCGGAA--------ACT-TGTTTCCGACGAGTTCTTTGT
GTCACTGCCCTATCAACTTTCGATGGTAAGGTATTGGCTTACCATGGTTGTAACGGGTGA
CGGGGAATCAGGGTTCGATTCCGGAGAGGGAGCCTGAGAAATGGCTACCACTTCTACGGA
AGGCAGCAGGCGCGCAAATT-ACTCAATCCCAATAC-GGGGAAGTAGTGACAAAAAATAC
TAATGCCTTTCCATTTATATGGGGG--GCAATTGGAATAAGTACAACTTAAATCGCTTAA
CG-AAAGTGATTGGAGGGCAAGTCTGGTGCCAGCAGCCGCGGTAATTCCAGCTCCAA-TA
GCGTATACTAAATTTGTTGCAGTTAAAA-AGC-TCGTAGTTGAGATTGAGATTTCTTG-G
GT-TTAG--CGTCCAAGATTGCCTTCACGGGTTAATCTTT-TGGT-ACGTAAAGCTTTTC
AGTGGAATTCT-ATTT-CA-TTGTTAAAGTTA-CCAAGGAATTTCTATCTGC-CCA--TT
TAAGGTGGCAACACTT---TA-AATTTAGGGTGATCTACTGTGAGAAAA-TTGTAGTGTT
CAAAGCA--GG-C---GTT---TT-CGCTTGTTCAATGCAGCATGGTATAGTAAAATATG
ACAC---TAAATATAT-GTTGGTTG-TATA---TT-T-TAGTGTAATGACTAATAGGGAA
GGGCGGGGCCGTTCATATTGATGGGCGAGAGGTGAAATTCGTTGACCCTATCAAGATGCA
CTACAGCGAAAGCATTCGGCAAGTACTTCTCCATTAATCAAGAACGAAAGTTTGGGGATC
GAAGACGATCAGATACCGTCGTAGTCCAAACCATAAACTATGTCGACCAGGGATTGGACG
GATAAT-TTTTTAAAAACTCGCTCAGAACCTT-GTGAGAAATCAT-GAGTGTTTGGACTC
TGGGGGGAGTATGGTCGCAAGTCTGAAACTTAAAGGAATTGACGGAAGGGCACACAATGG
AGTGGAA-CCTGCGGCTTAATTT-GACTCAACTCGGGAAA-ACTTACCAAGCTCAGATAT
AATAAGGATTGACAGA-CTAA-AAGATCTTTCATGATTGTATAAGTGGTGGTGCATGGTC
GTTCTTAG-TTGGTGGAGTGATTTGTCTGGTCAATTCCGATAACGGACGAGACCTCTACT
TACTAACTAGTGGTATTTATTTGGTCAATATGGGAGATAGCTATT-TGGTGTTGGT-GTT
AGGC--CGTCAAAA-GTTTA---GC-ATTCTTCATTGAG-TGGTGTGTATTCTGATCAGA
TAG--GTACTAAAATTTTAAATA---AA-CTTCTTAGAAGGACTACCT-ACCTCAAGTGG
GGGG-AAGTCGGAGGCAATAACAGGTCTGTGATGCCCTTAGATACCTT-GGGCTGCACGC
GCGTTACAATGTAGGCGAGAAAAAGGT----TTCC--------GGAACCGA-AAG---GT
TTC-GGTAATCATTTGAATTGCCTACGTAATGGGGATTA-ATTTTTGTAATTATCGATTA
TCAACGAGGAATTCCTTGTAAGCGTAAATCATTACTTTACGCTGAATATGTCCCTGCCCT
TTGTACACACCGCCCGTCGCTCCTACCGATCGAACGATTAGGTAAAACTGACGGACTA--
--GGC-GACTTT--CT---CGC-A-AGG-GATTGTT-------GTT-TGG-GAAG--TTA
-GT-TAAATCT--C----ATT--GTTT
>MN752217.1_Heterostelium_multibrachiatum_5916lun
TCTAAG-TATAA-CCTTTA-TACGGTGAAA----CTGCAGACGGCTCATT--ACAA--CA
---GT----GATAAACTA-AAGAACTTCCGC-GCTTC-----GGCGTTTT-GGATAACCG
CAGTAAATCGGGGCTAATACATGTAAA-CG-AGAGGATGAACGGG----CAACTGTG-AG
TCTTTGCGATTGTT----A-GCT-TTAAT----CA--CCAACCTCTTCGGAGTCAGTGGT
GAATCCCTACAATATTGCTGATCGGAA--------ACT-TGTTTCCGACGAGTTCTTTGT
GTCACTGCCCTATCAACTTTCGATGGTAAGGTATTGGCTTACCATGGTTGTAACGGGTGA
CGGGGAATCAGGGTTCGATTCCGGAGAGGGAGCCTGAGAAATGGCTACCACTTCTACGGA

AGGCAGCAGGCGCGCAAATT-ACTCAATCCCAATAC-GGGGAAGTAGTGACAAAAAATAC
TAATGCCTTTCCATTTATATGGGGG--GCAATTGGAATAAGTACAATTTAAATCGCTTAA
CG-AAAGTGATTGGAGGGCAAGTCTGGTGCCAGCAGCCGCGGTAATTCCAGCTCCAA-TA
GCGTATACTAAATTTGTTGCAGTTAAAA-AGC-TCGTAGTTGAGATTGAGATTTCTCA-G
GT-TTAG--CGGTCAACATTGCCTTCACGGGTTAATG-TT-TGAT-ACGTAAAGCTTTAT
AGTAGAATTTC-ATTTT-A-TTATTAAAGTTA-CTGAGGAATTTCCAACTGC-CCA--TG
GTAGCCAGCAATGGTTA--TC-AATA--GGGTGATCTACTGTGAGAAAA-TTGTAGTGTT
CAAAGCA--GG-C---GTC---TTTCGTTTGTTCAATGCAGCATGGTATAGTTAAATATG
ACAC---TAAATATAT-GTTGGTTG-TATA---T-CT-TAGTGTAATGACTAATAGGGAA
GGGCGGGGCCGTTCATATTGATGGGCGAGAGGTGAAATTCGTTGACCCTATCAAGATGCA
CTACAGCGAAAGCATTCGGCAAGTACTTCTCCATTAATCAAGAACGAAAGTTTGGGGATC
GAAGACGATCAGATACCGTCGTAGTCCAAACCATAAACTATGTCGACCAGGGATTGGACG
GATAAT-TTTTTAAAAACTCGCTCAGAACCTT-GTGAGAAATCAT-GAGTGTTTGGACTC
TGGGGGGAGTATGGTCGCAAGTCTGAAACTTAAAGGAATTGACGGAAGGGCACACAATGG
AGTGGAA-CCTGCGGCTTAATTT-GACTCAACTCGGGAAA-ACTTACCAAGCTCAGATAT
AATAAGGATTGACAGA-CTAA-AAGATCTTTCATGATTGTATAAGTGGTGGTGCATGGTC
GTTCTTAG-TTGGTGGAGTGATTTGTCTGGTCAATTCCGATAACGGACGAGACCTCTACT
TACTAACTAGTGATATTTATTTGGTCAATATGGGAGATAGTCATT-TGGTGTTGGTAGT-
AGGA--CGTCAAAA-TCTTA----CTATTCTTCATTGAG-TGGTGTGTATTCTGATCAGA
TAG--GTACTAATTTTAAAAATA---AA-CTTCTTAGAAGGACTACCT-ACCTCAAGTGG
GGGG-AAGTCGGAGGCAATAACAGGTCTGTGATGCCCTTAGATACCTT-GGGCTGCACGC
GCGTTACAATGTAGGCGAGAAAAAGGT----TTCC--------GGAATCGA-AAG---GT
TTC-GGTAATCAATTGAATTGCCTACGTAATGGGGATTA-ATTTTTGTAATTATCGATTA
TCAACGAGGAATTCCTTGTAAGCGTAAATCATTACTTTACGCTGAATATGTCCCTGCCCT
TTGTACACACCGCCCGTCGCTCCTACCGATCGAACGATTAGGTAAAACTGACGGACTA--
--GGC-GACTCT--TC---CGC-A-AGG-ATTTGTT-------GTT-TGG-GAAG--TTA
-GT-TAAATCT--C----ATT--GTTT
>MN752218.1_Heterostelium_multibrachiatum_5904lun
TCTAAG-TATAA-CCTTTA-TACGGTGAAA----CTGCAGACGGCTCATT--ACAA--CA
---GT----GATAAACTA-AAGAACTTCCGC-GCTTC-----GGCGTTTT-GGATAACCG
CAGTAAATCGGGGCTAATACATGTAAA-CG-AGAGGATGAACGGG----CAACTGTG-AG
TCTTTGCGATTGTT----A-GCT-TTAAT----CA--CCAACCTCTTCGGAGTCAGTGGT
GAATCCCTACAATATTGCTGATCGGAA--------ACT-TGTTTCCGACGAGTTCTTTGT
GTCACTGCCCTATCAACTTTCGATGGTAAGGTATTGGCTTACCATGGTTGTAACGGGTGA
CGGGGAATCAGGGTTCGATTCCGGAGAGGGAGCCTGAGAAATGGCTACCACTTCTACGGA
AGGCAGCAGGCGCGCAAATT-ACTCAATCCCAATAC-GGGGAAGTAGTGACAAAAAATAC
TAATGCCTTTCCATTTATATGGGGG--GCAATTGGAATAAGTACAATTTAAATCGCTTAA
CG-AAAGTGATTGGAGGGCAAGTCTGGTGCCAGCAGCCGCGGTAATTCCAGCTCCAA-TA
GCGTATACTAAATTTGTTGCAGTTAAAA-AGC-TCGTAGTTGAGATTGAGATTTCTCA-G
GT-TTAG--CGGTCAACATTGCCTTCACGGGTTAATG-TT-TGAT-ACGTAAAGCTTTAT
AGTAGAATTTC-ATTTT-A-TTATTAAAGTTA-CTGAGGAATTTCCAACTGC-CCA--TG
GTAGCCAGCAATGGTTA--TC-AATA--GGGTGATCTACTGTGAGAAAA-TTGTAGTGTT
CAAAGCA--GG-C---GTC---TTTCGTTTGTTCAATGCAGCATGGTATAGTTAAATATG
ACAC---TAAATATAT-GTTGGTTG-TATA---T-CT-TAGTGTAATGACTAATAGGGAA
GGGCGGGGCCGTTCATATTGATGGGCGAGAGGTGAAATTCGTTGACCCTATCAAGATGCA
CTACAGCGAAAGCATTCGGCAAGTACTTCTCCATTAATCAAGAACGAAAGTTTGGGGATC
GAAGACGATCAGATACCGTCGTAGTCCAAACCATAAACTATGTCGACCAGGGATTGGACG
GATAAT-TTTTTAAAAACTCGCTCAGAACCTT-GTGAGAAATCAT-GAGTGTTTGGACTC
TGGGGGGAGTATGGTCGCAAGTCTGAAACTTAAAGGAATTGACGGAAGGGCACACAATGG
AGTGGAA-CCTGCGGCTTAATTT-GACTCAACTCGGGAAA-ACTTACCAAGCTCAGATAT
AATAAGGATTGACAGA-CTAA-AAGATCTTTCATGATTGTATAAGTGGTGGTGCATGGTC
GTTCTTAG-TTGGTGGAGTGATTTGTCTGGTCAATTCCGATAACGGACGAGACCTCTACT
TACTAACTAGTGATATTTATTTGGTCAATATGGGAGATAGTCATT-TGGTGTTGGTAGT-
AGGA--CGTCAAAA-TCTTA----CTATTCTTCATTGAG-TGGTGTGTATTCTGATCAGA
TAG--GTACTAATTTTAAAAATA---AA-CTTCTTAGAAGGACTACCT-ACCTCAAGTGG
GGGG-AAGTCGGAGGCAATAACAGGTCTGTGATGCCCTTAGATACCTT-GGGCTGCACGC
GCGTTACAATGTAGGCGAGAAAAAGGT----TTCC--------GGAATCGA-AAG---GT
TTC-GGTAATCAATTGAATTGCCTACGTAATGGGGATTA-ATTTTTGTAATTATCGATTA
TCAACGAGGAATTCCTTGTAAGCGTAAATCATTACTTTACGCTGAATATGTCCCTGCCCT
TTGTACACACCGCCCGTCGCTCCTACCGATCGAACGATTAGGTAAAACTGACGGACTA--
--GGC-GACTCT--TC---CGC-A-AGG-ATTTGTT-------GTT-TGG-GAAG--TTA
-GT-TAAATCT--C----ATT--GTTT
>AM168096.1_Heterostelium_anisocaule_NZ47B
TCTAAG-TATAA-CCTTTA-TACGGTGAAA----CTGCAGACGGCTCATT--ACAA--CA
---GT----GATAAACTA-AAGAACTTCCGC-GCTTC-----GGCGTTTT-GGATAACCG
CAGTAAATCGGGGCTAATACATGTAAA-CG-AGAGGATGAACGGG----CAACTGTG-AG
TCTTTGCGATTGTT----A-GCT-TTAAT----CA--CCAACCTCTTCGGAGTCGGTGGT
GAATCCCTACAATATTGCTGATCGGAA--------ACT-TGTTTCCGACGAGTTCTTTGT
GTCACTGCCCTATCAACTTTCGATGGTAAGGTATTGGCTTACCATGGTTGTAACGGGTGA
CGGGGAATCAGGGTTCGATTCCGGAGAGGGAGCCTGAGAAATGGCTACCACTTCTACGGA
AGGCAGCAGGCGCGCAAATT-ACCCAATCCCAATAC-GGGGAAGTAGTGACAAAAAATAC
TAATGCCTTTCCATTTATATGGGGG--GCAATTGGAATAAGTACAATTTAAATCGCTTAA
CG-AAAGTGATTGGAGGGCAAGTCTGGTGCCAGCAGCCGCGGTAATTCCAGCTCCAA-TA

GCGTATACTAAATTTGTTGCAGTTAAAA-AGC-TCGTAGTTGAGATTGAGATTTCTCA-G
GT-TTAG--CGGTCAACATTGCCTTCACGGGTTAATG-TT-TGAT-ACGTAAAGCTTTAT
AGTAGAATTTC-ATTTT-A-TTATTAAAGTTA-CTGAGGAATTTCCAACTGC-CCA--TG
GTAGCCAGTAATGGTTA--TC-AATA--GGGTGATCTACTGTGAGAAAA-TTGTAGTGTT
CAAAGCA--GG-C---GTC---TTTCGTTTGTTCAATGCAGCATGGTATAGTTAAATATG
ACAC---TAGATATAT-GTTGGTTG-TATA---TTGT-TAGTGTAATGACTAATAGGGAA
GGGCGGGGCCGTTCATATTGATGGGCGAGAGGTGAAATTCGTTGACCCTATCAAGATGCA
CTACAGCGAAAGCATTCGGCAAGTACTTCTCCATTAATCAAGAACGAAAGTTTGGGGATC
GAAGACGATCAGATACCGTCGTAGTCCAAACCATAAACTATGTCGACCAGGGATTGGACG
GATAAT-TTTTTAAAAGCTCGCTCAGAACCTT-GTGAGAAATCAT-GAGTGTTTGGACTC
TGGGGGGAGTATGGTCGCAAGTCTGAAACTTAAAGGAATTGACGGAAGGGCACACAATGG
AGTGGAA-CCTGCGGCTTAATTT-GACTCAACTCGGGAAA-ACTTACCAAGCTCAGATAT
AATAAGGATTGACAGA-CTAA-AAGATCTTTCATGATTGTATAAGTGGTGGTGCATGGTC
GTTCTTAG-TTGGTGGAGTGATTTGTCTGGTCAATTCCGATAACGGACGAGACCTCTACT
TACTAACTAGTGATATTTATTTGGTCAATATGGGAGATAGTCATT-TGGTGTTGGTAGT-
AGGA--CGTCAAAA-TCTTA----CTATTCTTCATTGAG-TGGTGTGTATTCTGATCAGA
TAG--GTACTAATTTTAAAAATA---AA-CTTCTTAGAAGGACTACCT-ACCTCAAGTGG
GGGG-AAGTCGGAGGCAATAACAGGTCTGTGATGCCCTTAGATACCTT-GGGCTGCACGC
GCGTTACAATGTAGGCGAGAAAAAGGT----TTCC--------GGAATCGA-AAG---GT
TTC-GGTAATCAATTGAATTGCCTACGTAATGGGGATTA-ATTTTTGTAATTATCGATTA
TCAACGAGGAATTCCTTGTAAGCGTAAATCATTACTTTACGCTGAATATGTCCCTGCCCT
TTGTACACACCGCCCGTCGCTCCTACCGATCGAACGATTAGGTAAAACTGACGGACTA--
--GGC-GACTCT--TC---CGC-A-AGG-ATTTGTT-------GTT-TGG-GAAG--TTA
-GT-TAAATCT--C----ATT--GTTT
>AM168107.1_Heterostelium_pseudocandidum_TNS_C_91
TCTAAG-TATAA-CCTTTA-TACGGTGAAA----CTGCAGACGGCTCATT--ACAA--CA
---GT----GATAAACTA-AAGAACTTCCGC-GCTTC-----GGCGTTTT-GGATAACCG
CAGTAAATCGGGGCTAATACATGTAAA-CG-AGAGGATGAACGGG----CAACTGTG-AG
TCTTTGCGATTGTT----A-GCT-TTAAT----CA--CCAACCTCTTCGGAGTCAGTGGT
GAATCCCTACAATATTGCTGATCGGAA--------ACT-TGTTTCCGACGAGTTCTTTGT
GTCACTGCCCTATCAACTTTCGATGGTAAGGTATTGGCTTACCATGGTTGTAACGGGTGA
CGGGGAATCAGGGTTCGATTCCGGAGAGGGAGCCTGAGAAATGGCTACCACTTCTACGGA
AGGCAGCAGGCGCGCAAATT-ACTCAATCCCAATAC-GGGGAAGTAGTGACAAAAAATAC
TAATGCCTTTCCATTTATATGGGGG--GCAATTGGAATAAGTACAATTTAAATCGCTTAA
CG-AAAGTGATTGGAGGGCAAGTCTGGTGCCAGCAGCCGCGGTAATTCCAGCTCCAA-TA
GCGTATACTAAATTTGTTGCAGTTAAAA-AGC-TCGTAGTTGAGATTGAGATTTCTCA-G
GT-TTAG--CGGTCAACATTGCCTTTACGGGTTAATG-TT-TGAT-ACGTAAAGCTTTAT
AGTAGAATTTC-ATTTT-A-TTATTAAAGTTA-CTGAGGAATTTCCAACTGC-CCA--TG
GTAGCCAGCAATGGTTA--TC-AATA--GGGTGATCTACTGTGAGAAAA-TTGTAGTGTT
CAAAGCA--GG-C---GTC---TTTCGTTTGTTCAATGCAGCATGGTATAGTTAAATATG
ACAC---TAAATATAT-GTTGGTTG-TATA---T-CT-TAGTGTAATGACTAATAGGGAA
GGGCGGGGCCGTTCATATTGATGGGCGAGAGGTGAAATTCGTTGACCCTATCAAGATGCA
CTACAGCGAAAGCATTCGGCAAGTACTTCTCCATTAATCAAGAACGAAAGTTTGGGGATC
GAAGACGATCAGATACCGTCGTAGTCCAAACCATAAACTATGTCGACCAGGGATTGGACG
GATAAT-TTTTTAAAAACTCGCTCAGAACCTT-GTGAGAAATCAT-GAGTGTTTGGACTC
TGGGGGGAGTATGGTCGCAAGTCTGAAACTTAAAGGAATTGACGGAAGGGCACACAATGG
AGTGGAA-CCTGCGGCTTAATTT-GACTCAACTCGGGAAA-ACTTACCAAGCTCAGATAT
AATAAGGATTGACAGA-CTAA-AAGATCTTTCATGATTGTATAAGTGGTGGTGCATGGTC
GTTCTTAG-TTGGTGGAGTGATTTGTCTGGTCAATTCCGATAACGGACGAGACCTCTACT
TACTAACTAGTGATATTTATTTGGTCAATATGGGAGATAGTCATT-TGGTGTTGGTAGT-
AGGA--CGTCAAAA-TCTTA----CTATTCTTCATTGAG-TGGTGTGTATTCTGATCAGA
TAG--GTACTAATTTTAAAAATA---AA-CTTCTTAGAAGGACTACCT-ACCTCAAGTGG
GGGG-AAGTCGGAGGCAATAACAGGTCTGTGATGCCCTTAGATACCTT-GGGCTGCACGC
GCGTTACAATGTAGGCGAGAAAAAGGT----TTCC--------GGAATCGA-AAG---GT
TTC-GGTAATCAATTGAATTGCCTACGTAATGGGGATTA-ATTTTTGTAATTATCGATTA
TCAACGAGGAATTCCTTGTAAGCGTAAATCATTACTTTACGCTGAATATGTCCCTGCCCT
TTGTACACACCGCCCGTCGCTCCTACCGATCGAACGATTAGGTAAAACTGACGGACTA--
--GGC-GACTCT--TC---CGC-A-AGG-ATTTGTT-------GTT-TGG-GAAG--TTA
-GT-TAAATCT--C----ATT--GTTT
>KP167482.1_Heterostelium_pseudoplasmodiofascium
TCTAAG-TATAA-CCTTTC-TACGGTGAAG----CTGCAGAAGGATCAAGT-ACAA--CA
--TGT---AGATAAACTA-AAGAACTTCCGC-GCTTC-----GGCGTTTT-GGATAACCG
CAGTAAATCGGGGCTAATACATGTAAA-CG-AGAGGATGAACGGG----CAACTGTG-AG
TCTTTGCGATTGTT----A-GCT-TTAAT----CA--CCAACCTCTTCGGAGTCAGTGGT
GAATCCCTACAATATTGCTGATCGGAA--------ACT-TGTTTCCGACGAGTTCTTTGT
GTCACTGCCCTATCAACTTTCGATGGTAAGGTATTGGCTTACCATGGTTGTAACGGGTGA
CGGGGAATCAGGGTTCGATTCCGGAGAGGGAGCCTGAGAAATGGCTACCACTTCTACGGA
AGGCAGCAGGCGCGCAAATT-ACTCAATCCCAATAC-GGGGAAGTAGTGACAAAAAATAC
TAATGCCTTTCCATTTATATGGGGG--GCAATTGGAATAAGTACAATTTAAATCGCTTAA
CG-AAAGTGATTGGAGGGCAAGTCTGGTGCCAGCAGCCGCGGTAATTCCAGCTCCAA-TA
GCGTATACTAAATTTGTTGCAGTTAAAA-AGC-TCGTAGTTGAGATTGAGATTTCTCA-G
GT-TTAG--CGGTCGGCAT-GCCTTCACGGGTT-TTGC-T-TGAT-ACGTAAAGCTTTAT
AGTAGAATTTC-ATTTT-A-TTATTAAAGTTA-CTGAGGAATTTCCAACTGC-CCA--TG

GTTGTCAGCAATGGCTT--CC-AATA--GGGTGATCTACTGTGAGAAAA-TTGTAGTGTT
CAAAGCA--GG-C---GTC---TTTCGTTTGTTCAATGCAGCATGGTATAGTTAAATATG
ACAC---TAAATATAT-GTTGGTTG-TATA---TTCT-TAGTGTAATGACTAATAGGGAA
GGGCGGGGCCGTTCATATTGATGGGCGAGAGGTGAAATTCGTTGACCCTATCAAGATGCA
CTACAGCGAAAGCATTCGGCAAGTACTTCTCCATTAATCAAGAACGAAAGTTTGGGGATC
GAAGACGATCAGATACCGTCGTAGTCCAAACCATAAACTATGTCGACCAGGGATTGGACG
GATAAT-TTTTTAAAAACTCGCTCAGAACCTT-GTGAGAAATCAT-GAGTGTTTGGACTC
TGGGGGGAGTATGGTCGCAAGTCTGAAACTTAAAGGAATTGACGGAAGGGCACACAATGG
AGTGGAA-CCTGCGGCTTAATTT-GACTCAACTCGGGAAA-ACTTACCAAGCTCAGATAT
AATAAGGATTGACAGA-CTAA-AAGATCTTTCATGATTGTATAAGTGGTGGTGCATGGTC
GTTCTTAG-TTGGTGGAGTGATTTGTCTGGTCAATTCCGATAACGGACGAGACCTCTACT
TACTAACTAGTGATATTTATTTGGCCAATATGGGAGATAGTCATT-TGGTGTTGGTAGT-
AGGA--CGTCAAAA-TCTT----GCTATTCTTCATTGAG-TGGTGTGTATTCTGGTCAGA
TAG--GTACTAATTTTAAAAATA---AA-CTTCTTAGAAGGACTACCT-ACCTCAAGTGG
GGGG-AAGTCGGAGGCAATAACAGGTCTGTGATGCCCTTAGATACCTT-GGGCTGCACGC
GCGTTACAATGTAGGCGAGAAAAAGGT----TTCC--------GGAATCGA-AAG---GT
TTC-GGTAATCAATTGAATTGCCTACGTAATGGGGATTA-ATTTTTGTAATTATCGATTA
TCAACGAGGAATTCCTTGTAAGCGTAAATCATTACTTTACGCTGAATATGTCCCTGCCCT
TTGTACACACCGCCCGTCGCTCCTACCGATCGAACGATTAGGTAAAACTGACGGACTA--
--GGC-GACTCTCTGT---A-C-C-TGG---TTGAT-------CCT-------------
----------------------GCC-
>KP167483.1_Heterostelium_parvimigratum
TCTAAG-TATAA-CCTTTA-TACGGTGAAA----CTGCAGACGGATCAAT--ACAA--CA
---GT----GATAAACTA-AAGAACTTCCGC-GCTTC-----GGCGTTTT-GGATAACCG
CAGTAAATCGGGGCTAATACATGTAAA-CG-AGAGGATGAACGGG----CAACTGTG-AG
TCTTTGCGATTGTT----A-GCT-TTAAT----CA--CCAACCTCTTCGGAGTCAGTGGT
GAATCCCTACAATATTGCTGATCGGAA--------ACT-TGTTTCCGACGAGTTCTTTGT
GTCACTGCCCTATCAACTTTCGATGGTAAGGTATTGGCTTACCATGGTTGTAACGGGTGA
CGGGGAATCAGGGTTCGATTCCGGAGAGGGAGCCTGAGAAATGGCTACCACTTCTACGGA
AGGCAGCAGGCGCGCAAATT-ACTCAATCCCAATAC-GGGGAAGTAGTGACAAAAAATAC
TAATGCCTTTCCATTTATATGGGGG--GCAATTGGAATAAGTACAATTTAAATCGCTTAA
CG-AAAGTGATTGGAGGGCAAGTCTGGTGCCAGCAGCCGCGGTAATTCCAGCTCCAA-TA
GCGTATACTAAATTTGTTGCAGTTAAAA-AGC-TCGTAGTTGAGATTGAGATTTCTCA-G
GT-TTAG--CGGTCGGCAT-GCCTTCACGGGTT-TTGC-T-TGAT-ACGTAAAGCTTTAT
AGTAGAATTTC-ATTTT-A-TTATTAAAGTTA-CTGAGGAATTTCCAACTGC-CCA--TG
GTTGTCAGCAATGGCTT--CC-AATA--GGGTGATCTACTGTGAGAAAA-TTGTAGTGTT
CAAAGCA--GG-C---GTC---TTTCGTTTGTTCAATGCAGCATGGTATAGTTAAATATG
ACAC---TAAATATAT-GTTGGTTG-TATA---TTCT-TAGTGTAATGACTAATAGGGAA
GGGCGGGGCCGTTCATATTGATGGGCGAGAGGTGAAATTCGTTGACCCTATCAAGATGCA
CTACAGCGAAAGCATTCGGCAAGTACTTCTCCATTAATCAAGAACGAAAGTTTGGGGATC
GAAGACGATCAGATACCGTCGTAGTCCAAACCATAAACTATGTCGACCAGGGATTGGACG
GATAAT-TTTTTAAAAACTCGCTCAGAACCTT-GTGAGAAATCAT-GAGTGTTTGGACTC
TGGGGGGAGTATGGTCGCAAGTCTGAAACTTAAAGGAATTGACGGAAGGGCACACAATGG
AGTGGAA-CCTGCGGCTTAATTT-GACTCAACTCGGGAAA-ACTTACCAAGCTCAGATAT
AATAAGGATTGACAGA-CTAA-AAGATCTTTCATGATTGTATAAGTGGTGGTGCATGGTC
GTTCTTAG-TTGGTGGAGTGATTTGTCTGGTCAATTCCGATAACGGACGAGACCTCTACT
TACTAACTAGTGATATTTATTTGGCCAATATGGGAGATAGTCATT-TGGTGTTGGTAGT-
AGGA--CGTCAAAA-TCTT----GCTATTCTTCATTGAG-TGGTGTGTATTCTGGTCAGA
TAG--GTACTAATTTTAAAAATA---AA-CTTCTTAGAAGGACTACCT-ACCTCAAGTGG
GGGG-AAGTCGGAGGCAATAACAGGTCTGTGATGCCCTTAGATACCTT-GGGCTGCACGC
GCGTTACAATGTAGGCGAGAAAAAGGT----TTCC--------GGAATCGA-AAG---GT
TTC-GGTAATCAATTGAATTGCCTACGTAATGGGGATTA-ATTTTTGTAATTATCGATTA
TCAACGAGGAATTCCTTGTAAGCGTAAATCATTACTTTACGCTGAATATGTCCCTGCCCT
TTGTACACACCGCCCGTCGCTCCTACCGATCGAACGATTAGGTAAAACTGACGGACTA--
--GGC-GACTCT--TT---A---------------------------------------

>KP167481.1_Heterostelium_migratissimum

---------GATAAACTA-AAGAACTTCCGC-GCTTC-----GGCGTTTT-GGATAACCG
CAGTAAATCGGGGCTAATACATGTAAA-CG-AGAGGATGAACGGG----CAACTGTG-AG
TCTTTGCGATTGTT----A-GCT-TTAAT----CA--CCAACCTCTTCGGAGTCAGTGGT
GAATCCCTACAATATTGCTGATCGGAA--------ACT-TGTTTCCGACGAGTTCTTTGT
GTCACTGCCCTATCAACTTTCGATGGTAAGGTATTGGCTTACCATGGTTGTAACGGGTGA
CGGGGAATCAGGGTTCGATTCCGGAGAGGGAGCCTGAGAAATGGCTACCACTTCTACGGA
AGGCAGCAGGCGCGCAAATT-ACTCAATCCCAATAC-GGGGAAGTAGTGACAAAAAATAC
TAATGCCTTTCCATTTATATGGGGG--GCAATTGGAATAAGTACAATTTAAATCGCTTAA
CG-AAAGTGATTGGAGGGCAAGTCTGGTGCCAGCAGCCGCGGTAATTCCAGCTCCAA-TA
GCGTATACTAAATTTGTTGCAGTTAAAA-AGC-TCGTAGTTGAGATTGAGATTTCTCA-G
GT-TTAG--CGGTCGGCAT-GCCTTCACGGGTT-TTGC-T-TGAT-ACGTAAAGCTTTAT
AGTAGAATTTC-ATTTT-A-TTATTAAAGTTA-CTGAGGAATTTCCAACTGC-CCA--TG
GTTGTCAGCAATGGCTT--CC-AATA--GGGTGATCTACTGTGAGAAAA-TTGTAGTGTT
CAAAGCA--GG-C---GTC---TTTCGTTTGTTCAATGCAGCATGGTATAGTTAAATATG
ACAC---TAAATATAT-GTTGGTTG-TATA---TTCT-TAGTGTAATGACTAATAGGGAA

GGGCGGGGCCGTTCATATTGATGGGCGAGAGGTGAAATTCGTTGACCCTATCAAGATGCA
CTACAGCGAAAGCATTCGGCAAGTACTTCTCCATTAATCAAGAACGAAAGTTTGGGGATC
GAAGACGATCAGATACCGTCGTAGTCCAAACCATAAACTATGTCGACCAGGGATTGGACG
GATAAT-TTTTTAAAAACTCGCTCAGAACCTT-GTGAGAAATCAT-GAGTGTTTGGACTC
TGGGGGGAGTATGGTCGCAAGTCTGAAACTTAAAGGAATTGACGGAAGGGCACACAATGG
AGTGGAA-CCTGCGGCTTAATTT-GACTCAACTCGGGAAA-ACTTACCAAGCTCAGATAT
AATAAGGATTGACAGA-CTAA-AAGATCTTTCATGATTGTATAAGTGGTGGTGCATGGTC
GTTCTTAG-TTGGTGGAGTGATTTGTCTGGTCAATTCCGATAACGGACGAGACCTCTACT
TACTAACTAGTGATATTTATTTGGCCAATATGGGAGATAGTCATT-TGGTGTTGGTAGT-
AGGA--CGTCAAAA-TCTTA----CTATTCTTCATTGAG-TGGTGTGTATTCTGGTCAGA
TAG--GTACTAATTTTAAAAATA---AA-CTTCTTAGAAGGACTACCT-ACCTCAAGTGG
GGGG-AAGTCGGAGGCAATAACAGGTCTGTGATGCCCTTAGATACCTT-GGGCTGCACGC
GCGTTACAATGTAGGCGAGAAAAAGGT----TTCC--------GGAATCGA-AAG---GT
TTC-GGTAATCAATTGAATTGCCTACGTAATGGGGATTA-ATTTTTGTAATTATCGATTA
TCAACGAGGAATTCCTTGTAAGCGTAAATCATTACTTTACGCTGAATATGTCCCTGCCCT
TTGTACACACCGCCCGTCGCTCCTACCGATCGAACGATTAGGTAAAACTGACGGACTA--
--GGC-GAC--

>HQ141499.1_Heterostelium_boreale_BSB10A
TCTAAG-TATAAACCTTTA-TACGGTGAAC----CTGCAGAAGGATCAACT-ACAA--CT
-ATGT----GATAAACTA-AAGAACTTTCGC-GCTTC-----GGCGTCTT-GGATAACCG
CAGTAAATCGGGGCTAATACATATAAA-CG-AAAGGATGAACGGG----CAACTGTG-AG
TCTTTGCGATTGTT----A-GTT-TTCTTA---CA--CCAACCTCTTCGGAGTTTGTGGT
GAGTCCGAACAATATTGCTGATCG-AA--------ACT-TAGTTTCGACGAGTTCTTTGT
GTCACTGCCCTATCAACTTTCGATGGTAAGGTATTGGCTTACCATGGTTGTAACGGGTGA
CGGGGAATCAGGGTTCGATTCCGGAGAGGGAGCCTGAGAAATGGCTACCACTTCTACGGA
AGGCAGCAGGCGCGCAAATT-ACTCAATCCCAATAC-GGGGAAGTAGTGACAAAAAATAC
TAATGCCCTTCCATTT-TATGGGGG--GCAATTGGAATAAGTACAACTTAAATCGCTTAG
CA-AAAGTGATTGGAGGGCAAGTCTGGTGCCAGCAGCCGCGGTAATTCCAGCTCCAA-TA
GCGTATACTAAATTTGTTGCAGTTAAAA-AGC-TCGTAGTTGAGATTGAGATTTCTTA-G
GT-TTAAAGCTTCTATTTTTAGCTT--CGGTTAAATTTTAGTTGT-GTATAAAGCTTTTT
AGTAGACTTTT--GTTT-G-CTATTAAAGTTA-CTAAGAAATTTCTTTCTGC-CCA--TG
ATTTAGGTTCGCCCCTGAATC-AATC--GGGTGTTCTACTGTGAGAAAA-TTGTAGTGTT
TAAAGCA--GG-C---GTA---TT-AGTTTGTTCAATGCAGCATGGTATAGTAAAACAAG
ACAC---TAAACATTT-GTTGGTT--AATG---TTCTATAGTGTAATGACTAATAGGGAA
GGGCGGGGCCGTTCATATTGATGGGCGAGAGGTGAAATTCGTTGACCCTATCAAGATGCA
CTACAGCGAAAGCATTCGGCAAGTACTTCTCCATTAATCAAGAACGAAAGTTTGGGGATC
GAAGACGATCAGATACCGTCGTAGTCCAAACCATAAACTATGTCGACCAGGGACTGGACG
GTTAAT-TTTATAAAAACTCATTCAGAACCTT-GTGGGAAACCAT-GAGTGTTTGGACTC
TGGGGGGAGTATGGTCGCAAGTCTGAAACTTAAAGGAATTGACGGAAGGGCACACAATGG
AGTGGAA-CCTGCGGCTTAATTT-GACTCAACTCGGGAAA-ACTTACCAAGCTCAGATAT
AATAAGGATTGACAGA-CTAA-AAGATCTTTCATGATTGTATAAGTGGTGGTGCATGGTC
GTTCTTAG-TTGGTGGAGTGATTTGTCTGGTCAATTCCGATAACGGACGAGACCTCTACC
TACTAACTAGTGGTACTTATTTGGTCATTATGGGAGATAGTTAGT-TGGTGTTGGTAGTT
AGAT---TTC---G-GTTTA---ATTATTCTTCATTGATCTAGTGTGTATCCTGGTCGGA
TAA--GTACTAACTAAAAAAATA---AAACTTCTTAGAGGGACTACCT-ACCTCAAGTGG
GGGG-AAGTCGGAGGCAATAACAGGTCTGTGATGCCCTTAGATACCTT-GGGCTGCACGC
GCGTTACAATGTAAGTTGGAAAAAGGT----TTCC--------GGTATCGA-AAG---GT
ATC-GGTAATCATTTGAATAGCTTACGTAATGGGGATTA-ATCTTTGTAATTATCGGTTA
TCAACGAGGAATTCCTTGTAAGCGTAAATCATTACTTTACGCTGAATATGTCCCTGCCCT
TTGTACACACCGCCCGTCGCTCCTACCGATCGAACGATTAGGTAAAACTGACGGACTA--
--TGT-GATTTT-------A--------------------------GGT-GAACC--TG
-CA-GAAGGAT--C----A--------
>HQ141497.1_Heterostelium_oculare_oculare
TCTAAG-TATAAACCTTTA-TACGGTGAAC----CTGCAGAAGGATCAACTCACAG--TT
--TGT----GATAAACTA-AAGAACTTTCGC-GCTTA-----GGCGTCTT-GGATAACCG
CAGTAAATCGGGGCTAATACATATAAA-CG-AAAGGATGAGCGGG----TAACCGCG-AG
TCTTTGCGATTGTT----A-GTTTTGCTTTT-TCATACCAACCTCCTCGGAGTTTGTGTT
GAATCCGAACAATATTGCTGATCGGAA--------ACT-TGTTTCCGACGAGTTCTATGT
GTCACTGCCCTATCAACTTTCGATGGTAAGGTATTGGCTTACCATGGTTGTAACGGGTGA
CGGGGAATCAGGGTTCGATTCCGGAGAGGGAGCCTGAGAAATGGCTACCACTTCTACGGA
AGGCAGCAGGCGCGCAAATT-ACTCAATCCCAATAC-GGGGAAGTAGTGACAAAAAATAC
TAATGCCCTTCCATTTTTATGGGGG--GCAATTGGAATAAGTACAACTTAAATCGCTTAG
CA-AAAGTGATTGGAGGGCAAGTCTGGTGCCAGCAGCCGCGGTAATTCCAGCTCCAA-TA
GCGTATACTAAATTTGTTGCAGTTAAAA-AGC-TCGTAGTTTAGATTGAGATTTTTTG-G
GT-TTAAAGC--TCATCTTTTTGCTTAACGGTAATTAGGTGTAGT-GTATAAAGCTTTTA
TGTAGACTTCGC-GTTT-A-TATATAAAGTTA-CCAAAAAATTTCATTCTGC-CCA--T-
--ACTAAAATTTATTTTA-GT-AATC--GGGTGTTCTACTGTGAGAAAA-TTGTAGTGTT
TAAAGCA--GG-C---GTC---TT-TGTTTGTTCAATGCAGCATGGTATAGTAAAAAAAG
ACAC---TAAACATTT-GTTGGTT--AATG---TT-A-TAGTGTAATGACTAATAGGGAA
GGGCGGGGCCGTTCATATTGATGGGCGAGAGGTGAAATTCGTTGACCCTATCAAGATGCA
CTACAGCGAAAGCATTCGGCAAGTACTTCTCCATTAATCAAGAACGAAAGTTTGGGGATC
GAAGACGATCAGATACCGTCGTAGTCCAAACCATAAACTATGTCGACCAGGGACTGGACG

GTTAAT-TTTATAAAAACTCGCTCAGAACCTT-GTGGGAAACCAT-GAGTGTTTGGACTC
TGGGGGGAGTATGGTCGCAAGTCTGAAACTTAAAGGAATTGACGGAAGGGCACACAATGG
AGTGGAA-CCTGCGGCTTAATTT-GACTCAACTCGGGAAA-ACTTACCAAGCTCAGATAT
AATAAGGATTGACAGA-CTAA-AAGATCTTTCATGATTGTATAAGTGGTGGTGCATGGTC
GTTCTTAG-TTGGTGGAGTGATTTGTCTGGTCAATTCCGATAACGGACGAGACCTCTACC
TACTAACTAGTGGTACTTATTTAGTCAATATGGGGGATAGCTAGT-TGGTGTTGGTAATT
AGTC---TTC-ACG-GATTA---ATTATCTTTCATTGATCTAGTGTGTACTCTGATTAGA
TAA--GTACTAACTAAAAAAATA---AAACTTCTTAGAGGGACTACCT-ACCTCAAGTGG
GGGG-AAGTCGGAGGCAATAACAGGTCTGTGATGCCCTTAGATACCTT-GGGCTGCACGC
GCGTTACAATGTAAGTGAGAAAAAGGT----TTCC--------GGTATCGA-AAG---GT
ATC-GGTAATCATTTGAATTACCTACGTAATGGGGATTG-ATCTTTGTAATTATCGATCA
TCAACGAGGAATTCCTTGTAAGCGTAAATCATTACTTTACGCTGAATATGTCCCTGCCCT
TTGTACACACCGCCCGTCGCTCCTACCGATCGAACGCTTAGGTAAAACTGACGGACTA--
--TAT-GATATT-------AACGC-TGG---TTGATACTCTGTCCGATGTAGATACGATA
TGT-CTTGTCT-CC----AAA--GATT
>AM168079.1_Heterostelium_oculare_DB4B
TCTAAG-TATAAACCTTTA-TACGGTGAAA----CTGCAGACGGCTCATT--ACAA--CA
---GT----GATAAACTA-AAGAACTTTCGC-GCTTA-----GGCGTCTT-GGATAACCG
CAGTAAATCGGGGCTAATACATATAAA-CG-AAAGGATGAGCGGG----TAACCGCG-AG
TCTTTGCGATTGTT----A-GTTTTGCTTTT-TCATACCAACCTCCTCGGAGTTTGTGTT
GAATCCGAACAATATTGCTGATCGGAA--------ACT-TGTTTCCGACGAGTTCTATGT
GTCACTGCCCTATCAACTTTCGATGGTAAGGTATTGGCTTACCATGGTTGTAACGGGTGA
CGGGGAATCAGGGTTCGATTCCGGAGAGGGAGCCTGAGAAATGGCTACCACTTCTACGGA
AGGCAGCAGGCGCGCAAATT-ACTCAATCCCAATAC-GGGGAAGTAGTGACAAAAAATAC
TAATGCCCTTCCATTTTTATGGGGG--GCAATTGGAATAAGTACAACTTAAATCGCTTAG
CA-AAAGTGATTGGAGGGCAAGTCTGGTGCCAGCAGCCGCGGTAATTCCAGCTCCAA-TA
GCGTATACTAAATTTGTTGCAGTTAAAA-AGC-TCGTAGTTTAGATTGAGATTTTTTG-G
GT-TTAAAGC--TCATCTTTTTGCTTAACGGTAATTAGGTGTAGT-GTATAAAGCTTTTA
TGTAGACTTCGC-GTTT-A-TATATAAAGTTA-CCAAAAAATTTCATTCTGC-CCA--T-
--ACTAAAATTTATTTTA-GT-AATC--GGGTGTTCTACTGTGAGAAAA-TTGTAGTGTT
TAAAGCA--GG-C---GTC---TT-TGTTTGTTCAATGCAGCATGGTATAGTAAAAAAAG
ACAC---TAAACATTT-GTTGGTT--AATG---TT-A-TAGTGTAATGACTAATAGGGAA
GGGCGGGGCCGTTCATATTGATGGGCGAGAGGTGAAATTCGTTGACCCTATCAAGATGCA
CTACAGCGAAAGCATTCGGCAAGTACTTCTCCATTAATCAAGAACGAAAGTTTGGGGATC
GAAGACGATCAGATACCGTCGTAGTCCAAACCATAAACTATGTCGACCAGGGACTGGACG
GTTAAT-TTTATAAAAACTCGCTCAGAACCTT-GTGGGAAACCAT-GAGTGTTTGGACTC
TGGGGGGAGTATGGTCGCAAGTCTGAAACTTAAAGGAATTGACGGAAGGGCACACAATGG
AGTGGAA-CCTGCGGCTTAATTT-GACTCAACTCGGGAAA-ACTTACCAAGCTCAGATAT
AATAAGGATTGACAGA-CTAA-AAGATCTTTCATGATTGTATAAGTGGTGGTGCATGGTC
GTTCTTAG-TTGGTGGAGTGATTTGTCTGGTCAATTCCGATAACGGACGAGACCTCTACC
TACTAACTAGTGGTACTTATTTAGTCAATATGGGGGATAGCTAGT-TGGTGTTGGTAATT
AGTC---TTC-ACG-GATTA---ATTATCTTTCATTGATCTAGTGTGTACTCTGATTAGA
TAA--GTACTAACTAAAAAAATA---AAACTTCTTAGAGGGACTACCT-ACCTCAAGTGG
GGGG-AAGTCGGAGGCAATAACAGGTCTGTGATGCCCTTAGATACCTT-GGGCTGCACGC
GCGTTACAATGTAAGTGAGAAAAAGGT----TTCC--------GGTATCGA-AAG---GT
ATC-GGTAATCATTTGAATTACCTACGTAATGGGGATTG-ATCTTTGTAATTATCGATCA
TCAACGAGGAATTCCTTGTAAGCGTAAATCATTACTTTACGCTGAATATGTCCCTGCCCT
TTGTACACACCGCCCGTCGCTCCTACCGATCGAACGCTTAGGTAAAACTGACGGACTA--
--TAT-GATATT--AT---AGC-A-ATATGATACTTG------CTG-TGG-GAAG--TTA
-GT-TAAATCT--C----ATT--GTTT
>AM168077.1_Speleostelium_caveatum_WS695
TCTAAG-TATAAATCTTTA-TACGGTGAAA----CTGCAGACGGCTCATT--ACAA--TA
---GT----GATAAACTA-ATAGATTTTCGG-GTTTA-------CCTTTT-GGATAACCG
CAGTAAATCGGGGCTAATACATAGAAG-CG-AGGGG-TGACTGGC-----AAC-G-G-AA
GCTCCGCGATTATT----A-GTT-TAT--TA-CCAATCC---CGCAA-GGGCTAG-TGGT
GAAACCGAATAATATTGCTGATCGACA--------TGT-ATTTGTCGACAAATCTACTGT
GTCACTACCCTATCAACTTTCGATGGTACGGTATTGGCCTACCATGGTTGTAACGGGTAA
CGGGGAATCAGGATTCGATTCCGGAGAGGGAGCCTGAGAAATGGCTACCACTTCTACGGA
AGGCAGCAGGCGCGCAAATT-ACTCAATCCCAATAC-GGGGAAGTAGCGACAATAAATAT
TAATGCCCATCTATTT-TATAGAGG--GCAATTAAAATGGACAAAATATAAACTACATAG
TT-AATACAATTGGAGGGCAAGTCTGGTGCCAGCAGCCGCGGTAATTCCAGCTCCAA-TA
GCATATACTAAAGTTGTTGCAGTTAAAA-AGC-TCGTAGTTGAAATTTGGGCTATTAA-G
GG-CTCA----------AACCAACATCCACTTTATAG---------TGTTTGTA-----T
GGTTCC-TTATA-G--ACT-CAACT--TGA---CTCAGTATAGTCTACTTTT-----GTA
GTTCTG--TATCTG---------------AGTACTTCACTGTGAGAAAA-TTGTGGTGTT
TAAAGCA--AG-C---GTA---TC-GCTTGATCTTTTGCAGCATGGTATGATAAAACATG
ATCT--GCGTCAAC--CATTGGTAG--TTG----ACTGTA-TGTAATGATTAATAGGGGA
GGATGGGGATGTTCATATTGATGGGCGAGAGGTGAAATTCGTTGACCCTATCAAGATGAA
CTTCTGCGAAAGCATTCATCAAATACTCCCCCATTAATCAAGAACGAAAGTTTGGGGATC
AAAGACGTTCAGATACCGTCGTAGTCCAAACTATAAACGATGTCGACCAGGGATCAGCCG
AT--A--TTTATAAAAATGCGGTTGGCACCTT-GTGGGAAACCAT-GAGTTTTTAGACTC
TGGGGGAAGTATGGTCGCAAGTCTGAAACTTAAAGGAATTGACGGAAGGGCACACAATGG
AGTGGAG-CCTGCGGCTTAATTT-GACTCAACTCGGGAAA-ACTTACCAAGCTAAGATAT

AGTTAGGATTGACAGA-CTAA-AAGATCTTTCATGATTCTATAAGTGGTGGTGCATGGTC
GTTCTTAG-TTGGTGGAGCAATCTGTCTGGTCAATTCCGATAACGGACGAGATCTCGACC
TGCTAACTAGTATCACTTATCA-ACCAATATAGTCGGTACCTTTTCCTATGCTG--ACTT
TGTTT-ACTC-AGGGTG------AC-GG-GTGGGTTTAG----AATATTGGCTGGTTAGA
TAA--GTGAGATAAA----ATTA---AAACTTCTTAGAGGGACTACCTTGCGGTAAGCAG
GTGG-AAGTTCGAGGCAATAACAGGTCTGTGATGCCCTTAGATA----------------

>AM168073.1_Tieghemostelium_menorah_M1
TCTAAG-TATAAATCTTTA-TACGGTGAAA----CTGCAGACGGCTCATT--ACAA--CA
---GT----GATAAACTA-ATAGAGTTTCGG-GTCTAA---C--CTACCT-GGATATCCG
CAGTAAATCGGGGCTAATACATACAAA-CG-AGGGG-TGACTGTT-----TAC-G-G-AA
GCTCCGCGATTATT----A-GAT-T----AA-CCAATGC--CCGCAAGGGTTTTG-TGGT
GAAACCGAATAATATTGCAGATCGAAG--------CCT--AGCTTCGACAATTCTATTGT
GTCACTGCCCTATCAACTTTCGATGGTACGGTATTGGCCTACCATGGTTTTTACGGGTAA
CGGAGAATTAGGGTTCGATTCCGGAGAGGGCGCCTGAGAAATGGCGACCACTTCTATGGA
AGGCAGCAGGCGCGCAAATT-ACTCAATCCCAATAC-GGGGAAGTAGTGACAATAAATAT
TGATGCCTATTCGTTT-TTCGAAAG--GTAATTGAAATGAGTACAAATTAAATCTCTTAA
CT-AATACAATTGGAGGGCAAGTCTGGTGCCAGCAGCCGCGGTAATTCCAGCTCCAA-TA
GCATATACTAAAGTTGTTGCGGTTAAAA-AGC-TCGTAGTTGAAAGTAAATTTACATT-G
GG-TTAA----------AGTCCACGTCCGCTTTGG-----------TGGTCGCT-----G
GTCTCCATTGTATCTTTTT-AAACT--TCACC-CTTAAGGCCT-CTACTTT------GTA
GC--GGGTTCTTTTTA------------GGGTACTTCACTGTGAGAAAA-TTGTGGTGTT
CAAAGCG--GG-C---GTC---T-CGCCTGATCTTTTGCAGCATGGTATGATGGAACAAG
ACAT---TTTGTGT--AATTGGTT---GCA----TTTAAAGTGTAATGATTAATAGGGGT
GGATGGGGGTGTTCATATTGGTGGGCGAGAGGTGAAATTCGCTGACCCTATCAAGATGAA
CTTCTGCGAAAGCATTCACCAAATACTCCCCCATTAATCAAGAACGAAAGTTTGGGGATC
AAAGACGATCAGATACCGTCGTAGTCCAAACTATAAACTATGTCGACCAGGGATCGGTTA
CT--AT-TTTTTAAAAATTTGATCGGCACCTT-GTGAGAAATCAT-GAGTGTTTAGATTC
TGGGGGGAGTATGGTCGCAAGTCTGAAACTTAAAGGAATTGACGGAAGGGCACACAATGG
AGTGGAG-CCTGCGGCTTAATTT-GACTCAACTCGGGAAA-ACTTACCAAGCTAAGATAT
AGTAAGGATTGACAGA-CTAA-AAGATTTTTCATGATTCTATAAGTGGTGGTGCATGGTC
GTTCTTAG-TTGGTGGAGCAATCTGTCTGGTCAATTCCGATAACGGACGAGATCTCGACC
TGCTAACTAGTAGTACCTATTAATTCGATATGAATGAAAGCTCATTCGGAGTAAGTTAGT
CAT----CGCAAGG-TGT-----CTGACACTTCGGTGGG----TCGGTAATTTGATTTGA
TAG--GTACGATAT----ATAAA------CTTCTTAGAGGGACTACCT-GTGGCAAACAG
GGGG-AAGTTCGAGGCAATAACAGGTCTGTGATGCCCTTAGATACCTT-GGGCCGCACGC
GCGCTACAATGTAATAGGCAAAAAGCT-----AT-----CC--TGGTCCGG-AAG---GA
TT-GGGTAATCATATGAATTTATTACGTAACTGGGCTTG-ATCTTTGTAATTATTGATCA
TCAACGAGGAATTCCTTGTAAGCGCAAATCATTACTTTGTGCTGAATATGTCCCTGCCCT
TTGTACACACCGCCCGTCGCTCCTACCGATCGAATGATACGGTAAAGCCAACGGATAA--
--G-----G--C---TCTGCAG-C-AAT-GCGT---------GAGTTTCA-AAAG--TTG
-TT-TAAATCT--C----ATT--GTTT
>JF892725.1_Tieghemostelium_unicornutum_OH599

--------AGATAAACTA-ATAGAGTTTCGG-GTCTAA---C--CTTTAT-GGATATCCG
CAGTAAATCGGGGCTAATACATACAAA-CG-AGGGG-TGACTGTT-----TAC-A-G-AA
GCTTCGCGATTATT----A-GTC-T----AA-CCAATAC--CCGCAAGGGTCTAG-TGGT
GAAACCGAATAATATTGCAGATCGAAA--------CCT--AGTTTCGACGATTCTATTGT
GTCACTGCCCTATCAACTTTCGATGGTACGGTATTGGCCTACCATGGTTGTAACGGGTAA
CGGGGAATTAGGGTTCGATTCCGGAGAGGGCGCCTGAGAAATGGCGACCACTTCTACGGA
AGGCAGCAGGCGCGCAAATT-ACTCAATCCCAATAC-GGGGAAGTAGTGACAATAAATAT
CAATGCCTATTCGTTT-TTCGAAAG--GTAATTGAAATGGGTACAAATTAAACCCCTTAA
CC-AATACAATTGGAGGGCAAGTCTGGTGCCAGCAGCCGCGGTAATTCCAGCTCCAA-TA
GCATATACTAAAGTTGTTGCGGTTAAAA-AGC-TCGTAGTTGAAACTAAATTTGTATT-G
GG-CTCA----------AGCTTGTAGCCACTTTGG-----------TGGTTATC-----A
AGTTCCAGTGCATTTTTTT-GAAGC--G-GCC-CTTAAAGCCT-CTACCTC------GTA
GT--TGGTTTTCTT--------------GGGTTTTTCACTGTGAGAAAA-TTGTGGTGTT
CAAAGCG--GG-C---GTT---T-CGCCTGATCTTTTGCAGCATGGGATGATAAAACATG
ACAT---TCAATGC--AATTGGTT---GCA----TCTG-AGTGTAATGATTAATAGGGAT
GGATGGGGGTGTTCATATTGGTGGGCGAGAGGTGAAATTCGTTGACCCTATCAAGATGAA
CTTCTGCGAAAGCATTCATCAAATACTTCCCCATTAATCAAGAACGAAAGTTTGGGGATC
AAAGACGATCAGATACCGTCGTAGTCCAAACTATAAACTATGTCGACCAGGGATCGGGTA
GC--AT-TTTTCAAAAATCTATTTGGCACCTT-GTGAGAAATCAT-GAGTGTTTAGATTC
TGGGGGGAGTATGGTCGCAAGTCTGAAACTTAAAGGAATTGACGGAAGGGCACACAATGG
AGTGGAG-CCTGCGGCTTAATTT-GACTCAACTCGGGAAA-ACTTACCAAGCTAAGATAT
AGTAAGGATTGACAGA-CTAA-AAGATTTTTCATGATTCTATAAGTGGTGGTGCATGGTC
GTTCTTAG-TTGGTGGAGCAATCTGTCTGGTCAATTCCGATAACGGACGAGATCTCGACC
TGCTAACTAGTAGTACGTATTTGGTCGATATGGGTGAAGGCTCTTTTGGAGTAAGTTGTG

CGT----CGCAAGG-TGT-----ACAGCACTTCTGGGGG----TTTGTAACCTGGTCAGA
TAC--GTACGATTAA---AAAAA------CTTCTTAGAGGGACTACCT-GTGGCAAACAG
GGGG-AAGTTCGAGGCAATAACAGGTCTGTGATGCCCTTAGATACCTT-GGGCCGCACGC
GCGCTACAATGTAAAAGGCAAAAAGCT-----TT-----CC--TGGTCCGG-AAG---GA
CT-GGGTAATCATCTGAATTTTTTACGTAACTGGGATTG-ATCTTTGTAATTATTGATCA
TCAACGAGGAATTCCTTGTAAGCGCAAATCATTACTTTGTGCTGAATAGGTCCCTGCCCT
TTGTACACACCGCCCGTCGCTCCTACCGATCGAATGATACGGTAAAGCCAACAGATA---
----------------------C-----------------------------G----G
----------T--C-------------
>AM168045.1_Tieghemostelium_lacteum
TCTAAG-TATAAATCTTTA-TACGGTGAAA----CTGCAGACGGCTCATT--ACAA--CA
---GT----GATAAACTA-ATAGAGTTTCGG-GTCTAA---C--CTACCT-GGATATCCG
CAGTAAATCGGGGCTAATACATACAAA-CG-AGGGG-TGACTGTT-----TAC-G-G-GA
GCTCCGCGATTATT----A-GTC-T----AG-CCAATGC--CCGCAAGGGTTTTG-TGGT
GAAACCGAATAATATTGCAGATCGAAA--------TCT--GATTTCGACAATTCTATTGT
GTCACTGCCCTATCAACTTTCGATGGTACGGTATTGGCCTACCATGGTTGTAACGGGTAA
CGGAGAATTAGGGTTCGATTCCGGAGAGGGCGCCTGAGAAATGGCGACCACTTCTACGGA
AGGCAGCAGGCGCGCAAATT-ACTCAATCCCAATAC-GGGGAAGTAGTGACAATAAATAT
TAATGCCTATTCGTTT-TTCGAAAG--GTAATTAAAATGGGTACAAATTAAATCCATTAA
CT-AATACAATGGGAGGGCAAGTCTGGTGCCAGCAGCCGCGGTAATTCCAGCTCCAA-TA
GCATATACTAAAGTTGTTGCGGTTAAAA-AGC-CCGTAGTTGAAATTAAATTTGCATT-G
GG-CCAA----------AGTTTCTAGCCACTTTGG-----------TGGTTACG-----A
AATTCCAGTGCATTTTTTT-AAACC--T-ACC-CTTAAAGCCT-CTACTTT------GTA
GT--TGGTTTTCTT--------------GGGTACTTCACTGTGAGAAAA-TTGTGGTGTT
TAAAGCG--GG-C---GTC---T-CGCCTGATCTTTTGCAGCATGGTATGATAAAACATG
ACAT---TTTGTGC--AATTGGTT---GCA----TTTAAAGTGTAATGATTAATAGGGAT
GGATGGGGGTGTTCATATTGGTGGGCGAGAGGTGAAATTCGTTGACCCTATCAAGATGAA
CTTCTGCGAAAGCATTCATCAAATACTTCCCCATTAATCAAGAACGAAAGTTTGGGGATC
AAAGACGACCAGATACCGTCGTAGTCCAAACTATAAACTATGTCGACCAGGGATCGGTTA
AT--AT-TTTTTAAAAATTTACTCGGCACCTT-GTGAGAAATCAT-GAGTGTTTAGATTC
TGGGGGGAGTATGGTCGCAAGTCTGAAACTTAAAGGAGTTGACGGAAGGGCACACAATGG
AGTGGAG-CCTGCGGCTTAATTT-GACTCAACTCGGGAAA-ACTTACCAAGCTAAGATAT
AGTAAGGATTGACAGA-CTAA-AAGATTTTTCATGATTCTATAAGTGGTGGTGCATGGTC
GTTCTTAG-TTGGTGGAGCAATCTGTCTGGTCAATTCTGATAACGGACGAGATCTCGACA
CGCTAACTAGTAGTACTTATTCGGTCGATATGAGTGAAGGCTCTCAAGGAGTAAGTTGTG
CGT----CGAAAGG-TGT-----ATGACACTTCGGGGGG----TTTGTAACTTGACCAGA
TAT--GTACGATTTT---AAAAA------CTTCTTAGAGGGACTACCT-GTGGCAAACAG
GGGG-AAGTTCGAGGCAATAACAGGTCTGTGATGCCCTTAGATACCTT-GGGCCGCACGC
GCGCTACAATGTAATAGGCAAAAAGCT-----CT-----CC--TGGTCCGG-AAG---GA
TT-GGGTAATCATATGAATTTATTACGTAACTGGGCTTG-ATCTTTGTAATTATTGATCA
TCAACGAGGAATTCCTTGTAAGCGCAAATCATTACTTTGTGCTGAATATGTCCCTGCCCT
TTGTACACACCGCCCGTCGCTCTTACCGATCGAATGATACGGTAAAGCCAACGGATAA--
--GAT----------TCTGTAG-C-AAT-ACGT---------GAATTTTA-AAAG--TTG
-TT-TAAATCT--C----ATT--GTTT
>JF892717.1_Tieghemostelium_montium_57a

---------GATAAACTA-ATAGAGTTTCGG-GTCTAA---C--CTACAT-GGATATCCG
CAGTAAATCGGGGCTAATACATACAAA-CG-AGGGG-TGACTGTT-----TAC-G-G-AA
GCTCCGCGATTATT----A-GTC-T----AG-CCAATAC--CCGCAAGGGTTTTG-TGGT
GAAACCGAATAATATTGCAGATCGAAA--------TCT--GATTTCGACAATTCTATTGT
GTCACTGCCCTATCAACTTTCGATGGTACGGTATTGGCCTACCATGGTTGTAACGGGTAA
CGGAGAATTAGGGTTCGATTCCGGAGAGGGCGCCTGAGAAATGGCGACCACTTCTACGGA
AGGCAGCAGGCGCGCAAATT-ACTCAATCCCAATAC-GGGGAAGTAGTGACAATAAATAT
TAATGCCTATTCGTTT-TTCGAAAG--GTAATTAAAATGGGTACAAATTAAATCCATTAA
CT-AATACAATTGGAGGGCAAGTCTGGTGCCAGCAGCCGCGGTAATTCCAGCTCCAA-TA
GCATATACTAAAGTTGTTGCGGTTAAAA-AGC-TCGTAGTTGAAACTAAATTTGCATT-G
GG-TCAA----------AGTTTCTAGCCACTTTGG-----------TGGTTACG-----A
AATTCCAGTGCATTTTTTT-AAATC--T-GCC-CTTAAAGCCT-CTACTTT------GTA
GT--TGGTTTTCTT--------------GGGTACTTCACTGTGAGAAAA-TTGTGGTGTT
TAAAGCG--GG-C---GTC---T-CGCCTGATCTTTTGCAGCATGGTATGATAAAACATG
ACAT---TTTGTGC--AATTGGTT---GCA----TTTAAAGTGTAATGATTAATAGGGAT
GGATGGGGGTGTTCATATTGGTGGGCGAGAGGTGAAATTCGTTGACCCTATCAAGATGAA
CTTCTGCGAAAGCATTCATCAAATACTTCCCCATTAATCAAGAACGAAAGTTTGGGGATC
AAAGACGATCAGATACCGTCGTAGTCCAAACTATAAACTATGTCGACCAGGGATCGGTTA
AT--AT-TTTTTAAAAATTTACTCGGCACCTT-GTGAGAAATCAT-GAGTGTTTAGATTC
TGGGGGGAGTATGGTCGCAAGTCTGAAACTTAAAGGAATTGACGGAAGGGCACACAATGG
AGTGGAG-CCTGCGGCTTAATTT-GACTCAACTCGGGAAA-ACTTACCAAGCTAAGATAT
AGTAAGGATTGACAGA-CTAA-AAGATTTTTCATGATTCTATAAGTGGTGGTGCATGGTC
GTTCTTAG-TTGGTGGAGCAATCTGTCTGGTCAATTCCGATAACGGACGAGATCTCGACC
TGCTAACTAGTAGTACTTATTCGGTCGATATGAGTGAAGGCTCTCAGGGAGTAAGTTGTG
CGT----CGAAAGG-TGT-----ATGACACTTCGGGGGG----TTTGTAACTTGACCAGA
TAT--GTACGATTTT---AAAAA------CTTCTTAGAGGGACTACCT-GTGGCAAACAG
GGGG-AAGTTCGAGGCAATAACAGGTCTGTGATGCCCTTAGATACCTT-GGGCCGCACGC

GCGCTACAATGTAATAGGCAAAAAGCT-----CT-----CC--TGGTCCGG-AAG---GA
TT-GGGTAATCATATGAATTTATTACGTAACTGGGCTTG-ATCTTTGTAATTATTGATCA
TCAACGAGGAATTCCTTGTAAGCGCAAATCATTACTTTGCGCTGAATATGTCCCTGCCCT
TTGTACACACCGCCCGTCGCTCCTACCGATCGAATGATACGGTAAAGCCAACGGATAA--
--GAT------------------C-----TGGT---------GAAC-CTG-C--G--TTG
-TC-C---G----G----ACC---CGT
>JF892716.1_Tieghemostelium_angelicum_38B0
TCTAAG-TATAAATTTTTA-TACGGTGAAC----CTGCAGACGGATCAACTCACGAG-CA
--TGT---AGATAAACTA-ATAGAGTTTCGG-GTCTAA---C--CTACAT-GGATATCCG
CAGTAAATCGGGGCTAATACATACAAA-CG-AGGGG-TGACTGTT-----TAC-G-G-AA
GCTCCGCGATTATT----A-GTC-T----AG-CCAATAC--CCGCAAGGGTTTTG-TGGT
GAAACCGAATAATATTGCAGATCGAAA--------TCT--GATTTCGACAATTCTATTGT
GTCACTGCCCTATCAACTTTCGATGGTACGGTATTGGCCTACCATGGTTGTAACGGGTAA
CGGAGAATTAGGGTTCGATTCCGGAGAGGGCGCCTGAGAAATGGCGACCACTTCTACGGA
AGGCAGCAGGCGCGCAAATT-ACTCAATCCCAATAC-GGGGAAGTAGTGACAATAAATAT
TAATGCCTATTCGTTT-TTCGAAAG--GTAATTAAAATGGGTACAAATTAAATCCATTAA
CT-AATACAATTGGAGGGCAAGTCTGGTGCCAGCAGCCGCGGTAATTCCAGCTCCAA-TA
GCATATACTAAAGTTGTTGCGGTTAAAA-AGC-TCGTAGTTGAAACTAAATTTGCATT-G
GG-TCAA----------AGTTTCTAGCCACTTTGG-----------TGGTTACG-----A
AATTCCAGTGCATTTTTTT-AAATC--T-GCC-CTTAAAGCCT-CTACTTT------GTA
GT--TGGTTTTCTT--------------GGGTACTTCACTGTGAGAAAA-TTGTGGTGTT
TAAAGCG--GG-C---GTC---T-CGCCTGATCTTTTGCAGCATGGTATGATAAAACATG
ACAT---TTTGTGC--AATTGGTT---GCA----TTTAAAGTGTAATGATTAATAGGGAT
GGATGGGGGTGTTCATATTGGTGGGCGAGAGGTGAAATTCGTTGACCCTATCAAGATGAA
CTTCTGCGAAAGCATTCATCAAATACTTCCCCATTAATCAAGAACGAAAGTTTGGGGATC
AAAGACGATCAGATACCGTCGTAGTCCAAACTATAAACTATGTCGACCAGGGATCGGTTA
AT--AT-TTTTTAAAAATTTACTCGGCACCTT-GTGAGAAATCAT-GAGTGTTTAGATTC
TGGGGGGAGTATGGTCGCAAGTCTGAAACTTAAAGGAATTGACGGAAGGGCACACAATGG
AGTGGAG-CCTGCGGCTTAATTT-GACTCAACTCGGGAAA-ACTTACCAAGCTAAGATAT
AGTAAGGATTGACAGA-CTAA-AAGATTTTTCATGATTCTATAAGTGGTGGTGCATGGTC
GTTCTTAG-TTGGTGGAGCAATCTGTCTGGTCAATTCCGATAACGGACGAGATCTCGACC
TGCTAACTAGTAGTACTTATTCGGTCGATATGAGTGAAGGCTCTCAGGGAGTAAGTTGTG
CGT----CGAAAGG-TGT-----ATGACACTTCGGGGGG----TTTGTAACTTGACCAGA
TAT--GTACGATTTT---AAAAA------CTTCTTAGAGGGACTACCT-GTGGCAAACAG
GGGG-AAGTTCGAGGCAATAACAGGTCTGTGATGCCCTTAGATACCTT-GGGCCGCACGC
GCGCTACAATGTAATAGGCAAAAAGCT-----CT-----CC--TGGTCCGG-AAG---GA
TT-GGGTAATCATATGAATTTATTACGTAACTGGGCTTG-ATCTTTGTAATTATTGATCA
TCAACGAGGAATTCCTTGTAAGCGCAAATCATTACTTTGCGCTGAATATGTCCCTGCCCT
TTGTACACACCGCCCGTCGCTCCTACCGATCGAATGATACGGTAAAGCCAACGGATAA--
--GAT-----------CT-TAA-C---C-TGGTT--------GATC-CTG-CCAG--TAG
-TC-ATATGCT--T----GTC--TCAA
>JF892722.1_Tieghemostelium_dumosum_OH602

----------ATAAACTA-ATAGAGTTTCGG-GTCTAA---C--CTAC-T-GGATATCCG
CAGTAAATCGGGGCTAATACATACAAA-CG-AGGGG-TGACTGTT-----TAC-G-G-GA
GCTCCGCGATTATT----A-GTC-T----AG-CCAATAC--CCGCAAGGGTTTTG-TGGT
GAAACCGAATAATATTGCAGATCGAAA--------TCT--AATTTCGACAATTCTATTGT
GTCACTGCCCTATCAACTTTCGATGGTACGGTATTGGCCTACCATGGTTGTAACGGGTAA
CGGAGAATTAGGGTTCGATTCCGGAGAGGGCGCCTGAGAAATGGCGACCACTTCTACGGA
AGGCAGCAGGCGCGCAAATT-ACTCAATCCCAATAC-GGGGAAGTAGTGACAATAAATAT
TAATGCCTATTCGTTT-TTCGAAAG--GTAATTAAAATGGGTACAAATTAAATCCCTTAA
CT-AATACAATTGGAGGGCAAGTCTGGTGCCAGCAGCCGCGGTAATTCCAGCTCCAA-TA
GCATATACTAAAGTTGTTGCGGTTAAAA-AGC-TCGTAGTTGAAACTAAATTTGTATT-G
GG-TCCA----------AGTTCCTAGCCACTTTGG-----------TGGTTACG-----G
AATTCCAGTGCATTTTTTT-AAACC--T-GCC-CTTAAAGCCT-CTACTTT------GTA
GT--TGGTTTTCTT--------------G-GTACTTCACTGTGAGAAAA-TTGTGGTGTT
CAAAGCG--GG-C---GTC---T-CGCCTGATCTTTTGCAGCATGGTATGATAAAACATG
ACAT---TTTGTGC--AATTGGTT---GCA----TTT-AAGTGTAATGATTAATAGGGAT
GGATGGGGGTGTTCATATTGGTGGGCGAGAGGTGAAATTCGTTGACCCTATCAAGATGAA
CTTCTGCGAAAGCATTCATCAAATACTTCCCCATTAATCAAGAACGAAAGTTTGGGGATC
AAAGACGATCAGATACCGTCGTAGTCCAAACTATAAACTATGTCGACCAGGGATCGGTTA
AT--AT-TTTTTAAAAATTTACTCGGCACCTT-GTGAGAAATCAT-GAGTGTTTAGATTC
TGGGGGGAGTATGGTCGCAAGTCTGAAACTTAAAGGAATTGACGGAAGGGCACACAATGG
AGTGGAG-CCTGCGGCTTAATTT-GACTCAACTCGGGAAA-ACTTACCAAGCTAAGATAT
AGTAAGGATTGACAGA-CTAA-AAGATTTTTCATGATTCTATAAGTGGTGGTGCATGGTC
GTTCTTAG-TTGGTGGAGCAATCTGTCTGGTCAATTCCGATAACGGACGAGATCTCGACC
TGCTAACTAGTAGTACTTATTCGGTCGATATGAGTGAAGGCTCTCAAGGAGTAAGTTGTG
CGT----CGCAAGG-TGC-----ATGACACTTCGGGGGG----TTTGTAACTTGATCAGA
TAT--GTACGATTTT---AAAAA------CTTCTTAGAGGGACTACCT-GTGGCAAACAG
GGGG-AAGTTCGAGGCAATAACAGGTCTGTGATGCCCTTAGATACCTT-GGGCCGCACGC
GCGCTACAATGTAATAGGCAAAAAGCT-----CT-----CC--TGGTCCGG-AAG---GA
TT-GGGTAATCATATGAATTTATTACGTAACTGGGCTTG-ATCTTTGTAATTATTGATCA
TCAACGAGGAATTCCTTGTAAGCGCAAATCATTACTTTGCGCTGAATATGTCCCTGCCCT

TTGTACACACCGCCCGTCGCTCCTACCGATCGAATGATACGGTAAAGCCAACGGA-AA--
--------C--C---TCTGTAA-C---C-TGGTT--------GATC-CTG-CCAG--TAG
-TC-ATATGCT--T----GTC--TCAA
>JF892720.1_Tieghemostelium_simplex_OH598
TCTAAG-TATAAATCTTTA-TACGGTGAAC----CTGCAGAAGGATCAAC--CCGG----
-TT-T---AGATAAACTA-ATAGAGTTTCGG-GTCTAA---C--CTACCT-GGATATCCG
CAGTAAATCGGGGCTAATACATACAAA-CG-AGGGG-TGACTGTT-----TAC-G-G-GA
GCTCCGCGATTATT----A-GTC-T----AG-CCAATAC--CCGCAAGGGTTTTG-TGGT
GAAACCGAATAATATTGCAGATCGAAA--------TCT--AATTTCGACAATTCTATTGT
GTCACTGCCCTATCAACTTTCGATGGTACGGTATTGGCCTACCATGGTTGTAACGGGTAA
CGGAGAATTAGGGTTCGATTCCGGAGAGGGCGCCTGAGAAATGGCGACCACTTCTACGGA
AGGCAGCAGGCGCGCAAATT-ACTCAATCCCAATAC-GGGGAAGTAGTGACAATAAATAT
TAATGCCTATTCGTTT-TTCGAAAG--GTAATTAAAATGGGTACAAATTAAATCCCTTAA
CT-AATACAATTGGAGGGCAAGTCTGGTGCCAGCAGCCGCGGTAATTCCAGCTCCAA-TA
GCATATACTAAAGTTGTTGCGGTTAAAA-AGC-TCGTAGTTGAAACTAAATTTGTATT-G
GG-TCCA----------AGTTCCTAGCCACTTTGG-----------TGGTTACG-----A
A-TTCCAGTGCATTTTTTT-AAACC--T-GCC-CTTAAAGCCT-CTACTTT------GTA
GT--T-GTTTTCTT--------------GGGTACTTCACTGTGAGAAAA-TTGTGGTGTT
CAAAGCG--GG-C---GTC---T-CGCCTGATCTTTTGCAGCATGGTATGATAAAACATG
ACAT---TTTGTGC--AATTGGTT---GCA----TTT-AAGTGTAATGATTAATAGGGAT
GGATGGGGGTGTTCATATTGGTGGGCGAGAGGTGAAATTCGTTGACCCTATCAAGATGAA
CTTCTGCGAAAGCATTCATCAAATACTTCCCCATTAATCAAGAACGAAAGTTTGGGGATC
AAAGACGATCAGATACCGTCGTAGTCCAAACTATAAACTATGTCGACCAGGGATCGGTTA
AT--AT-TTTTTAAAAATTTACTCGGCACCTT-GTGAGAAATCAT-GAGTGTTTAGATTC
TGGGGGGAGTATGGTCGCAAGTCTGAAACTTAAAGGAATTGACGGAAGGGCACACAATGG
AGTGGAG-CCTGCGGCTTAATTT-GACTCAACTCGGGAAA-ACTTACCAAGCTAAGATAT
AGTAAGGATTGACAGA-CTAA-AAGATTTTTCATGATTCTATAAGTGGTGGTGCATGGTC
GTTCTTAG-TTGGTGGAGCAATCTGTCTGGTCAATTCCGATAACGGACGAGATCTCGACC
TGCTAACTAGTAGTACTTATTCGGTCGATATGAGTGAAGGCTCTCAAGGAGTAAGTTGTG
CGT----CGCAAGG-TGC-----ATGACACTTCGGGGGG----TTTGTAACTTGATCAGA
TAT--GTACGATTTT---AAAAA------CTTCTTAGAGGGACTACCT-GTGGCAAACAG
GGGG-AAGTTCGAGGCAATAACAGGTCTGTGATGCCCTTAGATACCTT-GGGCCGCACGC
GCGCTACAATGTAATAGGCAAAAAGCT-----CT-----CC--TGGTCCGG-AAG---GA
TT-GGGTAATCATATGAATTTATTACGTAACTGGGCTTG-ATCTTTGTAATTATTGATCA
TCAACGAGGAATTCCTTGTAAGCGCAAATCATTACTTTGCGCTGAATATGTCCCTGCCCT
TTGTACACACCGCCCGTCGCTCCTACCGATCGAATGATACGGTAAAGCCAACGTG-AA--
----T---CAA----GATCTAA-C---C-TGGTT--------GATC-CTG-CCAG--TAG
-TC-ATATGCT--T----GTC--TCAA
>AM168036.1_Hagiwaraea_coeruleostipes_CRLC53B
TCTAAG-TATAAATTTTTA-TATGATGAAA----CTGCAGAAGGCTCATT--ACAA--CA
---GT----GATAAACTA-CTAGACTTTCGG-GCCTC-----GGCCTTTT-GGATAACCG
CAGTAAATCGGGGCTAATACATACAAT-CG-ATGGG-TGACTGTT-----TAC-G-G-AA
TCTCAGCGATTATT----A-GCT-TTC--AG-CCAACAC--TCTTCGGAGTTTTG--GGT
GAACCCGGATAATATTGCAGATCGAAG--------CTTACGCTTTCGACAAGTCTAATGT
GTCACTGCCCTATCAACTTTCGATGGTACGGTATTGGCCTACCATGGTTGTAACGGGTAA
CGGGGAATTAGGGTTCGATTCCGGAGAGGGCGCCTGAGAAATGGCGACCACTTCTACGGA
AGGCAGCAGGCGCGCAAATT-ACTCAATCCCAATAC-GGGGAAGTAGTGACAAGAAATAT
TAATGCCTATCCTTTC-A-AGGAAG--GTAATTAAAATGGATTTAAACTAAACCCATTTT
TT-AATACAATTGGAGGGCAAGTCTGGTGCCAGCAGCCGCGGTAATTCCAGCTCCAA-TA
GCATATACTAAATTTGTTGCGGTTAAAA-AGC-TCGTAGTTGAAATTGAAATTATATT-G
GG-TTCA---------TAGGTTTTAGTCGACTTCGCGTC--------GGTTTAA-----A
ACTTCCAGTGTAAATTTTT-AACTT--TAATC-CAGGTTGCTAGTTACTTT------GTA
AT--TAGTTTTCTTT-------------GGATACTTCACTGTGAGGAAA-TTGTGGTGCT
TAAAGCA--GG-C---GTT---TTCGCTTGATCTTTTGCAGCATGGTATGATAGAATATG
ACAT---TTTATGT--AGTTGGCT--CGCA------TAAAGTGTAATGATTAATAGGGAT
GGATGGGGGTGTTCATATTGATGGGCGAGAGGTGAAATTCGTTGACCCTATCAAGATGAA
CTTCTGCGAAAGCATTCACCAAATACTTCTCCATTAATCAAGAACGAAAGTTTGGGGATC
AAAGACGATCAGATACCGTCGTAGTCCAAACTATAAACTATGTCGACCAGGGATCAGTTA
CT--AT-TTTTAAAAAAATATAATTGGCACCTT-GTGAGAAATCAT-GAGTGTTTAGATTC
TGGGGGGAGTATGGTCGCAAGTCTGAAACTTAAAGGAATTGACGGAAGGGCACACAATGG
AGTGGAG-CCTGCGGCTTAATTT-GACTCAACTCGGGAAA-ACTTACCAAGCTAAGATAT
AATAAGGATTGACAGA-CTAA-AAGATCTTTCATGATTCTATAAGTGGTGGTGCATGGTC
GTTCTTAG-TTGGTGGAGCAATCTGTCTGGTCAATTCCGATAACGGACGAGATCTCGACC
TGCTAACTAGTAGTACATATTGATTCGATATGCAAGAAAGATTTGTTGGAGCAAGTTAAA
GAT----CGAAAGG-TTT-----TTAGCTCTTCGCTGGT----TGAGTATTGTGGATTGA
TATA-GTGCGATAAATTTAAAAA---AAACTTCTTAGAGGGACTACCT-GTGGCAAACAG
GGGG-AAGTTCGAGGCAATAACAGGTCTGTGATGCCCTTAGATACCTT-GGGCCGCACGC
GCGCTACAATGTAGTACGCAAAATGTT-----CTAAAAACC--AAACCCGG-GAG---GG
TA-AGGTAATCATAT-AATTTACTACGTAACTGGGATTG-ATCTTTGTAATTTTTGATCA
TCAACGAGGAATTCCTTGTAAGCGCAAATCATTACTTTGTGCTGAATCTGTCCCTGCCCT
TTGTACACACCGCCCGTCGCTCCTACCGATCGAATGATACGGTAAAGCTAACAGATAC--
--GAT----T-----C-----G---CAA-G------------AATT-GTA-AAAG--TTA
-TT-TAAATCT--C----ATT--GTTT

>AM168063.1_Hagiwaraea_rhizopodium_AusKY_4
TCTAAG-TATAAATTTTTA-TATGATGAAA----CTGCAGAAGGCTCATT--ACAA--CA
---GT----GATAAACTA-CCAGACTTTCGG-GCCTTT----GGCCTTTT-GGATAACCG
CGGTAAATCGGGGCTAATACATACAAT-CG-AGGGG-TGACTGTT-----TAC-G-G-AA
TCTCCGCGATTATT----A-GCT-TTC--AG-CCAACAC--TCTTCGGAGTTTTG--GGT
GAACCCGGATAATATTGCAGATCGAAG--------CTTACGCTTTCGACAAGTCTGATGT
GTCACTGCCCTATCAACTTTCGATGGTACGGTATTGGCCTACCATGGTTGTAACGGGTAA
CGGAGAATTAGGGTTCGATTCCGGAGAGGGCGCCTGAGAAATGGCGACCACTTCTACGGA
AGGCAGCAGGCGCGCAAATT-ACTCAATCCCAATAC-GGGGAAGTAGTGACAAGAAATAT
TAATGCCTATCCC-TC-ACGGGAAG--GTAATTAAAATGGATTTAAACTAAACCCATTTT
TT-AATACAATTGGAGGGCAAGTCTGGTGCCAGCAGCCGCGGTAATTCCAGCTCCAA-TA
GCATATACTAAATTTGTTGCGGTTAAAA-AGC-TCGTAGTTGAAATTAAAATTATACT-G
GG-TTCA----------AGGTTTTAGTCGACTTCGG-TC--------GGTTTAA-----A
ACTTCCAGTGTAAATTTTA-AAACT---AATC-CAGGTTGCCAGTTACTTT------GTA
AT--TGGTTTTCTTT-------------GGATACTTCACTGTGAGGAAA-TTGTGGTGCT
TAAAGCA--AG-C---GTT---TTCGCTTGATCTTTTGCAGCATGGTATGATAGAATATG
ACAT---TTTATGT--AGTTGGCT--CGCA------TAAAGTGTAATGATTAATAGGGAT
GGATGGGGGTGTTCATATTGATGGGCGAGAGGTGAAATTCGTTGACCCTATCAAGATGAA
CTTCTGCGAAAGCATTCACCAAATACTTCTCCATTAATCAAGAACGAAAGTTTGGGGATC
AAAGACGATCAGATACCGTCGTAGTCCAAACTATAAACTATGTCGACCAGGGATCAGCTA
AT--AT-TTTTACAAAATATAGTTGGCACCTT-GTGAGAAATCAT-GAGTGTTTAGATTC
TGGGGGGAGTATGGTCGCAAGTCTGAAACTTAAAGGAATTGACGGAAGGGCACACAATGG
AGTGGAG-CCTGCGGCTTAATTT-GACTCAACTCGGGAAA-ACTTACCAAGCTAAGATAT
AATAAGGATTGACAGA-CTAA-AAGATCTTTCATGATTCTATAAGTGGTGGTGCATGGTC
GTTCTTAG-TTGGTGGAGCAATCTGTCTGGTCAATTCCGATAACGGACGAGATCTCGACC
TGCTAACTAGTAGTACATATTAGCTCGATATGCAAGAAAGGCTTGTTGGAGTAAGTTAGA
GAT----CGCAAGG-TTT-----CTAGCCCTTCGCTGGT----CGAGTATTGTGGGTTAA
TATA-GTGCGATAAATTTAAA--------CTTCTTAGAGGGACTACCT-GTGGCAAACAG
GGGG-AAGTTCGAGGCAATAACAGGTCTGTGATGCCCTTAGATACCTT-GGGCCGCACGC
GCGCTACAATGTAGTACGCAAAAGGTA-----CTTTT--CC--AAACCCGG-AAG---GG
TG-AGGTAATCCTAT-AATTTACTACGTAACTGGGCTTG-ATCTTTGTAATTTTTGATCA
TCAACGAGGAATTCCTTGTAAGCGCAAATCATTACTTTGTGCTGAATCTGTCCCTGCCCT
TTGTACACACCGCCCGTCGCTCCTACCGATCGAATGATACGGTAAAGCTAACAGATAT--
--G-T---C------C-----G---CAA-G------------GA-T-GTA-AAAG--TTA
-TT-TAAATCT--C----ATT--GTTT
>HQ141494.1_Hagiwaraea_radiculata_ML5A
TCTAAG-TATAAATTTTTA-TATGATGAAA----CTGCAGAAGGCTCATT--ACAA--CA
---GT----GATAAACTA-CCAGACTTTCGG-GCCTTT----GGCCTTTT-GGATAACCG
CAGTAAATCGGGGCTAATACATACAAT-CG-AGGGG-TGACTGTT-----TAC-G-G-AA
TCTCCGCGATTATT----A-GCT-TTC--AG-CCAACAC--TCTTCG-AGTTTTG--GGT
GAACCCGGATAATATTGCAGATCGAAG--------CTTACGCTTTCGACAAGTCTGATGT
GTCACTGCCCTATCAACTTTCGATGGTACGGTATTGGCCTACCATGGTTGTAACGGGTAA
CGGAGAATTAGGGTTCGATTCCGGAGAGGGCGCCTGAGAAATGGCGACCACTTCTACGGA
AGGCAGCAGGCGCGCAAATT-ACTCAATCCCAATAC-GGGGAAGTAGTGACAAGAAATAT
TAATGCCTATCCC-TC-ACGGGAAG--GTAATTAAAATGGATTTAAACTAAACCCATTTT
TT-AATACAATTGGAGGGCAAGTCTGGTGCCAGCAGCCGCGGTAATTCCAGCTCCAA-TA
GCATATACTAAATTTGTTGCGGTTAAAA-AGC-TCGTAGTTGAAATTAAAATTATACT-G
GG-TTCA----------AGGTTTTAGTCGACTTCGG-TC--------GGTTTAA-----A
ACTTCCAGTGTAAATTTTA-AAACT---AATC-CAGGTTGCCAGTTACTTT------GTA
AT--TGGTTTTCTTT-------------GGATACTTCACTGTGAGGAAA-TTGTGGTGCT
TAAAGCA--AG-C---GTT---TTCGCTTGATCTTTTGCAGCATGGTATGATAGAATATG
ACAT---TTTATGT--AGTTGGCT--CGCA------TAAAGTGTAATGATTAATAGGGAT
GGATGGGGGTGTTCATATTGATGGGCGAGAGGTGAAATTCGTTGACCCTATCAAGATGAA
CTTCTGCGAAAGCATTCACCAAATACTTCTCCATTAATCAAGAACGAAAGTTTGGGGATC
AAAGACGATCAGATACCGTCGTAGTCCAAACTATAAACTATGTCGACCAGGGATCAGCTA
AT--AT-TTTTACAAAATATAGTTGGCACCTT-GTGAGAAATCAT-GAGTGTTTAGATTC
TGGGGGGAGTATGGTCGCAAGTCTGAAACTTAAAGGAATTGACGGAAGGGCACACAATGG
AGTGGAG-CCTGCGGCTTAATTT-GACTCAACTCGGGAAA-ACTTACCAAGCTAAGATAT
AATAAGGATTGACAGA-CTAA-AAGATCTTTCATGATTCTATAAGTGGTGGTGCATGGTC
GTTCTTAG-TTGGTGGAGCAATCTGTCTGGTCAATTCCGATAACGGACGAGATCTCGACC
TGCTAACTAGTAGTACATATTAGCTCGATATGCAAGAAAGGCTTGTTGGAGTAAGTTAGA
GAT----CGCAAGG-TTT-----CTAGCCCTTCGCTGGT----CGAGTATTGTGGGTTAA
TATA-GTGCGATAAATTTAAA--------CTTCTTAGAGGGACTACCT-GTGGCAAACAG
GGGG-AAGTTCGAGGCAATAACAGGTCTGTGATGCCCTTAGATACCTT-GGGCCGCACGC
GCGCTACAATGTAGTACGCAAAAGGTA-----CTTTT--CC--AAACCCGG-AAG---GG
TG-AGGTAATCCTAT-AATTTACTACGTAACTGGGCTTG-ATCTTTGTAATTTTTGATCA
TCAACGAGGAATTCCTTGTAAGCGCAAATCATTACTTTGTGCTGAATCTGTCCCTGCCCT
TTGTACACACCGCCCGTCGCTCCTACCGATCGAATGATACGGTAAAGCTAACAGATAT--
--G-T---C------C-----G---CAA-G------------GACT-GTA-AAAG--TTA
-TT-TAAATCT--C----ATG--TTTT
>AM168062.1_Hagiwaraea_vinaceofusca_CC4
TCTAAG-TATAAATTTTTA-TATGATGAAA----CTGCAGAAGGCTCATT--ACAA--CA
---GT----GATAAACTA-CTAGACTTTCGG-GCCTT----CGGCCTTTT-GGATAACCG

CAGTAAATCGGGGCTAATACATACAAT-CG-AGGGG-TGACTGTT-----TAC-G-G-AA
TCTCCGCGATTATT----A-GCT-TTC--AG-CCAACAC--TCTTCGGAGTTTTG--GGT
GAACCCGGATAATATTGCAGATCGAAG--------CTTACGCTTTCGACAAGTCTAATGT
GTCACTGCCCTATCAACTTTCGATGGTACGGTATTGGCCTACCATGGTTGTAACGGGTAA
CGGAGAATTAGGGTTCGATTCCGGAGAGGGCGCCTGAGAAATGGCGACCACTTCTACGGA
AGGCAGCAGGCGCGCAAATT-ACTCAATCCCAATAC-GGGGAAGTAGTGACAAGAAATAT
TAATGCCTATCCC-TT-TCGGGAAG--GTAATTAAAATGGATTTAAACTAAACCCATTTT
TT-AATACAATTGGAGGGCAAGTCTGGTGCCAGCAGCCGCGGTAATTCCAGCTCCAA-TA
GCATATACTAAATTTGTTGCGGTTAAAA-AGC-TCGTAGTTGAAGTTAAAATTACACT-G
GG-TTCA----------AGGTTTTAATCGACTCTG--TC--------GGTTTAA-----A
ACTTCCAGTGTAGCTTTTA-CAACT---CATC-CAGGTTTCTTGGTACTTT------GTA
TC--AGGTTTTCTTT-------------GGATATTTCACTGCGAGGAAA-TTGTGGTGCT
TAAAGCA--GG-C---GTT---TTCGCTTGATCTTTTGCAGCATGGTATGATAGAATATG
ACAT---TTTATGT--AGTTGGCT--CGCA------TAAAGTGTAATGATTAATAGGGAT
GGATGGGGGTGTTCATATTGATGGGCGAGAGGTGAAATTCGTTGACCCTATCAAGATGAA
CTTCTGCGAAAGCATTCACCAAATACTTCTCCATTAATCAAGAACGAAAGTTTGGGGATC
AAAGACGATCAGATACCGTCGTAGTCCAAACTATAAACTATGTCGACCAGGGATCAGCTA
AT--AT-TTTTAAAAAAATATAGTTGGCACCTT-GTGAGAAATCAT-GAGTGTTTAGATTC
TGGGGGGAGTATGGTCGCAAGTCTGAAACTTAAAGGAATTAACGGAAGGGCACACAATGG
AGTGGAG-CCTGCGGCTTAATTT-GACTCAACTCGGGAAA-ACTTACCAAGCTAAGATAT
AATAAGGATTGACAGA-CTAA-AAGATCTTTCATGATTCTATAAGTGGTGGTGCATGGTC
GTTCTTAG-TTGGTGGAGCAATCTGTCTGGTCAATTCCGATAACGGACGAGATCTCGACC
TGCTAACTAGTAGTACATATTAGCTCGATATGCAAGAAAGGCTTGTTGGAGTAAGTTATG
GAT----CGCAAGG-TTC-----ATAGCCCTTCGCTGGT----CGAGTATTGTGAGTTAA
TATA-GTGCGATACAATAAAA--------CTTCTTAGAGGGACTACCT-GTGGCAAACAG
GGGG-AAGTTCGAGGCAATAACAGGTCTGTGATGCCCTTAGATACCTT-GGGCCGCACGC
GCGCTACAATGTAGTACGCAAAAGGTT-----TTTTG--CC--AAGCCCGG-AAG---GG
TG-AGGTAATCCTAT-AATTTACTACGTAACTGGGATTG-ATCTTTGTAATTTTTGATCA
TCAACGAGGAATTCCTTGTAAGCGCAAATCATTACTTTGTGCTGAATCTGTCCCTGCCCT
TTGTACACACCGCCCGTCGCTCCTACCGATCGAATGATACGGTAAAGCTAACAGATAC--
--AAT----T-----C-----G---CAA-G------------AGTT-GTA-AAAG--TTA
-TT-TAAATCT--C----ATT--GTTT
>AM168047.1_Hagiwaraea_lavandula_B15
TCTAAG-TATAAATTTTTA-TATGATGAAA----CTGCAGAAGGCTCATT--ACAA--CA
---GT----GATAAACTA-CTAGACTTTCGG-GCTTC----GGCCTTTTT-GGATAACCG
CAGTAAATCGGGGCTAATACATACAAT-CG-AGGGG-TGACTGTT-----TAC-G-G-AA
TCTCCGCGATTATT----A-GCT-TTC--AG-CCAACAC--TCTTCGGAGTTTTG--GGT
GAACCCGGATAATATTGCAGATCGAAG--------CTTACGCTTTCGACAAGTCTAATGT
GTCACTGCCCTATCAACTTTCGATGGTACGGTATTGGCCTACCATGGTTGTAACGGGTAA
CGGAGAATTAGGGTTCGATTCCGGAGAGGGCGCCTGAGAAATGGCGACCACTTCTACGGA
AGGCAGCAGGCGCGCAAATT-ACTCAATCCCAATAC-GGGGAAGTAGTGACAAGAAATAT
TAATGCCTATCCC-TT-TCGGGAAG--GTAATTAAAATGGATTTAAACTAAACCCATTTT
TT-AATACAATTGGAGGGCAAGTCTGGTGCCAGCAGCCGCGGTAATTCCAGCTCCAA-TA
GCATATACTAAATTTGTTGCGGTTAAAA-AGC-TCGTAGTTGAAGTTAAAATTATACT-G
GG-TTCA----------AGGTTTTAGCCGACTTTG--TC--------GGTTTAA-----A
ACTTCCAGTGTAGCTTTTA-CAACT---CGTC-CAGGTGTCTTGGTACTTT------GTA
TC--AGGTCTTCTTT-------------GGACATTTCACTGTGAGGAAA-TTGTGGTGCT
TAAAGCA--GG-C---GTT---TTCGCTTGATCTTTTGCAGCATGGTATGATAGAATATG
ACAT---TTTATGT--AGTTGGCT--CGCA------TAAAGTGTAATGATTAATAGGGAT
GGATGGGGGTGTTCATATTGATGGGCGAGAGGTGAAATTCGTTGACCCTATCAAGATGAA
CTTCTGCGAAAGCATTCACCAAATACTTCTCCATTAATCAAGAACGAAAGTTTGGGGATC
AAAGACGATCAGATACCGTCGTAGTCCAAACTATAAACTATGTCGACCAGGGATCAGCTA
AT--AT-TTTTAAAAAAATATAGTTGGCACCTT-GTGAGAAATCAT-GAGTGTTTAGATTC
TGGGGGGAGTATGGCCGCAAGTCTGAAACTTAAAGGAATTGACGGAAGGGCACACAATGG
AGTGGAG-CCTGCGGCTTAATTT-GACTCAACTCGGGAAA-ACTTACCAAGCTAAGATAT
AATAAGGATTGACAGA-CTAA-AAGATCTTTCATGATTCTATAAGTGGTGGTGCATGGTC
GTTCTTAG-TTGGTGGAGCAATCTGTCTGGTCAATTCCGATAACGGACGAGATCTCGACC
TGCTAACTAGTAGTACATATTAGCTCAATATGCAAGAAAGGCTTGTTGGAGTAGGTCATG
GAT----CGCAAGG-TTC-----ATGGCTCTTCGCTGGT----CGAGTATTGTGAGTTAA
TAT--GTGCGATAAAATAAAA--------CTTCTTAGAGGGACTACCT-GTGGCAAACAG
GGGG-AAGTTCGAGGCAATAACAGGTCTGTGGTGCCCTTAGATACCTT-GGGCCGCACGC
GCGCTACAATGTAGTACGCAAAAGGTA-----TTTTG--CC--AAGCCCGG-AAG---GG
TA-AGGTAATCCTAT-AATTTACTACGTAACTGGGCTTG-ATCTTTGTAATTTTTGATCA
TCAACGAGGAATTCCTTGTAAGCGCAAATCATTACTTTGTGCTGAATCTGTCCCTGCCCT
TTGTACACGCCGCCCGTCGCTCCTACCGATCGAATGATACGGTAAAGCTAACAGATAT--
--AA----CT-----C-----G---CAA-G------------AGTT-GTA-AAAG--TTA
-TT-TAAATCT--C----ATT--GTTT
>AM168075.1_Raperostelium_tenue_PR4
TCTAAG-TATAAATTTTTA-TATGATGAAA----CTGCAGACGGCTCATT--ACAA--CA
---GT----AATAAACTA-ATAGACTTTCGG-GTTTTA---TTACCTTTT-GGATAACCG
CAGTAAATCGGGGCTAATACATACAAT-CG-AGGGG-TGACTGTT-----TAC-G-G-AA
TCTCCGCGATTATT----A-GCT-TTCTCAA-CCAATAC--CCTTCGGGGTTTTGATGGT
GAAACCGAATAATATTGCAGATCGAAG--------CTT-CGGCATCGACAAGTCTATTGT

GTTACTGCCCTATCAACTTTCGATGGTACGGTATTGGCCTACCATGGTTGTAACGGGTAA
CGGGGAATTAGGGTTCGATTCCGGAGAGGGCGCCTGAGAAATGGCGACCACTTCTACGGA
AGGCAGCAGGCGCGCAAATT-ACTCAATCCCAATAC-GGGGAAGTAGTGACAAGAAATAT
TAATGCCTATCC--TC-ATAGGAAG--GTAATTAAAATGGGTCTAAACTAAATCCATTTT
CT-AATACAATTGGAGGGCAAGTCTGGTGCCAGCAGCCGCGGTAATTCCAGCTCCAA-TA
GCATATACTAAATTTGTTGCGGTTAAAA-AGC-TCGTAGTTGAAATTAAAGTCATATT-G
GG-TTAA---------TAGGTGTTAGCCG-ATTTTAATG-----T-CGGTTTAA-----T
GCTTCCAGTATGTATTTTT-TAAAT--TGACT-CTTGGGTTCGGTTACTTT------GTA
GT-CGGGCTCGTTA--------------GGGTTCTTCACTGTGAGAAAAATTGTGGTGCT
TAA-GCA--GG-C---GTT---T-CGCTTGATCTTTTGCAGCATGGTATGATAGAACATG
ACAT---TTTGCGC--TATTGGTT---GCG-----TTAAAGTGTAATGATTAATAGGGAT
GGATGGGGGTGTTCATATCGGTGGGCGAGAGGTGAAATTCGTTGACCCTATCAAGATGAA
CTTCTGCGAAAGCATTCACCAAATACTTCCCCATTAATCAAGAACGAAAGTTTGGGGATC
AAAGACGATCAGATACCGTCGTAGTCCAAACTATAAACTATGTCGACCAGGGATCAGCTA
AA--AT-TTCTAAAAAATTTAGTTGGCACCTT-GTGAGAAATCAT-GAGTGTTTAGATTC
TGGGGGGAGTATGGTCGCAAGTCTGAAACTTAAAGGAATTGACGGAAGGGCACACAATGG
AGTGGAG-CCTGCGGCTTAATTT-GACTCAACTCGGGAAA-ACTTACCAAGCTAAGATAT
AATAAGGATTGACAGA-CTAA-AAGATCTTTCATGATTGTATAAGTGGTGGTGCATGGTC
GTTCTTAG-TTGGTGGAGCCATCTGTCTGGTCAATTCCGATAACGGACGAGATCTCGACC
TGCTAACTAGTAGTACCTATTAGCTCAATATGCATGAAAGCTTTGTTGGAGTAAGTTGTA
GGT----CGAAAGG-TTT-----ACAACACTTCGCTGGG----TCGGTAATGTGAATTAA
TA-G-GTACGAT-AA---ACAAA---A--CTTCTTAGAGGGACTACCT-GTGGCAAACAG
GGGG-AAGTTCGAGGCAATAACAGGTCTGTGATGCCCTTAGATACCTT-GGGCCGCACGC
GCGCTACAATGTAGTACGCAAAAAGCT-----AT-----CC--TGGTCCGG-AAG---GA
TT-GGGTAATCATAA--ATTTACTACGTAACTGGGATTG-ATCTTTGTAATTATTGATCA
TCAACGAGGAATTCCTTGTAAGCGCAAATCATTACTTTGTGCTGAATATGTCCCTGCCCT
TTGTACACACCGCCCGTCGCTCCTACCGATCGAATGATACGGTAAAGCCAACAGATTG--
--G-----GT-----C-TGTAG---CAA-T-ACT-T------GATC-CGA-AAAG--TTG
-TT-TAAATCT--C----ATT--GTTT
>AM168094.1_Raperostelium_tenue_PJ6
TCTAAG-TATAAATTTTTA-TATGATGAAA----CTGCAGACGGCTCATT--ACAA--CA
---GT----AATAAACTA-ATAGACTTTCGG-GTTTTA---TTACCTTTT-GGATAACCG
CAGTAAATCGGGGCTAATACATACAAT-CG-AGGGG-TGACTGTT-----TAC-G-G-AA
TCTCCGCGATTATT----A-GCT-TTCTCAA-CCAATAC--CCTTCGGGGTTTTGATGGT
GAAACCGAATAATATTGCAGATCGAAG--------CTT-CGGCATCGACAAGTCTATTGT
GTTACTGCCCTATCAACTTTCGATGGTACGGTATTGGCCTACCATGGTTGTAACGGGTAA
CGGGGAATTAGGGTTCGATTCCGGAGAGGGCGCCTGAGAAATGGCGACCACTTCTACGGA
AGGCAGCAGGCGCGCAAATT-ACTCAATCCCAATAC-GGGGAAGTAGTGACAAGAAATAT
TAATGCCTATCC--TC-ATAGGAAG--GTAATTAAAATGGGTCTAAACTAAATCCATTTT
CT-AATACAATTGGAGGGCAAGTCTGGTGCCAGCAGCCGCGGTAATTCCAGCTCCAA-TA
GCATATACTAAATTTGTTGCGGTTAAAA-AGC-TCGTAGTTGAAATTAAAGTCATATT-G
GG-TTAA---------TAGGTGTTAGCCG-ATTTTAATG-----T-CGGTTTAA-----T
GCTTCCAGTATGTATTTTT-TAAAT--TGACT-CTTGGGTTCGGTTACTTT------GTA
GT-CGGGCTCGTTA--------------GGGTTCTTCACTGTGAGAAAA-TTGTGGTGCT
TAAAGCA--GG-C---GTT---T-CGCTTGATCTTTTGCAGCATGGTATGATAGAACATG
ACAT---TTTGCGC--TATTGGTT---GCG-----TTAAAGTGTAATGATTAATAGGGAT
GGATGGGGGTGTTCATATCGGTGGGCGAGAGGTGAAATTCGTTGACCCTATCAAGATGAA
CTTCTGCGAAAGCATTCACCAAATACTTCCCCATTAATCAAGAACGAAAGTTTGGGGATC
AAAGACGATCAGATACCGTCGTAGTCCAAACTATAAACTATGTCGACCAGGGATCAGCTA
AA--AT-TTCTAAAAAATTTAGTTGGCACCTT-GTGAGAAATCAT-GAGTGTTTAGATTC
TGGGGGGAGTATGGTCGCAAGTCTGAAACTTAAAGGAATTGACGGAAGGGCACACAATGG
AGTGGAG-CCTGCGGCTTAATTT-GACTCAACTCGGGAAA-ACTTACCAAGCTAAGATAT
AATAAGGATTGACAGA-CTAA-AAGATCTTTTATGATTGTATAAGTGGTGGTGCATGGTC
GTTCTTAG-TTGGTGGAGCAATCTGTCTGGTCAATTCCGATAACGGACGAGATCTCGACC
TGCTAACTAGTAGTACCTATTAGCTCAATATGCATGAAAGCTTTGTTGGAGTAAGTTGTA
GGT----CGAAAGG-TTT-----ACAACACTTCGCTGGG----TCGGTAATGTGAATTAA
TA-G-GTACGAT-AA---ACAAA---A--CTTCTTAGAGGGACTACCT-GTGGCAAACAG
GGGG-AAGTTCGAGGCAATAACAGGTCTGTGATGCCCTTAGATACCTT-GGGCCGCACGC
GCGCTACAATGTAGTACGCAAAAAGCT-----AT-----CC--TGGTCCGG-AAG---GA
TT-GGGTAATCATAA--ATTTACTACGTAACTGGGATTG-ATCTTTGTAATTATTGATCA
TCAACGAGGAATTCCTTGTAAGCGCAAATCATTACTTTGTGCTGAATATGTCCCTGCCCT
TTGTACACACCGCCCGTCGCTCCTACCGATCGAATGATACGGTAAAGCCAACAGATTG--
--G-----GT-----C-TGTAG---CAA-T-ACT-T------GATC-CGA-AAAG--TTG
-TT-TAAATCT--C----ATT--GTTT
>JF892721.1_Raperostelium_capillare_37A
TCTAAG-TATAAATTTTTA-TATGATGAAA----CTGCAGACGGCTCATT--ACAA--CA
---GT----AATAAACTA-ATAGACTTTCGG-GTTTAA---TTACCTTTT-GGATAACCG
CAGTAAATCGGGGCTAATACATACAAT-CG-AGGGG-TGACTGTT-----TAC-G-G-AA
TCTCCGCGATTATT----A-GCA-TT--CAA-CCAATAC--CTTTCGAGGTTTTG-TGGT
GAAACCGAATAATATTGCAGATCAAAG--------CTTTCGGCTTTGACAAGTCTATTGT
GTTACTGCCCTATCAACTTTCGATGGTACGGTATTGGCCTACCATGGTTGTAACGGGTAA
CGGGGAATTAGGGTTCGATTCCGGAGAGGGCGCCTGAGAAATGGCGACCACTTCTACGGA
AGGCAGCAGGCGCGCAAATT-ACTCAATCCCAATAC-GGGGAAGTAGTGACAAGAAATAT

TAATGCCTATCC--TT-TTAGGAAG--GTAATTAAAATGGGTCTAAACTAAATCCATTTT
CT-AATACAATTGGAGGGCAAGTCTGGTGCCAGCAGCCGCGGTAATTCCAGCTCCAA-TA
GCATATACTAAATTTGTTGCGGTTAAAA-AGC-TCGTAGTTTAAACAAAAATTATATT-G
GG-CTAA---------TAGGTGTTAGCCGGATTTTTATC-----C-CGGTTTAA-----T
ACTTCCAATATATATTTTT-TAAATATTAGCT-CTTGGATTTGGTTACTTTT-----GTA
ATTCAAGTTCGTTA--------------GAGTCCTTCACTGTGAGAAAA-TTGTGGTGCT
TAAAGCA--GG-C---GTT---T-CGCTTGATCTTTTGCAGCATGGTATGATAGAACATG
ACAT---TTTGTGC--TATTGGTT---GCA-----TTAAAGTGTAATGATTAATAGGGAT
GGATGGGGGTGTTCATATTGGTGGGCGAGAGGTGAAATTCGTTGACCCTATCAAGATGAA
CTTCTGCGAAAGCATTCACCAAATACTTCCCCATTAATCAAGAACGAAAGTTTGGGGATC
AAAGACGATCAGATACCGTCGTAGTCCAAACTATAAACTATGTCGACCAGGGATCAGCTA
AA--AT-TTCTAAAAAATTTAGTTGGCACCTT-GTGAGAAATCAC-GAGTGTTTAGATTC
TGGGGGGAGTATGGTCGCAAGTCTGAAACTTAAAGGAATTGACGGAAGGGCACACAATGG
AGTGGAG-CCTGCGGCTTAATTT-GACTCAACTCGGGAAA-ACTTACCAAGCTAAGATAT
AATAAGGATTGACAGA-CTAA-AAGATCTTTCATGATTGTATAAGTGGTGGTGCATGGTC
GTTCTTAG-TTGGTGGAGCAATCTGTCTGGTCAATTCCGATAACGGACGAGATCTCGACC
TGCTAACTAGTAGTACATATTAGCTCAATATGCATGAAAGATTAGTTGGAGTAAGTTGTA
GGT----CGAAAGG-TTT-----ACAGCACTTCACTGGT----TCGGTAATGTGAATTAA
TATG-GTACGAT-AA---ATAAA---A--CTTCTTAGAGGGACTACCT-GTGGCAAACAG
GGGG-AAGTTCGAGGCAATAACAGGTCTGTGATGCCCTTAGATACCTT-GGGCCGCACGC
GCGCTACAATGTAGTACGCAAAAAGCT-----CT-----CC--TGGTCCGG-AAG---GA
TT-GGGTAATCAAAA-AATTTACTACGTAACTGGGATTG-ATCTTTGTAATTATTGATCA
TCAACGAGGAATTCCTTGTAAGCGCAAATCATTACTTTGTGCTGAATATGTCCCTGCCCT
TTGTACACACCGCCCGTCGCTCCTACCGATCGAATGATACGGTAAAGCCAACAGATTG--
--GAT----------C-TGTAG---CAA-T-ACA--------GAGC-TGA-AAAG--TTG
-TT-TAAATCT--C----ATT--GTT-
>JF892724.1_Raperostelium_filiforme_OH603
TCTAAG-TATAAATTTTTA-TATGATGAAA----CTGCAGACGGCTCAAT--ACAA--CA
---GT----AATAAACTA-ATAGACTTTCGG-GTTTAA---TTACCTTTT-GGATAACCG
CAGTAAATCGGGGCTAATACATACAAT-CG-AGGGC-TGACTGTT-----TAC-G-G-AA
TGTCCGCGATTATT----A-GCA-TT--CAA-CCAATAC--CTTTCGAGGTTTTG-TGGT
GAAACCGAATAATATTGCAGATCAAAG--------CTT-CGGCTTTGACAAGTCTATTGT
GTTACTGCCCTATCAACTTTCGATGGTACGGTATTGGCCTACCATGGTTGTAACGGGTAA
CGGGGAATTAGGGTTCGATTCCGGAGAGGGCGCCTGAGAAATGGCGACCACTTCTACGGA
AGGCAGCAGGCGCGCAAATT-ACTCAATCCCAATAC-GGGGAAGTAGTGACAAGAAATAT
TAATGCCTATCC--TT-TTAGGAAG--GTAATTAAAATGGGTCTAAACTAAATCCATTTT
CT-AATACAATTGGAGGGCAAGTCTGGTGCCAGCAGCCGCGGTAATTCCAGCTCCAA-TA
GCATATACTAAATTTGTTGCGGTTAAAA-AGC-TCGTAGTTTAAACAAAAATTATATT-G
GG-CTAA---------TAGGTGTTAGCCGGATTTTTATC-----C-CGGTTTAA-----T
ACTTCCAATATATATTTTT-TAAATATTAGCT-CTTGGATCTGGTTACTTTT-----GTA
ATTCAGGTTCGTTA--------------GAGTCCTTCACTGTGAGAAAA-TTGTGGTGCT
TAAAGCA--GG-C---GTT---T-CGCTTGATCTTTTGCAGCATGGTATGATAGAACATG
ACAT---TTTGTGC--TATTGGTT---GCA-----TTAAAGTGTAATGATTAATAGGGAT
GGATGGGGGTGTTCATATTGGTGGGCGAGAGGTGAAATTCGTTGACCCTATCAAGATGAA
CTTCTGCGAAAGCATTCACCAAATACTTCCCCATTAATCAAGAACGAAAGTTTGGGGATC
AAAGACGATCAGATACCGTCGTAGTCCAAACTATAAACTATGTCGACCAGGGATCAGCTA
AA--AT-TTCTAAAAAATTTAGTTGGCACCTT-GTGAGAAATCAC-GAGTGTTTAGATTC
TGGGGGGAGTATGGTCGCAAGTCTGAAACTTAAAGGAATTGACGGAAGGGCACACAATGG
AGTGGAG-CCTGCGGCTTAATTT-GACTCAACTCGGGAAA-ACTTACCAAGCTAAGATAT
AATAAGGATTGACAGA-CTAA-AAGATCTTTCATGATTGTATAAGTGGTGGTGCATGGTC
GTTCTTAG-TTGGTGGAGCAATCTGTCTGGTCAATTCCGATAACGGACGAGATCTCGACC
TGCTAACTAGTAGTACATATTAGCTCAATATGCATGAAAGATTAGTTGGAGTAAGTTGTA
GGT----CGAAAGG-TTT-----ACAGCACTTCACTGGT----TCGGTAATGTGAATTAA
TATG-GTACGAT-AA---ATAAA---A--CTTCTTAGAGGGACTACCT-GTGGCAAACAG
GGGG-AAGTTCGAGGCAATAACAGGTCTGTGATGCCCTTAGATACCTT-GGGCCGCACGC
GCGCTACAATGTAGTACGCAAAAAGCT-----CT-----CC--TGGTCCGG-AAG---GA
TT-GGGTAATCAAAA-AATTTACTACGTAACTGGGATTG-ATCTTTGTAATTATTGATCA
TCAACGAGGAATTCCTTGTAAGCGCAAATCATTACTTTGTGCTGAATATGTCCCTGCCCT
TTGTACACACCGCCCGTCGCTCCTACCGATCGAATGATACGGTAAAGCCAACAGATTG--
--G-T----TT----C--GTAG-G-CAA-T-TCA--------GAGC-TGA-AAAG--ATA
-TT-TAAATCT--C----ATT--GTTT
>HQ141491.1_Raperosteliums_sp._TH14B
TCTAAG-TATAAATTTTTA-TATGATGAAA----CTGCAGACGGCTCATT--ACAA--CA
---GT----AATAAACTA-ATAGACTTTCGG-GTTTTA---TTACCTTTT-GGATAACCG
CAGTAAATCGGGGCTAATACATACAAT-CG-AGGGG-TGACTGTT-----TAC-G-G-AA
TCCCCGCGATTATT----A-GCT-TTT-CAA-CCAATACTCCTTT-GGAGTTTTG-TGGT
GAAACCGAATAATATTGCAGATCGAAG--------CTT-AGGCATCGACAAGTCTACTGT
GTTACTGCCCTATCAACTTTCGATGGTACGGTATTGGCCTACCATGGTTGTAACGGGTAA
CGGGGAATTAGGGTTCGATTCCGGAGAGGGCGCCTGAGAAATGGCGACCACTTCTACGGA
AGGCAGCAGGCGCGCAAATT-ACTCAATCCCAATAC-GGGGAAGTAGTGACAAGAAATAT
TAATGCCTATCC--TT-TTAGGAAG--GTAATTAAAATGGGTCTAAACTAAATCCATTTT
CT-AATACAATTGGAGGGCAAGTCTGGTGCCAGCAGCCGCGGTAATTCCAGCTCCAA-TA
GCATATACTAAATTTGTTGCGGTTAAAA-AGC-TCGTAGTTGAAATTAAAATTACATT-G

GG-CTAAA--------TAGGTTTTAGCCGATTTTTCATC--------GGTTTAA-----A
ACTTCCAATGTATATTTTT-TAAATTTTGGCT-CATGAATTTGGTTACTTTT-----GTA
ATTCAAGTTCTTTTT-------------GGGTTCTTCACTGTGAGAAAA-TTGTGGTGCT
TAAAGCA--GG-C---GTT---TTCGCTTGATCTTTTGCAGCATGGGATGATAGAACATG
ACAT---TTTGCGC--TATTGGTT---GCG-----TTAAAGTGTAATGATTAATAGGGAT
GGATGGGGGTGTTCATATTGGTGGGCGAGAGGTGAAATTCGTTGACCCTATCAAGATGAA
CTTCTGCGAAAGCATTCACCAAATACTTCCCCATTAATCAAGAACGAAAGTTTGGGGATC
AAAGACGATCAGATACCGTCGTAGTCCAAACTATAAACTATGTCGACCAGGGATCAGCTA
AA--AT-TTCTAAAAAATTTAGTTGGCACCTT-GTGAGAAATCAC-GAGTGTTTAGATTC
TGGGGGGAGTATGGTCGCAAGTCTGAAACTTAAAGGAATTGACGGAAGGGCACACAATGG
AGTGGAG-CCTGCGGCTTAATTT-GACTCAACTCGGGAAA-ACTTACCAAGCTAAGATAT
AATAAGGATTGACAGA-CTAA-AAGATCTTTCATGATTGTATAAGTGGTGGTGCATGGTC
GTTCTTAG-TTGGTGGAGCAATCTGTCTGGTCAATTCCGATAACGGACGAGATCTCGACC
TGCTAACTAGTAGTACTTATCAGCTCAATATGCACGAAAGCTTTGTTGGAGTAAGTTGTA
GGT----CGAAAGG-TTT-----ACAACACTTCGCTGAG----TCGGTAGTGTGAATTGA
TAA--GTACGCTTAA---ATAAA---A--CTTCTTAGAGGGACTACCT-GTGGCAAACAG
GGGG-AAGTTCGAGGCAATAACAGGTCTGTGATGCCCTTAGATACCTT-GGGCCGCACGC
GCGCTACAATGTAGTACGCAAAAAGCA-----AT-----CC--TGGTCCGG-AAG---GA
CT-GGGTAATCATAA--ATTTACTACGTAACTGGGATTG-ATCTTTGTAATTATTGATCA
TCAACGAGGAATTCCTTGTAAGCGCAAATCATTACTTTGTGCTGAATATGTCCCTGCCCT
TTGTACACACCGCCCGTCGCTCCTACCGATCGAATGATACGGTAAAGCCAACAGATTA--
---AT---GTA----T-T-TGG-G-AAA-CCATT-T------ACGT-TGA-AAAG--TTG
-TT-TAAATCT--C----AT---GTT-
>AM168078.1_Raperostelium_gracile_TNS_C_183
TCTAAG-TATAAATTTTTA-TATGATGAAA----CTGCAGACGGCTCATT--ACAA--CA
---GT----AATAAACTA-ATAGACTTTCGG-GTTTTA---TTACCTTTT-GGATAACCG
CAGTAAATCGGGGCTAATACATACAAT-CG-AGGGG-TGACTGTT-----TAC-G-G-AA
TCCCCGCGATTATT----A-GCT-TTT-CAA-CCAATACTCCTTTTGGAGTTTTG-TGGT
GAAACCGAATAATATTGCAGATCGAAG--------CTT-AGGCATCGACAAGTCTACTGT
GTTACTGCCCTATCAACTTTCGATGGTACGGTATTGGCCTACCATGGTTGTAACGGGTAA
CGGGGAATTAGGGTTCGATTCCGGAGAGGGCGCCTGAGAAATGGCGACCACTTCTACGGA
AGGCAGCAGGCGCGCAAATT-ACTCAATCCCAATAC-GGGGAAGTAGTGACAAGAAATAT
TAATGCCTATCC--TT-TTAGGAAG--GTAATTAAAATGGGTCTAAACTAAATCCATTTT
CT-AATACAATTGGAGGGCAAGTCTGGTGCCAGCAGCCGCGGTAATTCCAGCTCCAA-TA
GCATATACTAAATTTGTTGCGGTTAAAA-AGC-TCGTAGTTGAAATTAAAATTATATT-G
GG-CTAAA--------TAGGTTTTAGCCGATTTTTCATC--------GGTTTAA-----A
ACTTCCAATATATATTTTT-TAAATTTTGGCT-CATGAATTTGGTTACTTTT-----GTA
ATTCAAGTTCTTTTT-------------GGGTTCTTCACTGTGAGAAAA-TTGTGGTGCT
TAAAGCA--GG-C---GTT---TTCGCTTGATCTTTTGCAGCATGGGATGATAGAACATG
ACAT---TTTGCGC--TATTGGTT---GCG-----TTAAAGTGTAATGATTAATAGGGAT
GGATGGGGGTGTTCATATTGGTGGGCGAGAGGTGAAATTCGTTGACCCTATCAAGATGAA
CTTCTGCGAAAGCATTCACCAAATACTTCCCCATTAATCAAGAACGAAAGTTTGGGGATC
AAAGACGATCAGATACCGTCGTAGTCCAAACTATAAACTATGTCGACCAGGGATCAGCTA
AA--AT-TTCTAAAAAATTTAGTTGGCACCTT-GTGAGAAATCAC-GAGTGTTTAGATTC
TGGGGGGAGTATGGTCGCAAGTCTGAAACTTAAAGGAATTGACGGAAGGGCACACAATGG
AGTGGAG-CCTGCGGCTTAATTT-GACTCAACTCGGGAAA-ACTTACCAAGCTAAGATAT
AATAAGGATTGACAGA-CTAA-AAGATCTTTCATGATTGTATAAGTGGTGGTGCATGGTC
GTTCTTAG-TTGGTGGAGCAATCTGTCTGGTCAATTCCGATAACGGACGAGATCTCGACC
TGCTAACTAGTAGTACTTATCAGCTCAATATGCACGAAAGCTTTGTTGGAGTAAGTTGTA
GGT----CGAAAGG-TTT-----ACAACACTTCGCTTGG----TCGGTAGTGTGAATTGA
TAA--GTACGCTTAA---ATAAA---A--CTTCTTAGAGGGACTACCT-GTGGCAAACAG
GGGG-AAGTTCGAGGCAATAACAGGTCTGTGATGCCCTTAGATACCTT-GGGCCGCACGC
GCGCTACAATGTAGTACGCAAAAAGCA-----AT-----CC--TGGTCCGG-AAG---GA
CT-GGGTAATCATAA--ATTTACTACGTAACTGGGATTG-ATCTTTGTAATTATTGATCA
TCAACGAGGAATTCCTTGTAAGCGCAAATCATTACTTTGTGCTGAATATGTCCCTGCCCT
TTGTACACACCGCCCGTCGCTCCTACCGATCGAATGATACGGTAAAGCCAACAGATTA--
---AT---GTA----T-TATGG-G-CAA-CCATTTT------ACGT-TGA-AAAG--TTG
-TT-TAAATCT--C----ATT--GTTT
>JF892719.1_Raperostelium_maeandriforme_OH604
TCTAAG-TATAAATTTTTA-TATGATGAAA----CTGCAGACGGCTCATT--ACAA--CA
---GT----AATAAACTA-ATAGACTTTCGG-GTTTAA---TTACCTTTT-GGATAACCG
CAGTAAATCGGGGCTAATACATACAAT-CG-AGGGG-TGACTGTT-----TAC-G-G-AA
TCTCCGCGATTATT----A-GCA-TT--CAA-CCAATAC--CTTTCGAGGTTTTG-TGGT
GAAACCGAATAATATTGCAGATCGAAG--------CTT-CGGCTTTGACAAGTCTATTGT
GTTACTGCCCTATCAACTTTCGATGGTACGGTATTGGCCTACCATGGTTGTAACGGGTAA
CGGGGAATTAGGGTTCGATTCCGGAGAGGGCGCCTGAGAAATGGCGACCACTTCTACGGA
AGGCAGCAGGCGCGCAAATT-ACTCAATCCCAATAC-GGGGAAGTAGTGACAAGAAATAT
TAATGCCTATCC--TT-TTAGGAAG--GTAATTAAAATGGGTCTAAACTAAATCCATTTT
CT-AATACAATTGGAGGGCAAGTCTGGTGCCAGCAGCCGCGGTAATTCCAGCTCCAA-TA
GCATATACTAAATTTGTTGCGGTTAAAA-AGC-TCGTAGTTTAAACAAAAATTATATT-G
GG-CTAA---------TAGGTGTTAGCCGGATTTTTATAA---GC-CGGTTTAA-----T
ACTTCCAATATATATTTTT-TAAATATTAGCT-CTTGGATTTGGTTACTTTT-----GTA
ATTCAAGTTCGTTA-------------GAGTCCTTCACTGTGAGAAAA-TTGTGGTGCT

TAAAGCA--GG-C---GTT---T-CGCTTGATCTTTTGCAGCATGGTATGATAGAACATG
ACAT---TTTGTGC--TATTGGTT---GCA-----TTAAAGTGTAATGATTAATAGGGAT
GGATGGGGGTGTTCATATTGGTGGGCGAGAGGTGAAATTCGTTGACCCTATCAAGATGAA
CTTCTGCGAAAGCATTCACCAAATACTTCCCCATTAATCAAGAACGAAAGTTTGGGGATC
AAAGACGATCAGATACCGTCGTAGTCCAAACTATAAACTATGTCGACCAGGGATCAGCTA
AA--AT-TTCTAAAAAATTTAGTTGGCACCTT-GTGAGAAATCAC-GAGTGTTTAGATTC
TGGGGGGAGTATGGTCGCAAGTCTGAAACTTAAAGGAATTGACGGAAGGGCACACAATGG
AGTGGAG-CCTGCGGCTTAATTT-GACTCAACTCGGGAAA-ACTTACCAAGCTAAGATAT
AATAAGGATTGACAGA-CTAA-AAGATCTTTCATGATTGTATAAGTGGTGGTGCATGGTC
GTTCTTAG-TTGGTGGAGCAATCTGTCTGGTCAATTCCGATAACGGACGAGATCTCGACC
TGCTAACTAGTAGTACATATTAGCTCAATATGCATGAAAGATTAGTTGGAGTAAGTTGTA
GGT----CGAAAGG-TTT-----ACAGCACTTCACTGGT----TCGGTAATGTGAATTAA
TATG-GTACGAT-AA---ATAAA---A--CTTCTTAGAGGGACTACCT-GTGGCAAACAG
GGGG-AAGTTCGAGGCAATAACAGGTCTGTGATGCCCTTAGATACCTT-GGGCCGCACGC
GCGCTACAATGTAGTACGCAAAAAGCT-----CT-----CC--TGGTCCGG-AAG---GA
TT-GGGTAATCAAAA-AATTTACTACGTAACTGGGATTG-ATCTTTGTAATTATTGATCA
TCAACGAGGAATTCCTTGTAAGCGCAAATCATTACTTTGTGCTGAATATGTCCCTGCCCT
TTGTACACACCGCCCGTCGCTCCTACCGATCGAATGATACGGTAAAGCCAACAGATTG--
--GAT----------C-TGTAG---CAA-T-ACAA-------GGTC-TG--AAAG--TTG
-TT-TAAATCT--C----ATT--GTTT
>AM168051.1_Raperostelium_minutum_71_2
TCTAAG-TATAAATTTTTA-TATGATGAAA----CTGCAGACGGCTCATT--ACAA--CA
---GT----AATAAACTA-ATAGACTTTCGG-GTTTTA---TTACCTTTT-GGATAACCG
CAGTAAATCGGGGCTAATACATACAAT-CG-AGGGG-TGACTGTT-----TAC-G-G-AA
TCTCCGCGATTATT----A-GCT-TT--CAA-CCAATAC--CCTTCGGGGTTTTG-TGGC
GAGACCGAATAATATTGCAGATCAAGG--------CTT-CGGCTTTGACAAGTCTATTGT
GTTACTGCCCTATCAACTTTCGATGGTACGGTATTGGCCTACCATGGTTGTAACGGGTAA
CGGGGAATTAGGGTTCGATTCCGGAGAGGGCGCCTGAGAAATGGCGACCACTTCTACGGA
AGGCAGCAGGCGCGCAAATT-ACTCAATCCCAATAC-GGGGAAGTAGTGACAAGAAATAT
TAATGCCTATCC--T--TCGGGAAG--GTAATTAAAATGGGTCTAAACTAAATCCATTTT
CT-AATACAATTGGAGGGCAAGTCTGGTGCCAGCAGCCGCGGTAATTCCAGCTCCAA-TA
GCATATACTAAATTTGTTGCGGTTAAAA-AGC-TCGTAGTTGAAATTAAAATTACATT-G
GG-TCAA---------G-GGCTTTAGTCGATTTTTCGTC--------GGTTTAA-----A
GCTTCCAATGTATTTTTTT-TAAAA-TTAACT-CATAATCTTGGTTACTTT------GTA
ATTCAGGGTTTTTT-------------GGGTATTTCACTGTGAGAAAA-TTGTGGTGCT
TAAAGCG--GG-C---GTT---T-TGCTTGATCTTTTGCAGCATGGTATGATAGAACATG
ACAT---TTTGTGC--GATTGGTT---GCA-----TTAAAGTGTAATGATTAATAGGGAT
GGATGGGGGTGTTCATATTGGTGGGCGAGAGGTGAAATTCGTTGACCCTATCAAGATGAA
CTTCTGCGAAAGCATTCACCAAATACTTCCCCATTAATCAAGAACGAAAGTTTGGGGATC
AAAGACGATCAGATACCGTCGTAGTCCAAACTATAAACTATGTCGACCAGGGATCAGCTA
AA--AT-TTTACAAAAATTTAGTTGGCACCTT-GTGAGAAATCAC-GAGTGTTTAGATTC
TGGGGGGAGTATGGTCGCAAGTCTGAAACTTAAAGGAATTGACGGAAGGGCACACAATGG
AGTGGAG-CCTGCGGCTTAATTT-GACTCAACTCGGGAAA-ACTTACCAAGCTAAGATAT
AATAAGGATTGACAGA-CTAA-AAGATTTTTCATGATTGTATAAGTGGTGGTGCATGGTC
GTTCTTAG-TTGGTGGAGCAATCTGTCTGGTCAATTCCGATAACGGACGAGATCTCGACC
TGCTAACTAGTAGTACTTATCAGTTCGATATGCATGAAAGGCTTGTTGGAGTAAGTTGTA
GGT----CGAAAGG-TTT-----ACAACACTTCACTGGT----TCGGTAATGTGAGCAGA
TAA--GTACGAAAAA----TAAC---A--CTTCTTAGAGGGACTACCT-GTGGCAAACAG
GGGG-AAGTTCGAGGCAATAACAGGTCTGTGATGCCCTTAGATACCTT-GGGCCGCACGC
GCGCTACAATGTAGTACGCAAAAAGCT-----AA-----C---TGGTCTGG-GAA---GA
TT-GGTTAATCATAA--ATTTACTACGTAACTGGGATTG-ATCTTTGTAATTATTGATCA
TCAACGAGGAATTCCTTGTAAGCGCAAATCATTACTTTGTGCTGAATATGTCCCTGCCCT
TTGTACACACCGCCCGTCGCTCCTACCGATCGAATGATACGGTAAAGCCAACGGATGA--
--GAT----------CATGTAG---CAA-T-ACAAT------GGTC-TTA-AAAG--TTG
-TT-TAAATCT--C----ATT--GTTT
>HQ141493.1_Raperostelium_ohioense_Okla4C
TCTAAG-TATAAATTTTTA-TATGATGAAA----CTGCAGACGGCTCATT--ACAA--CA
---GT----AATAAACTA-ATAGACTTTCGG-GTTTTA---TTACCTTTT-GGATAACCG
CAGTAAATCGGGGCTAATACATACAAT-CG-AGGGG-TGACTGTT-----TAC-G-G-AA
TCTCCGCGATTATT----A-GCT-TT--CAA-CCAATAC--CCTTCG-GGTTTTG-TGGC
GAGACCGAATAATATTGCAGATCAAGG--------CTT-CGGCTTTGACAAGTCTATTGT
GTTACTGCCCTATCAACTTTCGATGGTACGGTATTGGCCTACCATGGTTGTAACGGGTAA
CGGGGAATTAGGGTTCGATTCCGGAGAGGGCGCCTGAGAAATGGCGACCACTTCTACGGA
AGGCAGCAGGCGCGCAAATT-ACTCAATCCCAATAC-GGGGAAGTAGTGACAAGAAATAT
TAATGCCTATCC--T--TCGGGAAG--GTAATTAAAATGGGTCTAAACTAAATCCATTTT
CT-AATACAATTGGAGGGCAAGTCTGGTGCCAGCAGCCGCGGTAATTCCAGCTCCAA-TA
GCATATACTAAATTTGTTGCGGTTAAAA-AGC-TCGTAGTTGAAATTAAAATTACATT-G
GG-TCAA---------G-GGCTTTAGTCGATTTTTCGTC--------GGTTTAA-----A
GCTTCCAATGTATTTTTTT-TAAAA-TTAACT-CATAATCTTGGTTACTTT------GTA
ATTCAGGGTTTTTT-------------GGGTATTTCACTGTGAGAAAA-TTGTGGTGCT
TAAAGCG--GG-C---GTT---T-TGCTTGATCTTTTGCAGCATGGTATGATAGAACATG
ACAT---TTTGTGC--GATTGGTT---GCA-----TTAAAGTGTAATGATTAATAGGGAT
GGATGGGGGTGTTCATATTGGTGGGCGAGAGGTGAAATTCGTTGACCCTATCAAGATGAA

CTTCTGCGAAAGCATTCACCAAATACTTCCCCATTAATCAAGAACGAAAGTTTGGGGATC
AAAGACGATCAGATACCGTCGTAGTCCAAACTATAAACTATGTCGACCAGGGATCAGCTA
AA--AT-TTTACAAAAATTTAGTTGGCACCTT-GTGAGAAATCAC-GAGTGTTTAGATTC
TGGGGGGAGTATGGTCGCAAGTCTGAAACTTAAAGGAATTGACGGAAGGGCACACAATGG
AGTGGAG-CCTGCGGCTTAATTT-GACTCAACTCGGGAAA-ACTTACCAAGCTAAGATAT
AATAAGGATTGACAGA-CTAA-AAGATTTTTCATGATTGTATAAGTGGTGGTGCATGGTC
GTTCTAAG-TTGGTGGAGCAATCTGTCTGGTCAATTCCGATAACGGACGAGATCTCGACC
TGCTAACTAGTAGTACTTATCAGTTCGATATGCATGAAAGGCTTGTTGGAGTAAGTTGTA
GGT----CGAAAGG-TTT-----ACAACACTTCACTGGT----TCGGTAATGTGAGCAGA
TAA--GTACGAAAAA----TAAC---A--CTTCTTAGAGGGACTACCT-GTGGCAAACAG
GGGG-AAGTTCGAGGCAATAACAGGTCTGTGATGCCCTTAGATACCTT-GGGCCGCACGC
GCGCTACAATGTAGTACGCAAAAAGCT-----AA-----C---TGGTCTGG-GAA---GA
TT-GGTTAATCATAA--ATTTACTACGTAACTGGGATTG-ATCTTTGTAATTATTGATCA
TCAACGAGGAATTCCTTGTAAGCGCAAATCATTACTTTGTGCTGAATATGTCCCTGCCCT
TTGTACACACCGCCCGTCGCTCCTACCGATCGAATGATACGGTAAAGCCAACGGATGA--
--GAT----------CATGTAG---CAA-T-ACAAT------GGTC-TTA-AAAG--TTG
-TT-TAAATCT--C----ATG--TTTT
>AM168069.1_Raperostelium_potamoides_FP1A
TCTAAG-TATAAATTTTTA-TATGATGAAA----CTGCAGACGGCTCATT--ACAA--CA
---GT----AATAAACTA-ATAGACTTTCAG-GTTTTA---TTACCTTTT-GGATAACCG
CAGTAAATCGGGGCTAATACATACAAT-TG-AGGGC-TGACTGTT-----TAC-G-G-GA
TGTCCGCGATTATT----A-GCA-TT--CAA-CCAATAC--CTCTCGGGGTTTTG-TGGT
GAAACCGAATAATATTGCAGATCGAGG--------CTT-CGGCTTCGACAAGTCTATTGT
GTTACTGCCCTATCAACTTTCGATGGTACGGTATTGGCCTACCATGGTTGTAACGGGTAA
CGGGGAATTAGGGTTCGATTCCGGAGAGGGCGCCTGAGAAATGGCGACCACTTCTACGGA
AGGCAGCAGGCGCGCAAATT-ACTCAATCCCAATAC-GGGGAAGTAGTGACAAGAAATAT
TAATGCCTATCC--TT-TTAGGAAG--GTAATTAAAATGGGTCTAAACTAAATCCATTTT
CT-AATACAATTGGAGGGCAAGTCTGGTGCCAGCAGCCGCGGTAATTCCAGCTCCAA-TA
GCATATACTAAATTTGTTGCGGTTAAAA-AGC-TCGTAGTTGAAATTAAAGCTATATT-G
GG-TCAA---------G-GGTTCTAGCCGGTTTTACACC--------GGTTTAGT----A
ACTTCCAGTATATGTTTTT-TAAAT-TTTTCC-CATAAGTTTGGTTACTTTT-----GTA
ATTTCAAACGGTTC--------------GGGTACTTCACTGTGAGAAAA-TTGTGGTGCT
TAAAGCG--AG-C---GTT---T-TGCTTGATCTTTTGCAGCCTGGTATGATAGAACATG
ACAT---TTTACGC--TATTGGTT---GCG-----TTAGAGTGTAATGATTAATAGGGAT
GGATGGGGGTGTTCATATTGGTGGGCGAGAGGTGAAATTCGTTGACCCTATCAAGATGAA
CTTCTGCGAAAGCATTCACCAAATACTTCCCCATTAATCAAGAACGAAAGTTTGGGGATC
AAAGACGATCAGATACCGTCGTAGTCCAAACTATAAACTATGTCGACCAAGGATCAGCTA
AA--AT-TTTACAAAAATTTAGTTGGCACTTT-GTGAGAAATCAT-GAGTGTTTAGATTC
TGGGGGGAGTATGGTCGCAAGTCTGAAACTTAAAGGAATTGACGGAAGGGCACACAATGG
AGTGGAG-CCTGCGGCTTAATTT-GACTCAACTCGGGAAA-ACTTACCAAGCTAAGATAT
AATAAGGATTGACAGA-CTAA-AAGATCTTTCATGATTGTATAAGTGGTGGTGCATGGTC
GTTCTTAG-TTGGTGGAGCAATCTGTCTGGTCAATTCCGATAACGGACGAGATCTCGACC
TGCTAACTAGTAGTACATATTAGCTCAATATGCGTGAAAGTTTCGGCGGAGTAAGTTGTA
GGT----CGAAAGG-TTT-----ATGACACTTCACGGGA----TCGGTAACGTGAATTAA
TATT-GTACGATAAA--AATAAA---A--CTTCTTAGAGGGACTACCT-GTGGCAAACAG
GGGG-AAGTTCGAGGCAATAACAGGTCTGTGATGCCCTTAGATACCTT-GGGCCGCACGC
GCGCTACAATGTAGTACGCAAAAAGCT-----TT-----CC--TGGTCCGG-GAG---GA
CT-GGGTAATCATTATAATTTACTACGTAACTGGGATTG-ATCTTTGTAATTATTGATCA
TCAACGAGGAATTCCTTGTAAGCGCAAATCATTACTTTGTGCTGAATATGTCCCTGCCCT
TTGTACACACCGCCCGTCGCTCCTACCGATCGAATGATACGGTAAAGCCAACAGATGA--
---A-----TT----TG-GTAG---TAA-T-AC--T------AAGT-TTA-AAAG--TTG
-TT-TAAATCT--C----ATT--GTTT
>EU672875.1_Raperostelium_sp._MR_2008
TCTA-G-TATAAATTTTTA-TATGATGAAA----CTGCAGACGGCTCATT--ACAA--CA
---GT----AATAAACTA-ATAGACTTTCAG-GTTTTA---TTACCTTTT-GGATAACCG
CAGTAAATCGGGGCTAATACATACAAT-TG-AGGGC-TGACTGTT-----TAC-G-G-AA
TGTCCGCGATTATT----A-GCA-TT--CAA-CCAATAT--CCTCCGGGATTTTG-TGGT
GAAACCGAATAATATTGCAGATCGGAG--------CCT-TGGCTCCGACAAGTCTATTGT
GTTACTGCCCTATCAACTTTCGATGGTACGGTATTGGCCTACCATGGTTGTAACGGGTAA
CGGGGAATTAGGGTTCGATTCCGGAGAGGGCGCCTGAGAAATGGCGACCACTTCTACGGA
AGGCAGCAGGCGCGCAAATT-ACTCAATCCCAATAC-GGGGAAGTAGTGACAAGAAATAT
TAATGCCTATCC--TT-TTAGGAAG--GTAATTAAAATGGGTCTAAACTAAATCCATTTT
CT-AATACAATTGGAGGGCAAGTCTGGTGCCAGCAGCCGCGGTAATTCCAGCTCCAA-TA
GCATATACTAAATTTGTTGCGGTTAAAA-AGC-TCGTAGTTGAAATTAAAGCCATATT-G
GG-TTAA---------T-GGTCTTAGCCGATTTTACGTC--------GGCCTAG-----G
ACTTCCAGTATGTGTTTTT-TAAAT-TTATCC-TATAAGTTTGGTTACTTTT-----GTA
ATT-CAGACGATCT--------------AGGTGTTTCACTGTGAGAAAA-TTGTGGTGCT
TAAAGCG--AG-C---GTT---T-TGCTTGATCTTTTGCAGCATGGTATGATAGAACATG
ACAT---TTTGCGC--TATTGGTT---GCG-----TTAAAGTGTAATGATTAATAGGGAT
GGATGGGGGTGTTCATATTGGTGGGCGAGAGGTGAAATTCGTTGACCCTATCAAGATGAA
CTTCTGCGAAAGCATTCACCAAATACTTCCCCATTAATCAAGAACGAAAGTTTGGGGATC
AAAGACGATCAGATACCGTCGTAGTCCAAACTATAAACTATGTCGACCAAGGATCAGCTA
AA--AT-TTTACAAAAATTTAGTTGGCACTTT-GTGAGAAATCAT-GAGTGTTTAGATTC

TGGGGGGAGTATGGTCGCAAGTCTGAAACTTAAAGGAATTGACGGAAGGGCACACAATGG
AGTGGAG-CCTGCGGCTTAATTT-GACTCAACTCGGGAAA-ACTTACCAAGCTAAGATAT
AATAAGGATTGACAGA-CTAA-AAGATCTTTCATGATTGTATAAGTGGTGGTGCATGGTC
GTTCTTAG-TTGGTGGAGCAATCTGTCTGGTCAATTCCGATAACGGACGAGATCTCGACC
TGCTAACTAGTAGTACATATTAGCTCAATATGCGTGAAAGCTTTGTTGGAGTAAGTTGTA
GGT----CGAAAGG-TTT-----ATGACACTTCGCTGGG----TCGGTAACGTGAATTAA
TAT--GTACGATAAA--AACAAA---A--CTTCTTAGAGGGACTACCT-GTGGCAAACAG
GGGG-AAGTTCGAGGCAATAACAGGTCTGTGATGCCCTTAGATACCTT-GGGCCGCACGC
GCGCTACAATGTAGTACGCAAAAAGCT-----AT-----CC--TGGTCCGG-GAG---GA
CT-GGGTAATCATTATAATTTACTACGTAACTGGGATTG-ATCTTTGTAATTATTGATCA
TCAACGAGGAATTCCTTGTAAGCGCAAATCATTACTTTGTGCTGAATATGTCCCTGCCCT
TTGTACACACCGCCCGTCGCTCCTACCGATCGAATGATACGGTAAAGCCAACAGATGA--
---A-----TT----TG-GTAG---CAA-T-AC--T------AAGT-TTA-AAAG--TTG
-TT-TAAATCT--C----AT---GTT-
>HQ141495.1_Raperostelium_ibericum_214rjb
TCTA-G-TATAAATTTTTA-TATGATGAAA----CTGCAGACGGCTCATT--ACAA--CA
---GT----AATAAACTA-ATAGACTTTCAG-GTTTTA---TTACCTTTT-GGATAACCG
CAGTAAATCGGGGCTAATACATACAAT-TG-AGGGC-TGACTGTT-----TAC-G-G-AA
TGTCCGCGATTATT----A-GCA-TT--CAA-CCAATAT--CCTCCGGGATTTTG-TGGT
GAAACCGAATAATATTGCAGATCGGAG--------CCT-TGGCTCCGACAAGTCTATTGT
GTTACTGCCCTATCAACTTTCGATGGTACGGTATTGGCCTACCATGGTTGTAACGGGTAA
CGGGGAATTAGGGTTCGATTCCGGAGAGGGCGCCTGAGAAATGGCGACCACTTCTACGGA
AGGCAGCAGGCGCGCAAATT-ACTCAATCCCAATAC-GGGGAAGTAGTGACAAGAAATAT
TAATGCCTATCC--TT-TTAGGAAG--GTAATTAAAATGGGTCTAAACTAAATCCATTTT
CT-AATACAATTGGAGGGCAAGTCTGGTGCCAGCAGCCGCGGTAATTCCAGCTCCAA-TA
GCATATACTAAATTTGTTGCGGTTAAAA-AGC-TCGTAGTTGAAATTAAAGCCATATT-G
GG-TTAA---------T-GGTCTTAGCCGATTTTACGTC--------GGCCTAG-----G
ACTTCCAGTATGTGTTTTT-TAAAT-TTATCC-TATAAGTTTGGTTACTTTT-----GTA
ATT-CAGACGATCT--------------AGGTGTTTCACTGTGAGAAAA-TTGTGGTGCT
TAAAGCG--AG-C---GTT---T-TGCTTGATCTTTTGCAGCATGGTATGATAGAACATG
ACAT---TTTGCGC--TATTGGTT---GCG-----TTAAAGTGTAATGATTAATAGGGAT
GGATGGGGGTGTTCATATTGGTGGGCGAGAGGTGAAATTCGTTGACCCTATCAAGATGAA
CTTCTGCGAAAGCATTCACCAAATACTTCCCCATTAATCAAGAACGAAAGTTTGGGGATC
AAAGACGATCAGATACCGTCGTAGTCCAAACTATAAACTATGTCGACCAAGGATCAGCTA
AA--AT-TTTACAAAAATTTAGTTGGCACTTT-GTGAGAAATCAT-GAGTGTTTAGATTC
TGGGGGGAGTATGGTCGCAAGTCTGAAACTTAAAGGAATTGACGGAAGGGCACACAATGG
AGTGGAG-CCTGCGGCTTAATTT-GACTCAACTCGGGAAA-ACTTACCAAGCTAAGATAT
AATAAGGATTGACAGA-CTAA-AAGATCTTTCATGATTGTATAAGTGGTGGTGCATGGTC
GTTCTTAG-TTGGTGGAGCAATCTGTCTGGTCAATTCCGATAACGGACGAGATCTCGACC
TGCTAACTAGTAGTACATATTAGCTCAATATGCGTGAAAGCTTTGTTGGAGTAAGTTGTA
GGT----CGAAAGG-TTT-----ATGACACTTCGCTGGG----TCGGTAACGTGAATTAA
TAT--GTACGATAAA--AACAAA---A--CTTCTTAGAGGGACTACCT-GTGGCAAACAG
GGGG-AAGTTCGAGGCAATAACAGGTCTGTGATGCCCTTAGATACCTT-GGGCCGCACGC
GCGCTACAATGTAGTACGCAAAAAGCT-----AT-----CC--TGGTCCGG-GAG---GA
CT-GGGTAATCATTATAATTTACTACGTAACTGGGATTG-ATCTTTGTAATTATTGATCA
TCAACGAGGAATTCCTTGTAAGCGCAAATCATTACTTTGTGCTGAATATGTCCCTGCCCT
TTGTACACACCGCCCGTCGCTCCTACCGATCGAATGATACGGTAAAGCCAACAGATGA--
---A-----TT----TG-GTAG---CAA-T-AC--T------AAGT-TTA-AAAG--TTG
-TT-TAAATCT--C----AT---GTT-
>MN338957.1_Raperostelium_stabile_M12A
TCTAAG-TATAAATTTTTA-TATGATGAAA----CTGCAGACGGCTCATT--ACAA--CA
---GT----AATAAACTA-ATAGACTTTCGG-GTTTCA---TTACCTTTT-GGATAACCG
CAGTAAATCGGGGCTAATACATACAAT-CG-AGGGC-TGACTGTT-----TAC-G-G-AA
TGTCCGCGATTATT----A-GTC-TT---AA-CCAGTAC--CCTTCGGGGTTTTGATGGT
GAAACCGAATAATATTGCAGATCGAAG--------CTT-ACGCTTTGACAAGTCTATTGT
GTTACTGCCCTATCAACTTTCGATGGTACGGTATTGGCCTACCATGGTTGTAACGGGTAA
CGGGGAATTAGGGTTCGATTCCGGAGAGGGCGCCTGAGAAATGGCGACCACTTCTACGGA
AGGCAGCAGGCGCGCAAATT-ACTCAATCCCAATAC-GGGGAAGTAGTGACAAGAAATAT
TAATGCCTATCC--TT-TTAGGAAG--GTAATTAAAATGGGTCTAAACTAAATCCATTTT
CT-AATACAATTGGAGGGCAAGTCTGGTGCCAGCAGCCGCGGTAATTCCAGCTCCAA-TA
GCATATACTAAATTTGTTGCGGTTAAAA-AGC-TCGTAGTTGAAGTTAAAGCCTTATT-G
GG-TTAA---------TAGGTTTTAGCCGATTTA--TTC--------GGTTTAA-----A
GCTTCCAATATGTGTTTTT-TAAAT--TAACT-CATGGATTAGGTTACTTTT-----GTA
ATTCTAGTTCTTTT--------------GGGTGTTTCACTGTGAGAAAA-TTGTGGTGCT
TAAAGCG--GG-C---GTT---T-TGCCTGATCTTTTGCAGCATGGTATGATAGAACATG
ACAT---TTTGCGC--TATTGGTT---GCG-----TTAAAGTGTAATGATTAATAGGGAT
GGATGGGGGTGTTCATATTGGTGGGCGAGAGGTGAAATTCGTTGACCCTATCAAGATGAA
CTTCTGCGAAAGCATTCACCAAATACTTCCCCATTAATCAAGAACGAAAGTTTGGGGATC
AAAGACGATCAGATACCGTCGTAGTCCAAACTATAAACTATGTCGACCAAGGATCAGCTA
AA--AT-TTTACAAAAATTTAGTTGGCACTTT-GTGAGAAATCAT-GAGTGTTTAGATTC
TGGGGGGAGTATGGTCGCAAGTCTGAAACTTAAAGGAATTGACGGAAGGGCACACAATGG
AGTGGAG-CCTGCGGCTTAATTT-GACTCAACTCGGGAAA-ACTTACCAAGCTAAGATAT
AATAAGGATTGACAGA-CTAA-AAGATTTTTCATGATTGTATAAGTGGTGGTGCATGGTC

GTTCTTAG-TTGGAGGAGCAATCTGTCTGGTCAATTCCGATAACGGACGAGATCTCGACC
TGCTAACTAGTAGTATATATCGATTCGATATGCATGAAAGCCTTGTCGGAGTAAGTTGTA
GGTT---CGCAAGGGTCT-----ATGGCACTTCGCTGGG----TCGGTAATGTGAGTTGA
TAG--GTACGAAAAATTAAAAAA---A--CTTCTTAGAGGGACTACCT-GTGGCAAACAG
GGGG-AAGTTCGAGGCAATAACAGGTCTGTGATGCCCTTAGATACCTT-GGGCCGCACGC
GCGCTACAATGTAGTACGCAAAAAGCA-----AT-----CC--TGGTCCGG-GAG---GA
CT-GGGTAATCATAA--ATTTACTACGTAACTGGGATTG-ATCTTTGTAATTTTTGATCA
TCAACGAGGAATTCCTTGTAAGCGCAAATCATTACTTTGTGCTGAATATGTCCCTGCCCT
TTGTACACACCGCCCGTCGCTCCTACCGATCGAATGATACGGTAAAGCCAACCGATAG--
--------GC-----CA-GTAG---CAA-T-ACA-T------GGCT-TTA-AAAG--TTG
-TT-TAAATCT--C----ATT--GTTT
>AM168029.1_Raperostelium_australe_NZ80B
TCTAAG-TATAAATTTTTA-TATGATGAAA----CTGCAGACGGCTCATT--ACAA--CA
---GT----AATAAACTA-ATAGACTTTCGG-GTTTCA---TTACCTTTT-GGATAACCG
CAGTAAATCGGGGCTAATACATACAAT-CG-AGGGC-TGACTGTT-----TAC-G-G-AA
TGTCCGCGATTATT----A-GCA-TTT--AA-CCAATAC--CCTTCGGGGTTTTG-TGGT
GAAACCGAATAATATTGCAGATCGAAG--------CTT-CGGCTTCGACAAGTCTATTGT
GTTACTGCCCTATCAACTTTCGATGGTACGGTATTGGCCTACCATGGTTGTAACGGGTAA
CGGGGAATTAGGGTTCGATTCCGGAGAGGGCGCCTGAGAAATGGCGACCACTTCTACGGA
AGGCAGCAGGCGCGCAAATT-ACTCAATCCCAATAC-GGGGAAGTAGTGACAAGAAATAT
TAATGCCTATCC--TT-TTAGGAAG--GTAATTAAAATGGGTCTAAACTAAATCCATTTT
CT-AATACAATTGGAGGGCAAGTCTGGTGCCAGCAGCCGCGGTAATTCCAGCTCCAA-TA
GCATATACTAAATTTGTTGCGGTTAAAA-AGC-TCGTAGTTGAAGTAAAAACCTTATT-G
GG-TTAA---------TAGGTCTTAGTCGATTTT--ATC--------GGTTTAA-----G
GCTTCCAATACGCGTTTTT-TAAAC--TAGCT-CATGGATTAGGTTACTTTT-----GTA
ATTCTGGTTCTTTT-------------GGGTGTTTCACTGTGAGAAAA-TTGTGGTGCT
TAAAGCG--GG-C---GTT---T-TGCCTGATCTTTTGCAGCATGGTATGATAGAACATG
ACAT---TTTGCGC--TATTGGTT---GCG-----TTAAAGTGTAATGATTAATAGGGAT
GGATGGGGGTGTTCATATTGGTGGGCGAGAGGTGAAATTCGTTGACCCTATCAAGATGAA
CTTCTGCGAAAGCATTCACCAAATACTTCCCCATTAATCAAGAACGAAAGTTTGGGGATC
AAAGACGATCAGATACCGTCGTAGTCCAAACTATAAACTATGTCGACCAAGGATCAGCTA
AA--AT-TTTACAAAAATTTAGTTGGCACTTT-GTGAGAAATCAT-GAGTGTTTAGATTC
TGGGGGGAGTATGGTCGCAAGTCTGAAACTTAAAGGAATTGACGGAAGGGCACACAATGG
AGTGGAG-CCTGCGGCTTAATTT-GACTCAACTCGGGAAA-ACTTACCAGGCTAAGATAT
AATAAGGATTGACAGA-CTAA-AAGATCTTTCATGATTGTATAAGTGGTGGTGCATGGTC
GTTCTTAG-TTGGTGGAGCAATCTGTCTGGTCAATTCCGATAACGGACGAGATCTCGACC
TGCTAACTAGTAGTATATATCGATTCGATATGCATGAAAGCTTTGTTGGAGTAAGTTGTA
GGTT---CGCAAGGGTCT-----ATGACACTTCACTGGG----TCGGTAATGTGGGTTGA
TAA--ATACGAAAAATTAAAAAA---A--CTTCTTAGAGGGACTACCT-GTGGCAAACAG
GGGG-AAGTTCGAGGCAATAACAGGTCTGTGATGCCCTTAGATACCTT-GGGCCGCACGC
GCGCTACAATGTAGTACGCAAAAAGCA-----AT-----CC--TGGTCCGG-GAG---GA
CT-GGGTAATCATAA--ATTTACTACGTAACTGGGATTG-ATCTTTGTAATTTTTGATCA
TTAACGAGGAATTCCTTGTAAGCGCAAATCATTACTTTGTGCTGAATATGTCCCTGCCCT
TTGTACACACCGCCCGTCGCTCCTACCGATCGAATGATACGGTAAAGCCAACGGATGG--
---A----GT-----CA-GTAG---CAA-T-ACA-T------GACT-CTA-AAAG--TTG
-TT-TAAATCT--C----ATT--GTTT
>AM168052.1_Raperostelium_monochasioides_HAG653
TCTAAG-TATAAATTTTTA-TATGATGAAA----CTGCAGACGGCTCATT--ACAA--CA
---GT----AATAAACTA-ATAGACTTTCGG-GTTTCA---TTACCTTTT-GGATAACCG
CAGTAAATCGGGGCTAATACATACAAT-CG-AGGGC-TGACTGTT-----TAC-G-G-AA
TGTCCGCGATTATT----A-GCA-TT--CAA-CCAATAC--CTTTCGGGGTTTTG-TGGT
GAAACCGAATAATATTGCAGATCGAAG--------CTT-CGGCTTCGACAAGTCTATTGT
GTTACTGCCCTATCAACTTTCGATGGTACGGTATTGGCCTACCATGGTTGTAACGGGTAA
CGGGGAATTAGGGTTCGATTCCGGAGAGGGCGCCTGAGAAATGGCGACCACTTCTACGGA
AGGCAGCAGGCGCGCAAATT-ACTCAATCCCAATAC-GGGGAAGTAGTGACAAGAAATAT
TAATGCCTATCC--TT-TTAGGAAG--GTAATTAAAATGGGTCTAAACTAAATCCATTTT
CT-AATACAATTGGAGGGCAAGTCTGGTGCCAGCAGCCGCGGTAATTCCAGCTCCAA-TA
GCATATACTAAATTTGTTGCGGTTAAAA-AGC-TCGTAGTTGAAATAAAAGCCTTATT-G
GG-TTAA---------TAGGTCTTAGTCGATTTTT-ATC--------GGTTTAA-----G
ACTTCCAATATGTGTTTTT-TAAAT--TAACT-CATGGATTAGGTTACTTTT-----GTA
ATTCTAGTTCGTTT-------------GGGTGCTTCACTGTGAGAAAA-TTGTGGTGCT
TAAAGCA--GG-C---GTT---T-TGCCTGATCTTTTGCAGCATGGTATGATAGAACATG
ACAT---TTTGCGC--TATTGGTT---GCG-----TTAAAGTGTAATGATTAATAGGGAT
GGATGGGGGTGTTCATATTGGTGGGCGAGAGGTGAAATTCGTTGACCCTATCAAGATGAA
CTTCTGCGAAAGCATTCACCAAATACTTCCCCATTAATCAAGAACGAAAGTTTGGGGATC
AAAGACGATCAGATACCGTCGTAGTCCAAACTATAAACTATGTCGACCAAGGATCAGCTA
AA--AT-TTTACAAAAATTTAGTTGGCACTTT-GTGAGAAATCAT-GAGTGTTTAGATTC
TGGGGGGAGTATGGTCGCAAGTCTGAAACTTAAAGGAATTGACGGAAGGGCACACAATGG
AGTGGAG-CCTGCGGCTTAATTT-GACTCAACTCGGGAAA-ACTTACCAAGCTAAGATAT
AATAAGGATTGACAGA-CTAA-AAGATCTTTCATGATTGTATAAGTGGTGGTGCATGGTC
GTTCTTAG-TTGGTGGAGCAATCTGTCTGGTCAATTCCGATAACGGACGAGATCTCGACC
TGCTAACTAGTAGTATATATCGATTCGATATGCATGAAAGCTTAGTCGGAGTAAGTTGTA
GGTT---CGCAAGGGTCT-----ATGGCACTTCGCTGGG----TCGGTAATGTGAGTTGA

TAA--ATACGAAAAATTAAAAAA---A--CTTCTTAGAGGGACTACCT-GTGGCAAACAG
GGGG-AAGTTCGAGGCAATAACAGGTCTGTGATGCCCTTAGATACCTT-GGGCCGCACGC
GCGCTACAATGTAGTACGCAAAAAGCA-----TT-----CC--TGGTCCGG-GAG---GA
CT-GGGTAATCATAA--ATTTACTACGTAACTGGGATTG-ATCTTTGTAATTTTTGATCA
TCAACGAGGAATTCCTTGTAAGCGCAAATCATTACTTTGTGCTGAATATGTCCCTGCCCT
TTGTACACACCGCCCGTCGCTCCTACCGATCGAATGATACGGTAAAGCCAACGGACGG--
---A----GT-----CA-GTAG---CAA-T-ACA-T------GACT-CTA-AAAG--TTG
-TT-TAAATCT--C----GTT--GTTT
>HQ141492.1_Raperostelium_sp._TH8C_TH8C
TCTAAG-TATAAATTTTTA-TATGATGAAA----CTGCAGACGGCTCATT--ACAA--CA
---GT----AATAAACTA-ATAGACTTTCGG-GTTTCA---TTACCTTTT-GGATAACCG
CAGTAAATCGGGGCTAATACATACAAT-CG-AGGGC-TGACTGTT-----TAC-G-G-AA
TGTCCGCGATTATT----A-GCA-TT--CAA-CCAATAC--CTCTCGGGGTTTTG-TGGT
GAAACCGAATAATATTGCAGATCGAAG--------CTT-CGGCTTCGACAAGTCTATTGT
GTTACTGCCCTATCAACTTTCGATGGTACGGTATTGGCCTACCATGGTTGTAACGGGTAA
CGGGGAATTAGGGTTCGATTCCGGAGAGGGCGCCTGAGAAATGGCGACCACTTCTACGGA
AGGCAGCAGGCGCGCAAATT-ACTCAATCCCAATAC-GGGGAAGTAGTGACAAGAAATAT
TAATGCCTATCC--TT-TTAGGAAG--GTAATTAAAATGGGTCTAAACTAAATCCATTTT
CT-AATACAATTGGAGGGCAAGTCTGGTGCCAGCAGCCGCGGTAATTCCAGCTCCAA-TA
GCATATACTAAATTTGTTGCGGTTAAAA-AGC-TCGTAGTTGAAATAAAAGCCTTATT-G
GG-TTAA---------AAGGTCTTAGTCGATTCT--ATC--------GGTTTAA-----G
GCTTCCAATATGTGTTTTT-TAAAC--TAGCT-CATGGATTAGGTTACTTTT-----GTA
ATTCTAGTTCTTTT--------------GGGTGTTTCACTGTGAGAAAA-TTGTGGTGCT
TAAAGCA--GG-C---GTT---T-TGCCTGATCTTTTGCAGCATGGTATGATAGAACATG
ACAT---TTTGCGC--TATTGGTT---GCG-----TTAAAGTGTAATGATTAATAGGGAT
GGATGGGGGTGTTCATATTGGTGGGCGAGAGGTGAAATTCGTTGACCCTATCAAGATGAA
CTTCTGCGAAAGCATTCACCAAATACTTCCCCATTAATCAAGAACGAAAGTTTGGGGATC
AAAGACGATCAGATACCGTCGTAGTCCAAACTATAAACTATGTCGACCAAGGATCAGCTA
AA--AT-TTTACAAAAATTTAGTTGGCACTTT-GTGAGAAATCAT-GAGTGTTTAGATTC
TGGGGGGAGTATGGTCGCAAGTCTGAAACTTAAAGGAATTGACGGAAGGGCACACAATGG
AGTGGAG-CCTGCGGCTTAATTT-GACTCAACTCGGGAAA-ACTTACCAAGCTAAGATAT
AATAAGGATTGACAGA-CTAA-AAGATCTTTCATGATTGTATAAGTGGTGGTGCATGGTC
GTTCTTAG-TTGGTGGAGCAATCTGTCTGGTCAATTCCGATAACGGACGAGATCTCGACC
TGCTAACTAGTAGTATATATCGATTCGATATGCATGAAAGCCTTGTCGGAGTAAGTTGTA
GGTT---CGCAAGGGTCT-----ATGACACTTCGCTGGG----TCGGTAATGTGAGTTGA
TAA--ATACGAAAAATTAAAAAA---A--CTTCTTAGAGGGACTACCT-GTGGCAAACAG
GGGG-AAGTTCGAGGCAATAACAGGTCTGTGATGCCCTTAGATACCTT-GGGCCGCACGC
GCGCTACAATGTAGTACGCAAAAAGCA-----AT-----CC--TGGTCCGG-GAG---GA
CT-GGGTAATCATAA--ATTTACTACGTAACTGGGATTG-ATCTTTGTAATTTTTGATCA
TCAACGAGGAATTCCTTGTAAGCGCAAATCATTACTTTGTGCTGAATATGTCCCTGCCCT
TTGTACACACCGCCCGTCGCTCCTACCGATCGAATGATACGGTAAAGCCAACGGATGG--
---A----G------CA-GTAG---CAA-T-ACG-A------G-CT-CTA-AAAG--TTG
-TT-TAAATCT--C----AT---GTT-
>JF892723.1_Raperostelium_reciprocatum_var._transitum_OH601
TCTAAG-TATAAATTTTTA-TATGATGAAA----CTGCAGACGGCTCATT--ACAA--CA
---GT----AATAAACTA-ATAGACTTTCGG-GTTTCA---TTACCTTTT-GGATAACCG
CAGTAAATCGGGGCTAATACATACAAT-CG-AGGGC-TGACTGTT-----TAC-G-G-AA
TGTCCGCGATTATT----A-GCA-TT--CAA-CCAATAC--CTCTCGGGGTTTTG-TGGT
GAAACCGAATAATATTGCAGATCGAAG--------CTT-CGGCTTCGACAAGTCTATTGT
GTTACTGCCCTATCAACTTTCGATGGTACGGTATTGGCCTACCATGGTTGTAACGGGTAA
CGGGGAATTAGGGTTCGATTCCGGAGAGGGCGCCTGAGAAATGGCGACCACTTCTACGGA
AGGCAGCAGGCGCGCAAATT-ACTCAATCCCAATAC-GGGGAAGTAGTGACAAGAAATAT
TAATGCCTATCC--TC-TTAGGAAG--GTAATTAAAATGGGTCTAAACTAAATCCATTTT
CT-AATACAATTGGAGGGCAAGTCTGGTGCCAGCAGCCGCGGTAATTCCAGCTCCAA-TA
GCATATACTAAATTTGTTGCGGTTAAAA-AGC-TCGTAGTTGAAGTAAAAGCCTTATT-G
GG-TTAA---------TAGGTCTTAGCCGACTTC--GTC--------GGTTTAA-----G
GCTTCCAATATGTGTTTTT-TAAAT--TAATT-CATGGATTAGGTTACTTTT-----GTA
ATTCTAGTTCTTTT--------------GGGTGTTTCACTGTGAGAAAA-TTGTGGTGCT
TAAAGCG--GG-C---GTT---T-AGCCTGATCTTTTGCAGCATGGTATGATAGAACATG
ACAT---TTTGCGC--TATTGGTT---GCG-----TTAAAGTGTAATGATTAATAGGGAT
GGATGGGGGTGTTCATATTGGTGGGCGAGAGGTGAAATTCGTTGACCCTATCAAGATGAA
-TTCTGCGAAAGCATTCACCAAATACTTCCCCATTAATCAAGAACGAAAGTTTGGGGATC
AAAGACGATCAGATACCGTCGTAGTCCAAACTATAAACTATGTCGACCAAGGATCAGCTA
AA--AT-TTTACAAAAATTTAGTTGGCACTTT-GTGAGAAATCAT-GAGTGTTTAGATTC
TGGGGGGAGTATGGTCGCAAGTCTGAAACTTAAAGGAATTGACGGAAGGGCACACAATGG
AGTGGAG-CCTGCGGCTTAATTT-GACTCAACTCGGGAAA-ACTTACCAAGCTAAGATAT
AATAAGGATTGACAGA-CTAA-AAGATCTTTCATGATTGTATAAGTGGTGGTGCATGGTC
GTTCTTAG-TTGGTGGAGCAATCTGTCTGGTCAATTCCGATAACGGACGAGATCTCGACC
TGCTAACTAGTAGTATATATCGATTCAATATGCATGAAAGCCTTGTTGGAGTAAGTTGTA
GGT----CGCAAGG-TCT-----ATGACACTTCGCTGGG----TCGGTAATGTGAGTTGA
TAA--ATACGAAAAATTAAAAAA---A--CTTCTTAGAGGGACTACCT-GTGGCAAACAG
GGGG-AAGTTCGAGGCAATAACAGGTCTGTGATGCCCTTAGATACCTT-GGGCCGCACGC
GCGCTACAATGTAGTACGCAAAAAGCA-----AT-----CC--TGGTCCGG-GAG---GA

CT-GGGTAATCATAA--ATTTACTACGTAACTGGGATTG-ATCTTTGTAATTTTTGATCA
TCAACGAGGAATTCCTTGTAAGCGCAAATCATTACTTTGTGCTGAATATGTCCCTGCCCT
TTGTACACACCGCCCGTCGCTCCTACCGATCGAATGATACGGTAAAGCCAACAGATGG--
---A----GT-----CA-GTAG---CAA-T-ACA-T------GACT-TTA-AAAG--TTG
-TT-TAAATCT--C----ATT--GTT-
>AM168076.1_Raperostelium_tenue_Pan52
TCTAAG-TATAAATTTTTA-TATGATGAAA----CTGCAGACGGCTCATT--ACAA--CA
---GT----AATAAACTA-ATAGACTTTCGG-GTTTCA---TTACCTTTT-GGATAACCG
CAGTAAATCGGGGCTAATACATACAAT-CG-AGGGC-TGACTGTT-----TAC-G-G-AA
TGTCCGCGATTATT----A-GCA-TT--CAA-CCAATAC--CTCTCGGGGTTTTG-TGGT
GAAACCGAATAATATTGCAGATCGAAG--------CTT-CGGCTTCGACAAGTCTATTGT
GTTACTGCCCTATCAACTTTCGATGGTACGGTATTGGCCTACCATGGTTGTAACGGGTAA
CGGGGAATTAGGGTTCGATTCCGGAGAGGGCGCCTGAGAAATGGCGACCACTTCTACGGA
AGGCAGCAGGCGCGCAAATT-ACTCAATCCCAATAC-GGGGAAGTAGTGACAAGAAATAT
TAATGCCTATCC--TC-TTAGGAAG--GTAATTAAAATGGGTCTAAACTAAATCCATTTT
CT-AATACAATTGGAGGGCAAGTCTGGTGCCAGCAGCCGCGGTAATTCCAGCTCCAA-TA
GCATATACTAAATTTGTTGCGGTTAAAA-AGC-TCGTAGTTGAAGTAAAAGCCTTATT-G
GG-TTAA---------TAGGTCTTAGCCGACTTC--GTC--------GGTTTAA-----G
GCTTCCAATATGTGTTTTT-TAAAT--TAATT-CATGGATTAGGTTACTTTT-----GTA
ATTCTAGTTCTTTT--------------GGGTGTTTCACTGTGAGAAAA-TTGTGGTGCT
TAAAGCG--GG-C---GTT---T-AGCCTGATCTTTTGCAGCATGGTATGATAGAACATG
ACAT---TTTGCGC--TATTGGTT---GCG-----TTAAAGTGTAATGATTAATAGGGAT
GGATGGGGGTGTTCATATTGGTGGGCGAGAGGTGAAATTCGTTGACCCTATCAAGATGAA
CTTCTGCGAAAGCATTCACCAAATACTTCCCCATTAATCAAGAACGAAAGTTTGGGGATC
AAAGACGATCAGATACCGTCGTAGTCCAAACTATAAACTATGTCGACCAAGGATCAGCTA
AA--AT-TTTACAAAAATTTAGTTGGCACTTT-GTGAGAAATCAT-GAGTGTTTAGATTC
TGGGGGGAGTATGGTCGCAAGTCTGAAACTTAAAGGAATTGACGGAAGGGCACACAATGG
AGTGGAG-CCTGCGGCTTAATTT-GACTCAACTCGGGAAA-ACTTACCAAGCTAAGATAT
AATAAGGATTGACAGA-CTAA-AAGATCTTTCATGATTGTATAAGTGGTGGTGCATGGTC
GTTCTTAG-TTGGTGGAGCAATCTGTCTGGTCAATTCCGATAACGGACGAGATCTCGACC
TGCTAACTAGTAGTATATATCGATTCAATATGCATGAAAGCCTTGTTGGAGTAAGTTGTA
GGT----CGCAAGG-TCT-----ATGACACTTCGCTGGG----TCGGTAATGTGAGTTGA
TAA--ATACGAAAAATTAAAAAA---A--CTTCTTAGAGGGACTACCT-GTGGCAAACAG
GGGG-AAGTTCGAGGCAATAACAGGTCTGTGATGCCCTTAGATACCTT-GGGCCGCACGC
GCGCTACAATGTAGTACGCAAAAAGCA-----AT-----CC--TGGTCCGG-GAG---GA
CT-GGGTAATCATAA--ATTTACTACGTAACTGGGATTG-ATCTTTGTAATTTTTGATCA
TCAACGAGGAATTCCTTGTAAGCGCAAATCATTACTTTGTGCTGAATATGTCCCTGCCCT
TTGTACACACCGCCCGTCGCTCCTACCGATCGAATGATACGGTAAAGCCAACAGATGG--
---A----GT-----CA-GTAG---CAA-T-ACA-T------GACT-TTA-AAAG--TTG
-TT-TAAATCT--C----ATT--GTTT
>JF892718.1_Raperostelium_reciprocatum_38A
TCTAAG-TATAAATTTTTA-TATGATGAAA----CTGCAGACGGCTCATT--ACAA--CA
---GT----AATAAACTA-ATAGACTTTCGG-GTTTCA---TTACCTTTT-GGATAACCG
CAGTAAATCGGGGCTAATACATACAAT-CG-AGGGC-TGACTGTT-----TAC-G-G-AA
TGTCCGCGATTATT----A-GCA-TT--CAA-CCAATAC--CTCTCGGGGTTTTG-TGGT
GAAACCGAATAATATTGCAGATCGAAG--------CTT-CGGCTTCGACAAGTCTATTGT
GTTACTGCCCTATCAACTTTCGATGGTACGGTATTGGCCTACCATGGTTGTAACGGGTAA
CGGGGAATTAGGGTTCGATTCCGGAGAGGGCGCCTGAGAAATGGCGACCACTTCTACGGA
AGGCAGCAGGCGCGCAAATT-ACTCAATCCCAATAC-GGGGAAGTAGTGACAAGAAATAT
TAATGCCTATCC--TC-TTAGGAAG--GTAATTAAAATGGGTCTAAACTAAATCCATTTT
CT-AATACAATTGGAGGGCAAGTCTGGTGCCAGCAGCCGCGGTAATTCCAGCTCCAA-TA
GCATATACTAAATTTGTTGCGGTTAAAA-AGC-TCGTAGTTGAAGTAAAAGCCTTATT-G
GG-TTAA---------TAGGTCTTAGCCGACTTC--GTC--------GGTTTAA-----G
GCTTCCAATATGTGTTTTT-TAAAT--TAATT-CATGGATTAGGTTACTTTT-----GTA
ATTCTAGTTCTTTT--------------GGGTGTTTCACTGTGAGAAAA-TTGTGGTGCT
TAAAGCG--GG-C---GTT---T-AGCCTGATCTTTTGCAGCATGGTATGATAGAACATG
ACAT---TTTGCGC--TATTGGTT---GCG-----TTAAAGTGTAATGATTAATAGGGAT
GGATGGGGGTGTTCATATTGGTGGGCGAGAGGTGAAATTCGTTGACCCTATCAAGATGAA
CTTCTGCGAAAGCATTCACCAAATACTTCCCCATTAATCAAGAACGAAAGTTTGGGGATC
AAAGACGATCAGATACCGTCGTAGTCCAAACTATAAACTATGTCGACCAAGGATCAGCTA
AA--AT-TTTACAAAAATTTAGTTGGCACTTT-GTGAGAAATCAT-GAGTGTTTAGATTC
TGGGGGGAGTATGGTCGCAAGTCTGAAACTTAAAGGAATTGACGGAAGGGCACACAATGG
AGTGGAG-CCTGCGGCTTAATTT-GACTCAACTCGGGAAA-ACTTACCAAGCTAAGATAT
AATAAGGATTGACAGA-CTAA-AAGATCTTTCATGATTGTATAAGTGGTGGTGCATGGTC
GTTCTTAG-TTGGTGGAGCAATCTGTCTGGTCAATTCCGATAACGGACGAGATCTCGACC
TGCTAACTAGTAGTATATATCGATTCAATATGCATGAAAGCCTTGTTGGAGTAAGTTGTA
GGT----CGCAAGG-TCT-----ATGACACTTCGCTGGG----TCGGTAATGTGAGTTGA
TAA--ATACGAAAAATTAAAAAA---A--CTTCTTAGAGGGACTACCT-GTGGCAAACAG
GGGG-AAGTTCGAGGCAATAACAGGTCTGTGATGCCCTTAGATACCTT-GGGCCGCACGC
GCGCTACAATGTAGTACGCAAAAAGCA-----AT-----CC--TGGTCCGG-GAG---GA
CT-GGGTAATCATAA--ATTTACTACGTAACTGGGATTG-ATCTTTGTAATTTTTGATCA
TCAACGAGGAATTCCTTGTAAGCGCAAATCATTACTTTGTGCTGAATATGTCCCTGCCCT
TTGTACACACCGCCCGTCGCTCCTACCGATCGAATGATACGGTAAAGCCAACAGATGG--

---A----GT-----CA-GTAG---CAA-T-ACA-T------GACT-TTA-AAAG--TTG
-TT-TAAATCT--C----ATT--GTT-
>HQ141490.1_Coremiostelium_polycephalum_Landolt_#1675_GUAM
TCTAAG-TATAAATCTTTA-TACGATGAAA----CTGCAGACGGCTCATT--ACAA--CA
---GT----GATAAACTA-CTAGACTTTCGG-GTTTCA---CGACCTTTT-GGATAACCG
CAGTAAATCGGGGCTAATACATATAAA-CG-AAGGG-CGACTGGGC----AACTG-G-AA
GCTCTGCGATTATT----A-GCT-TTT--TA-CCAATCC--CCGCAAGGGGCCAGTTGGT
GAGACCAAATAATGCTGCCGATCGAGA--------TCT-AATCT-CGACAAGTCTATTGT
GTCACTGCCCTATCAACTTTCGATGGTACGGTATTGGCCTACCATGGTTGTAACGGGTAA
CGGGGAATCAGGGTTCGATTCCGGAGAGGGAGCCTGAGAAATGGCTACCACTTCTATGGA
AGGCAGCAGGCGCGCAAATT-ACTCAATCCCAATAC-GGGGAAGTAGTGACAATAAATAT
CAATGCTCATCCTGAT-AAAGGAGG--GCAATTGAAATGAACACAAACTAAACCTCTTAA
TT-AATACAATTGGAGGGCAAGTCTGGTGCCAGCAGCCGCGGTAATTCCAGCTCCAA-TA
GCATATACTAAAGTTGTTGCAGTTAAAA-AGC-TCGTAGTTTAAACTAAGGTC-TAGGAG
GGCTCAA---------TATCACCTATCGGGCCTT------------GCGTTCAATA-GGG
GATCCTCTTAGAACTTTTT--AAA-----CTG-CCCATTCCTGGCTAGCAAT----AG--
-C-CCG---G-TTTA-------------GGGTGATTCACTGTGAGAAAA-TTGTGGTGTT
CAAAGCA--GG-C---GTC---TC-GCCTGATCCTTTGCAGCATGGTATGATAAAACACG
ACAC---CTAACGCCATATTGGTT---GCGA---TATTAAGTGTAATGATTAATAGGGAT
GGATGGGGATGTTCATATTGGTGGGCGAGAGGTGAAATTCGTTGACCCTATCAAGATGAA
CTTCTGCGAAAGCATTCATCAAATACTTCCCCATTAATCAAGAACGAAAGTTTGGGGATC
GAAGACGATCAGATACCGTCGTAGTCCAAACTATAAACTATGTCGACCAGGGATCGGCTG
GA--GT-TTTTTAAAAATCCAGCCGGCATCTT-GTGAGAAATCAC-GAGTGTTTAGATTC
CGGGGGGAGTATGGTCGCAAGTCTGAAACTTAAAGGAATTGACGGAAGGGCACACAATGG
AGTGGAG-CCTGCGGCTTAATTT-GACTCAACTCGGGAAA-ACTTACCAAGCTAAGATAT
AGCTATGATTGACAGA-CTAA-AAGATCTTTCATGATTCTATAAGTGGTGGTGCATGGTC
GTTCTTAG-TTGGTGGAGCGATTTGTCTGGTCAATTCCGATAACGGACGAGACCTCGACC
TGCTAACTAGTGGTGCCTATCCTATCGCCATGGGTGATAGGTAGTTGGGTGTGCCG-GTT
CCA-----GCAA--TGGT-----GCCGACCCTCGGCTAT----TGTGTAGCCTGATTGGA
TAGGCATCCAAATTC---AAAAA---A--CTTCTTAGAGGGACTACCT-GCCTCAAGCAG
GCGG-AAGTCCGAGGCAATAACAGGTCTGTGATGCCCTTAGATACCTT-GGGCCGCACGC
GCGCTACATTGCGATGCGCAAAAAGGT-----TG-----CC--TGGTCCGG-AAG---GA
TT-GGGTAATCA--GGAATTCATCACGTAACAGGGATTG-ATCTTTGTAATTATCGATCA
TCAACGAGGAATTCCTTGTAAGCGTAAGTCATTACCTTATGCTGAATATGTCCCTGCCCT
TTGTACACACCGCCCGTCGCTCCTACCGATCGAATGATACGGTAAAACCAACGGCATT--
--------GTTG--GCTTGCGT-A-AAA-CCAGGTC-------AGC-GAT-AATG--TTG
-TT-TAAACCT--C----ATT--GTT-
>AM168056.1_Coremiostelium_polycephalum_MY1_1
TCTAAG-TATAAATCTTTA-TACGATGAAA----CTGCAGACGGCTCATT--ACAA--CA
---GT----GATAAACTA-CTAGACTTTCGG-GTTTCA---CGACCTTTT-GGATAACCG
CAGTAAATCGGGGCTAATACATGTAAA-CG-AGGGG-CGACTGGGC----AACTA-G-AA
ACTCCGCGATTATT----A-GCT-TTT--TA-CCAATCC--CCGCAAGGGGCTAGTTGGT
GAAACCAAATAATGCTGCCGATCGAGA--------TTT-AATCT-CGACAAGTCTATTGT
GTCACTGCCCTATCAACTTTCGATGGTACGGTATTGGCCTACCATGGTTGTAACGGGTAA
CGGGGAATCAGGGTTCGATTCCGGAGAGGGAGCCTGAGAAATGGCTACCACTTCTATGGA
AGGCAGCAGGCGCGCAAATT-ACTCAATCCCAATAC-GGGGAAGTAGTGACAATAAATAT
CAATGCTCATCCTGAC-AAAGGAGG--GTAATTGAAATGAACACAAACTAAACCTCTTAA
TT-AATACAATTGGAGGGCAAGTCTGGTGCCAGCAGCCGCGGTAATTCCAGCTCCAA-TA
GCATATACTAAAGTTGTTGCAGTTAAAA-AGC-TCGTAGTTTAAATAAAGGTC-TAGGGG
GGCTCAA---------TATTTCCTATTGGGTCTT------------GCATTCAATATGGG
AATCCCCTTAGAACTTTTT--AAA-----CTG-CCCATTTCAGCCGGGCAAC----CGG-
------T-TGTCTTC-------------GGGTGATTCACTGTGAGAAAA-TTGTGGTGTT
CAAAGCA--GG-C---GTC---TC-GCCTGATCCTTTGCAGCATGGTATGATAGAACACG
ACAC---ATAACGCCACATTGGTT---GCGA----TT-AAGTGTAATGATTAATAGGGAT
GGATGGGGATGTTCATATTGGTGGGCGAGAGGTGAAATTCGTTGACCCTATCAAGATGAA
CTTCTGCGAAAGCATTCATCAAATACTTCTCCATTAATCAAGAACGAAAGTTTGGGGATC
GAAGACGATCAGATACCGTCGTAGTCCAAACTATAAACTATGTCGACCAGGGATCGGCTG
GA--GT-TCTTTAAAAATCCAGTCGGCACCTT-GTGAGAAATCAC-GAGTGTTTAGATTC
CGGGGGGAGTATGGTCGCAAGTCTGAAACTTAAAGGAATTGACGGAAGGGCACACAATGG
AGTGGAG-CCTGCGGCTTAATTT-GACTCAACTCGGGAAA-ACTTACCAAGCTAAGATAT
AGCTAGGATTGACAGA-CTAA-AAGATCTTTCATGATTCTATAAGTGGTGGTGCATGGTC
GTTCTTAG-TTGGTGGAGCGATTTGTCTGGTCAATTCCGATAACGGACGAGACCTCGACC
TGCTAACTAGTGGTGTCTATCCTATCGCCATGGGTGATAGCTAGGTGGGTGTGTT--GTA
GACG---GGCAA--CCGTT----TGCTTCCCTCGCCTGG----TGTGTAGCCTGATTGGA
TAGGCATCCAAAAAC---AAAAT---A--CTTCTTAGAGGGACTACCT-GCCTCAAGCAG
GCGG-AAGTCCGAGGCAATAACAGGTCTGTGATGCCCTTAGATACCTT-GGGCCGCACGC
GCGCTACATTGCGATGCGCAAAAAGGT-----TG-----CC--TGGTCCGG-AAG---GA
TT-GGGTAATCA--GGAATTCATCACGTAACAGGGATTG-ATCTTTGTAATTATCGATCA
TCAACGAGGAATTCCTTGTAAGCGTAAGTCATTACCTTACGCTGAATATGTCCCTGCCCT
TTGTACACACCGCCCGTCGCTCCTACCGATCGAATGATACGGTAAAACCAACGGCATC--
--------GTTG--GCTGGTTT-A-ATC-GCTAGTC------T-GT-GAT-AATG--TTG
-TT-TAAACCT--C----ATT--GTTT
>HQ141489.1_Coremiostelium_polycephalum_Landolt_#2132_B_9c

TCTAAG-TATAAATCTTTA-TACGATGAAA----CTGCAGACGGCTCATT--ACAA--CA
---GT----GATAAACTA-CTAGACTTTCGG-GTTTCA---CGACCTTTT-GGATAACCG
CAGTAAATCGGGGCTAATACATGTAAA-CG-AGGGG-TGACTGGGC----AACTG-G-AA
GCTCCGCGATTATT----A-GCT-TTT--TA-CCAATCC--CCGCAAGGGGTCAGTTGGT
GAGACCAAATAATGCTGCCGATCGAGA--------TTT-AATCT-CGACAAGTCTACTGT
GTCACTGCCCTATCAACTTTCGATGGTACGGTATTGGCCTACCATGGTTGTAACGGGTAA
CGGGGAATCAGGGTTCGATTCCGGAGAGGGAGCCTGAGAAATGGCTACCACTTCTATGGA
AGGCAGCAGGCGCGCAAATT-ACTCAATCCCAATAC-GGGGAAGTAGTGACAATAAATAT
CAATGCTCATCCTGAC-AAAGGAGG--GTAATTGAAATGAACACAAACTAAACCTCTTAA
TT-AATACAATTGGAGGGCAAGTCTGGTGCCAGCAGCCGCGGTAATTCCAGCTCCAA-TA
GCATATACTAAAGTTGTTGCAGTTAAAA-AGC-TCGTAGTTTAAGTAAAGGTC-TAGGGG
GGCTCAA---------TATTCCCTATTGGGTCTC------------GCATTCAATATGGG
GATCCCCTTAGAACTTTTT--AAA-----CTG-CCCATTTCAGGCGTCCCGC----AAGG
GCTCCGTCTGGCTTC-------------GGGTGATTCACTGTGAGAAAA-TTGTGGTGTT
CAAAGCA--GG-C---GTC---TC-GCCTGATCCTTTGCAGCATGGTATGATAAAACACG
ACAC---CTAACGCCACATTGGTT---GCGA----TT-AAGTGTAATGATTAATAGGGAT
GGATGGGGATGTTCATATTGGTGGGCGAGAGGTGAAATTCGTTGACCCTATCAAGATGAA
CTTCTGCGAAAGCATTCATCAAATACTTCTCCATTAATCAAGAACGAAAGTTTGGGGATC
GAAGACGATCAGATACCGTCGTAGTCCAAACTATAAACTATGTCGACCAGGGATCGGCTG
GA--GT-TCTTTAAAAATCCAGTCGGCACCTT-GTGAGAAATCAC-GAGTGTTTAGATTC
CGGGGGGAGTATGGTCGCAAGTCTGAAACTTAAAGGAATTGACGGAAGGGCACACAATGG
AGTGGAG-CCTGCGGCTTAATTT-GACTCAACTCGGGAAA-ACTTACCAAGCTAAGATAT
AGCTAGGATTGACAGA-CTAA-AAGATCTTTCATGATTCTATAAGTGGTGGTGCATGGTC
GTTCTTAG-TTGGTGGAGCGATTTGTCTGGTCAATTCCGATAACGGACGAGACCTCGACC
TGCTAACTAGTGGTGTCTATCCTATCGCCATGGGTGATAGTTAGCTGGGTGTCTGG-CTC
GCTC----GCAA--GAGCG----GGTCTGCCTCGGCTGG----CGTGTAGCCTGATTGGA
TAGGCATCCAAAAAC---AAAAA---A--CTTCTTAGAGGGACTACCT-GCCTCAAGCAG
GCGG-AAGTCCGAGGCAATAACAGGTCTGTGATGCCCTTAGATACCTT-GGGCCGCACGC
GCGCTACATTGCGATGCGCAAAAAGGT-----TG-----CC--TGGTCCGG-AAG---GA
TT-GGGTAATCA--GGAATTCATCACGTAACAGGGATTG-ATCTTTGTAATTATCGATCA
TCAACGAGGAATTCCTTGTAAGCGTAAGTCATTACCTTACGCTGAATATGTCCCTGCCCT
TTGTACACACCGCCCGTCGCTCCTACCGATCGAATGATACGGTAAAACCAACGGCATT--
--------GCTG--GCTGGCTT-A-AAC-TCTGGTC------TAGC-GAT-AATG--TTG
-TT-TAAACCT--C----ATT--GTT-
>HQ141488.1_Coremiostelium_polycephalum_Landolt_#1130_SS3B
TCTAAG-TATAAATCTTTA-TACGATGAAA----CTGCAGACGGCTCATT--ACAA--CA
---GT----GATAAACTA-CTAGACTTTCGG-GTTTCA---CGACCTTTT-GGATAACCG
CAGTAAATCGGGGCTAATACATGTAAA-CG-AGGGG-TGACTGGGC----AACTG-G-AA
GCTCCGCGATTATT----A-GCT-TTT--TA-CCAATCC--CCGCAAGGGGTCAGTTGGT
GAGACCAAATAATGCTGCCGATCGAGA--------TCT-AATCT-CGACAAGTCTACTGT
GTCACTGCCCTATCAACTTTCGATGGTACGGTATTGGCCCTACCATGGTTGTAACGGGTAA
CGGGGAATCAGGGTTCGATTCCGGAGAGGGAGCCTGAGAAATGGCTACCACTTCTATGGA
AGGCAGCAGGCGCGCAAATT-ACTCAATCCCAATAC-GGGGAAGTAGTGACAATAAATAT
CAATGCTCATCCTGAT-AAAGGAGG--GTAATTGAAATGAACACAAACTAAACCTCTTAA
TT-AATACAATTGGAGGGCAAGTCTGGTGCCAGCAGCCGCGGTAATTCCAGCTCCAA-TA
GCATATACTAAAGTTGTTGCAGTTAAAA-AGC-TCGTAGTTTAAGTAAAGGTC-TGGGGG
GGCTCAA---------TATTCCCTATTGGGTCTC------------GCATTCAATACGGG
GATCCCCTCAGAACTTTTT--AAA-----CTG-CCCATTTCAGGCG-CTCGC----GC--
------TCTGACTTC-------------GGGTGATTCACTGTGAGAAAA-TTGTGGTGTT
CAAAGCA--GG-C---GTC---TC-GCCTGATCCTTTGCAGCATGGTATGATAGAACACG
ACAC---CTAACGCCACATTGGTT---GCGA----TT-AAGTGTAATGATTAATAGGGAT
GGATGGGGATGTTCATATTGGTGGGCGAGAGGTGAAATTCGTTGACCCTATCAAGATGAA
CTTCTGCGAAAGCATTCATCAAATACTTCTCCATTAATCAAGAACGAAAGTTTGGGGATC
GAAGACGATCAGATACCGTCGTAGTCCAAACTATAAACTATGTCGACCAGGGATCGGCTG
GA--GT-TCTTTAAAAATCCAGTCGGCACCTT-GTGAGAAATCAC-GAGTGTTTAGATTC
CGGGGGGAGTATGGTCGCAAGTCTGAAACTTAAAGGAATTGACGGAAGGGCACACAATGG
AGTGGAG-CCTGCGGCTTAATTT-GACTCAACTCGGGAAA-ACTTACCAAGCTAAGATAT
AGCTAGGATTGACAGA-CTAA-AAGATCTTTCATGATTCTATAAGTGGTGGTGCATGGTC
GTTCTTAG-TTGGTGGAGCGATTTGTCTGGTCAATTCCGATAACGGACGAGACCTCGACC
TGCTAACTAGTGGTGTCTATCCTATCGCCATGGGTGATAGCCAGATGGGTGTGTT--TTC
GTTC---AGCAA--TGAGC----GGGATCCCTCGCCTGG----TGTGTAGCCTGATTGGA
TAGGCATCCAAAATC---AAAAA------CTTCTTAGAGGGACTACCT-GCCTCAAGCAG
GCGG-AAGTCCGAGGCAATAACAGGTCTGTGATGCCCTTAGATACCTT-GGGCCGCACGC
GCGCTACATTGCGATGCGCAAAAAGGT-----TG-----CC--TGGTCCGG-AAG---GA
TT-GGGTAATCA--GGAATTCATCACGTAACAGGGATTG-ATCTTTGTAATTATCGATCA
TCAACGAGGAATTCCTTGTAAGCGTAAGTCATTACCTTACGCTGAATATGTCCCTGCCCT
TTGTACACACCGCCCGTCGCTCCTACCGATCGAATGATACGGTAAAACCAACGGCATT--
--------GCTG--GCTGGCTT-A-ACC-TCTGGTC------TAGC-GAT-AATG--TTG
-TT-TAAACCT--C----ATT--GTT-
>JX173877.1_Polysphondylium_fuscans_Sweden_11D
TCTAAG-TATAAATTCTTG-TACGATGAAA----CTGCAGACGGCTCATT--ACAA--CA
---GT----GATAAACTA-ATAGACTTTCGG-GTTTT------ACCT-TTTGGATAACCG
CAGTAAATCGGGGCTAATACATACAAG-CG-AGGGG-TGATTGATT---TATC-A-A-GA

GCTCCGCGATTATT----A-GCA-TT--CAA-CCAATAC--CCGCAAGGGTTCTGTTGGT
GAAACCGAATAATATTGCAGATCGAAG--------ATT-TATCTTCGACAAGTCTACTGT
GTCACTGCCCTATCAACTTTCGATGGTACGGTATTGGCCTACCATGGTTGTAACGGGTAA
CGGGGAATTAGGGTTCGATTCCGGAGAGGGAGCCTGAGAAATGGCTACCACTTCTACGGA
AGGCAGCAGGCGCGCAAATT-ACTCAATCCCAATAC-GGGGAAGTAGTGACAATAAATAT
CGATGCCTAACCATTT-TTGGAAGG--GTAATTGAAATGAACACAAATTAAAACTCTTAA
TT-AATACAATTGGAGGGCAAGTCTGGTGCCAGCAGCCGCGGTAATTCCAGCTCCAA-TA
GCATATACTAAAGTTGTTGCAGTTAAAA-AGC-TCGTAGTTGAAGTTAAAGCTTTATTTG
GG-TTAA--------AAATTCATTTA-CCGTTTA----------T-TGGTTAAATC---G
AATTCCAGTATTGCTTTTT-T-------AAAG-TTCAGTTTGTATTGCCTT-------TG
GTAGTATTTATTTG---------------GACATTTCACTGTGAGAAAA-TTGTGGTGTT
TAAAGCA--GG-C---GTC---TCTGCCTGATCTTTTGCAGCATGGTATGATGGAACATG
ACAT---TTTGCGC--AATTGGTT---GCG----ATTAAAGTGTAATGATTAATAGGGAT
GGATGGGGGTGTTCATATTGGTGGGCGAGAGGTGAAATTCGTTGACCCTATCAAGATGAA
CTTCTGCGAAAGCATTCACCAAATACTTCCCCATTAATCAAGAACGAAAGTTTGGGGATC
GAAGACGATCAGATACCGTCGTAGTCCAAACTATAAACTATGTCGACCAGGGATCGGTTA
AA--AC-TTTTTAAAAGTTTAATCGGCACCTT-GTGAGAAATCAC-GAGTGTTTAGATTC
CGGGGGGAGTATGGTCGCAAGTCTGAAACTTAAAGGAATTGACGGAAGGGCACACAATGG
AGTGGAG-CCTGCGGCTTAATTT-GACTCAACTCGGGAAA-ACTTACCAAGCTAAGATAT
AGTAAGGATTGACAGA-CTAA-GAGATCTTTCATGATTCTATAAGTGGTGGTGCATGGTC
GTTCTTAG-TTGGTGGAGCGATTTGTCTGGTCAATTCCGATAACGGACGAGACCTCGACC
TGCTAACTAGTAGTATTTATTG-GCCGTTATGGATGATAGTCATT-CGGGGTTTGG-AAG
GGCT----TC--GGTTCTG----TCC--GCTTCGTGTGG----TGTGTAATCTGGTCGGA
TAG--GTACGAATTA------AA---A--CTTCTTAGAGGGACTACCT-GCCTCAAGCAG
GCGG-AAGTCCGAGGCAATAACAGGTCTGTGATGCCCTTAGATACCTT-GGGCCGCACGC
GCGCTACAATGTAGAAAGCAAAAAGGT-----TCC--------TGGTCCGG-AAG---GA
TT-GGGTAATCAATTGAATTTTCTACGTAACTGGGATTG-ATCTTTGTAATTATTGATCA
TCAACGAGGAATTCCTTGTAAGCGTAAGTCATTACCTTATGCTGAATATGTCCCTGCCCT
TTGTACACACCGCCCGTCGCTCCTACCGATCGAATGATACGGTAAAGCCAACGGATAA--
--------GGTC--CTATTGGG-C-AAC-CAGTATG------GATT-TTA-AAAG--TTG
-TT-TAAATCT--C----ATT--GTT-
>HQ141487.1_Polysphondylium_sp._Tibet_10A
TCTA-G-TATAAATTCTTG-TACGATGAAA----CTGCAGACGGCTCATT--ACAA--CA
---GT----GATAAACTA-ATAGACTTTCGG-GTTTT------ACCT-TTTGGATAACCG
CAGTAAATCGGGGCTAATACATACAAG-CG-AGGGG-TGATTGATT---TATC-A-A-GA
GCTCCGCGATTATT----A-GCA-TT--CAA-CCAATAC--CCGCAAGGGTTCTGTTGGT
GAAACCGAATAATATTGCAGATCGAAG--------ATT-TATCTTCGACAAGTCTACTGT
GTCACTGCCCTATCAACTTTCGATGGTACGGTATTGGCCTACCATGGTTGTAACGGGTAA
CGGGGAATTAGGGTTCGATTCCGGAGAGGGAGCCTGAGAAATGGCTACCACTTCTACGGA
AGGCAGCAGGCGCGCAAATT-ACTCAATCCCAATAC-GGGGAAGTAGTGACAATAAATAT
CGATGCCTAACCATTT-TTGGAAGG--GTAATTGAAATGAACACAAATTAAAACTCTTAA
TT-AATACAATTGGAGGGCAAGTCTGGTGCCAGCAGCCGCGGTAATTCCAGCTCCAA-TA
GCATATACTAAAGTTGTTGCAGTTAAAA-AGC-TCGTAGTTGAAGTTAAAGCTTTATTTG
GG-TTAA--------AAATTCATTTA-CCGTTTA----------T-TGGTTAAATC---G
AATTCCAGTATTGCTTTTT-T-------AAAG-TTCAGTTTGTATTGCCTT-------TG
GTAGTATTTATTTG---------------GACATTTCACTGTGAGAAAA-TTGTGGTGTT
TAAAGCA--GG-C---GTC---TCTGCCTGATCTTTTGCAGCATGGTATGATGGAACATG
ACAT---TTTGCGC--AATTGGTT---GCG----ATTAAAGTGTAATGATTAATAGGGAT
GGATGGGGGTGTTCATATTGGTGGGCGAGAGGTGAAATTCGTTGACCCTATCAAGATGAA
CTTCTGCGAAAGCATTCACCAAATACTTCCCCATTAATCAAGAACGAAAGTTTGGGGATC
GAAGACGATCAGATACCGTCGTAGTCCAAACTATAAACTATGTCGACCAGGGATCGGTTA
AA--AC-TTTTAAAAGTTTAATCGGCACCTT-GTGAGAAATCAC-GAGTGTTTAGATTC
CGGGGGGAGTATGGTCGCAAGTCTGAAACTTAAAGGAATTGACGGAAGGGCACACAATGG
AGTGGAG-CCTGCGGCTTAATTT-GACTCAACTCGGGAAA-ACTTACCAAGCTAAGATAT
AGTAAGGATTGACAGA-CTAA-GAGATCTTTCATGATTCTATAAGTGGTGGTGCATGGTC
GTTCTTAG-TTGGTGGAGCGATTTGTCTGGTCAATTCCGATAACGGACGAGACCTCGACC
TGCTAACTAGTAGTATTTATTG-GCCGTTATGGATGATAGTCATT-CGGGGTTAGG-AAG
GGCT----TC--GGTTCTC----TCC--GCTTCGTGTGG----TGTGTAATCTGGTCGGA
TAG--GTACGAATTA------AA---A--CTTCTTAGAGGGACTACCT-GCCTCAAGCAG
GCGG-AAGTCCGAGGCAATAACAGGTCTGTGATGCCCTTAGATACCTT-GGGCCGCACGC
GCGCTACAATGTAGAAAGCAAAAAGGT-----TCC--------TGGTCCGG-AAG---GA
TT-GGGTAATCAATTGAATTTTCTACGTAACTGGGATTG-ATCTTTGTAATTATTGATCA
TCAACGAGGAATTCCTTGTAAGCGTAAGTCATTACCTTATGCTGAATATGTCCCTGCCCT
TTGTACACACCGCCCGTCGCTCCTACCGATCGAATGATACGGTAAAGCCAACGGATAA--
--------GGTC--CTATTGGG-C-AAC-CAGTATG------GATT-TTA-AAAG--TTG
-TT-TAAATCT--C----ATT--GTTT
>AM168046.1_Polysphondylium_laterosorum_AE4
TCTAAG-TATAAATTCTTG-TACGATGAAA----CTGCAGACGGCTCATT--ACAA--CA
---GT----GATAAACTA-ATAGACTTTCGG-GTTTT------ACCT-TTTGGATAACCG
CAGTAAATCGGGGCTAATACATAGAAG-CG-AGGGG-TGACTGATT---TATC-G-G-AA
GCTCCGCGATTATT----A-GCA-TT--CAA-CCAATAC--CCGCAAGGGTTCTGTTGGT
GAAACCGAATAATATTGCAGATCGAAG--------ATT-TATCTTCGACAAGTCTACTGT
GTCACTGCCCTATCAACTTTCGATGGTACGGTATTGGCCTACCATGGTTGTAACGGGTAA

CGGGGAATTAGGGTTCGATTCCGGAGAGGGAGCCTGAGAAATGGCTACCACTTCTACGGA
AGGCAGCAGGCGCGCAAATT-ACTCAATCCCAATAC-GGGGAAGTAGTGACAATAAATAT
CGATGCCTATCCATTT-ATGGAAGG--GTAATTGAAATGAACACAAATTAAAACTCTTAA
TT-AATACAATTGGAGGGCAAGTCTGGTGCCAGCAGCCGCGGTAATTCCAGCTCCAA-TA
GCATATACTAAAGTTGTTGCAGTTAAAA-AGC-TCGTAGTTGAAGTTAAAGCTTTATTTG
GG-TTAA--------AAATTCATTTG-CCTTTTT----------T-TGGTTAAATA---G
AATTCCAGTATTGCTTTTT-T-------AAAG-TTCAGTTTGTAGTACTTT---------
GTATTACTTATTTG---------------GACATTTCACTGTGAGAAAA-TTGTGGTGTT
TAAAGCA--GG-C---GTC---TCTGCCTGATCTTTTGCAGCATGGTATGATGGAACATG
ACAT---TTTGCGC--AATTGGTT---GCG----ATTAAAGTGTAATGATTAATAGGGAT
GGATGGGGGTGTTCATATTGGTGGGCGAGAGGTGAAATTCGTTGACCCTATCAAGATGAA
CTTCTGCGAAAGCATTCACCAAATACTTCCCCATTAATCAAGAACGAAAGTTTGGGGATC
GAAGACGATCAGATACCGTCGTAGTCCAAACTATAAACTATGTCGACCAGGGATCGGTTA
AA--AC-TTTTTAAAAGTTTAATCGGCACCTT-GTGAGAAATCAC-GAGTGTTTAGATTC
CGGGGGGAGTATGGTCGCAAGTCTGAAACTTAAAGGAATTGACGGAAGGGCACACAATGG
AGTGGAG-CCTGCGGCTTAATTT-GACTCAACTCGGGAAA-ACTTACCAAGCTAAGATAT
AGTAAGGATTGACAGA-CTAA-GAGATCTTTCATGATTCTATAAGTGGTGGTGCATGGTC
GTTCTTAG-TTGGTGGAGCGATTTGTCTGGTCAATTCCGATAACGGACGAGACCTCGACC
TGCTAACTAGTAGTATTTATTG-GCCGTTATGGATGATAGCTATTTCGGGGTTTGG-AAG
GTCT----TC--GGATCTC----TCC--GCTTCGTGTGG----TGTGTAATCTGGTCGGA
TAG--GTACGAATTA------AA---A--CTTCTTAGAGGGACTACCT-GCCTCAAGCAG
GCGG-AAGTCCGAGGCAATAACAGGTCTGTGATGCCCTTAGATACCTT-GGGCCGCACGC
GCGCTACAATGTAGAAAGCAAAAAGGT-----TCC--------TGGTCCGG-AAG---GA
TT-GGGTAATCAATTGAATTTTCTACGTAACTGGGATTG-ATCTTTGTAATTATTGATCA
TCAACGAGGAATTCCTTGTAAGCGTAAGTCATTACCTTATGCTGAATATGTCCCTGCCCT
TTGTACACACCGCCCGTCGCTCCTACCGATCGAATGATACGGTAAAGCCAACGGATAA--
--------GATC--CTATTGGG-C-AAC-TAATACG------GATT-TTA-AAAG--TTG
-TT-TAAATCT--C----ATT--GTTT
>AM168108.1_Polysphondylium_violaceum_P6
TCTAAG--ATAAAT-CTTG-TACGATGAAA----CTGCAGACGGCTCATT--ACAA--CA
---GT----GATAAACTA-ATAGACTTTCGG-GTTTT------ACCNCNTTGGAAAACCG
CAGTAAATCGGGGCTAATACATAGAAG-CG-AGGGG-TGACTGATT---TATC-G-G-AA
GCTCCGCGATTATT----A-GCA-TT--CAA-CCAATAC--CCGCAAGGGTTCTGTTGGT
GAAACCGAATAATATTGCAGATCGAAG--------ATT-TATCTTCGACAAGTCTACTGT
GTCACTGCCCTATCAACTTTCGATGGTACGGTATTGGCCTACCATGGTTGTAACGGGTAA
CGGGGAATTAGGGTTCGATTCCGGAGAGGGAGCCTGAGAAATGGCTACCACTTCTACGGA
AGGCAGCAGGCGCGCAAATT-ACTCAATCCCAATAC-GGGGAAGTAGTGACAATAAATAT
CGATGCCTATCCATTT-TTGGAAGG--GTAATTGAAATGAACACAAATTAAAACTCTTAA
TT-AATACAATTGGAGGGCAAGTCTGGTGCCAGCAGCCGCGGTAATTCCAGCTCCAA-TA
GCATATACTAAAGTTGTTGCAGTTAAAA-AGC-TCGTAGTTGAAGTTAAAGCTTTATTTG
GG-TTAA--------AAATCCATTTG-CCTTTTA----------A-CGGTTAAATT---G
GATTCCAGTATTGCTTCTT-T-------AAAG-TTCAGTTTGTATTGCCTT-------TG
GTAGTATTTATTTG---------------GACATTTCACTGTGAGAAAA-TTGTGGTGTT
TAAAGCA--GG-C---GTC---TCTGTCTGATCTTTTGCAGCATGGTATGATGGAACATG
ACAT---TTTGCGC--AATTGGTT---GCG----ATTAAAGTGTAATGATTAATAGGGAT
GGATGGGGGTGTTCATATTGGTGGGCGAGAGGTGAAATTCGTTGACCCTATCAAGATGAA
CTTCTGCGAAAGCATTCACCAAATACTTCCCCATTAATCAAGAACGAAAGTTTGGGGATC
GAAGACGATCAGATACCGTCGTAGTCCAAACTATAAACTATGTCGACCAGGGATCGGTTA
AA--AC-TTTTTAAAAGTTTAATCGGCACCTT-GTGAGAAATCAC-GAGTGTTTAGATTC
CGGGGGGAGTATGGTCGCAAGTCTGAAACTTAAAGGAATTGACGGAAGGGCACACAATGG
AGTGGAG-CCTGCGGCTTAATTT-GACTCAACTCGGGAAA-ACTTACCAAGCTAAGATAT
AGTAAGGATTGACAGA-CTAA-GAGATCTTTCATGATTCTATAAGTGGTGGTGCATGGTC
GTTCTTAG-TTGGTGGAGCGATTTGTCTGGTCAATTCCGATAACGGACGAGACCTCGACC
TGCTAACTAGTAGTATTTATTG-GCCGTTATGGATGATAGCTATT-CGGGGTTTGG-AAG
GGCT----TC--GGTTCTC----TCC--GCTTCGTGTGG----TGTGTAATCTGGTCGGA
TAG--GTACGAATTA------AA---A--CTTCTTAGAGGGACTACCT-GCCTCAAGCAG
GCGG-AAGTCCGAGGCAATAACAGGTCTGTGATGCCCTTAGATACCTT-GGGCCGCACGC
GCGCTACAATGTAGAAAGCAAAAAGGT-----TCC--------TGGTCCGG-AAG---GA
TT-GGGTAATCAATTGAATTTTCTACGTAACTGGGATTG-ATCTTTGTAATTATTGATCA
TCAACGAGGAATTCCTTGTAAGCGTAAGTCATTACCTTATGCTGAATATGTCCCTGCCCT
TTGTACACACCGCCCGTCGCTCCTACCGATCGAATGATACGGTAAAGCCAACGGATAA--
--------GATC--CTATTGGG-C-AAC-TAATATG------GATT-TTA-AAAG--TTG
-TT-TAAATCT--C----ATT--GTTT
>HQ141486.1_Polysphondylium_violaceum_209
TCTA-G-TATAAATTCTTG-TACGATGAAA----CTGCAGACGGCTCATT--ACAA--CA
---GT----GATAAACTA-ATAGACTTTCGG-GTTTT------ACCT-TTTGGATAACCG
CAGTAAATCGGGGCTAATACATAGAAG-CG-AGGGG-TGACTGATT---TATC-G-G-AA
GCTCCGCGATTATT----A-GCA-TT--CAA-CCAATAC--CCGCAAGGGTTCTGTTGGT
GAAACCGAATAATATTGCAGATCGAAG--------ATT-TATCTTCGACAAGTCTACTGT
GTCACTGCCCTATCAACTTTCGATGGTACGGTATTGGCCTACCATGGTTGTAACGGGTAA
CGGGGAATTAGGGTTCGATTCCGGAGAGGGAGCCTGAGAAATGGCTACCACTTCTACGGA
AGGCAGCAGGCGCGCAAATT-ACTCAATCCCAATAC-GGGGAAGTAGTGACAATAAATAT
CGATGCCTATCCATTT-TTGGAAGG--GTAATTGAAATGAACACAAATTAAAACTCTTAA

TT-AATACAATTGGAGGGCAAGTCTGGTGCCAGCAGCCGCGGTAATTCCAGCTCCAA-TA
GCATATACTAAAGTTGTTGCAGTTAAAA-AGC-TCGTAGTTGAAGTTAAAGCTTTATTTG
GG-TTAA--------AAATCCATTTG-CCTTTTA----------A-CGGTTAAATT---G
GATTCCAGTATTGCTTTTT-T-------AAAG-TTCAGTTTGTATTGCCTT-------TG
GTAGTATTTATTTG---------------GACATTTCACTGTGAGAAAA-TTGTGGTGTT
TAAAGCA--GG-C---GTC---TCTGTCTGATCTTTTGCAGCATGGTATGATGGAACATG
ACAT---TTTGCGC--AATTGGTT---GCG----ATTAAAGTGTAATGATTAATAGGGAT
GGATGGGGGTGTTCATATTGGTGGGCGAGAGGTGAAATTCGTTGACCCTATCAAGATGAA
CTTCTGCGAAAGCATTCACCAAATACTTCCCCATTAATCAAGAACGAAAGTTTGGGGATC
GAAGACGATCAGATACCGTCGTAGTCCAAACTATAAACTATGTCGACCAGGGATCGGTTA
AA--AC-TTTTTAAAAGTTTAATCGGCACCTT-GTGAGAAATCAC-GAGTGTTTAGATTC
CGGGGGGAGTATGGTCGCAAGTCTGAAACTTAAAGGAATTGACGGAAGGGCACACAATGG
AGTGGAG-CCTGCGGCTTAATTT-GACTCAACTCGGGAAA-ACTTACCAAGCTAAGATAT
AGTAAGGATTGACAGA-CTAA-GAGATCTTTCATGATTCTATAAGTGGTGGTGCATGGTC
GTTCTTAG-TTGGTGGAGCGATTTGTCTGGTCAATTCCGATAACGGACGAGACCTCGACC
TGCTAACTAGTAGTATTTATTG-GCCGTTATGGATGATAGCTATT-CGGGGTTTGG-AAG
GGCT----TC--GGTTCTC----TCC--GCTTCGTGTGG----TGTGTAATCTGGTCGGA
TAG--GTACGAATTA------AA---A--CTTCTTAGAGGGACTACCT-GCCTCAAGCAG
GCGG-AAGTCCGAGGCAATAACAGGTCTGTGATGCCCTTAGATACCTT-GGGCCGCACGC
GCGCTACAATGTAGAAAGCAAAAAGGT-----TCC--------TGGTCCGG-AAG---GA
TT-GGGTAATCAATTGAATTTTCTACGTAACTGGGATTG-ATCTTTGTAATTATTGATCA
TCAACGAGGAATTCCTTGTAAGCGTAAGTCATTACCTTATGCTGAATATGTCCCTGCCCT
TTGTACACACCGCCCGTCGCTCCTACCGATCGAATGATACGGTAAAGCCAACGGATAA--
--------GATC--CTATTGGG-C-AAC-TAATATG------GATT-TTA-AAAG--TTG
-TT-TAAATCT--C----ATT--GTTT
>HQ141485.1_Polysphondylium_sp._Laos4_Laos4
TCTAAG-TATAAATTCTTG-TACGATGAAA----CTGCAGACGGCTCATT--ACAA--CA
--TGT----GATAAACTA-ATAGACTTTCGG-GTTTT------ACCT-TTTGGATAACCG
CAGTAAATCGGGGCTAATACATAGAAG-CG-AGGGG-TGACTGATT---TATC-G-G-AA
GCTCCGCGATTATT----A-GCA-TT--CAA-CCAATAC--CCGCAAGGGTTCTGTTGGT
GAAACCGAATAATATTGCAGATCGAAG--------ATT-TATCTTCGACAAGTCTACTGT
GTCACTGCCCTATCAACTTTCGATGGTACGGTATTGGCCTACCATGGTTGTAACGGGTAA
CGGGGAATTAGGGTTCGATTCCGGAGAGGGAGCCTGAGAAATGGCTACCACTTCTACGGA
AGGCAGCAGGCGCGCAAATT-ACTCAATCCCAATAC-GGGGAAGTAGTGACAATAAATAT
CGATGCCTATCCATTT-ATGGAAGG--GTAATTGAAATGAACACAAATTAAAACTCTTAA
TT-AATACAATTGGAGGGCAAGTCTGGTGCCAGCAGCCGCGGTAATTCCAGCTCCAA-TA
GCATATACTAAAGTTGTTGCAGTTAAAA-AGC-TCGTAGTTGAAGTTAAAGCTTTATTTG
GG-TTAA--------AAATCCATTTG-CCTTTTA----------A-CGGTTAAATA---G
GATTCCAGTATTGCTTTTT-T-------AAAG-TTCAGTTTGTATTGCCTT-------TG
GTAGTATTTATTTG---------------GACATTTCACTGTGAGAAAA-TTGTGGTGTT
TAAAGCA--GG-C---GTC---TCTGTCTGATCTTTTGCAGCATGGTATGATGGAACATG
ACAT---TTTGCGC--AATTGGTT---GCG----ATTAAAGTGTAATGATTAATAGGGAT
GGATGGGGGTGTTCATATTGGTGGGCGAGAGGTGAAATTCGTTGACCCTATCAAGATGAA
CTTCTGCGAAAGCATTCACCAAATACTTCCCCATTAATCAAGAACGAAAGTTTGGGGATC
GAAGACGATCAGATACCGTCGTAGTCCAGACTATAAACTATGTCGACCAGGGATCGGTTA
AA--AC-TTTTTAAAAGTTTAATCGGCACCTT-GTGAGAAATCAC-GAGTGTTTAGATTC
CGGGGGGAGTATGGTCGCAAGTCTGAAACTTAAAGGAATTGACGGAAGGGCACACAATGG
AGTGGAG-CCTGCGGCTTAATTT-GACTCAACTCGGGAAA-ACTTACCAAGCTAAGATAT
AGTAAGGATTGACAGA-CTAA-GAGATCTTTCATGATTCTATAAGTGGTGGTGCATGGTC
GTTCTTAG-TTGGTGGAGCGATTTGTCTGGTCAATTCCGATAACGGACGAGACCTCGACC
TGCTAACTAGTAGTATTTATTG-GCCGTTATGGATGATAGCTATT-CGGGGTTTGG-AAG
GGTT----TC--GGCTCTC----TCC--GCTTCGTGTGG----TGTGTAATCTGGTCGGA
TAG--GTACGAATTA------AA---A--CTTCTTAGAGGGACTACCT-GCCTCAAGCAG
GCGG-AAGTCCGAGGCAATAACAGGTCTGTGATGCCCTTAGATACCTT-GGGCCGCACGC
GCGCTACAATGTAGAAAGCAAAAAGGT-----TCC--------TGGTCCGG-AAG---GA
TT-GGGTAATCAATTGAATTTTCTACGTAACTGGGATTG-ATCTTTGTAATTATTGATCA
TCAACGAGGAATTCCTTGTAAGCGTAAGTCATTACCTTATGCTGAATATGTCCCTGCCCT
TTGTACACACCGCCCGTCGCTCCTACCGATCGAATGATACGGTAAAGCCAACGGATGA--
--------G-TC--CTATTGGG-C-AAC-CAATATG------GATT-TTA-AAAG--TTG
-TT-TAAATCT--C----ATT--GTT-
>GQ496156.1_Polysphondylium_patagonicum
TCTAAG-TATAAATTCTTG-TACGATGAAA----CTGCAGACGGCTCATT--ACAA--CA
---GT----GATAAACTA-ATAGACTTTCGG-GTTTT------ACCT-TTTGGATAACCG
CAGTAAATCGGGGCTAATACATACAAG-CG-AGGGG-TGACTGATT---TATC-G-G-AA
GCTCCGCGATTATT----A-GCA-TT--CAA-CCAATAC--CCGCAAGGGTTCTGTTGGT
GAAACCGAATAATATTGCAGATCGAAG--------ATT-TATCTTCGACAAGTCTACTGT
GTCACTGCCCTATCAACTTTCGATGGTACGGTATTGGCCTACCATGGTTGTAACGGGTAA
CGGGGAATTAGGGTTCGATTCCGGAGAGGGAGCCTGAGAAATGGCTACCACTTCTACGGA
AGGCAGCAGGCGCGCAAATT-ACTCAATCCCAATAC-GGGGAAGTAGTGACAATAAATAT
CGATGCCTATCCATTT--TGGAAGG--GTAATTGAAATGAACACAAATTAAAACTCTTAA
TT-AATACAATTGGAGGGCAAGTCTGGTGCCAGCAGCCGCGGTAATTCCAGCTCCAA-TA
GCATATACTAAAGTTGTTGCAGTTAAAA-AGC-TCGTAGTTGAAGTTAAAGCTTTATTTG
GG-TTAA--------AAATCCATTTT-GCCTATA-----------CGGTTTAATA---G

GATTCCAGTATTGCTTTTT-T-------AAAG-TTCAGTTTGTATTGCCTT-------TG
GTAGTATTTATTTG---------------GACATTTCACTGTGAGAAAA-TTGTGGTGTT
TAAAGCA--GG-C---GTC---TCTGTCTGATCTTTTGCAGCATGGTATGATGGAACATG
ACAT---TTTGCGC--AATTGGTT---GCG----ATTAAAGTGTAATGATTAATAGGGAT
GGATGGGGGTGTTCATATTGGTGGGCGAGAGGTGAAATTCGTTGACCCTATCAAGATGAA
CTTCTGCGAAAGCATTCACCAAATACTTCCCCATTAATCAAGAACGAAAGTTTGGGGATC
GAAGACGATCAGATACCGTCGTAGTCCAAACTATAAACTATGTCGACCAGGGATCGGTTA
AA--AC-TTTTTAAAAGTTTAATCGGCACCTT-GTGAGAAATCAC-GAGTGTTTAGATTC
CGGGGGGAGTATGGTCGCAAGTCTGAAACTTAAAGGAATTGACGGAAGGGCACACAATGG
AGTGGAG-CCTGCGGCTTAATTT-GACTCAACTCGGGAAA-ACTTACCAAGCTAAGATAT
AGTAAGGATTGACAGA-CTAA-GAGATCTTTCATGATTCTATAAGTGGTGGTGCATGGTC
GTTCTTAG-TTGGTGGAGCGATTTGTCTGGTCAATTCCGATAACGGACGAGACCTCGACC
TGCTAACTAGTAGTATTTATTG-GCCGTTATGGATGATAGCTATT-CGGGGTTTGG-AAG
GGTT----TC--GGCTCTC----TCC--GCTTCGTGTGG----TGTGTAATCTGGTCGGA
TAG--GTACGAATTA------AA---A--CTTCTTAGAGGGACTACCT-GCCTCAAGCAG
GCGG-AAGTCCGAGGCAATAACAGGTCTGTGATGCCCTTAGATACCTT-GGGCCGCACGC
GCGCTACAATGTAGAAAGCAAAAAGGT-----TCC--------TGGTCCGG-AAG---GA
TT-GGGTAATCAATTGAATTTTCTACGTAACTGGGATTG-ATCTTTGTAATTATTGATCA
TCAACGAGGAATTCCTTGTAAGCGTAAGTCATTACCTTATGCTGAATATGTCCCTGCCCT
TTGTACACACCGCCCGTCGCTCCTACCGATCGAATGATACGGTAAAGCCAACGGATAA--
--------GATC--CTATTGGG-C-AAC-TAATATG------GATT-TTA-AAAG--TTG
-TT-TAAATCT--C----ATT--GTT-
>AM168041.1_Dictyostelium_firmibasis_TNS_C_14
TCTAAG-TATAAATTCTTG-TACGATGAAA----CTGCAGACGGCTCATT--ACAA--CA
---GT----GATAAACTA-ATAGACTTTCGG-GTTTT------ACCTTTT-GGATAACCG
CAGTAAATCGGGGCTAATACATACAAG-CG-ATGGG-CGACTGGC-----AAC-G-G-AA
GCTCAGCGATTATT----A-GCA-TT--CTA-CCAATGC--CTTC--GGGTTTTG--GGT
GAGACCGAATAATATTGCAGATCGAGG--------ATT-TATCTTCGACAAGTCTACTGT
GTCACTGCCCTATCAACTTTCGATGGTACGGTATTGGCCTACCATGGTTGTAACGGGTAA
CGGGGAATTAGGGTTCGATTCCGGAGAGGGAGCCTGAGAAATGGCTACCACTTCTACGGA
AGGCAGCAGGCGCGCAAATT-ACTCAATCCCAATAC-GGGGAAGTAGTGACAATAAATAT
CAATGCTTATCC--TT-TTTGGAGG--GCAATTGAAATGAACACAAATTAAAACTCTTAA
TT-AACACAATTGGAGGGCAAGTCTGGTGCCAGCAGCCGCGGTAATTCCAGCTCCAA-TA
GCATATACTAAAGTTGTTGCAGTTAAAA-AGC-TCGTAGTTGAAGTTAAAGGTTTATT-G
GG-TAAA----------CGTCGTTTG-TCACTTC----------G-TGATTAAAC----T
GACTCCAGTATCTCTTT-C-TT-----AATAG-TTCAGCTTTCGTTATCTT-------TG
ATAGCGGTTGTTTG---------------GACATTTCACTGTGAGAAAA-TTGTGGTGTT
TAAAGCA--GG-C---GTC---TC-GCCTGATCTTTTGCAGCATGGTATGATGAAACATG
ACAT---TTTACGC--TATTGGTTT--GCG----TCTAAAGTGTAATGATTAATAGGGAT
GGATGGGGGTGTTCATATTGGTGGGCGAGAGGTGAAATTCGTTGACCCTATCAAGATGAA
CTTCTGCGAAAGCATTCACCAAATACTTCCCCATTAATCAAGAACGAAAGTTTGGGGATC
GAAGACGATCAGATACCGTCGTAGTCCAAACTATAAACTATGTCGACCAGGGATCGGTTA
AA--AT-TTTTTTAAAATTTAATCGGCACCTT-GTGAGAAATCAC-GAGTGTTTAGATTC
CGGGGGGAGTATGGTCGCAAGTCTGAAACTTAAAGGAATTGACGGAAGGGCACACAATGG
AGTGGAG-CCTGCGGCTTAATTT-GACTCAACTCGGGAAA-ACTTACCAAGCTAAGATAT
AGTAAGGATTGACAGA-CTAA-AAGATCTTTCATGATTCTATAAGTGGTGGTGCATGGTC
GTTCTTAG-TTGGTGGAGCGATTTGTCTGGTCAATTCCGATAACGGACGAGACCTCGACC
TGCTAACTAGTAGTATTTATTA-GTCGATATAGGCGATAGCTTTTCTGGGGTTTGG-AAT
GATT----TC--GGTCATC----TCC-GGCTTCAAGGAG----TGTGTAGTCTGACTCGA
TAG--GTACGAATTT---AAAAA------CTTCTTAGAGGGACTACCT-GCCTCAAGCAG
GCGG-AAGTCCGAGGCAATAACAGGTCTGTGATGCCCTTAGATACCTT-GGGCCGCACGC
GCGCTACAATGTAGGAAACAAAAAGGC-----TCC--------TGGTCCGG-AAG---GA
TT-GGGTAATCATTTGAATTTCCTACGTAACTGGGATTG-ATCTTTGTAATTATTGATCA
TAAACGAGGAATTCCTTGTAAGCATAAGTCATTACCTTATGCTGAATATGTCCCTGCCCT
TTGTACACACCGCCCGTCGCTCCTACCGATCGAATGATACGGTAAAGTTAACGGATCG--
--------TTTT--ATCTGTGG-C-AAC-ACTGATA------TAAA-TTA-AAAG--TTA
-TT-TAAATCT--C----ATT--GTTT
>AM168071.1_Dictyostelium_discoideum_NC4
TCTAAG-TATAAATTCTTG-TACGATGAAA----CTGCAGACGGCTCATT--ACAA--CA
---GT----GATAAACTA-ATAGACTTTCGG-GTTTT------ACCTTTT-GGATAACCG
CAGTAAATCGGGGCTAATACATACAAG-CG-ATGGG-TGACTGGC-----AAC-G-G-AA
GCTCAGCGATTATT----A-GCA-TT--CTA-CCAATGC--CTTC--GGGTTTTG--GGT
GATACCGAATAATATTGCAGATCGAGG--------ATT-TATCTTCGACAAGTCTACTGT
GTCACTGCCCTATCAACTTTCGATGGTACGGTATTGGCCTACCATGGTTGTAACGGGTAA
CGGGGAATTAGGGTTCGATTCCGGAGAGGGAGCCTGAGAAATGGCTACCACTTCTACGGA
AGGCAGCAGGCGCGCAAATT-ACTCAATCCCAATAC-GGGGAAGTAGTGACAATAAATAT
CAATACCTATCC--TT-TTTGGAGG--GCAATTGAAATGAACACAAATTAAAACTCTTAA
TT-AACACAATTGGAGGGCAAGTCTGGTGCCAGCAGCCGCGGTAATTCCAGCTCCAA-TA
GCATATACTAAAGTTGTTGCAGTTAAAA-AGC-TCGTAGTTGAAGTTTAAGGTTTACC-G
GG-TTTA----------TGTCATTTA-CCACTTC----------G-TGGTTAAAT----C
GACACCGGTATCTCTTT-C-TT-----AATAG-TTCAGCTTGTATTATCTT-------TG
ATAGTGCTTGTTTG---------------GACATTTCACTGTGAGAAAA-TTGTGGTGTT
TAAAGCA--GG-C---GTC---TC-GCCTGATCTTTTGCAGCATGGTATGATGAAACATG

ACAT---TTTACGC--TATTGGTTT--GCG----TTTAAAGTGTAATGATTAATAGGGAT
GGATGGGGGTGTTCATATTGGTGGGCGAGAGGTGAAATTCGTTGACCCTATCAAGATGAA
CTTCTGCGAAAGCATTCACCAAATACTTCCCCATTAATCAAGAACGAAAGTTTGGGGATC
GAAGACGATCAGATACCGTCGTAGTCCAAACTATAAACTATGTCGACCAGGGATCGGTTA
AA--AT-TTTTTCAAAATTTAATCGGCACCTT-GTGAGAAATCAT-GAGTGTTTAGATTC
CGGGGGGAGTATGGTCGCAAGTCTGAAACTTAAAGGAATTGACGGAAGGGCACACAATGG
AGTGGAG-CCTGCGGCTTAATTT-GACTCAACTCGGGAAA-ACTTACCAAGCTAAGATAT
AGTAAGGATTGACAGA-CTAA-AAGATCTTTCATGATTCTATAAGTGGTGGTGCATGGTC
GTTCTTAG-TTGGTGGAGCGATTTGTCTGGTCAATTCCGATAACGGACGAGACCTCGACC
TGCTAACTAGTAGTATTTATTA-GTCGATATAGACGATAGCTTTTCTGGGGTTTGG-AAT
GATT----TC--GGTCATC----TCC-TGCTTCAAGGAG----TGTGTAGTCTGACTCGA
TAG--GTACGAATTA---A--AA------CTTCTTAGAGGGACTACCT-GCCTCAAGCAG
GCGG-AAGTCCGAGGCAATAACAGGTCTGTGATGCCCTTAGATACCTT-GGGCCGCACGC
GCGCTACAATGTAGGAAACAAAAAGGC-----TCC--------TGGTCCGG-AAG---GA
TT-GGGTAATCATTTGAATTTCCTACGTAACTGGGCTTG-ATCTTTGTAATTATTGATCA
TAAACGAGGAATTCCTTGTAAGCGTAAGTCATTACCTTATGCTGAATATGTCCCTGCCCT
TTGTACACACCGCCCGTCGCTCCTACCGATCGAATGATACGGTAAAGTTAACGGATCG--
--------TTTT--ATCTGTGG-C-AAC-ACTGATA------TAAA-TTA-AAAG--TTA
-TT-TAAATCT--C----ATT--GTTT
>AM168038.1_Dictyostelium_dimigraformum_AR5b
TCTAAG-TATAAATTCTTG-TACGATGAAA----CTGCAGACGGCTCATT--ACAA--CA
---GT----GATAAACTA-ATAGACTTTCGG-GTTTT------ACCTTTT-GGATAACCG
CAGTAAATCGGGGCTAATACATACAAG-CG-ATGGG-TGACTGGC-----AAC-G-G-AA
GCTCAGCGATTATT----A-GCA-TT--CTA-CCAATAC--CTTC--GGGTTTTG--GGT
GAGACCGAATAATATTGCAGATCGAGG--------ATT-TATCTTCGACAAGTCTACTGT
GTCACTGCCCTATCAACTTTCGATGGTACGGTATTGGCCTACCATGGTTGTAACGGGTAA
CGGGGAATTAGGGTTCGATTCCGGAGAGGGAGCCTGAGAAATGGCTACCACTTCTACGGA
AGGCAGCAGGCGCGCAAATT-ACTCAATCCCAATAC-GGGGAAGTAGTGACAATAAATAT
CAATACCTATCC--TT-TTTGGAGG--GCAATTGAAATGAACACAAATTAGAACTCTTAA
TT-AACACAATTGGAGGGCAAGTCTGGTGCCAGCAGCCGCGGTAATTCCAGCTCCAA-TA
GCATATACTAAAGTTGTTGCAGTTAAAA-AGC-TCGTAGTTGAAGTTTAAGGTTTACT-G
GG-TTTT---------ATGTGA-TTG-TCACTTC----------G-TGATTAA------T
CACACCAGTATCTCTTT-C-TT-----AATAG-TTCAGCTTGTATTATCTT-------TG
ATAGTGCTTGTTTG---------------GACATTTCACTGTGAGAAAA-TTGTGGTGTT
TAAAGCA--GG-C---GTC---TC-GCCTGATCTTTTGCAGCATGGTATGATGAAACATG
ACAT---TTTACGC--TATTGGTTT--GCG----TTTAAAGTGTAATGATTAATAGGGAT
GGATGGGGGTGTTCATATTGGTGGGCGAGAGGTGAAATTCCTTGACCCTATCAAGATGAA
CTTCTGCGAAAGCATTCACCAAATACTTCCCCATTAATCAAGAACGAAAGTTTGGGGATC
GAAGACGATCAGATACCGTCGTAGTCCAAACTATAAACTATGTCGACCAGGGATCGGTTA
AA--AT-TTTTATAAAATTTAATCGGCACCTT-GTGAGAAATCAT-GAGTGTTTAGATTC
CGGGGGGAGTATGGTCGCAAGTCTGAAACTTAAAGGAATTGACGGAAGGGCACACAATGG
AGTGGAG-CCTGCGGCTTAATTT-GACTCAACTCGGGAAA-ACTTACCAAGCTAAGATAT
AGTAAGGATTGACAGA-CTAA-AAGATCTTTCATGATTCTATAAGTGGTGGTGCATGGTC
GTTCTTAG-TTGGTGGAGCGATTTGTCTGGTCAATTCCGATAACGGACGAGACCTCGACC
TGCTAACTAGTAGTATTTATTA-GTCGATATGGACGATAGCTTTTCTGGGGTTTGG-AAT
GATT----TC--GGTCATC----TCC-TGCTTCAAGGAG----TGTGTAGTCTGACTCGA
TAG--GTACGAATTA---A--AA------CTTCTTAGAGGGACTACCT-GCCTCAAGCAG
GCGG-AAGTCCGAGGCAATAACAGGTCTGTGATGCCCTTAGATACCTT-GGGCCGCACGC
GCGCTACAATGTAGGAAACAAAAAGGC-----TCC--------TGGTCCGG-AAG---GA
TT-GGGTAATCATTTGAATTTCCTACGTAACTGGGCTTG-ATCTTTGTAATTATTGATCA
TAAACGAGGAATTCCTTGTAAGCGTAAGTCATTACCTTATGCTGAATATGTCCCTGCCCT
TTGTACACACCGCCCGTCGCTCCTACCGATCGAATGATACGGTAAAGTTAACGGATCG--
--------TTTT--ATCTGTGG-C-AAC-ACTGATA------TAAA-TTA-AAAG--TTA
-TT-TAAATCT--C----ATT--GTTT
>AM168033.1_Dictyostelium_citrinum_OH494
TCTAAG-TATAAATTCTTG-TACGATGAAA----CTGCAGACGGCTCATT--ACAA--CA
---GT----GATAAACTA-ATAGACTTTCGG-GTTTT------ACCTTTT-GGATAACCG
CAGTAAATCGGGGCTAATACATACAAG-CG-ATGGG-TGACTGGC-----AAC-G-G-AA
GCTCAGCGATTATT----A-GCG-TT--CTA-CCAATAC--CTTC--GGGTTTTG--GGT
GAGACCGAATAATATTGCAGATCGAGG--------ATT-TATCTTCGACAAGTCTACTGT
GTCACTGCCCTATCAACTTTCGATGGTACGGTATTGGCCTACCATGGTTGTAACGGGTAA
CGGGGAATTAGGGTTCGATTCCGGAGAGGGAGCCTGAGAAATGGCTACCACTTCTACGGA
AGGCAGCAGGCGCGCAAATT-ACTCAATCCCAATAC-GGGGAAGTAGTGACAATAAATAT
CAATACCTATCC--TT-TTTGGAGG--GCAATTGAAATGAACACAAATTAAAACTCTTAA
TT-AACACAATTGGAGGGCAAGTCTGGTGCCAGCAGCCGCGGTAATTCCAGCTCCAA-TA
GCATATACTAAAGTTGTTGCAGTTAAAA-AGC-TCGTAGTTGAAGTTTAAGGTTTACT-G
GG-TTTA----------TGTCGTTTG-TCACTTTT---------G-TGGCCAAAC----T
GACACCAGTATCTCTTT-C-TT-----AATAG-TTCAGCTTGCATTATCTT-------TG
ATAGTGCTTGTTTG---------------GACATTTCACTGTGAGAAAA-TTGTGGTGTT
TAAAGCA--GG-C---GTC---TC-GCCTGATCTTTTGCAGCATGGTATGATGAAACATG
ACAT---TTTACGC--TATTGGTTT--GCG----TTTAAAGTGTAATGATTAATAGGGAT
GGATGGGGGTGTTCATATTGGTGGGCGAGAGGTGAAATTCGTTGACCCTATCAAGATGAA
CTTCTGCGAAAGCATTCACCAAATACTTCCCCATTAATCAAGAACGAAAGTTTGGGGATC

GAAGACGATCAGACACCGTCGTAGTCCAAACTATAAACTATGTCGACCAGGGATCGGTTA
AA--AT-TTTTTTAAAATTTAATCGGCACCTT-GTGAGAAATCAT-GAGTGTTTAGATTC
CGGGGGGAGTATGGTCGCAAGTCTGAAACTTAAAGGAATTGACGGAAGGGCACACAATGG
AGTGGAG-CCTGCGGCTTAATTT-GACTCAACTCGGGAAA-ACTTACCAAGCTAAGATAT
AGTAAGGATTGACAGA-CTAA-AAGATCTTTCATGATTCTATAAGTGGTGGTGCATGGTC
GTTCTTAG-TTGGTGGAGCGATTTGTCTGGTCAATTCCGATAACGGACGAGACCTCGACC
TGCTAACTAGTAGTATTTATTA-GTCGATATGGACGATAGCTTCTCTGGGGTTTGG-AAT
GATT----TC--GGTCATC----TCC-TGCTTCAAGGAG----TGTGTAGTCTGACTTGA
TAG--GTACGAATCA---A--AA------CTTCTTAGAGGGACTACCT-GCCTCAAGCAG
GCGG-AAGTCCGAGGCAATAACAGGTCTGTGATGCCCTTAAATACCTT-GGGCCGCACGC
GCGCTACAATGTAGGAAACAAAAAGGC-----TCC--------TGGTCCGG-AAG---GA
TT-GGGTAATCATTTGAATTTCCTACGTAACTGGGCTTG-ATCTTTGTAATTATTGATCA
TAAACGAGGAATTCCTTGTAAGCGTAAGTCATTACCTTATGCTGAATATGTCCCTGCCCT
TTGTACACACCGCCCGTCGCTCCTACCGATCGAATGATACGGTAAAGTTAACGGATCG--
--------TTTT--ATCTGTGG-C-AAC-ACTGATA------TAAA-TTA-AAAG--TTA
-TT-TAAATCT--C----ATT--GTTT
>AM168039.1_Dictyostelium_discoideum_V34
TCTAAG-TATAAATTCTTG-TACGATGAAA----CTGCAGACGGCTCATT--ACAA--CA
---GT----GATAAACTA-ATAGACTTTCGG-GTTTT------ACCTTTT-GGATAACCG
CAGTAAATCGGGGCTAATACATACAAG-CG-ATGGG-TGACTGGC-----AAC-G-G-AA
GCTCAGCGATTATT----A-GCA-TT--CTA-CCAATGC--CTTC--GGGTTTTG--GGT
GAGACCGAATAATATTGCAGATCGAGG--------ATT-TATCTTCGACAAGTCTACTGT
GTCACTGCCCTATCAACTTTCGATGGTACGGTATTGGCCTACCATGGTTGTAACGGGTAA
CGGGGAATTAGGGTTCGATTCCGGAGAGGGAGCCTGAGAAATGGCTACCACTTCTACGGA
AGGCAGCAGGCGCGCAAATT-ACTCAATCCCAATAC-GGGGAAGTAGTGACAATAAATAT
CAATACCTATCC--TT-TTTGGAGG--GCAATTGAAATGAACACAAATTAAAACTCTTAA
TT-AACACAATTGGAGGGCAAGTCTGGTGCCAGCAGCCGCGGTAATTCCAGCTCCAA-TA
GCATATACTAAAGTTGTTGCAGTTAAAA-AGC-TCGTAGTTGAAGTTTAAGGTTTACT-G
GG-TTTA----------TGTCGTTTG-TCACTTTT---------G-TGGT-AAAC----T
GACACCAGTATCTCTTT-C-TT-----AATAG-TTCAGCTTACATTATCTT-------TG
ATAGTGTTTGTTTG---------------GACATTTCACTGTGAGAAAA-TTGTGGTGTT
TAAAGCA--GG-C---GTC---TC-GCCTGATCTTTTGCAGCATGGTATGATGAAACATG
ACAT---TTTACGC--TATTGGTTT--GCG----TTTAAAGTGTAATGATTAATAGGGAT
GGATGGGGGTGTTCATATTGGTGGGCGAGAGGTGAAATTCGTTGACCCTATCAAGATGAA
CTTCTGCGAAAGCATTCACCAAATACTTCCCCATTAATCAAGAACGAAAGTTTGGGGATC
GAAGACGATCAGATACCGTCGTAGTCCAAACTATAAACTATGTCGACCAGGGATCGGTTA
AA--AT-TTTTTTAAAATTTAATCGGCACCTT-GTGAGAAATCAT-GAGTGTTTAGATTC
CGGGGGGAGTATGGTCGCAAGTCTGAAACTTAAAGGAATTGACGGAAGGGCACACAATGG
AGTGGAG-CCTGCGGCTTAATTT-GACTCAACTCGGGAAA-ACTTACCAAGCTAAGATAT
AGTAAGGATTGACAGA-CTAA-AAGATCTTTCATGATTCTATAAGTGGTGGTGCATGGTC
GTTCTTAG-TTGGTGGAGCGATTTGTCTGGTCAATTCCGATAACGGACGAGACCTCGACC
TGCTAACTAGTAGTATTTATTA-GTCGATATGGACGATAGCTTCTCTGGGGTTTGG-AAT
GATT----TC--GGTCATC----TCC-TGCTTCAAGGAG----TGTGTAGTCTGACTTGA
TAG--GTACGAATTA---A--AA------CTTCTTAGAGGGACTACCT-GCCTCAAGCAG
GCGG-AAGTCCGAGGCAATAACAGGTCTGTGATGCCCTTAGATACCTT-GGGCCGCACGC
GCGCTACAATGTAGGAAACAAAAAGGC-----TCC--------TGGTCCGG-AAG---GA
TT-GGGTAATCATTTGAATTTCCTACGTAACTGGGCTTG-ATCTTTGTAATTATTGATCA
TAAACGAGGAATTCCTTGTAAGCGTAAGTCATTACCTTATGCTGAATATGTCCCTGCCCT
TTGTACACACCGCCCGTCGCTCCTACCGATCGAATGATACGGTAAAGTTAACGGATCG--
--------TTTT--ATCTGTGG-C-AAC-ACTGATA------TAAA-TTA-AAAG--TTA
-TT-TAAATCT--C----ATT--GTTT
>AM168044.1_Dictyostelium_intermedium_PJ11
TCTAAG-TATAAATTCTTG-TACGATGAAA----CTGCAGACGGCTCATT--ACAA--CA
---GT----GATAAACTA-ATAGACTTTCGG-GTTTT------ACCTTTT-GGATAACCC
CAGTAAATCGGGGCTAATACATACAAG-CG-ATGGG-TGACTGGT-----AAC-G-G-AA
GCTCAGCGATTATT----A-GCA-TT--CTA-CCAATAC--CTTC--GGGTTTTG--GGT
GAGACCGAATAATATTGCAGATCGAGG--------ATT-TATCTTCGACAAGTCTACTGT
GTCACTGCCCTATCAACTTTCGATGGTACGGTATTGGCCTACCATGGTTGTAACGGGTAA
CGGGGAATTAGGGTTCGATTCCGGAGAGGGAGCCTGAGAAATGGCTACCACTTCTACGGA
AGGCAGCAGGCGCGCAAATT-ACTCAATCCCAATAC-GGGGAAGTAGCGACAATAAATAT
CAATACCTATCC--TT-TTTGGAGG--GCAATTGAAATGAACACAAATTAAAACTCTTAA
TT-AACACAATTGGAGGGCAAGTCTGGTGCCAGCAGCCGCGGTAATTCCAGCTCCAA-TA
GCATATACTAAAGTTGTTGCAGTTAAAA-AGC-TCGTAGTTGAAGTTAAAGGTTTACC-G
GG-TTTA----------CGTCATTTG-TCACTTC----------G-TGGCTAAAT----C
GACTCCGGTATCTCTTT-C-TT-----AATAG-TTCAGCTTTCATTATCTT-------TG
ATAGTGGTTGTTTG---------------GACATTTCACTGTGAGAAAA-TTGTGGTGTT
TAAAGCA--GG-C---GTC---TC-GCCTGATCTTTTGCAGCATGGTATGATGAAACATG
ACAT---TTTACGT--TATTGGTTT--GCG----TCTAAAGTGTAATGATTAATAGGGAT
GGATGGGGGTGTTCATATTGGTGGGCGAGAGGTGAAATTCGTTGACCCTATCAAGATGAA
CTTCTGCGAAAGCATTCACCAAATACTTCCCCATTAATCAAGAACGAAAGTTTGGGGATC
GAAGACGATCAGATACCGTCGTAGTCCAAACTATAAACTATGTCGACCAGGGATCGGTTA
AA--AT-TTTTTTAAAATTTAATCGGCACCTT-GTGAGAAATCAC-GAGTGTTTAGATTC
CGGGGGGAGTATGGTCGCAAGTCTGAAACTTAAAGGAATTGACGGAAGGGCACACAATGG

AGTGGAG-CCTGCGGCTTAATTT-GACTCAACTCGGGAAA-ACTTACCAAGCTAAGATAT
AGTAAGGATTGACAGA-CTAA-AAGATCTTTCATGATTCTATAAGTGGTGGTGCATGGTC
GTTCTTAG-TTGGTGGAGCGATTTGTCTGGTCAATTCCGATAACGGACGAGACCTCGACC
TGCTAACTAGTAGTATTTATTA-GTCGATATGGACGATAGCTTTTCTGGGGTTTGG-AAT
GATT----TC--GGTCATC----TCC-TGCTTCAAGGAG----TGTGTAGTCTGACTCGA
TAG--GTACGAATTC---AAAAA------CTTCTTAGAGGGACTACCT-GCCTCAAGCAG
GCGG-AAGTCCGAGGCAATAACAGGTCTGTGATGCCCTTAGATACCTT-GGGCCGCACGC
GCGCTACAATGTAGGAAACAAAAAGGC-----TCC--------TGGTCCGG-AAG---GA
TT-GGGTAATCATTTGAATTTCCTACGTAACTGGGATTG-ATCTTTGTAATTATTGATCA
TAAACGAGGAATTCCTTGTAAGCATAAGTCATTACCTTATGCTGAATATGTCCCTGCCCT
TTGTACACACCGCCCGTCGCTCCTACCGATCGAATGATACGGTAAAGTTAACGGATCG--
--------TTTT--ATCTGTGG-C-AAC-ACTGATA------TAAA-TTA-AAAG--TTA
-TT-TAAATCT--C----ATT--GTTT
>HQ141484.1_Dictyostelium_sp._Laos5_Laos5
TCTAAG-TATAAATTCTTG-TACGATGAAA----CTGCAGACGGCTCATT--ACAA--CA
---GT----GATAAACTA-ATAGACTTTCGG-GTTTT------ACCTTTT-GGATAACCG
CAGTAAATCGGGGCTAATACATACAAG-CG-ATGGG-TGACTGGC-----AAC-G-G-AA
GCTCAGCGATTATT----A-GCA-TT--CTA-CCAATAC--CTTC--GGGTTTTG--GGT
GAGACCGAATAATATTGCAGATCGAGG--------ATT-TATCTTCGACAAGTCTACTGT
GTCACTGCCCTATCAACTTTCGATGGTACGGTATTGGCCTACCATGGTTGTAACGGGTAA
CGGGGAATTAGGGTTCGATTCCGGAGAGGGAGCCTGAGAAATGGCTACCACTTCTACGGA
AGGCAGCAGGCGCGCAAATT-ACTCAATCCCAATAC-GGGGAAGTAGTGACAATAAATAT
CAATACCTATCC--TT-TTTGGAGG--GCAATTGAAATGAACACAAATTAAAACTCTTAA
TT-AACACAATTGGAGGGCAAGTCTGGTGCCAGCAGCCGCGGTAATTCCAGCTCCAA-TA
GCATATACTAAAGTTGTTGCAGTTAAAA-AGC-TCGTAGTTGAAGTTAAAGGTTTACT-G
GG-TAAA----------CGTCATTTG-TCACTTC----------G-TGGCTAAAT----C
GACTCCAGTATCTCTTT-C-TT-----AATAG-TTCAGCTTTCGTTATCTT-------TG
ATAGCGGTTGTTTG---------------GACATTTCACTGTGAGAAAA-TTGTGGTGTT
TAAAGCA--GG-C---GTC---TC-GCCTGATCTTTTGCAGCATGGTATGATGAAACATG
ACAT---TTTACGC--TATTGGTTT--GCG----TCTAAAGTGTAATGATTAATAGGGAT
GGATGGGGGTGTTCATATTGGTGGGCGAGAGGTGAAATTCGTTGACCCTATCAAGATGAA
CTTCTGCGAAAGCATTCACCAAATACTTCCCCATTAATCAAGAACGAAAGTTTGGGGATC
GAAGACGATCAGATACCGTCGTAGTCCAAACTATAAACTATGTCGACCAGGGATCGGTTA
AA--AT-TTTTTTAAAATTTAATCGGCACCTT-GTGAGAAATCAC-GAGTGTTTAGATTC
CGGGGGGAGTATGGTCGCAAGTCTGAAACTTAAAGGAATTGACGGAAGGGCACACAATGG
AGTGGAG-CCTGCGGCTTAATTT-GACTCAACTCGGGAAA-ACTTACCAAGCTAAGATAT
AGTAAGGATTGACAGA-CTAA-AAGATCTTTCATGATTCTATAAGTGGTGGTGCATGGTC
GTTCTTAG-TTGGTGGAGCGATTTGTCTGGTCAATTCCGATAACGGACGAGACCTCGACC
TGCTAACTAGTAGTATTTATTA-GTCGATATGGGCGATAGCTTTTCTGGGGTTTGG-AAT
GATT----TC--GGTCATC----TCC-TGCTTCAAGGAG----TGTGTAGTCTGACTCGA
TAG--GTACGATTTC------AA------CTTCTTAGAGGGACTACCT-GCCTCAAGCAG
GCGG-AAGTCCGAGGCAATAACAGGTCTGTGATGCCCTTAGATACCTT-GGGCCGCACGC
GCGCTACAATGTAGGAAACAAAAAGGC-----TCC--------TGGTCCGG-AAG---GA
TT-GGGTAATCATTTGAATTTCCTACGTAACTGGGATTG-ATCTTTGTAATTATTGATCA
TAAACGAGGAATTCCTTGTAAGCGTAAGTCATTACCTTATGCTGAATATGTCCCTGCCCT
TTGTACACACCGCCCGTCGCTCCTACCGATCGAATGATACGGTAAAGTTAACGGATCG--
--------TTTT--ATCTGTGG-C-AAC-ACTGATA------TAAA-TTA-AAAG--TTA
-TT-TAAATCT--C----ATT--GTTT
>MH280023.1_Dictyostelium_purpureum_var._pseudosessile_MR273_4446
---------TAAATTCTTG-TACGATGAAA----CTGCAGACGGCTCATT--ACAA--CA
---GT----GATAAACTG-CTAGACTTTCGG-GTTTT-----AACCTTTT-GGATAACCG
CAGTAAATCGGGGCTAATACATACAAG-CG-ATGGG-TGACTGGC-----AAC-G-G-AA
GCTCAGCGATTATT----A-GCG-TTA-CTA-CCAATAC--CTTC--GGGTCTTGT-GGT
GAAACCGAATAATATTGCAGATCGGGG--------ATT-TATCTTCGACAAGTCTACTGT
GTCACTGCCCTATCAACTTTCGATGGTACGGTATTGGCCTACCATGGTTGTAACGGGTAA
CGGGGAATTAGGGTTCGATTCCGGAGAGGGAGCCTGAGAAATGGCTACCACTTCTACGGA
AGGCAGCAGGCGCGCAAATT-ACTCAATCCCAATAC-GGGGAAGTAGTGACAATAAATAT
CAATACCTATCC--TT-TTTGGAGG--GCAATTGAAATGAACACAAATTAAAACTCTTAA
TT-AACACAATTGGAGGGCAAGTCTGGTGCCAGCAGCCGCGGTAATTCCAGCTCCAA-TA
GCATATACTAAAGTTGTTGCAGTTAAAA-AGC-TCGTAGTTGAAGTTAAAGTTATATT-G
GG-CTCA----------AGTTTTTA-CCACGTCT---------G-TGGCTAAAA----T
AACTCCAATATTTCTTTTT-TT-----AATAG-CTCAGTTTCTAGG-TCTT-------TG
ACTCTAGTTATTTG---------------GGCATTTCACTGTGAGAAAA-TTGTGGTGTT
TAAAGCA--GG-C---GTC---TC-GTCTGATCTTTTGCAGCATGGTATGATGGAACATG
ACAT---TTTACGC--TATTGGTTT--GCG----TCTAAAGTGTAATGATTAATAGGGAT
GGATGGGGATGTTCATATTGGTGGGCGAGAGGTGAAATTCGTTGACCCTATCAAGATGAA
CTTCTGCGAAAGCATTCATCAAATACTTCCCCATTAATCAAGAACGAAAGTTTGGGGATC
GAAGACGATCAGATACCGTCGTAGTCCAAACTATAAACTATGTCGACCAGGGATCGGCTA
AA--AT-TTTTTTAAAATTTAGTCGGCACCTT-GTGAGAAATCAT-GAGTGTTTAGATTC
CGGGGGGAGTATGGTCGCAAGTCTGAAACTTAAAGGAATTGACGGAAGGGCACACAATGG
AGTGGAG-CCTGCGGCTTAATTT-GACTCAACTCGGGAAA-ACTTACCAAGCTAAGATAT
AGTAAGGATTGACAGA-CTAA-AAGATCTTTCATGATTCTATAAGTGGTGGTGCATGGTC
GTTCTTAG-TTGGTGGAGCGATTTGTCTGGTCAATTCCGATAACGGACGAGACCTCGACC

TGCTAACTAGTAGTATTTATTA-GCCGATATGGGCGATAGCTTCTCTGGGGTTT-G-AGT
G-CG----GT--AA-CGTA----TCTTGGCTTCAAGGAG----TGTGTAGTCTGGCTTGA
TAG--GTACGATATT---AAAAA---A--CTTCTTAGAGGGACTACCT-GCCTCAAGCAG
GCGG-AAGTCCGAGGCAATAACAGGTCTGTGATGCCCTTAGATACCTT-GGGCCGCACGC
GCGCTACAATGCAGATAGCAAAAAGGT-----TCC--------TGGCCTGG-AAA---GG
TT-GGGTAATCAATTGAATTTTCTGCGTAACTGGGATTG-ATCTTTGTAATTATTGATCA
TCAACGAGGAATTCCTTGTAAGCGTAAGTCATTACCTTATGCTGAATATGTCCCTGCCCT
TTGTACACACCGCCCGTCGCTCCTACCGATCGAATGATACGGTAAAGTTAACGGATTG--
--------TTTT---TTTGTGG-C-AAC-ACAATTA------AAA--TTA-AAAG--TTA
-TT-TAAATCT--C----ATT--GTTT
>GQ496161.1_Dictyostelium_gargantuum
TCTAAG-TATAAATTCTTG-TACGATGAAA----CTGCAGACGGCTCATT--ACAA--CA
---GT----GATAAACTA-ATAGACTTTCGG-GTTTTT----AACCTTTT-GGATAACCG
CAGTAAATCGGGGCTAATACATAGAAG-CG-ATGGG-TGACTGGC-----AAC-G-G-AA
GCTCAGCGATTATT----A-GCA-TTA-CTA-CCAATAC--CTTC--GGGTCTTGT-GGT
GAAACCGAATAATATTGCAGATCGAGG--------ATT-TATCTTCGACAAGTCTATTGT
GTCACTGCCCTATCAACTTTCGATGGTACGGTATTGGCCTACCATGGTTGTAACGGGTAA
CGGGGAATTAGGGTTCGATTCCGGAGAGGGAGCCTGAGAAATGGCTACCACTTCTACGGA
AGGCAGCAGGCGCGCAAATT-ACTCAATCCCAATAC-GGGGAAGTAGTGACAATAAATAT
CAATACCTATCC--TT-TTTGGAGG--GCAATTGAAATGAACACAAATTAAAACTCTTAA
TT-AACACAATTGGAGGGCAAGTCTGGTGCCAGCAGCCGCGGTAATTCCAGCTCCAA-TA
GCATATACTAAAGTTGTTGCAGTTAAAA-AGC-TCGTAGTTGAAGTTAAGGGTTTATC-G
GG-CTAA----------AGTTATTTG-CCACTTT----------G-TGGTTAAATT---C
AACTCCGGTATTCCTTTTC-TT-----AATAG-TTCAGCTTCTATTATCTT-------TG
ATAGTAGTTGTTTG---------------GGCATTTCACTGTGAGAAAA-TTGTGGTGTT
TAAAGCA--GG-C---GTC---TC-GCCTGATCTTTTGCAGCATGGTATGATGAAACATG
ACAT---TTTACGC--TATTGGTTT--GCG----TCTAAAGTGTAATGATTAATAGGGAT
GGATGGGGGTGTTCATATTGGTGGGCGAGAGGTGAAATTCGTTGACCCTATCAAGATGAA
CTTCTGCGAAAGCATTCACCAAATACTTCCCCATTAATCAAGAACGAAAGTTTGGGGATC
GAAGACGATCAGATACCGTCGTAGTCCAAACTATAAACTATGTCGACCAGGGATCGGTTA
AA--AT-TTTTTTAAAATTTAATCGGCACCTT-GTGAGAAATCAC-GAGTGTTTAGATTC
CGGGGGGAGTATGGTCGCAAGTCTGAAACTTAAAGGAATTGACGGAAGGGCACACAATGG
AGTGGAG-CCTGCGGCTTAATTT-GACTCAACTCGGGAAA-ACTTACCAAGCTAAGATAT
AGTAAGGATTGACAGA-CTAA-AAGATCTTTCATGATTCTATAAGTGGTGGTGCATGGTC
GTTCTTAG-TTGGTG-AGCGATTTGTCTGGTCAATTCCGATAACGGACGAGACCTCGACC
TGCTAACTAGTAGTATTTATTA-GTCGATATGGGCGATAGCTTCTCTGGGGTTTGG-AAC
GGCT----TC--GGCCGTC----TCC-TGCTTCAAGGAG----TGTGTAGTCTGACTTGA
TAG--GTACGAATTC---AAAAA------CTTCTTAGAGGGACTACCT-GCCTCAAGCAG
GCGG-AAGTCCGAGGCAATAACAGGTCTGTGATGCCCTTAGATACCTT-GGGCCGCACGC
GCGCTACAATGTATAAAACAAAAAGGT-----TCC--------TGGTCCGG-AAG---GA
TT-GGGTAATCAATTGAATTTTCTACGTAACTGGGATTG-ATCTTTGTAATTATTGATCA
TCAACGAGGAATTCCTTGTAAGCGTAAGTCATTACCTTATGCTGAATATGTCCCTGCCCT
TTGTACACACCGCCCGTCGCTCCTACCGATCGAATGATACGGTAAAGTTAACGGATCG--
--------TTTT--ATTTGTGG-C-AAC-ACAAATT------AAGA-CTA-AAAG--TTA
-TT-TAAATCT--C----ATT--GTT-
>AM168064.1_Dictyostelium_robustum_TNS_C_219
TCTAAG-TATAAATTCTTG-TACGATGAAA----CTGCAGACGGCTCATT--ACAA--CA
---GT----GATAAACTA-ATAGACTTTCGG-GTTTTT----AACCTTTT-GGATAACCG
CAGTAAATCGGGGCTAATACATAGAAG-CG-ATGGG-TGACTGGC-----AAC-G-G-AA
GCTCAGCGATTATT----A-GCA-TTA-CTA-CCAATAC--CTTC--GGGTCTTGT-GGT
GAAACCGAATAATATTGCAGATCGAGG--------ATT-TATCTTCGACAAGTCTATTGT
GTCACTGCCCTATCAACTTTCGATGGTACGGTATAGGCCCTACCATGGTTGTAACGGGTAA
CGGGGAATTAGGGTTCGATTCCGGAGAGGGAGCCTGAGAAATGGCTACCACTTCTACGGA
AGGCAGCAGGCGCGCAAATT-ACTCAATCCCAATAC-GGGGAAGTAGTGACAATAAATAT
CAATACCTATCC--TT-TTTGGAGG--GCAATTGAAATGAACACAAATTAAAACTCTTAA
TT-AACACAATTGGAGGGCAAGTCTGGTGCCAGCAGCCGCGGTAATTCCAGCTCCAA-TA
GCATATACTAAAGTCGTTGCAGTTAAAA-AGC-TCGTAGTTGAAGTTAAGGGTTTATC-G
GG-CTAA----------AGTTATTTG-CCACTTT----------G-TGGTTAAATT---C
AACTCCGGTATTCCTTTTC-TT-----AATAG-TTCAGCTTCTATTATCTT-------TG
ATAGTAGTTGTTTG---------------GGCATTTCACTGTGAGAAAA-TTGTGGTGTT
TAAAGCA--GG-C---GTC---TC-GCCTGATCTTTTGCAGCATGGTATGATGAAACATG
ACAT---TTTACGC--TATTGGTTT--GCG----TCTAAAGTGTAATGATTAATAGGGAT
GGATGGGGGTGTTCATATTGGTGGGCGAGAGGTGAAATTCGTTGACCCTATCAAGATGAA
CTTCTGCGAAAGCATTCACCAAATACTTCCCCATTAATCAAGAACGAAAGTTTGGGGATC
GAAGACGATCAGATACCGTCGTAGTCCAAACTATAAACTATGTCGACCAGGGATCGGTTA
AA--AT-TTTTTTAAAATTTAATCGGCACCTT-GTGAGAAATCAC-GAGTGTTTAGATTC
CGGGGGGAGTATGGTCGCAAGTCTGAAACTTAAAGGAATTGACGGAAGGGCACACAATGG
AGTGGAG-CCTGCGGCTTAATTT-GACTCAACTCGGGAAA-ACTTACCAAGCTAAGATAT
AGTAAGGATTGACAGA-CTAA-AAGATCTTTCATGATTCTATAAGTGGTGGTGCATGGTC
GTTCTTAG-TGGGTGGAGCGATTTGTCTGGTCAATTCCGATAACGGACGAGACCTCGACC
TGCTAACTAGTAGTATTTATTA-GTCGATATGGGCGATAGCTTCTCTGGGGTTTGG-AAC
GGCT----TC--GGCCGTC----TCC-TGCTTCAAGGAG----TGTGTAGTCTGACTTGA
TAG--GTACGAATTC---AAAAA------CTTCTTAGAGGGACTACCT-GCCTCAAGCAG

GCGG-AAGTCCGAGGCAATAACAGGTCTGTGATGCCCTTAGATACCTT-GGGCCGCACGC
GCGCTACAATGTAGAAAACAAAAAGGT-----TCC--------TGGTCCGG-AAG---GA
TT-GGGTAATCAATTGAATTTTCTACGTAACTGGGATTG-ATCTTTGTAATTATTGATCA
TCAACGAGGAATTCCTTGTAAGCGTAAGTCATTACCTTATGCTGAATATGTCCCTGCCCT
TTGTACACACCGCCCGTCGCTCCTACCGATCGAATGATACGGTAAAGTTAACGGATCG--
--------TTTT--ATTTGTGG-C-AAC-ACAAATT------AAGA-CTA-AAAG--TTA
-TT-TAAATCT--C----ATT--GTTT
>AM168031.1_Dictyostelium_brunneum_WS700
TCTAAG-TATAAATTCTTG-TACGATGAAA----CTGCAGACGGCTCATT--ACAA--CA
---GT----GATAAACTA-ATAGACTTTCGG-GTTTTT----AACCTTTT-GGATAACCG
CAGTAAATCGGGGCTAATACATAGAAG-CG-ATGGG-TGACTGGC-----AAC-G-G-AA
GCTCAGCGATTATT----A-GCA-TTA-CTA-CCAATAC--CTTC--GGGTCTTGT-GGT
GAAACCGAATAATATTGCAGATCGAGG--------ATT-TATCTTCGACAAGTCTACTGT
GTCACTGCCCTATCAACTTTCGATGGTACGGTATTGGCCTACCATGGTTGTAACGGGTAA
CGGGGAATTAGGGTTCGATTCCGGAGAGGGAGCCTGAGAAATGGCTACCACTTCTACGGA
AGGCAGCAGGCGCGCAAATT-ACTCAATCCCAATAC-GGGGAAGTAGTGACAATAAATAT
CAATACCTATCC--TT-TTTGGAGG--GCAATTGAAATGAACACAAATTAAAACTCTTAA
TT-AACACAATTGGAGGGCAAGTCTGGTGCCAGCAGCCGCGGTAATTCCAGCTCCAA-TA
GCATATACTAAAGTTGTTGCAGTTAAAA-AGC-TCGTAGTTGAAGTTAAGGGTTTATC-G
GG-CTAA----------AGTTATTTG-CCACTTT----------G-TGGTTAAATT---C
AACTCCGGTATTCCTTTTC-TT-----AATAG-TTCAGCTTCTATTATCTT-------TG
ATAGTAGTTGTTTG---------------GGCATTTCACTGTGAGAAAA-TTGTGGTGTT
TAAAGCA--GG-C---GTC---TC-GCCTGATCTTTTGCAGCATGGTATGATGAAACATG
ACAT---TTTACGC--TATTGGTTT--GCG----TCTAAAGTGTAATGATTAATAGGGAT
GGATGGGGGTGTTCATATTGGTGGGCGAGAGGTGAAATTCGTTGACCCTATCAAGATGAA
CTTCTGCGAAAGCATTCACCAAATACTTCCCCATTAATCAAGAACGAAAGTTTGGGGATC
GAAGACGATCAGATACCGTCGTAGTCCAAACTATAAACTATGTCGACCAGGGATCGGTTA
AA--AT-TTTTTTAAAATTTAATCGGCACCTT-GTGAGAAATCAC-GAGTGTTTAGATTC
CGGGGGGAGTATGGTCGCAAGTCTGAAACTTAAAGGAATTGACGGAAGGGCACACAATGG
AGTGGAG-CCTGCGGCTTAATTT-GACTCAACTCGGGAAA-ACTTACCAAGCTAAGATAT
AGTAAGGATTGACAGA-CTAA-AAGATCTTTCATGATTCTATAAGTGGTGGTGCATGGTC
GTTCTTAG-TTGGTGGAGCGATTTGTCTGGTCAATTCCGATAACGGACGAGACCTCGACC
TGCTAACTAGTAGTATTTATTA-GTCAACATAGGCGATAGCTTTTCTGGGGTTTGG-AAT
GATT----TC--GGTCATC----TCC-TGCTTCAAGGAG----TGTGTAGTCCGGCTTGA
TAG--GTACGAATTC---AAAAA------CTTCTTAGAGGGACTACCT-GCCTCAAGCAG
GCGG-AAGTCCGAGGCAATAACAGGTCTGTGATGCCCTTAGATACCTT-GGGCCGCACGC
GCGCTACAATGTAGAAAACAAAAAGGT-----TCC--------TGGTCCGG-AAG---GA
TT-GGGTAATCAATTGAATTTTCTACGTAACTGGGATTG-ATCTTTGTAATTATTGATCA
TCAACGAGGAATTCCTTGTAAGCGCAAGTCATTACCTTGTGCTGAATATGTCCCTGCCCT
TTGTACACACCGCCCGTCGCTCCTACCGATCGAATGATACGGTAAAGTTAACGGATCG--
--------TTTT--ATTTGTGG-C-AAC-ACATTAT------AAGA-CTA-AAAG--TTA
-TT-TAAATCT--C----ATT--GTTT
>AM168042.1_Dictyostelium_giganteum_WS589
TCTAAG-TATAAATTCTTG-TACGATGAAA----CTGCAGACGGCTCATT--ACAA--CA
---GT----GATAAACTA-ATAGACTTTCGG-GTTTTT----AACCTTTT-GGATAACCG
CAGTAAATCGGGGCTAATACATAGAAG-CG-ATGGG-TGACTGGC-----AAC-G-G-AA
GCTCAGCGATTATT----A-GCA-TTA-CTA-CCAATAC--CTTC--GGGTCTTGT-GGT
GAAACCGAATAATATTGCAGATCGAGG--------ATT-TATCTTCGACAAGTCTACTGT
GTCACTGCCCTATCAACTTTCGATGGTACGGTATTGGCCTACCATGGTTGTAACGGGTAA
CGGGGAATTAGGGTTCGATTCCGGAGAGGGAGCCTGAGAAATGGCTACCACTTCTACGGA
AGGCAGCAGGCGCGCAAATT-ACTCAATCCCAATAC-GGGGAAGTAGTGACAATAAATAT
CAATACCTATCC--TT-TTTGGAGG--GCAATTGAAATGAACACAAATTAAAACTCTTAA
TT-AACACAATTGGAGGGCAAGTCTGGTGCCAGCAGCCGCGGTAATTCCAGCTCCAA-TA
GCATATACTAAAGTTGTTGCAGTTAAAA-AGC-TCGTAGTTGAAGTTAAGGGTTTATC-G
GG-CTAA----------AGTTATTTG-CCACTTT----------G-TGGTTAAATT---C
AACTCCGGTATTCCTTTTC-TT-----AATAG-TTCAGCTTCCATTATCTT-------TG
ATAGTGGTTGTTTG---------------GGCATTTCACTGTGAGAAAA-TTGTGGTGTT
TAAAGCA--GG-C---GTC---TC-GCCTGATCTTTTGCAGCATGGTATGATGAAACATG
ACAT---TTTACGC--TATTGGTTT--GCG----TCTAAAGTGTAATGATTAATAGGGAT
GGATGGGGGTGTTCATATTGGTGGGCGAGAGGTGAAATTCGTTGACCCTATCAAGATGAA
CTTCTGCGAAAGCATTCACCAAATACTTCCCCATTAATCAAGAACGAAAGTTTGGGGATC
GAAGACGATCAGATACCGTCGTAGTCCAAACTATAAACTATGTCGACCAGGGATCGGTTA
AA--AT-TTTTTTAAAATTTAATCGGCACCTT-GTGAGAAATCAC-GAGTGTTTAGATTC
CGGGGGGAGTATGGTCGCAAGTCTGAAACTTAAAGGAATTGACGGAAGGGCACACAATGG
AGTGGAG-CCTGCGGCTTAATTT-GACTCAACTCGGGAAA-ACTTACCAAGCTAAGATAT
AGTAAGGATTGACAGA-CTAA-AAGATCTTTCATGATTCTATAAGTGGTGGTGCATGGTC
GTTCTTAG-TTGGTGGAGCGATTTGTCTGGTCAATTCCGATAACGGACGAGACCTCGACC
TGCTAACTAGTAGTATTTATTA-GTCGATATGGGCGATAGCTTCTCTGGGGTTTGG-AAT
GATT----TC--GGTCATC----TCC-TGCTTCAAGGAG----TGTGTAGTCTGACTTGA
TAG--GTACGAATTC---AAAAA------CTTCTTAGAGGGACTACCT-GCCTCAAGCAG
GCGG-AAGTCCGAGGCAATAACAGGTCTGTGATGCCCTTAGATACCTT-GGGCCGCACGC
GCGCTACAATGTAGAAAACAAAAAGGT-----TCC--------TGGTCCGG-AAG---GA
TT-GGGTAATCAATTGAATTTTCTACGTAACTGGGATTG-ATCTTTGTAATTATTGATCA

TCAACGAGGAATTCCTTGTAAGCGCAAGTCATTACCTTGTGCTGAATATGTCCCTGCCCT
TTGTACACACCGCCCGTCGCTCCTACCGATCGAATGATACGGTAAAGTTAACGGATCG--
--------TTTT--ATTTGTGG-C-AAC-ACATTAT------AAGA-CTA-AAAG--TTA
-TT-TAAATCT--C----ATT--GTTT
>HQ141483.1_Dictyostelium_sp._Laos1_Laos1
TCTAAG-TATAAATTCTTG-TACGATGAAA----CTGCAGA-GGCTCATT--ACAA--CA
---GT----GATAAACTA-ATAGACTTTCGG-GTTTTT----AACCTTTT-GGATAACCG
CAGTAAATCGGGGCTAATACATAGAAG-CG-ATGGG-TGACTGGC-----AAC-G-G-AA
GCTCAGCGATTATT----A-GCA-TTA-CTA-CCAATAC--CTTC--GGGTCTTGT-GGT
GAAACCGAATAATATTGCAGATCGAGG--------ATT-TATCTTCGACAAGTCTACTGT
GTCACTGCCCTATCAACTTTCGATGGTACGGTATTGGCCTACCATGGTTGTAACGGGTAA
CGGGGAATTAGGGTTCGATTCCGGAGAGGGAGCCTGAGAAATGGCTACCACTTCTACGGA
AGGCAGCAGGCGCGCAAATT-ACTCAATCCCAATAC-GGGGAAGTAGTGACAATAAATAT
CAATACCTATCC--TT-TTTGGAGG--GCAATTGAAATGAACACAAATTAAAACTCTTAA
TT-AACACAATTGGAGGGCAAGTCTGGTGCCAGCAGCCGCGGTAATTCCAGCTCCAA-TA
GCATATACTAAAGTTGTTGCAGTTAAAA-AGC-TCGTAGTTGAAGTTAAGGGTTTATC-G
GG-CTAA----------AGTTATTTG-CCACTTT----------G-TGGTTAAATT---C
AACTCCGGTATTCCTTTTC-TT-----AATAG-TTCAGCTTCCATTATCTT-------TG
ATAGTGGTTGTTTG---------------GGCATTTCACTGTGAGAAAA-TTGTGGTGTT
TAAAGCA--GG-C---GTC---TC-GCCTGATCTTTTGCAGCATGGTATGATGAAACATG
ACAT---TTTACGC--TATTGGTTT--GCG----TCTAAAGTGTAATGATTAATAGGGAT
GGATGGGGGTGTTCATATTGGTGGGCGAGAGGTGAAATTCGTTGACCCTATCAAGATGAA
CTTCTGCGAAAGCATTCACCAAATACTTCCCCATTAATCAAGAACGAAAGTTTGGGGATC
GAAGACGATCAGATACCGTCGTAGTCCAAACTATAAACTATGTCGACCAGGGATCGGTTA
AA--AT-TTTTTTAAAATTTAATCGGCACCTT-GTGAGAAATCAC-GAGTGTTTAGATTC
CGGGGGGAGTATGGTCGCAAGTCTGAAACTTAAAGGAATTGACGGAAGGGCACACAATGG
AGTGGAG-CCTGCGGCTTAATTT-GACTCAACTCGGGAAA-ACTTACCAAGCTAAGATAT
AGTAAGGATTGACAGA-CTAA-AAGATCTTTCATGATTCTATAAGTGGTGGTGCATGGTC
GTTCTTAG-TTGGTGGAGCGATTTGTCTGGTCAATTCCGATAACGGACGAGACCTCGACC
TGCTAACTAGTAGTATTTATTA-GTCGATATGGGCGATAGCTTCTCTGGGGTTTGG-AAT
GACT----TC--GGTCATC----TCC-TGCTTCAAGGAG----TGTGTAGTCTGACTTGA
TAG--GTACGAATTC---AAAAA------CTTCTTAGAGGGACTACCT-GCCTCAAGCAG
GCGG-AAGTCCGAGGCAATAACAGGTCTGTGATGCCCTTAGATACCTT-GGGCCGCACGC
GCGCTACAATGTAGAAAACAAAAAGGT-----TCC--------TGGTCCGG-AAG---GA
TT-GGGTAATCAATTGAATTTTCTACGTAACTGGGATTG-ATCTTTGTAATTATTGATCA
TCAACGAGGAATTCCTTGTAAGCGCAAGTCATTACCTTGTGCTGAATATGTCCCTGCCCT
TTGTACACACCGCCCGTCGCTCCTACCGATCGAATGATACGGTAAAGTTAACGGATCG--
--------TTTT--ATTTGTGG-C-AAC-ACATTAT------AAGA-CTA-AAAG--TTA
-TT-TAAATCT--C----ATT--GTT-
>FJ424829.1_Dictyostelium_purpureum_QSpu1
TCTAAG-TATAAATTCTTG-TACGATGAAA----CTGCAGACGGCTCATT--ACAA--CA
---GT----GATAAACTG-CTAGACTTTCGG-GTTTT-----AACCTTTT-GGATAACCG
CAGTAAATCGGGGCTAATACATACAAG-CG-ATGGG-TGACTGGC-----AAC-G-G-AA
GCTCAGCGATTATT----A-GCG-TTA-CTA-CCAATAC--CTTC--GGGTCTTGT-GGT
GAAACCGAATAATATTGCAGATCGGGG--------ATT-TATCTTCGACAAGTCTACTGT
GTCACTGCCCTATCAACTTTCGATGGTACGGTATTGGCCTACCATGGTTGTAACGGGTAA
CGGGGAATTAGGGTTCGATTCCGGAGAGGGAGCCTGAGAAATGGCTACCACTTCTACGGA
AGGCAGCAGGCGCGCAAATT-ACTCAATCCCAATAC-GGGGAAGTAGTGACAATAAATAT
CAATACCTATCC--TT-TTTGGAGG--GCAATTGAAATGAACACAAATTAAAACTCTTAA
TT-AACACAATTGGAGGGCAAGTCTGGTGCCAGCAGCCGCGGTAATTCCAGCTCCAA-TA
GCATATACTAAAGTTGTTGCAGTTAAAA-AGC-TCGTAGTTGAAGTTAAAGTTATATT-G
GG-CTCA----------AGTTTTTTA-CCACGTCT---------G-TGGCTAAAA----T
AACTCCAATATTTCTTTTT-TT-----AATAG-CTCAGTTTCTAGG-TCTT-------TG
ACTCTAGTTATTTG---------------GGCATTTCACTGTGAGAAAA-TTGTGGTGTT
TAAAGCA--GG-C---GTC---TC-GTCTGATCTTTTGCAGCATGGTATGATGGAACATG
ACAT---TTTACGC--TATTGGTTT--GCG----TCTAAAGTGTAATGATTAATAGGGAT
GGATGGGGATGTTCATATTGGTGGGCGAGAGGTGAAATTCGTTGACCCTATCAAGATGAA
CTTCTGCGAAAGCATTCATCAAATACTTCCCCATTAATCAAGAACGAAAGTTTGGGGATC
GAAGACGATCAGATACCGTCGTAGTCCAAACTATAAACTATGTCGACCAGGGATCGGCTA
AA--AT-TTTTTTAAAATTTAGTCGGCACCTT-GTGAGAAATCAT-GAGTGTTTAGATTC
CGGGGGGAGTATGGTCGCAAGTCTGAAACTTAAAGGAATTGACGGAAGGGCACACAATGG
AGTGGAG-CCTGCGGCTTAATTT-GACTCAACTCGGGAAA-ACTTACCAAGCTAAGATAT
AGTAAGGATTGACAGA-CTAA-AAGATCTTTCATGATTCTATAAGTGGTGGTGCATGGTC
GT-CTTAG-TTGGNNNNNNNNNNNNNNNNNNNNNNNNNNTCCGATAACGGACGAGACCTCGACC
TGCTAACTAGTAGTATTTATTA-GCCGATATGGGCGATAGCTTCTCTGGGGTTT-G-AGT
G-CG----GT--AA-CGTA----TCTTGGCTTCAAGGAG----TGTGTAGTCTGGCTTGA
TAG--GTACGATATT---AAAAA---A--CTTCTTAGAGGGACTACCT-GCCTCAAGCAG
GCGG-AAGTCCGAGGCAATAACAGGTCTGTGATGCCCTTAGATACCTT-GGGCCGCACGC
GCGCTACAATGCAGATAGCAAAAAGGT-----TCC--------TGGCCTGG-AAA---GG
TT-GGGTAATCAATTGAATTTTCTGCGTAACTGGGATTG-ATCTTTGTAATTATTGATCA
TCAACGAGGAATTCCTTGTAAGCGTAAGTCATTACCTTATGCTGAATATGTCCCTGCCCT
TTGTACACACCGCCCGTCGCTCCTACCGATCGAATGATACGGTAAAGTTAACGGATTG--
--------TTTT---TTTGTGG-C-AAC-ACAATTA------AAA--TTA-AAAG--TTA

-TT-TAAATCT--C----ATT--GTTT
>FJ424839.1_Dictyostelium_purpureum_QSpu2
--ACAA--CA
---GT----GATAAACTG-CTAGACTTTCGG-GTTTTT----AACCTTTT-GGATAACCG
CAGTAAATCGGGGCTAATACATAGAAG-CG-ATGGG-TGACTGGC-----AAC-G-G-AA
GCTCAGCGATTATT----A-GCG-TTA-CTA-CCAATAC--CTTC--GGGTCTTGT-GGT
GAAACCGAATAATATTGCAGATCGAGG--------ATT-TATCTTCGACAAGTCTACTGT
GTCACTGCCCTATCAACTTTCGATGGTACGGTATTGGCCTACCATGGTTGTAACGGGTAA
CGGGGAATTAGGGTTCGATTCCGGAGAGGGAGCCTGAGAAATGGCTACCACTTCTACGGA
AGGCAGCAGGCGCGCAAATT-ACTCAATCCCAATAC-GGGGAAGTAGTGACAATAAATAT
CAATACCTATCC--TT-TTTGGAGG--GCAATTGAAATGAACACAAATTAAAACTCTTAA
TT-AACACAATTGGAGGGCAAGTCTGGTGCCAGCAGCCGCGGTAATTCCAGCTCCAA-TA
GCATATACTAAAGTTGTTGCAGTTAAAA-AGC-TCGTAGTTGAAGTTAAAGTTATATT-G
GG-CTCA----------AGTTTTTA-CCACTCT----------GGTGGCTAAAA----C
AACTCCAATATTTCTTTTT-TT-----AATAG-CTCAGTTTCTAGG-TCTT-------TG
ACTCTAGTTATTTG--------------GGCATTTCACTGTGAGAAAA-TTGTGGTGTT
TAAAGCA--GG-C---GTC---TC-GTCTGATCTTTTGCAGCATGGTATGATGAAACATG
ACAT---TTTACGC--TATTGGTTT--GCG----TCTAAAGTGTAATGATTAATAGGGAT
GGATGGGGATGTTCATATTGGTGGGCGAGAGGTGAAATTCGTTGACCCTATCAAGATGAA
CTTCTGCGAAAGCATTCATCAAATACTTCCCCATTAATCAAGAACGAAAGTTTGGGGATC
GAAGACGATCAGATACCGTCGTAGTCCAAACTATAAACTATGTCGACCAGGGATCGGCTA
AA--AT-TTTTTTAAAATTTAGTCGGCACCTT-GTGAGAAATCAT-GAGTGTTTAGATTC
CGGGGGGAGTATGGTCGCAAGTCTGAAACTTAAAGGAATTGACGGAAGGGCACACAATGG
AGTGGAG-CCTGCGGCTTAATTT-GACTCAACTCGGGAAA-ACTTACCAAGCTAAGATAT
AGTAAGGATTGACAGA-CTAA-AAGATCTTTCATGATTCTATAAGTGGTGGTGCATGGTC
GTTCTTAG-TTGGTGGAGCGATTTGTCTGGTCAATTCCGATAACGGACGAGACCTCGACC
TGCTAACTAGTAGTATTTATTA-GTCGATATGGGCGATAGCTTTTCTGGGGTTAGG-A-T
G-CGA---GC--AATCGTA----TTCCTGCTTCAAGGAG----TGTGTAGTCTGACTTGA
TAG--GTACGCTTTC---AAAAA---A--CTTCTTAGAGGGACTACCT-GCCTCAAGCAG
GCGG-AAGTCCGAGGCAATAACAGGTCTGTGATGCCCTTAGATACCTT-GGGCCGCACGC
GCGCTACAATGCAGATAGCAAAAAGGT-----TCC--------TGGCCCGG-AAG---GG
TT-GGGTAATCAATTGAATTTTCTGCGTAACTGGGATTG-ATCTTTGTAATTATTGATCA
TCAACGAGGAATTCCTTGTAAGCGTAAGTCATTACCTTATGCTGAATATGTCCCTGCCCT
TTGTACACACCGCCCGTCGCTCCTACCGATCGAATGATACGGTAAAGTTAACGGATAG--
--------TTTT---TTTGTGG-C-AAC-ACAACTA------AAA--TTA-AAAG--TTA
-TT-TAAATCT--C----ATT--GTTT
>DQ340386.1_Dictyostelium_purpureum
TCTAAG-TATAAATTCTTG-TACGATGAAA----CTGCAGACGGCTCATT--ACAA--CA
---GT----GATAAACTG-CTAGACTTTCGG-GTTTTT----AACCTTTT-GGATAACCG
CAGTAAATCGGGGCTAATACATAGAAG-CG-ATGGG-TGACTGGC-----AAC-G-G-AA
GCTCAGCGATTATT----A-GCA-TTA-CTA-CCAATAC--CTTC--GGGTCTTGT-GGT
GAAACCGAATAATATTGCAGATCGGGG--------ATT-TATCTTCGACAAGTCTACTGT
GTCACTGCCCTATCAACTTTCGATGGTACGGTATTGGCCTACCATGGTTGTAACGGGTAA
CGGGGAATTAGGGTTCGATTCCGGAGAGGGAGCCTGAGAAATGGCTACCACTTCTACGGA
AGGCAGCAGGCGCGCAAATT-ACTCAATCCCAATAC-GGGGAAGTAGTGACAATAAATAT
CAATACCTATCC--TT-TTTGGAGG--GCAATTGAAATGAACACAAATTAAAACTCTTAA
TT-AACACAATTGGAGGGCAAGTCTGGTGCCAGCAGCCGCGGTAATTCCAGCTCCAA-TA
GCATATACTAAAGTTGTTGCAGTTAAAA-AGC-TCGTAGTTGAAGTTAAAGTTTTATT-G
GG-CTTA----------AGTTTTTA-CCAC-TCT---------G-TGGCTAAAA----T
AACTCCAATAATTCTTTTT-TT-----AATAG-CTCAGTTTCTAGG-TCTT-------TG
ACTCTAGTTATTTG--------------GGCATTTCACTGTGAGAAAA-TTGTGGTGTT
TAAAGCA--GG-C---GTC---TC-GTCTGATCTTTTGCAGCATGGTATGATGGAACATG
ACAT---TTTACGC--TATTGGTTT--GCG----TCTAAAGTGTAATGATTAATAGGGAT
GGATGGGGATGTTCATATTGGTGGGCGAGAGGTGAAATTCGTTGACCCTATCAAGATGAA
CTTCTGCGAAAGCATTCATCAAATACTTCCCCATTAATCAAGAACGAAAGTTTGGGGATC
GAAGACGATCAGATACCGTCGTAGTCCAAACTATAAACTATGTCGACCAGGGATCGGCTA
AA--AT-TTTTTTAAAATTTAGTCGGCACCTT-GTGAGAAATCAT-GAGTGTTTAGATTC
CGGGGGGAGTATGGTCGCAAGTCTGAAACTTAAAGGAATTGACGGAAGGGCACACAATGG
AGTGGAG-CCTGCGGCTTAATTT-GACTCAACTCGGGAAA-ACTTACCAAGCTAAGATAT
AGTAAGGATTGACAGA-CTAA-AAGATCTTTCATGATTCTATAAGTGGTGGTGCATGGTC
GTTCTTAG-TTGGTGGAGCGATTTGTCTGGTCAATTCCGATAACGGACGAGACCTCGACC
TGCTAACTAGTAGTATTTATTA-GTCGATATGGGCGATAGCTTCTCTGGGGTT-GG-AGT
G-CG----GC--AA-CGTA----TCTCTGCTTCAAGGAG----TGTGTAGTCTGACTTGA
TAG--GTACGTTATT---TAAAA---A--CTTCTTAGAGGGACTACCT-GCCTCAAGCAG
GCGG-AAGTCCGAGGCAATAACAGGTCTGTGATGCCCTTAGATACCTT-GGGCCGCACGC
GCGCTACAATGCAGATAGCAAAAAGGT-----TCC--------TGGCCTGG-AAA---GG
TT-GGGTAATCAATTGAATTTTCTGCGTAACTGGGATTG-ATCTTTGTAATTATTGATCA
TCAACGAGGAATTCCTTGTAAGCGTAAGTCATTACCTTATGCTGAATATGTCCCTGCCCT
TTGTACACACCGCCCGTCGCTCCTACCGATCGAATGATACGGTAAAGTTAACGGATAG--
--------TTTT---TTTGTGG-C-AAC-ACAATTA------AAA--TTA-AAAG--TTA
-TT-TAAATCT--C----ATT--GTTT
>FJ424826.1_Dictyostelium_purpureum_QSpu4
---------------CTTG-TACGATGAAA----CTGCAGACGGCTCATT--ACAA--CA

---GT----GATAAACTG-CTAGACTTTCGG-GTTTTT----AACCTTTT-GGATAACCG
CAGTAAATCGGGGCTAATACATAGAAG-CG-ATGGG-TGACTGGC-----AAC-G-G-AA
GCTCAGCGATTATT----A-GCA-TTA-CTA-CCAATAC--CTTC--GGGTCTTGT-GGT
GAAACCGAATAATATTGCAGATCGAGG--------ATT-TATCTTCGACAAGTCTACTGT
GTCACTGCCCTATCAACTTTCGATGGTACGGTATTGGCCTACCATGGTTGTAACGGGTAA
CGGGGAATTAGGGTTCGATTCCGGAGAGGGAGCCTGAGAAATGGCTACCACTTCTACGGA
AGGCAGCAGGCGCGCAAATT-ACTCAATCCCAATAC-GGGGAAGTAGTGACAATAAATAT
CAATACCTATCC--TT-TTTGGAGG--GCAATTGAAATGAACACAAATTAAAACTCTTAA
TT-AACACAATTGGAGGGCAAGTCTGGTGCCAGCAGCCGCGGTAATTCCAGCTCCAA-TA
GCATATACTAAAGTTGTTGCAGTTAAAA-AGC-TCGTAGTTGAAGTTAAAGTTTTATT-G
GG-CTTA----------AGTTTTTTA-CCACCTA----------G-TGGCTAAAA----T
AACTCCAATAATTCTTTTT-TT-----AATAG-CTCAGTTTCTAGG-TCTT-------TG
ACTCTAGTTATTTG---------------GGCATTTCACTGTGAGAAAA-TTGTGGTGTT
TAAAGCA--GG-C---GTC---TC-GTCTGATCTTTTGCAGCATGGTATGATGGAACATG
ACAT---TTTACGC--TATTGGTTT--GCG----TCTAAAGTGTAATGATTAATAGGGAT
GGATGGGGATGTTCATATTGGTGGGCGAGAGGTGAAATTCGTTGACCCTATCAAGATGAA
CTTCTGCGAAAGCATTCATCAAATACTTCCCCATTAATCAAGAACGAAAGTTTGGGGATC
GAAGACGATCAGATACCGTCGTAGTCCAAACTATAAACTATGTCGACCAGGGATCGGCTA
AA--AT-TTTTTTAAAATTTAGTCGGCACCTT-GTGAGAAATCAT-GAGTGTTTAGATTC
CGGGGGGAGTATGGTCGCAAGTCTGAAACTTAAAGGAATTGACGGAAGGGCACACAATGG
AGTGGAG-CCTGCGGCTTAATTT-GACTCAACTCGGGAAA-ACTTACCAAGCTAAGATAT
AGTAAGGATTGACAGA-CTAA-AAGATCTTTCATGATTCTATAAGTGGTGGTGCATGGTC
GTTCTTAG-TTGGTGGAGCGATTTGTCTGGTCAATTCCGATAACGGACGAGACCTCGACC
TGCTAACTAGTAGTATTTATTA-GTCGATATGGGCGATAGCTTCTCTGGGGTT-GG-AGT
G-CG----GC--AA-CGTA----TCTCTGCTTCAAGGAG----TGTGTAGTCTGACTTGA
TAG--GTACGTTATT---AAAAA---A--CTTCTTAGAGGGACTACCT-GCCTCAAGCAG
GCGG-AAGTCCGAGGCAATAACAGGTCTGTGATGCCCTTAGATACCTT-GGGCCGCACGC
GCGCTACAATGCAGATAGCAAAAAGGT-----TCC--------TGGCCTGG-AAA---GG
TT-GGGTAATCAATTGAATTTTCTGCGTAACTGGGATTG-ATCTTTGTAATTATTGATCA
TCAACGAGGAATTCCTTGTAAGCGTAAGTCATTACCTTATGCTGAATATGTCCCTGCCCT
TTGTACACACCGCCCGTCGCTCCTACCGATCGAATGATACGGTAAAGTTAACGGATAG--
--------TTTT---TTTGTGG-C-AAC-ACAATTA------AAA--TTA-AAAG--TTA
-TT-TAAATCT--C----ATT--GTTT
>FJ424832.1_Dictyostelium_purpureum_QSpu23
-------------TTCTTG-TACGATGAAA----CTGCAGACGGCTCATT--ACAA--CA
---GT----GATAAACTG-CTAGACTTTCGG-GTTTT-----AACCTTTT-GGATAACCG
CAGTAAATCGGGGCTAATACATAGAAG-CG-ATGGG-TGACTGGC-----AAC-G-G-AA
GCTCAGCGATTATT----A-GCG-TTA-CTA-CCAATAC--CTTC--GGGTCTTGT-GGT
GAAACCGAATAATATTGCAGATCGGGG--------ATT-TATCTTCGACAAGTCTACTGT
GTCACTGCCCTATCAACTTTCGATGGTACGGTATTGGCCTACCATGGTTGTAACGGGTAA
CGGGGAATTAGGGTTCGATTCCGGAGAGGGAGCCTGAGAAATGGCTACCACTTCTACGGA
AGGCAGCAGGCGCGCAAATT-ACTCAATCCCAATAC-GGGGAAGTAGTGACAATAAATAT
CAATACCTATCC--TT-TTTGGAGG--GCAATTGAAATGAACACAAATTAAAACTCTTAA
TT-AACACAATTGGAGGGCAAGTCTGGTGCCAGCAGCCGCGGTAATTCCAGCTCCAA-TA
GCATATACTAAAGTTGTTGCAGTTAAAA-AGC-TCGTAGTTGAAGTTAAAGTTATATT-G
GG-CTCA----------AGTTTTTA-CCACGTCT---------G-TGGCTAAAA----T
AACTCCAATATTTCTTTTT-TT-----AATAG-CTCAGTTTCTAGG-TCTT-------TG
ACTCTAGTTATTTG---------------GGCATTTCACTGTGAGAAAA-TTGTGGTGTT
TAAAGCA--GG-C---GTC---TC-GTCTGATCTTTTGCAGCATGGTATGATGGAACATG
ACAT---TTTACGC--TATTGGTTT--GCG----TCTAAAGTGTAATGATTAATAGGGAT
GGATGGGGATGTTCATATTGGTGGGCGAGAGGTGAAATTCGTTGACCCTATCAAGATGAA
CTTCTGCGAAAGCATTCATCAAATACTTCCCCATTAATCAAGAACGAAAGTTTGGGGATC
GAAGACGATCAGATACCGTCGTAGTCCAAACTATAAACTATGTCGACCAGGGATCGGCTA
AA--AT-TTTTTTAAAATTTAGTCGGCACCTT-GTGAGAAATCAT-GAGTGTTTAGATTC
CGGGGGGAGTATGGTCGCAAGTCTGAAACTTAAAGGAATTGACGGAAGGGCACACAATGG
AGTGGAG-CCTGCGGCTTAATTT-GACTCAACTCGGGAAA-ACTTACCAAGCTAAGATAT
AGTAAGGATTGACAGA-CTAA-AAGATCTTTCATGATTCTATAAGTGGTGGTGCATGGTC
GTCNNNNN-NNNNNNNNNNNNNNNNNNNNNNNNNNNNNTTCCGATAACGGACGAGACCTCGACC
TGCTAACTAGTAGTATTTATTA-GCCGATATGGGCGATAGCTTCTCTGGGGTT-TG-AGT
G-CG----GC--AA-CGTA----TCTTGGCTTCAAGGAG----TGTGTAGTCTGGCTTGA
TAG--GTACGATATT---AAAAA---A--CTTCTTAGAGGGACTACCT-GCCTCAAGCAG
GCGG-AAGTCCGAGGCAATAACAGGTCTGTGATGCCCTTAGATACCTT-GGGCCGCACGC
GCGCTACAATGCAGATAGCAAAAAGGT-----TCC--------TGGCCTGG-AAA---GG
TT-GGGTAATCAATTGAATTTTCTGCGTAACTGGGATTG-ATCTTTGTAATTATTGATCA
TCAACGAGGAATTCCTTGTAAGCGTAAGTCATTACCTTATGCTGAATATGTCCCTGCCCT
TTGTACACACCGCCCGTCGCTCCTACCGATCGAATGATACGGTAAAGTTAACGGATTG--
--------TTTT---TTTGTGG-C-AAC-ACAATTA------AAA--TTA-AAAG--TTA
-TT-TAAATCT--C----ATT--GTTT
>FJ424836.1_Dictyostelium_purpureum_QSpu28
-------TATAAATTCTTG-TACGATGAAA----CTGCAGACGGCTCATT--ACAA--CA
---GAT---GATAAACTG-CTAGACTTTCGG-GTTTT-----AACCTTTT-GGATAACCG
CAGTAAATCGGGGCTAATACATAGAAG-CG-ATGGG-TGACTGGC-----AAC-G-G-AA
GCTCAGCGATTATT----A-GCG-TTA-CTA-CCAATAC--CTTC--GGGTCTTGT-GGT

GAAACCGAATAATATTGCAGATCGGGG--------ATT-TATCTTCGACAAGTCTACTGT
GTCACTGCCCTATCAACTTTCGATGGTACGGTATTGGCCTACCATGGTTGTAACGGGTAA
CGGGGAATTAGGGTTCGATTCCGGAGAGGGAGCCTGAGAAATGGCTACCACTTCTACGGA
AGGCAGCAGGCGCGCAAATT-ACTCAATCCCAATAC-GGGGAAGTAGTGACAATAAATAT
CAATACCTATCC--TT-TTTGGAGG--GCAATTGAAATGAACACAAATTAAAACTCTTAA
TT-AACACAATTGGAGGGCAAGTCTGGTGCCAGCAGCCGCGGTAATTCCAGCTCCAA-TA
GCATATACTAAAGTTGTTGCAGTTAAAA-AGC-TCGTAGTTGAAGTTAAAGTTATATT-G
GG-CTCA----------AGTTTTTA-CCACGTCT---------G-TGGCTAAAA----T
AACTCCAATATTTCTTTTT-TT-----AATAG-CTCAGTTTCTAGG-TCTT-------TG
ACTCTAGTTATTTG---------------GGCATTTCACTGTGAGAAAA-TTGTGGTGTT
TAAAGCA--GG-C---GTC---TC-GTCTGATCTTTTGCAGCATGGTATGATGGAACATG
ACAT---TTTACGC--TATTGGTTT--GCG----TCTAAAGTGTAATGATTAATAGGGAT
GGATGGGGATGTTCATATTGGTGGGCGAGAGGTGAAATTCGTTGACCCTATCAAGATGAA
CTTCTGCGAAAGCATTCATCAAATACTTCCCCATTAATCAAGAACGAAAGTTTGGGGATC
GAAGACGATCAGATACCGTCGTAGTCCAAACTATAAACTATGTCGACCAGGGATCGGCTA
AA--AT-TTTTTTAAAATTTAGTCGGCACCTT-GTGAGAAATCAT-GAGTGTTTAGATTC
CGGGGGGAGTATGGTCGCAAGTCTGAAACTTAAAGGAATTGACGGAAGGGCACACAATGG
AGTGGAG-CCTGCGGCTTAATTT-GACTCAACTCGGGAAA-ACTTACCAAGCTAAGATAT
AGTAAGGATTGACAGA-CTAA-AAGATCTTTCATGATTCTATAAGTGGTGGTGCATGGTC
GTTCTTAG-TTGGTGGAGCGATTTGTCTGGTCAATTCCGATAACGGACGAGACCTCGACC
TGCTAACTAGTAGTATTTATTA-GCCGATATGGGCGATAGCTTCTCTGGGGTT-TG-AGT
G-CG----GC--AA-CGTA----TCTTGGCTTCAAGGAG----TGTGTAGTCTGGCTTGA
TAG--GTACGATATT---AAAAA---A--CTTCTTAGAGGGACTACCT-GCCTCAAGCAG
GCGG-AAGTCCGAGGCAATAACAGGTCTGTGATGCCCTTAGATACCTT-GGGCCGCACGC
GCGCTACAATGCAGATAGCAAAAAGGT-----TCC--------TGGCCTGG-AAA---GG
TT-GGGTAATCAATTGAATTTTCTGCGTAACTGGGATTG-ATCTTTGTAATTATTGATCA
TCAACGAGGAATTCCTTGTAAGCGTAAGTCATTACCTTATGCTGAATATGTCCCTGCCCT
TTGTACACACCGCCCGTCGCTCCTACCGATCGAATGATACGGTAAAGTTAACGGATTG--
--------TTTT---TTTGTGG-C-AAC-ACAATTA------AAA--TTA-AAAG--TTA
-TT-TAAATCT--C----ATT--GTTT
>AY040335.1_Dictyostelium_purpureum
TCTAAG-TATAAATTCTTG-TACGATGAAA----CTGCAGACGGCTCATT--ACAA--CA
---GT----GATAAACTG-CTAGACTTTCGG-GTTTTT----AACCTTTT-GGATAACCG
CAGTAAATCGGGGCTAATACATAGAAG-CG-ATGGG-TGACTGGC-----AAC-G-G-AA
GCTCAGCGATTATT----A-GCA-TTA-CTA-CCAATAC--CTTC--GGGTCTTGT-GGT
GAAACCGAATAATATTGCAGATCGAGG--------ATT-TATCTTCGACAAGTCTACTGT
GTCACTGCCCTATCAACTTTCGATGGTACGGTATTGGCCTACCATGGTTGTAACGGGTAA
CGGGGAATTAGGGTTCGATTCCGGAGAGGGAGCCTGAGAAATGGCTACCACTTCTACGGA
AGGCAGCAGGCGCGCAAATT-ACTCAATCCCAATAC-GGGGAAGTAGTGACAATAAATAT
CAATACCTATCC--TT-AACGGAGG--GCAATTGAAATGAACACAAATTAAAACTCTTAA
TT-AACACAATTGGAGGGCAAGTCTGGTGCCAGCAGCCGCGGTAATTCCAGCTCCAA-TA
GCATATACTAAAGTTGTTGCAGTTAAAA-AGC-TCGTAGTTGAAGTTAAAGTTTTATT-G
GG-CTAA----------CGTTTTTTA-CCACCCT----------G-TGGCTAAAA----T
AACTCCAATATTTCTTTTT-TT-----AATAG-CTCAGTTTCTAGG-TCTT-------TG
ACTCTAGTTATTTG---------------GGCATTTCACTGTGAGAAAA-TTGTGGTGTT
TAAAGCA--GG-C---GTC---TC-GTCTGATCTTTTGCAGCATGGTATGATGGAACATG
ACAT---TTTACGC--TATTGGTTT--GCG----TCTAAAGTGTAATGATTAATAGGGAT
GGATGGGGATGTTCATATTGGTGGGCGAGAGGTGAAATTCGTTGACCCTATCAAGATGAA
CTTCTGCGAAAGCATTCATCAAATACTTCCCCATTAATCAAGAACGAAAGTTTGGGGATC
GAAGACGATCAGATACCGTCGTAGTCCAAACTATAAACTATGTCGACCAGGGATCGGCTA
AT--AT-TTTTTTAAAATTTAGTCGGCACCTT-GTGAGAAATCAT-GAGTGTTTAGATTC
CGGGGGGAGTATGGTCGCAAGTCTGAAACTTAAAGGAATTGACGGAAGGGCACACAATGG
AGTGGAG-CCTGCGGCTTAATTT-GACTCAACTCGGGAAA-ACTTACCAAGCTAAGATAT
AGTAAGGATTGACAGA-CTAA-AAGATCTTTCATGATTCTATAAGTGGTGGTGCATGGTC
GTTCTTAG-TTGGTGGAGCGATTTGTCTGGTCAATTCCGATAACGGACGAGACCTCGACC
TGCTAACTAGTAGTATTTATTA-GTCGATATAGGCGATAGCTTCTCTGGGGTT-GG-AGT
G-CG----GC--AA-CGTA----TCTCTGCTTCAAGGAG----TGTGTAGTCTGACTTGA
TAG--GTACGTTAAC---AAAAA---A--CTTCTTAGAGGGACTACCT-GCCTCAAGCAG
GCGG-AAGTCCGAGGCAATAACAGGTCTGTGATGCCCTTAGATACCTT-GGGCCGCACGC
GCGCTACAATGCAGATAGCAAAAAGGT-----TCC--------TGGTCTGG-AAA---GA
TT-GGGTAATCAATTGAATTTTCTGCGTAACTGGGATTG-ATCTTTGTAATTATTGATCA
TCAACGAGGAATTCCTTGTAAGCGTAAGTCATTACCTTATGCTGAATATGTCCCTGCCCT
TTGTACACACCGCCCGTCGCTCCTACCGATCGAATGATACGGTAAAGTTAACGGATAG--
--------TTTT---TTTGTGG-C-AAC-ACAATTA------AAA--TTA-AAAG--TTA
-TT-TAAATCT--C----ATT--GTTT
>FJ424828.1_Dictyostelium_purpureum_QSpu36
TCTAAG-TATAAATTCTTG-TACGATGAAA----CTGCAGACGGCTCATT--ACAA--CA
---GT----GATAAACTG-CTAGACTTTCGG-GTTTTT----AACCTTTT-GGATAACCG
CAGTAAATCGGGGCTAATACATAGAAG-CG-ATGGG-TGACTGGC-----AAC-G-G-AA
GCTCAGCGATTATT----A-GCA-TTA-CTA-CCAATAC--CTTC--GGGTCTTGT-GGT
GAAACCGAATAATATTGCAGATCGAGG--------ATT-TATCTTCGACAAGTCTACTGT
GTCACTGCCCTATCAACTTTCGATGGTACGGTATTGGCCTACCATGGTTGTAACGGGTAA
CGGGGAATTAGGGTTCGATTCCGGAGAGGGAGCCTGAGAAATGGCTACCACTTCTACGGA

AGGCAGCAGGCGCGCAAATT-ACTCAATCCCAATAC-GGGGAAGTAGTGACAATAAATAT
CAATACCTATCC--TT-AACGGAGG--GCAATTGAAATGAACACAAATTAAAACTCTTAA
TT-AACACAATTGGAGGGCAAGTCTGGTGCCAGCAGCCGCGGTAATTCCAGCTCCAA-TA
GCATATACTAAAGTTGTTGCAGTTAAAA-AGC-TCGTAGTTGAAGTTAAAGTTTTATT-G
GG-CTAA----------CGTTTTTA-CCACCCT----------G-TGGCTAAAA----T
AACTCCAATATTTCTTTTT-TT-----AATAG-CTCAGTTTCTAGG-TCTT-------TG
ACTCTAGTTATTTG---------------GGCATTTCACTGTGAGAAAA-TTGTGGTGTT
TAAAGCA--GG-C---GTC---TC-GTCTGATCTTTTGCAGCATGGTATGATGGAACATG
ACAT---TTTACGC--TATTGGTTT--GCG----TCTAAAGTGTAATGATTAATAGGGAT
GGATGGGGATGTTCATATTGGTGGGCGAGAGGTGAAATTCGTTGACCCTATCAAGATGAA
CTTCTGCGAAAGCATTCATCAAATACTTCCCCATTAATCAAGAACGAAAGTTTGGGGATC
GAAGACGATCAGATACCGTCGTAGTCCAAACTATAAACTATGTCGACCAGGGATCGGCTA
AT--AT-TTTTTTAAAATTTAGTCGGCACCTT-GTGAGAAATCAT-GAGTGTTTAGATTC
CGGGGGGAGTATGGTCGCAAGTCTGAAACTTAAAGGAATTGACGGAAGGGCACACAATGG
AGTGGAG-CCTGCGGCTTAATTT-GACTCAACTCGGGAAA-ACTTACCAAGCTAAGATAT
AGTAAGGATTGACAGA-CTAA-AAGATCTTTCATGATTCTATAAGTGGTGGTGCATGGTC
GTTCTTAG-TTGGTGGAGCGATTTGTCTGGTCAATTCCGATAACGGACGAGACCTCGACC
TGCTAACTAGTAGTATTTATTA-GTCGATATAGGCGATAGCTTCTCTGGGGTT-GG-AGT
G-CG----GC--AA-CGTA----TCTCTGCTTCAAGGAG----TGTGTAGTCTGACTTGA
TAG--GTACGTTAAC---AAAAA---A--CTTCTTAGAGGGACTACCT-GCCTCAAGCAG
GCGG-AAGTCCGAGGCAATAACAGGTCTGTGATGCCCTTAGATACCTT-GGGCCGCACGC
GCGCTACAATGCAGATAGCAAAAAGGT-----TCC--------TGGTCTGG-AAA---GA
TT-GGGTAATCAATTGAATTTTCTGCGTAACTGGGATTG-ATCTTTGTAATTATTGATCA
TCAACGAGGAATTCCTTGTAAGCGTAAGTCATTACCTTATGCTGAATATGTCCCTGCCCT
TTGTACACACCGCCCGTCGCTCCTACCGATCGAATGATACGGTAAAGTTAACGGATAG--
--------TTTT---TTTGTGG-C-AAC-ACAATTA------AAA--TTA-AAAG--TTA
-TT-TAAATCT--C----ATT--GTTT
>AM168061.1_Dictyostelium_purpureum_WS321
TCTAAG-TATAAATTCTTG-TACGATGAAA----CTGCAGACGGCTCATT--ACAA--CA
---GT----GATAAACTG-CTAGACTTTCGG-GTTTTT----AACCTTTT-GGATAACCG
CAGTAAATCGGGGCTAATACATAGAAG-CG-ATGGG-TGACTGGC-----AAC-G-G-AA
GCTCAGCGATTATT----A-GCA-TTA-CTA-CCAATAC--CTTC--GGGTCTTGT-GGT
GAAACCGAATAATATTGCAGATCGAGG--------ATT-TATCTTCGGCAAGTCTACTGT
GTCACTGCCCTATCAACTTTCGATGGTACGGTATTGGCCTACCATGGTTGTAACGGGTAA
CGGGGAATTAGGGTTCGATTCCGGAGAGGGAGCCTGAGAAATGGCTACCACTTCTACGGA
AGGCAGCAGGCGCGCAAATT-ACTCAATCCCAATAC-GGGGAAGTAGTGACAATAAATAT
CAATACCTATCC--TT-AACGGAGG--GCAATTGAAATGAACACAAATTAAAACTCTTAA
TT-AACACAATTGGAGGGCAAGTCTGGTGCCAGCAGCCGCGGTAATTCCAGCTCCAA-TA
GCATATACTAAAGTTGTTGCAGTTAAAA-AGC-TCGTAGTTGAAGTTAAAGTTTTATT-G
GG-CTAA----------CGTTTTTA-CCGCTCT----------GC-GGCTAAAA----T
AACTCCAATAATTCTTTTT-TT-----AATAG-CTCAGTTTCTAGG-TCTT-------TG
ACTCTAGTTATTTG---------------GGCATTTCACTGTGAGAAAA-TTGTGGTGTT
TAAAGCA--GG-C---GTC---TC-GTCTGATCTTTTGCAGCATGGTATGATGGAACATG
ACAT---TTTACGC--TATTGGTTT--GCG----TCTAAAGTGTAATGATTAATAGGGAT
GGATGGGGATGTTCATATTGGTGGGCGAGAGGTGAAATTCGTTGACCCTATCAAGATGAA
CTTCTGCGAAAGCATTCATCAAATACTTCCCCATTAATCAAGAACGAAAGTTTGGGGATC
GAAGACGATCAGATACCGTCGTAGTCCAAACTATAAACTATGTCGACCAGGGATCGGCTA
AT--AT-TTTTTTAAAATTTAGTCGGCACCTT-GTGAGAAATCAT-GAGTGTTTAGATTC
CGGGGGGAGTATGGTCGCAAGTCTGAAACTTAAAGGAATTGACGGAAGGGCACGCAATGG
AGTGGAG-CCTGCGGCTTAATTT-GACTCAACTCGGGAAA-ACTTACCAAGCTAAGATAT
AGTAAGGATTGACAGA-CTAA-AAGATCTTTCATGATTCTATAAGTGGTGGTGCATGGTC
GTTCTTAG-TTGGTGGAGCGATTTGTCTGGTCAATTCCGATAACGGACGAGACCTCGACC
TGCTAACTAGTAGTATTTATTG-GTCAATACGGGCGATAGCTTTTCTGGGGTT-AG-AGT
G-CG----GC--AA-CGTA----TCTCTGCTTCAAGGAG----TGTGTAGTCTGACTTGA
TAG--GTACGTTAAC---AAAAA---A--CTTCTTAGAGGGACTACCT-GCCTCAAGCAG
GCGG-AAGTCCGAGGCAATAACAGGTCTGTGATGCCCTTAGATACCTT-GGGCCGCACGC
GCGCTACAATGCAGATAGCAAAAAGGT-----TCC--------TGGTCTGG-AAA---GA
TT-GGGTAATCAATTGAATTTTCTGCGTAACTGGGATTG-ATCTTTGTAATTATTGATCA
TCAACGAGGAATTCCTTGTAAGCGTAAGTCATTACCTTATGCTGAATATGTCCCTGCCCT
TTGTACACACCGCCCGTCGCTCCTACCGATCGAATGATACGGTAAAGTTAACGGATAG--
--------TTTT---TTTGTGG-C-AAC-ACAATTA------AAA--TTA-AAAG--TTA
-TT-TAAATCT--C----ATT--GTTT
>HQ141481.1_Dictyostelium_purpureum_cavender
TCTA-G-TATAAATTCTTG-TACGATGAAA----CTGCAGACGGCTCATT--ACAA--CA
---GT----GATAAACTG-CTAGACTTTCGG-GTTTTT----AACCTTTT-GGATAACCG
CAGTAAATCGGGGCTAATACATAGAAG-CG-ATGGG-TGACTGGC-----AAC-G-G-AA
GCTCAGCGATTATT----A-GCA-TTA-CTA-CCAATAC--CTTC--GGGTCTTGT-GGT
GAAACCGAATAATATTGCAGATCGAGG--------ATT-TATCTTCGACAAGTCTACTGT
GTCACTGCCCTATCAACTTTCGATGGTACGGTATTGGCCTACCATGGTTGTAACGGGTAA
CGGGGAATTAGGGTTCGATTCCGGAGAGGGAGCCTGAGAAATGGCTACCACTTCTACGGA
AGGCAGCAGGCGCGCAAATT-ACTCAATCCCAATAC-GGGGAAGTAGTGACAATAAATAT
CAATACCTATCC--TT-AACGGAGG--GCAATTGAAATGAACACAAATTAAAACTCTTAA
TT-AACACAATTGGAGGGCAAGTCTGGTGCCAGCAGCCGCGGTAATTCCAGCTCCAA-TA

GCATATACTAAAGTTGTTGCAGTTAAAA-AGC-TCGTAGTTGAAGTTAAAGTTTTATT-G
GG-CTAA----------CGTTTTTTA-CCGCTCT----------GCTGGCTAAAA----T
AACTCCAATAATTCTTTTT-TT-----AATAG-CTCAGTTTCTAGG-TCTT-------TG
ACTCTAGTTATTTG---------------GGCATTTCACTGTGAGAAAA-TTGTGGTGTT
TAAAGCA--GG-C---GTC---TC-GTCTGATCTTTTGCAGCATGGTATGATGGAACATG
ACAT---TTTACGC--TATTGGTTT--GCG----TCTAAAGTGTAATGATTAATAGGGAT
GGATGGGGATGTTCATATTGGTGGGCGAGAGGTGAAATTCGTTGACCCTATCAAGATGAA
CTTCTGCGAAAGCATTCATCAAATACTTCCCCATTAATCAAGAACGAAAGTTTGGGGATC
GAAGACGATCAGATACCGTCGTAGTCCAAACTATAAACTATGTCGACCAGGGATCGGCTA
AT--AT-TTTTTTAAAATTTAGTCGGCACCTT-GTGAGAAATCAT-GAGTGTTTAGATTC
CGGGGGGAGTATGGTCGCAAGTCTGAAACTTAAAGGAATTGACGGAAGGGCACACAATGG
AGTGGAG-CCTGCGGCTTAATTT-GACTCAACTCGGGAAA-ACTTACCAAGCTAAGATAT
AGTAAGGATTGACAGA-CTAA-AAGATCTTTCATGATTCTATAAGTGGTGGTGCATGGTC
GTTCTTAG-TTGGTGGAGCGATTTGTCTGGTCAATTCCGATAACGGACGAGACCTCGACC
TGCTAACTAGTAGTATTTATTG-GTCAATACGGGCGATAGCTTTTCTGGGGTT-AG-AGT
G-CG----GC--AA-CGTA----TCTCTGCTTCAAGGAG----TGTGTAGTCTGACTTGA
TAG--GTACGTTAAC---AAAAA------CTTCTTAGAGGGACTACCT-GCCTCAAGCAG
GCGG-AAGTCCGAGGCAATAACAGGTCTGTGATGCCCTTAGATACCTT-GGGCCGCACGC
GCGCTACAATGCAGATAGCAAAAAGGT-----TCC--------TGGTCTGG-AAA---GA
TT-GGGTAATCAATTGAATTTTCTGCGTAACTGGGATTG-ATCTTTGTAATTATTGATCA
TCAACGAGGAATTCCTTGTAAGCGTAAGTCATTACCTTATGCTGAATATGTCCCTGCCCT
TTGTACACACCGCCCGTCGCTCCTACCGATCGAATGATACGGTAAAGTTAACGGATAG--
--------TTTT---TTTGTGG-C-AAC-ACAATTA------AAAA-TTA-AAAG--TTA
-TT-TAAATCT--C----ATT--GTTT
>AM168060.1_Dictyostelium_purpureum_C143
TCTAAG-TATAAATTCTTG-TACGATGAAA----CTGCAGACGGCTCATT--ACAA--CA
---GT----GATAAACTG-CTAGACTTTCGG-GTTTTT----AACCTTTT-GGATAACCG
CAGTAAATCGGGGCTAATACATAGAAG-CG-ATGGG-TGACTGGC-----AAC-G-G-AA
GCTCAGCGATTATT----A-GCA-TTA-CTA-CCAATAC--CTTC--GGGTCTTGT-GGT
GAAACCGAATAATATTGCAGATCGAGG--------ATT-TATCTTCGACAAGTCTACTGT
GTCACTGCCCTATCAACTTTCGATGGTACGGTATTGGCCTACCATGGTTGTAACGGGTAA
CGGGGAATTAGGGTTCGATTCCGGAGAGGGAGCCTGAGAAATGGCTACCACTTCTACGGA
AGGCAGCAGGCGCGCAAATT-ACTCAATCCCAATAC-GGGGAAGTAGTGACAATAAATAT
CAATACCTATCC--TT-TTTGGAGG--GCAATTGAAATGAACACAAATTAAAACTCTTAA
TT-AACACAATTGGAGGGCAAGTCTGGTGCCAGCAGCCGCGGTAATTCCAGCTCCAA-TA
GCATATACTAAAGTTGTTGCAGTTAAAA-AGC-TCGTAGTTGAAGTTAAAGTTTTATT-G
GG-CTTA----------AGTTTTTTA-CCACCTA----------G-TGGCTAAAA----C
AACTCCAATAATTCTTTTT-TT-----AATAG-CTCAGTTTCTAGG-TCTT-------TG
ACTCTAGTTATTTG---------------GGCATTTCACTGTGAGAAAA-TTGTGGTGTT
TAAAGCA--GG-C---GTC---TC-GTCTGATCTTTTGCAGCATGGTATGATGGAACATG
ACAT---TTTACGC--TATTGGTTT--GCG----TCTAAAGTGTAATGATTAATAGGGAT
GGATGGGGATGTTCATATTGGTGGGCGAGAGGTGAAATTCGTTGACCCTATCAAGATGAA
CTTCTGCGAAAGCATTCATCAAATACTTCCCCATTAATCAAGAACGAAAGTTTGGGGATC
GAAGACGATCAGATACCGTCGTAGTCCAAACTATAAACTATGTCCACCAGGGATCGGTTA
AA--AT-TTTTTTAAAATTTAGTCGGCACCTT-GTGAGAAATCAT-GAGTGTTTAGATTC
CGGGGGGAGTATGGTCGCAAGTCTGAAA-TTAAAGGAATTGACGGAAGGGCACACAATGG
AGTGGAG-CCTGCGGCTTAATTT-GACTCAACTCGGGAAA-ACTTACCAAGCTAAGATAT
AGTAAGGATTGACAGA-CTAA-AAGATCTTTCATGATTCTATAAGTGGTGGTGCATGGTC
GTTCTTAG-TTGGTGGAGCGATTTGTCTGGTCAATTCCGATAACGGACGAGACCTCGACC
TGCTAACTAGTAGTATTTATTA-GTCAATATGGGCGATAGCTTTTCTGGGATTTAG-AGC
GATT----TC--GGTCGTT----TCT-GGTTTCAAGGAG----TGTGTAGTCTGACTTGA
TAG--GTACGAATTA---AAAAA---A--CTTCTTAGAGGGACTACCT-GCCTCAAGCAG
GCGG-AAGTCCGAGGCAATAACAGGTCTGTGATGCCCTTAGATACCTT-GGGCCGCACGC
GCGCTACAATGTATAAAACAAAAAGGT-----TCC--------TGGTCCGG-AAG---GA
TT-GGGTAATCAATTGAATTTTCTACGTAACTGGGATTG-ATCTTTGTAATTATTGATCA
TAAACGAGGAATTCCTTGTAAGCGCAGGTCATTACCCTGTGCTGAATATGTCCCTGCCCT
TTGTACACACCGCCCGTCGCTCCTACCGATCGAATGATACGGTAAAGTTAACGGATAG--
--------TTTT--ATCTTTGG-C-AAC-ATTGATA------TAAA-TTA-AAAG--TTA
-TT-TAAATCT--C----ATT--GTTT
>MK322959.1_Dictyostelium_barbarae_1_5
TCTAAG-TATAAATTCTTG-TACGATGAAA----CTGCAGACGGCTCATT--ACAA--CA
---GT----GATAAACTA-ATAGACTTTCGG-GTTTTT----AACCTTTT-GGATAACCG
CAGTAAATCGGGGCTAATACATACAAG-CG-ATGGG-TGACTGGC-----AAC-G-G-AA
GCTCAGCGATTATT----A-GCA-TTA-CTA-CCAATAC--CTTC--GGGTCTTGT-GGT
GAAACCGAATAATATTGCAGATCGAGG--------ATT-TATCTTCGACAAGTCTACTGT
GTCACTGCCCTATCAACTTTCGATGGTACGGTATTGGCCTACCATGGTTGTAACGGGTAA
CGGGGAATTAGGGTTCGATTCCGGAGAGGGAGCCTGAGAAATGGCTACCACTTCTACGGA
AGGCAGCAGGCGCGCAAATT-ACTCAATCCCAATAC-GGGGAAGTAGTGACAATAAATAT
CAATACCTATCC--TT-AACGGAGG--GCAATTGAAATGAACACAAATTAAAACTCTTAA
TT-AACACAATTGGAGGGCAAGTCTGGTGCCAGCAGCCGCGGTAATTCCAGCTCCAA-TA
GCATATACTAAAGTTGTTGCAGTTAAAA-AGC-TCGTAGTTGAAGTTAAAGGTTTACT-G
GG-CTAA----------AGTTATTTG-CCACTCT----------GGTGGTTAAAT----C
AACTCCAGTATTCCTTTTT-TT-----AATAG-CTCTGCTTCTAGCATCTT-------TG

ATGTTAGTTGTTTG---------------GGCATTTCACTGTGAGAAAA-TTGTGGTGTT
TAAAGCA--GG-C---GTC---TC-GCCTGATCTTTTGCAGCATGGTATGATGAAACATG
ACAT---TTTACGC--TATTGGTTT--GCG----TCTAAAGTGTAATGATTAATAGGGAT
GGATGGGGGTGTTCATATTGGTGGGCGAGAGGTGAAATTCGTTGACCCTATCAAGATGAA
CTTCTGCGAAAGCATTCACCAAATACTTCCCCATTAATCAAGAACGAAAGTTTGGGGATC
GAAGACGATCAGATACCGTCGTAGTCCAAACTATAAACTATGTCGACCAGGGATCGGTCA
AA--AT-TTTTTTAAAATTTGATCGGCACCTT-GTGAGAAATCAT-GAGTGTTTAGATTC
CGGGGGGAGTATGGTCGCAAGTCTGAAACTTAAAGGAATTGACGGAAGGGCACACAATGG
AGTGGAG-CCTGCGGCTTAATTT-GACTCAACTCGGGAAA-ACTTACCAAGCTAAGATAT
AGTAAGGATTGACAGA-CTAA-AAGATCTTTCATGATTCTATAAGTGGTGGTGCATGGTC
GTTCTTAG-TTGGTGGAGCGATTTGTCTGGTCAATTCCGATAACGGACGAGACCTCGACC
TGCTAACTAGTAGTATTTATTA-GCCGATATGGGCGATAGCTTCTCTGGGGTTTGG-AAT
GATT----TC--GGTCATC----TCC-TACTTCAAGGAG----TGTGTAGTCTGGCTTGA
TAG--GTACGAATTA---AAAAA------CTTCTTAGAGGGACTACCT-GCCTCAAGCAG
GCGG-AAGTCCGAGGCAATAACAGGTCTGTGATGCCCTTAGATACCTT-GGGCCGCACGC
GCGCTACAATGTAGAAAACAAAAAGGC-----TCC--------TGGTCCGG-AAG---GA
TT-GGGTAATCATTTGAATTTTCTACGTAACTGGGATTG-ATCTTTGTAATTATTGATCA
TCAACGAGGAATTCCTTGTAAGCGCAGGTCATTACCCTGTGCTGAATATGTCCCTGCCCT
TTGTACACACCGCCCGTCGCTCCTACCGATCGAATGATACGGTAAAGTTAACGGATCG--
--------TTTT--ATCTGTGG-C-AAC-ACTGATA------TAAA-CTA-AAAG--TTA
-TT-TAAATCT--C----ATT--GTTT
>MK322958.1_Dictyostelium_insulinativitatis
TCTAAG-TATAAATTCTTG-TACGATGAAA----CTGCAGACGGCTCATT--ACAA--CA
---GT----GATAAACTA-ATAGACTTTCGG-GTTTTT----AACCTTTT-GGATAACCG
CAGTAAATCGGGGCTAATACATACAAG-CG-ATGGG-TGACTGGC-----AAC-G-G-AA
GCTCAGCGATTATT----A-GCA-TTA-CTA-CCAATAC--CTTC--GGGTCTTGT-GGT
GAAACCGAATAATATTGCAGATCGAGG--------ATT-TATCTTCGACAAGTCTACTGT
GTCACTGCCCTATCAACTTTCGATGGTACGGTATTGGCCTACCATGGTTGTAACGGGTAA
CGGGGAATTAGGGTTCGATTCCGGAGAGGGAGCCTGAGAAATGGCTACCACTTCTACGGA
AGGCAGCAGGCGCGCAAATT-ACTCAATCCCAATAC-GGGGAAGTAGTGACAATAAATAT
CAATACCTATCC--TT-AACGGAGG--GCAATTGAAATGAACACAAATTAAAACTCTTAA
TT-AACACAATTGGAGGGCAAGTCTGGTGCCAGCAGCCGCGGTAATTCCAGCTCCAA-TA
GCATATACTAAAGTTGTTGCAGTTAAAA-AGC-TCGTAGTTGAAGTTAAAGGTTTACT-G
GG-CTAA----------AGTTATTTG-CCACTCT----------GGTGGTTAAAT----C
AACTCCAGTATTCCTTTTT-TT-----AATAG-CTCTGCTTCTAGCATCTT-------TG
ATGTTAGTTGTTTG---------------GGCATTTCACTGTGAGAAAA-TTGTGGTGTT
TAAAGCA--GG-C---GTC---TC-GCCTGATCTTTTGCAGCATGGTATGATGAAACATG
ACAT---TTTACGC--TATTGGTTT--GCG----TCTAAAGTGTAATGATTAATAGGGAT
GGATGGGGGTGTTCATATTGGTGGGCGAGAGGTGAAATTCGTTGACCCTATCAAGATGAA
CTTCTGCGAAAGCATTCACCAAATACTTCCCCATTAATCAAGAACGAAAGTTTGGGGATC
GAAGACGATCAGATACCGTCGTAGTCCAAACTATAAACTATGTCGACCAGGGATCGGTCA
AA--AT-TTTTTTAAAATTTGATCGGCACCTT-GTGAGAAATCAT-GAGTGTTTAGATTC
CGGGGGGAGTATGGTCGCAAGTCTGAAACTTAAAGGAATTGACGGAAGGGCACACAATGG
AGTGGAG-CCTGCGGCTTAATTT-GACTCAACTCGGGAAA-ACTTACCAAGCTAAGATAT
AGTAAGGATTGACAGA-CTAA-AAGATCTTTCATGATTCTATAAGTGGTGGTGCATGGTC
GTTCTTAG-TTGGTGGAGCGATTTGTCTGGTCAATTCCGATAACGGACGAGACCTCGACC
TGCTAACTAGTAGTATTTATTA-GCCGATATGGGCGATAGCTTCTCTGGGGTTTGG-AAT
GATT----TC--GGTCATC----TCC-TACTTCAAGGAG----TGTGTAGTCTGGCTTGA
TAG--GTACGAATTA---AAAAA------CTTCTTAGAGGGACTACCT-GCCTCAAGCAG
GCGG-AAGTCCGAGGCAATAACAGGTCTGTGATGCCCTTAGATACCTT-GGGCCGCACGC
GCGCTACAATGTAGAAAACAAAAAGGC-----TCC--------TGGTCCGG-AAG---GA
TT-GGGTAATCATTTGAATTTTCTACGTAACTGGGATTG-ATCTTTGTAATTATTGATCA
TCAACGAGGAATTCCTTGTAAGCGCAGGTCATTACCCTGTGCTGAATATGTCCCTGCCCT
TTGTACACACCGCCCGTCGCTCCTACCGATCGAATGATACGGTAAAGTTAACGGATCG--
--------TTTT--ATCTGTGG-C-AAC-ACTGATA------TAAA-CTA-AAAG--TTA
-TT-TAAATCT--C----ATT--GTTT
>AM168049.1_Dictyostelium_macrocephalum_B33
TCTAAG-TATAAATTCTTG-TACGATGAAA----CTGCAGACGGCTCATT--ACAA--CA
---GT----GATAAACTA-ATAGACTTTCGG-GTTTTT----AACCTTTT-GGATAACCG
CAGTAAATCGGGGCTAATACATACAAG-CG-ATGGG-TGACTGGC-----AAC-G-G-AA
GCTCAGCGATTATT----A-GCA-TTA-CTA-CCAATAC--CTTC--GGGTCTTGT-GGT
GAAACCGAATAATATTGCAGATCGAGG--------ATT-TATCTTCGACAAGTCTACTGT
GTCACTGCCCTATCAACTTTCGATGGTACGGTATTGGCCTACCATGGTTGTAACGGGTAA
CGGGGAATTAGGGTTCGATTCCGGAGAGGGAGCCTGAGAAATGGCTACCACTTCTACGGA
AGGCAGCAGGCGCGCAAATT-ACTCAATCCCAATAC-GGGGAAGTAGTGACAATAAATAT
CAATACCTATCC--TT-AACGGAGG--GCAATTGAAATGAACACAAATTAAAACTCTTAA
TT-AACACAATTGGAGGGCAAGTCTGGTGCCAGCAGCCGCGGTAATTCCAGCTCCAA-TA
GCATATACTAAAGTTGTTGCAGTTAAAA-AGC-TCGTAGTTGAAGTTAAAGGTTTACT-G
GG-CTAA----------AGTTATTTG-CCACTCT----------GGTGGTTAAAT----C
AACTCCAGTATTCCTTTTT-TT-----AATAG-CTCTGCTTCTAGCATCTT-------TG
ATGTTAGTTGTTTG---------------GGCATTTCACTGTGAGAAAA-TTGTGGTGTT
TAAAGCA--GG-C---GTC---TC-GCCTGATCTTTTGCAGCATGGTATGATGAAACATG
ACAT---TTTACGC--TATTGGTTT--GCG----TCTAAAGTGTAATGATTAATAGGGAT

GGATGGGGGTGTTCATATTGGTGGGCGAGAGGTGAAATTCGTTGACCCTATCAAGATGAA
CTTCTGCGAAAGCATTCACCAAATACTTCCCCATTAATCAAGAACGAAAGTTTGGGGATC
GAAGACGATCAGATACCGTCGTAGTCCAAACTATAAACTATGTCGACCAGGGATCGGTCA
AA--AT-TTTTTTAAAATTTGATCGGCACCTT-GTGAGAAATCAT-GAGTGTTTAGATTC
CGGGGGGAGTATGGTCGCAAGTCTGAAACTTAAAGGAATTGACGGAAGGGCACACAATGG
AGTGGAG-CCTGCGGCTTAATTT-GACTCAACTCGGGAAA-ACTTACCAAGCTAAGATAT
AGTAAGGATTGACAGA-CTAA-AAGATCTTTCATGATTCTATAAGTGGTGGTGCATGGTC
GTTCTTAG-TTGGTGGAGCGATTTGTCTGGTCAATTCCGATAACGGACGAGACCTCGACC
TGCTAACTAGTAGTATTTATTA-GCCGATATGGGCGATAGCTTCTCTGGGGTTTGG-AAT
GATT----TC--GGTCATC----TCC-TATTTCAAGGAG----TGTGTAGTCTGGCTTGA
TAG--GTACGAATTA---AAAAA------CTTCTTAGAGGGACTACCT-GCCTCAAGCAG
GCGG-AAGTCCGAGGCAATAACAGGTCTGTGATGCCCTTAGATACCTT-GGGCCGCACGC
GCGCTACAATGTAGAAAACAAAAAGGC-----TCC--------TGGTCCGG-AAG---GA
TT-GGGTAATCATTTGAATTTTCTACGTAACTGGGATTG-ATCTTTGTAATTATTGATCA
TCAACGAGGAATTCCTTGTAAGCGCAGGTCATTACCCTGTGCTGAATATGTCCCTGCCCT
TTGTACACACCGCCCGTCGCTCCTACCGATCGAATGATACGGTAAAGTTAACAGATCG--
--------TTTT--ATCTGTGG-C-AAC-ACTGATA------TAAA-CTA-AAAG--TTA
-TT-TAAATCT--C----ATT--GTTT
>MG490371.1_Dictyostelium_multiforme_4009
-------TATAAATTCTTG-TACGATGAAA----CTGCAGACGGCTCATT--ACAA--CA
---GT----GATAAACTA-ATAGACTTTCGG-GTTTTT----AACCTTTT-GGATAACCG
CAGTAAATCGGGGCTAATACATAGAAG-CG-ATGGG-TGACTGGC-----AAC-G-G-AA
GCTCAGCGATTATT----A-GCA-TTA-CTA-CCAATAC--CTTC--GGGTCTTGT-GGT
GAAACCGAATAATATTGCAGATCGAGG--------ATT-TATCTTCGACAAGTCTACTGT
GTCACTGCCCTATCAACTTTCGATGGTACGGTATTGGCCTACCATGGTTGTAACGGGTAA
CGGGGAATTAGGGTTCGATTCCGGAGAGGGAGCCTGAGAAATGGCTACCACTTCTACGGA
AGGCAGCAGGCGCGCAAATT-ACTCAATCCCAATAC-GGGGAAGTAGTGACAATAAATAT
CAATACCTATCC--TT-AACGGAGG--GCAATTGAAATGAACACAAATTAAAACTCTTAA
TT-AACACAATTGGAGGGCAAGTCTGGTGCCAGCAGCCGCGGTAATTCCAGCTCCAA-TA
GCATATACTAAAGTTGTTGCAGTTAAAA-AGC-TCGTAGTTGAAGTTAAAGGTTTACT-G
GG-CTAA----------AGTTATTTG-CCGCTCT----------GGTGGTTAAAT----C
AACTCCAGTATCTCTTTTT-TT-----AATAG-TTCAGCTTCTATTATCTT-------TG
ATAGTAGTTGTTTG---------------GACATTTCACTGTGAGAAAA-TTGTGGTGTT
TAAAGCA--GG-C---GTT---TC-GCCTGATCTTTTGCAGCATGGTATGATGAAACATG
ACAT---TTTACGC--TATTGGTTT--GCG----TCTAAAGTGTAATGATTAATAGGGAT
GGATGGGGGTGTTCATATTGGTGGGCGAGAGGTGAAATTCGTTGACCCTATCAAGATGAA
CTTCTGCGAAAGCATTCACCAAATACTTCCCCATTAATCAAGAACGAAAGTTTGGGGATC
GAAGACGATCAGATACCGTCGTAGTCCAAACTATAAACTATGTCGACCAGGGATCGGTTA
AA--AT-TTTTTTAAAATTTAATCGGCACCTT-GTGAGAAATCAC-GAGTGTTTAGATTC
CGGGGGGAGTATGGTCGCAAGTCTGAAACTTAAAGGAATTGACGGAAGGGCACACAATGG
AGTGGAG-CCTGCGGCTTAATTT-GACTCAACTCGGGAAA-ACTTACCAAGCTAAGATAT
AGTAAGGATTGACAGA-CTAA-AAGATCTTTCATGATTCTATAAGTGGTGGTGCATGGTC
GTTCTTAG-TTGGTGGAGCGATTTGTCTGGTCAATTCCGATAACGGACGAGACCTCGACC
TGCTAACTAGTAGTATTTATTA-GTCAATATGGGCGATAGCTTCTCTGGGGTTTGG-AAT
GGTT----TC--GGCCATC----TCC-TGCTTCAAGGAG----TGTGTAGTCTGACTTGA
TAG--GTACGAATTA---AAAAA------CTTCTTAGAGGGACTACCT-GCCTCAAGCAG
GCGG-AAGTCCGAGGCAATAACAGGTCTGTGATGCCCTTAGATACCTT-GGGCCGCACGC
GCGCTACAATGTAGAAAACAAAAAGGT-----TCC--------TGGTCCGG-AAG---GA
TT-GGGTAATCATTTGAATTTTCTACGTAACTGGGATTG-ATCTTTGTAATTATTGATCA
TCAACGAGGAATTCCTTGTAAGCGTAGGTCATTACCCTATGCTGAATATGTCCCTGCCCT
TTGTACACACCGCCCGTCGCTCCTACCGATCGAATGATACGGTAAAGTTAACGGATCG--
--------TTTT--ATCTTTGG-C-AAC-ATTGATA------TAAA-CTA-AAAG--TTA
-TT-TAAATCT--C----ATT--GTTT
>MG490370.1_Dictyostelium_multiforme_4007
TCTAAG-TATAAATTCTTG-TACGATGAAA----CTGCAGACGGCTCATT--ACAA--CA
---GT----GATAAACTA-ATAGACTTTCGG-GTTTTT----AACCTTTT-GGATAACCG
CAGTAAATCGGGGCTAATACATAGAAG-CG-ATGGG-TGACTGGC-----AAC-G-G-AA
GCTCAGCGATTATT----A-GCA-TTA-CTA-CCAATAC--CTTC--GGGTCTTGT-GGT
GAAACCGAATAATATTGCAGATCGAGG--------ATT-TATCTTCGACAAGTCTACTGT
GTCACTGCCCTATCAACTTTCGATGGTACGGTATTGGCCTACCATGGTTGTAACGGGTAA
CGGGGAATTAGGGTTCGATTCCGGAGAGGGAGCCTGAGAAATGGCTACCACTTCTACGGA
AGGCAGCAGGCGCGCAAATT-ACTCAATCCCAATAC-GGGGAAGTAGTGACAATAAATAT
CAATACCTATCC--TT-AACGGAGG--GCAATTGAAATGAACACAAATTAAAACTCTTAA
TT-AACACAATTGGAGGGCAAGTCTGGTGCCAGCAGCCGCGGTAATTCCAGCTCCAA-TA
GCATATACTAAAGTTGTTGCAGTTAAAA-AGC-TCGTAGTTGAAGTTAAAGGTTTACT-G
GG-CTAA----------AGTTATTTG-CCGCTCT----------GGTGGTTAAAT----C
AACTCCAGTATCTCTTTTT-TT-----AATAG-TTCAGCTTCTATTATCTT-------TG
ATAGTAGTTGTTTG---------------GACATTTCACTGTGAGAAAA-TTGTGGTGTT
TAAAGCA--GG-C---GTT---TC-GCCTGATCTTTTGCAGCATGGTATGATGAAACATG
ACAT---TTTACGC--TATTGGTTT--GCG----TCTAAAGTGTAATGATTAATAGGGAT
GGATGGGGGTGTTCATATTGGTGGGCGAGAGGTGAAATTCGTTGACCCTATCAAGATGAA
CTTCTGCGAAAGCATTCACCAAATACTTCCCCATTAATCAAGAACGAAAGTTTGGGGATC
GAAGACGATCAGATACCGTCGTAGTCCAAACTATAAACTATGTCGACCAGGGATCGGTTA

AA--AT-TTTTTTAAAATTTAATCGGCACCTT-GTGAGAAATCAC-GAGTGTTTAGATTC
CGGGGGGAGTATGGTCGCAAGTCTGAAACTTAAAGGAATTGACGGAAGGGCACACAATGG
AGTGGAG-CCTGCGGCTTAATTT-GACTCAACTCGGGAAA-ACTTACCAAGCTAAGATAT
AGTAAGGATTGACAGA-CTAA-AAGATCTTTCATGATTCTATAAGTGGTGGTGCATGGTC
GTTCTTAG-TTGGTGGAGCGATTTGTCTGGTCAATTCCGATAACGGACGAGACCTCGACC
TGCTAACTAGTAGTATTTATTA-GTCAATATGGGCGATAGCTTCTCTGGGGTTTGG-AAT
GGTT----TC--GGCCATC----TCC-TGCTTCAAGGAG----TGTGTAGTCTGACTTGA
TAG--GTACGAATTA---AAAAA------CTTCTTAGAGGGACTACCT-GCCTCAAGCAG
GCGG-AAGTCCGAGGCAATAACAGGTCTGTGATGCCCTTAGATACCTT-GGGCCGCACGC
GCGCTACAATGTAGAAAACAAAAAGGT-----TCC--------TGGTCCGG-AAG---GA
TT-GGGTAATCATTTGAATTTTCTACGTAACTGGGATTG-ATCTTTGTAATTATTGATCA
TCAACGAGGAATTCCTTGTAAGCGTAGGTCATTACCCTATGCTGAATATGTCCCTGCCCT
TTGTACACACCGCCCGTCGCTCCTACCGATCGAATGATACGGTAAAGTTAACGGATCG--
--------TTTT--ATCTTTGG-C-AAC-ATTGATA------TAAA-CTA-AAAG--TTA
-TT-TAAATCT--C----ATT--GTTT
>MG490369.1_Dictyostelium_minimum
TCTAAG-TATAAATTCTTG-TACGATGAAA----CTGCAGACGGCTCATT--ACAA--CA
---GT----GATAAACTA-ATAGACTTTCGG-GTTTTT----AACCTTTT-GGATAACCG
CAGTAAATCGGGGCTAATACATAGAAG-CG-ATGGG-TGACTGGC-----AAC-G-G-AA
GCTCAGCGATTATT----A-GCA-TTA-CTA-CCAATAC--CTTC--GGGTCTTGT-GGT
GAAACCGAATAATATTGCAGATCGAGG--------ATT-TATCTTCGACAAGTCTACTGT
GTCACTGCCCTATCAACTTTCGATGGTACGGTATTGGCCTACCATGGTTGTAACGGGTAA
CGGGGAATTAGGGTTCGATTCCGGAGAGGGAGCCTGAGAAATGGCTACCACTTCTACGGA
AGGCAGCAGGCGCGCAAATT-ACTCAATCCCAATAC-GGGGAAGTAGTGACAATAAATAT
CAATACCTATCC--TT-AACGGAGG--GCAATTGAAATGAACACAAATTAAAACTCTTAA
TT-AACACAATTGGAGGGCAAGTCTGGTGCCAGCAGCCGCGGTAATTCCAGCTCCAA-TA
GCATATACTAAAGTTGTTGCAGTTAAAA-AGC-TCGTAGTTGAAGTTAAAGGTTTACT-G
GG-CTAA----------AGTTATTTG-CCGCTCT----------GGTGGTTAAAT----C
AACTCCAGTATCTCTTTTT-TT-----AATAG-TTCAGCTTCTATTATCTT-------TG
ATAGTAGTTGTTTG---------------GACATTTCACTGTGAGAAAA-TTGTGGTGTT
TAAAGCA--GG-C---GTT---TC-GCCTGATCTTTTGCAGCATGGTATGATGAAACATG
ACAT---TTTACGC--TATTGGTTT--GCG----TCTAAAGTGTAATGATTAATAGGGAT
GGATGGGGGTGTTCATATTGGTGGGCGAGAGGTGAAATTCGTTGACCCTATCAAGATGAA
CTTCTGCGAAAGCATTCACCAAATACTTCCCCATTAATCAAGAACGAAAGTTTGGGGATC
GAAGACGATCAGATACCGTCGTAGTCCAAACTATAAACTATGTCGACCAGGGATCGGTTA
AA--AT-TTTTTTAAAATTTAATCGGCACCTT-GTGAGAAATCAC-GAGTGTTTAGATTC
CGGGGGGAGTATGGTCGCAAGTCTGAAACTTAAAGGAATTGACGGAAGGGCACACAATGG
AGTGGAG-CCTGCGGCTTAATTT-GACTCAACTCGGGAAA-ACTTACCAAGCTAAGATAT
AGTAAGGATTGACAGA-CTAA-AAGATCTTTCATGATTCTATAAGTGGTGGTGCATGGTC
GTTCTTAG-TTGGTGGAGCGATTTGTCTGGTCAATTCCGATAACGGACGAGACCTCGACC
TGCTAACTAGTAGTATTTATTA-GTCAATATGGGCGATAGCTTCTCTGGGGTTTGG-AAT
GGTT----TC--GGCCATC----TCC-TGCTTCAAGGAG----TGTGTAGTCTGACTTGA
TAG--GTACGAATTA---AAAAA------CTTCTTAGAGGGACTACCT-GCCTCAAGCAG
GCGG-AAGTCCGAGGCAATAACAGGTCTGTGATGCCCTTAGATACCTT-GGGCCGCACGC
GCGCTACAATGTAGAAAACAAAAAGGT-----TCC--------TGGTCCGG-AAG---GA
TT-GGGTAATCATTTGAATTTTCTACGTAACTGGGATTG-ATCTTTGTAATTATTGATCA
TCAACGAGGAATTCCTTGTAAGCGTAGGTCATTACCCTATGCTGAATATGTCCCTGCCCT
TTGTACACACCGCCCGTCGCTCCTACCGATCGAATGATACGGTAAAGTTAACGGATCG--
--------TTTT--ATCTTTGG-C-AAC-ATTGATA------TAAA-CTA-AAAG--TTA
-TT-TAAATCT--C----ATT--GTTT
>AM168053.1_Dictyostelium_mucoroides_TNS_C_114
TCTAAG-TATAAATTCTTG-TACGATGAAA----CTGCAGACGGCTCATT--ACAA--CA
---GT----GATAAACTA-ATAGACTTTCGG-GTTTTT----AACCTTTT-GGATAACCG
CAGTAAATCGGGGCTAATACATAGAAG-CG-ATGGG-TGACTGGC-----AAC-G-G-AA
GCTCAGCGATTATT----A-GCA-TTA-CTA-CCAATAC--CTTC--GGGTCTTGT-GGT
GAAACCGAATAATATTGCAGATCGAGG--------ATT-TATCTTCGACAAGTCTACTGT
GTCACTGCCCTATCAACTTTCGATGGTACGGTATTGGCCTACCATGGTTGTAACGGGTAA
CGGGGAATTAGGGTTCGATTCCGGAGAGGGAGCCTGAGAAATGGCTACCACTTCTACGGA
AGGCAGCAGGCGCGCAAATT-ACTCAATCCCAATAC-GGGGAAGTAGTGACAATAAATAT
CAATACCTATCC--TT-AACGGAGG--GCAATTGAAATGAACACAAATTAAAACTCTTAA
TT-AACACAATTGGAGGGCAAGTCTGGTGCCAGCAGCCGCGGTAATTCCAGCTCCAA-TA
GCATATACTAAAGTTGTTGCAGTTAAAA-AGC-TCGTAGTTGAAGTTAAAGGTTTACT-G
GG-CTAA----------AGTTATTTG-CCGCTCT----------GGTGGTTAAAT----C
AACTCCAGTATCTCTTTTT-TT-----AATAG-TTCAGCTTCTATTATCTT-------TG
ATAGTAGTTGTTTG---------------GACATTTCACTGTGAGAAAA-TTGTGGTGTT
TAAAGCA--GG-C---GTT---TC-GCCTGATCTTTTGCAGCATGGTATGATGAAACATG
ACAT---TTTACGC--TATTGGTTT--GCG----TCTAAAGTGTAATGATTAATAGGGAT
GGATGGGGGTGTTCATATTGGTGGGCGAGAGGTGAAATTCGTTGACCCTATCAAGATGAA
CTTCTGCGAAAGCATTCACCAAATACTTCCCCATTAATCAAGAACGAAAGTTTGGGGATC
GAAGACGATCAGATACCGTCGTAGTCCAAACT---------------------------
--TC
CGGGGGGAGTATGGTCGCAAGTCTGAAACTTAAAGGAATTGACGGAAGGGCACACAATGG
AGTGGAG-CCTGCGGCTTAATTT-GACTCAACTCGGGAAA-ACTTACCAAGCTAAGATAT

AGTAAGGATTGACAGA-CTAA-AAGATCTTTCATGATTCTATAAGTGGTGGTGCATGGTC
GTTCTTAG-TTGGTGGAGCGATTTGTCTGGTCAATTCCGATAACGGACGAGACCTCGACC
TGCTAACTAGTAGTATTTATTA-GTCAATATGGGCGATAGCTTCTCTGGGGTTTGG-AAT
GGTT----TC--GGCCATC----TCC-TGCTTCAAGGAG----TGTGTAGTCTGACTTGA
TAG--GTACGAATTA---AAAAA------CTTCTTAGAGGGACTACCT-GCCTCAAGCAG
GCGG-AAGTCCGAGGCAATAACAGGTCTGTGATGCCCTTAGATACCTT-GGGCCGCACGC
GCGCTACAATGTAGAAAACAAAAAGGT-----TCC--------TGGTCCGG-AAG---GA
TT-GGGTAATCATTTGAATTTTCTACGTAACTGGGATTG-ATCTTTGTAATTATTGATCA
TCAACGAGGAATTCCTTGTAAGCGTAGGTCATTACCCTATGCTGAATATGTCCCTGCCCT
TTGTACACACCGCCCGTCGCTCCTACCGATCGAATGATACGGTAAAGTTAACGGATCG--
--------TTTT--ATCTTTGG-C-AAC-ATTGATA------TAAA-CTA-AAAG--TTA
-TT-TAAATCT--C----ATT--GTTT
>AM168065.1_Dictyostelium_rosarium_M45
TCTAAG-TATAAATTCTTG-TACGATGAAA----CTGCAGACGGCTCATT--ACAA--CA
---GT----GATAAACTA-ATAGACTTTCGG-GTTTTT----AACCTTTT-GGATAACCG
CAGTAAATCGGGGCTAATACATAGAAG-CG-ATGGG-CGACTGGC-----AAC-G-G-AA
GCTCAGCGATTATT----A-GCA-TTA-CTA-CCAATAC--CTTC--GGGTCTTGT-GGT
GAAACCGAATAATATTGCAGATCGAGG--------ATT-TATCTTCGACAAGTCTACTGT
GTCACTGCCCTATCAACTTTCGATGGTACGGTATTGGCCTACCATGGTTGTAACGGGTAA
CGGGGAATTAGGGTTCGATTCCGGAGAGGGAGCCTGAGAAATGGCTACCACTTCTACGGA
AGGCAGCAGGCGCGCAAATT-ACTCAATCCCAATAC-GGGGAAGTAGTGACAATAAATAT
CAATACCTATCC--TT-AACGGAGG--GCAATTGAAATGAACACAAATTAAAACTCTTAA
TT-AACACAATTGGAGGGCAAGTCTGGTGCCAGCAGCCGCGGTAATTCCAGCTCCAA-TA
GCATATACTAAAGTTGTTGCAGTTAAAA-AGC-TCGTAGTTGAAGTTAAAGGTTTACT-G
GG-CTAA----------AGTTATTTG-CCGCTCT----------GGTGGTTAAAT----C
AACTCCAGTATCTCTTTTT-TT-----AATAG-TTCAGCTTCTATTATCTT-------TG
ATAGTAGTTGTTTG---------------GACATTTCACTGTGAGAAAA-TTGTGGTGTT
TAAAGCA--GG-C---GTT---TC-GCCTGATCTTTTGCAGCATGGTATGATGAAACATG
ACAT---TTTACGC--TATTGGTTT--GCG----TCTAAAGTGTAATGATTAATAGGGAT
GGATGGGGGTGTTCATATTGGTGGGCGAGAGGTGAAATTCGTTGACCCTATCAAGATGAA
CTTCTGCGAAAGCATTCACCAAATACTTCCCCATTAATCAAGAACGAAAGTTTGGGGATC
GAAGACGATCAGATACCGTCGTAGTCCAAACTATAAACTATGTCGACCAGGGATCGGTTA
AA--AT-TTTTTTAAAATTTAATCGGCACCTT-GTGAGAAATCAC-GAGTGTTTAGATTC
CGGGGGGAGTATGGTCGCAAGTCTGAAACTTAAAGGAATTGACGGAAGGGCACACAATGG
AGTGGAG-CCTGCGGCTTAATTT-GACTCAACTCGGGAAA-ACTTACCAAGCTAAGATAT
AGTAAGGATTGACAGA-CTAA-AAGATCTTTCATGATTCTATAAGTGGTGGTGCATGGTC
GTTCTTAG-TTGGTGGAGCGATTTGTCTGGTCAATTCCGATAACGGACGAGACCTCGACC
TGCTAACTAGTAGTATTTATTA-GTCAATATGGGCGATAGCTTCTCTGGGGTTTGG-AAT
GGCT----TC--GGTCATC----TCC-TGCTTCAAGGAG----TGTGTAGTCTGACTTGA
TAG--GTACGAATTA---AAAAA------CTTCTTAGAGGGACTACCT-GCCTCAAGCAG
GCGG-AAGTCCGAGGCAATAACAGGTCTGTGATGCCCTTAGATACCTT-GGGCCGCACGC
GCGCTACAATGTAGAAAACAAAAAGGT-----TCC--------TGGTCCGG-AAG---GA
TT-GGGTAATCATTTGAATTTTCTACGTAACTGGGATTG-ATCTTTGTAATTATTGATCA
TCAACGAGGAATTCCTTGTAAGCGTAGGTCATTACCCTATGCTGAATATGTCCCTGCCCT
TTGTACACACCGCCCGTCGCTCCTACCGATCGAATGATACGGTAAAGTTAACGGATCG--
--------TTTT--ATCTTTGG-C-AAC-ATTGATA------TAAA-CTA-AAAG--TTA
-TT-TAAATCT--C----ATT--GTTT
>AM168068.1_Dictyostelium_sphaerocephalum_GR11
TCTAAG-TATAAATTCTTG-TACGATGAAA----CTGCAGACGGCTCATT--ACAA--CA
---GT----GATAAACTA-ATAGACTTTCGG-GTTTTT----AACCTTTT-GGATAACCG
CAGTAAATCGGGGCTAATACATAGAAG-CG-ATGGG-TGACTGGC-----AAC-G-G-AA
GCTCAGCGATTATT----A-GCA-TTA-CTA-CCGATAC--CTTC--GGGTCTTGT-GGT
GAAACCGAATAATATTGCAGATCGAGG--------ATT-TATCTTCGACAAGTCTACTGT
GTCACTGCCCTATCAACTTTCGATGGTACGGTATTGGCCTACCATGGTTGTAACGGGTAA
CGGGGAATTAGGGTTCGATTCCGGAGAGGGAGCCTGAGAAATGGCTACCACTTCTACGGA
AGGCAGCAGGCGCGCAAATT-ACTCAATCCCAATAC-GGGGAAGTAGTGACAATAAATAT
CAATACCTATCC--TT-AACGGAGG--GCAATTGAAATGAACACAAATTAAAACTCTTAA
TT-AACACAATTGGAGGGCAAGTCTGGTGCCAGCAGCCGCGGTAATTCCAGCTCCAA-TA
GCATATACTAAAGTTGTTGCAGTTAAAA-AGC-TCGTAGTTGAAGTTAAAGGTTTACT-G
GG-CTAA----------AGTTATTTG-CCGCTCT----------GGTGGTTAAAT----C
AACTCCAGTATCTCTTTTT-TT-----AATAG-TTCAGCTTCTATTATCTT-------TG
ATAGTAGTTGTTTG---------------GACATTTCACTGTGAGAAAA-TTGTGGTGTT
TAAAGCA--GG-C---GTT---TC-GCCTGATCTTTTGCAGCATGGTATGATGAAACATG
ACAT---TTTACGC--TATTGGTTT--GCG----TCTAAAGTGTAATGATTAATAGGGAT
GGATGGGGGTGTTCATATTGGTGGGCGAGAGGTGAAATTCGTTGACCCTATCAAGATGAA
CTTCTGCGAAAGCATTCACCAAATACTTCCCCATTAATCAAGAACGAAAGTTTGGGGATC
GAAGACGATCAGATACCGTCGTAGTCCAAACTATAAACTATGTCGACCAGGGATCGGTTA
AA--AT-CTTTTTAAAATTTAATCGGCACCTT-GTGAGAAATCAC-GAGTGTTTAGATTC
CGGGGGGAGTATGGTCGCAAGTCTGAAACTTAAAGGAATTGACGGAAGGGCACACAATGG
AGTGGAG-CCTGCGGCTTAATTT-GACTCAACTCGGGAAA-ACTTACCAAGCTAAGATAT
AGTAAGGATTGACAGA-CTAA-AAGATCTTTCATGATTCTATAAGTGGTGGTGCATGGTC
GTTCTTAG-TTGGTGGAGCGATTTGTCTGGTCAATTCCGATAACGGACGAGACCTCGACC
TGCTAACTAGTAGTATTTATTA-GTCAATATGGGCGATAGCTTCTCTGGGGTTTGG-AAT

GGTT----TC--GGCCATC----TCC-TGCTTCAAGGAG----TGTGTAGTCTGACTTGA
TAG--GTACGAATTA---AAAAA------CTTCTTAGAGGGACTACCT-GCCTCAAGCAG
GCGG-AAGTCCGAGGCAATAACAGGTCTGTGATGCCCTTAGATACCTT-GGGCCGCACGC
GCGCTACAATGTAGAAAACAAAAAGGT-----TCC--------TGGTCCGG-AAG---GA
TT-GGGTAATCATTTGAATTTTCTACGTAACTGGGATTG-ATCTTTGTAATTATTGATCA
TCTACGAGGAATTCCTTGTAAGCGTAGGTCATTACCCTATGCTGAATATGTCCCTGCCCT
TTGTACACACCGCCCGTCGCTCCTACCGATCGAATGATACGGTAAAGTTAACGGATCG--
--------TTTT--ATCTTTGG-C-AAC-ATTGATA------TAAA-CTA-AAAG--TTA
-TT-TAAATCT--C----ATT--GTTT
>HQ141479.1_Dictyostelium_quercibrachium_NZ201B
TCTAAG-TATAAATTCTTG-TACGATGAAA----CTGCAGACGGCTCATT--ACAA--CA
---GT----GATAAACTA-ATAGACTTTCGG-GTTTTT----AACCTTTT-GGATAACCG
CAGTAAATCGGGGCTAATACATAGAAG-CG-ATGGG-TGACTGGC-----AAC-G-G-AA
GCTCAGCGATTATT----A-GCA-TTA-CTA-CCAATAC--CTTC--GGGTCTTGT-GGT
GAAACCGAATAATATTGCAGATCGAGG--------ATT-TATCTTCGACAAGTCTACTGT
GTCACTGCCCTATCAACTTTCGATGGTACGGTATTGGCCTACCATGGTTGTAACGGGTAA
CGGGGAATTAGGGTTCGATTCCGGAGAGGGAGCCTGAGAAATGGCTACCACTTCTACGGA
AGGCAGCAGGCGCGCAAATT-ACTCAATCCCAATAC-GGGGAAGTAGTGACAATAAATAT
CAATACCTATCC--TT-AACGGAGG--GCAATTGAAATGAACACAAATTAAAACTCTTAA
TT-AACACAATTGGAGGGCAAGTCTGGTGCCAGCAGCCGCGGTAATTCCAGCTCCAA-TA
GCATATACTAAAGTTGTTGCAGTTAAAA-AGC-TCGTAGTTGAAGTTAAAGGTTTACT-G
GG-CTAA----------AGTTATTTG-CCGCTCT----------GGTGGTTAAAT----C
AACTCCAGTATCTCTTTTT-TT-----AATAG-TTCAGCTTCTATTATCTT-------TG
ATAGTAGTTGTTTG---------------GACATTTCACTGTGAGAAAA-TTGTGGTGTT
TAAAGCA--GG-C---GTT---TC-GCCTGATCTTTTGCAGCATGGTATGATGGAACATG
ACAT---TTTACGC--TATTGGTTT--GCG----TCTAAAGTGTAATGATTAATAGGGAT
GGATGGGGGTGTTCATATTGGTGGGCGAGAGGTGAAATTCGTTGACCCTATCAAGATGAA
CTTCTGCGAAAGCATTCACCAAATACTTCCCCATTAATCAAGAACGAAAGTTTGGGGATC
GAAGACGATCAGATACCGTCGTAGTCCAAACTATAAACTATGTCGACCAGGGATCGGTTA
AA--AT-TTTTTTAAAATTTAATCGGCACCTT-GTGAGAAATCAC-GAGTGTTTAGATTC
CGGGGGGAGTATGGTCGCAAGTCTGAAACTTAAAGGAATTGACGGAAGGGCACACAATGG
AGTGGAG-CCTGCGGCTTAATTT-GACTCAACTCGGGAAA-ACTTACCAAGCTAAGATAT
AGTAAGGATTGACAGA-CTAA-AAGATCTTTCATGATTCTATAAGTGGTGGTGCATGGTC
GTTCTTAG-TTGGTGGAGCGATTTGTCTGGTCAATTCCGATAACGGACGAGACCTCGACC
TGCTAACTAGTAGTATTTATTA-GTCAATATGGGCGATAGCTTCTCTGGGGTTTGG-AAT
GGTT----TC--GGCCATC----TCC-TGCTTCAAGGAG----TGTGTAGTCTGACTTGA
TAG--GTACGAATTA---AAAAA---A--CTTCTTAGAGGGACTACCT-GCCTCAAGCAG
GCGG-AAGTCCGAGGCAATAACAGGTCTGTGATGCCCTTAGATACCTT-GGGCCGCACGC
GCGCTACAATGTAGAAAACAAAAAGGT-----TCC--------TGGTCCGG-AAG---GA
TT-GGGTAATCAATTGAATTTTCTACGTAACTGGGATTG-ATCTTTGTAATTATTGATCA
TCAACGAGGAATTCCTTGTAAGCGTAGGTCATTACCCTATGCTGAATATGTCCCTGCCCT
TTGTACACACCGCCCGTCGCTCCTACCGATCGAATGATACGGTAAAGTTAACGGATCG--
--------TTTT--ATCTGTGG-C-AAC-ACTGATA------TAAA-CTA-AAAG--TTA
-TT-TAAATCT--C----ATT--GTT-
>AM168034.1_Dictyostelium_clavatum_TNS_C_189
TCTAAG-TATAAATTTTTG-TACGATGAAA----CTGCAGACGGCTCATT--ACAA--CA
---GT----GATAAACTA-ATAGACTTTCGG-GTTTTT----AACCTTTT-GGATAACCG
CAGTAAATCGGGGCTAATACATAGAAG-CG-ATGGG-TGACTGGC-----AAC-G-G-AA
GCTCAGCGATTATT----A-GCA-TTA-CTA-CCAATAC--CTTC--GGGTCTTGT-GGT
GAAACCGAATAATATTGCAGATCGAGG--------ATT-TATCTTCGACAAGTCTACTGT
GTCACTGCCCTATCAACTTTCGATGGTACGGTATTGGCCTACCATGGTTGTAACGGGTAA
CGGGGAATTAGGGTTCGATTCCGGAGAGGGAGCCTGAGAAATGGCTACCACTTCTACGGA
AGGCAGCAGGCGCGCAAATT-ACTCAATCCCAATAC-GGGGAAGTAGTGACAATAAATAT
CAATACCTATCC--TT-AACGGAGG--GCAATTGAAATGAACACAAATTAAAACTCTTAA
TT-AACACAATTGGAGGGCAAGTCTGGTGCCAGCAGCCGCGGTAATTCCAGCTCCAA-TA
GCATATACTAAAGTTGTTGCAGTTAAAA-AGC-TCGTAGTTGAAGTTAAAGGTTTACT-G
GG-CTAA----------AGTTATTTG-CCGCTCT----------GGTGGTTAAAT----C
AACTCCAGTATCTCTTTTT-TT-----AATAG-TTCAGCTTCTATTATCTT-------TG
ATAGTAGTTGTTTG---------------GACATTTCACTGTGAGAAAA-TTGTGGTGTT
TAAAGCA--GG-C---GTT---TC-GCCTGATCTTTTGCAGCATGGTATGATGGAACATG
ACAT---TTTACGC--TATTGGTTT--GCG----TCTAAAGTGTAATGATTAATAGGGAT
GGATGGGGGTGTTCATATTGGTGGGCGAGAGGTGAAATTCGTTGACCCTATCAAGATGAA
CTTCTGCGAAAGCATTCACCAAATACTTCCCCATTAATCAAGAACGAAAGTTTGGGGATC
GAAGACGATCAGATACCGTCGTAGTCCAAACTATAAACTATGTCGACCAGGGATCGGTTA
AA--AT-TTTTTTAAAATTTAATCGGCACCTT-GTGAGAAATCAC-GAGTGTTTAGATTC
CGGGGGGAGTATGGTCGCAAGTCTGAAACTTAAAGGAATTGACGGAAGGGCACACAATGG
AGTGGAG-CCTGCGGCTTAATTT-GACTCAACTCGGGAAA-ACTTACCAAGCTAAGATAT
AGTAAGGATTGACAGA-CTAA-AAGATCTTTCATGATTCTATAAGTGGTGGTGCATGGTC
GTTCTTAG-TTGGTGGAGCGATTTGTCTGGTCAATTCCGATAACGGACGAGACCTCGACC
TGCTAACTAGTAGTATTTATTA-GTCAATATGGGCGATAGCTTCTCTGGGGTTTGG-AAT
GGTT----TC--GGCCATC----TCC-TGCTTCAAGGAG----TGTGTAGTCTGACTTGA
TAG--GTACGAATTA---AAAAA------CTTCTTAGAGGGACTACCT-GCCTCAAGCAG
GCGG-AAGTCCGAGGCAATAACAGGTCTGTGATGCCCTTAGATACCTT-GGGCCGCACGC

GCGCTACAATGTAGAAAACAAAAAGGT-----TCC--------TGGTCCGG-AAG---GA
TT-GGGTAATCAATTGAATTTTCTACGTAACTGGGATTG-ATCTTTGTAATTATTGATCA
TCAACGAGGAATTCCTTGTAAGCGTAGGTCATTACCCTATGCTGAATATGTCCCTGCCCT
TTGTACACACCGCCCGTCGCTCCTACCGATCGAATGATACGGTAAAGTTAACGGATCG--
--------TTTT--ATCTGTGG-C-AAC-ACTGATA------TAAA-CTA-AAAG--TTA
-TT-TAAATCT--C----ATT--GTTT
>AM168035.1_Dictyostelium_clavatum_TNS_C_220
TCTAAG-TATAAATTCTTG-TACGATGAAA----CTGCAGACGGCTCATT--ACAA--CA
---GT----GATAAACTA-ATAGACTTTCGG-GTTTTT----AACCTTTT-GGATAACCG
CAGTAAATCGGGGCTAATACATAGAAG-CG-ATGGG-TGACTGGC-----AAC-G-G-AA
GCTCAGCGATTATT----A-GCA-TTA-CTA-CCAATAC--CTTC--GGGTCTTGT-GGT
GAAACCGAATAATATTGCAGATCGAGG--------ATT-TATCTTCGACAAGTCTACTGT
GTCACTGCCCTATCAACTTTCGATGGTACGGTATTGGCCTACCATGGTTGTAACGGGTAA
CGGGGAATTAGGGTTCGATTCCGGAGAGGGAGCCTGAGAAATGGCTACCACTTCTACGGA
AGGCAGCAGGCGCGCAAATT-ACTCAATCCCAATAC-GGGGAAGTAGTGACAATAAATAT
CAATCCCTATCC--TT-AACGGAGG--GCAATTGAAATGAACACAAATTAAAACTCTTAA
TT-AACACAATTGGAGGGCAAGTCTGGTGCCAGCAGCCGCGGTAATTCCAGCTCCAA-TA
GCATATACTAAAGTTGTTGCAGTTAAAA-AGC-TCGTAGTTGAAGTTAAAGGTTTACT-G
GG-CTAA----------AGTTATTTG-CCGCTCT----------GGTGGTTAAAT----C
AACTCCAGTATCTCTTTTT-TT-----AATAG-TTCAGCTTCTATTATCTT-------TG
ATAGTAGTTGTTTG---------------GACATTTCACTGTGAGAAAA-TTGTGGTGTT
TAAAGCA--GG-C---GTT---TC-GCCTGATCTTTTGCAGCATGGTATGATGGAACATG
ACAT---TTTACGC--TATTGGTTT--GCG----TCTAAAGTGTAATGATTAATAGGGAT
GGATGGGGGTGTTCATATTGGTGGGCGAGAGGTGAAATTCGTTGACCCTATCAAGATGAA
CTTCTGCGAAAGCATTCACCAAATACTTCCCCATTAATCAAGAACGAAAGTTTGGGGATC
GAAGACGATCAGATACCGTCGTAGTCCAAACTATAAACTATGTCGACCAGGGATCGGTTA
AA--AT-TTTTTTAAAATTTAATCGGCACCTT-GTGAGAAATCAC-GAGTGTTTAGATTC
CGGGGGGAGTATGGTCGCAAGTCTGAAACTTAAAGGAATTGACGGAAGGGCACACAATGG
AGTGGAG-CCTGCGGCTTAATTT-GACTCAACTCGGGAAA-ACTTACCAAGCTAAGATAT
AGTAAGGATTGACAGA-CTAA-AAGATCTTTCATGATTCTATAAATGGTGGTGCATGGTC
GTTCTTAG-TTGGTGGAGCGATTTGTCTGGTCAATTCCGATAACGGACGAGACCTCGACC
TGCTAACTAGTAGTATTTATTA-GTCAATATGGGCGATAGCTTCTCTGGGGTTTGG-AAT
GGCT----GC--GGTCATC----TCC-TGCTTCAAGGAG----TGTGTAGTCTGACTTGA
TAG--GTACGAATTA---AAAAA------CTTCTTAGAGGGACTACCT-GCCTCAAGCAG
GCGG-AAGTCCGAGGCAATAACAGGTCTGTGATGCCCTTAGATACCTT-GGGCCGCACGC
GCGCTACAATGTAGAAAACAAAAAGGT-----TCC--------TGGTCCGG-AAG---GA
TT-GGGTAATCAATTGAATTTTCTACGTAACTGGGATTG-ATCTTTGTAATTATTGATCA
TCAACGAGGAATTCCTTGTAAGCGTAGGTCATTACCCTATGCTGAATATGTCCCTGCCCT
TTGTACACACCGCCCGTCGCTCCTACCGATCGAATGATACGGTAAAGTTAACGGATCG--
--------TTTT--ATCTGTGG-C-AAC-ACTGATA------TAAA-CTA-AAAG--TTA
-TT-TAAATCT--C----ATT--GTTT
>AM168048.1_Dictyostelium_longosporum_TNS_C_109
TCTAAG-TATAAATTCTTG-TACGATGAAA----CTGCAGACGGCTCATT--ACAA--CA
---GT----GATAAACTA-ATAGACTTTCGG-GTTTTT----AACCTTTT-GGATAACCG
CAGTAAATCGGGGCTAATACATAGAAG-CG-ATGGG-TGACTGGC-----AAC-G-G-AA
GCTCAGCGATTATT----A-GCA-TTA-CTA-CCAATAC--CTTC--GGGTCTTGT-GGT
GAAACCGAATAATATTGCAGATCGAGG--------ATT-TATCTTCGACAAGTCTACTGT
GTCACTGCCCTATCAACTTTCGATGGTACGGTATTGGCCTACCATGGTTGTAACGGGTAA
CGGGGAATTAGGGTTCGATTCCGGAGAGGGAGCCTGAGAAATGGCTACCACTTCTACGGA
AGGCAGCAGGCGCGCAAATT-ACTCAATCCCAATAC-GGGGAAGTAGTGACAATAAATAT
CAATACCTATCC--TT-AACGGAGG--GCAATTGAAATGAACACAAATTAAAACTCTTAA
TT-AACACAATTGGAGGGCAAGTCTGGTGCCAGCAGCCGCGGTAATTCCAGCTCCAA-TA
GCATATACTAAAGTTGTTGCAGTTAAAA-AGC-TCGTAGTTGAAGTTAAAGGTTTACT-G
GG-CTAA----------AGTTATTTG-CCGCTCT----------GGTGGTTAAAT----C
AACTCCAGTATCTCTTTTT-TT-----AATAG-TTCAGCTTCTATTATCTT-------TG
ATAGTAGTTGTTTG---------------GACATTTCACTGTGAGAAAA-TTGTGGTGTT
TAAAGCA--GG-C---GTT---TC-GCCTGATCTTTTGCAGCATGGTATGATGGAACATG
ACAT---TTTACGC--TATTGGTTT--GCG----TCTAAAGTGTAATGATTAATAGGGAT
GGATGGGGGTGTTCATATTGGTGGGCGAGAGGTGAAATTCGTTGACCCTATCAAGATGAA
CTTCTGCGAAAGCATTCACCAAATACTTCCCCATTCATCAAGAACGAAAGTTTGGGGATC
GAAGACGATCAGATACCGTCGTAGTCCAAACTATAAACTATGTCGACCAGGGATCGGTTA
AA--AT-TTTTTTACAATTTAATCGGCACCTT-GTGAGAAATCAC-GAGTGTTTAGATTC
CGGGGGGAGTATGGTCGCAAGTCTGAAA-TTAAAGGAATTGACGGAAGGGCACACAATGG
AGTGGAG-CCTGCGGCTTAATTT-GACTCAACTCGGGAAA-ACTTACCAAGCTAAGATAT
AGTAAGGATTGACAGA-CTAA-AAGATCTTTCATGATTCTATAAGTGGTGGTGCATGGTC
GTTCTTAG-TTGGTGGAGCGATTTGTCTGGTCAATTCCGATAACGGACGAGACCTCGACC
TGCTAACTAGTAGTATTTATTA-GTCAATATGGGCGATAGCTTCTCTGGGGTTTGG-AAT
GGCT----TC--GGTCATC----TCC-TGCTTCAAGGAG----TGTGTAGTCTGACTTGA
TAG--GTACGAATTA---AAAAA------CTTCTTAGAGGGACTACCT-GCCTCAAGCAG
GCGG-AAGTCCGAGGCAATAACAGGTCTGTGATGCCCTTAGATACCTT-GGGCCGCACGC
GCGCTACAATGTAGAAAACAAAAAGGT-----TCC--------TGGTCCGG-AAG---GA
TT-GGGTAATCAATTGAATTTTCTACGTAACTGGGATTG-ATCTTTGTAATTATTGATCA
TCAACGAGGAATTCCTTGTAAGCGTAGGTCATTACCCTATGCTGAATATGTCCCTGCCCT

TTGTACACACCGCCCGTCGCTCCTACCGATCGAATGATACGGTAAAGTTAACGGATCG--
--------TTTT--ATCTGTGG-C-AAC-ACTGATA------TAAA-CTA-AAAG--TTA
-TT-TAAATCT--C----ATT--GTTT
>HQ141478.1_Dictyostelium_ammophilum_KBK4A
TCTAAG-TATAAATTCTTG-TACGATGAAA----CTGCAGACGGCTCATT--ACAA--CA
---GT----GATAAACTA-ATAGACTTTCGG-GTTTTT----AACCTTTT-GGATAACCG
CAGTAAATCGGGGCTAATACATAGAAG-CG-ATGGG-TGACTGGC-----AAC-G-G-AA
GCTCAGCGATTATT----A-GCA-TTA-CTA-CCAATAC--CTTC--GGGTCTTGT-GGT
GAAACCGAATAATATTGCAGATCGAGG--------ATT-TATCTTCGACAAGTCTACTGT
GTCACTGCCCTATCAACTTTCGATGGTACGGTATTGGCCTACCATGGTTGTAACGGGTAA
CGGGGAATTAGGGTTCGATTCCGGAGAGGGAGCCTGAGAAATGGCTACCACTTCTACGGA
AGGCAGCAGGCGCGCAAATT-ACTCAATCCCAATAC-GGGGAAGTAGTGACAATAAATAT
CAATACCTATCC--TT-AACGGAGG--GCAATTGAAATGAACACAAATTAAAACTCTTAA
TT-AACACAATTGGAGGGCAAGTCTGGTGCCAGCAGCCGCGGTAATTCCAGCTCCAA-TA
GCATATACTAAAGTTGTTGCAGTTAAAA-AGC-TCGTAGTTGAAGTTAAAGGTTTACT-G
GG-CTAA----------AGTTATTTG-CCGCTCT----------GGTGGTTAAAT----A
TACTCCAGTATCTCTTTTT-TT-----AATAG-TTCAGCTTCTATTATCTT-------TG
ATAGTAGTTGTTTG---------------GACATTTCACTGTGAGAAAA-TTGTGGTGTT
TAAAGCA--GG-C---GTT---TC-GCCTGATCTTTTGCAGCATGGTATGATGGAACATG
ACAT---TTTACGC--TATTGGTTT--GCG----TCTAAAGTGTAATGATTAATAGGGAT
GGATGGGGGTGTTCATATTGGTGGGCGAGAGGTGAAATTCGTTGACCCTATCAAGATGAA
CTTCTGCGAAAGCATTCACCAAATACTTCCCCATTAATCAAGAACGAAAGTTTGGGGATC
GAAGACGATCAGATACCGTCGTAGTCCAAACTATAAACTATGTCGACCAGGGATCGGTTA
AA--AT-TTTTTTAAAATTTAATCGGCACCTT-GTGAGAAATCAC-GAGTGTTTAGATTC
CGGGGGGAGTATGGTCGCAAGTCTGAAACTTAAAGGAATTGACGGAAGGGCACACAATGG
AGTGGAG-CCTGCGGCTTAATTT-GACTCAACTCGGGAAA-ACTTACCAAGCTAAGATAT
AGTAAGGATTGACAGA-CTAA-AAGATCTTTCATGATTCTATAAGTGGTGGTGCATGGTC
GTTCTTAG-TTGGTGGAGCGATTTGTCTGGTCAATTCCGATAACGGACGAGACCTCGACC
TGCTAACTAGTAGTATTTATTA-GTCAATATGGGCGATAGCTTTTCTGGGGTTTGG-AAT
GATT----TC--GGTCATC----TCC-TGCTTCAAGGAG----TGTGTAGTCTGACTTGA
TAG--GTACGAATTA---AAAAA------CTTCTTAGAGGGACTACCT-GCCTCAAGCAG
GCGG-AAGTCCGAGGCAATAACAGGTCTGTGATGCCCTTAGATACCTT-GGGCCGCACGC
GCGCTACAATGTAGAAAACAAAAAGGT-----TCC--------TGGTCCGG-AAG---GA
TT-GGGTAATCAATTGAATTTTCTACGTAACTGGGATTG-ATCTTTGTAATTATTGATCA
TCAACGAGGAATTCCTTGTAAGCGTAGGTCATTACCCTATGCTGAATATGTCCCTGCCCT
TTGTACACACCGCCCGTCGCTCCTACCGATCGAATGATACGGTAAAGTTAACGGATCG--
--------TTTT--ATCTGTGG-C-AAC-ACTGATA------TAAA-CTA-AAAG--TTA
-TT-TAAATCT--C----AT---GTT-
>GQ496155.1_Dictyostelium_valdivianum
TCTAAG-TATAAATTCTTG-TACGATGAAA----CTGCAGACGGCTCATT--ACAA--CA
---GT----GATAAACTA-ATAGACTTTCGG-GTTTTT----AACCTTTT-GGATAACCG
CAGTAAATCGGGGCTAATACATAGAAG-CG-ATGGG-TGACTGGC-----AAC-G-G-AA
GCTCAGCGATTATT----A-GCA-TTA-CTA-CCAATAC--CTTC--GGGTCTTGT-GGT
GAAACCGAATAATATTGCAGATCGAGG--------ATT-TATCTTCGACAAGTCTACTGT
GTCACTGCCCTATCAACTTTCGATGGTACGGTATTGGCCTACCATGGTTGTAACGGGTAA
CGGGGAATTAGGGTTCGATTCCGGAGAGGGAGCCTGAGAAATGGCTACCACTTCTACGGA
AGGCAGCAGGCGCGCAAATT-ACTCAATCCCAATAC-GGGGAAGTAGTGACAATAAATAT
CAATACCTATCC--TT-AACGGAGG--GCAATTGAAATGAACACAAATTAAAACTCTTAA
TT-AACACAATTGGAGGGCAAGTCTGGTGCCAGCAGCCGCGGTAATTCCAGCTCCAA-TA
GCATATACTAAAGTTGTTGCAGTTAAAA-AGC-TCGTAGTTGAAGTTAAAGGTTTACT-G
GG-CTAA----------AGTTATTTG-CCGCTCT----------GGTGGTTAAAT----C
AACTCCAGTATCTCTTTTT-TT-----AATAG-TTCAGCTTCTATTATCTT-------TG
ATAGTAGTTGTTTG---------------GACATTTCACTGTGAGAAAA-TTGTGGTGTT
TAAAGCA--GG-C---GTT---TC-GCCTGATCTTTTGCAGCATGGTATGATGGAACATG
ACAT---TTTACGC--TATTGGTTT--GCG----TCTAAAGTGTAATGATTAATAGGGAT
GGATGGGGGTGTTCATATTGGTGGGCGAGAGGTGAAATTCGTTGACCCTATCAAGATGAA
CTTCTGCGAAAGCATTCACCAAATACTTCCCCATTAATCAAGAACGAAAGTTTGGGGATC
GAAGACGATCAGATACCGTCGTAGTCCAAACTATAAACTATGTCGACCAGGGATCGGTTA
AA--AT-TTTTTTAAAATTTAATCGGCACCTT-GTGAGAAATCAC-GAGTGTTTAGATTC
CGGGGGGAGTATGGTCGCAAGTCTGAAACTTAAAGGAATTGACGGAAGGGCACACAATGG
AGTGGAG-CCTGCGGCTTAATTT-GACTCAACTCGGGAAA-ACTTACCAAGCTAAGATAT
AGTAAGGATTGACAGA-CTAA-AAGATCTTTCATGATTCTATAAGTGGTGGTGCATGGTC
GTTCTTAG-TTGGTGGAGCGATTTGTCTGGTCAATTCCGATAACGGACGAGACCTCGACC
TGCTAACTAGTAGTATTTATTA-GTCAATATGGGCGATAGCTTCTCTGGGGTTTGG-AAT
GACT----TC--GGTCATC----TCC-TGCTTCAAGGAG----TGTGTAGTCTGACTTGA
TAG--GTACGAATTA---AAAAA------CTTCTTAGAGGGACTACCT-GCCTCAAGCAG
GCGG-AAGTCCGAGGCAATAACAGGTCTGTGATGCCCTTAGATACCTT-GGGCCGCACGC
GCGCTACAATGTAGAAAACAAAAAGGT-----TCC--------TGGTCCGG-AAG---GA
TT-GGGTAATCAATTGAATTTTCTACGTAACTGGGATTG-ATCTTTGTAATTATTGATCA
TCAACGAGGAATTCCTTGTAAGCGTAGGTCATTACCCTATGCTGAATATGTCCCTGCCCT
TTGTACACACCGCCCGTCGCTCCTACCGATCGAATGATACGGTAAAGTTAACGGATCG--
--------TTTT--ATCTGTGG-C-AAC-ACTGATA------TAAA-CTA-AAAG--TTA
-TT-TAAATCT--C----AT---GTT-

>AM168066.1_Dictyostelium_septentrionale_IY49
TCTAAG-TATAAGTTCTTG-TACGATGAAA----CTGCAGACGGCTCATT--ACAA--CA
---GT----GATAAACTA-TTAGACTTTCGG-GTTTTT-----ACCTTTT-GGATAACCG
CAGTAAATCGGGGCTAATACATAGAAG-CG-ATGGG-TAACTGGC-----AAC-G-G-AA
GCTCAGCGATTATT----A-GCA-T---CTA-TCAATAC--CTTC--GGGTCTTGT-GGT
GAAACCGAATAATATTGCAGATCGAAG--------ATT-TATCTTCGACAAGTCTATTGT
GTCACTGCCCTATCAACTTTCGATGGTACGGTATTGGCCTACCATGGTTGTAACGGGTAA
CGGGGAATTAGGGTTCGATTCCGGAGAGGGAGCCTGAGAAATGGCTACCACTTCTACGGA
AGGCAGCAGGCGCGCAAATT-ACTCAATCCCAATAC-GGGGAAGTAGTGACAATAAATAT
CAATGCCTATCC--TT-AACGGAGG--GCAATTGAAATGAACACAAATTAAAACTCTTAA
TT-AACACAATTGGAGGGCAAGTCTGGTGCCAGCAGCCGCGGTAATTCCAGCTCCAA-TA
GCATATACTAAAGTTGTTGCAGTTAAAA-AGC-TCGTAGTTGAAGTCAAAGGTTTGTC-G
GG-TCAA----------AGTTGTTTCTCCGCTCT----------GGTGGTTAAAT----A
TACTCCGGCATCTCTTTTT-TT-----AATAG-TTCTGCTTGTGCTATCTT-------TG
ATGGTACTTGTTTG---------------AACAGTTCACCGTGAGAAAA-TTGTGGTGTT
CAAAGCA--GG-C---GTT---TC-GCCTGATCTTTTGCAGCATGGTATGATGAAACATG
ACAT---TTTACGC--TATTGGTTT--GCG----TCTAGAGTGTAATGATTAATAGGGAT
GGATGGGGGTGTTCATATTGGTGGGCGAGAGGTGAAATTCGTTGACCCTATCAAGATGAA
CTTCTGCGAAAGCATTCGCCAAATACTTCTCCATTAATCAAGAACGAAAGTTTGGGGATC
GAAGACGATCAGATACCGTCGTAGTCCAAACTATAAACTATGTCGACCAGGGATCGGTTA
AA--AT-TTTTTAAAATTTAATCGGCACCTT-GTGAGAAATCAC-GAGTGTTTAGATTC
CGGGGGGAGTATGGTCGCAAGTCTGAAACTTAAAGGAATTGACGGAAGGGCACACAATGG
AGTGGAG-CCTGCGGCTTAATTT-GACTCAACTCGGGAAA-ACTTACCAAGCTAAGATAT
AGTAAGGATTGACAGA-CTAA-AAGATCTTTCATGATTCTATAAGTGGTGGTGCATGGTC
GTTCTTAG-TTGGTGGAGCGATTTGTCTGGTCAATTCCGATAACGGACGAGACCTCGACC
TGCTAACTAGTAGTGTTTATTTCGCCAATATGGGCGATAGCTTTTTTGGGATTTCG-GTT
CATT----TC--GGTGGAT----TCG-GGTTTCAGGGAG----TGTGTAGTCTGGTGGGA
TAG--GCACGAATTC---AAAAA---A--CTTCTTAAAGGGACTACCT-GCCTCAAGCAG
GCGG-AAGTCCGAGGCAATAACAGGTCTGTGATGCCCTTAGATACCTT-GGGCCGCACGC
GCGCTACAATGTAGAAAACAAAAAGGT-----TCC--------TGGTCTGG-AAG---GA
TT-GGGTAATCAATTGAATTTTCTACGTAACTGGGATTG-ATCTTTGTAATTATCGATCA
TCAACGAGGAATTCCTTGTAAGCGTAGGTCATTACCCTATGCTGAATATGTCCCTGCCCT
TTGTACACACCGCCCGTCGCTCCTACCGATCGAATGATACGGTAAAGTTAACGGATTG--
--------TTTT--G-CTTT---CGTTATAGGGGCA------TTA--TGA-CAAG--TTA
-TT-TAAATCT--C----ATT--GTTT
>AM168067.1_Dictyostelium_septentrionale_AK2
TCTAAG-TATAAATTCTTG-TACGATGAAA----CTGCAGACGGCTCATT--ACAA--CA
---GT----GATAAACTA-TTAGACTTTCGG-GTTTTT-----ACCTTTT-GGATAACCG
CAGTAAATCGGGGCTAATACATAGAAG-CG-ATGGG-TAACTGGC-----AAC-G-G-AA
GCTCAGCGATTATT----A-GCA-T---CTA-TCAATAC--CTTC--GGGTCTTGT-GGT
GAAACCGAATAATATTGCAGATCGAAG--------ATT-TATCTTCGACAAGTCTATTGT
GTCACTGCCCTATCAACTTTCGATGGTACGGTATTGGCCTACCATGGTTGTAACGGGTAA
CGGGGAATTAGGGTTCGATTCCGGAGAGGGAGCCTGAGAAATGGCTACCACTTCTACGGA
AGGCAGCAGGCGCGCAAATT-ACTCAATCCCAATAC-GGGGAAGTAGTGACAATAAATAT
CAATGCCTATCC--TT-A-TGGAGG--GCAATTGAAATGAACACAAATTAAAACTCTTAA
TT-AACACAATTGGAGGGCAAGTCTGGTGCCAGCAGCCGCGGTAATTCCAGCTCCAA-TA
GCATATACTAAAGTTGTTGCAGTTAAAA-AGC-TCGTAGTTGAAGTCAAAGGTTTGTC-G
GG-TCAA----------AGTTGTTTTTCCGCTCT----------GGTGGT-AAAC----A
TACTCCGGCATCTCTTTTT-TT-----AATAG-TTCTGCTTGTGCTATCTT-------TG
ATGGTACTTGTTTG---------------AACAGTTCACCGTGAGAAAA-TTGTGGTGTT
CAAAGCA--GG-C---GTT---TC-GCCTGATCTTTTGCAGCATGGTATGATGAAACATG
ACAT---TTTACGC--TATTGGTTT--GCG----TCTAGAGTGTAATGATTAATAGGGAT
GGATGGGGGTGTTCATATTGGTGGGCGAGAGGTGAAATTCGTTGACCCTATCAAGATGAA
CTTCTGCGAAAGCATTCACCAAATACTTCTCCATTAATCAAGAACGAAAGTTTGGGGATC
GAAGACGATCAGATACCGTCGTAGTCCAAACTATAAACTATGTCGACCAGGGATCGGTTA
AA--AT-TTTTTTAAAATTTAATCGGCACCTT-GTGAGAAATCAC-GAGTGTTTAGATTC
CGGGGGGAGTATGGTCGCAAGTCTGAAACTTAAAGGAATTGACGGAAGGGCACACAATGG
AGTGGAG-CCTGCGGCTTAATTT-GACTCAACTCGGGAAA-ACTTACCAAGCTAAGATAT
AGTAAGGATTGACAGA-CTAA-AAGATCTTTCATGATTCTATAAGTGGTGGTGCATGGTC
GTTCTTAG-TTGGTGGAGCGATTTGTCTGGTCAATTCCGATAACGGACGAGACCTCGACC
TGCTAACTAGTAGTGTTTATTTCGCCAATATGGGCGATAGCTTTTTTGGGATTTCG-GTT
CATT----TC--GGTGGAT----TCG-GGTTTCAGGGAG----TGTGTAGTCTGGTGGGA
TAG--GCACGAATTC---AAAAA---A--CTTCTTAGAGGGACTACCT-GCCTCAAGCAG
GCGG-AAGTCCGAGGCAATAACAGGTCTGTGATGCCCTTAGATACCTT-GGGCCGCACGC
GCGCTACAATGTAGAAAACAAAAAGGT-----TCC--------TGGTCTGG-AAG---GA
TT-GGGTAATCAATTGAATTTTCTACGTAACTGGGATTG-ATCTTTGTAATTATCGATCA
TCAACGAGGAATTCCTTGTAAGCGTAGGTCATTACCCTATGCTGAATATGTCCCTGCCCT
TTGTACACACCGCCCGTCGCTCCTACCGATCGAATGATACGGTAAAGTTAACGGATTG--
--------TTTT--G-CTTT---CGTTATAGGGGCA------TTA--TGA-AAAG--TTA
-TT-TAAATCT--C----ATT--GTTT
>AM168037.1_Dictyostelium_crassicaule_93HO_33
TCTAAG-TATAAATTCTTG-TACGATGAAA----CTGCAGACGGCTCATT--ACAA--CA
---GT----GATAAACTA-ATAGACTTTCGG-GTTTTT----AACCTTTT-GGATAACCG

CAGTAAATCGGGGCTAATACATAGAAG-CG-ATGGG-TGACTGGC-----AAC-G-G-AA
TCTCAGCGATTATT----A-GCA-TAA-CTA-CCAATAC--CTTC--GGGTCTTGT-GGT
GAAACCGAATAATATTGCAGATCGAAG--------ATT-TATCTTCGACAAGTCTACTGT
GTCACTGCCCTATCAACTTTCGATGGTACGGTATTGGCCTACCATGGTTGTAACGGGTAA
CGGGGAATTAGGGTTCGATTCCGGAGAGGGAGCCTGAGAAATGGCTACCACTTCTACGGA
AGGCAGCAGGCGCGCAAATT-GCTCAATCCCAATAC-GGGGAAGTAGTGACAATAAATAT
CAATACCTATCC--TT-AATGGAGG--GCAATTGAAATGAACACAAATTAAAACTCTTAA
TT-AACACAGTTGGAGGGCAAGTCTGGTGCCAGCAGCCGCGGTAATTCCAGCTCCAA-TA
GCATATACTAAAGTTGTTGCAGTTAAAA-AGC-TCGTAGTTGAAGTTAAAGGTTTACT-G
GG-CTCA----------AGTTATTTG-CCACTCT----------GGTGGTTAAAT----A
TACTCCAGTATCTCTTTTT-TTTCT--AATAG-TTCAGCTTGTATCAACCT-------AG
TTGGTACTCGTTTG---------------GACATTTCACTGTGAGAAAA-TTGTGGTGTT
TAAAGCA--GG-C---GTC---TC-GCCTGATCTTTTGCAGCATGGTATGATGAAACATG
ACAT---TTTGCGC--TATTGGTTT--GCG----TCTAAAGTGTAATGATTAATAGGGAT
GGATGGGGGTGTTCATATTGGTGGGCGAGAGGTGAAACTCGTTGACCCTATCAAGATGAA
CTTCTGCGAAAGCATTCACCAAATACTTCCCCATTAATCAAGAACGAAAGTTTGGGGATC
GAAGACGATCAGATACCGTCGTAGTCCAAACTATAAACTATGTCGACCAGGGATCGGTTA
AA--AT-TTTTTTAAAATTTAATCGGCACCTT-GTGAGAAATCAC-GAGTGTTTAGATTC
CGGGGGGAGTATGGTCGCAAGTCTGAAACTTAAAGGAATTGACGGAAGGGCACACAATGG
AGTGGAG-CCTGCGGCTTAATTT-GACTCAACTCGGGAAA-ACTTACCAAGCTAAGATAT
AGTAAGGATTGACAGA-CTAA-AAGATCTTTCATGATTCTATAAGTGGTGGTGCATGGTC
GTTCTTAG-TTGGTGGAGCGATTTGTCTGGTCAATTCCGATAACGGACGAGACCTCGACC
TGCTAACTAGTAGTATTTATTA-GTCAATATGGGCGATAGCTTTTCTGGGGTGGAGTAAT
AGCC----TCACGGTTATT----ATT-TACTTCAAGGAG----TGTGTAGTCTGACTTGA
TAG--GTACGAATTA---AAAAA---A--CTTCTTAGAGGGACTACCT-GCCTCAAGCAG
GCGG-AAGTCCGAGGCAATAACAGGTCTGTGATGCCCTTAGATACCTT-GGGCCGCACGC
GCGCTACAATGTAGAAAACAAAAAGGT-----TCC--------TGGTCCGG-AAG---GA
TT-GGGTAATCAATTGAATTTTCTACGTAACTGGGATTG-ATCTTTGTAATTATTGATCA
TCAACGAGGAATTCCTTGTAAGCGTAGGTCATTACCCTATGCTGAATATGTCCCTGCCCT
TTGTACACACCGCCCGTCGCTCCTACCGATCGAATGATACGGTAAACTTAACGGATCG--
--------TTTT--ATCTTTGG-C-AAC-ATTGATA------TAAA-CTA-AAAG--TTA
-TT-TAAATCT--C----ATT--GTTT
>AM168050.1_Dictyostelium_medium_TNS_C_205
TCTAAG-TATAAATTCTTG-TACGATGAAA----CTGCAGACGGCTCATT--ACAA--CA
---GT----GATAAACTA-ATAGACTTTCGG-GTTTTT----AACCTTTT-GGATAACCG
CAGTAAATCGGGGCTAATACATAGAAG-CG-ATGGG-TGAC-GGC-----AAC-G---AA
GCTCAGCGATTATT----A-GCA-TTA-CTA-CCAATAC--CTTC--GGGTCTTGT-GGT
GAAACCGAATAATATTGCAGATCGAGG--------ATT-TATCTTCGACAAGTCTACTGT
GTCACTGCCCTATCAACTTTCGATGGTACGGTATTGGCCTACCATGGTTGTAACGGGTAA
CGGGGAATTAGGGTTCGATTCCGGAGAGGGAGCCTGAGAAATGGCTACCACTTCTACGGA
AGGCAGCAGGCGCGCAAATT-ACTCAATCCCAATAC-GGGGAAGTAGTGACAATAAATAT
CAATACCTATCC--TT-AATGGAGG--GCAATTGAAATGAACACAAATTAAAACTCTTAA
TT-AACACAATTGGAGGGCAAGTCTGGTGCCAGCAGCCGCGGTAATTCCAGCTCCAA-TA
GCATATACTAAAGTTGTTGCAGTTAAAA-AGC-TCGTAGTTGAAGTTAAAGGTTTACT-G
GG-CTAA----------AGTTATTTG-CCGCTCT----------GGTGGTTAAAT----C
AACTCCAGTATCTCTTTCG-CTC----AATAG-TTCAGCTTGTATCAACCT-------AG
TTGGTACTCGTTTG---------------GACATTTCACTGTGAGAAAA-TTGTGGTGTT
TAAAGCA--GG-C---GTC---TC-GCCTGATCTTTTGCAGCATGGTATGATGGAACATG
ACAT---TTTGCGC--TATTGGTTT--GCG----TCTAAAGTGTAATGATTAATAGGGAT
GGATGGGGGTGTTCATATTGGTGGGCGAGAGGTGAAATTCGTTGACCCTATCAAGATGAA
CTTCTGCGAAAGCATTCACCAAATACTTCCCCATTAATCAAGAACGAAAGTTTGGGGATC
GAAGACGATCAGATACCGTCGTAGTCCAAACTATAAACTATGTCGACCAGGGATCGGTTA
AA--AT-TTTTTTAAAATTTAATCGGCACCTT-GTGAGAAATCAC-GAGTGTTTAGATTC
CGGGGGGAGTATGGTCGCAAGTCTGAAACTTAAAGGAATTGACGGAAGGGCACACAATGG
AGTGGAG-CCTGCGGCTTAATTT-GACTCAACTCGGGAAA-ACTTACCAAGCTAAGATAT
AGTAAGGATTGACAGA-CTAA-AAGATCTTTCATGATTCTATAAGTGGTGGTGCATGGTC
GTTCTTAG-TTGGTGGAGCGATTTGTCTGGTCAATTCCGATAACGGACGAGACCTCGACC
TGCTAACTAGTAGTATTTATTA-GTCGATATAGGCGATAGCTTTTCTGGGGTTTGG-AAT
GATT----TC--GGTCATC----TCC-TATTTCAAGGAG----TGTGTAGTCTGGCTTGA
TAG--GTACGAATTA---AAAAA------CTTCTTAGAGGGACTACCT-GCCTCAAGCAG
GCGG-AAGTCCGAGGCAATAACAGGTCTGTGATGCCCTTAGATACCTT-GGGCCGCACGC
GCGCTACAATGTAGAAAACAAAAAGGC-----TCC--------TGGTCCGG-AAG---GA
TT-GGGTAATCATTTGAATTTTCTACGTAACTGGGATTG-ATCTTTGTAATTATTGATCA
TCAACGAGGAATTCCTTGTAAGCGCAGGTCATTACCCTGTGCTGAATATGTCCCTGCCCT
TTGTACACACCGCCCGTCGCTCCTACCGATCGAATGATACGGTAAAGTTAACGGATCG--
--------TTTT--ATCTGTGG-C-AAC-ACTGATA------TAAA-CTA-AAAG--TTA
-TT-TAAATCT--C----ATT--GTTT
>AM168055.1_Dictyostelium_mucoroides_var._stoloniferum_FOII_1
TCTAAG-TATAAATTCTTG-TACGATGAAA----CTGCAGACGGCTCATT--ACAA--CA
---GT----GATAAACTA-ATAGACTTTCGG-GTTTTT----AACCTTTT-GGATAACCG
CAGTAAATCGGGGCTAATACATAGAAG-CG-ATGGG-TGAC-GGC-----AAC-G---AA
GCTCAGCGATTATT----A-GCA-TTA-CTA-CCAATAC--CTTC--GGGTCTTGT-GGT
GAAACCGAATAATATTGCAGATCGAGG--------ATT-TATCTTCGACAAGTCTACTGT

GTCACTGCCCTATCAACTTTCGATGGTACGGTATTGGCCTACCATGGTTGTAACGGGTAA
CGGGGAATTAGGGTTCGATTCCGGAGAGGGAGCCTGAGAAATGGCTACCACTTCTACGGA
AGGCAGCAGGCGCGCAAATT-ACTCAATCCCAATAC-GGGGAAGTAGTGACAATAAATAT
CAATACCTATCC--TT-AATGGAGG--GCAATTGAAATGAACACAAATTAAAACTCTTAA
TT-AACACAATTGGAGGGCAAGTCTGGTGCCAGCAGCCGCGGTAATTCCAGCTCCAA-TA
GCATATACTAAAGTTGTTGCAGTTAAAA-AGC-TCGTAGTTGAAGTTAAAGGTTTACT-G
GG-CTAA----------AGTTATTTG-CCGCTCT----------GGTGGTTAAAT----C
AACTCCAGTATCTCTTTCG-CTC----AATAG-TTCAGCTTGTATCAACCT-------AG
TTGGTACTCGTTTG---------------GACATTTCACTGTGAGAAAA-TTGTGGTGTT
TAAAGCA--GG-C---GTC---TC-GCCTGATCTTTTGCAGCATGGTATGATGGAACATG
ACAT---TTTGCGC--TATTGGTTT--GCG----TCTAAAGTGTAATGATTAATAGGGAT
GGATGGGGGTGTTCATATTGGTGGGCGAGAGGTGAAATTCGTTGACCCTATCAAGATGAA
CTTCTGCGAAAGCATTCACCAAATACTTCCCCATTAATCAAGAACGAAAGTTTGGGGATC
GAAGACGATCAGATACCGTCGTAGTCCAAACTATAAACTATGTCGACCAGGGATCGGTTA
AA--AT-TTTTTTAAAATTTAATCGGCACCTT-GTGAGAAATCAC-GAGTGTTTAGATTC
CGGGGGGAGTATGGTCGCAAGTCTGAAACTTAAAGGAATTGACGGAAGGGCACACAATGG
AGTGGAG-CCTGCGGCTTAATTT-GACTCAACTCGGGAAA-ACTTACCAAGCTAAGATAT
AGTAAGGATTGACAGA-CTAA-AAGATCTTTCATGATTCTATAAGTGGTGGTGCATGGTC
GTTCTTAG-TTGGTGGAGCGATTTGTCTGGTCAATTCCGATAACGGACGAGACCTCGACC
TGCTAACTAGTAGTATTTATTA-GTCGATATGGGCGATAGCTTTTCTGGGGTGGAG-AAC
GATT----TC--GGTCGCT----TCT-TACTTCAAGGAG----TGTGTAGTCTGACTTGA
TAG--GTACGAATTA---AAAAA---A--CTTCTTAGAGGGACTACCT-GCCTCAAGCAG
GCGG-AAGTCCGAGGCAATAACAGGTCTGTGATGCCCTTAGATACCTT-GGGCCGCACGC
GCGCTACAATGTAGAAAACAAAAAGGC-----TCC--------TGGTCCGG-AAG---GA
TT-GGGTAATCAATTGAATTTTCTACGTAACTGGGATTG-ATCTTTGTAATTATTGATCA
TCAACGAGGAATTCCTTGTAAGCGTAAGTCATTACCTTATGCTGAATATGTCCCTGCCCT
TTGTACACACCGCCCGTCGCTCCTACCGATCGAATGATACGGTAAAGTTAACGGATCG--
--------TTTT--ACCTTTGG-C-AAC-ATTGGTA------TAAA-CTA-AAAG--TTA
-TT-TAAATCT--C----ATT--GTTT
>AM168028.1_Dictyostelium_aureum_SL1
TCTAAG-TATAAATTCTTG-TACGATGAAA----CTGCAGACGGCTCATT--ACAA--CA
---GT----GATAAACTG-CTAGACTTTCGG-GTTTTT----AACCTTTT-GGATAACCG
CAGTAAATCGGGGCTAATACATAGAAG-CG-ATGGG-CGACTGGT-----AAC-G-G-AA
GCTCAGCGATTATT----A-GCA-TAA-CTA-CCAATAC--CTTC--GGGTCTTGT-GGT
GAAACCGAATAATATTGCAGATCGAAG--------ATT-TATCTTCGACAAGTCTAATGT
GTCACTGCCCTATCAACTTTCGATGGTACGGTATTGGCCTACCATGGTTGTAACGGGTAA
CGGGGAATTAGGGTTCGATTCCGGAGAGGGAGCCTGAGAAATGGCTACCACTTCTATGGA
AGGCAGCAGGCGCGCAAATT-ACTCAATCCCAATAC-GGGGAAGTAGTGACAATAAATAT
CAATACCTATCC--TT-AATGGAGG--GCAATTGAAATGAACACAAATTAAAACTCTTAA
TT-AACACAATTGGAGGGCAAGTCTGGTGCCAGCAGCCGCGGTAATTCCAGCTCCAA-TA
GCATATACTAAAGTTGTTGCAGTTAAAA-AGC-TCGTAGTTGAAGTTAAAGGTTTATT-G
GG-TCAA----------AGCCATTTG-CCGCTCT----------GGTGGTTAAAT----G
TGCTCCAGTATTTCTTTTT-TTTGAAAAATTG-TTCAGCTTGTATCAACCT-------AG
TTGGTACTCGTTTG---------------GACATTTCACTGTGAGAAAA-TTGTGGTGTT
TAAAGCA--GG-C---GTC---TC-GCCTGATCTTTTGCAGCATGGTATGATGAAACATG
ACAT---TTTGCGC--TATTGGTTT--GCG----TCTAAAGTGTAATGATTAATAGGGAT
GGATGGGGGTGTTCATATTGGTGGGCGAGAGGTGAAATTCGTTGACCCTATCAAGATGAA
CTTCTGCGAAAGCATTCATCAAATACTTCCCCATTAATCAAGAACGAAAGTTTGGGGATC
GAAGACGATCAGATACCGTCGTAGTCCAAACTATAAACTATGTCGACCAGGGATCGGTTA
AA--AT-TTTTTTAAAATTTAATCGGCACCTT-GTGAGAAATCAC-GAGTGTTTAGATTC
CGGGGGGAGTATGGTCGCAAGTCTGAAACTTAAAGGAATTGACGGAAGGGCACACAATGG
AGTGGAG-CCTGCGGCTTAATTT-GACTCAACTCGGGAAA-ACTTACCAAGCTAAGATAT
AGTTAGGATTGACAGA-CTAA-AAGATCTTTCATGATTCTATAAGTGGTGGTGCATGGTC
GTTCTTAG-TTGGTGGAGCGATTTGTCTGGTCAATTCCGATAACGGACGAGACCTCGACC
TGCTAACTAGTAGTATTTATTA-GTCAACATGGGCGATAGCTTTTCTGGGATTTAG-AAC
GATT----TC--GGTCGTT----TCT-GGTTTCAAGGAG----TGTGTAGTCTGACTTGA
TAG--GTACGAATTA---AAAAA---A--CTTCTTAGAGGGACTACCT-GCCTCAAGCAG
GCGG-AAGTCCGAGGCAATAACAGGTCTGTGATGCCCTTAGATACCTT-GGGCCGCACGC
GCGCTACAATGTATAAAACAAAAAGGT-----TCC--------TGGTCCGG-AAG---GA
TT-GGGTAATCAATTGAATTTTCTACGTAACTGGGATTG-ATCTTTGTAATTATTGATCA
TAAACGAGGAATTCCTTGTAAGCGTAGGTCATTACCCTATGCTGAATATGTCCCTGCCCT
TTGTACACACCGCCCGTCGCTCCTACCGATCGAATGATACGGTAAAGTTAACGGATAG--
--------TTTT--ATCTTTGG-C-AAC-ATTGATA------TAAA-TTA-AAAG--TTA
-TT-TAAATCT--C----ATT--GTTT
>AM167876.1_Dictyostelium_aureocephalum_TNS_C_180
TCTAAG-TATAAATTCTTG-TACGATGAAA----CTGCAGACGGCTCATT--ACAA--CA
---GT----GATAAACTG-CTAGACTTTCGG-GTTTTT----AACCTTTT-GGATAACCG
CAGTAAATCGGGGCTAATACATAGAAG-CG-ATGGG-CGACTGGT-----AAC-G-G-AA
GCTCAGCGATTATT----A-GCA-TAA-CTA-CCAATAC--CTTC--GGGTCTTGT-GGT
GAAACCGAATAATATTGCAGATCGAAG--------ATT-TATCTTCGACAAGTCTAATGT
GTCACTGCCCTATCAACTTTCGATGGTACGGTATTGGCCTACCATGGTTGTAACGGGTAA
CGGGGAATTAGGGTTCGATTCCGGAGAGGGAGCCTGAGAAATGGCTACCACTTCTACGGA
AGGCAGCAGGCGCGCAAATT-ACTCAATCCCAATAC-GGGGAAGTAGTGACAATAAATAT

CAATACCTATCC--TT-AATGGAGG--GCAATTGAAATGAACACAAATTAAAACTCTTAA
TT-AACACAATTGGAGGGCAAGTCTGGTGCCAGCAGCCGCGGTAATTCCAGCTCCAA-TA
GCATATACTAAAGTTGTTGCAGTTAAAA-AGC-TCGTAGTTGAAGTTAAAGGTTTATT-G
GG-TCAA----------AGCTATTTG-CCGCTCT----------GGTGGTTAAAT----A
CGCTCCAGTATTTCTTTTT-TTTGAAAAATTG-TTCAGCTTGTGTCAACCT-------AG
TTGGCGCTCGTTTG---------------GACATTTCACTGTGAGAAAA-TTGTGGTGTT
TAAAGCA--GG-C---GTC---TC-GCCTGATCTTTTGCAGCATGGTATGATGAAACATG
ACAT---TTTGCGC--TATTGGTTT--GCG----TCTAAAGTGTAATGATTAATAGGGAT
GGATGGGGGTGTTCATATTGGTGGGCGAGAGGTGAAATTCGTTGACCCTATCAAGATGAA
CTTCTGCGAAAGCATTCACCAAATACTTCTCCATTAATCAAGAACGAAAGTTTGGGGATC
GAAGACGATCAGATACCGTCGTAGTCCAAACTATAAACTATGTCGACCAGGGATCGGTTA
AA--AT-TTTTTTAAAATTTAATCGGCACCTT-GTGAGAAATCAC-GAGTGTTTAGATTC
CGGGGGGAGTATGGTCGCAAGTCTGAAACTTAAAGGAATTGACGGAAGGGCACACAATGG
AGTGGAG-CCTGCGGCTTAATTT-GACTCAACTCGGGAAA-ACTTACCAAGCTAAGATAT
AGTTAGGATTGACAGA-CTAA-AAGATCTTTCATGATTCTATAAGTGGTGGTGCATGGTC
GTTCTTAG-TTGGTGGAGCGATTTGTCTGGTCAATTCCGATAACGGACGAGACCTCGACC
TGCTAACTAGTAGTATTTATTA-GTCAATATGGGCGATAGCTTTTCTGGGATGGAG-AGC
CCTT----TC--GGGGGTT----TCT-TATTTCAAGGAG----TGTGTAGTCTGACTTGA
TAG--GTACGAATTA---AAAAA---A--CTTCTTAGAGGGACTACCT-GCCTCAAGCAG
GCGG-AAGTCCGAGGCAATAACAGGTCTGTGATGCCCTTAGATACCTT-GGGCCGCACGC
GCGCTACAATGTATAAAACAAAAAGGT-----TCC--------TGGTCCGG-AAG---GA
TT-GGGTAATCAATTGAATTTTCTACGTAACTGGGATTG-ATCTTTGTAATTATTGATCA
TAAACGAGGAATTCCTTGTAAGCGTAGGTCATTACCCTATGCTGAATATGTCCCTGCCCT
TTGTACACACCGCCCGTCGCTCCTACCGATCGAATGATACGGTAAAGTTAACGGATAG--
--------TTTT--ATCTTTGG-C-AAC-ATTGATA------TAAA-TTA-AAAG--TTA
-TT-TAAATCT--C----ATT--GTTT
>HQ141480.1_Dictyostelium_leptosomum_NZN49A
TCTAAG-TATAAATTCTTG-TACGATGAAA----CTGCAGACGGCTCATT--ACAA--CA
---GT----GATAAACTG-CTAGACTTTCGG-GTTTTT----AACCTTTT-GGATAACCG
CAGTAAATCGGGGCTAATACATAGAAG-CG-ATGGG-TGACTGGT-----AAC-G-G-AA
GCTCAGCGATTATT----A-GCA-TAA-CTA-CCAATAC--CTTC--GGGTCTTGT-GGT
GAAACCGAATAATATTGCAGATCGAGG--------ATT-TATCTTCGACAAGTCTAATGT
GTCACTGCCCTATCAACTTTCGATGGTACGGTATTGGCCTACCATGGTTGTAACGGGTAA
CGGGGAATTAGGGTTCGATTCCGGAGAGGGAGCCTGAGAAATGGCTACCACTTCTACGGA
AGGCAGCAGGCGCGCAAATT-ACTCAATCCCAATAC-GGGGAAGTAGTGACAATAAATAT
CAATACCTATCC--TT-AATGGAGG--GCAATTGAAATGAACACAAATTAAAACTCTTAA
TT-AACACAATTGGAGGGCAAGTCTGGTGCCAGCAGCCGCGGTAATTCCAGCTCCAA-TA
GCATATACTAAAGTTGTTGCAGTTAAAA-AGC-TCGTAGTTGAAGTTAAAGGTTTATT-G
GG-TCAA----------AGCTATTTG-CCGCTCT----------GGTGGTTAAAT----A
TGCTCCAGTATTTCTTTTT-TTTGAAAAATTG-TTCAGCTTGTATCAACTT-------TG
TTGGTACTCGTTTG---------------GACATTTCACTGTGAGAAAA-TTGTGGTGTT
TAAAGCA--GG-C---GTC---TC-GCCTGATCTTTTGCAGCATGGTATGATGAAACATG
ACAT---TTTGCGC--TATTGGTTT--GCG----TCTAAAGTGTAATGATTAATAGGGAT
GGATGGGGGTGTTCATATTGGTGGGCGAGAGGTGAAATTCGTTGACCCTATCAAGATGAA
CTTCTGCGAAAGCATTCACCAAATACTTCTCCATTAATCAAGAACGAAAGTTTGGGGATC
GAAGACGATCAGATACCGTCGTAGTCCAAACTATAAACTATGTCGACCAGGGATCGGTTA
AA--AT-TTTTTTAAAATTTAATCGGCACCTT-GTGAGAAATCAC-GAGTGTTTAGATTC
CGGGGGGAGTATGGTCGCAAGTCTGAAACTTAAAGGAATTGACGGAAGGGCACACAATGG
AGTGGAG-CCTGCGGCTTAATTT-GACTCAACTCGGGAAA-ACTTACCAAGCTAAGATAT
AGTTAGGATTGACAGA-CTAA-AAGATCTTTCATGATTCTATAAGTGGTGGTGCATGGTC
GTTCTTAG-TTGGTGGAGCGATTTGTCTGGTCAATTCCGATAACGGACGAGACCTCGACC
TGCTAACTAGTAGTATTTATTA-GTCAATATGGGCGATAGCTTTTCTGGGATTTAG-AGC
GATT----TC--GGTCGTT----TCT-GGTTTCAAGGAG----TGTGTAGTCTGGCTTGA
TAG--GTACGAATTA---AAAAA---A--CTTCTTAGAGGGACTACCT-GCCTCAAGCAG
GCGG-AAGTCCGAGGCAATAACAGGTCTGTGATGCCCTTAGATACCTT-GGGCCGCACGC
GCGCTACAATGTATAAAACAAAAAGGT-----TCC--------TGGTCCGG-AAG---GA
TT-GGGTAATCAATTGAATTTTCTACGTAACTGGGATTG-ATCTTTGTAATTATTGATCA
TAAACGAGGAATTCCTTGTAAGCGTAGGTCATTACCCTATGCTGAATATGTCCCTGCCCT
TTGTACACACCGCCCGTCGCTCCTACCGATCGAATGATACGGTAAAGTTAACGGATAG--
--------TTTT--ATCTTTGG-C-AAC-ATTGATA------TAAA-TTA-AAAG--TTA
-TT-TAAATCT--C----AT---GTT-
>AM168032.1_Dictyostelium_capitatum_91HO_50
TCTAAG-TATAAATTCTTG-TACGATGAAA----CTGCAGACGGCTCATT--ACAA--CA
---GT----GATAAACTG-CTAGACTTTCGG-GTTTTT----AACCTTTT-GGATAACCG
CAGTAAATCGGGGCTAATACATAGAAG-CG-ATGGG-CGACTGGT-----AAC-G-G-AA
GCTCAGCGATTATT----A-GCA-TAA-CTA-CCAATAC--CTTC--GGGTCTTGT-GGT
GAAACCGAATAATATTGCAGATCGAAG--------ATT-TATCTTCGACAAGTCTAATGT
GTCACTGCCCTATCAACTTTCGATGGTACGGTATTGGCCTACCATGGTTGTAACGGGTAA
CGGGGAATTAGGGTTCGATTCCGGAGAGGGAGCCTGAGAAATGGCTACCACTTCTACGGA
AGGCAGCAGGCGCGCAAATT-ACTCAATCCCAATAC-GGGGAAGTAGTGACAATAAATAT
CAATACCTATCC--TT-AATGGAGG--GCAATTGAAATGAACACAAATTAAAACTCTTAA
TT-AACACAATTGGAGGGCAAGTCTGGTGCCAGCAGCCGCGGTAATTCCAGCTCCAA-TA
GCATATACTAAAGTTGTTGCAGTTAAAA-AGC-TCGTAGTTGAAGTTAAAGGTTTATT-G

GG-TCAA----------AGCTATTTG-CCGCTCT----------GGTGGTTAAAT----A
TGCTCCAGTATTTCTTTTT-TTTGAAAAATTG-TTCAGCTTGTATCAACTT-------TG
TTGGTACTCGTTTG---------------GACATTTCACTGTGAGAAAA-TTGTGGTGTT
TAAAGCA--GG-C---GTC---TC-GCCTGATCTTTTGCAGCATGGTATGATGAAACATG
ACAT---TTTGCGC--TATTGGTTT--GCG----TCTAAAGTGTAATGATTAATAGGGAT
GGATGGGGGTGTTCATATTGGTGGGCGAGAGGTGAAATTCGTTGACCCTATCAAGATGAA
CTTCTGCGAAAGCATTCACCAAATACTTCTCCATTAATCAAGAACGAAAGTTTGGGGATC
GAAGACGATCAGATACCGTCGTAGTCCAAACTATAAACTATGTCGACCAGGGATCGGTTA
AA--AT-TTTTTTAAAATTTAATCGGCACCTT-GTGAGAAATCAC-GAGTGTTTAGATTC
CGGGGGGAGTATGGTCGCAAGTCTGAAACTTAAAGGAATTGACGGAAGGGCACACAATGG
AGTGGAG-CCTGCGGCTTAATTT-GACTCAACTCGGGAAA-ACTTACCAAGCTAAGATAT
AGTTAGGATTGACAGA-CTAA-AAGATCTTTCATGATTCTATAAGTGGTGGTGCATGGTC
GTTCTTAG-TTGGTGGAGCGATTTGTCTGGTCAATTCCGATAACGGACGAGACCTCGACC
TGCTAACTAGTAGTATTTATTA-GTCAATATGGGCGATAGCTTTTCTGGGATTTAG-AGC
GATT----TC--GGTCGTT----TCT-GGTTTCAAGGAG----TGTGTAGTCTGACTTGA
TAG--GTCCGAATTA---AAAAA---A--CTTCTTAGAGGGACTACCT-GCCTCAAGCAG
GCGG-AAGTCCGAGGCAATAACAGGTCTGTGATGCCCTTAGATACCTT-GGGCCGCACGC
GCGCTACAATGTATAAAACAAAAAGGT-----TCC--------TGGTCCGG-AAG---GA
TT-GGGTAATCAATTGAATTTTCTACGTAACTGGGATTG-ATCTTTGTAATTATTGATCA
TAAACGAGGAATTCCTTGTAAGCGCAGGTCATTACCCTGTGCTGAATATGTCCCTGCCCT
TTGTACACACCGCCCGTCGCTCCTACCGATCGAATGATACGCTAAAGTTAACGGATAG--
--------TTTT--ATCTTTGG-C-AAC-ATTGATA------TAAA-TTA-AAAG--TTA
-TT-TAAATCT--C----ATT--GTTT
>AM168059.1_Dictyostelium_pseudobrefeldianum_91HO_8
TCTAAG-TATAAATTCTTG-TACGATGAAA----CTGCAGACGGCTCATT--ACAA--CA
---GT----GATAAACTG-CTAGACTTTCGG-GTTTTT----AACCTTTT-GGATAACCG
CAGTAAATCGGGGCTAATACATAGAAG-CG-ATGGG-CGACTGGT-----AAC-G-G-AA
GCTCAGCGATTATT----A-GCA-TAA-CTA-CCAATAC--CTTC--GGGTCTTGT-GGT
GAAACCGAATAATATTGCAGATCGAAG--------ATT-TATCTTCGACAAGTCTAATGT
GTCACTGCCCTATCAACTTTCGATGGTACGGTATTGGCCTACCATGGTTGTAACGGGTAA
CGGGGAATTAGGGTTCGATTCCGGAGAGGGAGCCTGAGAAATGGCTACCACTTCTACGGA
AGGCAGCAGGCGCGCAAATT-ACTCAATCCCAATAC-GGGGAAGTAGTGACAATAAATAT
CAATACCTATCC--TT-AATGGAGG--GCAATTGAAATGAACACAAATTAAAACTCTTAA
TT-AACACAATTGGAGGGCAAGTCTGGTGCCAGCAGCCGCGGTAATTCCAGCTCCAA-TA
GCATATACTAAAGTTGTTGCAGTTAAAA-AGC-TCGTAGTTGAAGTTAAAGGTTTATT-G
GG-TCAA----------AGCTATTTG-CCGCTCT----------GGTGGTTAAAT----A
TGCTCCAGTATTTCTTTTT-TTTGAAAAATTG-TTCAGCTTGTATCAACTT-------TG
TTGGTACTCGTTTG---------------GACATTTCACTGTGAGAAAA-TTGTGGTGTT
TAAAGCA--GG-C---GTC---TC-GCCTGATCTTTTGCAGCATGGTATGATGAAACATG
ACAT---TTTGCGC--TATTGGTTT--GCG----TCTAAAGTGTAATGATTAATAGGGAT
GGATGGGGGTGTTCATATTGGTGGGCGAGAGGTGAAATTCGTTGACCCTATCAAGATGAA
CTTCTGCGAAAGCATTCACCAAATACTTCTCCATTAATCAAGAACGAAAGTTTGGGGATC
GAAGACGATCAGATACCGTCGTAGTCCAAACTATAAACTATGTCGACCAGGGATCGGTTA
AA--AT-TTTTTTAAAATTTAATCGGCACCTT-GTGAGAAATCAC-GAGTGTTTAGATTC
CGGGGGGAGTATGGTCGCAAGTCTGAAACTTAAAGGAATTGACGGAAGGGCACACAATGG
AGTGGAG-CCTGCGGCTTAATTT-GACTCAACTCGGGAAA-ACTTACCAAGCTAAGATAT
AGTTAGGATTGACAGA-CTAA-AAGATCTTTCATGATTCTATAAGTGGTGGTGCATGGTC
GTTCTTAG-TTGGTGGAGCGATTTGTCTGGTCAATTCCGATAACGGACGAGACCTCGACC
TGCTAACTAGTAGTATTTATTA-GTCAATATGGGCGATAGCTTTTCTGGGATTTAG-AGC
GACT----TC--GGTTGTT----TCT-GGTTTCAAGGAG----TGTGTAGTCTGACTTGA
TAG--GTACGAATTA---AAAAA---A--CTTCTTAGAGGGACTACCT-GCCTCAAGCAG
GCGG-AAGTCCGAGGCAATAACAGGTCTGTGATGCCCTTAGATACCTT-GGGCCGCACGC
GCGCTACAATGTATAAAACAAAAAGGT-----TCC--------TGGTCCGG-AAG---GA
TT-GGGTAATCAATTGAATTTTCTACGTAACTGGGATTG-ATCTTTGTAATTATTGATCA
TAAACGAGGAATTCCTTGTAAGCGTAGGTCATTACCCTATGCTGAATATGTCCCTGCCCT
TTGTACACACCGCCCGTCGCTCCTACCGATCGAATGATACGGTAAAGTTAACGGATAG--
--------TTTT--ATCTTTGG-C-AAC-ATTGATA------TAAA-TTA-AAAG--TTA
-TT-TAAATCT--C----ATT--GTTT
>GQ496158.1_Dictyostelium_austroandinum
TCTAAG-TATAAATTCTTG-TACGATGAAA----CTGCAGACGGCTCATT--ACAA--CA
---GT----GATAAACTG-CTAGACTTTCGG-GTTTTT----AACCTTTT-GGATAACCG
CAGTAAATCGGGGCTAATACATAGAAG-CG-ATGGG-CGACTGGT-----AAC-G-G-AA
GCTCAGCGATTATT----A-GCA-TAA-CTA-CCAATAC--CTTC--GGGTCTTGT-GGT
GAAACCGAATAATATTGCAGATCGAAG--------ATT-TATCTTCGACAAGTCTAATGT
GTCACTGCCCTATCAACTTTCGATGGTACGGTATTGGCCTACCATGGTTGTAACGGGTAA
CGGGGAATTAGGGTTCGATTCCGGAGAGGGAGCCTGAGAAATGGCTACCACTTCTACGGA
AGGCAGCAGGCGCGCAAATT-ACTCAATCCCAATAC-GGGGAAGTAGTGACAATAAATAT
CAATACCTATCC--TT-AATGGAGG--GCAATTGAAATGAACACAAATTAAAACTCTTAA
TT-AACACAATTGGAGGGCAAGTCTGGTGCCAGCAGCCGCGGTAATTCCAGCTCCAA-TA
GCATATACTAAAGTTGTTGCAGTTAAAA-AGC-TCGTAGTTGAAGTTAAAGGTTTATT-G
GG-TCAA----------AGCTATTTG-CCGCTCT----------GGTGGTTAAAT----A
TGCTCCAGTATTTCTTTTT-TTTGAAAAATTG-TTCAGCTTGTATCAACTT-------TG
TTGGTACTCGTTTG---------------GACATTTCACTGTGAGAAAA-TTGTGGTGTT

TAAAGCA--GG-C---GTC---TC-GCCTGATCTTTTGCAGCATGGTATGATGAAACATG
ACAT---TTTGCGC--TATTGGTTT--GCG----TCTAAAGTGTAATGATTAATAGGGAT
GGATGGGGGTGTTCATATTGGTGGGCGAGAGGTGAAATTCGTTGACCCTATCAAGATGAA
CTTCTGCGAAAGCATTCACCAAATACTTCTCCATTAATCAAGAACGAAAGTTTGGGGATC
GAAGACGATCAGATACCGTCGTAGTCCAAACTATAAACTATGTCGACCAGGGATCGGTTA
AA--AT-TTTTTTAAAATTTAATCGGCACCTT-GTGAGAAATCAC-GAGTGTTTAGATTC
CGGGGGGAGTATGGTCGCAAGTCTGAAACTTAAAGGAATTGACGGAAGGGCACACAATGG
AGTGGAG-CCTGCGGCTTAATTT-GACTCAACTCGGGAAA-ACTTACCAAGCTAAGATAT
AGTTAGGATTGACAGA-CTAA-AAGATCTTTCATGATTCTATAAGTGGTGGTGCATGGTC
GTTCTTAG-TTGGTGGTGCGATTTGTCTGGTCAATTCCGATAACGGACGAGACCTCGACC
TGCTAACTAGTAGTATTTATTA-GTCAATATGGGCGATAGCTTTTCTGGGATTTAG-AGC
GATT----TC--GGTCGTT----TCT-GGTTTCAAGGAG----TGTGTAGTCTGACTTGA
TAG--GTACGAATTA---AAAAA---A--CTTCTTAGAGGGACTACCT-GCCTCAAGCAG
GCGG-AAGTCCGAGGCAATAACAGGTCTGTGATGCCCTTAGATACCTT-GGGCCGCACGC
GCGCTACAATGTATAAAACAAAAAGGT-----TCC--------TGGTCCGG-AAG---GA
TT-GGGTAATCAATTGAATTTTCTACGTAACTGGGATTG-ATCTTTGTAATTATTGATCA
TAAACGAGGAATTCCTTGTAAGCGTAGGTCATTACCCTATGCTGAATATGTCCCTGCCCT
TTGTACACACCGCCCGTCGCTCCTACCGATCGAATGATACGGTAAAGTTAACGGATAG--
--------TTTT--ATCTTTGG-C-AAC-ATTGATA------TAAA-TTA-AAAG--TTA
-TT-TAAATCT--C----ATT--GTT-
>HM159992.1_Dictyostelium_leptosomopsis_Araucaria_1
TCTAAG-TATAAATTCTTG-TACGATGAAA----CTGCAGACGGCTCATT--ACAA--CA
---GT----GATAAACTG-CTAGACTTTCGG-GTTTTT----AACCTTTTCGGATAACCG
CAGTAAATCGGGGCTAATACATAGAAG-CG-ATGGG-CGACTGGT-----AAC-G-G-AA
GCTCAGCGATTATT----A-GCA-TAA-CTA-CCAATAC--CTTC--GGGTCTTGT-GGT
GAAACCGAATAATATTGCAGATCGAAG--------ATT-TATCTTCGACAAGTCTAATGT
GTCACTGCCCTATCAACTTTCGATGGTACGGTATTGGCCTACCATGGTTGTAACGGGTAA
CGGGGAATTAGGGTTCGATTCCGGAGAGGGAGCCTGAGAAATGGCTACCACTTCTACGGA
AGGCAGCAGGCGCGCAAATT-ACTCAATCCCAATAC-GGGGAAGTAGTGACAATAAATAT
CAATACCTATCC--TT-AATGGAGG--GCAATTGAAATGAACACAAATTAAAACTCTTAA
TT-AACACAATTGGAGGGCAAGTCTGGTGCCAGCAGCCGCGGTAATTCCAGCTCCAA-TA
GCATATACTAAAGTTGTTGCAGTTAAAA-AGC-TCGTAGTTGAAGTTAAAGGTTTATT-G
GG-TCAA----------AGCTATTTG-CCGCTCT----------GGTGGTTAAAT----A
TGCTCCAGTATTTCTTTTT-TTTGAAAAATTG-TTCAGCTTGTATCAACTT-------TG
TTGGTACTCGTTTG---------------GACATTTCACTGTGAGAAAA-TTGTGGTGTT
TAAAGCA--GG-C---GTC---TC-GCCTGATCTTTTGCAGCATGGTATGATGAAACATG
ACAT---TTTGCGC--TATTGGTTT--GCG----TCTAAAGTGTAATGATTAATAGGGAT
GGATGGGGGTGTTCATATTGGTGGGCGAGAGGTGAAATTCGTTGACCCTATCAAGATGAA
CTTCTGCGAAAGCATTCACCAAATACTTCTCCATTAATCAAGAACGAAAGTTTGGGGATC
GAAGACGATCAGATACCGTCGTAGTCCAAACTATAAACTATGTCGACCAGGGATCGGTTA
AA--AT-TTTTTTAAAATTTAATCGGCACCTT-GTGAGAAATCAC-GAGTGTTTAGATTC
CGGGGGGAGTATGGTCGCAAGTCTGAAACTTAAAGGAATTGACGGAAGGGCACACAATGG
AGTGGAG-CCTGCGGCTTAATTT-GACTCAACTCGGGAAA-ACTTACCAAGCTAAGATAT
AGTTAGGATTGACAGA-CTAA-AAGATCTTTCATGATTCTATAAGTGGTGGTGCATGGTC
GTTCTTAG-TTGGTGGAGCGATTTGTCTGGTCAATTCCGATAACGGACGAGACCTCGACC
TGCTAACTAGTAGTATTTATTA-GTCAATATGGGCGATAGCTTTTCTGGGATTTAG-AGC
GATT----TC--GGTCGTT----TCT-GGTTTCAAGGAG----TGTGTAGTCTGACTTGA
TAG--GTACGAATTA---AAAAA---A--CTTCTTAGAGGGACTACCT-GCCTCAAGCAG
GCGG-AAGTCCGAGGCAATAACAGGTCTGTGATGCCCTTAGATACCTT-GGGCCGCACGC
GCGCTACAATGTATAAAACAAAAAGGT-----TCC--------TGGTCCGG-AAG---GA
TT-GGGTAATCAATTGAATTTTCTACGTAACTGGGATTG-ATCTTTGTAATTATTGATCA
TAAACGAGGAATTCCTTGTAAGCGTAGGTCATTACCCTATGCTGAATATGTCCCTGCCCT
TTGTACACACCGCCCGTCGCTCCTACCGATCGAATGATACGGTAAAGTTAACGGATAG--
--------TTTT--ATCTTTGG-C-AAC-ATTGATA------TAAA-TTA-AAAG--TTA
-TT-TAAATCT--C----ATT--GTT-
>AM168054.1_Dictyostelium_mucoroides_S28b
TCTAAG-TATAAATTCTTG-TACGATGAAA----CTGCAGACGGCTCATT--ACAA--CA
---GT----GATAAACTG-CTAGACTTTCGG-GTTTTT----AACCTTTT-GGATAACCG
CAGTAAATCGGGGCTAATACATAGAAG-CG-ATGGG-CGACTGGT-----AAC-G-G-AA
GCTCAGCGATTATT----A-GCA-TAA-CTA-CCAATAC--CTTC--GGGTCTTGT-GGT
GAAACCGAATAATATTGCAGATCGAAG--------ATT-TATCTTCGACAAGTCTAATGT
GTCACTGCCCTATCAACTTTCGATGGTACGGTATTGGCCTACCATGGTTGTAACGGGTAA
CGGGGAATTAGGGTTCGATTCCGGAGAGGGAGCCTGAGAAATGGCTACCACTTCTACGGA
AGGCAGCAGGCGCGCAAATT-ACTCAATCCCAATAC-GGGGAAGTAGTGACAATAAATAT
CAATACCTATCC--TT-AATGGAGG--GCAATTGAAATGAACACAAATTAAAACTCTTAA
TT-AACACAATTGGAGGGCAAGTCTGGTGCCAGCAGCCGCGGTAATTCCAGCTCCAA-TA
GCATATACTAAAGTTGTTGCAGTTAAAA-AGC-TCGTAGTTGAAGTTAAAGGTTTATT-G
GG-TCAA----------AGCTATTTG-CCGCTCT----------GGTGGTTAAAT----A
TGCTCCAGTATTTCTTTTT-TTTGAAAAATTG-TTCAGCTTGTATCAACTT-------TG
TTGGTACTCGTTTG---------------GACATTTCACTGTGAGAAAA-TTGTGGTGTT
TAAAGCA--GG-C---GTC---TC-GCCTGATCTTTTGCAGCATGGTATGATGAAACATG
ACAT---TTTGCGC--TATTGGTTT--GCG----TCTAAAGTGTATTGATTAATAGGGAT
GGATGGGGGTGTTCATATTGGTGGGCGAGAGGTGAAATTCGTTGACCCTATCAAGATGAA

CTTCTGCGAAAGCATTCACCAAATACTTCTCCATTAATCAAGAACGAAAGTTTGGGGATC
GAAGACGATCAGATACCGTCGTAGTCCAAACTATAAACTATGTCGACCAGGGATCGGTTA
AA--AT-TTTTTTAAAATTTAATCGGCACCTT-GTGAGAAATCAC-GAGTGTTTAGATTC
CGGGGGGAGTATGGTCGCAAGTCTGAAACTTAAAGGAATTGACGGAAGGGCACACAATGG
AGTGGAG-CCTGCGGCTTAATTT-GACTCAACTCGGGAAA-ACTTACCAAGCTAAGATAT
AGTTAGGATTGACAGA-CTAA-AAGATCTTTCATGATTCTATAAGTGGTGGTGCATGGTC
GTTCTTAG-TTGGTGGAGCGATTTGTCTGGTCAATTCCGATAACGGACGAGACCTCGACC
TGCTAACTAGTAGTATTTATTA-GTCAATATGGGCGATAGCTTTTCTGGGATTTAG-AGC
GATT----TC--GGTCGTT----TCT-GGTTTCAAGGAG----TGTGTAGTCTGACTTGA
TAG--GTACGAATTA---AAAAA---A--CTTCTTAGAGGGACTACCT-GCCTCAAGCAG
GCGG-AAGTCCGAGGCAATAACAGGTCTGTGATGCCCTTAGATACCTT-GGGCCGCACGC
GCGCTACAATGTATAAAACAAAAAGGT-----TCC--------TGGTCCGG-AAG---GA
TT-GGGTAATCAATTGAATTTTCTACGTAACTGGGATTG-ATCTTTGTAATTATTGATCA
TAAACGAGGAATTCCTTGTAAGCGTAGGTCATTACCCTATGCTGAATATGTCCCTGCCCT
TTGTACACACCGCCCGTCGCTCCTACCGATCGAATGATACGGTAAAGTTAACGGATAG--
--------TTTT--ATCTTTGG-C-AAC-ATTGATA------TAAA-TTA-AAAG--TTA
-TT-TAAATCT--C----ATT--GTTT
>AM168030.1_Dictyostelium_brefeldianum_TNS_C_115
TCTAAG-TATAAATTCTTG-TACGATGAAA----CTGCAGACGGCTCATT--ACAA--CA
---GT----GATAAACTG-CTAGACTTTCGG-GTTTTT----AACCTTTT-GGATAACCG
CAGTAAATCGGGGCTAATACATAGAAG-CG-ATGGG-CGACTGGT-----AAC-G-G-AA
GCTCAGCGATTATT----A-GCA-TAA-CTA-CCAATAC--CTTC--GGGTCTTGT-GGT
GAAACCGAATAATATTGCAGATCGAAG--------ATT-TATCTTCGACAAGTCTAATGT
GTCACTGCCCTATCAACTTTCGATGGTACGGTATTGGCCTACCATGGTTGTAACGGGTAA
CGGGGAATTAGGGTTCGATTCCGGAGAGGGAGCCTGAGAAATGGCTACCACTTCTACGGA
AGGCAGCAGGCGCGCAAATT-ACTCAATCCCAATAC-GGGGAAGTAGTGACAATAAATAT
CAATACCTATCC--TT-AATGGAGG--GCAATTGAAATGAACACAAATTAAAACTCTTAA
TT-AACACAATTGGAGGGCAAGTCTGGTGCCAGCAGCCGCGGTAATTCCAGCTCCAA-TA
GCATATACTAAAGTTGTTGCAGTTAAAA-AGC-TCGTAGTTGAAGTTAAAGGTTTATT-G
GG-TCAA----------AGCTATTTG-CCGCTCT----------GGTGGTTAAAT----A
TGCTCCAGTATTTCTTTTT-TTTGAAAAATTG-TTCAGCTTGTATCAACTT-------TG
TTGGTACTCGTTTG---------------GACATTTCACTGTGAGAAAA-TTGTGGTGTT
TAAAGCA--GG-C---GTC---TC-GCCTGATCTTTTGCAGCATGGTATGATGAAACATG
ACAT---TTTGCGC--TATTGGTTT--GCG----TCTAAAGTGTAATGATTAATAGGGAT
GGATGGGGGTGTTCATATTGGTGGGCGAGAGGTGAAATTCGTTGACCCTATCAAGATGAA
CTTCTGCGAAAGCATTCACCAAATACTTCTCCATTAATCAAGAACGAAAGTTTGGGGATC
GAAGACGATCAGATACCGTCGTAGTCCAAACTATAAACTATGTCGACCAGGGATCGGTTA
AA--AT-TTTTTTAAAATTTAATCGGCACCTT-GTGAGAAATCAC-GAGTGTTTAGATTC
CGGGGGGAGTATGGTCGCAAGTCTGAAACTTAAAGGAATTGACGGAAGGGCACACAATGG
AGTGGAG-CCTGCGGCTTAATTT-GACTCAACTCGGGAAA-ACTTACCAAGCTAAGATAT
AGTTAGGATTGACAGA-CTAA-AAGATCTTTCATGATTCTATAAGTGGTGGTGCATGGTC
GTTCTTAG-TTGGTGGAGCGATTTGTCTGGTCAATTCCGATAACGGACGAGACCTCGACC
TGCTAACTAGTAGTATTTATTA-GTCAATATGGGCGATAGCTTTTCTGGGATTTAG-AGC
GATT----TC--GGTCGTT----TCT-GGTTTCAAGGAG----TGTGTAGTCTGACTTGA
TAG--GTACGAATTA---AAAAA---A--CTTCTTAGAGGGACTACCT-GCCTCAAGCAG
GCGG-AAGTCCGAGGCAATAACAGGTCTGTGATGCCCTTAGATACCTT-GGGCCGCACGC
GCGCTACAATGTATAAAACAAAAAGGT-----TCC--------TGGTCCGG-AAG---GA
TT-GGGTAATCAATTGAATTTTCTACGTAACTGGGATTG-ATCTTTGTAATTATTGATCA
TAAACGAGGAATTCCTTGTAAGCGTAGGTCATTACCCTATGCTGAATATGTCCCTGCCCT
TTGTACACACCGCCCGTCGCTCCTACCGATCGAATGATACGGTAAAGTTAACGGATAG--
--------TTTT--ATCTTTGG-C-AAC-ATCGATA------TAAA-TTA-AAAG--TTA
-TT-TAAATCT--C----ATT--GTTT
>HQ141482.1_Dictyostelium_mucoroides_sweden_20
TCTAAG-TATAAATTCTTG-TACGATGAAA----CTGCAGACGGCTCATT--ACAA--CA
---GT----GATAAACTG-CTAGACTTTCGG-GTTTTT----AACCTTTT-GGATAACCG
CAGTAAATCGGGGCTAATACATAGAAG-CG-ATGGG-CGACTGGT-----AAC-G-G-AA
GCTCAGCGATTATT----A-GCA-TAA-CTA-CCAATAC--CTTC--GGGTCTTGT-GGT
GAAACCGAATAATATTGCAGATCGAAG--------ATT-TATCTTCGACAAGTCTAATGT
GTCACTGCCCTATCAACTTTCGATGGTACGGTATTGGCCTACCATGGTTGTAACGGGTAA
CGGGGAATTAGGGTTCGATTCCGGAGAGGGAGCCTGAGAAATGGCTACCACTTCTACGGA
AGGCAGCAGGCGCGCAAATT-ACTCAATCCCAATAC-GGGGAAGTAGTGACAATAAATAT
CAATACCTATCC--TT-AATGGAGG--GCAATTGAAATGAACACAAATTAAAACTCTTAA
TT-AACACAATTGGAGGGCAAGTCTGGTGCCAGCAGCCGCGGTAATTCCAGCTCCAA-TA
GCATATACTAAAGTTGTTGCAGTTAAAA-AGC-TCGTAGTTGAAGTTAAAGGTTTATT-G
GG-TCAA----------AGCTATTTG-CCGCTCT----------GGTGGTTAAAT----A
TGCTCCAGTATTTCTTTTT-TTTGAAAAATTG-TTCAGCTTGTATCAACTT-------TG
TTGGTACTCGTTTG---------------GACATTTCACTGTGAGAAAA-TTGTGGTGTT
TAAAGCA--GG-C---GTC---TC-GCCTGATCTTTTGCAGCATGGTATGATGAAACATG
ACAT---TTTGCGC--TATTGGTTT--GCG----TCTAAAGTGTAATGATTAATAGGGAT
GGATGGGGGTGTTCATATTGGTGGGCGAGAGGTGAAATTCGTTGACCCTATCAAGATGAA
CTTCTGCGAAAGCATTCACCAAATACTTCTCCATTAATCAAGAACGAAAGTTTGGGGATC
GAAGACGATCAGATACCGTCGTAGTCCAAACTATAAACTATGTCGACCAGGGATCGGTTA
AA--AT-TTTTTTAAAATTTAATCGGCACCTT-GTGAGAAATCAC-GAGTGTTTAGATTC

CGGGGGGAGTATGGTCGCAAGTCTGAAACTTAAAGGAATTGACGGAAGGGCACACAATGG
AGTGGAG-CCTGCGGCTTAATTT-GACTCAACTCGGGAAA-ACTTACCAAGCTAAGATAT
AGTTAGGATTGACAGA-CTAA-AAGATCTTTCATGATTCTATAAGTGGTGGTGCATGGTC
GTTCTTAG-TTGGTGGAGCGATTTGTCTGGTCAATTCCGATAACGGACGAGACCTCGACC
TGCTAACTAGTAGTATTTATTA-GTCAATATGGGCGATAGCTTTTCTGGGATTTAG-AGC
GATT----TC--GGTCGTT----TCT-GGTTTCAAGGAG----TGTGTAGTCTGACTTGA
TAG--GTACGAATTA---AAAAA---A--CTTCTTAGAGGGACTACCT-GCCTCAAGCAG
GCGG-AAGTCCGAGGCAATAACAGGTCTGTGATGCCCTTAGATACCTT-GGGCCGCACGC
GCGCTACAATGTATAAAACAAAAAGGT-----TCC--------TGGTCCGG-AAG---GA
TT-GGGTAATCAATTGAATTTTCTACGTAACTGGGATTG-ATCTTTGTAATTATTGATCA
TAAACGAGGAATTCCTTGTAAGCGTAGGTCATTACCCTATGCTGAATATGTCCCTGCCCT
TTGTACACACCGCCCGTCGCTCCTACCGATCGAATGATACGGTAAAGTTAACGGATAG--
--------TTTT--ATCTTTGG-C-AAC-ATTGATA------TAAA-TTA-AAAG--TTA
-TT-TAAATCT--C----ATT--GTTT
>AM168043.1_Dictyostelium_implicatum_93HO_1
TCTAAG-TATAAATTCTTG-TACGATGAAA----CTGCAGACGGCTCATT--ACAA--CA
---GT----GATAAACTA-ATAGACTTTCGG-GTTTTT----AACCTTTT-GGATAACCG
CAGTAAATCGGGGCTAATACATAGAAG-CG-ATGGG-TGACTGGC-----AAC-G-G-AA
TCTCAGCGATTATT----A-GCA-TTA-CTA-CCAATAC--CTTC--GGGTCTTGT-GGT
GAAACCGAATAATATTGCAGATCGAGG--------ATT-TATCTTCGACAAGTCTACTGT
GTCACTGCCCTATCAACTTTCGATGGTACGGTATTGGCCTACCATGGTTGTAACGGGTAA
CGGGGAATTAGGGTTCGATTCCGGAGAGGGAGCCTGAGAAATGGCTACCACTTCTACGGA
AGGCAGCAGGCGCGCAAATT-ACTCAATCCCAATAC-GGGGAAGTAGTGACAATAAATAT
TAATACCTATCC--TT-AATGGAGG--GCAATTGAAATGAACACAAATTAAAACTCTTAA
TT-AACACAATTGGAGGGCAAGTCTGGTGCCAGCAGCCGCGGTAATTCCAGCTCCAA-TA
GCATATACTAAAGTTGTTGCAGTTAAAA-AGC-TCGTAGTTGAAGTCAAAGGTTTATT-G
GG-TCAA----------AGTTATTTG-CCGCTCT----------GGTGGTTAAAT----A
TACTCCAGTATCTCTTTTT-TT-----AATAG-TTCAGCTTGTATTAACTT-------TG
TTAGTACTTGTTTG---------------GACATTTCACTGTGAGAAAA-TTGTGGTGTT
TAAAGCA--GG-C---GTT---TC-GCCTGATCTTTTGCAGCATGGTATGATGAAACATG
ACAT---TTTACGC--TATTGGTTT--GCG----TCTAAAGTGTAATGATTAATAGGGAT
GGATGGGGGTGTTCATATTGGTGGGCGAGAGGTGAAATTCGTTGACCCTATCAAGATGAA
CTTCTGCGAAAGCATTCACCAAATACTTCCCCATTAATCAAGAACGAAAGTTTGGGGATC
GAAGACGATCAGATACCGTCGTAGTCCAAACTATAAACTATGTCGACCAGGGATCGGTTA
AA--AT-TTTTTTAAAATTTAATCGGCACCTT-GTGAGAAATCAC-GAGTGTTTATATTC
CGGGGGGAGTATGGTCGCAAGTCTGAAACTTAAAGGAATTGACGGAAGGGCACACAATGG
AGTGGAG-CCTGCGGCTTAATTT-GACTCAACTCGGGAAA-ACTTACCAAGCTAAGATAT
AGTAAGGATTGACAGA-CTAA-AAGATCTTTCATGATTCTATAAGTGGTGGTGCATGGTC
GTTCTTAG-TTGGTGGAGCGATTTGTCTGGTCAATTCCGATAACGGACGAGACCTCGACC
TGCTAACTAGTAGTATTTATTA-GTCAATATGGGCGATAGCTTTTCTGGGATTTAG-AGC
GATT----TC--GGTCGTT----TCT-TGTTTCAAGGAG----TGTGTAGTCTGGCTTGA
TAG--GTACGAATTA---AAAAA---A--CTTCTTAGAGGGACTACCT-GCCTCAAGCAG
GCGG-AAGTCCGAGGCAATAACAGGTCTGTGATGCCCTTAGATACCTT-GGGCCGCACGC
GCGCTACAATGTAGAAAACAAAAAGGT-----TCC--------TGGTCCGG-AAG---GA
TT-GGGTAATCAATTGAATTTTCTACGTAACTGGGATTG-ATCTTTGTAATTATTGATCA
TCAACGAGGAATTCCTTGTAAGCGTAGGTCATTACCCTATGCTGAATATGTCCCTGCCCT
TTGTACACACCGCCCGTCGCTCCTACCGATCGAATGATACGGTAAAGTTAACGGATCG--
--------TTTT--ATCTTTGG-C-AAC-ATTGATA------TAAA-TTA-AAAG--TTA
-TT-TAAATCT--C----ATT--GTTT
>GQ496159.1_Dictyostelium_chordatum
TCTAAG-TATAAATTCTTG-TACGATGAAA----CTGCAGACGGCTCATT--ACAA--CA
---GT----GATAAACTA-ATAGACTTTCGG-GTTTTT----AACCTTTT-GGATAACCG
CAGTAAATCGGGGCTAATACATAGAAG-CG-ATGGG-TGACTGGC-----AAC-G-G-AA
TCTCAGCGATTATT----A-GCA-TTA-CTA-CCAATAC--CTTC--GGGTCTTGT-GGT
GAAACCGAATAATATTGCAGATCGAGG--------ATT-TATCTTCGACAAGTCTACTGT
GTCACTGCCCTATCAACTTTCGATGGTACGGTATTGGCCTACCATGGTTGTAACGGGTAA
CGGGGAATTAGGGTTCGATTCCGGAGAGGGAGCCTGAGAAATGGCTACCACTTCTACGGA
AGGCAGCAGGCGCGCAAATT-ACTCAATCCCAATAC-GGGGAAGTAGTGACAATAAATAT
TAATACCTATCC--TT-AATGGAGG--GCAATTGAAATGAACACAAATTAAAACTCTTAA
TT-AACACAATTGGAGGGCAAGTCTGGTGCCAGCAGCCGCGGTAATTCCAGCTCCAA-TA
GCATATACTAAAGTTGTTGCAGTTAAAA-AGC-TCGTAGTTGAAGTCAAAGGTTTGTT-G
GG-TCAA----------AGTTATTTG-CCGCTCT----------GGTGGTTAAAT----A
TACTCCAGCATCTCTTTTT-TT-----AATAG-TTCAGCTTGTATTGACTT-------TG
TTAGTACTTGTTTG---------------GACATTTCACTGTGAGAAAA-TTGTGGTGTT
TAAAGCA--GG-C---GTC---TC-GCCTGATCTTTTGCAGCATGGTATGATGAAACATG
ACAT---TTTACGC--TATTGGTTT--GCG----TCTAAAGTGTAATGATTAATAGGGAT
GGATGGGGGTGTTCATATTGGTGGGCGAGAGGTGAAATTCGTTGACCCTATCAAGATGAA
CTTCTGCGAAAGCATTCACCAAATACTTCCCCATTAATCAAGAACGAAAGTTTGGGGATC
GAAGACGATCAGATACCGTCGTAGTCCAAACTATAAACTATGTCGACCAGGGATCGGTTA
AA--AT-TTTTTTAAAATTTAATCGGCACCTT-GTGAGAAATCAC-GAGTGTTTAGATTC
CGGGGGGAGTATGGTCGCAAGTCTGAAACTTAAAGGAATTGACGGAAGGGCACACAATGG
AGTGGAG-CCTGCGGCTTAATTT-GACTCAACTCGGGAAA-ACTTACCAAGCTAAGATAT
AGTAAGGATTGACAGA-CTAA-AAGATCTTTCATGATTCTATAAGTGGTGGTGCATGGTC

GTTCTTAG-TTGGTGGAGCGATTTGTCTGGTCAATTCCGATAACGGACGAGACCTCGACC
TGCTAACTAGTAGTATTTATTA-GTCAATATGGGCGATAGCTTTTCTGGGATGTAG-AGC
GATT----TC--GGTCGTT----TCT-TATTTCAAGGAG----TGTGTAGTCTGGCTTGA
TAG--GTACGAATTA---AAAAA---A--CTTCTTAGAGGGACTACCT-GCCTCAAGCAG
GCGG-AAGTCCGAGGCAATAACAGGTCTGTGATGCCCTTAGATACCTT-GGGCCGCACGC
GCGCTACAATGTAGAAAACAAAAAGGT-----TCC--------TGGTCCGG-AAG---GA
TT-GGGTAATCAATTGAATTTTCTACGTAACTGGGATTG-ATCTTTGTAATTATTGATCA
TCAACGAGGAATTCCTTGTAAGCGTAGGTCATTACCCTATGCTGAATATGTCCCTGCCCT
TTGTACACACCGCCCGTCGCTCCTACCGATCGAATGATACGGTAAAGTTAACGGATCG--
--------TTTT--ATCTTTGG-C-AAC-ATTGATA------TAAA-TTA-AAAG--TTA
-TT-TAAATCT--C----AT---GTT-
>JX173878.1_Dictyostelium_barbibulus_Sweden_4R
TCTAAG-TATAAATTCTTG-TACGATGAAA----CTGCAGACGGCTCATT--ACAA--CA
---GT----GATAAACTA-ATAGACTTTCGG-GTTTTT----AACCTTTT-GGATAACCG
CAGTAAATCGGGGCTAATACATAGAAG-CG-ATGGG-TGACTGGC-----AAC-G-G-AA
TCTCAGCGATTATT----A-GCA-TTA-CTA-CCAATAC--CTTC--GGGTCTTGT-GGT
GAAACCGAATAATATTGCAGATCGAGG--------ATT-TATCTTCGACAAGTCTACTGT
GTCACTGCCCTATCAACTTTCGATGGTACGGTATTGGCCTACCATGGTTGTAACGGGTAA
CGGGGAATTAGGGTTCGATTCCGGAGAGGGAGCCTGAGAAATGGCTACCACTTCTACGGA
AGGCAGCAGGCGCGCAAATT-ACTCAATCCCAATAC-GGGGAAGTAGTGACAATAAATAT
TAATACCTATCC--TT-AATGGAGG--GCAATTGAAATGAACACAAATTAAAACTCTTAA
TT-AACACAATTGGAGGGCAAGTCTGGTGCCAGCAGCCGCGGTAATTCCAGCTCCAA-TA
GCATATACTAAAGTTGTTGCAGTTAAAA-AGC-TCGTAGTTGAAGTCAAAGGTTTGTT-G
GG-TCAA----------AGTTATTTG-CCGCTCT----------GGTGGTTAAAT----A
TACTCCAGCATCTCTTTTT-TT-----AATAG-TTCAGCTTGTATTAACTT-------TG
TTGGTACTTGTTTG---------------GACATTTCACTGTGAGAAAA-TTGTGGTGTT
TAAAGCA--GG-C---GTC---TC-GCCTGATCTTTTGCAGCATGGTATGATGAAACATG
ACAT---TTTACGC--TATTGGTTT--GCG----TCTAAAGTGTAATGATTAATAGGGAT
GGATGGGGGTGTTCATATTGGTGGGCGAGAGGTGAAATTCGTTGACCCTATCAAGATGAA
CTTCTGCGAAAGCATTCACCAAATACTTCCCCATTAATCAAGAACGAAAGTTTGGGGATC
GAAGACGATCAGATACCGTCGTAGTCCAAACTATAAACTATGTCGACCAGGGATCGGTTA
AA--AT-TTTTTTAAAATTTAATCGGCACCTT-GTGAGAAATCAC-GAGTGTTTAGATTC
CGGGGGGAGTATGGTCGCAAGTCTGAAACTTAAAGGAATTGACGGAAGGGCACACAATGG
AGTGGAG-CCTGCGGCTTAATTT-GACTCAACTCGGGAAA-ACTTACCAAGCTAAGATAT
AGTAAGGATTGACAGA-CTAA-AAGATCTTTCATGATTCTATAAGTGGTGGTGCATGGTC
GTTCTTAG-TTGGTGGAGCGATTTGTCTGGTCAATTCCGATAACGGACGAGACCTCGACC
TGCTAACTAGTAGTATTTATTA-GTCAATATGGGCGATAGCTTTTCTGGGATGTAG-AGC
GACT----TC--GGTTGTT----TCT-TATTTCAAGGAG----TGTGTAGTCTGGCTTGA
TAG--GTACGAATTA---AAAAA---A--CTTCTTAGAGGGACTACCT-GCCTCAAGCAG
GCGG-AAGTCCGAGGCAATAACAGGTCTGTGATGCCCTTAGATACCTT-GGGCCGCACGC
GCGCTACAATGTAGAAAACAAAAAGGT-----TCC--------TGGTCCGG-AAG---GA
TT-GGGTAATCAATTGAATTTTCTACGTAACTGGGATTG-ATCTTTGTAATTATTGATCA
TCAACGAGGAATTCCTTGTAAGCGTAGGTCATTACCCTATGCTGAATATGTCCCTGCCCT
TTGTACACACCGCCCGTCGCTCCTACCGATCGAATGATACGGTAAAGTTAACGGATCG--
--------TTTT--ATCTTTGG-C-AAC-ATTGATA------TAAA-TTA-AAAG--TTA
-TT-TAAATCT--C----ATT--GTT-
>AM168057.1_Synstelium_polycarpum_VE1b
TCTAAG-TATAAATCTTTG-TACGGTGAAA----CTGCAGACGGCTCATT--ATAA--CA
---GT----GATAAACTA-CAGAACTTTCGC-GTTAA-----TTCGTTAT-GGATAACCG
CAGTAAATCGGGGCTAATACATACAAA-CG-AAAGG-CGAGCGGG----CAACCGTG-AG
TCCTTGCGATTATT----AACCA-TT--CTAACCAAT-CT-CTTC--GGAGTTTGT-GGC
GAAACCGAATAATATTGCTGATCGA----------AAC-TAGTTTCGACAAGTTCTATGT
GTCACTGCCCTATCAACTTTCGATGGTACGGTATTGGCCTACCATGGTTGTAACGGGTAA
CGGGGAATTAGGGTTCGATTCCGGAGAGGGCGCCTGAGAAATGGCGACCACTTCTACGGA
AGGCAGCAGGCGCGCAAATT-ACTCAATCCCGACAC-GGGGAAGTAGTGACAATAAATAT
TAATGCCTATCAGTTT-TACTGAGG--GCAATTGAAATAAGTACAATCTAAATCGCTTAA
CC-AATTTAATTGGAGGGCAAGTCTGGTGCCAGCAGCCGCGGTAATTCCAGCTCCAA-TA
GCGTATACTAAATTTGTTGCAGTTAAAA-AGC-TCGTAGTTAAAGTTGAAATTCTTC--G
GA-TAAA----------ACT---T-----GAGTA----------GCATC-ATTAT-----
---TGGTGTTT-ACTCTTG-CCG----TTTGT-TTCTTTTTAT-TCAACTCCT-CGTCTA
ACGTTGGATGTTTG---------------AATTCTCCACTGTGAGAAAA-ATGTAGTGTT
TAAAGCA--GG-C---GAT---TA-GTCTGATCTTTTGCAGCATGGTATGGTAAAATAAG
ATAC---TAAACACT-TATTGGTT---GTG----T-TGTAGTATAATGATTAATAGGGAA
GGGCGGGGCCGTTCATATTGATGGGCGAGAGGTGAAATTCGTTGACCCTATCAAGATGCA
CTACAGCGGAAGCATTCGGCAAGTACTTCTCCATTAATCAAGAACGAAAGTTTGGGGATC
GAAGACGATCAGATACCGTCGTAGTCCAAACCATAAACTATGTCGACCAGGGATTGGATG
GGTAAT-TTTTAATAAACCTATTCAGAACCTT-GTGGGAAACCAT-GAGTGTTTGGACTC
TGGGGGGAGTATGGTCGCAAGTCTGAAACTTAAAGGAATTGACGGAAGGGCACACAATGG
AGTGGAA-CCTGCGGCTTAATTT-GACTCAACTCGGGAAA-ACTTACCAAGCTAAGATAT
AGCAAGGATTGACAGA-CTAA-AAGATTTTTCATGATTCTATAAGTGGTGGTGCATGGTC
GTTCTTAG-TTGGTGGAGTGATTTGTCTGGTCAATTCCGATAACGGACGAGACCTCGACC
TACTAAATAGTGGTGTGTATTTGGTCAATATGGGTGATAGAGTTATGGCAATTTTG-CAT
TGAT---TTC--GGTTGGT-----GTAACGTTGACGTGG--CTTGTGTAATCTGATCATT

TAG--ACACTAA--AAAATTAAA---ATACTTCTTAGAGGGACTACCT-ACCTCAAGTGG
GGGG-AAGTTCGAGGCAATAACAGGTCTGTGATGCCCTTAGATACCTT-GGGCTGCACGC
GCGTTACAATGTAAAGTGGAAAAAGGT-----TCC--------TGGACCGA-AAG---GT
TT-GGGTAATCATTTGAATACTCTACGTAATGGGGATTG-TTCTTTGTAATTATCGAACA
TCAACGAGGAATTCCTTGTAAGCGTAAATCATTACTTTACGCTGAATATGTCCCTGCCCT
TTGTACACACCGCCCGTCGCTCCTACCGATCGAACGATACGGTAAAATCGACGGATCG--
--------TTCTTAAT-TCCGA-A-AGG-AAA-CCT------AGAA-TGA-AAAG--TTG
-TT-TAAATCT--C----ATT--GTTT
>AM168058.1_Synstelium_polycarpum_OhioWILDS
TCTAAG-TATAAATCTTTG-TACGGTGAAA----CTGCAGACGGCTCATT--ACAA--CA
---GT----GATAAACTA-CAGAACTTTCGC-GATTT-----ATCGTTAT-GGGTAACCG
CAGTAAATCGGGGCTAATACATACAAA-CG-GAAGG-CGAGCGGG----CAGCTGCG-AG
TCCTTGCGATTATT----AACCA-TT--CTAACCAAT-CT-CTTC--GGAGTTTGT-GGC
GAAACCGAATAATATTGCTGATCGA----------ATT-TTAATTCGACAAGTTCTATGT
GTCACTGCCCTATCAACTTTCGATGGTACGGTATTGGCCTACCATGGTTGTAACGGGTAA
CGGGGAATTAGGGTTCGATTCCGGAGAGGGCGCCTGAGAAATGGCGACCACTTCTACGGA
AGGCAGCAGGCGCGCAAATT-ACTCAATCCCAACAC-GGGGAAGTAGTGACAATAAATAT
TAATGCCTATCAGTTT-TACTGAGG--GCAATTGAAATAAGTACAATTTAAATCGCTTAA
CC-AATTTAATTGGAGGGCAAGTCTGGTGCCAGCAGCCGCGGTAATTCCAGCTCCAA-TA
GCGTATACTAAATTTGTTGCAGTTAAAA-AGC-TCGTAGTTAAAATTGAAATTATAT--G
GA-TAAA----------ACT---T-----GATTA----------GCATC-ATTCG-----
---TGGTGTTTTATTCTTG-CCA----TTAAT-TTCTTATGAT-TCAACTCCT-CGCCTA
GCGTTGGATGTTTG---------------AATTCTCCACTGTGAGAAAA-ATGTAGTGTT
TAAAGCA--GG-C---GAT---TA-GTCTGATCTTTTGCAGCATGGTATGGTAAAATAAG
ATAC---TAAACACT-CGTTGGTT---GTG----T-TCTGGTATAATGATTAATAGGGAA
GGGCGGGGCCGTTCATATTGATGGGCGAGAGGTGAAATTCGTTGACCCTATCAAGATGCA
CTACAGCGAAAGCATTCGGCAAGTACTTCTCCATTAATCAAGAACGAAAGTTTGGGGATC
GAAGACGATCAGATACCGTCGTAGTCCAAACCATAAACTATGTCGACCAGGGATTGGGTG
GGTAAT-TTTTAATAAACCTATTCAGAACCTT-GTGGGAAACCAT-GAGTGTTTGGACTC
TGGGGGGAGTATGGTCGCAAGTCTGAAACTTAAAGGAATTGACGGAAGGGCACACAGTGG
AGTGGAA-CCTGCGGCTTAATTT-GACTCAACTCGGGAAA-ACTTACCAAGCTAAGATAT
AGCAAGGATTGACAGA-CTAA-AAGATTTTTCATGATTCTATAAGTGGTGGTGCATGGTC
GTTCTTAG-TTGGTGGAGTGATTTGTCTGGTCAATTCCGATAACGGACGAGACCTCGACC
TACTAAATAGTGGTGCGTATTTGGTCAATATGGGTGATAGTATTATGGCAGTGCTG-CAG
CGTT---TTC--GGATGTT-----GTAGTATTGACATAG--TATGTGTAATTCGATCATT
TAG--GCACTAA--AAAATTAAA---ATACTTCTTAGAGGGACTACCT-ACCTCAAGTGG
GGGG-AAGTTCGAGGCAATAACAGGTCTGTGATGCCCTTAGATACCTT-GGGCTGCACGC
GCGTTACAATGTAAGGTGGAAAAAGGT-----TCC--------TGGACCGA-AAG---GT
TT-GGGTAATCATTTGAATACCCTACGTAATGGGGATTG-TTCTTTGTAATTATCGAACA
TCAACGAGGAATTCCTTGTAAGCGTAAATCATTACTTTACGCTGAATATGTCCCTGCCCT
TTGTACACACCGCCCGTCGCTCCTACCGATCGAACGATACGGTAAAATTGACAGATTG--
--------TTTTTAAT-TCCGA-A-AGG-AAAGTCT------AAGA-CAA-AAAG--TTA
-TT-TAAATCT--C----ATT--GTTT
>MT125951_Dictyostelium_discoideum_5751_1_23_HMJAU_MR302
TCTAAG-TATAAATTCTTG-TACGATGAAA----CTGCAGACGGCTCATT--ACAA--CA
---GT----GATAAACTA-ATAGACTTTCGG-GTTTT------ACCTTTT-GGATAACCG
CAGTAAATCGGGGCTAATACATACAAG-CG-ATGGG-TGACTGGC-----AAC-G-G-AA
GCTCAGCGATTATT----A-GCA-TT--CTA-CCAATGC--CTTC--GGGTTTTG--GGT
GATACCGAATAATATTGCAGATCGAGG--------ATT-TATCTTCGACAAGTCTACTGT
GTCACTGCCCTATCAACTTTCGATGGTACGGTATTGGCCTACCATGGTTGTAACGGGTAA
CGGGGAATTAGGGTTCGATTCCGGAGAGGGAGCCTGAGAAATGGCTACCACTTCTACGGA
AGGCAGCAGGCGCGCAAATT-ACTCAATCCCAATAC-GGGGAAGTAGTGACAATAAATAT
CAATACCTATCC--TT-TTTGGAGG--GCAATTGAAATGAACACAAATTAAAACTCTTAA
TT-AACACAATTGGAGGGCAAGTCTGGTGCCAGCAGCCGCGGTAATTCCAGCTCCAA-TA
GCATATACTAAAGTTGTTGCAGTTAAAA-AGC-TCGTAGTTGAAGTTTAAGGTTTACC-G
GG-TTTA----------TGTCATTTA-CCACTTC----------G-TGGTTAAAT----C
GACACCGGTATCTCTTTCT-T------AATAG-TTCAGCTTGTATTATCTT-------TG
ATAGTGCTTGTTTG---------------GACATTTCACTGTGAGAAAA-TTGTGGTGTT
TAAAGCA--GG-C---GTC---TC-GCCTGATCTTTTGCAGCATGGTATGATGAAACATG
ACAT---TTTACGC--TATTGGTTT--GCG----TTTAAAGTGTAATGATTAATAGGGAT
GGATGGGGGTGTTCATATTGGTGGGCGAGAGGTGAAATTCGTTGACCCTATCAAGATGAA
CTTCTGCGAAAGCATTCACCAAATACTTCCCCATTAATCAAGAACGAAAGTTTGGGGATC
GAAGACGATCAGATACCGTCGTAGTCCAAACTATAAACTATGTCGACCAGGGATCGGTTA
AA--AT-TTTTTCAAAATTTAATCGGCACCTT-GTGAGAAATCAT-GAGTGTTTAGATTC
CGGGGGGAGTATGGTCGCAAGTCTGAAACTTAAAGGAATTGACGGAAGGGCACACAATGG
AGTGGAG-CCTGCGGCTTAATTT-GACTCAACTCGGGAAA-ACTTACCAAGCTAAGATAT
AGTAAGGATTGACAGA-CTAA-AAGATCTTTCATGATTCTATAAGTGGTGGTGCATGGTC
GTTCTTAG-TTGGTGGAGCGATTTGTCTGGTCAATTCCGATAACGGACGAGACCTCGACC
TGCTAACTAGTAGTATTTATTA-GTCGATATAGACGATAGCTTTTCTGGGGTTTGG-AAT
GATT----TC--GGTCATC----TCC-TGCTTCAAGGAG----TGTGTAGTCTGACTCGA
TAG--GTACGAATTA---AAA--------CTTCTTAGAGGGACTACCT-GCCTCAAGCAG
GCGG-AAGTCCGAGGCAATAACAGGTCTGTGATGCCCTTAGATACCTT-GGGCCGCACGC
GCGCTACAATGTAGGAAACAAAAAGGC-----TCC--------TGGTCCGG-AAG---GA

TT-GGGTAATCATTTGAATTTCCTACGTAACTGGGCTTG-ATCTTTGTAATTATTGATCA
TAAACGAGGAATTCCTTGTAAGCGTAAGTCATTACCTTATGCTGAATATGTCCCTGCCCT
TTGTACACACCGCCCGTCGCTCCTACCGATCGAATGATACGGTAAAGTTAACGGATCG--
--------TTTT--ATCTGTGG-C-AAC-ACTGATA------TAAA-TTA-AAAG--TTA
-TT-TAAATCT--C----ATT--GTTT
>MT125952_Dictyostelium_discoideum_5752_3_17_HMJAU_MR302
TCTAAG-TATAAATTCTTG-TACGATGAAA----CTGCAGACGGCTCATT--ACAA--CA
---GT----GATAAACTA-ATAGACTTTCGG-GTTTT------ACCTTTT-GGATAACCG
CAGTAAATCGGGGCTAATACATACAAG-CG-ATGGG-TGACTGGC-----AAC-G-G-AA
GCTCAGCGATTATT----A-GCA-TT--CTA-CCAATGC--CTTC--GGGTTTTG--GGT
GATACCGAATAATATTGCAGATCGAGG--------ATT-TATCTTCGACAAGTCTACTGT
GTCACTGCCCTATCAACTTTCGATGGTACGGTATTGGCCTACCATGGTTGTAACGGGTAA
CGGGGAATTAGGGTTCGATTCCGGAGAGGGAGCCTGAGAAATGGCTACCACTTCTACGGA
AGGCAGCAGGCGCGCAAATT-ACTCAATCCCAATAC-GGGGAAGTAGTGACAATAAATAT
CAATACCTATCC--TT-TTTGGAGG--GCAATTGAAATGAACACAAATTAAAACTCTTAA
TT-AACACAATTGGAGGGCAAGTCTGGTGCCAGCAGCCGCGGTAATTCCAGCTCCAA-TA
GCATATACTAAAGTTGTTGCAGTTAAAA-AGC-TCGTAGTTGAAGTTTAAGGTTTACC-G
GG-TTTA----------TGTCATTTA-CCACTTC----------G-TGGTTAAAT----C
GACACCGGTATCTCTTTCT-T------AATAG-TTCAGCTTGTATTATCTT-------TG
ATAGTGCTTGTTTG---------------GACATTTCACTGTGAGAAAA-TTGTGGTGTT
TAAAGCA--GG-C---GTC---TC-GCCTGATCTTTTGCAGCATGGTATGATGAAACATG
ACAT---TTTACGC--TATTGGTTT--GCG----TTTAAAGTGTAATGATTAATAGGGAT
GGATGGGGGTGTTCATATTGGTGGGCGAGAGGTGAAATTCGTTGACCCTATCAAGATGAA
CTTCTGCGAAAGCATTCACCAAATACTTCCCCATTAATCAAGAACGAAAGTTTGGGGATC
GAAGACGATCAGATACCGTCGTAGTCCAAACTATAAACTATGTCGACCAGGGATCGGTTA
AA--AT-TTTTTCAAAATTTAATCGGCACCTT-GTGAGAAATCAT-GAGTGTTTAGATTC
CGGGGGGAGTATGGTCGCAAGTCTGAAACTTAAAGGAATTGACGGAAGGGCACACAATGG
AGTGGAG-CCTGCGGCTTAATTT-GACTCAACTCGGGAAA-ACTTACCAAGCTAAGATAT
AGTAAGGATTGACAGA-CTAA-AAGATCTTTCATGATTCTATAAGTGGTGGTGCATGGTC
GTTCTTAG-TTGGTGGAGCGATTTGTCTGGTCAATTCCGATAACGGACGAGACCTCGACC
TGCTAACTAGTAGTATTTATTA-GTCGATATAGACGATAGCTTTTCTGGGGTTTGG-AAT
GATT----TC--GGTCATC----TCC-TGCTTCAAGGAG----TGTGTAGTCTGACTCGA
TAG--GTACGAATTA---AAA--------CTTCTTAGAGGGACTACCT-GCCTCAAGCAG
GCGG-AAGTCCGAGGCAATAACAGGTCTGTGATGCCCTTAGATACCTT-GGGCCGCACGC
GCGCTACAATGTAGGAAACAAAAAGGC-----TCC--------TGGTCCGG-AAG---GA
TT-GGGTAATCATTTGAATTTCCTACGTAACTGGGCTTG-ATCTTTGTAATTATTGATCA
TAAACGAGGAATTCCTTGTAAGCGTAAGTCATTACCTTATGCTGAATATGTCCCTGCCCT
TTGTACACACCGCCCGTCGCTCCTACCGATCGAATGATACGGTAAAGTTAACGGATCG--
--------TTTT--ATCTGTGG-C-AAC-ACTGATA------TAAA-TTA-AAAG--TTA
-TT-TAAATCT--C----ATT--GTTT
>MW660746_Dictyostelium_mucoroides_5035_23_HMJAU_MR303
TCTAAG-TATAAATTCTTG-TACGATGAAA----CTGCAGACGGCTCATT--ACAA--CA
---GT----GATAAACTG-CTAGACTTTCGG-GTTTTT----AACCTTTT-GGATAACCG
CAGTAAATCGGGGCTAATACATAGAAG-CG-ATGGG-CGACTGGT-----AAC-G-G-AA
GCTCAGCGATTATT----A-GCA-TAA-CTA-CCAATAC--CTTC--GGGTCTTGT-GGT
GAAACCGAATAATATTGCAGATCGAAG--------ATT-TATCTTCGACAAGTCTAATGT
GTCACTGCCCTATCAACTTTCGATGGTACGGTATTGGCCTACCATGGTTGTAACGGGTAA
CGGGGAATTAGGGTTCGATTCCGGAGAGGGAGCCTGAGAAATGGCTACCACTTCTACGGA
AGGCAGCAGGCGCGCAAATT-ACTCAATCCCAATAC-GGGGAAGTAGTGACAATAAATAT
CAATACCTATCC--TT-AATGGAGG--GCAATTGAAATGAACACAAATTAAAACTCTTAA
TT-AACACAATTGGAGGGCAAGTCTGGTGCCAGCAGCCGCGGTAATTCCAGCTCCAA-TA
GCATATACTAAAGTTGTTGCAGTTAAAA-AGC-TCGTAGTTGAAGTTAAAGGTTTATT-G
GG-TCAA----------AGCTATTTG-CCGCTCT----------GGTGGTTAAAT----A
TGCTCCAGTATTTCTTTTT-TTTGAAAAATTG-TTCAGCTTGTATCAACTT-------TG
TTGGTACTCGTTTG---------------GACATTTCACTGTGAGAAAA-TTGTGGTGTT
TAAAGCA--GG-C---GTC---TC-GCCTGATCTTTTGCAGCATGGTATGATGAAACATG
ACAT---TTTGCGC--TATTGGTTT--GCG----TCTAAAGTGTAATGATTAATAGGGAT
GGATGGGGGTGTTCATATTGGTGGGCGAGAGGTGAAATTCGTTGACCCTATCAAGATGAA
CTTCTGCGAAAGCATTCACCAAATACTTCTCCATTAATCAAGAACGAAAGTTTGGGGATC
GAAGACGATCAGATACCGTCGTAGTCCAAACTATAAACTATGTCGACCAGGGATCGGTTA
AA--AT-TTTTTTAAAATTTAATCGGCACCTT-GTGAGAAATCAC-GAGTGTTTAGATTC
CGGGGGGAGTATGGTCGCAAGTCTGAAACTTAAAGGAATTGACGGAAGGGCACACAATGG
AGTGGAG-CCTGCGGCTTAATTT-GACTCAACTCGGGAAA-ACTTACCAAGCTAAGATAT
AGTTAGGATTGACAGA-CTAA-AAGATCTTTCATGATTCTATAAGTGGTGGTGCATGGTC
GTTCTTAG-TTGGTGGAGCGATTTGTCTGGTCAATTCCGATAACGGACGAGACCTCGACC
TGCTAACTAGTAGTATTTATTA-GTCAATATGGGCGATAGCTTTTCTGGGATTTAG-AGC
GATT----TC--GGTCGTT----TCT-GGTTTCAAGGAG----TGTGTAGTCTGACTTGA
TAG--GTACGAATTA---AAAAAA-----CTTCTTAGAGGGACTACCT-GCCTCAAGCAG
GCGG-AAGTCCGAGGCAATAACAGGTCTGTGATGCCCTTAGATACCTT-GGGCCGCACGC
GCGCTACAATGTATAAAACAAAAAGGT-----TCC--------TGGTCCGG-AAG---GA
TT-GGGTAATCAATTGAATTTTCTACGTAACTGGGATTGGATCTTTGTAAT-AT------

>MW660747_Dictyostelium_mucoroides_5735_3_23_HMJAU_MR303
TCTAAG-TATAAATTCTTG-TACGATGAAA----CTGCAGACGGCTCATT--ACAA--CA
---GT----GATAAACTG-CTAGACTTTCGG-GTTTTT----AACCTTTT-GGATAACCG
CAGTAAATCGGGGCTAATACATAGAAG-CG-ATGGG-CGACTGGT-----AAC-G-G-AA
GCTCAGCGATTATT----A-GCA-TAA-CTA-CCAATAC--CTTC--GGGTCTTGT-GGT
GAAACCGAATAATATTGCAGATCGAAG--------ATT-TATCTTCGACAAGTCTAATGT
GTCACTGCCCTATCAACTTTCGATGGTACGGTATTGGCCTACCATGGTTGTAACGGGTAA
CGGGGAATTAGGGTTCGATTCCGGAGAGGGAGCCTGAGAAATGGCTACCACTTCTACGGA
AGGCAGCAGGCGCGCAAATT-ACTCAATCCCAATAC-GGGGAAGTAGTGACAATAAATAT
CAATACCTATCC--TT-AATGGAGG--GCAATTGAAATGAACACAAATTAAAACTCTTAA
TT-AACACAATTGGAGGGCAAGTCTGGTGCCAGCAGCCGCGGTAATTCCAGCTCCAA-TA
GCATATACTAAAGTTGTTGCAGTTAAAA-AGC-TCGTAGTTGAAGTTAAAGGTTTATT-G
GG-TCAA----------AGCTATTTG-CCGCTCT----------GGTGGTTAAAT----A
TGCTCCAGTATTTCTTTTT-TTTGAAAAATTG-TTCAGCTTGTATCAACTT-------TG
TTGGTACTCGTTTG---------------GACATTTCACTGTGAGAAAA-TTGTGGTGTT
TAAAGCA--GG-C---GTC---TC-GCCTGATCTTTTGCAGCATGGTATGATGAAACATG
ACAT---TTTGCGC--TATTGGTTT--GCG----TCTAAAGTGTAATGATTAATAGGGAT
GGATGGGGGTGTTCATATTGGTGGGCGAGAGGTGAAATTCGTTGACCCTATCAAGATGAA
CTTCTGCGAAAGCATTCACCAAATACTTCTCCATTAATCAAGAACGAAAGTTTGGGGATC
GAAGACGATCAGATACCGTCGTAGTCCAAACTATAAACTATGTCGACCAGGGATCGGTTA
AA--AT-TTTTTTAAAATTTAATCGGCACCTT-GTGAGAAATCAC-GAGTGTTTAGATTC
CGGGGGGAGTATGGTCGCAAGTCTGAAACTTAAAGGAATTGACGGAAGGGCACACAATGG
AGTGGAG-CCTGCGGCTTAATTT-GACTCAACTCGGGAAA-ACTTACCAAGCTAAGATAT
AGTTAGGATTGACAGA-CTAA-AAGATCTTTCATGATTCTATAAGTGGTGGTGCATGGTC
GTTCTTAG-TTGGTGGAGCGATTTGTCTGGTCAATTCCGATAACGGACGAGACCTCGACC
TGCTAACTAGTAGTATTTATTA-GTCAATATGGGCGATAGCTTTTCTGGGATTTAG-AGC
GATT----TC--GGTCGTT----TCT-GGTTTCAAGGAG----TGTGTAGTCTGACTTGA
TAG--GTACGAATTA---AAAAAA-----CTTCTTAGAGGGACTACCT-GCCTCAAGCAG
GCGG-AAGTCCGAGGCAATAACAGGTCTGTGATGCCCTTAGATACCTT-GGGCCGCACGC
GCGCTACAATGTATAAAACAAAAAGGT-----TCC--------TGGTCCGG-AAG---GA
TT-GGGTAATCAATTGAATTTTCTACGTAACTGGGATTG-ATCTTTGTAATTATTGATCA
TAAACGAGGAATTCCTTGTAAGCGTAGGTCATTACCCTATGCTGAATATGTCCCTGCCCT
TTGTACACACCGCCCGTCGCTCCTACCGATCGAATGATACGGTAAAGTTAACGGATAG--
--------TTTT--ATCTTTGG-C-AAC-ATTGATA------TAAA-TTA-AAAG--TTA
-TT-TAAATCT--C----ATT--GTTT
>MW931857_Dictyostelium_robusticaule_5729_bai_2021_HMJAU_MR305
TCTAAG-TATAAATTCTTG-TACGATGAAA----CTGCAGACGGCTCATT--ACAA--CA
---GT----GATAAACTA-ATAGACTTTCGG-GTTTTT----AACCTTTT-GGATAACCG
CAGTAAATCGGGGCTAATACATAGAAG-CG-ATGGG-TGACTGGC-----AAC-G-G-AA
GCTCAGCGATTATT----A-GCA-TTA-CTA-CCAATAC--CTTC--GGGTCTTGT-GGT
GAAACCGAATAATATTGCAGATCGAGG--------ATT-TATCTTCGACAAGTCTACTGT
GTCACTGCCCTATCAACTTTCGATGGTACGGTATTGGCCTACCATGGTTGTAACGGGTAA
CGGGGAATTAGGGTTCGATTCCGGAGAGGGAGCCTGAGAAATGGCTACCACTTCTACGGA
AGGCAGCAGGCGCGCAAATT-ACTCAATCCCAATAC-GGGGAAGTAGTGACAATAAATAT
CAATACCTATCC--TT-AACGGAGG--GCAATTGAAATGAACACAAATTAAAACTCTTAA
TT-AACACAATTGGAGGGCAAGTCTGGTGCCAGCAGCCGCGGTAATTCCAGCTCCAA-TA
GCATATACTAAAGTTGTTGCAGTTAAAA-AGC-TCGTAGTTGAAGTTAAAGGTTTACT-G
GG-CTAA----------AGTTATTTG-CCGCTCT----------GGTGGTTAAAT----C
AACTCCAGTATCTCTTTTT-TT-----AATAG-TTCAGCTTCTATTATCTT-------TG
ATAGTAGTTGTTTG---------------GACATTTCACTGTGAGAAAA-TTGTGGTGTT
TAAAGCA--GG-C---GTT---TC-GCCTGATCTTTTGCAGCATGGTATGATGAAACATG
ACAT---TTTACGC--TATTGGTTT--GCG----TCTAAAGTGTAATGATTAATAGGGAT
GGATGGGGGTGTTCATATTGGTGGGCGAGAGGTGAAATTCGTTGACCCTATCAAGATGAA
CTTCTGCGAAAGCATTCACCAAATACTTCCCCATTAATCAAGAACGAAAGTTTGGGGATC
GAAGACGATCAGATACCGTCGTAGTCCAAACTATAAACTATGTCGACCAGGGATCGGTTA
AA--AT-TTTTTTAAAATTTAATCGGCACCTT-GTGAGAAATCAC-GAGTGTTTAGATTC
CGGGGGGAGTATGGTCGCAAGTCTGAAACTTAAAGGAATTGACGGAAGGGCACACAATGG
AGTGGAG-CCTGCGGCTTAATTT-GACTCAACTCGGGAAA-ACTTACCAAGCTAAGATAT
AGTAAGGATTGACAGA-CTAA-AAGATCTTTCATGATTCTATAAGTGGTGGTGCATGGTC
GTTCTTAG-TTGGTGGAGCGATTTGTCTGGTCAATTCCGATAACGGACGAGACCTCGACC
TGCTAACTAGTAGTATTTATTA-GTCAATATGGGCGATAGCTTCTCTGGGGTTTGG-AAT
GGTT----TC--GGCCATC----TCC-TGCTTCAAGGAG----TGTGTAGTCTGACTTGA
TAG--GTACGAATTA---AAAAA------CTTCTTAGAGGGACTACCT-GCCTCAAGCAG
GCGG-AAGTCCGAGGCAATAACAGGTCTGTGATGCCCTTAGATACCTT-GGGCCGCACGC
GCGCTACAATGTAGAAAACAAAAAGGT-----TCC--------TGGTCCGG-AAG---GA
TT-GGGTAATCATTTGAATTTTCTACGTAACTGGGATTG-ATCTTTGTAATTATTGATCA
TCAACGAGGAATTCCTTGTAAGCGTAGGTCATTACCCTATGCTGAATATGTCCCTGCCCT
TTGTACACACCGCCCGTCGCTCCTACCGATCGAATGATACGGTAAAGTTAACGGATCG--
--------TTTT--ATCTTTGG-C-AAC-ATTGATA------TAAA-CTA-AAAG--TTA
-TT-TAAATCT--C----ATT--GTT-
>MW931856_Dictyostelium_robusticaule_5729_huang_2021_HMJAU_MR305

TCTAAG-TATAAATTCTTG-TACGATGAAA----CTGCAGACGGCTCATT--ACAA--CA
---GT----GATAAACTA-ATAGACTTTCGG-GTTTTT----AACCTTTT-GGATAACCG
CAGTAAATCGGGGCTAATACATAGAAG-CG-ATGGG-TGACTGGC-----AAC-G-G-AA
GCTCAGCGATTATT----A-GCA-TTA-CTA-CCAATAC--CTTC--GGGTCTTGT-GGT
GAAACCGAATAATATTGCAGATCGAGG--------ATT-TATCTTCGACAAGTCTACTGT
GTCACTGCCCTATCAACTTTCGATGGTACGGTATTGGCCTACCATGGTTGTAACGGGTAA
CGGGGAATTAGGGTTCGATTCCGGAGAGGGAGCCTGAGAAATGGCTACCACTTCTACGGA
AGGCAGCAGGCGCGCAAATT-ACTCAATCCCAATAC-GGGGAAGTAGTGACAATAAATAT
CAATACCTATCC--TT-AACGGAGG--GCAATTGAAATGAACACAAATTAAAACTCTTAA
TT-AACACAATTGGAGGGCAAGTCTGGTGCCAGCAGCCGCGGTAATTCCAGCTCCAA-TA
GCATATACTAAAGTTGTTGCAGTTAAAA-AGC-TCGTAGTTGAAGTTAAAGGTTTACT-G
GG-CTAA----------AGTTATTTG-CCGCTCT----------GGTGGTTAAAT----C
AACTCCAGTATCTCTTTTT-TT-----AATAG-TTCAGCTTCTATTATCTT-------TG
ATAGTAGTTGTTTG---------------GACATTTCACTGTGAGAAAA-TTGTGGTGTT
TAAAGCA--GG-C---GTT---TC-GCCTGATCTTTTGCAGCATGGTATGATGAAACATG
ACAT---TTTACGC--TATTGGTTT--GCG----TCTAAAGTGTAATGATTAATAGGGAT
GGATGGGGGTGTTCATATTGGTGGGCGAGAGGTGAAATTCGTTGACCCTATCAAGATGAA
CTTCTGCGAAAGCATTCACCAAATACTTCCCCATTAATCAAGAACGAAAGTTTGGGGATC
GAAGACGATCAGATACCGTCGTAGTCCAAACTATAAACTATGTCGACCAGGGATCGGTTA
AA--AT-TTTTTTAAAATTTAATCGGCACCTT-GTGAGAAATCAC-GAGTGTTTAGATTC
CGGGGGGAGTATGGTCGCAAGTCTGAAACTTAAAGGAATTGACGGAAGGGCACACAATGG
AGTGGAG-CCTGCGGCTTAATTT-GACTCAACTCGGGAAA-ACTTACCAAGCTAAGATAT
AGTAAGGATTGACAGA-CTAA-AAGATCTTTCATGATTCTATAAGTGGTGGTGCATGGTC
GTTCTTAG-TTGGTGGAGCGATTTGTCTGGTCAATTCCGATAACGGACGAGACCTCGACC
TGCTAACTAGTAGTATTTATTA-GTCAATATGGGCGATAGCTTCTCTGGGGTTTGG-AAT
GGTT----TC--GGCCATC----TCC-TGCTTCAAGGAG----TGTGTAGTCTGACTTGA
TAG--GTACGAATTA---AAAAA------CTTCTTAGAGGGACTACCT-GCCTCAAGCAG
GCGG-AAGTCCGAGGCAATAACAGGTCTGTGATGCCCTTAGATACCTT-GGGCCGCACGC
GCGCTACAATGTAGAAAACAAAAAGGT-----TCC--------TGGTCCGG-AAG---GA
TT-GGGTAATCATTTGAATTTTCTACGTAACTGGGATTG-ATCTTTGTAATTATTGATCA
TCAACGAGGAATTCCTTGTAAGCGTAGGTCATTACCCTATGCTGAATATGTCCCTGCCCT
TTGTACACACCGCCCGTCGCTCCTACCGATCGAATGATACGGTAAAGTTAACGGATCG--
--------TTTT--ATCTTTGG-C-AAC-ATTGATA------TAAA-CTA-AAAG--TTA
-TT-TAAATCT--C----ATT--GTT-
>MW660750_Cavenderia_fasciculata_5754_2wang_23_HMJAU_MR313
TCTAAG-TATAAGCTCTTG-TACGGCTA-GA---CTGCAGACGGCTCATT--ACAA--CG
GTTGT-----AT---CTTACAGGGCATCCGG-GTCGCA---AGACCTTCT-GGATAACCG
CAGTAAATCGGGGCTAATACATACAAA-CGGAGGGATGGAGAGGGC----AACCTTGAAG
TTTCTGCGATGGACATTTA-GCTATTC--GA-CCAACCC--CG-CAA-GGGAATG--GTT
GGAACCGGTTCATATTGCTAATCGACT-CTAGCTTGCTAGTAGTCTGATAAGCCCTATAG
ACAACCGCCCTATCAACTT--GATGGTAAGGTTTTGGCTTACCATGGTTGTAACGGGTAA
CGGGGAATCAGGGTTCGATTCCGGAGAGGACGCCTGAGAAACGGCGTCCACATCTACGGG
TGGCAGCAGGCGCGTAAATT-GCCCAATCTCAACAGAGAGGAGGCGGTGACAATAAATCC
CGATGGCTTTGGGGGCAACCCCAGG--CTAATCAGAATAAGTACACATTAAATCCCTTAA
CC-AATATAATTGGAGGGCAAGTCTGGTGCCAGCAGCCGCGGTAATTCCAGCTCCAA-TA
GCATATACTAATGTTGTTGCAGTTAAAA-CGC-TCGTAGCT-----TAATATCTTT--G
AG-CTAC------TATCGACTGTC---ATCCTCTTATG-TAG----CCGCAAGGT-GAAG
TAGGAG------GACCAGT-CGGCTCAAACAC-TGTAGGTCATG-----CGGC----TAG
CAATA-GTTG----------------CATAA---TCGACTGTGCATAAA-CCTTGATGCT
CAAGGTA--GG-CCT-TT---ATAGGGTAGATACACAGT-GCATGGCATTGTGGAACAAG
GCAT--CTCGCGGCTTAGTTGGT-GGGCC-------GCGGGGGCAATGATTAATAGGGAG
GAGCGGGGGCCTTCATATTGCAGGGCGAGAGGTGAAATTCGTTGACCCTTGCAAGATGTC
CTACAGCGAAAGCATTGGCCAAGTGCCTCCCCATTAGTCAAGAACGAAAGTTTGGGGATC
AAAGACGATCAGATACCGTCGTAGTCCAAACCATAAACTATGTCGACCAGCGATTAGGCG
GGCTACCTTCTTCGAGAGCTGCCTAGCAGCTT-GTGGGAAACCAT-GAGTGCTTGGACTC
TGGGGGGAGTATGGTCGCAAGGCTGAAACTTAAAGGAATTGACGGAAGGGCACACCATGG
AGTGGAG-CCTGCGGCTTAATTT-GACTCAACACGGGAAA-GCTTACCAAGCTCAGATAT
GATTAGGATTGACAGA-CTAA-AAGATCTTTCATGATCTCATAAGTGGTGGTGCATGGTC
GTTCTTAG-TTGGTGGAGTGATTTGTCAGGTCAATTCCGGTAACGGACGAGACCTCGACC
TGCTAACTAGTGGGATTCATTCTTTCG--ATTGACGAGGCAGGTATTGC-TTTGATTATA
G------GGGGCAACTTT-----TATAGTCGGG-TAGTGCTTGTAT-TAGTCTGGGAGAG
TGG--GTTTCAAA-AATTTAATT---AATCTTCCTAGAGGTACTTCTG-GCTCTAAGCCA
GAGG-AAGTCCGAGGCAATAACAGGTCTGTGATGCCCTTAGATATCTT-GGGCCGCACGC
GTGCTACAATGTAGGCGCTAATGAGTT---TATTTACATCC--ATCTCCGC-AAG---GA
GTATGGTAATC-TTGGAATCCCCTGCGTGATTGGGCTTG-ACCACTGTAACTGT-GGTCA
TCAACGAGGAATTCCTTGTATGCGCGAGTCACTATCTCGCGCAGAATCTGTCCCTGCCCT
TTGTACACACCGCCCGTCGCTCCTACCGATCGAACGATCAGGTAAAGTGGACAGACT---
--------------GAAACCCG-C-AAG-GGTT-----------TT-TGG--AAGT-CC-
A-T-TGAACCT--C----GCC--GTTT
>MW660751_Cavenderia_fasciculata_5259_1_23_HMJAU_MR313
TCTA-G-TATAAGCTCTTG-TACGGCTA-GA---CTGCAGACGGCTCATT--ACAA--CG
GTTGT-----AT---CTTACAGGGCATCCGG-GTCGCA---AGACCTTCT-GGATAACCG
CAGTAAATCGGGGCTAATACATACAAA-CGGAGGGATGGAGAGGGC----AACCTTGAAG

TTTCTGCGATGGACATTTA-GCTATTC--GA-CCAACCC--CG-CAA-GGGAATG--GTT
GGAACCGGTTCATATTGCTAATCGACT-CTAGCTTGCTAGTAGTCTGATAAGCCCTATAG
ACAACCGCCCTATCAACTT--GATGGTAAGGTTTTGGCTTACCATGGTTGTAACGGGTAA
CGGGGAATCAGGGTTCGATTCCGGAGAGGACGCCTGAGAAACGGCGTCCACATCTACGGG
TGGCAGCAGGCGCGTAAATT-GCCCAATCTCAACAGAGAGGAGGCGGTGACAATAAATCC
CGATGGCTTTGGGGGCAACCCCAGG--CTAATCAGAATAAGTACACATTAAATCCCTTAA
CC-AATATAATTGGAGGGCAAGTCTGGTGCCAGCAGCCGCGGTAATTCCAGCTCCAA-TA
GCATATACTAATGTTGTTGCAGTTAAAA-CGC-TCGTAGCT-----TAATATCTTTT--G
AG-CTAC------TATCGACTGTC---ATCCTCTTATG-TAG----CCGCAAGGT-GAAG
TAGGAG------GACCAGT-CGGCTCAAACAC-TGTAGGTCATG-----CGGC----TAG
CAATA-GTTG----------------CATAA---TCGACTGTGCATAAA-CCTTGATGCT
CAAGGTA--GG-CCT-TT---ATAGGGTAGATACACAGT-GCATGGCATTGTGGAACAAG
GCAT--CTCGCGGCTTAGTTGGT-GGGCC-------GCGGGGGCAATGATTAATAGGGAG
GAGCGGGGGCCTTCATATTGCAGGGCGAGAGGTGAAATTCGTTGACCCTTGCAAGATGTC
CTACAGCGAAAGCATTGGCCAAGTGCCTCCCCATTAGTCAAGAACGAAAGTTTGGGGATC
AAAGACGATCAGATACCGTCGTAGTCCAAACCATAAACTATGTCGACCAGCGATTAGGCG
GGCTACCTTCTTCGAGAGCTGCCTAGCAGCTT-GTGGGAAACCAT-GAGTGCTTGGACTC
TGGGGGGAGTATGGTCGCAAGGCTGAAACTTAAAGGAATTGACGGAAGGGCACACCATGG
AGTGGAG-CCTGCGGCTTAATTT-GACTCAACACGGGAAA-GCTTACCAAGCTCAGATAT
GATTAGGATTGACAGA-CTAA-AAGATCTTTCATGATCTCATAAGTGGTGGTGCATGGTC
GTTCTTAG-TTGGTGGAGTGATTTGTCAGGTCAATTCCGGTAACGGACGAGACCTCGACC
TGCTAACTAGTGGGATTCATTCTTTCG--ATTGACGAGGCAGGTATTGC-TTTGATTATA
G------GGGGCAACTTT-----TATAGTCGGG-TAGTGCTTGTAT-TAGTCTGGGAGAG
TGG--GTTTCAAA-AATTTAATT---AATCTTCCTAGAGGTACTTCTG-GCTCTAAGCCA

>MW660748_Heterostelium_pallidum_5759_1_23_HMJAU_MR314
TCTAAG-TATAA-CCTTTA-TACGGTGAAA----CTGCAGACGGCTCAATT-ACAA--CA
---GT----GATAAACTA-AAGAACTTCCGC-GCTTC-----GGCGTCTT-GGATAACCG
CAGTAAATCGGGGCTAATACATATAAA-CG-AGAGGGTGAGCGGG----CAACTGCG-AA
CCTTTGCGATTGTT----A-GCTATCTTTTT--CA--CCAACCTCTTCGGAGTTTGTGGT
GAATCCGAACAATATTGCTGATCGAAA--------ATT-TATTTTCGACGAGTTCTTTGT
GTCACTGCCCTATCAACTTTCGATGGTAAGGTATTGGCTTACCATGGTTGTAACGGGTGA
CGGGGAATCAGGGTTCGATTCCGGAGAGGGAGCCTGAGAAATGGCTACCACTTCTACGGA
AGGCAGCAGGCGCGCAAATT-ACTCAATCCCAATAC-GGGGAAGTAGTGACAAAAAATAC
TAATGCCTTTCCATAT-TATGGGGG--GCAATTGGAATAAGTACAACTTAAATCGCTTAG
CA-AAAGTGATTGGAGGGCAAGTCTGGTGCCAGCAGCCGCGGTAATTCCAGCTCCAA-TA
GCGTATACTAAATTTGTTGCAGTTAAAA-AGC-TCGTAGTTGAGATTGAGATTTCTTG-G
GT-TTAAGCCAGTCATAGTAGCTTT--CGGGTTATTATGA-T-TTCGGTTAAAGCTTTTG
AGTGGATTTTA-TTTTTCA-CTTTTAAAGTTA-CCAAGGGATTTCCAACTGC-CCA--TG
TAAGCTGGCAACAGTTT--AC-AATC--GGGTGATCTACTGTGAGAAAA-TTGTAGTGTT
CAAAGCA--GG-C---GTC---TTACGTTTGTTCAATGCAGCATGGTATAGTAAAATATG
ACAC---TAAATATAT-GTTGGTTG-TATA---TTCT-TAGTGTAATGACTAATAGGGAA
GGGCGGGGCCGTTCATATTGATGGGCGAGAGGTGAAATTCGTTGACCCTATCAAGATGCA
CTACAGCGAAAGCATTCGGCAAGTACTTCTCCATTAATCAAGAACGAAAGTTTGGGGATC
GAAGACGATCAGATACCGTCGTAGTCCAAACCATAAACTATGTCGACCAGGGATTGGACG
GATAAT-TTTTTAAAAACTCGCTCAGAACCTT-GTGAGAAATCAT-GAGTGTTTGGACTC
TGGGGGGAGTATGGTCGCAAGTCTGAAACTTAAAGGAATTGACGGAAGGGCACACAATGG
AGTGGAA-CCTGCGGCTTAATTT-GACTCAACTCGGGAAA-ACTTACCAAGCTCAGATAT
AATAAGGATTGACAGA-CTAA-AAGATCTTTCATGATTGTATAAGTGGTGGTGCATGGTC
GTTCTTAG-TTGGTGGAGTGATTTGTCTGGTCAATTCCGATAACGGACGAGACCTCTACC
TGCTAACTAGTGGTATTTATTTGGTCAATATGGAAGATAGTCATT-TGGTGTTGGT-GTC
AGG-----TCAAA---CTTG---GC-ATTCTTCATTGAG-TGGTGTGTATTCTGGTCAGA
TAG--GTACTAACTAAAAAAATA---AA-CTTCTTAGAGGGACTACCT-ACCTCAAGTGG
GGGGGAAGTCGGAGGCAATAACAGGTCTGTGATGCCCTTAGATACCTTTGGGCTGCACGC
GCGTTACA-TGTAGATGAGAAAAAGGT----TTCC--------GA--------------

>MW660749_Heterostelium_pallidum_5759_2_23_HMJAU_MR314
TCTAAG-TATAA-CCTTTA-TACGGTGAAA----CTGCAGACGGCTCATT--ACAA--CA
---GT----GATAAACTA-AAGAACTTCCGC-GCTTC-----GGCGTCTT-GGATAACCG
CAGTAAATCGGGGCTAATACATATAAA-CG-AGAGGGTGAGCGGG----CAACTGCG-AA
CCTTTGCGATTGTT----A-GCTATCTTTTT--CA--CCAACCTCTTCGGAGTTTGTGGT
GAATCCGAACAATATTGCTGATCGAAA--------ATT-TATTTTCGACGAGTTCTTTGT
GTCACTGCCCTATCAACTTTCGATGGTAAGGTATTGGCTTACCATGGTTGTAACGGGTGA

CGGGGAATCAGGGTTCGATTCCGGAGAGGGAGCCTGAGAAATGGCTACCACTTCTACGGA
AGGCAGCAGGCGCGCAAATT-ACTCAATCCCAATAC-GGGGAAGTAGTGACAAAAAATAC
TAATGCCTTTCCATAT-TATGGGGG--GCAATTGGAATAAGTACAACTTAAATCGCTTAG
CA-AAAGTGATTGGAGGGCAAGTCTGGTGCCAGCAGCCGCGGTAATTCCAGCTCCAA-TA
GCGTATACTAAATTTGTTGCAGTTAAAA-AGC-TCGTAGTTGAGATTGAGATTTCTTG-G
GT-TTAAGCCAGTCATAGTAGCTTT--CGGGTTATTATGA-T-TTCGGTTAAAGCTTTTG
AGTGGATTTTA-TTTTTCA-CTTTTAAAGTTA-CCAAGGGATTTCCAACTGC-CCA--TG
TAAGCTGGCAACAGTTT--AC-AATC--GGGTGATCTACTGTGAGAAAA-TTGTAGTGTT
CAAAGCA--GG-C---GTC---TTACGTTTGTTCAATGCAGCATGGTATAGTAAAATATG
ACAC---TAAATATAT-GTTGGTTG-TATA---TTCT-TAGTGTAATGACTAATAGGGAA
GGGCGGGGCCGTTCATATTGATGGGCGAGAGGTGAAATTCGTTGACCCTATCAAGATGCA
CTACAGCGAAAGCATTCGGCAAGTACTTCTCCATTAATCAAGAACGAAAGTTTGGGGATC
GAAGACGATCAGATACCGTCGTAGTCCAAACCATAAACTATGTCGACCAGGGATTGGACG
GATAAT-TTTTTAAAAACTCGCTCAGAACCTT-GTGAGAAATCAT-GAGTGTTTGGACTC
TGGGGGGAGTATGGTCGCAAGTCTGAAACTTAAAGGAATTGACGGAAGGGCACACAATGG
AGTGGAA-CCTGCGGCTTAATTT-GACTCAACTCGGGAAA-ACTTACCAAGCTCAGATAT
AATAAGGATTGACAGA-CTAA-AAGATCTTTCATGATTGTATAAGTGGTGGTGCATGGTC
GTTCTTAG-TTGGTGGAGTGATTTGTCTGGTCAATTCCGATAACGGACGAGACCTCTACC
TGCTAACTAGTGGTATTTATTTGGTCAATATGGAAGA-----------------------

>MW857293_Heterostelium_recretum_5756_1_17_HMJAU_MR315
TCTAAG-TATAA-CCTTTA-TACGGTGAAA----CTGCAGACGGCTCATT--ACAA--CA
---GT----GATAAACTA-AAGAACTTCCGC-GCTTC-----GGCGTTTT-GGATAACCG
CAGTAAATCGGGGCTAATACATGTAAA-CG-AGAGGATGAACGGG----CAACTGTG-AG
TCTTTGCGATTGTT----A-GCT-TTAAT----CA--CCAACCTCTTCGGAGTCAGTGGT
GAATCCCTACAATATTGCTGATCGGAA--------ACT-TGTTTCCGACGAGTTCTTTGT
GTCACTGCCCTATCAACTTTCGATGGTAAGGTATTGGCTTACCATGGTTGTAACGGGTGA
CGGGGAATCAGGGTTCGATTCCGGAGAGGGAGCCTGAGAAATGGCTACCACTTCTACGGA
AGGCAGCAGGCGCGCAAATT-ACTCAATCCCAATAC-GGGGAAGTAGTGACAAAAAATAC
TAATGCCTTTCCATTTATATGGGGG--GCAATTGGAATAAGTACAATTTAAATCGCTTAA
CG-AAAGTGATTGGAGGGCAAGTCTGGTGCCAGCAGCCGCGGTAATTCCAGCTCCAA-TA
GCGTATACTAAATTTGTTGCAGTTAAAA-AGC-TCGTAGTTGAGATTGAGATTTCTCA-G
GT-TTAG--CGGTCAACATTGCCTTCACGGGTTAATG-TT-TGAT-ACGTAAAGCTTTAT
AGTAGAATTTC-ATTTT-A-TTATTAAAGTTA-CTGAGGAATTTCCAACTGC-CCA--TG
GTAGCCAGCAATGGTTA--TC-AATA--GGGTGATCTACTGTGAGAAAA-TTGTAGTGTT
CAAAGCA--GG-C---GTC---TTTCGTTTGTTCAATGCAGCATGGTATAGTTAAATATG
ACAC---TAAATATAT-GTTGGTTG-TATA---T-CT-TAGTGTAATGACTAATAGGGAA
GGGCGGGGCCGTTCATATTGATGGGCGAGAGGTGAAATTCGTTGACCCTATCAAGATGCA
CTACAGCGAAAGCATTCGGCAAGTACTTCTCCATTAATCAAGAACGAAAGTTTGGGGATC
GAAGACGATCAGATACCGTCGTAGTCCAAACCATAAACTATGTCGACCAGGGATTGGACG
GATAAT-TTTTTAAAAACTCGCTCAGAACCTT-GTGAGAAATCAT-GAGTGTTTGGACTC
TGGGGGGAGTATGGTCGCAAGTCTGAAACTTAAAGGAATTGACGGAAGGGCACACAATGG
AGTGGAA-CCTGCGGCTTAATTT-GACTCAACTCGGGAAA-ACTTACCAAGCTCAGATAT
AATAAGGATTGACAGA-CTAA-AAGATCTTTCATGATTGTATAAGTGGTGGTGCATGGTC
GTTCTTAG-TTGGTGGAGTGATTTGTCTGGTCAATTCCGATAACGGACGAGACCTCTACT
TACTAACTAGTGATATTTATTTGGTCAATATGGGAGATAGTCATT-TGGTGTTGGTAGT-
AGGA--CGTCAAAA-TCTTA----CTATTCTTCATTGAG-TGGTGTGTATTCTGATCAGA
TAG--GTACTAATTTTAAAAATA---AA-CTTCTTAGAAGGACTACCT-ACCTCAAGTGG
GGGG-AAGTCGGAGGCAATAACAGGTCTGTGATGCCCTTAGATACCTT-GGGCTGCACGC
GCGTTACAATGTAGGCGAGAAAAAGGT----TTCC--------GGAATCGA-AAG---GT
TTC-GGTAATCAATTGAATTGCCTACGTAATGGGGATTA-ATTTTTGTAATTATCGATTA
TCAACGAGGAATTCCTTGTAAGCGTAAATCATTACTTTACGCTGAATATGTCCCTGCCCT
TTGTACACACCGCCCGTCGCTCCTACCGATCGAACGATTAGGTAAAACTGACGGACTA--
--GGC-GACTCT--TC---CGC-A-AGG-ATTTGTT-------GTT-TGG-GAAG--TTA
-GT-TAAATCT--C----ATT--GTT-
>X13160.1_Physarum_polycephalum_CL
TCTCCGAATAGAAGAGCAAGTCTCTCTGAAT----CTGCGAACGGCTCCGCATACCAGTT
GTAAACCATAGCAAGCAAGCCGCGTTGTTGCCGCAAGGCGACGGCGCGGTTCACAGGGAT
AACCCTGGTAATTCTGAGGCTAATACAAGAACGTACCACCCGCTTCGACCCGTAAGGGGA
GGGCGGGGGTTGTGTGACCCAG---GTCGCAAATATTAACTGGGAGTGGCCAC--ACGAT
CTGACCACCATACCAAACGGTTATCCGCTTCGAAAGCTTCGGTGAGTAACGGCGGATTTC
TGGGTGGCTCTCGCTGTGTGCTTCTGACCTATCAACT--AGATGGCAGCGTAACGGACAT
GCCATGGTAACAACGGGTA-CAGAGGATAAGGGTTCGATCCTGGAGAGTGGGCCTGAGAG
ATTGCTCACACTTCTAAGGAAGGCAGCAGGCGCGCAACGT-TCCCATTGGGCAAAGC--T
CGAGGGCGTTAGGGGACATATGAATGCCTGCCT-----TATGGTGG--GCAATTCAAATG

GGACTGTTTTAAACATCCTATCGAGTAACAATTAGAGGACAAGTCTGGTGCCAGCACCCG
CGGTAATTCCAGCTCTAA-TAGCATACGTTAAAGTTGTTGCGGTTAAAA-CGC-TCGTAG
TCGGCTCCAGACCTTCAGA--GCCTTGATTCGGGACACTGGGTCAGCTGCTCTTCTCCAA
GAGCGCGCCAACTACTGATGATAGGGGACGTCGGTCAATCGTGCCCTGGGCTGTCTCCCC
CCCGGGGGAGTCACAGGGTCGTTCAGCGGTCTTCGCGATCGCGGTTCGGCTCGGCTCGGG
G--TACCAATCACCATGATTAAA-CCGTAGTGACCAAAGCA-----C---GTCT--TTAG
ACGGGCACGGCACAGCATGG---GACGAAACGCACCGGG--CTCGCCTTTTTTTTGCGGG
GGCG------TGACTCGGTAAAAGCGAAAGGGATGTTCGAGGGTGACCGAATTGCTGGGC
GAGTGGTGAAATACGTTGACCCTAGCAAGTCGACCAAAGGCGTAAGCAGTCATCAAGGGC
ATTCCCGTTGATCAAGAGCGAAAGTTAAGGGTTCGAAGACGATCAGATACCGTCGTAGTC
TTAACTATAAATGATGCAGACCAGGGATAGGACAGT--GT-CCATCTCGACTCT--TCCG
GACCTT--GGAGAAATCAC-GAGTCTATGGGTTCTGGGGGGAGTATGGTCGCAAGGCTGA
AACTTAAAGGAATTGACGGAAGGGCACACAA-AGAGTGGAA-CCTGCGGCTTAATTT-GA
CTCAACACGGGAAA-ACTCACCAGGTCCGGATACACGTATGAAAGTCAAG-CTGA-AAGA
CTTTACTCAATGATGTAAGTGGTGGTGCATGGTCGTTCTTAG-TTCGTGGATTGATTTGT
CTGGTTTATTCCGATAACGAGCGAGACCCCGGCGTTC---CTAATAG------------
-----GGGTGGCAGCCAGACCGG----------------------------TC-------
-----GCAAG----------ACAGGTTAGCTCGCCAC----CTGAAGTT------ATG--
---CTTCTTAGACGTATCA----GAGCCGATAAGGTTCTTGAAATGGGTTAATAACAGGT
CAGTCATGCCCTTAGATGTTCT-GGGCCGCACGCGCGTTACAATGGCATGTAAAACGAGT
GCT----TGAACAAGGCGTCCCACGGCCGAAA-GGTCGTGGTAACC--CTTAGTCCCTGC
TTTGACTGGGACAG-ATCTTTGCAATTATTGGTCTCAAACGAGGAATTTTTAGTAATCGC
AGGTCATTAACCTGCGTTGAATGCGTCCCTGCCCTTTGTACACACCGCCCGTCGCTGCTA
CCGATTGGGTTTTACAGTTACGCGTTCGGAGGAC-------GATGTT---TTCGGGTCTT
AACGGGCTCCG-------GGGCGTCGG

Text S2. Alignment of *atp1* sequences on *Cavenderia* tree

>KF662075.1_Cavenderia_granulophora_CH11_4
GTATTAGGACAACCAATTGATGGAGGAGTTGAAAT--TAAAAATTCTAAAAATAC-----
--AAGATTAATTAGTTTAGAAAGAAAAGCACCAGGAATTGTTACAAGAAAATCTGTACAT
GAATCTATGTTAACAGGAGTAAAAATCGTTGACGCATTATTACCAATTGGAAGAGGACAA
CGTGAGTTAATCATTGGAGATCGTCAAACAGGAAAATCTTCAGTAGCTATTGATGCAATT
TTAAATCAAA---AAACTA--ATAAAGATATTGTT-----TGTGTTTATGTATCTGTAGG
ACAAAAAAAATCTACAATTAGAAGATTAGTAGAAATGTTAAATATGAGAGGATCTTTAAA
ATATAGTATTGTAGTAGTATCAACTGCATCAGATGCTTCTCCATTACAATTTTTAGCACC
ATATACAGGATGTGCAATTGGAGAATATTTCAGAGATAAAGGAGAACATGCTTTAATTAT
TTATGACGATTTAAGTAAACATGCAGTAGCATACCGTCAAATGTCTTTATTATTAAGACG
TCCTCCAGGACGA
>MZ318389_Cavenderia_fasciculata_5259_1_23_HMJAU_MR313
GTATTAGGACAACCAATCGATGGAGGAGTTGCATT--TAAAGATACAAAAACAAC-----
--AAGATTAATCAGTTTAGAAAGAAAAGCACCAGGAATTGTAACTAGAAAATCTGTACAT
GAGTCAATGTTAACAGGAGTAAAAATTGTTGATGCATTATTACCAATTGGAAGAGGACAA
CGTGAATTAATTATTGGAGATCGTCAAACAGGTAAATCTTCAATTGCAATTGATGCAATC
TTAAATCAAA---AAACAA--ATAAAGATATTGTA-----TGTGTATACGTATCCGTAGG
ACAAAAAAAATCAACAATTAGAAGATTAGTTGAAATGTTAAATGTTAAAGGTTCTTTAAA
ATATAGTATTGTAGTAGTATCAACAGCGTCTGATGCATCTCCTTTACAATTTTTAGCACC
ATATACAGGATGTGCAATCGGAGAATATTTTAGAGATAAAGGAGAACATGCTTTAATTAT
TTATGATGATTTAAGTAAACATGCCGTAGCATACCGTCAAATGTCTTTATTATTAAGACG
TCCTCCAGGAAGA
>MZ318390_Cavenderia_fasciculata_5754_2wang_23_HMJAU_MR313
GTATTAGGACAACCAATCGATGGAGGAGTTGCATT--TAAAGATACAAAAACAAC-----
--AAGATTAATCAGTTTAGAAAGAAAAGCACCAGGAATTGTAACTAGAAAATCTGTACAT
GAGTCAATGTTAACAGGAGTAAAAATTGTTGATGCATTATTACCAATTGGAAGAGGACAA
CGTGAATTAATTATTGGAGATCGTCAAACAGGTAAATCTTCAATTGCAATTGATGCAATC
TTAAATCAAA---AAACAA--ATAAAGATATTGTA-----TGTGTATACGTATCCGTAGG
ACAAAAAAAATCAACAATTAGAAGATTAGTTGAAATGTTAAATGTTAAAGGTTCTTTAAA
ATATAGTATTGTAGTAGTATCAACAGCGTCTGATGCATCTCCTTTACAATTTTTAGCACC
ATATACAGGATGTGCAATCGGAGAATATTTTAGAGATAAAGGAGAACATGCTTTAATTAT
TTATGATGATTTAAGTAAACATGCCGTAGCATACCGTCAAATGTCTTTATTATTAAGACG
TCCTCCAGGAAGA
>KF662113.1_Cavenderia_sp._TH18D_TH18D
GTATTAGGACAACCAATTGATGGAGGAGTAGAATT--TAAAGATACAAAAACAAC-----
--AAGATTAATTAGTTTAGAAAGAAAAGCACCAGGAATTGTAACAAGAAAATCAGTACAT
GAATCAATGTTAACAGGAGTAAAAATCGTAGATGCATTATTACCAATAGGAAGAGGACAA
CGTGAATTAATTATTGGAGATCGTCAAACAGGAAAATCATCAGTAGCAATTGATGCAATT
TTAAATCAAA---AAACAA--ATAAAGATATTGTA-----TGTGTATACGTATCAGTAGG
ACAAAAAAAATCAACAATTAGAAGATTAGTAGAAATGTTAAATTTAAAAGGATCTTTAAA
ATATAGTATTGTAGTAGTATCAACAGCATCAGATGCATCACCATTACAATTCTTAGCACC
ATATACAGGATGTGCTATTGGAGAATATTTCAGAGATAAAGGAGAACATGCTTTAATTAT
TTATGATGATTTAAGTAAACATGCAGTAGCATATCGTCAAATGTCTTTATTATTAAGACG
TCCTCCAGGACGA
>KF662112.1_Cavenderia_aureostipes_AU7B
GTATTAGGACAACCAATTGATGGAGGAGTAGAATT--CAAAGATACAAAAACAAC-----
--AAGATTAATTAGTTTAGAAAGAAAAGCACCAGGAATTGTAACAAGAAAATCCGTACAT
GAATCTATGTTAACAGGAGTAAAAATTGTAGATGCATTATTACCAATTGGAAGAGGACAA
CGTGAATTAATTATTGGAGATCGTCAAACAGGAAAATCTTCAGTAGCAATTGATGCAATT
TTAAACCAAA---AAACAA--ATAAAGATATTGTA-----TGTGTATACGTATCAGTAGG
ACAAAAAAAATCAACAATTAGAAGATTAGTAGAAATGTTAAACTTAAAAGGATCTTTAAA
ATATAGTATTGTAGTAGTATCAACAGCATCAGATGCATCACCATTACAATTCTTAGCACC
TTATACAGGATGTGCAATTGGAGAATATTTCAGAGATAAAGGAGAACATGCTTTAATTAT
TTATGATGATTTAAGTAAACATGCAGTAGCATATCGTCAAATGTCTTTATTATTAAGACG
TCCACCAGGACGA
>KF662123.1_Cavenderia_sp._TH1A_TH1A
GTATTAGGACAACCAATTGATGGAGGAGTAGAATT--TAAAGATACAAAAACAAC-----
--AAGATTAATTAGTTTAGAAAGAAAAGCACCAGGAATTGTAACAAGAAAATCAGTACAT
GAATCAATGTTAACAGGAGTAAAAATTGTAGATGCATTATTACCAATTGGAAGAGGACAA
CGTGAATTAATTATTGGAGATCGTCAAACAGGAAAATCTTCAGTAGCAATTGATGCAATT
TTAAACCAAA---AAACAA--ATAAAGATATTGTT-----TGTGTATATGTATCAGTAGG
ACAAAAAAAATCAACAATTAGAAGATTAGTAGAAATGTTAAATTTAAAAGGATCTTTAAA
ATATAGTATTGTAGTAGTATCAACAGCATCAGATGCATCACCATTACAATTCTTAGCACC
TTATACAGGATGTGCTATTGGAGAATATTTCAGAGATAAAGGAGAACATGCTTTAATTAT
TTATGATGATTTAAGTAAACATGCAGTAGCATACCGTCAAATGTCTTTATTATTAAGACG
TCCACCAGGACGA
>KF662116.1_Cavenderia_aureostipes_B15A
GTATTAGGACAACCAATTGATGGAGGAGTAGAATT--TAAAGATACAAAAACAAC-----
--AAGATTAATTAGTTTAGAAAGAAAAGCACCAGGAATTGTAACAAGAAAATCCGTACAT

GAATCTATGTTAACAGGAGTAAAAATTGTAGATGCATTATTACCAATTGGAAGAGGACAA
CGTGAATTAATTATTGGAGATCGTCAAACAGGAAAATCTTCAGTAGCAATTGATGCAATT
TTAAATCAAA---AAACAA--ATAAAGATATTGTA-----TGTGTATACGTATCAGTAGG
ACAAAAAAAATCAACAATTAGAAGATTAGTAGAAATGTTAAATTTAAAAGGATCTTTAAA
ATATAGTATTGTAGTAGTATCAACAGCATCAGATGCATCACCATTACAATTCTTAGCACC
ATATACAGGATGTGCTATTGGAGAATATTTCAGAGATAAAGGAGAACATGCTTTAATTAT
TTATGATGATTTAAGTAAACATGCAGTAGCATATCGTCAAATGTCTTTATTATTAAGACG
TCCTCCAGGACGA
>KF662117.1_Cavenderia_sp._TH39A_TH39A
GTATTAGGACAACCAATTGATGGAGGAGTAGAATT--CAAAGATACAAAAACAAC-----
--AAGATTAATTAGTTTAGAAAGAAAAGCACCAGGAATTGTAACAAGAAAATCCGTACAT
GAATCTATGTTAACAGGAGTAAAAATTGTAGATGCATTATTACCAATTGGAAGAGGACAA
CGTGAATTAATTATTGGAGATCGTCAAACAGGAAAATCTTCAGTAGCAATTGATGCAATT
TTAAATCAAA---AAACAA--ATAAAGATATTGTA-----TGTGTATACGTATCAGTAGG
ACAAAAAAAATCAACAATTAGAAGATTAGTAGAAATGTTAAATTTAAAAGGATCTTTAAA
ATATAGTATTGTAGTAGTATCAACAGCATCAGATGCATCACCATTACAATTCTTAGCACC
ATATACAGGATGTGCTATTGGAGAATATTTCAGAGATAAAGGAGAACATGCTTTAATTAT
TTATGATGATTTAAGTAAACATGCCGTAGCATATCGTCAAATGTCTTTATTATTAAGACG
TCCTCCAGGACGA
>KF662122.1_Cavenderia_myxobasis_NT2A
GTATTAGGACAACCAATTGATGGAGGAGTAGAATT--TAAAGATACAAAAACAAC-----
--AAGATTAATTAGTTTAGAAAGAAAAGCACCAGGAATTGTAACAAGAAAATCAGTACAT
GAATCAATGTTAACAGGAGTAAAAATTGTAGATGCATTATTACCAATAGGAAGAGGACAA
CGTGAATTAATTATTGGAGATCGTCAAACAGGAAAATCTTCAGTAGCAATTGATGCAATT
TTAAACCAAA---AAACAA--ATAAAGATATTGTT-----TGTGTATATGTATCAGTAGG
ACAAAAAAAATCAACAATTAGAAGATTAGTAGAAATGTTAAATTTAAAAGGATCTTTAAA
ATATAGTATTGTAGTAGTATCAACAGCATCAGATGCATCACCATTACAATTCTTAGCACC
TTATACAGGATGTGCTATTGGAGAATATTTCAGAGATAAAGGAGAACATGCTTTAATTAT
TTATGATGATTTAAGTAAACATGCAGTAGCATACCGTCAAATGTCTTTATTATTAAGACG
TCCCCCAGGACGA
>KF662118.1_Cavenderia_aureostipes_Thai2C1
GTATTAGGACAACCAATTGATGGAGGAGTAGAATT--TAAAGATACAAAAACAAC-----
--AAGATTAATTAGTTTAGAAAGAAAAGCACCAGGAATTGTAACAAGAAAATCCGTACAT
GAATCAATGTTAACAGGAGTAAAAATCGTAGATGCATTATTACCTATTGGAAGAGGACAA
CGTGAATTAATTATTGGAGATCGTCAAACAGGAAAATCTTCAGTAGCAATTGATGCAATT
TTAAATCAAA---AAACAA--ATAAAGATATTGTG-----TGTGTATATGTATCCGTAGG
ACAAAAAAAATCAACAATTAGAAGATTAGTAGAAATGTTAAATTTAAAAGGATCTTTAAA
ATATAGTATTGTAGTAGTATCAACAGCATCAGATGCATCACCATTACAATTCTTAGCACC
TTATACAGGATGTGCTATTGGAGAATATTTCAGAGATAAAGGAGAACATGCTTTAATTAT
TTATGATGATTTAAGTAAACATGCAGTAGCCTACCGTCAAATGTCTTTATTATTAAGACG
TCCACCAGGACGA
>KF662110.1_Cavenderia_stellata_SAB7B
GTATTAGGACAACCAATTGATGGAGGAGATGAATT--TAAAGATACCAAAACTAC-----
--AAGATTAATTAGTTTAGAAAGAAAAGCACCAGGAATTGTAACTAGAAAATCTGTACAT
GAATCAATGTTAACAGGAGTAAAAATTGTAGATGCATTATTACCAATAGGAAGAGGTCAA
CGTGAATTAATTATTGGAGATCGTCAAACAGGAAAATCTTCAGTAGCAATAGATGCAATA
TTAAACCAAA---AAACAA--ATAAAGATATTGTA-----TGTGTATATGTCTCTGTAGG
ACAAAAAAAATCTACAATAAGAAGATTAGTAGAAATGTTAAATAGTAAAGGATCTTTAAA
ATATAGTATTGTAGTAGTATCCACAGCATCAGATGCATCTCCTTTACAATTTTTAGCACC
ATATACAGGATGTGCAATTGGAGAATATTTCAGAGATAAAGGAGAACATGCATTAATTAT
TTATGATGATTTAAGTAAACATGCAGTAGCTTACCGTCAAATGTCCTTATTATTAAGACG
TCCACCAGGACGA
>KF662120.1_Cavenderia_aureostipes_OH396
GTATTAGGACAACCAATTGATGGAGGAGTAGAATT--TAAAGATACAAAAACAAC-----
--AAGATTAATTAGTTTAGAAAGAAAAGCACCAGGAATTGTAACAAGAAAATCAGTACAT
GAATCAATGTTAACAGGAGTAAAAATTGTAGATGCATTATTACCAATTGGAAGAGGACAA
CGTGAATTAATTATTGGAGATCGTCAAACAGGAAAATCTTCAGTAGCAATTGATGCAATT
TTAAACCAAA---AAACAA--ATAAAGATATTGTT-----TGTGTATATGTATCCGTAGG
GCAAAAAAAATCAACAATTAGAAGATTAGTCGAAATGTTAAATTTAAAAGGATCTTTAAA
ATATAGTATAGTAGTAGTATCAACAGCATCAGATGCATCCCCATTACAGTTCTTAGCACC
TTATACAGGATGTGCTATTGGAGAATATTTCAGAGATAAAGGAGAACATGCTTTAATTAT
TTATGATGATTTAAGTAAACATGCCGTAGCATACCGTCAAATGTCTTTATTATTAAGACG
TCCACCAGGACGA
>KF662109.1_Cavenderia_aureostipes_var._helvetia_HM594
GTATTAGGACAACCAATTGATGGAGGAGTAGAATT--CAAAGATACAAAAACAAC-----
--AAGATTAATCAGCTTAGAAAGAAAAGCACCAGGAATTGTAACAAGAAAATCAGTACAT
GAATCTATGTTAACAGGAGTAAAAATCGTAGATGCCTTATTACCAATTGGAAGAGGACAA
CGTGAATTAATCATTGGGGATCGTCAAACAGGAAAATCATCAATAGCCATCGATGCAATA
TTAAACCAAA---GAACAA--ATAAAGATATTATT-----TGTGTATATGTATCAGTAGG
ACAAAAAAAAGCAACAATTAGAAGATTAGTAGCAATGTTAAATATAAAAGGAGCTTTAAA
ATATAGTATAATCGTAGTATCAACAGCATCAGATGCATCACCATTACAATTCTTAGCACC
TTATACAGGATGTGCAATTGGAGAATATTTCAGAGATAAAGGAGAACACGCATTAATTAT
ATATGATGATTTAAGTAAACATGCAGTAGCATATCGTCAAATGTCTTTATTATTAAGACG

TCCACCAGGACGA
>KF662100.1_Cavenderia_aureostipes_var._helvetia_52A1
GTATTAGGACAACCAATTGATGGAGGAGTAGAATT--CAAAGATACAAAAACAAC-----
--AAGATTAATCAGCTTAGAAAGAAAAGCACCAGGAATTGTAACAAGAAAATCAGTACAT
GAATCTATGTTAACAGGAGTAAAAATCGTAGATGCCTTATTACCAATTGGAAGAGGACAA
CGTGAATTAATCATTGGGGATCGTCAAACAGGAAAATCATCAATAGCTATCGATGCAATA
TTAAACCAAA---GAACAA--ATAAAGATATTATT-----TGTGTATATGTATCAGTAGG
ACAAAAAAAAGCAACAATTAGAAGATTAGTAGCAATGTTAAATATAAAAGGAGCTTTAAA
ATATAGTATAATCGTAGTATCAACAGCATCAGATGCATCACCATTACAATTCTTAGCACC
TTATACAGGATGTGCAATTGGAGAATATTTCAGAGATAAAGGAGAACACGCATTAATTAT
ATATGATGATTTAAGTAAACATGCAGTAGCATATCGTCAAATGTCTTTATTATTAAGACG
TCCACCAGGACGA
>KF662099.1_Cavenderia_aureostipes_var._helvetia_HM593
GTATTAGGACAACCAATTGATGGAGGAGTAGAATT--CAAAGATACAAAAACAAC-----
--CAGATTAATCAGCTTAGAAAGAAAAGCACCAGGAATTGTAACAAGAAAATCAGTACAT
GAATCTATGTTAACAGGAGTAAAAATCGTAGATGCCTTATTACCAATTGGAAGAGGACAA
CGTGAATTAATCATTGGGGATCGTCAAACAGGAAAATCATCAATAGCTATCGATGCAATA
TTAAACCAAA---GAACAA--ATAAAGATATTATT-----TGTGTATATGTATCAGTAGG
ACAAAAAAAAGCAACAATTAGAAGATTAGTAGCAATGTTAAATATAAAAGGAGCTTTAAA
ATATAGTATAATCGTAGTATCAACAGCATCAGATGCATCACCATTACAATTCTTAGCACC
TTATACAGGATGTGCAATTGGAGAATATTTCAGAGATAAAGGAGAACACGCATTAATTAT
ATATGATGATTTAAGTAAACATGCAGTAGCATATCGTCAAATGTCTTTATTATTAAGACG
TCCACCAGGACGA
>KF662115.1_Cavenderia_aureostipes_CR6B1
---AAAACAAC-----
--AAGATTAATTAGTTTAGAAAGAAAAGCACCAGGAATTGTAACAAGAAAATCCGTACAT
GAATCTATGTTAACAGGAGTAAAAATTGTAGATGCATTATTACCAATAGGAAGAGGACAA
CGTGAATTAATTATTGGAGATCGTCAAACAGGAAAATCTTCAGTAGCAATTGATGCAATT
TTAAATCAAA---AAACAA--ATAAAGATATTGTA-----TGTGTATATGTATCAGTAGG
ACAAAAAAAATCAACAATTAGAAGATTAGTAGAAATGTTAAATTTAAAAGGATCTTTAAA
ATATAGTATTGTAGTAGTATCAACAGCATCAGATGCATCACCATTACAATTCTTAGCACC
ATATACAGGATGTGCTATTGGAGAATATTTCAGAGATAAAGGAGAACATGCATTAATTAT
TTATGATGATTTAAGTAAACATGCAGTAGCATATCGTCAAATGTCTTTATTATTAAGACG
TCCTCCAGGACGA
>KF662111.1_Cavenderia_bifurcata_UK5
GTATTAGGACAACCAATTGATGGAGGTGTAGAAAT--TAAAGATACAAAAACAAC-----
--AAGAGTTATAAGTTTAGAGAGAAAAGCTCCAGGAATTGTAACTAGAAAATCTGTACAT
GAGTCTATGTTAACAGGAGTAAAAATTGTAGATGCATTATTACCTATAGGAAGAGGACAA
CGTGAATTAATTATTGGAGATCGTCAAACAGGAAAATCTTCAGTAGCTATTGATGCAATT
TTAAATCAAA---AAACAA--ATAAAGATATAATA-----TGTGTATATGTATCTGTAGG
ACAAAAAAAAGCAACAATTAGAAGATTAGTAGAAATGTTAAAATTAAAAGGATCATTAAA
ATATAGTATTGTAGTAGTATCAACAGCATCTGATGCATCACCATTACAATTTTTAGCACC
ATATACAGGATGTGCTATTGGAGAATATTTTAGAGATAAAGGAGAACATGCATTAATTAT
TTATGATGATTTAAGTAAACATGCTGTAGCTTACCGTCAAATGTCTTTATTATTAAGACG
TCCACCAGGACGA
>EU275727.1_Cavenderia_fasciculata_SH3
GTATTAGGACAACCAATTGATGGAGGAGTCGCATT--TAAAGATACAAAAACAAC-----
--AAGATTAATCAGTTTAGAAAGAAAAGCCCCAGGAATTGTAACTAGAAAATCTGTACAT
GAGTCAATGTTAACAGGAGTAAAAATTGTTGATGCATTATTACCAATTGGAAGAGGACAA
CGTGAATTAATTATTGGAGATCGTCAAACAGGTAAATCTTCAATTGCAATTGATGCAATC
TTAAATCAAA---AAACAA--ACAAAGATATTGTA-----TGTGTATACGTATCCGTAGG
ACAAAAAAAATCAACAATTAGAAGATTAGTTGAAATGTTAAATGTTAAAGGTTCTTTAAA
ATATAGTATTGTAGTAGTATCAACAGCGTCTGATGCTTCACCTTTACAGTTTTTAGCACC
TTATACAGGATGTGCCATTGGAGAATATTTTAGAGATAAAGGAGAACATGCTTTAATTAT
TTATGACGATTTAAGTAAACATGCCGTAGCATACCGTCAAATGTCTTTATTATTAAGACG
TCCACCAGGAAGA
>KF662084.1_Cavenderia_aureostipes_KAL7A
GTATTAGGACAACCAATCGATGGAGGAGTTGCATT--TAAAGATACAAAAACAAC-----
--AAGATTAATCAGTTTAGAAAGAAAAGCACCAGGAATTGTAACTAGAAAATCTGTACAT
GAGTCAATGTTAACAGGAGTAAAAATTGTTGATGCATTATTACCAATTGGAAGAGGACAA
CGTGAATTAATTATTGGAGATCGTCAAACAGGTAAATCTTCAATTGCAATTGATGCAATC
TTAAATCAAA---AAACAA--ATAAAGATATTGTA-----TGTGTATACGTATCCGTAGG
ACAAAAAAAATCAACAATTAGAAGATTAGTTGAAATGTTAAATGTTAAAGGTTCTTTAAA
ATATAGTATTGTAGTAGTATCAACAGCGTCTGATGCATCTCCTTTACAATTTTTAGCACC
ATATACAGGATGTGCAATCGGAGAATATTTTAGAGATAAAGGAGAACATGCTTTAATTAT
TTATGATGATTTAAGTAAACATGCCGTAGCATACCGTCAAATGTCTTTATTATTAAGACG
TCCTCCAGGACGA
>KF662082.1_Cavenderia_aureostipes_Chile10B1
GTATTAGGACAACCAATCGATGGAGGAGTTGCATT--TAAAGATACAAAAACAAC-----
--AAGATTAATCAGTTTAGAAAGAAAAGCACCAGGAATTGTAACTAGAAAATCTGTACAT
GAGTCAATGTTAACAGGAGTAAAAATTGTTGATGCATTATTACCAATTGGAAGAGGACAA
CGTGAATTAATTATTGGAGATCGTCAAACAGGTAAATCTTCAATTGCAATTGATGCAATC
TTAAATCAAA---AAACAA--ATAAAGATATTGTA-----TGTGTATACGTATCCGTAGG

ACAAAAAAAATCAACAATTAGAAGATTAGTTGAAATGTTAAATGTTAAAGGTTCTTTAAA
ATATAGTATTGTAGTAGTCTCATCAGCGTCTGATGCATCACCTTTACAATTTTTAGCACC
ATATACAGGATGTGCAATCGGAGAATATTTTAGAGATAAAGGAGAACATGCTTTAATTAT
TTATGATGATTTAAGTAAACATGCCGTAGCATACCGTCAAATGTCTTTATTATTAAGACG
TCCTCCAGGACGA
>KF662083.1_Cavenderia_aureostipes_GSE7B
GTATTAGGACAACCAATCGATGGCGGAGTTGCATT--TAAAGATACAAAAACAAC-----
--AAGATTAATCAGTTTAGAAAGAAAAGCACCAGGAATTGTAACTAGAAAATCTGTACAT
GAGTCAATGTTAACAGGAGTAAAAATTGTTGATGCATTATTACCAATTGGAAGAGGACAA
CGTGAATTAATTATTGGAGATCGTCAAACAGGTAAATCTTCAATTGCAATTGATGCAATC
TTAAATCAAA---AAACAA--ATAAAGATATTGTA-----TGTGTATACGTATCCGTAGG
ACAAAAAAAATCAACAATTAGAAGATTAGTTGAAATGTTAAATGTTAAAGGTTCTTTAAA
ATATAGTATTGTAGTAGTATCAACAGCGTCTGATGCATCTCCTTTACAATTTTTAGCACC
ATATACAGGATGTGCAATCGGAGAATATTTTAGAGATAAAGGAGAACATGCTTTAATTAT
TTATGATGATTTAAGTAAACATGCCGTAGCATACCGTCAAATGTCTTTATTATTAAGACG
TCCTCCAGGACGA
>KF662073.1_Cavenderia_mexicana_TF4B1
GTATTAGGACAACCAATTGATGGAGGAGTTGAAAT--TAAAGATACAAAAACTAC-----
--TAGATTAATTAGTTTAGAAAGAAAAGCTCCAGGAATTGTAACTAGAAAATCTGTACAT
GAATCTATGTTAACTGGAGTAAAAATTGTTGATGCATTATTACCAATTGGAAGAGGACAA
CGTGAATTAATTATTGGAGATCGTCAAACAGGAAAATCTTCTATTGCAATTGATTCAATT
TTAAATCAAA---AAACTA--ATAAAGATATTGTA-----TGTGTTTATGTTTCTGTAGG
ACAAAAAAAATCAACAATTAGAAGATTAGTAGAAATGTTAAATATGAAAGGTTCTTTAAA
ATATAGTATTGTAGTTGTATCCACAGCTTCTGATGCATCTCCTTTACAATTTTTAGCACC
TTATACAGGATGTGCAATTGGAGAATATTTTAGAGATAAAGGAGAACATGCTTTAATCAT
TTATGATGATTTAAGTAAACATGCAGTAGCTTACCGTCAAATGTCTTTATTATTAAGACG
TCCACCAGGACGA
>KF662080.1_Cavenderia_delicata_TNS_266
GTATTAGGACAACCAATCGATGGAGGAGTTGCATT--TAAAGATACAAAAACAAC-----
--AAGATTAATCAGTTTAGAAAGAAAAGCACCAGGAATTGTAACTAGAAAATCTGTACAT
GAGTCAATGTTAACAGGAGTAAAAATTGTTGATGCATTATTACCAATTGGAAGAGGACAA
CGTGAATTAATTATTGGAGATCGTCAAACAGGTAAATCTTCAATTGCAATTGATGCAATC
TTAAATCAAA---AAACAA--ATAAAGATATTGTA-----TGTGTATACGTATCCGTAGG
ACAAAAAAAATCTACAATTAGAAGATTAGTTGAAATGTTAAATGTTAAAGGTTCTTTAAA
ATATAGTATTGTAGTAGTATCAACAGCGTCTGATGCGTCACCTTTACAATTTTTAGCACC
TTATACAGGATGTGCAATCGGAGAATATTTTAGAGATAAAGGAGAACATGCTTTAATTAT
CTATGATGATTTAAGTAAACATGCCGTAGCATACCGTCAAATGTCTTTATTATTAAGACG
TCCTCCAGGACGA
>KF662127.1_Cavenderia_aureostipes_BP7B
GTATTAGGACAACCAATTGATGGAGGAGTAGAATT--TAAAGATACTAAAACTAC-----
--TAGATTAATTAGTTTAGAAAGAAAAGCACCAGGAATTGTAACCAGAAAATCTGTACAT
GAATCTATGTTAACAGGAGTAAAAATTGTAGATGCATTATTACCAATTGGTAGAGGACAA
CGTGAGTTAATTATTGGAGATCGTCAAACAGGAAAATCTTCAGTAGCAATTGATGCAATC
TTAAATCAAA---AAACAA--ACAAAGATATTGTT-----TGTGTATATGTATCCGTAGG
ACAAAAAAAATCAACAATTAGAAGATTAGTTGAAATGTTAAATATGAAAGGATCTTTAAA
ATATAGTATTGTAGTAGTATCCACAGCATCAGATGCTTCTCCATTACAATTTTTAGCACC
TTATACAGGATGTGCTATTGGAGAATATTTTAGAGATAAAGGAGAACATGCATTAATTAT
TTATGATGATTTAAGTAAACATGCTGTTGCTTACCGTCAAATGTCTTTATTATTAAGACG
TCCTCCAGGACGA
>KF662094.1_Cavenderia_aureostipes_Got5B4
GTATTAGGACAACCAATTGATGGAGGAGTCGCATT--TAAAGATACAAAAACAAC-----
--AAGATTAATCAGTTTAGAAAGAAAAGCCCCAGGAATTGTAACTAGAAAATCTGTACAT
GAGTCAATGTTAACAGGAGTAAAAATTGTTGATGCATTATTACCAATTGGAAGAGGACAA
CGTGAATTAATTATTGGAGATCGTCAAACAGGTAAATCTTCAATTGCAATTGATGCAATC
TTAAATCAAA---AAACAA--ACAAAGATATTGTA-----TGTGTATACGTATCCGTAGG
ACAAAAAAAATCAACAATTAGAAGATTAGTTGAAATGTTAAATGTTAAAGGTTCTTTAAA
ATATAGTATTGTAGTAGTATCAACAGCGTCTGATGCTTCACCTTTACAGTTTTTAGCACC
ATATACAGGATGTGCCATTGGAGAATATTTTAGAGATAAAGGAGAACATGCTTTAATTAT
TTATGACGATTTAAGTAAACATGCAGTAGCATACCGTCAAATGTCTTTATTATTAAGACG
TCCACCAGGACGA
>KF662081.1_Cavenderia_antarctica_NZ43B
GTATTAGGACAACCAATCGATGGAGGAGTTGCATT--TAAAGATACAAAAACAAC-----
--AAGATTAATCAGTTTAGAAAGAAAAGCACCAGGAATTGTAACTAGAAAATCTGTACAT
GAGTCAATGTTAACAGGAGTAAAAATTGTTGATGCCTTATTACCAATTGGAAGAGGACAA
CGTGAATTAATTATTGGAGATCGTCAAACAGGTAAATCTTCAATTGCAATTGATGCAATC
TTAAATCAAA---AAACAA--ATAAAGATATTGTA-----TGTGTATACGTATCAGTAGG
ACAAAAAAAATCAACAATTAGAAGATTAGTTGAAATGTTAAATGTTAAAGGTTCTTTAAA
ATATAGTATTGTAGTAGTATCATCAGCGTCTGATGCATCTCCTTTACAATTTTTAGCACC
ATATACAGGATGTGCAATCGGAGAATATTTTAGAGATAAAGGAGAACATGCTTTAATTAT
TTATGATGATTTAAGTAAACATGCCGTAGCCTACCGTCAAATGTCTTTATTATTAAGACG
TCCTCCAGGACGA
>KF662124.1_Cavenderia_aureostipes_LR2
GTATTAGGACAACCAATTGATGGAGGTGTAGAATT--TAAAGATACTAAAACTAC-----

--AAGATTAATTAGTTTAGAAAGAAAAGCACCAGGAATTGTAACAAGAAAATCTGTACAT
GAATCTATGTTAACAGGAGTAAAAATCGTTGATGCATTATTACCTATAGGTAGAGGACAA
CGTGAATTAATTATCGGAGATCGTCAAACAGGAAAATCTTCCGTAGCAATCGATGCAATT
TTAAATCAAA---AAACAA--ACAAAGATATTGTA-----TGTGTCTATGTATCCGTTGG
ACAAAAAAAATCAACAATTAGAAGATTAGTTGAAATGTTAAACATGAAAGGATCTTTAAA
ATATAGTATTGTAGTAGTATCAACCGCATCAGATGCTTCACCATTACAATTTTTAGCACC
TTATACAGGATGTGCAATTGGAGAATATTTCAGAGATAAAGGGGAACATGCATTAATTAT
TTATGATGATTTAAGTAAACATGCTGTAGCTTACCGTCAAATGTCTTTATTATTAAGACG
TCCTCCAGGACGA
>KF662095.1_Cavenderia_aureostipes_AKL43C
GTATTAGGACAACCAATTGATGGAGGAGTCGCATT--TAAAGATACAAAAACAAC-----
--AAGATTAATCAGTTTAGAAAGAAAAGCCCCAGGAATTGTAACTAGAAAATCTGTACAT
GAGTCAATGTTAACAGGAGTAAAAATTGTTGATGCATTATTACCAATTGGAAGAGGACAA
CGTGAATTAATTATTGGAGATCGTCAAACAGGTAAATCTTCAATTGCAATTGATGCAATC
TTAAATCAAA---AAACAA--ACAAAGATATTGTA-----TGTGTATACGTATCCGTAGG
ACAAAAAAAATCAACAATTAGAAGATTAGTTGAAATGTTAAATGTTAAAGGTTCTTTAAA
ATATAGTATTGTAGTAGTATCAACAGCGTCTGATGCTTCACCTTTACAGTTTTTAGCACC
ATATACAGGATGTGCCATTGGAGAATATTTTAGAGATAAAGGAGAACATGCTTTAATTAT
TTATGACGATTTAAGTAAACATGCCGTAGCATACCGTCAAATGTCTTTATTATTAAGACG
TCCACCAGGACGA
>KF662130.1_Cavenderia_aureostipes_KP3
GTATTAGGACAACCAATTGATGGGGGGGTAGAATT--TAAAGATACTAAAACTAC-----
--TAGATTAATTAGTTTAGAAAGAAAAGCACCAGGAATTGTAACAAGAAAATCTGTACAT
GAATCTATGTTAACTGGGGTAAAAATTGTAGATGCATTATTACCAATTGGTAGAGGTCAA
CGTGAGTTAATTATTGGAGATCGTCAAACAGGAAAATCTTCTGTAGCAATTGATGCAATT
TTAAATCAAA---AAACTA--ATAAAGATATTGTA-----TGTATATATGTATCCGTAGG
ACAAAAAAAATCAACAATTAGAAGATTAGTTGAAATGTTAAATATGAAAGGATCTTTAAA
ATATAGTATTGTAGTAGTATCTACAGCATCTGATGCTTCTCCATTACAATTTTTAGCACC
ATATACAGGATGTTCTATTGGAGAATATTTTAGAGATAAAGGGGAACATGCATTAATTAT
TTATGATGATTTAAGTAAACATGCTGTTGCTTACCGTCAAATGTCTTTATTATTAAGACG
TCCACCAGGACGA
>KF662092.1_Cavenderia_aureostipes_Got9A1
GTATTAGGACAACCAATTGATGGAGGAGTCGCATT--TAAAGATACAAAAACAAC-----
--AAGATTAATCAGTTTAGAAAGAAAAGCCCCAGGAATTGTAACTAGAAAATCTGTACAT
GAGTCAATGTTAACAGGAGTAAAAATTGTTGATGCATTATTACCAATTGGAAGAGGACAA
CGTGAATTAATTATTGGAGATCGTCAAACAGGTAAATCTTCAATTGCAATTGATGCAATC
TTAAATCAAA---AAACAA--ACAAAGATATTGTA-----TGTGTATACGTATCCGTAGG
ACAAAAAAAATCAACAATTAGAAGATTAGTTGAAATGTTAAATGTTAAAGGTTCTTTAAA
ATATAGTATTGTAGTAGTATCAACAGCGTCTGATGCTTCACCTTTACAGTTTTTAGCACC
TTATACAGGATGTGCCATTGGAGAATATTTTAGAGATAAAGGAGAACATGCTTTAATTAT
TTATGACGATTTAAGTAAACATGCCGTAGCATACCGTCAAATGTCTTTATTATTAAGACG
TCCACCAGGACGA
>KF662076.1_Cavenderia_fasciculata_HM595
GTATTAGGACAACCAATCGATGGAGGAGTTGCATT--TAAAGATACAAAAACAAC-----
--AAGATTAATCAGTTTAGAAAGAAAAGCCCCAGGAATTGTAACTAGAAAATCTGTACAT
GAGTCAATGTTAACAGGAGTAAAAATTGTTGATGCATTATTACCAATTGGAAGAGGACAA
CGTGAATTAATTATTGGAGATCGTCAAACAGGTAAATCTTCAATTGCAATTGATGCCATC
TTAAATCAAA---AAACAA--ACAAAGATATTGTC-----TGTGTATACGTATCAGTAGG
ACAAAAAAAATCAACAATCAGAAGATTAGTAGAAATGTTAAATGTTAAAGGTTCTTTAAA
ATATAGTATTGTAGTTGTATCAACAGCGTCTGATGCTTCACCTTTACAATTTTTAGCACC
TTATACAGGATGTGCCATTGGAGAATATTTCAGAGATAAAGGAGAACATGCTTTAATTAT
TTATGATGATTTAAGTAAACATGCAGTAGCTTACCGTCAAATGTCTTTATTATTAAGACG
TCCACCAGGACGA
>KF662133.1_Cavenderia_aureostipes_I3
GTATTAGGACAACCAATTGATGGGGGCGTAGAATT--TAAAGATACTAAAACTAC-----
--TAGATTAATTAGTTTAGAAAGAAAAGCACCAGGAATTGTAACAAGAAAATCTGTACAT
GAATCTATGTTAACTGGGGTAAAAATTGTAGATGCATTATTACCAATTGGTAGAGGTCAA
CGTGAGTTAATTATTGGAGATCGTCAAACAGGAAAATCTTCTGTAGCAATTGATGCAATT
TTAAATCAAA---AAACTA--ATAAAGATATTGTA-----TGTATATACGTATCCGTAGG
ACAAAAAAAATCAACAATTAGAAGATTAGTTGAAATGTTAAATATGAAAGGATCTTTAAA
ATATAGTATTGTAGTAGTATCTACAGCATCTGATGCTTCTCCATTACAATTTTTAGCACC
ATATACAGGATGTTCTATTGGAGAATATTTTAGAGATAAAGGGGAACATGCATTAATTAT
TTATGATGATTTAAGTAAACATGCTGTTGCTTACCGTCAAATGTCTTTATTATTAAGACG
TCCACCAGGACGA
>KF662131.1_Cavenderia_aureostipes_OH110_BW6
GTATTAGGACAACCAATTGATGGGGGAGTAGAATT--TAAAGATACTAAAACTAC-----
--TAGATTAATTAGTTTAGAAAGAAAAGCACCAGGAATTGTAACAAGAAAATCTGTACAT
GAATCTATGTTAACTGGGGTAAAAATTGTAGATGCATTATTACCAATTGGTAGAGGTCAA
CGTGAGTTAATTATTGGGGATCGTCAAACAGGAAAATCTTCTGTAGCAATTGATGCAATT
TTAAATCAAA---AAACTA--ATAAAGATATTGTA-----TGTATATACGTATCCGTAGG
ACAAAAAAAATCAACAATTAGAAGATTAGTTGAAATGTTAAATATGAAAGGATCTTTAAA
ATATAGTATTGTAGTAGTATCTACAGCATCTGATGCTTCTCCATTACAATTTTTAGCACC
ATATACAGGATGTTCTATTGGAGAATATTTTAGAGATAAAGGGGAACATGCATTAATTAT

TTATGATGATTTAAGTAAACATGCTGTTGCTTACCGTCAAATGTCTTTATTATTAAGACG
TCCACCAGGACGA
>KF662077.1_Cavenderia_fasciculata_Nor84E
GTATTAGGACAACCAATCGATGGAGGAGTTGCATT--TAAAGATACAAAAACAAC-----
--AAGATTAATCAGTTTAGAAAGAAAAGCCCCAGGAATTGTAACTAGAAAATCTGTACAT
GAGTCAATGTTAACAGGAGTAAAAATTGTTGATGCATTATTACCAATTGGAAGAGGACAA
CGTGAATTAATTATTGGAGATCGTCAAACAGGTAAATCTTCAATTGCAATTGATGCCATC
TTAAATCAAA---AAACAA--ACAAAGATATTGTC-----TGTGTATACGTATCAGTAGG
ACAAAAAAAAATCAACAATCAGAAGATTAGTTGAAATGTTAAATGTTAAAGGTTCTTTAAA
ATATAGTATTGTAGTTGTATCAACAGCGTCTGATGCTTCACCTTTACAATTTTTAGCACC
TTATACAGGATGTGCCATTGGAGAATATTTCAGAGATAAAGGAGAACATGCTTTAATTAT
TTATGATGATTTAAGTAAACATGCAGTAGCTTACCGTCAAATGTCTTTATTATTAAGACG
TCCACCAGGACGA
>KF662088.1_Cavenderia_aureostipes_OH438
GTATTAGGACAACCAATCGATGGAGGAGTTGCATT--TAAAGATACAAAAACAAC-----
--AAGATTAATCAGTTTAGAAAGAAAAGCACCAGGAATTGTAACTAGAAAATCTGTACAT
GAGTCAATGTTAACAGGAGTAAAAATTGTTGATGCATTATTACCAATTGGAAGAGGACAA
CGTGAATTAATTATTGGAGATCGTCAAACAGGTAAATCTTCAATTGCAATTGATGCAATC
TTAAATCAAA---AAACAA--ATAAAGATATTGTA-----TGTGTATACGTATCCGTAGG
ACAAAAAAAAATCAACAATTAGAAGATTAGTTGAAATGTTAAATGTTAAAGGTTCTTTAAA
ATATAGTATTGTAGTAGTATCAACAGCGTCTGATGCATCTCCTTTACAATTTTTAGCACC
ATATACAGGATGTGCAATCGGAGAATATTTTAGAGATAAAGGAGAACATGCTTTAATTAT
TTATGATGATTTAAGTAAACATGCCGTAGC------------------------------

>KF662074.1_Cavenderia_medusoides_OH592
GTATTAGGACAACCAATTGATGGAGGAGTTGAAAT--TAAAAATTCTAAAAATAC-----
--AAGATTAATTAGTTTAGAAAGAAAAGCACCAGGAATTGTAACAAGAAAATCTGTACAT
GAATCTATGTTAACTGGAGTAAAAATCGTTGATGCTTTATTACCTATTGGAAGAGGGCAA
CGTGAATTAATTATTGGAGATCGTCAAACTGGAAAATCTTCAGTAGCTATTGATGCAATT
TTAAATCAAA---AAACTA--ATAAAGATATTATT-----TGTGTTTATGTATCTGTAGG
GCAAAAAAAAATCAACAATTAGAAGATTAGTAGAAATGTTAAATATGAGAGGTTCTTTAAA
ATATAGTATTGTAGTAGTATCTACAGCATCAGATGCTTCTCCATTACAATTTTTAGCACC
ATATACAGGATGTGCTATTGGAGAATATTTTAGAGATAAAGGAGAACATGCTTTAATTAT
TTATGATGATTTAAGTAAACATGCAGTAGCTTATCGTCAAATGTCTTTATTATTAAGACG
TCCACCAGGACGA
>KF662098.1_Cavenderia_aureostipes_var._helvetia_HM592
--GATACAAAAACAAC-----
--CAGATTAATCAGCTTAGAAAGAAAAGCACCAGGAATTGTAACAAGAAAATCAGTACAT
GAATCTATGTTATCAGGAGTAAAAATAGTTGATGCCTTTTTCCAATTGGAAGGGGGCAA
CGTGAATTAATCATTGGGGATCGTCAAACAGGAAAATCATCAATAGCTATCGATGCAATA
TTAAACCAAA---GAACAA--ATAAAGATATTATT-----TGTGTATATGTATCAGTAGG
ACAAAAAAAAGCAACAATTAGAAGATTAGTAGCAATGTTAAATATAAAAGGAGCTTTAAA
ATATAGTATAATCGTAGTATCAACAGCATCAGATGCATCACCATTACAATTCTTAGCACC
TTATACAGGATGTGCAATTGGAGAATATTTCAGAGATAAAGGAGAACACGCATTAATTAT
ATATGATGATTTAAGTAAACATGCAGTAGCATATCGTCAAATGTCTTTATTATTAAGACG
TCCACCAGGACGA
>KF662096.1_Cavenderia_exigua_KP94
---AC-----
--AAGATTAATTAGTTTAGAAAGAAAAGCACCAGGAATTGTAACTAGAAAATCTGTACAT
GAATCTATGTTAACAGGAGTTAAAATTGTTGATGCATTATTACCAATTGGAAGAGGACAA
CGTGAATTAATTATTGGAGATCGTCAAACAGGAAAGTCTTCAATTGCAATTGATGCAATT
CTTAACCAAA---AAACAA--ATAAAGATATTATT-----TGTGTCTATGTTTCCGTAGG
TCAAAAAAAAATCTACAATTAGAAGATTAGTAGAAATGTTAAATACAAAAGGATCTTTAAA
ATATAGTATTGTTGTAGTTTCTACTGCATCAGATGCATCTCCATTACAATTCTTAGCACC
TTATACAGGATGTGCTATTGGAGAATATTTCAGAGATAAAGGAGAACATGCTTTAATTAT
TTATGATGATTTAAGTAAACATGCTGTAGCATATCGTCAAATGTCTTTATTATTAAGACG
TCCACCAGGACGA
>KF662071.1_Cavenderia_parvispora_OS126
GTATTAGGACAACCAATTGATGGAGGAGTAGAGTT--TAAAGATACAAAAACTAC-----
--ACGTTTGATCAGTTTAGAAAGAAAAGCACCAGGAATTGTAACAAGAAAATCTGTACAT
GAATCAATGTTAACAGGAATAAAAATCGTAGATGCTTTGTTACCTATCGGAAGAGGTCAA
CGTGAATTAATTATTGGAGATCGTCAAACAGGAAAATCATCCATAGCGATTGATGCTATA
CTAAATCAAA---AAACAA--ATAAAGATATTATT-----TGTGTATATGTATCAGTTGG
ACAAAAAAAAATCCACAATACGAAGATTAGTAGAAATGTTAAATAATAAAGGATCTTTAAA
ATATAGTGTAATCGTTGTCTCAACAGCATCAGATGCATCACCATTACAATTTTTAGCACC
TTATACAGGATGTGCAATTGGTGAGTATTTTAGAGATAAAGGAGAACATGCTTTAATTAT
TTATGATGATTTAAGTAAACATGCAGTAGCCTACCGTCAAATGTCCTTATTATTAAGACG
TCCACCAGGACGA
>KF662072.1_Cavenderia_microspora_Hagiwara143
---ACAAC-----
--ACGTTTGATCAGTTTAGAAAGAAAAGCACCAGGAATTGTAACAAGAAAATCTGTACAC
GAATCAATGTTAACAGGAGTAAAAATTGTAGATGCTTTATTACCTATAGGAAGAGGTCAA
CGGGAATTAATCATTGGAGATCGTCAAACAGGAAAATCATCAATAGCTATTGATGCTATA

```
CTAAACCAAA---AAACAA--ATAAAGATATTATT-----TGTGTATATGTATCAGTAGG
ACAAAAAAAAATCCACAATAAGAAGATTAGTTGAAATGTTAAATAATAAAGGATCTTTAAA
ATATAGTGTAGTTGTAGTCTCAACAGCATCAGATGCATCACCATTACAATTTTTAGCACC
TTATACAGGATGTGCCATTGGTGAGTATTTCAGAGATAAAGGAGAACATGCTTTAATTAT
TTATGATGATTTAAGTAAACATGCTGTAGCATATCGTCAAATGTCCTTATTAT-------
-------------
>NC_002508.1_Physarum_polycephalum
TTATTAGGTCGTGTTGT-GATGGTATTGGTAATTTATCGATGGTGGTGAAACTATGCTTT
TGAGGAATATCTCAATGTGAACGTAAAGCTCCTGGTGTTAT-ACTCGTGAATCTGTTACT
GAA-CAATGTTAACTGGT-TATAAAATGTTGATTCTATGTTACCTAT-GGACGTGGTCAA
AGGGAACTTATTGTTGGTGATCGT-AAACAGGTAAAACTACTATTGCTAT-GATACTATT
CTTAATCAACGTTATACAATGAAGAAGATATTGATCTTATTGTGTGTATGTTGGAATTGG
TCAAAAAAAAAGTTCTATTTGAATATTCAAACTTACTTCAAAATAATAAAGCGCTC---A
TTATACTACTATTGTTGCTGCAACGCTGCACAAAGTGCCTCTCTT-CAGTTTATTGCTCC
ATATACAGGATGTGC-ATTGCTGAATTTTATCGTGAT-AAGGTGAACATGC-TTAATTAT
TTATGATGATTTAACAAAACATGCTGCTGCTTAT-GACAATTATCTTTAT-ATTAAGACG
CCCACCAGGACGT
```

Text S3. Alignment of *atp1* sequences on *Dictyostelium* tree

>KF662068.1_Dictyostelium_sp._TAS30A
GTATTAGGACAACCAATTGATGGATTAGGAGAATT--ACAAGATACAAAAACTAC-----
--AAGAGTAATGAGTGTAGAAAGAAAAGCACCAGGAATTGTAACAAGAAAATCAGTACAT
GAATCAATGTTAACAGGAGTAAAAATTGTAGATGCATTATTACCAATTGGAAGAGGACAA
AGGGAGTTAATCATTGGAGATAGACAAACAGGAAAATCAGCAATTGCAGTAGATGCAATC
TTAAATCAAC---AAGTAA--ATAAAGATATTGTA-----TGTATTTACGTAGCTGTAGG
ACAAAAAAAATCAACAGTAAGAAGATTAGTAGAAATGTTAAATACAAGAGGAGCATTAGA
ATATACAATCGTAGTAGTATCAACAGCATCAGATGCAGCACCATTACAATTCTTAGCACC
ATATACAGGATGTACAATTGGAGAGTATTTCAGAGATGAAGGAAAACATGCATTAATCGT
ATACGATGACTTAAGTAAACATGCAGTAGCATATAGACAAATGTCATTATTATTAAGAAG
ACCACCAGGACGA
>_MZ318391_Dictyostelium_robusticaule_5729_bai_2021_HMJAU_MR305
GTATTAGGACAACCAATTGATGGATTAGGAGAATT--ACAAGATACTAAAACAAC-----
--CAGAGTAATGAGTATTGAAAGAAAAGCACCAGGAATCGTAACAAGAAAATCAGTACAT
GAATCAATGTTAACTGGAGTAAAAATCGTAGATGCATTATTACCAATCGGAAGAGGACAA
AGGGAGTTAATTATTGGAGATAGACAAACAGGAAAATCAGCAATTGCAGTAGATGCAATC
TTAAATCAAC---AAGTAA--ATAAAGACATTGTA-----TGTATTTATGTAGCTGTAGG
ACAAAAAAAATCAACAGTAAGAAGATTAGTAGAAATGTTAAACACAAGAGGAGCTTTAGA
ATATACAATTGTAGTAGTATCAACAGCATCAGATGCAGCACCATTACAATTCTTAGCACC
ATATACAGGATGTACAATTGGAGAATATTTCAGAGATGCAGGAAAACATGCATTAATTGT
ATATGATGATTTAAGTAAACATGCAGTAGCATATAGACAAATGTCATTATTATTAAGAAG
ACCACCAGGAAGA
>MZ318392_Dictyostelium_robusticaule_5729_huang_2021_HMJAU_MR305
GTATTAGGACAACCAATTGATGGATTAGGAGAATT--ACAAGATACTAAAACAAC-----
--CAGAGTAATGAGTATTGAAAGAAAAGCACCAGGAATCGTAACAAGAAAATCAGTACAT
GAATCAATGTTAACTGGAGTAAAAATCGTAGATGCATTATTACCAATCGGAAGAGGACAA
AGGGAGTTAATTATTGGAGATAGACAAACAGGAAAATCAGCAATTGCAGTAGATGCAATC
TTAAATCAAC---AAGTAA--ATAAAGACATTGTA-----TGTATTTATGTAGCTGTAGG
ACAAAAAAAATCAACAGTAAGAAGATTAGTAGAAATGTTAAACACAAGAGGAGCTTTAGA
ATATACAATTGTAGTAGTATCAACAGCATCAGATGCAGCACCATTACAATTCTTAGCACC
ATATACAGGATGTACAATTGGAGAATATTTCAGAGATGCAGGAAAACATGCATTAATTGT
ATATGATGATTTAAGTAAACATGCAGTAGCATATAGACAAATGTCATTATTATTAAGAAG
ACCACCAGGAAGA
>MZ318393_Dictyostelium_mucoroides_5735_3_23_HMJAU_MR303
GTATTAGGACAACCAATTGATGGATTAGGAGAATT--ACAAGATACAAAAACTAC-----
--AAGAGTAATGAGTGTAGAAAGAAAAGCACCAGGAATTGTAACAAGAAAATCAGTACAT
GAATCAATGTTAACAGGAGTAAAAATTGTAGATGCATTATTACCAATTGGAAGAGGACAA
AGGGAATTAATTATTGGAGATAGACAAACAGGAAAATCAGCAATCGCAGTAGATGCAATC
TTAAATCAAC---AAGTAA--ATAAAGATATTGTA-----TGTATTTATGTAGCTGTAGG
ACAAAAAAAATCAACAGTAAGAAGATTAGTAGAAATGTTAAACACAAGAGGAGCATTAGA
GTATACAATTGTAGTAGTTTCAACAGCATCAGATGCAGCACCATTACAATTCTTAGCACC
ATATACAGGATGTACAATCGGAGAGTATTTCAGAGATGAAGGAAAACATGCATTAATCGT
ATACGATGACTTAAGTAAACATGCAGTAGCATATAGACAAATGTCATTATTATTAAGAAG
ACCACCAGGAAGA
>MZ318394_Dictyostelium_discoideum_5752_3_17_HMJAU_MR302
GTATTAGGACAACCAATCGATGGATTAGGCGAATT--AAAAGATACAAAAACAAC-----
--AAGAGTAATGAGTGTAGAAAGAAAAGCACCAGGAATAGTAACAAGAAAATCAGTACAT
GAATCAATGTTAACAGGAGTAAAAATAGTAGATGCATTATTACCAATTGGAAGAGGACAA
AGGGAGTTAATAATTGGAGATAGACAAACAGGAAAATCAGCAATAGCAGTAGATGCAATT
TTAAATCAAC---AAGTAA--ATAAAGATATTGTA-----TGTATATATGTAGCAGTTGG
ACAAAAAAAATCAACAGTTAGACGATTAGTAGAAATGTTAAATACAAAAGGAGCATTAGA
ATATACAATTGTCGTAGTATCAACAGCATCAGATGCAGCACCATTACAATTTTTAGCACC
ATATACAGGATGTACAATTGGAGAATATTTTAGAGATGAAGGAAAACATGCATTAATAGT
CTATGATGATTTAAGTAAACATGCAGTAGCATATCGACAAATGTCATTATTATTAAGAAG
ACCACCAGGAAGA
>MZ318395_Dictyostelium_discoideum_5751_1_23_HMJAU_MR302
GTATTAGGACAACCAATCGATGGATTAGGCGAATT--AAAAGATACAAAAACAAC-----
--AAGAGTAATGAGTGTAGAAAGAAAAGCACCAGGAATAGTAACAAGAAAATCAGTACAT
GAATCAATGTTAACAGGAGTAAAAATAGTAGATGCATTATTACCAATTGGAAGAGGACAA
AGGGAGTTAATAATTGGAGATAGACAAACAGGAAAATCAGCAATAGCAGTAGATGCAATT
TTAAATCAAC---AAGTAA--ATAAAGATATTGTA-----TGTATATATGTAGCAGTTGG
ACAAAAAAAATCAACAGTTAGACGATTAGTAGAAATGTTAAATACAAAAGGAGCATTAGA
ATATACAATTGTCGTAGTATCAACAGCATCAGATGCAGCACCATTACAATTTTTAGCACC
ATATACAGGATGTACAATTGGAGAATATTTTAGAGATGAAGGAAAACATGCATTAATAGT
CTATGATGATTTAAGTAAACATGCAGTAGCATATCGACAAATGTCATTATTATTAAGAAG
ACCACCAGGAAGA
>AB000109.1_Dictyostelium_discoideum_AX3
GTATTAGGACAACCAATCGATGGATTAGGAGAATT--AAAAGATACAAAAACAAC-----
--AAGAGTAATGAGTGTAGAAAGAAAAGCACCAGGAATAGTAACAAGAAAATCAGTACAT

GAATCAATGTTAACAGGAGTAAAAATAGTAGATGCATTATTACCAATTGGAAGAGGACAA
AGGGAATTAATAATTGGAGATAGACAAACAGGAAAATCAGCAATAGCAGTAGATGCGATT
TTAAATCAAC---AAGTAA--ATAAAGATATTGTA-----TGTATATATGTAGCAGTTGG
ACAAAAAAAATCAACAGTTAGACGATTAGTAGAAATGTTAAATACAAAAGGAGCATTAGA
ATATACAATTGTCGTAGTATCAACAGCATCAGATGCAGCACCATTACAATTTTTAGCACC
ATATACAGGATGTACAATTGGAGAATATTTTAGAGATGAAGGAAAACATGCATTAATAGT
ATATGATGATTTAAGTAAACATGCAGTAGCATATCGACAAATGTCATTATTATTAAGAAG
ACCACCAGGAAGA
>DQ336395.4_Dictyostelium_citrinum
GTATTAGGACAACCAATTGATGGATTAGGAGAATT--AAAAGATACTAAAACAAC-----
--AAGAGTAATGAGTGTAGAAAGAAAAGCACCAGGAATCGTAACACGAAAATCAGTACAT
GAATCAATGTTAACAGGAGTAAAAATGGTAGATGCATTATTACCAATTGGAAGAGGACAA
CGGGAATTAATTATTGGAGATCGACAAACCGGAAAATCAGCAATAGCAGTAGATGCAATT
TTAAACCAAC---AAGTAA--ATAAAGATATCGTA-----TGTATCTATGTAGCAGTTGG
ACAAAAAAAATCAACTGTACGACGATTAGTAGAAATGTTAAATACAAAAGGAGCATTAGA
ATATACAATTGTAGTAGTATCCACTGCATCAGATGCAGCACCATTACAATTTTTAGCACC
ATATACAGGATGTACAATTGGAGAATATTTTCGAGATGAAGGAAAACATGCATTAATTGT
TTATGATGATTTAAGTAAACATGCAGTAGCATATCGACAAATGTCTTTATTATTACGACG
ACCACCAGGACGA
>NC_002508.1_Physarum_polycephalum
TTATTAGGTCGTGTTGT-GATGGTATTGGTAATTTATCGATGGTGGTGAAACTATGCTTT
TGAGGAATATCTCAATGTGAACGTAAAGCTCCTGGTGTTAT-ACTCGTGAATCTGTTACT
GAA-CAATGTTAACTGGT-TATAAAATGTTGATTCTATGTTACCTAT-GGACGTGGTCAA
AGGGAACTTATTGTTGGTGATCGT-AAACAGGTAAAACTACTATTGCTAT-GATACTATT
CTTAATCAACGTTATACAATGAAGAAGATATTGATCTTATTGTGTGTATGTTGGAATTGG
TCAAAAAAAAAGTTCTATTTGAATATTCAAACTTACTTCAAAATAATAAAGCGCTC---A
TTATACTACTATTGTTGCTGCAACGCTGCACAAAGTGCCTCTCTT-CAGTTTATTGCTCC
ATATACAGGATGTGC-ATTGCTGAATTTTATCGTGAT-AAGGTGAACATGC-TTAATTAT
TTATGATGATTTAACAAAACATGCTGCTGCTTAT-GACAATTATCTTTAT-ATTAAGACG
CCCACCAGGACGT

Text S4. Alignment of *atp1* sequences on *Heterostelium* tree

>MZ318386_Heterostelium_recretum_5756_1_17_HMJAU_MR315
GTATTAGGACAACCAATCGATGGAAAAAACGAAAT--TAAAGAAACTAAAAATGC-----
--AAGATTAATTAGTTTAGAAAGAAAAGCTCCAGGTATTGTAACTAGAAAATCTGTACAT
GAATCTATGTTAACTGGAGTTAAAATTGTAGATGCATTATTACCTATTGGAAGAGGACAA
AGAGAGTTAATTATTGGAGATCGTCAAACAGGAAAATCTGCAATTGCAATTGACGCAATT
TTAAATCAAA---AAACAA--ATAAAGATATTATT-----TGTGTTTATGTTTCTGTAGG
ACAAAAAAAATCTACAATTAGAAGATTAGTAGAAATGTTAAATATGAGAGGAGCATTAGA
ATATAGTATTGTAGTAGTTTCTACTGCATCTGATGCATCTCCATTACAATTCTTAGCACC
ATATTCTGGATGTGCAATTGGAGAATTTTTTAGAGATAAAGGAGAACATGCTTTAATTAT
TTATGATGATTTAAGTAAACATGCTGTAGCTTATCGTCAAATGTCTTTATTATTAAGAAG
ACCACCAGGAAGA
>MZ318387_Heterostelium_pallidum_5759_2_23_HMJAU_MR314
GTATTAGGACAACCAATTGATGGAAAAAATGAAAT--TAAAGAAACTAAAAATGC-----
--AAGATTAATTAGTTTAGAAAGAAAAGCTCCAGGAATTGTAACAAGAAAATCTGTACAT
GAATCTATGTTAACAGGAGTAAAAATTGTAGATGCATTATTACCAATTGGAAGAGGACAA
AGGGAATTAATTATTGGAGATCGTCAAACAGGAAAATCTGCAATTGCAATTGATGCAATT
TTAAATCAAA---AAACAA--ATAAAGATATTATT-----TGTGTATATGTATCTGTAGG
ACAAAAAAAATCTACAATTAGAAGATTAGTAGAAATGTTAAATATGAAAGGAGCATTAGA
TTATAGTATCGTTGTAGTATCTACAGCATCTGATGCATCTCCATTACAATTCTTAGCACC
ATATTCAGGATGTGCTATTGGAGAATTCTTTAGAGATAAAGGAGAACATGCATTAATTAT
TTATGATGATTTAAGTAAACATGCAGTAGCATATCGTCAAATGTCTTTATTATTAAGAAG
ACCTCCAGGAAGA
>MZ318388_Heterostelium_pallidum_5759_1_23_HMJAU_MR314
GTATTAGGACAACCAATTGATGGAAAAAATGAAAT--TAAAGAAACTAAAAATGC-----
--AAGATTAATTAGTTTAGAAAGAAAAGCTCCAGGAATTGTAACAAGAAAATCTGTACAT
GAATCTATGTTAACAGGAGTAAAAATTGTAGATGCATTATTACCAATTGGAAGAGGACAA
AGGGAATTAATTATTGGAGATCGTCAAACAGGAAAATCTGCAATTGCAATTGATGCAATT
TTAAATCAAA---AAACAA--ATAAAGATATTATT-----TGTGTATATGTATCTGTAGG
ACAAAAAAAATCTACAATTAGAAGATTAGTAGAAATGTTAAATATGAAAGGAGCATTAGA
TTATAGTATCGTTGTAGTATCTACAGCATCTGATGCATCTCCATTACAATTCTTAGCACC
ATATTCAGGATGTGCTATTGGAGAATTCTTTAGAGATAAAGGAGAACATGCATTAATTAT
TTATGATGATTTAAGTAAACATGCAGTAGCATATCGTCAAATGTCTTTATTATTAAGAAG
ACCTCCAGGAAGA
>AY700145.1_Heterostelium_pallidum_CK8
GTATTAGGACAACCAATTGATGGAAAAAATGAAAT--TAAAGAAACTAAAAATGC-----
--AAGATTAATTAGTTTAGAAAGAAAAGCTCCAGGAATTGTAACAAGAAAATCTGTACAT
GAATCTATGTTAACAGGAGTAAAAATTGTAGATGCATTATTACCAATTGGAAGAGGACAA
AGGGAATTAATTATTGGAGATCGTCAAACAGGAAAATCTGCAATTGCAATTGATGCAATT
TTAAATCAAA---AAACAA--ATAAAGATATTATT-----TGTGTATATGTATCTGTAGG
ACAAAAAAAATCTACAATTAGAAGATTAGTAGAAATGTTAAATATGAAAGGAGCATTAGA
TTATAGTATCGTTGTAGTATCTACAGCATCTGATGCATCTCCATTACAATTCTTAGCACC
ATATTCAGGATGTGCTATTGGAGAATTCTTTAGAGATAAAGGAGAACATGCATTAATTAT
TTATGATGATTTAAGTAAACATGCAGTAGCATATCGTCAAATGTCTTTATTATTAAGAAG
ACCTCCAGGAAGA
>EU275726.1_Heterostelium_pallidum_PN500
GTATTAGGACAACCAATTGATGGAAAAAATGAAAT--TAAAGAAACTAAAAATGC-----
--AAGATTAATTAGTTTAGAAAGAAAAGCTCCAGGAATTGTAACTAGAAAGTCTGTACAT
GAATCTATGTTAACAGGAGTAAAAATTGTAGATGCATTATTACCTATTGGACGTGGACAA
CGTGAGTTAATTATTGGAGATCGTCAAACAGGAAAATCTGCGATTGCAATTGATGCTATT
TTAAATCAAA---AAACAA--ATAAAGATATTATT-----TGTGTATACGTATCTGTAGG
ACAAAAAAAATCTACAATTAGAAGATTAGTAGAAATGTTAAATATGAAAGGAGCTTTAGA
GTATAGTATTATAGTAGTTTCTACAGCATCTGATGCATCTCCTTTACAATTTTTAGCACC
ATATTCTGGATGTGCAATTGGAGAATTCTTCAGAGATAAAGGAGAACATGCTTTAATTAT
TTATGATGATTTAAGTAAACATGCTGTAGCATATCGTCAAATGTCTTTATTATTAAGAAG
ACCTCCAGGAAGA
>NC_002508.1_Physarum_polycephalum
TTATTAGGTCGTGTTGT-GATGGTATTGGTAATTTATCGATGGTGGTGAAACTATGCTTT
TGAGGAATATCTCAATGTGAACGTAAAGCTCCTGGTGTTAT-ACTCGTGAATCTGTTACT
GAA-CAATGTTAACTGGT-TATAAAATGTTGATTCTATGTTACCTAT-GGACGTGGTCAA
AGGGAACTTATTGTTGGTGATCGT-AAACAGGTAAAACTACTATTGCTAT-GATACTATT
CTTAATCAACGTTATACAATGAAGAAGATATTGATCTTATTGTGTGTATGTTGGAATTGG
TCAAAAAAAAAGTTCTATTTGAATATTCAAACTTACTTCAAAATAATAAAGCGCTC---A
TTATACTACTATTGTTGCTGCAACGCTGCACAAAGTGCCTCTCTT-CAGTTTATTGCTCC
ATATACAGGATGTGC-ATTGCTGAATTTTATCGTGAT-AAGGTGAACATGC-TTAATTAT
TTATGATGATTTAACAAAACATGCTGCTGCTTAT-GACAATTATCTTTAT-ATTAAGACG
CCCACCAGGACGT

References

1. Hagiwara H, Chien CY, Yeh ZY. 1992. Dictyostelid cellular slime molds of Taiwan. Bulletin of the National Science Museum, Tokyo, Series B 18:39–52.
2. Yeh ZY, Chien CY. 1983. Cellular slime molds in Taiwan (I): four newly recorded species. Biological Bulletin of National Taiwan Normal University 18:69–86.
3. Fan YC, Chen JW, Yeh ZY. 2002. Notes on dictyostelid cellular slime molds of Taiwan (I): *Dictyostelium minutum* and *Dictyostelium clavatum*. Taiwania 47:31–36.
4. An X, Liu P, Li Y. 2018. The life cycle of *Dictyostelium firmibasis*. Mycosystema 37:516–521 (in Chinese).
5. Hagiwara H, Yeh ZY, Chien CY. 1985. *Dictyostelium macrocephalum*, a new dictyostelid cellular slime mold from Taiwan. Bulletin of the National Science Museum, Tokyo, Series B 11:103–108.
6. Liu P, Li Y. 2014. Dictyostelids from Jilin Province, China. I. Phytotaxa 183:279–283.
7. Yeh ZY. 2003. Biodiversity inventory of dictyostelid cellular slime molds in Taiwan. Mycotaxon 86:103–110.
8. Fan YC, Yeh ZY. 2001. Notes on a dictyostelid cellular slime mold new to Taiwan. BioFormosa 36:43–46.
9. Liu P, Zhang SH, Zhou XY, Zhao JJ, Li Y. 2019. Two *Heterostelium* species newly recorded from China. Mycotaxon 134:353–357.
10. He XL, Li Y. 2008. Three new records of dictyostelids in China. Mycosystema 27:532–537.
11. Lin SP, Yeh ZY. 1999. Six species of dictyostelid cellular slime molds isolated from YangMing Shan district of Taipei. Biological Bulletin of National Taiwan Normal University 34:69–80 (in Chinese).
12. Hsu SL, Fan YC, Ma MS, Chou HM, Yeh ZY. 2001. *Dictyostelium delicatum*, a new record of dictyostelid cellular slime molds to Taiwan. Taiwania 46:199–203.
13. Yeh ZY, Chen MJ. 2004. Notes on dictyostelid cellular slime molds from Taiwan (2): *Dictyostelium exiguum* and its ITS-5.8S rDNA sequences. Mycotaxon 89:489–496.
